# SPARC overexpression in allogeneic adipose-derived mesenchymal stem cells in dog dry eye model induced by benzalkonium chloride

**DOI:** 10.1186/s13287-024-03815-z

**Published:** 2024-07-02

**Authors:** Chenchen Li, Balun Li, Miao Han, Hongkai Tian, Jiaqi Gao, Dongyao Han, Zixi Ling, Yuanxiang Jing, Na Li, Jinlian Hua

**Affiliations:** College of Veterinary Medicine, Shaanxi Centre of Stem Cells Engineering & Technology, Northwest A&F University, Yangling, Shaanxi 712100 China

**Keywords:** Adipose-derived mesenchymal stem cells (ADMSC), Secreted protein acidic and rich in cysteine (SPARC), Keratoconjunctivitis (KCS), Corneal epithelial cell (HCECs), Mesenchymal stem cells (MSC)

## Abstract

**Background:**

Nowadays, companion and working dogs hold significant social and economic importance. Dry eye, also known as dry keratoconjunctivitis (KCS), a common disease in ophthalmology, can readily impact a dog’s working capacity and lead to economic losses. Although there are several medications available for this disease, all of them only improve the symptoms on the surface of the eye, and they are irritating and not easy to use for long periods of time. Adipose-derived mesenchymal stem cells (ADMSC) are promising candidates for tissue regeneration and disease treatment. However, long-term in vitro passaging leads to stemness loss of ADMSC. Here, we aimed to use ADMSC overexpressing Secreted Protein Acidic and Rich in Cysteine (SPARC) to treat 0.25% benzalkonium chloride-treated dogs with dry eye to verify its efficacy. For in vitro validation, we induced corneal epithelial cell (HCECs) damage using 1 µg/mL benzalkonium chloride.

**Methods:**

Fifteen male crossbred dogs were randomly divided into five groups: normal, dry eye self-healing control, cyclosporine-treated, ADMSC-CMV-treated and ADMSC-OESPARC-treated. HCECs were divided into four groups: normal control group, untreated model group, ADMSC-CMV supernatant culture group and ADMSC-OESRARC supernatant culture group.

**Results:**

SPARC-modified ADMSC had the most significant effect on canine ocular surface inflammation, corneal injury, and tear recovery, and the addition of ADMSC-OESPARC cell supernatant also had a salvage effect on HCECs cellular damage, such as cell viability and cell proliferation ability. Moreover, analysis of the co-transcriptome sequencing data showed that SPARC could promote corneal epithelial cell repair by enhancing the in vitro viability, migration and proliferation and immunosuppression of ADMSC.

**Conclusion:**

The in vitro cell test and in vivo model totally suggest that the combination of SPARC and ADMSC has a promising future in novel dry eye therapy.

**Graphical abstract:**

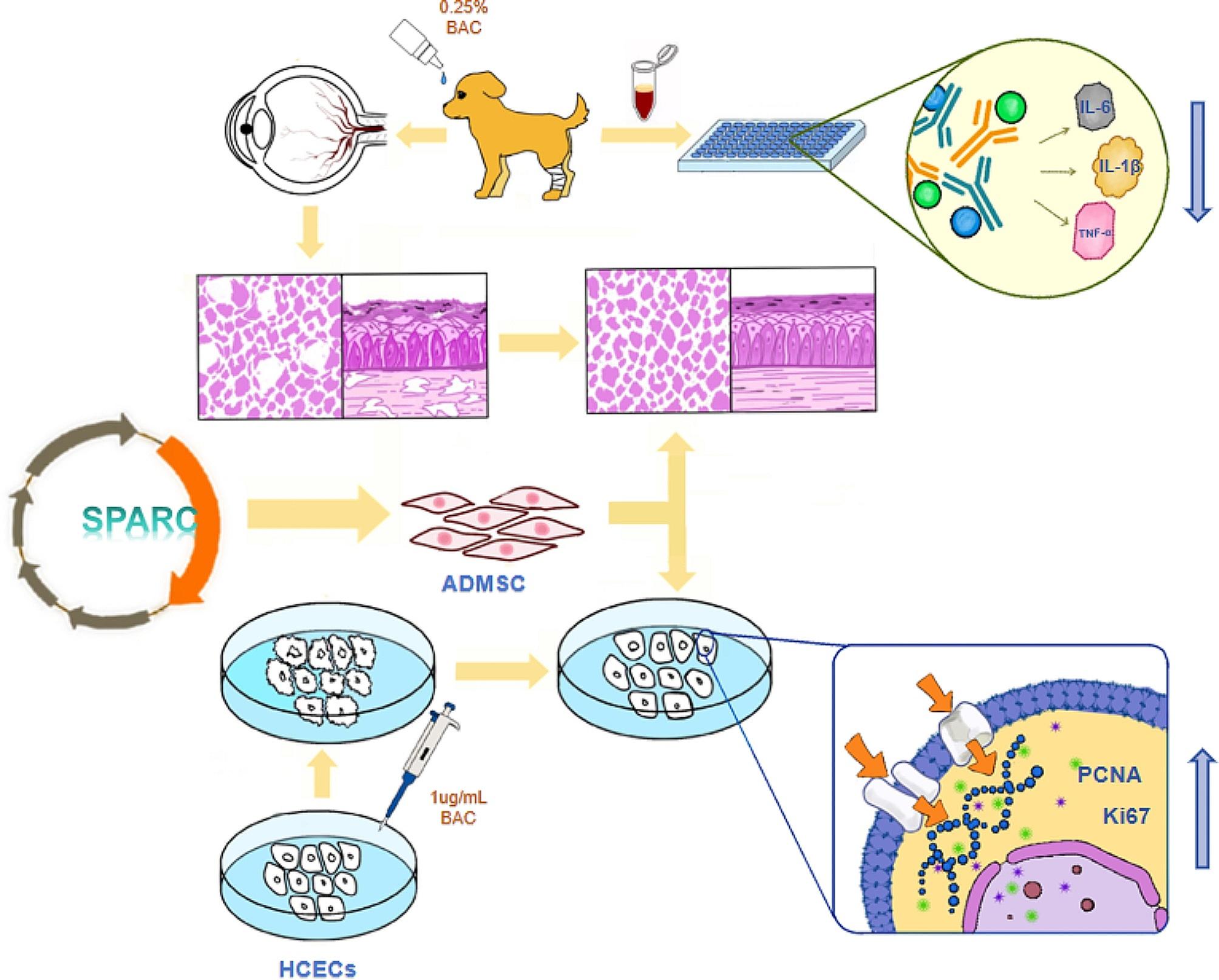

**Supplementary Information:**

The online version contains supplementary material available at 10.1186/s13287-024-03815-z.

## Introduction

In recent years, the demand for eye care has been rising in China due to rapid economic development and significant lifestyle changes. This has resulted in a substantial increase in the incidence of ocular diseases. Concurrently, veterinary clinics have been witnessing a high prevalence of ophthalmic diseases among animals [1]. The social and economic development, coupled with urbanization, has led to an improvement in people’s spiritual, cultural, and material lives. Additionally, the emergence of objective environmental factors, such as aging families, has made pet dogs an essential source of emotional support for individuals [2]. Moreover, dogs serve various functions, including search and rescue, drug detection, guiding, and companionship, making their economic value immeasurable [3]. Consequently, safeguarding the health status of dogs is crucial to ensure their working effectiveness and to realize social and economic benefits. One of the most prevalent canine ophthalmic diseases is dry eye syndrome, with its incidence rate steadily increasing over the years, as revealed by research findings.

Dry eye, also known as Keratoconjunctivitis Sicca (KCS), is a disease characterized by inflammation, pain, redness, and itching in the eye [4]. It is caused by abnormal tear quality or quantity, as well as damage to the eye’s surface resulting from unstable lacrimal secretion. The main symptoms of dry eye include dryness, a foreign body sensation, and a burning sensation in the eye [5]. In the early stages, the eye’s surface becomes cloudy and dry before progressing to fibrosis and even blepharospasm. Another common cause of dry eye is Sjögren syndrome, an unexplained chronic systemic autoimmune disease that affects the exocrine glands, resulting in dryness of the mouth, eyes, and other mucous membranes due to lymphocytic infiltration and glandular dysfunction [6]. Therefore, the pathogenesis of dry eye is complex, with multiple etiologies. Current research suggests that the localized inflammatory immune response of the eye plays a significant role in the pathological damage associated with dry eye. Consequently, anti-inflammatory treatment is a prominent area of investigation [7]. Although various drugs, such as the immunosuppressant cyclosporine, are available for KCS, they are irritating and not suitable for long-term use [8]. Conversely, adipose-derived mesenchymal stem cells (ADMSC) have the advantage of weak immunogenicity and do not actively release their identity information once they enter the host. By participating in the immune process, they can evade the host’s immune response through immune privilege. Moreover, ADMSC can exert regulatory effects on various aspects of the immune response, which gives them an edge in treating dry eye compared to immunosuppressants [9]. However, there are several critical issues associated with ADMSC as a treatment option. The cellular state and activity of ADMSC are influenced by unfavorable factors in the in vivo environment and the outside world, resulting in reduced therapeutic effects. Prolonged in vitro culture leads to decreased proliferative capacity, senescence, and morphological changes of ADMSC. After transplantation, approximately 90% of the ADMSC die within 72 h, and their survival and proliferation rates are not as high as desired. This can be attributed to the lack of required nutrients or growth factors in the body. Although some studies have attempted to enhance the viability of ADMSC in both in vivo and in vitro settings, the results have been unsatisfactory [10].

SPARC (Secreted Protein Acidic and Rich in Cysteine), also known as Secreted Protein Acidic Collagen Osteoproteins, was first identified by Termine et al. in bone tissue in 1981 [11]. This protein, rich in cysteine and containing osteocalcin and BM-40, is a 43 kDa protein secreted into the extracellular matrix by various cell types [12]. It plays a significant role in regulating the extracellular matrix by participating in various processes, including cell adhesion, cell migration, cellular differentiation, and matrix remodeling [13]. Several key findings regarding the functional role of SPARC have been elucidated in the literature. Nie J et al. demonstrated that SPARC modulates WAT body composition by influencing ASC mobilization through the α5β1 integrin complex [14]. Moreover, Bradshaw AD et al. highlighted the impact of SPARC expression on the cell structure of mesangial cells, in addition to its involvement in regulating the cell cycle of fibroblasts and smooth muscle cells [15]. Importantly, SPARC has been shown to be particularly abundant during cell construction of peripheral structures and tissue remodeling, notably during the remodeling and repair of adult tissues such as chondrocytes and osteoblasts [16]. Furthermore, studies indicate that SPARC plays a regulatory role in the activity of key growth factors including platelet-derived growth factor (PDGF), fibroblast growth factor (FGF), and vascular endothelial growth factor (VEGF). These growth factors are intricately involved in diverse biological processes such as embryonic development, angiogenesis, tissue remodeling, and cell renewal [17]. Moreover, it has been observed that the expression of SPARC is regulated during corneal injury and repair. It is involved in the migration and regeneration of corneal epithelial cells, as well as a series of inflammatory responses. Jessica Feldt et al. showed that SPARC, a novel blastocyte glycoprotein, is expressed by lacrimal myoepithelial cells. The lack of SPARC in adulthood may impair myoepithelial cell contraction and tear secretion [18]. Consequently, its involvement is closely associated with the occurrence and development of ocular diseases such as dry eye.

Thus, the potential of using SPARC gene-modified ADMSC to treat dry eye is a significant scientific inquiry that merits investigation. This study examines the impact of ADMSC with SPARC overexpression on the treatment of benzalkonium chloride-induced dry eye in canines [19, 20].

## Materials and methods

### Animals

All animal experiments and conducted procedures were in accordance with the law on animal experimentation and are approved by the regulatory authorities. The work has been reported in line with the ARRIVE guidelines 2.0.

To establish the dry eye-dry keratoconjunctivitis model, twelve 1-year-old healthy male crossbreeds weighing 2.0 ± 0.5 kg were obtained from the Experimental Animal Center of Northwest Agriculture and Forestry University. Additionally, three 1-year-old healthy male crossbreeds weighing 2.0 ± 0.5 kg were utilized as the normal control. For the ADMSC isolation, a 6-month-old female hybrid dog weighing 1.5 kg was obtained from the Laboratory Animal Center of Northwest A&F University. All animal experimental protocols were conducted in strict accordance with the Guide for the Care and Use of Laboratory Animals (Ministry of Science and Technology of the People’s Republic of China, Policy No. GB/T35892-2018). The animals were housed in routine sanitary facilities with the required constant temperature and relative humidity.

### Cell isolation and culture

ADMSC were derived from the abdominal subcutaneous adipose tissue of a 6-month-old female crossbred dog, as female dogs generally have more adipose content. The detailed ADMSC isolation steps and the identification of ADMSC and the establishment of the ADMSC immortalized cell line were described in our previous report [21]. Cells were cultured at 37 °C and 5% CO_2_ incubator in α-MEM (Invitrogen, Carlsbad, CA, USA) complete medium with 10% FBS (Gibco original, origin Australia). When cells were attached to the bottom of the plate at approximately 80% density, a 1:3 passaging was performed. Cells were frozen using DMSO cell freezing solution (Beyotime) [22, 23]. We used cells that were passaged to the third generation after resuscitation in all subsequent experiments.

### Establishment of ADMSC cell lines overexpressing SPARC

The lentiviral expression vector for SPARC was reconstructed by inserting SPARC into the polyclonal site (CMV) of the pCDH-CMV-MSC-EF1 vector. Subsequently, this plasmid was transfected into 293T cells in the presence of two helper plasmids (PAX, VSVG) for lentiviral packaging. After 48 h of viral tapping, the cell supernatant was collected and the virus particles were purified and concentrated. The concentrated virus particles were then tapped into immortalized ADMSC cell lines for further experimentation. Green fluorescence-positive cell clones were screened using 1 µg/mL Puromycin, and single-cell clones displaying green fluorescence were selected through the dilution method. Finally, the selected clones were expanded in culture to measure mRNA levels using real-time quantitative PCR and protein levels using ELISA [24].

### Real-time fluorescence quantitative polymerase chain reaction analysis

Total RNA was extracted from ADMSC-CMV and ADMSC-OESPARC using TRIzol reagent (Takara, Japan) according to the instructions provided by the reagent vendors. The extracted RNA was then reverse transcribed to cDNA using a reverse transcriptase kit (Thermo Fisher Scientific). Subsequently, a quantitative real-time polymerase chain reaction (qRT-PCR) was carried out using a CFX96 real-time PCR system. The qRT-PCR protocol involved pre-denaturation at 94 °C for 5 min, denaturation at 94 °C for 30 s, annealing at 58 °C for 30 s, extension at 70 °C for 30 s, and a total of 39 cycles. As an internal reference, GAPDH was used. The relative expression of genes was evaluated through the utilization of comparative CT values obtained from the qRT-PCR. The following primer sequences were used: GAPDH, F: GCTGCCAAATATGACGACATCA, R:GTAGCCCAGGATGCCTTTGAG; SPARC, F:ATGAGGGCCTGGATCTTCTT, R: TTAGATCACAAGATCCTTGT [25].

### Detection of SPARC protein levels in cells by ELISA

SPARC levels in ADMSC-CMV and ADMSC-OESPARC supernatants were determined by double-antibody sandwich assay using the enzyme-linked immunoassay 96T kit for cysteine-rich acidic protein (SPARC), and five replicate wells were set up for each group. The assay was performed according to the instructions provided by the reagent supplier (FANKEWEI, Shanghai, China). Absorbance (OD) was measured at 450 nm using an enzyme counter and the concentration of SPARC in the samples was calculated from the standard curve [26].

### Cell growth curves

Both ADMSC-CMV and ADMSC-OESPARC groups of cells were inoculated into 24-well plates at 5 × 10^3^ cells per well. The medium in the plates was changed daily using α-MEM (+). Cells were manually counted in 3 wells of each group every 24 h. This process was repeated until day 8. Finally, based on the cell count results, a cell growth curve was plotted, with the horizontal coordinate indicating time and the vertical coordinate indicating the number of cells [27].

### EdU cell proliferation assay

Cells in logarithmic growth phase were used in this study. First, ADMSC-CMV and ADMSC-OESPARC cells were inoculated into 96-well plates with approximately 1 × 10^3^ cells per well. The plates were then cultured until reaching a density of 60–70%. After discarding the culture medium, EdU staining was performed based on the instructions provided by the reagent vendor (Reebok Bio, Guangzhou, China). The staining process was observed and photographed under a fluorescence microscope immediately upon completion. Multiple fields of view were randomly selected for each sample, followed by the counting of EdU fluorescence-positive cells and calculation of the percentage of positive cells using Image J [28].

### In vivo potency studies

#### Models of canine dry eye disease

After a 3-week acclimatization period, fifteen male crossbred dogs were randomly divided into five groups: normal, dry eye self-healing control, cyclosporine-treated, ADMSC-CMV-treated, and ADMSC-OESPARC-treated, three dogs in each group. To model canine dry eye, all dogs, except the normal group, were given 0.25% benzalkonium chloride drops in both eyes twice a day for seven consecutive days [29]. From day 8 onwards, different treatments were administered to each group. The dry eye self-healing control dogs received 0.7% saline drops in both eyes once a day for eight consecutive days. The cyclosporine treatment group received cyclosporine ophthalmic solution in both eyes once a day for eight consecutive days. The ADMSC-CMV treatment group received 200 µL of 0.7% saline suspension containing 1 × 10^5^ ADMSC in both eyes once a day for eight consecutive days. Similarly, the ADMSC-OESPARC treatment group received 200 µL of 0.7% saline suspension containing 1 × 105 ADMSC in both eyes once a day for eight consecutive days [30, 31]. Each time the 0.7% saline cell suspension was freshly prepared before using, with no more than a 15-minute interval between isolation of the cells from the petri dish and their use on the surface of the experimental dog’s eye.

#### Ocular surface inflammation index

Ocular surface inflammation was observed in dogs at eight time points: day 1, day 3, day 5, day 7, day 9, day 11, day 13, and day 15 of the experiment. The dogs were anesthetized with 1% sodium pentobarbital 0.1 mL/ 10 g intraperitoneally and kept sedentary to facilitate the detection of various dry eye indices. Ciliary congestion (absent, 0; present but less than 1 mm, 1; present and greater than 1 mm less than 2 mm, 2; present and greater than 2 mm, 3); central corneal edema, peripheral corneal edema and peripheral corneal edema (absent, 0; present but clear iris texture can be seen, 1; present but clear iris texture cannot be seen, 2; present but pupil cannot be seen, 3). The sum of the three scores is the final Keratoconus Inflammation Index, and it is important that the same person who don’t know the grouping situation performed and scored each operation [32, 33].

#### Corneal sodium fluorescein staining

The corneal fluorescein sodium staining method is commonly used in clinical practice to evaluate the integrity of the corneal epithelium. A positive result indicates a corneal epithelial defect, suggesting discontinuity of the corneal epithelial cell layer. Corneal sodium fluorescein staining experiments were performed at five time points: day 1, day 3, day 7, day 9, and day 15 of the experiment. To perform the staining, 0.5 mL of 2% liquid fluorescein sodium was pipetted onto the surface of the canine eye using a 1 mL spiking gun. After 2 min, corneal epithelial staining was observed under cobalt light using a slit lamp, and photographs were taken to record the staining. The cornea was divided into four regions: supratemporal, infratemporal, supranasal, and infranasal. A scoring system ranging from 0 to 4 was used to evaluate each region’s staining. The scoring criteria were as follows: no staining (0 points), scattered punctate staining (1 point), slight diffuse punctate staining (2 points), severe diffuse staining but not in obvious lamellae (3 points), and obvious lamellar staining (4 points). After scoring was completed for each region, the scores were totaled. It is important that the same person who don’t know the grouping situation performed and scored each operation [34].

#### Tear secretion experiments

Tear volume was measured at four time points: day 1, day 7, day 10, and day 15 of the experiment. In order to ensure accurate measurements, precautions were taken to avoid the use of eye drops and bright light stimulation in the examination room prior to the assessment. The measurement technique involved using a 40 mm×5 mm strip of Whatman 41# filter paper. This strip was carefully folded at a right angle using sterile forceps and then clamped inside the conjunctival sac at the inner 1/3 of the lower eyelid. The other end of the strip was hung on the outside of the lower eyelid and left in place for 2 min. After this time, the wet length of the strip was observed and recorded. According to the established criteria, a wet length of 15 mm to 25 mm was considered normal, while a wet length of 11 mm to 14 mm indicated early dry eye. A wet length of 6 mm to 10 mm was classified as moderate dry eye, and a wet length of ≤ 5 mm was indicative of severe dry eye [35].

#### Collection of canine serum

On the 15th day of experiment, serum was collected from all groups of dogs. To collect the serum, blood was drawn from the cephalic vein of the forearm. Before drawing the blood, the hair in the area was clipped, and the skin was sterilized with iodine and alcohol. The blood collector then tightly held the upper part of the clipped area using the thumb and forefinger of the left hand. This caused the veins of the lower limbs to fill. With the syringe attached to the 6-gauge needle, the right hand quickly punctured into the vein, and blood was withdrawn at an appropriate speed. Once 2 mL of whole blood was drawn, the needle was removed, and the blood was slowly injected into a 1.5 mL centrifuge tube along the wall. The tube, containing the blood, was placed in a 37℃ warm box for 1 h to promote coagulation. Afterward, centrifugation was performed at 2000 rpm for 10 min. This step aided in collection of the supernatant while avoiding aspiration of impurities. During this process, the serum appeared clear and transparent, either colorless or slightly yellow. Finally, the collected canine serum was stored in portions at -20 °C [27].

#### Histological analysis

After 8 days of treatment, dogs in the dry eye model group and the remaining four groups were euthanized by intraperitoneal injection of an overdose of anesthetics (Ketamine and Xylazine). Periocular tissues, such as corneal tissues and transient membrane glands, were gently separated and collected. These tissues were then washed with PBS and fixed with a 4% paraformaldehyde solution fixative at 4 °C for 24 h. Following fixation, the tissues were gradually dehydrated, embedded in paraffin wax, and sliced into 4 µM sections. Hematoxylin-eosin (H&E) staining and peridynamic acid-schiff (PAS) staining were performed according to the instructions of the reagent vendor (Beyotime). Morphological changes of corneal epithelial cells and transient membrane gland tissues were observed under a light microscope, starting from low magnification and progressing to high magnification. Histopathological observation of the ocular surface was conducted, with 3 sections observed, recorded, and photographed in each eye [36].

#### Immunohistochemistry staining

Eye sections were deparaffinized twice with xylene for 10 min each time. Then, they were deparaffinized with a xylene-ethanol mixture for 5 min, followed by sequential washing with anhydrous ethanol for 5 min, 95% alcohol for 5 min, 85% alcohol for 5 min, 75% alcohol for 5 min, 50% alcohol for 5 min, and distilled water twice for 2 min each time. Next, Tris/EDTA pH 9.0 restoration buffer was used for antigen repair by microwaving the sections for 15–20 min and allowing them to cool at room temperature. After three washes with PBS, the sections were treated with 3% H_2_O_2_ for 15 min to eliminate endogenous peroxidase activity, and the tissues were blocked with animal serum. Incubation with PCNA antibody (1:150; immunoway Inc) was carried out overnight at 4 °C. Subsequently, the sections were washed three times with PBS and incubated with horseradish peroxidase-labeled streptavidin working solution for 30 min. After three more washes with PBS, the sections were treated with DAB chromogenic solution for color development and hematoxylin staining solution for nuclear staining. Finally, the samples were dehydrated, sealed with drops of neutral resin, and analyzed under a light microscope [36].

#### ELISA for inflammatory factors

The levels of interleukin-6 (IL-6), interleukin-1β (IL-1β), and tumor necrosis factor-α (TNF-α) in the serum of each group of dogs were determined by double antibody sandwich assay using the 96T ELISA kits for canine IL-6, canine IL-1β, and canine TNF-α, respectively. The assay procedure was performed according to the instructions provided by the reagent supplier (FANKEWEI, Shanghai, China). Five replicate wells were set up in each group of canine sera for the determination of these three inflammatory factors. The absorbance (OD) was measured at 450 nm with an enzyme meter, and the concentrations of IL-6, IL-1β, and TNF-α in the serum of the samples were calculated by a standard curve [26].

### In vitro efficacy studies

#### Human corneal epithelial cell culture

HCECs cells, gifted by Dr. Shaohui Pan from Wenzhou Medical University, were cultured in an incubator at 37 ℃ with 5% CO_2_ using DMEM/F12 medium (Invitrogen, Carlsbad, CA, USA) containing 10% FBS and 1% ITS (insulin-transferrin-selenium-aminoethanol) (both from Gibco original, origin Australia). When the cell density reached approximately 80%, the cells were digested and passaged using 0.25% trypsin. For different experimental requirements, the HCECs were inoculated into 6-well, 48-well, and 96-well plates [37, 38].

#### Cell scratch experiment

Mark a horizontal line on the back of the 6-well plate for observation, inoculate HCECs cells equally into the prepared 6-well plate, waiting for the cells to fully integrate, use a 200 µL tip perpendicular to the marked horizontal line, scrape off the cells with equal widths, and make cell scratches. Excess supernatant should be aspirated, and the cells should be washed twice by adding PBS. The HCECs should be then classified into two groups: the culture group with ADMSC-CMV supernatant and the culture group with ADMSC-OESRARC supernatant. In the first group, 1mL of ADMSC-CMV supernatant and 1mL of DMEM/F12 medium containing 2% serum should be added. In the second group, 1mL of ADMSC-OESRARC supernatant and 1mL of DMEM/F12 medium containing 2% serum were added. Subsequently, the cells were cultured, and the width of the scratch should be photographed and recorded at 12 h and 24 h [27]. The culture solution of ADMSC used was as described in 2.2.

#### Giemsa staining

In 48-well plates, 250 µL/well of HCECs cell suspension was inoculated. The plates were divided into four groups: normal control group, untreated model group, ADMSC-CMV supernatant culture group, and ADMSC-OESRARC supernatant culture group. Each group consisted of three replicate wells. Use culture solution as described in 2.9.1. After the cells were adhered to the wall, except for the normal control group, each group was treated with benzalkonium chloride (1 µg/mL per well) for 24 h [39]. Following this treatment, the culture medium was changed. In the ADMSC-CMV supernatant culture group, 125 µL of ADMSC-CMV supernatant and 125 µL of DMEM/F12 medium containing 10% serum were added. Similarly, in the ADMSC-OESRARC supernatant culture group, 125 µL of ADMSC-OESRARC supernatant and 125 µL of DMEM/F12 medium containing 10% serum were added. The culture solution of ADMSC used was as described in 2.2. In the untreated model group, 250 µL of normal DMEM/F12 medium containing 10% serum was added, and the cells were cultured for an additional 24 h. Subsequently, the cell supernatant of each group was discarded. The cells were then fixed with 4% paraformaldehyde at room temperature for 20 min, washed three times with PBS, and stained with the configured Giemsa Staining Solution working solution dropwise for 15 min. After three additional washes with PBS, the cells were observed and photographed under a light microscope, following the instructions provided by the Giemsa Staining Kit (Beyotime) [25].

#### CCK-8 cell proliferation assay

The 96-well plate was inoculated with HCECs cell suspension (100 µL/well), divided into four groups, namely the normal control group, untreated model group, ADMSC-CMV supernatant culture group, and ADMSC-OESRARC supernatant culture group. Each group consisted of 5 replicate wells. Use culture solution was described as in 2.9.1. After the cells adhered to the wall, benzalkonium chloride was added to each well (except for the normal control group) at 1 µg/mL for 24 h. Then, the culture medium was changed. For the ADMSC-CMV supernatant culture group, 50 µL of ADMSC-CMV supernatant and 50 µL of DMEM/F12 medium containing 10% serum were added. Similarly, for the ADMSC-OESRARC supernatant culture group, 50 µL of ADMSC-OESRARC supernatant and 50 µL of DMEM/F12 medium containing 10% serum were added. The culture solution of ADMSC used was as described in 2.2. The untreated model group received 100 µL of plain DMEM/F12 medium containing 10% serum. After incubating the cells for an additional 24 h, according to the reagent vendor’s instructions (Mishushengwu, Xian, China), 10 µL of CCK-8 solution was added to each well. The cells were incubated for another hour in a cell culture incubator, and the absorbance value at 450 nm was measured using an enzyme marker [40].

#### Real-time fluorescence quantitative polymerase chain reaction analysis

The six-well plates were first inoculated with HCECs cell suspensions (2 mL/well) and then divided into four groups: normal control group, untreated model group, ADMSC-CMV supernatant culture group, and ADMSC-OESRARC supernatant culture group. Use culture solution as described in 2.9.1. The cells were allowed to attach to the wall, and benzalkonium chloride was added to each group (except the normal control group) at a concentration of 1 µg/mL per well for 24 h. After that, the culture medium was changed. In the ADMSC-CMV supernatant culture group, 1 mL of ADMSC-CMV supernatant and 1mL of DMEM/F12 medium containing 10% serum were added. Similarly, in the ADMSC-OESRARC supernatant culture group, 1mL of ADMSC-OESRARC supernatant and 1mL of DMEM/F12 medium containing 10% serum were added. The culture solution of ADMSC used was as described in 2.2. As for the untreated model group, 2mL of plain DMEM/F12 medium containing 10% serum was replaced and the cells were incubated for an additional 24 h, the total RNA was extracted from the HCECs of each group using the TRIzol reagent (Takara, Japan) according to the instructions of reagent vendors. Reverse transcription to cDNA was performed using a reverse transcriptase kit (Thermo Fisher Scientific). quantitative real-time polymerase chain reaction (qRT-PCR) was performed using the CFX96 Real-Time Polymerase Chain Reaction (PCR) System: pre-denaturation at 94 °C for 5 min, denaturation at 94 °C for 30 s, annealing at 58 °C for 30 s, and extension at 70 °C for 30 s. 39 cycles. GAPDH was used as an internal reference. The relative expression of inflammatory factors IL-10, TNF-α, MMP9 and epidermal growth factor EGF was measured using the comparative CT values of qRT-PCR. The primer sequences were as follows: GAPDH, F: GCTGCCAAATATGACGACATCA, R: GTAGCCCAGGATGCCTTTGAG; IL-10, F: TTGCCTGGTCCTCCTGACTG, R: GTCTTCACTCTGCTGAAGGCA; TNF-α, F: CCCGAGTGACAAGCCTGTAG, R: TGAGGTACAGGCCCTCTGAT; MMP, F: GGACAAGCTCTTCGGCTTCT, R: TCGCTGGTACAGGTCGAGTA; EGF, F: GTGAGATGGGTGTGTCCCAGTG, R: GGGGTGGAGTAGAGTCAAGA [4].

#### Flow cytometry

First, HCECs cell suspension was inoculated into 6-well plates, following the grouping and processing methods outlined in Sect. 2.9.5. The cells were then incubated for 24 h. After that, the cell culture solution from each group was aspirated into a suitable centrifuge tube. Next, the adherent cells were washed once with PBS and digested with 0.25% trypsin. The cell culture solution collected earlier was added to the digestion mix, and the cells were gently blown down and transferred to a centrifuge tube. Subsequently, each group of HCECs was stained using the Annexin V-FITC Apoptosis Detection Kit according to the instructions provided by the reagent vendor (Beyotime). After resuspending 100,000 HCECs cells, the suspension was centrifuged, and the supernatant was discarded. Then, 195 µL of Annexin V-FITC conjugate was added to gently resuspend the cells. Next, 5 µL of Annexin V-FITC was added and gently mixed, followed by the addition of 10 µL of propidium iodide (PI) staining solution, also gently mixed. The cells were incubated at room temperature and protected from light for 15 min. Finally, the cells were analyzed using an analytical flow cytometer, and apoptosis was detected in each group. Additionally, In situ FITC/PI double-staining fluorescence assay was also performed on adherent cells to corroborate the results of flow cytometry [42].

#### Cellular immunofluorescence

The cells were inoculated in 96-well plates and grouped according to the treatment described in Sect. 2.9.4. Following a 24 h incubation period, the cells were washed with PBS. Subsequently, the cells in each group were fixed using 4% paraformaldehyde at room temperature for 20 min and then washed three times with PBS. To break the membrane, a solution of 0.2–0.5% Triton X-100 dissolved in citrate buffer was added and incubated for 15 min at room temperature. After washing with PBS three more times, each well was blocked with 70 µL of 10% FBS at room temperature for 1 h. Once the FBS was aspirated, 40 µL of primary antibody (PCNA at 1:100 dilution and Ki67 at 1:100 dilution, both from immunoway) was added to each well and incubated overnight at 4 °C. The cells were then washed three times with PBS, and 30 µL of hochest33342 was added to each well. The reaction was carried out at room temperature for 5 min, after which the cells were observed and photographed under a fluorescence microscope [43].

### RNA sequencing

The cells were divided into ADMSC-CMV group and ADMSC-OESRARC group, and the cell samples were collected and sent to Shanghai Jiayin Biotechnology Ltd. for transcriptome sequencing comparison according to the company’s delivery requirements. Gene Ontology (GO) enrichment of potential target gene information and Kyoto Encyclopedia of Genes and Genomes (KEGG) pathway annotation analysis were performed using annotation databases, and pathways with a *P* value < 0.05 were considered reliable.

### Statistical analysis

Statistical analysis was performed using IBM Corporation’s SPSS version 19.0 software (Chicago, IL, USA). All experimental data were examined using one-way analysis of variance (ANOVA) and are presented as mean SD±. Statistical significance was observed in the comparisons if the *p*-value was less than 0.05. GraphPad Prism software was used to analyze all the data.

## Results

### The viability of ADMSC is enhanced by the overexpression of SPARC

**Fig. 1:**
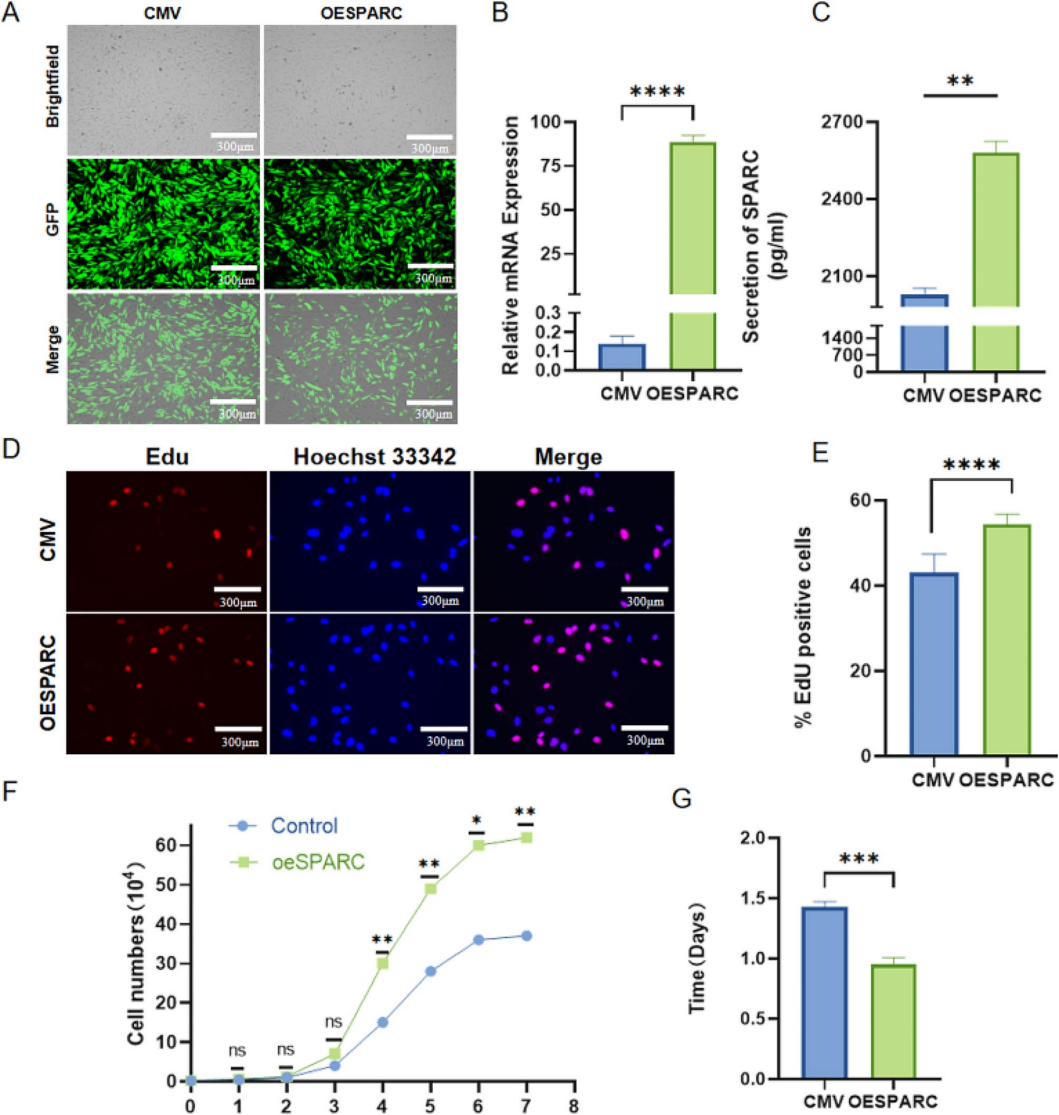
The viability of ADMSC is enhanced by the overexpression of SPARC. (**A**) Steady transfected SPARC cell lines expressing green fluorescence. (**B**) mRNA expression of SPARC in ADMSC cells with SPARC overexpression. (**C**) SPARC content of supernatants from ADMSC cells with SPARC overexpression. (**D**) Edu staining. (**E**) Quantitative analysis of Edu staining. (**F**) Cell growth curves. (**G**) Group doubling time. ns: Not significant; * : *P* < 0.05 ; ** : *P* < 0.01 ; *** : *P* < 0.001

To examine the effects of SPARC overexpression, a lentiviral expression vector of pCDH-CMV-SPARC-EF1-copGFP-T2A-Puro was constructed and transfected into 293T cells. In the control group, pCDH-CMV-EF1-copGFP-T2A-Puro was used. Subsequently, viral particles were collected and attacked the ADMSC cell line, resulting in the establishment of steady-transfected green fluorescence-positive cell lines (Fig. 1A). The mRNA levels were evaluated using real-time quantitative PCR and shown more 700-fold up-regulation of SPARC compared to the control group (Fig. 1B). In addition, protein levels were measured using ELISA (Fig. 1C). Furthermore, the overexpression of SPARC exhibited a better promotion effect on ADMSC, as evidenced by the results of Edu staining, cell growth curve, and population doubling. Notably, the cell proliferation rate of ADMSC-OESPARC was significantly faster than that of the ADMSC-CMV group (Fig. 1D, E, F, G). These findings strongly suggest that overexpression of SPARC enhances the activity and proliferation of ADMSC.

### The efficacy of mesenchymal stem cells in canine dry eye syndrome is enhanced by overexpression of SPARC

**Fig. 2:**
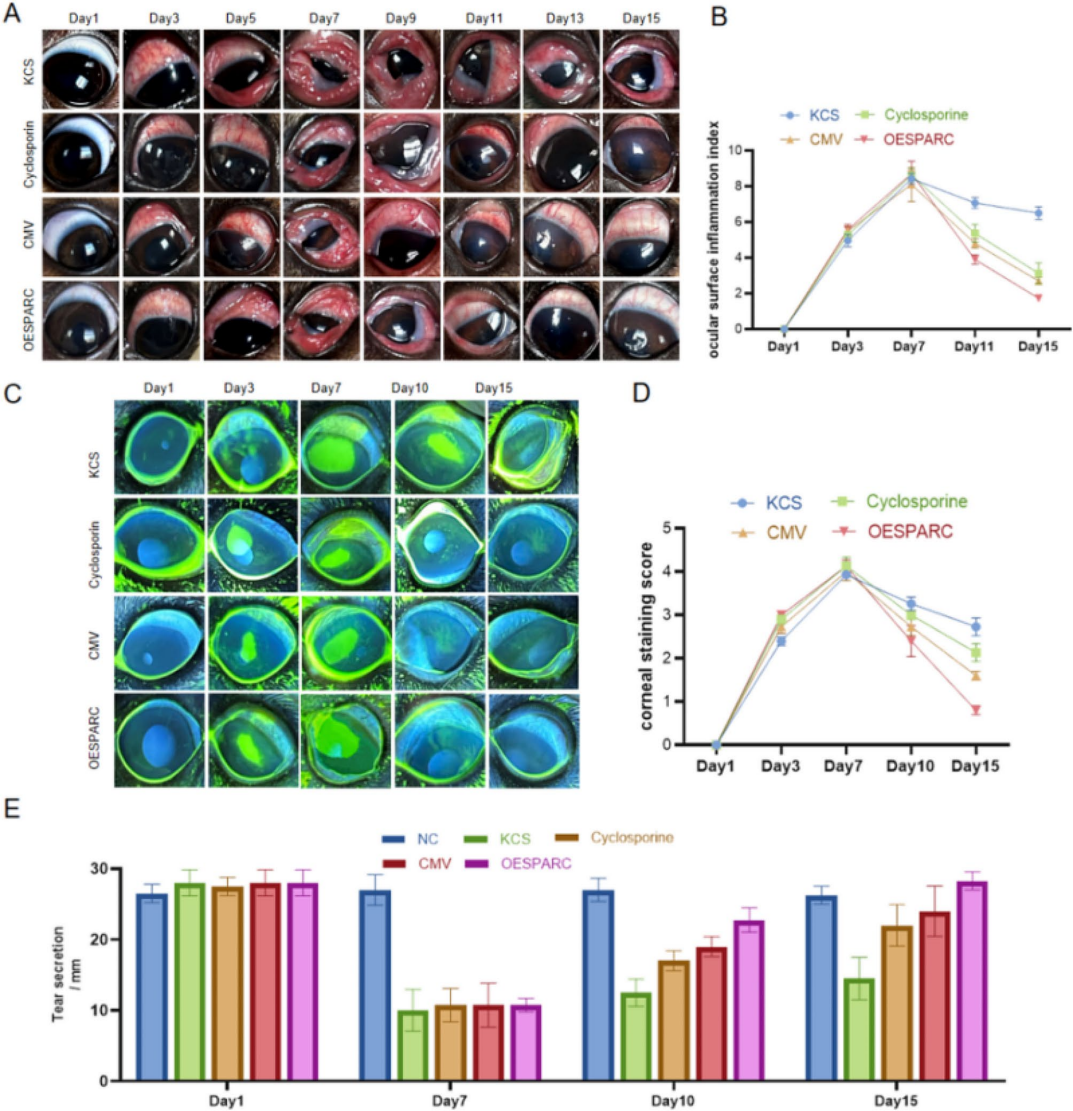
The efficacy of ADMSC in canine dry eye disease is enhanced by overexpression of SPARC. (**A**) Observed variations in ocular surface inflammation in canines. (**B**) Ocular surface inflammation index score. (**C**) Corneal sodium fluorescein staining changes recorded. (**D**) Corneal sodium fluorescein staining score. (**E**) Record of basal tear secretion

The canine dry eye model was created using 0.25% benzalkonium chloride ophthalmic spotting. Autologous and allograft recovery around the canine eye was recorded. Ciliary congestion, corneal redness (Fig. 2A, B), corneal damage (Fig. 2C, D), and tear secretion (Fig. 2E) of canine eyes recovered with time in all groups. The slowest recovery was observed in the group of the untreated KCS model, while the fastest recovery was observed in the group of ADMSC-OESPARC. The group of ADMSC-OESPARC showed a more significant effect than that of cyclosporine drops. Repair was basically completed and reached the normal level on the 15th day. These results demonstrate that ADMSC overexpressing SPARC can shorten the repair time of canine corneal injury and alleviate dry eye symptoms.

### Overexpression of SPARC enhances the beneficial effects of ADMSC in alleviating dry eye through multiple mechanisms

**Fig. 3:**
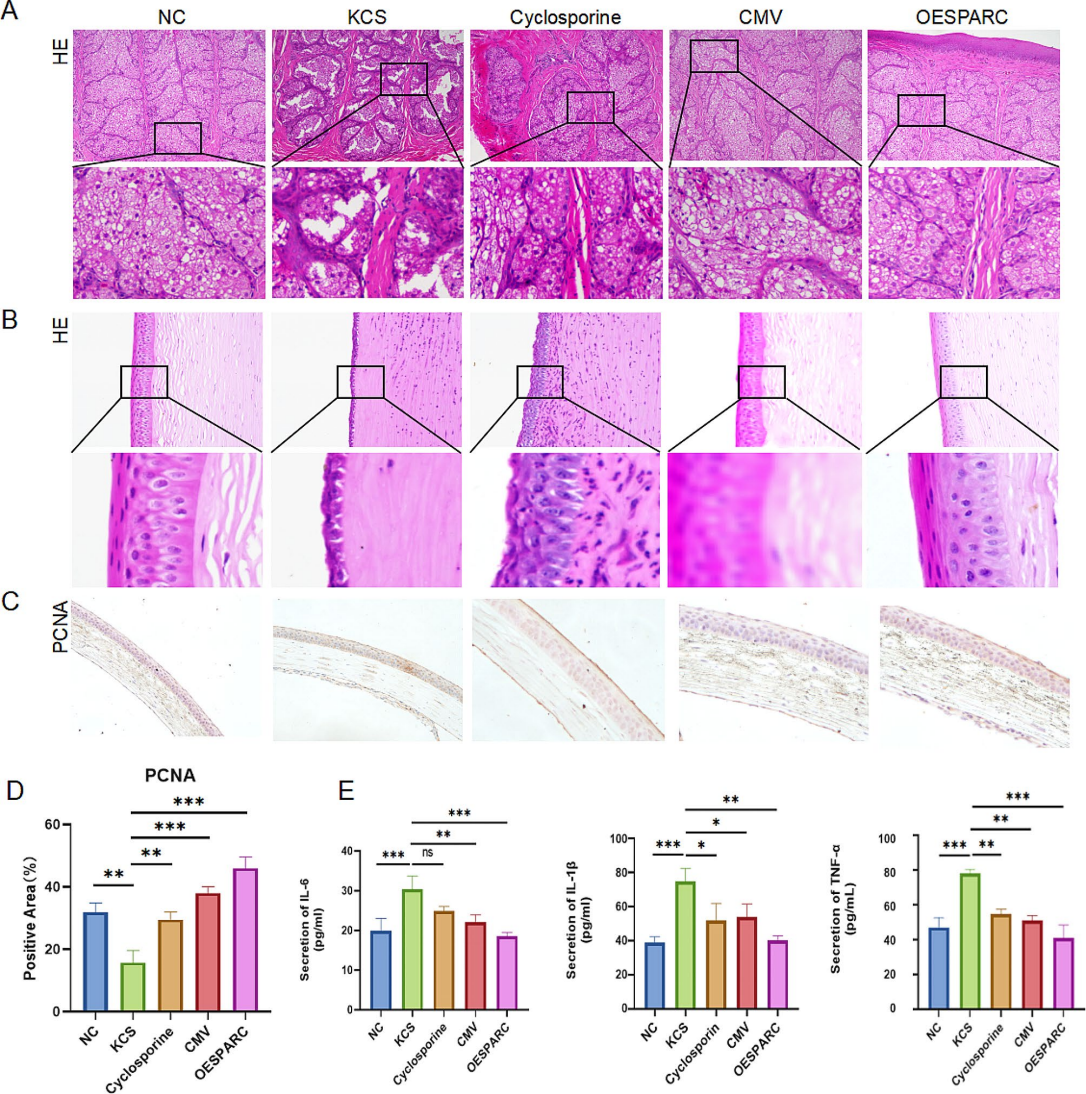
Overexpression of SPARC enhances the beneficial effects of ADMSC in alleviating dry eye through multiple mechanisms. (**A**) HE staining is used to observe the structure of transient membrane glands. (**B**) HE staining is used to examine the structure of corneal epithelial cells. (**C**) Immunohistochemistry for PCNA expression observation. (**D**) Quantitative analysis of immunohistochemical results. (**E**) ELISA is used to detect inflammatory factors. ns: Not significant; * : *P* < 0.05 ; ** : *P* < 0.01 ; *** : *P* < 0.001

The untreated KCS model group showed damaged, disorganized, rough, and even ruptured follicular structure of the transient membrane glands as observed in HE staining. Corneal hyperplasia was also evident, along with disorganized epithelial cell layer, loose and ruptured structure of the cupular cell bundles. In contrast, the ADMSC-OESPARC-treated group exhibited more noticeable improvement compared to the cyclosporine and ADMSC-CMV-treated groups. The treated group showed clear vesicular structures with smooth edges, attenuated corneal hyperplasia, aligned epithelial cell layer with a smoother surface, and a denser stromal layer of the cupular cell bundles(Figs. 3A, B). In immunohistochemistry, by PCNA staining, there were fewer brown cells in the untreated KCS model group and an increased brown cells in the ADMSC-OESPARC-treated group, which indicated the best proliferation of cells in this group, and the results of immunohistochemistry were quantified (Fig. 3C, D). Furthermore, the ADMSC-OESPARC treatment group exhibited the most significant decrease in serum levels of IL-6, IL-1β, and TNF-α, indicating a notable suppression of inflammation(Fig. 3E). Overall, these results suggest that overexpression of SPARC contributes to the repair of transient membrane glands and cuprocytes, proliferation of corneal epithelial cells, and inhibition of inflammatory response, thereby promoting the efficacy of ADMSC in treating dry eye syndrome.

### SPARC improves cell viability and anti-inflammatory capacity of HCECs

**Fig. 4:**
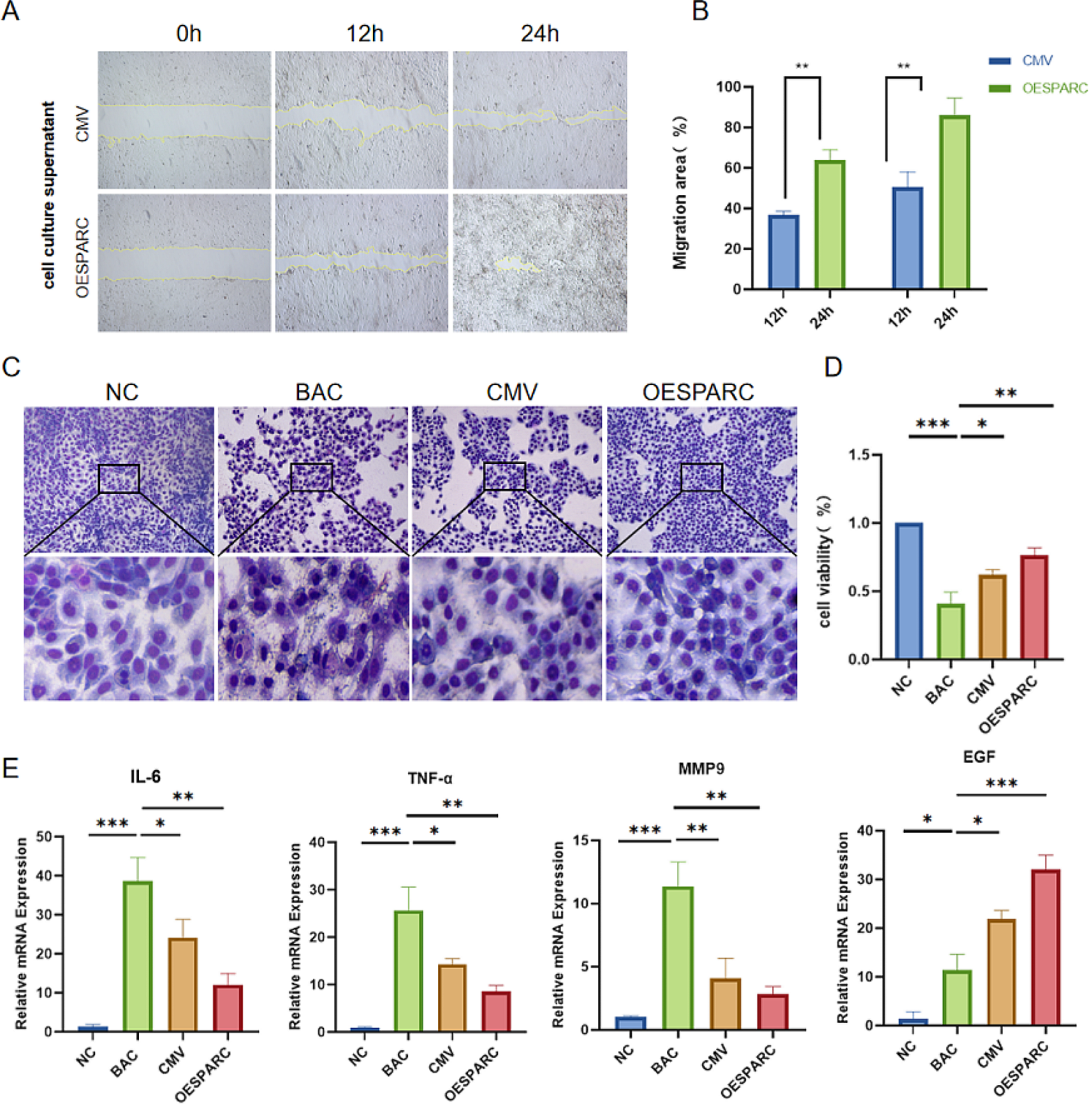
SPARC improves cell viability and anti-inflammatory capacity of HCECs. (**A**) The HCECs cell scratch assay detects the cell migration ability of each group. (**B**) Quantitative analysis of cell migration assay. (**C**) Giemsa staining was used to observe the morphology of HCECs in all groups. (**D**) CCK8 detection of cell viability in each group. (**E**) The mRNA expression of IL-6, TNF-α, MMP9 and EGF in each group. ns: Not significant; * : *P* < 0.05 ; ** : *P* < 0.01 ; *** : *P* < 0.001

To investigate the relationship between ADMSC-OESPARC on HCECs viability and anti-inflammation, we established an in vitro model of corneal injury by inducing HCECs using 1 µg/mL BAC. We then evaluated the effect of the ADMSC-OESPARC supernatant on cell migration of HCECs. The results showed that the ADMSC-OESPARC supernatant exhibited a higher capability in promoting cell migration of HCECs (Fig. 4A, B). Moreover, Giemsa staining demonstrated that HCEC cell morphology returned to normal after adding the ADMSC-OESPARC supernatant, appearing as smooth and densely packed transverse ellipsoids (Fig. 4C). Additionally, the viability of HCEC cells was markedly improved as detection by CCK8 (Fig. 4D). Furthermore, the expression levels of inflammation-related factors IL-6, TNF-α and MMP9 in HCECs cells significantly decreased after 24 h, whereas the expression of epidermal growth factor (EGF) was up-regulated (Fig. 4E).

### Effect of SPARC on proliferation and apoptosis of HCECs

**Fig. 5:**
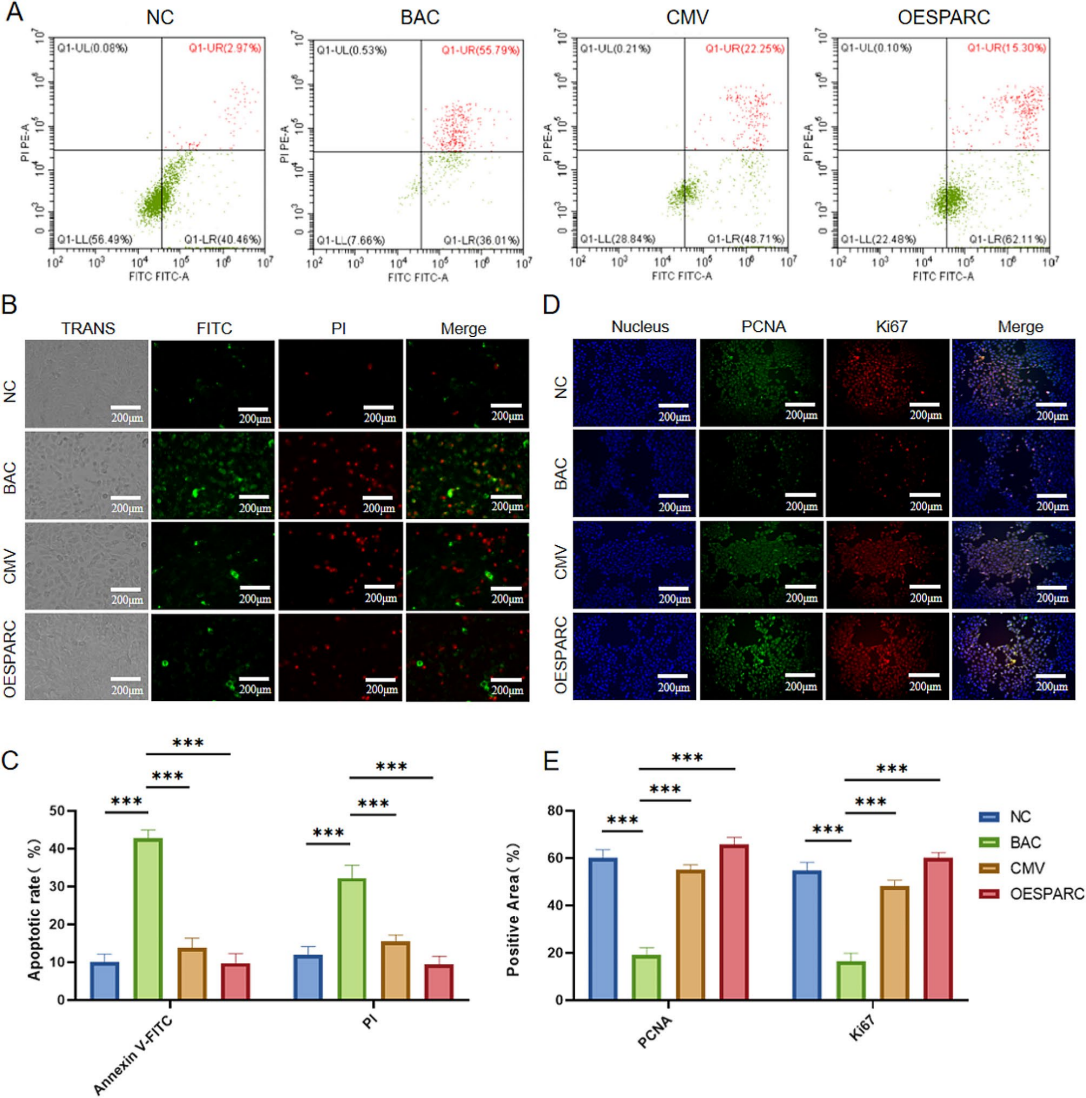
Effect of SPARC on proliferation and apoptosis of HCECs. (**A**) Flow cytometry to detect apoptosis. (**B**) The adherent cells were detected for FITC/PI double staining using in situ fluorescence. (**C**) Quantification of FITC/PI fluorescence assay results. (**D**) PCNA and Ki67 expression were detected using cellular immunofluorescence. (**E**) Quantification of cellular immunofluorescence results. ns: Not significant; * : *P* < 0.05 ; ** : *P* < 0.01 ; *** : *P* < 0.001

After BAC induction, flow cytometry detected an increase in late apoptotic and necrotic cells in HCECs. The addition of ADMSC-OESPARC supernatant to culture had the most significant effect on apoptosis(Fig. 5A). Furthermore, in situ FITC/PI double-staining fluorescence assay on adherent cells corroborated the results of the flow cytometry, and the fluorescence results were quantified(Fig. 5B, C). Moreover, A notable rise in the intracellular PCNA and Ki67 expression in HCECs was observed upon the addition of ADMSC-OESPARC supernatant culture and the cellular immunofluorescence results were quantified (Fig. 5D, E). The results above indicate that ADMSC overexpressing SPARC influence the migration and differentiation of HCECs by enhancing the proliferation and anti-apoptotic capacity of HCECs, thereby affecting their migration and differentiation.

### Transcriptome analysis of ADMSC reveals the differentially expressed genes and pathways influenced by SPARC

**Fig. 6:**
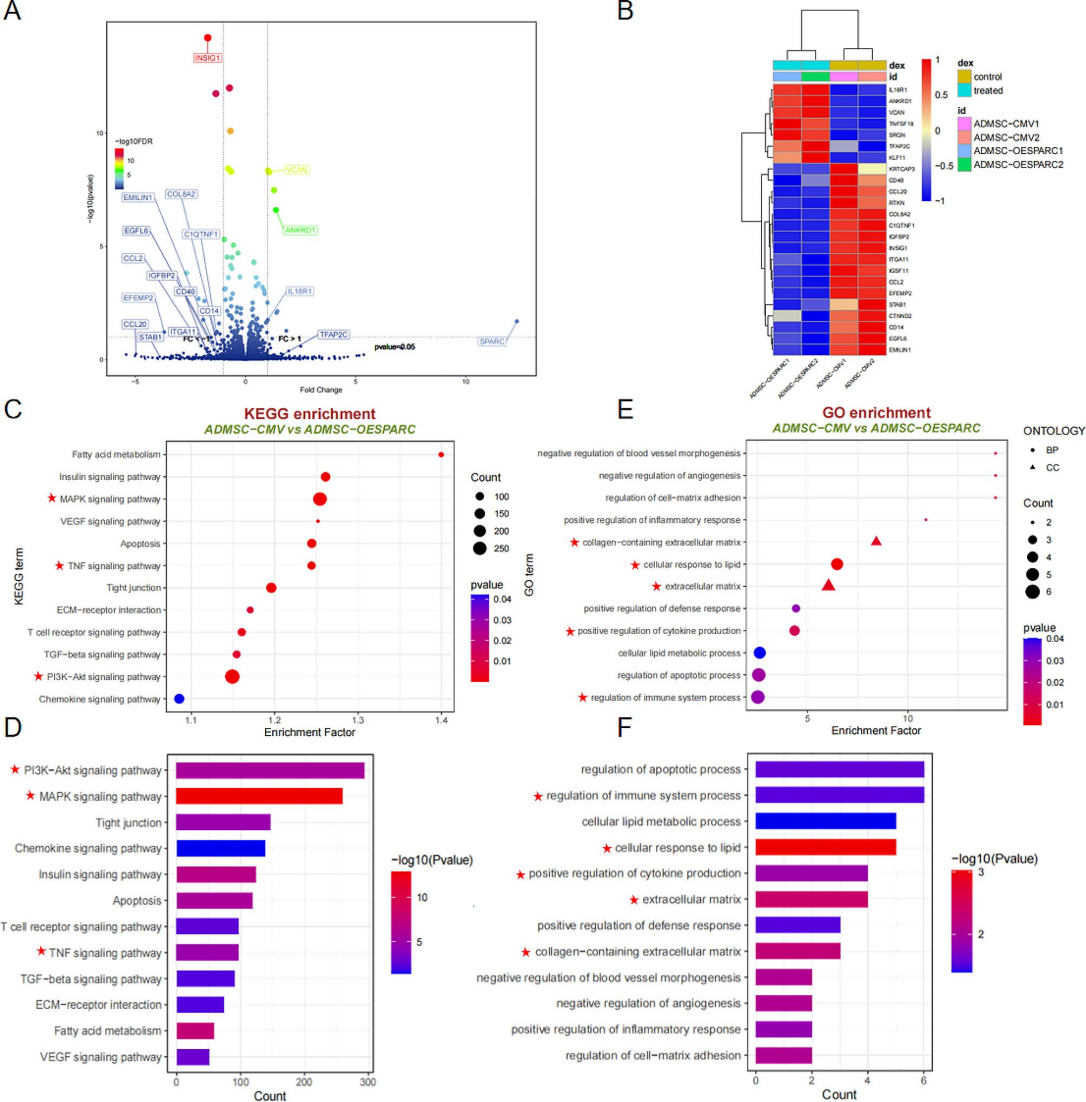
Transcriptome analysis of ADMSC reveals the differentially expressed genes and pathways influenced by SPARC. (**A**) Volcano map of differentially expressed genes identified by RNA-seq of ADMSC stably expressing SPARC. (**B**) RNA-seq data heatmap showed representative differential genes regulated by SPARC. (**C**) KEGG enrichment analysis bubble diagram of ADMSC-OESPARC & ADMSC-CMV differentially expressed genes. (**D**) KEGG enrichment analysis histogram of ADMSC-OESPARC & ADMSC-CMV differentially expressed genes. (**E**) GO enrichment analysis bubble diagram of ADMSC-OESPARC & ADMSC-CMV differentially expressed genes. (**F**) GO enrichment analysis histogram of ADMSC-OESPARC & ADMSC-CMV differentially expressed genes

In order to further explore the target genes and downstream signaling pathways regulated by SPARC, transcriptome sequencing was performed on ADMSC-OESPARC and ADMSC-CMV. The sequencing results showed that 60 genes were up-regulated and 66 genes were down-regulated in the ADMSC-OESPARC group (Fig. 6A). The heat map showed the representative genes positively and negatively regulated by SPARC. The negatively regulated genes included CD40, CCL20, COL8A2 and IGFBP2, and the positively regulated genes included IL18R1, VCAN and SRGN (Fig. 6B). KEGG analysis of these up-regulated and down-regulated differentially expressed genes revealed that they were enriched in KEGG pathways, including MAPK, VEGF, TNF and PI3K-Akt signaling pathways (Fig. 6C, D). The enriched GO pathways mainly included cell adhesion, cell response to lipid, positive regulation of glial cell proliferation, and regulation of angiogenesis, as revealed by the GO analysis performed on the up-regulated and down-regulated differentially expressed genes (Fig. 6E, F).

## Discussion

The key to the pathogenesis of dry eye lies in the change in the stability of the tear film on the corneal surface. This change can lead to corneal epithelial defects, which not only damage the normal structure and function of the corneal epithelium but also potentially result in the partial or complete loss of the corneal epithelial cell layer. Consequently, this can impede or delay the repair process, creating a detrimental cycle that complicates the treatment of dry eye [44]. Impaired stability of corneal cells increases the osmotic pressure of tears, thereby activating the mitogen-activated protein kinase (MAPK) and nuclear factor-κB (NF-κB) signaling pathways in the corneal epithelium [44]. This activation triggers the release of various inflammatory mediators, including interleukin-6 (IL-6), tumor necrosis factor-α (TNF-α), and matrix metalloproteinases (MMPs), especially MMP-9. MMP-9 can disrupt the tight junction protein complex, leading to corneal epithelial cell shedding, filamentous keratitis, and an increased likelihood of developing moderate to severe dry eye [45]. ADMSC have gained attention for their therapeutic potential in treating chronic ophthalmic conditions due to their regenerative, repair, and immunomodulatory capabilities. When ADMSC are used in dry eye dogs, they can migrate to the injured corneal site and enhance the proliferation and differentiation of corneal cells [46]. However, despite their promising attributes, single mesenchymal stem cell therapy has limitations, such as poor cell activity and low cell survival rates.

SPARC plays a crucial role in the regulation of the extracellular matrix. It can affect the stability of cell-matrix adhesion of the cytoskeleton and regulate cell adhesion through interaction with cell membrane receptors of mesenchymal stem cells, thereby enhancing the stability of mesenchymal stem cells in a damaged microenvironment [47]. Research indicates that SPARC can modulate the activity of cell proliferation-related signaling pathways, such as the PI3K/Akt and ERK/MAPK pathways. Activating these signaling pathways aids in boosting the proliferation capacity of mesenchymal stem cells, ultimately enhancing their ability to repair damaged corneal epithelial cells [48]. Furthermore, the expression of SPARC undergoes regulation during corneal repair, which is closely intertwined with the pathogenesis and progression of eye disorders like dry eye [49]. Following a corneal injury, corneal cells and adjacent stromal fibroblasts become activated and migrate to the affected area to engage in the reparative process. These new stromal cells, rich in endoplasmic reticulum, have the capacity to synthesize cytoskeleton components, adhesion molecules, and various collagen types [50]. By interacting with corneal stromal cells, SPARC significantly influences various processes, including collagen fiber contraction, regulation of cell-matrix forces, modulation of cell morphology, and ultimately facilitating the repair of corneal injuries [51]. To validate this concept, an ADMSC cell line that overexpresses SPARC was engineered in this study. Through evaluating the cell line’s growth and proliferation abilities, it was discerned that SPARC can enhance the activity, proliferation rate, and growth potential of ADMSC in vitro. Analysis of transcriptome sequencing data further corroborated that SPARC contributes to promoting lipid synthesis, vascular development, cell adhesion, collagen fiber regulation, anti-inflammatory responses, and other essential biological processes within ADMSC. This substantiates the notion that SPARC augments the healing and growth capabilities of ADMSC in the context of corneal epithelium restoration.

To investigate the therapeutic potential of ADMSC in treating dry eye disease, this study utilized Benzalkonium Chloride (BAC) to induce a dry eye disease model in dogs and conducted live experiments. Benzalkonium chloride, a common preservative found in eye drops, can lead to dry eye symptoms due to its detrimental effects on the connexin of the corneal epithelium and the lipid layer of the tear film upon prolonged excessive usage [52]. Recent research has increasingly used BAC to establish animal models of dry eye disease. Notably, studies have demonstrated that applying a 0.25% BAC solution to the eyes of rabbits for 14 days resulted in reduced tear secretion and corneal sodium fluorescein staining by the third day, and the appearance of cup cells by the seventh day [29]. Building upon these findings, a dry eye model was induced in mice by utilizing a 0.1% BAC solution. By the 7th day, mice exhibited corneal inflammation and heightened apoptosis of corneal epithelial cells [53]. In this investigation, a 0.25% BAC solution was employed to create a moderate to severe dry eye syndrome model in dogs. As the blood of dogs from each group needed to be collected for serological analysis post-treatment, each dog in the study was considered an independent experimental sample, and no internal control model was established. Using established criteria for grading dry eye severity, model dogs displaying moderate to severe clinical symptoms were selected to evaluate the therapeutic potential of ADMSC overexpressing SPARC in treating dry eye. Given the potential complications associated with ADMSC therapy, the dosage of cells administered is crucial to ensure treatment safety and efficacy. Typically, the recommended dosage range for administering ADMSC in ophthalmic diseases in animal models is between 0.5 × 10^5^ to 1 × 10^6^ cells per kilogram of body weight, for either systemic or local delivery [30, 31]. Despite the absence of consensus on optimal dosages, empirical evidence suggests a dose-dependent immunosuppressive effect of ADMSC within a defined range, beyond which higher doses do not confer additional therapeutic benefits. This study determined that administering 1 × 10^5^ ADMSC (equivalent to 0.5 × 10^5^ cells/kg) was both safe and efficacious in treating dry eye. Furthermore, the delivery route of therapeutic agents significantly impacts drug absorption and utilization efficiency. While intravenous injection is commonly used in mesenchymal stem cell studies, its efficacy may be limited when treating eye diseases, potentially leading to pulmonary embolism risk [54]. Alternative methods for ocular administration in experimental animals include eye drops, subconjunctival and intravitreal injections [55]. Of these, eye-drop administration is considered safer and more convenient, albeit with reduced ocular surface residence time and efficiency of drug absorption, so we adopt the means of repeated administration to make the treatment effect more significant. In this research, a cell suspension overexpressing SPARC was applied to the affected dog’s ocular surface. Treatment efficacy was assessed based on the clinical rating standard for dry eye disease, pathological biopsy examinations, and serum inflammation indicators. Results indicated that SPARC modification enhanced the therapeutic effects of ADMSC by expediting corneal swelling recovery, promoting nictitating gland, corneal, and conjunctival tissue repair, enhancing tear secretion restoration, reducing inflammation in dry eye dogs, and accelerating epithelial cell proliferation in corneal tissue. This suggests that SPARC-modified ADMSC may enhance local administration efficacy and exhibit improved therapeutic benefits for dry eye.

We aimed to further ascertain the enhancing impact of SPARC on the efficacy of ADMSC at the cellular level, given that the destabilization of corneal epithelial cells stands as the focal point of dry eye pathogenesis. To induce injury in human corneal epithelium cells (HCECs) in vitro, we utilized a 1 µg/mL BAC solution, following the investigation conducted by Jillian F. et al. into the effects of benzalkonium chloride concentrations ranging from 0.2 to 200 µg/mL on the lipid set of HCECs [39]. Subsequently, a non-contact two-dimensional co-culture model was established by adding ADMSC-OESPARC supernatant to the injured corneal epithelial cell lines for analysis of the therapeutic potential of ADMSC-OESPARC [56]. The outcomes revealed a restoration of HCECs morphology towards a normal transverse oval shape and a remarkable enhancement in cell viability. Furthermore, following a 24-hour co-culture period, there was a notable decrease in the expression levels of inflammatory mediators IL-6, TNF-α, and MMP-9 released by HCECs, coupled with an up-regulation in the expression level of epidermal growth factor EGF. It was observed that SPARC played a crucial role in boosting the reparative effects of ADMSC on injured corneal epithelial cells by fostering cell proliferation and impeding cell apoptosis. This investigation contributes vital insights and robust evidence for the management of dry eye disease, underscoring its significance in clinical interventions for dry eye disease in veterinary practice. Moreover, drawing on the groundwork of animal studies, this research is anticipated to offer valuable insights for the exploration of related human conditions. Accordingly, our data encourage the need for a prospectively randomized clinical trial to investigate the efficacy and safety of such a combined regimen for patients with KCS.

In our study, we have addressed certain questions regarding the effects of SPARC-modified ADMSC. However, long-term effects of this treatment remain unclear. Furthermore, the optimal dose and frequency of ADMSC administration require further investigation, especially considering the large size of dogs and the limited sample size in our study. The exact mechanism underlying the potential immunomodulatory effects of SPARC-modified ADMSC also warrants further exploration. Additionally, the clinical application of this therapy presents challenges that need to be considered in future research endeavors. These challenges include determining how to manage the cost associated with mass expansion of mesenchymal stem cells, preparing cellular agents, and developing more convenient drug delivery technology.

## Conclusions

This study demonstrated that SPARC-modified ADMSC showed superior efficacy compared to either ADMSC or cyclosporine eye drops alone in promoting corneal healing, up-regulating tear secretion, and suppressing periocular inflammation in a canine model of dry eye induced with 0.25% BAC. The cellular supernatant of SPARC-modified ADMSC not only strengthened the cellular viability and anti-inflammatory capacity of HCECs but also proved to be the most effective in rescuing 1 µg/mL BAC-induced damage to HCECs. Furthermore, the co-sequencing results revealed that SPARC played a crucial role in promoting the repair of corneal epithelial cells and regulated the production of inflammatory mediators by increasing the in vitro viability, migration and proliferation, immunosuppression, regulation of neovascularization, and anti-scarring of ADMSC. This evidence suggests the potential future clinical application of SPARC in combination with ADMSC as cell therapy. This study offers important insights for the development of new approaches in treating dry eye syndrome.

### Electronic supplementary material

Below is the link to the electronic supplementary material.

Supplementary Material 1

## Data Availability

All data generated and analyzed during this study are included in this published article.

## References

[1] Leonard BC, Sebbag L (2023). Veterinary Ophthalmology-Ocular Surface diseases. Vet Ophthalmol.

[2] Cherniack EP, Cherniack AR (2014). The benefit of pets and animal-assisted therapy to the health of older individuals. Curr Gerontol Geriatr Res.

[3] Fine AH (2018). The role of therapy and service animals in the lives of persons with disabilities. Rev Sci Tech.

[4] Pflugfelder SC, Stern ME (2020). The cornea in keratoconjunctivitis sicca. Exp Eye Res.

[5] Caban M, Omulecki W, Latecka-Krajewska B (2022). Dry eye in Sjogren’s syndrome - characteristics and therapy. Eur J Ophthalmol.

[6] Bjordal O, Norheim KB, Rødahl E, Jonsson R, Omdal R (2020). Primary Sjögren’s syndrome and the eye. Surv Ophthalmol.

[7] Williams DL, Am-Small N (2008). Anim Pract.

[8] de Paiva CS, Pflugfelder SC, Ng SM, Akpek EK (2019). Topical cyclosporine A therapy for dry eye syndrome. Cochrane Database Syst Rev.

[9] Maria ATJ, Maumus M, Le Quellec A, Jorgensen C, Noel D, Guilpain P (2017). Adipose-derived mesenchymal stem cells in Autoimmune disorders: state of the art and perspectives for systemic sclerosis. Clin Rev Allergy Immunol.

[10] Xia Y, Yang R, Hou Y (2022). Application of mesenchymal stem cell-derived exosomes from different sources in intervertebral disc degeneration. Front Bioeng Biotechnol.

[11] Termine JD, Kleinman HK, Whitson SW, Conn KM, McGarvey ML, Martin GR (1981). Osteonectin, a bone-specific protein linking mineral to collagen. Cell.

[12] Mendis DB, Malaval L, Brown IR (1995). SPARC, an extracellular matrix glycoprotein containing the follistatin module, is expressed by astrocytes in synaptic enriched regions of the adult brain. Brain Res.

[13] Ham SM, Song MJ, Yoon HS, Lee DH, Chung JH, Lee ST (2023). SPARC is highly expressed in Young skin and promotes Extracellular Matrix Integrity in fibroblasts via the TGF- beta signaling pathway. Int J Mol Sci.

[14] Nie J, Chang B, Traktuev DO, Sun J, March K, Chan L, Sage EH, Pasqualini R, Arap W, Kolonin MG (2008). IFATS collection: combinatorial peptides identify alpha5beta1 integrin as a receptor for the matricellular protein SPARC on adipose stromal cells. Stem Cells.

[15] Bradshaw AD, Francki A, Motamed K, Howe C, Sage EH (1999). Primary mesenchymal cells isolated from SPARC-null mice exhibit altered morphology and rates of proliferation. Mol Biol Cell.

[16] Lafuse WP, Wozniak DJ, Rajaram MVS (2020). Role of Cardiac macrophages on Cardiac inflammation, fibrosis and tissue repair. Cells.

[17] Luo Z, Zhou Y, Luo P, Zhao Q, Xiao N, Yu Y, Yan Q, Lu G, Cheng L (2014). SPARC deficiency affects bone marrow stromal function, resulting in impaired B lymphopoiesis. J Leukoc Biol.

[18] Feldt J, Garriz A, Rodriguez Benavente MC, Woodward AM, Zoukhri D, Argüeso P (2022). The Matricellular protein SPARC decreases in the Lacrimal Gland at Adulthood and during inflammation. Invest Ophthalmol Vis Sci.

[19] Walter MNM, Wright KT, Fuller HR, MacNeil S, Johnson WEB (2010). Mesenchymal stem cell-conditioned medium accelerates skin wound healing: an in vitro study of fibroblast and keratinocyte scratch assays. Exp Cell Res.

[20] Kozdon K, Fitchett C, Rose GE, Ezra DG, Bailly M (2015). Mesenchymal Stem Cell-Like properties of Orbital fibroblasts in Graves’Orbitopathy. Invest Ophthalmol Vis Sci.

[21] Wei Y, Fang J, Cai S, Lv C, Zhang S, Hua J (2016). Primordial germ cell-like cells derived from canine adipose mesenchymal stem cells. Cell Prolif.

[22] Peng F, Wu H, Zheng Y, Xu X, Yu J (2012). The effect of noncoherent red light irradiation on proliferation and osteogenic differentiation of bone marrow mesenchymal stem cells. Lasers Med Sci.

[23] Fang J, Yan Y, Teng X (2018). Melatonin prevents senescence of canine adipose-derived mesenchymal stem cells through activating NRF2 and inhibiting ER stress. Aging-US.

[24] Zhang M, Li N, Liu W (2021). Eif2s3y promotes the proliferation of Spermatogonial Stem cells by activating ERK Signaling. Stem Cells Int.

[25] Li B, Cheng X, Aierken A (2021). Melatonin promotes the therapeutic effect of mesenchymal stem cells on type 2 diabetes Mellitus by regulating TGF-β pathway. Front Cell Dev Biol.

[26] Zhang MF, Wan SC, Chen WB (2023). Transcription factor Dmrt1 triggers the SPRY1-NF-κB pathway to maintain testicular immune homeostasis and male fertility. Zool Res.

[27] Kou Z, Li B, Aierken A (2023). Mesenchymal stem cells pretreated with collagen promote skin Wound-Healing. Int J Mol Sci.

[28] Aierken A, Li B, Liu P (2022). Melatonin treatment improves human umbilical cord mesenchymal stem cell therapy in a mouse model of type II diabetes mellitus via the PI3K/AKT signaling pathway. Stem Cell Res Ther.

[29] Thacker M, Sahoo A, Reddy AA, Bokara KK, Singh S, Basu S, Singh V (2023). Benzalkonium chloride-induced dry eye disease animal models: current understanding and potential for translational research. Indian J Ophthalmol.

[30] Fuentes-Julián S, Arnalich-Montiel F, Jaumandreu L, Leal M, Casado A, García-Tuñon I (2015). Adipose-derived mesenchymal stem cell administration does not improve corneal graft survival outcome. PLoS ONE.

[31] Mittal SK, Foulsham W, Shukla S, Elbasiony E, Omoto M, Chauhan SK (2019). Mesenchymal stromal cells modulate corneal alloimmunity via secretion of hepatocyte growth factor. Stem Cells Transl Med.

[32] Feng J, Liu Y, Ren Y, et al. Evaluation of Dry Eye Severity and Ocular Surface inflammation in patients with Pemphigus and Pemphigoid. Ocul Immunol Inflamm Published Online January. 2023;15. 10.1080/09273948.2022.2154680. 10.1080/09273948.2022.2154680 36637982

[33] Mullick R, Annavajjhala S, Thakur P, Mohapatra A, Shetty R, D’Souza S (2021). Efficacy of topical cyclosporine 0.05% and osmoprotective lubricating eye drops in treating dry eye disease and inflammation. Indian J Ophthalmol.

[34] Dogaroiu C, Zarojanu A, Scurtu R, Morosanu G, Tataru CP, Puscasu AC (2014). Postmortem corneal changes evaluated by fluorescent staining. Rom J Leg Med.

[35] Xu K, Liu X, ning, Zhang H bing, Zhu X ping, Zhang X. jiao. Tear film instability is associated with weakened colocalization between occludin and MUC5AC in scopolamine-induced dry eye disease (DED) rats. Int Ophthalmol. 2023;43(2):463–473. 10.1007/s10792-022-02443-x. 10.1007/s10792-022-02443-x 35908134

[36] He W, Qin D, Li B (2021). Immortalized canine adipose-derived mesenchymal stem cells alleviate gentamicin-induced acute kidney injury by inhibiting endoplasmic reticulum stress in mice and dogs. Res Vet Sci.

[37] Baba K, Sasaki K, Morita M (2020). Cell jamming, stratification and p63 expression in cultivated human corneal epithelial cell sheets. Sci Rep.

[38] Bourcier T, Forgez P, Borderie V, Scheer S, Rostene W, Laroche L (2000). Regulation of human corneal epithelial cell proliferation and apoptosis by dexamethasone. Invest Ophthalmol Vis Sci.

[39] Ziemanski JF, Wilson L, Barnes S, Nichols KK (2023). Evaluation of the effects of latanoprost and benzalkonium chloride on the cell viability and nonpolar lipid profile produced by human meibomian gland epithelial cells in culture. Mol Vis.

[40] Yang D, Wei Y, Lu Q (2021). Melatonin alleviates LPS-induced endoplasmic reticulum stress and inflammation in spermatogonial stem cells. J Cell Physiol.

[41] Yang XC, Wu XL, Li WH (2022). OCT6 inhibits differentiation of porcine-induced pluripotent stem cells through MAPK and PI3K signaling regulation. Zool Res.

[42] Wu XL, Zhu ZS, Xiao X (2021). LIN28A inhibits DUSP family phosphatases and activates MAPK signaling pathway to maintain pluripotency in porcine induced pluripotent stem cells. Zool Res.

[43] Daguia Zambe JC, Zhai Y, Zhou Z (2018). miR-19b-3p induces cell proliferation and reduces heterochromatin-mediated senescence through PLZF in goat male germline stem cells. J Cell Physiol.

[44] Wang L, Wang X, Chen Q, Wei Z, Xu X, Han D, Zhang Y, Chen Z, Liang Q (2023). MicroRNAs of extracellular vesicles derived from mesenchymal stromal cells alleviate inflammation in dry eye disease by targeting the IRAK1/Table 2/NF-κB pathway. Ocul Surf.

[45] Baba K, Sasaki K, Morita M, Tanaka T, Teranishi Y, Ogasawara T, Oie Y, Kusumi I, Inoie M, Hata KI, Quantock AJ, Kino-Oka M, Nishida K (2020). Cell jamming, stratification and p63 expression in cultivated human corneal epithelial cell sheets. Sci Rep.

[46] Gugjoo MB, Amarpal A, Sharma GT (2019). Mesenchymal stem cell basic research and applications in dog medicine. J Cell Physiol.

[47] Bradshaw AD (2012). Diverse biological functions of the SPARC family of proteins. Int J Biochem Cell Biol.

[48] Zhu J, Wang LY, Li CY, Wu JY, Zhang YT, Pang KP, Wei Y, Du LQ, Liu M, Wu XY (2020). SPARC promotes self-renewal of limbal epithelial stem cells and ocular surface restoration through JNK and p38-MAPK signaling pathways. Stem Cells.

[49] Salonen J, Domenicucci C, Goldberg HA, Sodek J (1990). Immunohistochemical localization of SPARC (osteonectin) and denatured collagen and their relationship to remodelling in rat dental tissues. Arch Oral Biol.

[50] Lin JB, Shen X, Pfeifer CW, Shiau F, Santeford A, Ruzycki PA, Clark BS, Liu Q, Huang AJW, Apte RS (2023). Dry eye disease in mice activates adaptive corneal epithelial regeneration distinct from constitutive renewal in homeostasis. Proc Natl Acad Sci USA.

[51] Sangaletti S, Tripodo C, Cappetti B, Casalini P, Chiodoni C, Piconese S, Santangelo A, Parenza M, Arioli I, Miotti S, Colombo MP (2011). SPARC oppositely regulates inflammation and fibrosis in bleomycin-induced lung damage. Am J Pathol.

[52] Rath A, Eichhorn M, Träger K, Paulsen F, Hampel U (2019). In vitro effects of benzalkonium chloride and prostaglandins on human meibomian gland epithelial cells. Ann Anat.

[53] Datta S, Baudouin C, Brignole-Baudouin F, Denoyer A, Cortopassi GA (2017). The Eye Drop Preservative Benzalkonium Chloride Potently induces mitochondrial dysfunction and preferentially affects LHON Mutant cells. Invest Ophthalmol Vis Sci.

[54] Furlani D, Ugurlucan M, Ong L, Bieback K, Pittermann E, Westien I (2009). Is the intravascular administration of mesenchymal stem cells safe? Mesenchymal stem cells and intravital microscopy. Microvasc Res.

[55] Gough G, Szapacs M, Shah T, Clements P, Struble C, Wilson R (2018). Ocular tissue distribution and pharmacokinetic study of a small 13 kDa domain antibody after intravitreal, subconjuctival and eye drop administration in rabbits. Exp Eye Res.

[56] Miklíková M, Jarkovská D, Čedíková M, Švíglerová J, Kuncová J, Nalos L, Kubíková T, Liška V, Holubová M, Lysák D, Králíčková M, Vištejnová L, Štengl M (2018). Beneficial effects of mesenchymal stem cells on adult porcine cardiomyocytes in non-contact co-culture. Physiol Res.

