# Supplementary material for: SPARC overexpression in allogeneic adipose-derived mesenchymal stem cells in dog dry eye model induced by benzalkonium chloride
Source: Stem Cell Res Ther. 2024 Jul 2;15:195. doi: 10.1186/s13287-024-03815-z (PMC11218109; doi:10.1186/s13287-024-03815-z)
Supplement: Supplementary file 1 — Supplementary Material 1 [file 13287_2024_3815_MOESM1_ESM.pdf]

AccID	cmv-1	cmv-2	oeSPARC-1	oeSPARC-2
ENSCAFG00845017357	0	0	0	0
ENSCAFG00845017356	0	0	0	0
ENSCAFG00845017355	2	0	1	3
ENSCAFG00845017354	0	0	0	0
ENSCAFG00845017353	0	0	0	0
ENSCAFG00845017352	0	0	0	0
ENSCAFG00845017351	3	2	5	5
ENSCAFG00845005370	950	952	961	912
ENSCAFG00845017350	0	0	0	0
ENSCAFG00845005371	1	1	0	0
ENSCAFG00845005372	0	0	0	0
ENSCAFG00845005373	0	0	0	0
ENSCAFG00845005374	13	20	12	4
ENSCAFG00845005375	1	3	0	0
ENSCAFG00845029339	140	130	122	134
ENSCAFG00845005376	3208	3061	3340	3113
ENSCAFG00845005377	0	0	0	0
ENSCAFG00845029337	0	0	0	0
ENSCAFG00845005378	2	10	6	7
ENSCAFG00845029338	0	1	0	0
ENSCAFG00845005379	514	477	446	472
ENSCAFG00845029335	793	767	778	707
ENSCAFG00845030335	1941	1809	1675	1700
ENSCAFG00845029336	2	1	0	1
ENSCAFG00845030336	0	0	0	0
ENSCAFG00845029333	2	0	6	0
ENSCAFG00845030337	0	0	0	0
ENSCAFG00845029334	36	34	38	46
ENSCAFG00845030338	22	27	22	31
ENSCAFG00845029331	179	198	135	156
ENSCAFG00845030339	0	0	4	4
ENSCAFG00845029332	1178	1053	1186	1115
ENSCAFG00845029330	1072	1027	1154	1274
ENSCAFG00845030330	0	0	1	0
ENSCAFG00845030331	0	0	0	0
ENSCAFG00845030332	0	0	0	0
ENSCAFG00845017359	94	86	107	135
ENSCAFG00845030333	4	2	1	6
ENSCAFG00845017358	0	0	0	0
ENSCAFG00845030334	261	308	288	327

ENSCAFG00845017346	476	440	442	406
ENSCAFG00845020998	0	0	0	0
ENSCAFG00845017345	306	305	298	338
ENSCAFG00845020999	0	0	0	0
ENSCAFG00845017344	2328	2273	1987	2038
ENSCAFG00845020996	0	0	0	0
ENSCAFG00845017343	320	267	315	279
ENSCAFG00845020997	0	0	0	0
ENSCAFG00845017342	0	0	0	0
ENSCAFG00845017341	0	0	0	0
ENSCAFG00845017340	0	0	0	0
ENSCAFG00845005360	0	0	0	0
ENSCAFG00845020990	1	0	0	0
ENSCAFG00845005361	1	3	0	0
ENSCAFG00845020991	0	0	0	0
ENSCAFG00845005362	32	28	26	19
ENSCAFG00845005363	662	623	714	711
ENSCAFG00845005364	2334	2140	2261	2299
ENSCAFG00845020994	1508	1557	1584	1649
ENSCAFG00845029328	10	15	10	8
ENSCAFG00845005365	0	0	2	0
ENSCAFG00845020995	9	4	2	5
ENSCAFG00845029329	242	265	287	245
ENSCAFG00845005366	3	8	8	8
ENSCAFG00845020992	1	0	0	0
ENSCAFG00845029326	27	22	35	13
ENSCAFG00845005367	9	9	4	13
ENSCAFG00845020993	0	0	0	0
ENSCAFG00845029327	43	55	81	73
ENSCAFG00845005368	1	1	0	1
ENSCAFG00845029324	0	0	0	0
ENSCAFG00845030346	0	0	0	0
ENSCAFG00845005369	1433	1467	1347	1398
ENSCAFG00845029325	30	30	44	45
ENSCAFG00845030347	888	898	912	910
ENSCAFG00845029322	0	1	0	0
ENSCAFG00845030348	306	289	348	352
ENSCAFG00845029323	3	1	6	2
ENSCAFG00845030349	0	0	0	0
ENSCAFG00845029320	1	2	0	0
ENSCAFG00845029321	1552	1578	1520	1628

ENSCAFG00845030340	1	1	3	3
ENSCAFG00845030341	0	0	0	0
ENSCAFG00845030342	0	0	0	0
ENSCAFG00845017349	0	0	0	0
ENSCAFG00845030343	0	0	0	0
ENSCAFG00845017348	0	0	0	0
ENSCAFG00845030344	0	0	0	0
ENSCAFG00845017347	0	0	0	0
ENSCAFG00845030345	0	0	0	0
ENSCAFG00845017335	0	0	0	0
ENSCAFG00845019998	114	113	124	145
ENSCAFG00845020987	144	120	115	131
ENSCAFG00845017334	0	0	0	1
ENSCAFG00845019999	0	0	0	0
ENSCAFG00845020988	302	290	297	297
ENSCAFG00845017333	0	0	0	0
ENSCAFG00845020985	6	9	9	7
ENSCAFG00845017332	0	0	0	0
ENSCAFG00845020986	64	92	76	60
ENSCAFG00845017331	10	10	10	13
ENSCAFG00845017330	1031	1005	986	980
ENSCAFG00845020989	0	0	0	0
ENSCAFG00845005350	213	187	141	161
ENSCAFG00845020980	2424	2236	2393	2404
ENSCAFG00845005351	0	0	0	0
ENSCAFG00845029319	13	12	11	15
ENSCAFG00845005352	0	0	0	0
ENSCAFG00845005353	603	554	582	632
ENSCAFG00845020983	1	2	0	3
ENSCAFG00845029317	4723	4532	4021	4097
ENSCAFG00845005354	0	0	0	0
ENSCAFG00845020984	4	0	3	1
ENSCAFG00845029318	50	36	47	47
ENSCAFG00845005355	0	0	0	0
ENSCAFG00845020981	693	570	740	737
ENSCAFG00845029315	5	0	2	1
ENSCAFG00845005356	4494	3973	4610	4697
ENSCAFG00845020982	0	0	0	0
ENSCAFG00845029316	958	922	872	943
ENSCAFG00845005357	435	393	406	385
ENSCAFG00845029313	0	0	0	0

ENSCAFG00845030357	202	194	209	197
ENSCAFG00845005358	0	0	0	0
ENSCAFG00845029314	95	97	71	61
ENSCAFG00845030358	0	0	0	0
ENSCAFG00845005359	0	0	0	0
ENSCAFG00845029311	468	469	380	390
ENSCAFG00845030359	142	133	154	140
ENSCAFG00845029312	0	7	2	2
ENSCAFG00845029310	0	0	0	0
ENSCAFG00845019990	0	0	0	0
ENSCAFG00845019991	594	574	619	594
ENSCAFG00845030350	15	15	18	4
ENSCAFG00845019992	640	677	620	652
ENSCAFG00845030351	239	227	257	238
ENSCAFG00845019993	1145	1097	1095	956
ENSCAFG00845030352	1515	1477	1579	1624
ENSCAFG00845017339	22	9	11	13
ENSCAFG00845019994	2	0	0	0
ENSCAFG00845030353	0	0	0	0
ENSCAFG00845017338	993	828	1021	1003
ENSCAFG00845019995	0	0	0	0
ENSCAFG00845030354	340	308	293	335
ENSCAFG00845017337	289	286	355	286
ENSCAFG00845019996	0	0	0	0
ENSCAFG00845030355	0	0	0	0
ENSCAFG00845017336	0	0	0	0
ENSCAFG00845019997	2	4	2	5
ENSCAFG00845030356	0	0	0	0
ENSCAFG00845017324	0	0	0	0
ENSCAFG00845019987	98	87	94	85
ENSCAFG00845020976	483	509	425	445
ENSCAFG00845017323	0	0	0	0
ENSCAFG00845019988	1214	1209	1172	1258
ENSCAFG00845020977	0	0	0	0
ENSCAFG00845017322	0	1	3	0
ENSCAFG00845019989	0	0	0	0
ENSCAFG00845020974	70	49	53	68
ENSCAFG00845017321	0	0	0	0
ENSCAFG00845020975	1102	1160	1143	1133
ENSCAFG00845017320	227	220	194	190
ENSCAFG00845020978	0	2	1	0

ENSCAFG00845020979	0	0	0	0
ENSCAFG00845005340	13	8	11	5
ENSCAFG00845029308	0	0	0	0
ENSCAFG00845005341	23	16	14	12
ENSCAFG00845029309	47	40	42	42
ENSCAFG00845005342	0	0	0	0
ENSCAFG00845020972	1304	1335	1200	1206
ENSCAFG00845029306	0	0	0	0
ENSCAFG00845005343	0	0	0	0
ENSCAFG00845020973	0	0	0	0
ENSCAFG00845029307	0	0	0	0
ENSCAFG00845005344	0	0	0	0
ENSCAFG00845020970	200	209	215	230
ENSCAFG00845029304	1	4	0	0
ENSCAFG00845005345	121	87	106	82
ENSCAFG00845020971	4	1	1	2
ENSCAFG00845029305	0	0	0	0
ENSCAFG00845005346	611	616	589	519
ENSCAFG00845029302	253	231	276	229
ENSCAFG00845030368	375	281	340	388
ENSCAFG00845005347	0	0	0	0
ENSCAFG00845029303	396	378	282	269
ENSCAFG00845030369	0	0	0	0
ENSCAFG00845005348	0	0	0	0
ENSCAFG00845029300	1151	1146	963	1015
ENSCAFG00845005349	431	387	454	442
ENSCAFG00845029301	0	0	0	0
ENSCAFG00845030360	0	0	0	0
ENSCAFG00845019980	143	163	160	156
ENSCAFG00845030361	3510	3242	3343	3383
ENSCAFG00845019981	8	11	19	11
ENSCAFG00845030362	0	0	0	0
ENSCAFG00845017329	0	1	1	3
ENSCAFG00845019982	0	0	0	0
ENSCAFG00845030363	0	0	0	0
ENSCAFG00845017328	0	1	0	0
ENSCAFG00845019983	0	0	0	0
ENSCAFG00845030364	0	0	0	0
ENSCAFG00845017327	0	0	0	0
ENSCAFG00845019984	0	0	0	0
ENSCAFG00845030365	5	6	6	7

ENSCAFG00845017326	2376	2317	2152	2186
ENSCAFG00845019985	0	0	0	0
ENSCAFG00845030366	602	566	585	607
ENSCAFG00845017325	22	30	16	10
ENSCAFG00845019986	4741	4588	4577	4880
ENSCAFG00845030367	0	0	0	0
ENSCAFG00845017313	998	938	920	1042
ENSCAFG00845019976	594	503	520	568
ENSCAFG00845020965	0	0	0	0
ENSCAFG00845017312	1	0	0	2
ENSCAFG00845019977	33	31	36	54
ENSCAFG00845020966	0	0	0	0
ENSCAFG00845017311	0	0	0	0
ENSCAFG00845019978	1352	1210	1195	1184
ENSCAFG00845020963	0	0	0	0
ENSCAFG00845017310	7	3	2	2
ENSCAFG00845019979	0	0	0	0
ENSCAFG00845020964	0	0	0	0
ENSCAFG00845020969	40	50	50	47
ENSCAFG00845020967	0	0	0	0
ENSCAFG00845020968	0	0	0	0
ENSCAFG00845030370	1	1	2	0
ENSCAFG00845007990	1	3	5	0
ENSCAFG00845007992	0	0	1	2
ENSCAFG00845005330	124	109	141	146
ENSCAFG00845007991	1	2	0	0
ENSCAFG00845005331	2366	2333	2272	2418
ENSCAFG00845007994	0	0	0	0
ENSCAFG00845020961	0	0	0	0
ENSCAFG00845005332	0	0	0	0
ENSCAFG00845007993	0	0	0	0
ENSCAFG00845020962	71	90	48	32
ENSCAFG00845005333	1207	1263	1301	1330
ENSCAFG00845007996	4	1	2	0
ENSCAFG00845005334	0	0	0	0
ENSCAFG00845007995	0	0	0	0
ENSCAFG00845020960	1142	1060	974	1086
ENSCAFG00845005335	0	0	0	0
ENSCAFG00845007998	1	0	0	0
ENSCAFG00845030379	338	368	395	380
ENSCAFG00845005336	211	184	122	138

ENSCAFG00845007997	455	423	501	436
ENSCAFG00845005337	0	0	0	0
ENSCAFG00845005338	0	0	0	0
ENSCAFG00845007999	0	0	0	0
ENSCAFG00845005339	0	0	0	0
ENSCAFG00845030371	2	5	3	1
ENSCAFG00845030372	0	0	0	0
ENSCAFG00845017319	3	2	0	0
ENSCAFG00845019970	0	0	0	0
ENSCAFG00845030373	299	277	201	193
ENSCAFG00845017318	1	0	0	1
ENSCAFG00845019971	0	0	0	0
ENSCAFG00845030374	0	0	0	0
ENSCAFG00845017317	15	3	12	7
ENSCAFG00845019972	1	0	1	2
ENSCAFG00845030375	386	328	348	316
ENSCAFG00845017316	585	546	521	543
ENSCAFG00845019973	5	4	4	6
ENSCAFG00845030376	3	0	0	1
ENSCAFG00845017315	0	0	0	0
ENSCAFG00845019974	0	0	0	0
ENSCAFG00845030377	7	2	1	0
ENSCAFG00845017314	0	0	0	0
ENSCAFG00845019975	0	0	0	0
ENSCAFG00845030378	0	0	0	0
ENSCAFG00845017302	0	0	0	0
ENSCAFG00845019965	0	0	0	0
ENSCAFG00845020954	0	0	0	0
ENSCAFG00845017301	0	0	0	0
ENSCAFG00845019966	0	0	0	0
ENSCAFG00845020955	805	716	870	815
ENSCAFG00845017300	2	1	0	0
ENSCAFG00845019967	0	0	0	0
ENSCAFG00845020952	17	13	4	16
ENSCAFG00845019968	3739	3588	3891	3940
ENSCAFG00845020953	7788	7292	7079	7136
ENSCAFG00845019969	0	0	0	0
ENSCAFG00845020958	1643	1500	1372	1475
ENSCAFG00845020959	678	738	651	709
ENSCAFG00845020956	43	42	54	57
ENSCAFG00845030380	0	0	0	0

ENSCAFG00845020957	520	564	636	672
ENSCAFG00845030381	108	108	83	116
ENSCAFG00845007981	472	408	458	491
ENSCAFG00845007980	3	0	0	0
ENSCAFG00845005320	0	0	0	0
ENSCAFG00845007983	0	0	0	0
ENSCAFG00845020950	656	661	632	693
ENSCAFG00845005321	63	50	57	54
ENSCAFG00845007982	0	0	0	0
ENSCAFG00845020951	0	0	0	0
ENSCAFG00845005322	1	3	0	3
ENSCAFG00845007985	108	114	118	115
ENSCAFG00845005323	0	0	0	0
ENSCAFG00845007984	0	0	0	0
ENSCAFG00845005324	0	0	0	0
ENSCAFG00845007987	268	329	277	249
ENSCAFG00845005325	0	0	2	0
ENSCAFG00845007986	79	97	73	84
ENSCAFG00845005326	1064	904	928	920
ENSCAFG00845007989	44	24	14	14
ENSCAFG00845005327	0	0	0	0
ENSCAFG00845007988	0	0	0	0
ENSCAFG00845005328	4100	3824	3917	3748
ENSCAFG00845005329	0	0	0	0
ENSCAFG00845030382	2243	2307	2379	2566
ENSCAFG00845017309	0	0	0	0
ENSCAFG00845030383	0	0	0	0
ENSCAFG00845017308	0	0	0	0
ENSCAFG00845020949	5	9	10	7
ENSCAFG00845030384	0	0	0	0
ENSCAFG00845017307	55	42	53	67
ENSCAFG00845019960	107	79	106	93
ENSCAFG00845030385	0	0	0	0
ENSCAFG00845017306	1265	1248	929	999
ENSCAFG00845019961	0	0	0	0
ENSCAFG00845030386	48	60	47	48
ENSCAFG00845017305	1	0	0	0
ENSCAFG00845019962	4	4	4	5
ENSCAFG00845030387	0	0	0	0
ENSCAFG00845017304	350	332	351	345
ENSCAFG00845019963	79	85	62	51

ENSCAFG00845030388	0	0	0	0
ENSCAFG00845017303	0	0	0	0
ENSCAFG00845019964	5	3	1	2
ENSCAFG00845030389	0	0	2	0
ENSCAFG00845019954	1	4	4	0
ENSCAFG00845020943	1	1	1	0
ENSCAFG00845019955	1615	1547	1538	1345
ENSCAFG00845020944	970	971	962	910
ENSCAFG00845019956	2731	2598	2474	2663
ENSCAFG00845020941	374	331	342	355
ENSCAFG00845019957	0	0	0	0
ENSCAFG00845020942	1481	1506	1361	1427
ENSCAFG00845019958	0	0	0	0
ENSCAFG00845020947	2162	2114	2148	2076
ENSCAFG00845019959	1	1	4	2
ENSCAFG00845020948	20	31	13	27
ENSCAFG00845030390	0	0	0	0
ENSCAFG00845020945	0	0	0	0
ENSCAFG00845030391	0	2	2	0
ENSCAFG00845020946	0	0	0	0
ENSCAFG00845030392	0	0	0	0
ENSCAFG00845007970	0	0	0	0
ENSCAFG00845007972	120	124	161	176
ENSCAFG00845005310	542	449	509	552
ENSCAFG00845007971	1	0	1	0
ENSCAFG00845020940	1590	1405	1587	1532
ENSCAFG00845005311	188	178	188	238
ENSCAFG00845007974	0	0	0	2
ENSCAFG00845005312	0	0	0	0
ENSCAFG00845007973	491	409	520	494
ENSCAFG00845005313	0	0	0	0
ENSCAFG00845007976	1139	1119	1124	1065
ENSCAFG00845005314	0	0	0	0
ENSCAFG00845007975	486	444	442	449
ENSCAFG00845005315	0	0	0	0
ENSCAFG00845007978	532	533	632	645
ENSCAFG00845005316	0	0	0	0
ENSCAFG00845007977	0	0	0	0
ENSCAFG00845005317	0	1	0	0
ENSCAFG00845005318	0	0	0	0
ENSCAFG00845007979	4	6	8	2

ENSCAFG00845005319	797	778	732	758
ENSCAFG00845030393	0	0	0	0
ENSCAFG00845030394	758	688	636	635
ENSCAFG00845020938	1	0	5	0
ENSCAFG00845030395	0	0	0	0
ENSCAFG00845020939	0	1	0	0
ENSCAFG00845030396	0	0	0	0
ENSCAFG00845019950	119	137	134	153
ENSCAFG00845030397	0	0	0	0
ENSCAFG00845019951	48	41	57	31
ENSCAFG00845030398	14	11	10	19
ENSCAFG00845019952	0	1	3	5
ENSCAFG00845030399	14	14	16	16
ENSCAFG00845019953	0	0	0	0
ENSCAFG00845019943	1	0	0	1
ENSCAFG00845020932	245	197	197	211
ENSCAFG00845019944	17042	16540	14799	15362
ENSCAFG00845020933	9032	9173	9074	9688
ENSCAFG00845019945	252	227	327	329
ENSCAFG00845020930	0	0	0	0
ENSCAFG00845019946	0	2	2	5
ENSCAFG00845020931	0	0	0	0
ENSCAFG00845019947	0	0	0	0
ENSCAFG00845020936	7	10	7	14
ENSCAFG00845019948	848	795	746	767
ENSCAFG00845020937	0	0	0	0
ENSCAFG00845019949	931	984	947	1046
ENSCAFG00845020934	0	0	0	0
ENSCAFG00845020935	66	48	86	71
ENSCAFG00845007961	6	3	9	6
ENSCAFG00845007960	0	0	0	0
ENSCAFG00845005300	1254	1225	1191	1175
ENSCAFG00845007963	1	1	2	0
ENSCAFG00845005301	0	0	0	0
ENSCAFG00845007962	0	0	0	0
ENSCAFG00845005302	977	931	1053	960
ENSCAFG00845007965	599	552	495	532
ENSCAFG00845005303	101	89	71	91
ENSCAFG00845007964	1	3	1	4
ENSCAFG00845005304	0	2	1	3
ENSCAFG00845007967	308	275	220	228

ENSCAFG00845005305	158	104	96	152
ENSCAFG00845007966	1185	1227	1147	1151
ENSCAFG00845005306	0	0	0	0
ENSCAFG00845007969	359	387	404	351
ENSCAFG00845005307	0	0	0	0
ENSCAFG00845007968	254	253	243	241
ENSCAFG00845005308	0	0	0	0
ENSCAFG00845005309	461	450	387	407
ENSCAFG00845020929	275	246	249	262
ENSCAFG00845020927	0	0	0	0
ENSCAFG00845020928	251	228	296	312
ENSCAFG00845019940	0	0	0	0
ENSCAFG00845019941	91	72	82	107
ENSCAFG00845019942	0	3	0	0
ENSCAFG00845029399	0	0	0	0
ENSCAFG00845029397	0	0	0	0
ENSCAFG00845029398	948	875	886	893
ENSCAFG00845029395	0	2	0	0
ENSCAFG00845029396	181	192	191	172
ENSCAFG00845029393	4	1	1	1
ENSCAFG00845029394	0	1	0	0
ENSCAFG00845029391	7	13	16	10
ENSCAFG00845029392	0	0	0	1
ENSCAFG00845029390	0	0	1	1
ENSCAFG00845029388	2728	2608	2760	2774
ENSCAFG00845029389	5411	5018	5029	5182
ENSCAFG00845029386	0	0	0	1
ENSCAFG00845029387	0	1	1	1
ENSCAFG00845029384	0	0	0	0
ENSCAFG00845029385	1511	1434	1266	1432
ENSCAFG00845029382	8	2	5	4
ENSCAFG00845029383	706	744	745	779
ENSCAFG00845029380	11	12	19	17
ENSCAFG00845029381	0	0	0	0
ENSCAFG00845017399	0	0	0	0
ENSCAFG00845017398	337	347	468	470
ENSCAFG00845017397	2	1	4	0
ENSCAFG00845017396	0	0	0	0
ENSCAFG00845017395	20	16	13	10
ENSCAFG00845017394	0	0	0	0
ENSCAFG00845017393	1136	1020	1082	1054

ENSCAFG00845017392	1051	1041	876	905
ENSCAFG00845017391	29	40	40	36
ENSCAFG00845017390	0	0	0	0
ENSCAFG00845029379	5	5	3	5
ENSCAFG00845029377	3	4	1	4
ENSCAFG00845029378	0	0	3	0
ENSCAFG00845029375	448	420	421	421
ENSCAFG00845029376	8	10	5	11
ENSCAFG00845029373	16	10	10	9
ENSCAFG00845029374	16	21	22	28
ENSCAFG00845029371	515	505	531	529
ENSCAFG00845029372	1	3	6	2
ENSCAFG00845029370	667	727	713	680
ENSCAFG00845017389	3254	3136	3047	3025
ENSCAFG00845017388	1	0	0	0
ENSCAFG00845017387	145	87	123	115
ENSCAFG00845017386	0	0	0	0
ENSCAFG00845017385	0	0	0	0
ENSCAFG00845017384	118	127	108	92
ENSCAFG00845017383	779	789	772	838
ENSCAFG00845017382	4988	4886	5042	5182
ENSCAFG00845017381	281	227	238	280
ENSCAFG00845017380	78	112	84	68
ENSCAFG00845029368	0	0	0	0
ENSCAFG00845030302	46	43	53	41
ENSCAFG00845029369	1275	1219	1300	1237
ENSCAFG00845030303	11	17	21	26
ENSCAFG00845029366	1240	1251	1232	1262
ENSCAFG00845030304	10	15	24	17
ENSCAFG00845029367	3146	2881	2830	2859
ENSCAFG00845030305	455	358	475	478
ENSCAFG00845029364	180	162	142	160
ENSCAFG00845030306	0	0	0	0
ENSCAFG00845029365	1	0	0	2
ENSCAFG00845030307	0	0	0	0
ENSCAFG00845029362	0	0	0	0
ENSCAFG00845030308	15	22	13	15
ENSCAFG00845029363	0	6	0	0
ENSCAFG00845030309	922	950	849	899
ENSCAFG00845029360	35	25	44	33
ENSCAFG00845029361	5	2	2	6

ENSCAFG00845030300	1002	977	1036	1049
ENSCAFG00845030301	0	0	0	0
ENSCAFG00845017379	3167	2902	2956	3058
ENSCAFG00845017378	0	0	0	0
ENSCAFG00845017377	3	5	0	2
ENSCAFG00845017376	0	0	2	0
ENSCAFG00845017375	1	0	0	0
ENSCAFG00845005390	1969	1955	2093	1840
ENSCAFG00845017374	16	23	27	23
ENSCAFG00845005391	15	3	16	8
ENSCAFG00845017373	0	0	0	0
ENSCAFG00845005392	0	0	0	0
ENSCAFG00845017372	1	0	0	0
ENSCAFG00845005393	0	0	0	0
ENSCAFG00845017371	0	0	0	0
ENSCAFG00845005394	7	1	8	10
ENSCAFG00845017370	13	15	6	10
ENSCAFG00845005395	453	405	404	377
ENSCAFG00845005396	0	0	0	0
ENSCAFG00845005397	1	3	0	3
ENSCAFG00845005398	2377	2418	2199	2237
ENSCAFG00845005399	0	0	0	0
ENSCAFG00845029359	0	0	0	0
ENSCAFG00845029357	0	2	0	0
ENSCAFG00845030313	461	391	346	335
ENSCAFG00845029358	0	0	1	4
ENSCAFG00845030314	0	0	0	0
ENSCAFG00845029355	2320	2311	2175	2152
ENSCAFG00845030315	0	0	0	0
ENSCAFG00845029356	0	0	0	0
ENSCAFG00845030316	915	893	1167	1071
ENSCAFG00845029353	13	10	7	6
ENSCAFG00845030317	0	0	0	0
ENSCAFG00845029354	20	21	28	14
ENSCAFG00845030318	12	7	1	11
ENSCAFG00845029351	8	5	7	11
ENSCAFG00845030319	7	8	11	6
ENSCAFG00845029352	448	379	367	370
ENSCAFG00845029350	0	0	0	0
ENSCAFG00845030310	7	4	0	2
ENSCAFG00845030311	0	0	2	0

ENSCAFG00845030312	0	0	1	0
ENSCAFG00845017368	2	3	1	1
ENSCAFG00845017367	3	2	4	5
ENSCAFG00845017366	0	1	0	4
ENSCAFG00845017365	0	2	1	0
ENSCAFG00845017364	0	3	4	1
ENSCAFG00845017363	11	19	11	22
ENSCAFG00845005380	527	520	512	514
ENSCAFG00845017362	0	0	0	0
ENSCAFG00845005381	8	0	5	6
ENSCAFG00845017361	0	0	0	0
ENSCAFG00845005382	0	0	0	0
ENSCAFG00845017360	0	0	0	0
ENSCAFG00845005383	1	2	1	0
ENSCAFG00845005384	0	0	0	0
ENSCAFG00845005385	25	21	20	18
ENSCAFG00845005386	2	0	1	5
ENSCAFG00845005387	1794	1701	1636	1577
ENSCAFG00845005388	1	0	0	0
ENSCAFG00845029348	972	1015	1107	1088
ENSCAFG00845005389	898	944	1024	1112
ENSCAFG00845029349	106	108	105	113
ENSCAFG00845029346	456	418	419	385
ENSCAFG00845030324	2	0	2	4
ENSCAFG00845029347	31	25	41	51
ENSCAFG00845030325	16	22	11	31
ENSCAFG00845029344	6	0	1	5
ENSCAFG00845030326	1120	1148	1162	1208
ENSCAFG00845029345	1814	1778	1639	1644
ENSCAFG00845030327	64	42	96	85
ENSCAFG00845029342	112	139	105	159
ENSCAFG00845030328	462	503	454	404
ENSCAFG00845029343	0	0	0	0
ENSCAFG00845030329	1621	1635	1362	1442
ENSCAFG00845029340	0	0	0	0
ENSCAFG00845029341	178	173	225	228
ENSCAFG00845030320	2	0	0	0
ENSCAFG00845030321	0	0	0	0
ENSCAFG00845030322	0	1	0	0
ENSCAFG00845017369	0	2	3	0
ENSCAFG00845030323	5	3	5	3

ENSCAFG00845019932	0	0	0	0
ENSCAFG00845020921	1080	1042	894	971
ENSCAFG00845019933	0	0	0	0
ENSCAFG00845020922	0	0	0	0
ENSCAFG00845019934	1828	1704	1959	1760
ENSCAFG00845019935	0	0	0	0
ENSCAFG00845020920	209	164	172	148
ENSCAFG00845019936	0	0	0	0
ENSCAFG00845020925	1029	943	887	777
ENSCAFG00845030290	9	6	11	11
ENSCAFG00845019937	6	8	5	2
ENSCAFG00845020926	0	0	0	0
ENSCAFG00845030291	0	0	0	0
ENSCAFG00845019938	0	0	0	0
ENSCAFG00845020923	0	0	0	0
ENSCAFG00845030292	301	235	296	279
ENSCAFG00845019939	274	238	235	233
ENSCAFG00845020924	63	62	52	69
ENSCAFG00845030293	0	0	0	0
ENSCAFG00845007950	0	0	0	0
ENSCAFG00845007952	2161	2253	2471	2506
ENSCAFG00845007951	1005	947	951	962
ENSCAFG00845007954	840	856	789	893
ENSCAFG00845007953	3	1	4	1
ENSCAFG00845007956	104	74	84	59
ENSCAFG00845007955	13	11	9	19
ENSCAFG00845007958	1218	1230	1168	1065
ENSCAFG00845007957	0	0	0	0
ENSCAFG00845007959	251	239	280	274
ENSCAFG00845020918	0	0	2	2
ENSCAFG00845030294	6468	6230	6810	6910
ENSCAFG00845020919	0	0	0	0
ENSCAFG00845030295	805	731	825	798
ENSCAFG00845020916	0	0	0	0
ENSCAFG00845030296	0	0	0	0
ENSCAFG00845020917	0	0	0	0
ENSCAFG00845030297	7	7	6	4
ENSCAFG00845030298	132	167	186	164
ENSCAFG00845030299	56	58	79	85
ENSCAFG00845019930	0	0	0	0
ENSCAFG00845019931	61	63	66	61

ENSCAFG00845019921	1225	1099	1125	1059
ENSCAFG00845020910	0	0	0	0
ENSCAFG00845019922	0	0	3	1
ENSCAFG00845020911	1	2	0	0
ENSCAFG00845019923	173	147	121	147
ENSCAFG00845019924	551	497	647	596
ENSCAFG00845019925	0	0	0	0
ENSCAFG00845020914	0	0	0	1
ENSCAFG00845019926	0	0	0	0
ENSCAFG00845020915	0	0	0	0
ENSCAFG00845019927	0	2	0	1
ENSCAFG00845020912	0	0	0	0
ENSCAFG00845019928	1	0	0	1
ENSCAFG00845020913	0	0	0	0
ENSCAFG00845019929	3393	3224	2997	2877
ENSCAFG00845007941	0	0	0	0
ENSCAFG00845007940	251	233	244	230
ENSCAFG00845007943	28	31	32	31
ENSCAFG00845007942	0	2	3	5
ENSCAFG00845007945	0	4	0	1
ENSCAFG00845007944	0	0	0	0
ENSCAFG00845007947	3	0	1	1
ENSCAFG00845007946	0	1	0	0
ENSCAFG00845007949	35	42	29	29
ENSCAFG00845007948	2357	2170	2153	2198
ENSCAFG00845020907	108	118	124	79
ENSCAFG00845020908	0	0	0	0
ENSCAFG00845020905	0	0	0	0
ENSCAFG00845020906	0	0	0	0
ENSCAFG00845020909	0	0	0	0
ENSCAFG00845019920	846	835	820	842
ENSCAFG00845019910	0	0	0	0
ENSCAFG00845019911	177	159	203	198
ENSCAFG00845020900	0	0	0	0
ENSCAFG00845019912	0	0	0	0
ENSCAFG00845019913	0	3	1	2
ENSCAFG00845019914	0	0	0	0
ENSCAFG00845020903	0	0	0	0
ENSCAFG00845019915	0	0	0	0
ENSCAFG00845020904	535	477	501	533
ENSCAFG00845019916	0	0	0	0

ENSCAFG00845020901	24	17	17	13
ENSCAFG00845019917	0	0	0	0
ENSCAFG00845020902	0	0	0	0
ENSCAFG00845019918	0	0	1	0
ENSCAFG00845019919	622	565	645	640
ENSCAFG00845007930	0	0	0	5
ENSCAFG00845007932	0	0	0	0
ENSCAFG00845007931	0	0	2	0
ENSCAFG00845007934	2118	1979	2203	2231
ENSCAFG00845007933	44	30	32	22
ENSCAFG00845007936	0	0	0	0
ENSCAFG00845007935	232	215	207	198
ENSCAFG00845007938	0	0	0	0
ENSCAFG00845007937	71	84	66	81
ENSCAFG00845007939	2	1	0	1
ENSCAFG00845019900	0	0	0	0
ENSCAFG00845019901	247	274	225	219
ENSCAFG00845019902	0	0	0	0
ENSCAFG00845019903	0	0	0	0
ENSCAFG00845019904	0	0	0	0
ENSCAFG00845019905	0	0	0	0
ENSCAFG00845019906	0	0	0	0
ENSCAFG00845019907	0	0	0	0
ENSCAFG00845019908	56	60	34	28
ENSCAFG00845019909	566	543	598	658
ENSCAFG00845007921	0	0	0	0
ENSCAFG00845007920	38	35	37	36
ENSCAFG00845007923	0	0	0	0
ENSCAFG00845007922	203	191	182	185
ENSCAFG00845007925	0	0	0	0
ENSCAFG00845007924	3963	3696	3598	3610
ENSCAFG00845007927	2116	2102	2172	2242
ENSCAFG00845007926	1	0	0	0
ENSCAFG00845007929	0	0	0	4
ENSCAFG00845007928	174	144	171	163
ENSCAFG00845007910	0	0	0	0
ENSCAFG00845007912	32	56	55	48
ENSCAFG00845007911	1375	1299	1237	1288
ENSCAFG00845007914	16314	16143	16854	16398
ENSCAFG00845007913	625	607	538	524
ENSCAFG00845007916	0	0	0	0

ENSCAFG00845007915	1	0	0	0
ENSCAFG00845007918	0	0	0	0
ENSCAFG00845007917	2	1	1	6
ENSCAFG00845007919	18	5	17	17
ENSCAFG00845007901	4	1	3	3
ENSCAFG00845007900	1	6	0	0
ENSCAFG00845007903	2	0	0	0
ENSCAFG00845007902	0	0	0	0
ENSCAFG00845007905	0	0	0	0
ENSCAFG00845007904	193	179	192	199
ENSCAFG00845007907	0	0	0	0
ENSCAFG00845007906	0	0	0	0
ENSCAFG00845007909	0	0	0	0
ENSCAFG00845007908	0	0	0	0
ENSCAFG00845017478	3	3	0	1
ENSCAFG00845017477	0	0	0	0
ENSCAFG00845017476	0	0	0	0
ENSCAFG00845017475	6	3	4	8
ENSCAFG00845017474	260	215	246	273
ENSCAFG00845017473	0	0	0	0
ENSCAFG00845005490	0	0	0	0
ENSCAFG00845017472	0	0	0	0
ENSCAFG00845005491	240	213	221	275
ENSCAFG00845017471	790	716	793	777
ENSCAFG00845005492	0	0	0	0
ENSCAFG00845017470	783	739	770	669
ENSCAFG00845005493	1807	1728	1635	1726
ENSCAFG00845005494	0	0	0	0
ENSCAFG00845005495	20643	19627	20405	21300
ENSCAFG00845005496	0	0	0	0
ENSCAFG00845005497	0	0	0	0
ENSCAFG00845005498	994	899	963	1004
ENSCAFG00845029458	374	316	336	333
ENSCAFG00845005499	598	472	506	534
ENSCAFG00845029459	213	180	197	203
ENSCAFG00845029456	0	0	0	0
ENSCAFG00845030456	292	256	394	362
ENSCAFG00845029457	0	0	0	0
ENSCAFG00845030457	0	0	0	0
ENSCAFG00845029454	0	0	0	0
ENSCAFG00845030458	66	57	47	35

ENSCAFG00845029455	483	478	473	521
ENSCAFG00845030459	1275	1224	880	967
ENSCAFG00845029452	2	3	2	3
ENSCAFG00845029453	0	0	0	0
ENSCAFG00845029450	1	1	0	0
ENSCAFG00845029451	0	0	0	0
ENSCAFG00845030450	23	18	5	12
ENSCAFG00845030451	0	0	0	0
ENSCAFG00845030452	0	0	0	0
ENSCAFG00845030453	5	4	2	6
ENSCAFG00845030454	0	0	0	0
ENSCAFG00845017479	0	0	0	0
ENSCAFG00845030455	2	0	0	0
ENSCAFG00845017467	0	0	0	0
ENSCAFG00845017466	0	0	0	0
ENSCAFG00845017465	0	0	0	0
ENSCAFG00845017464	2325	2101	2402	2503
ENSCAFG00845017463	2	4	5	4
ENSCAFG00845017462	0	0	0	0
ENSCAFG00845017461	0	0	0	0
ENSCAFG00845005480	0	0	3	1
ENSCAFG00845017460	4	2	3	4
ENSCAFG00845005481	0	0	0	0
ENSCAFG00845005482	0	0	0	0
ENSCAFG00845005483	6	0	4	1
ENSCAFG00845005484	258	246	193	221
ENSCAFG00845005485	61	62	65	79
ENSCAFG00845029449	0	0	0	0
ENSCAFG00845005486	0	0	0	0
ENSCAFG00845005487	2412	2389	2468	2638
ENSCAFG00845029447	494	542	500	475
ENSCAFG00845005488	13042	12550	12934	12769
ENSCAFG00845029448	1102	1163	1097	1038
ENSCAFG00845005489	0	0	0	0
ENSCAFG00845029445	173	186	165	169
ENSCAFG00845030467	0	0	0	0
ENSCAFG00845029446	0	0	0	0
ENSCAFG00845030468	852	890	867	880
ENSCAFG00845029443	2870	2916	2632	2639
ENSCAFG00845030469	0	0	0	0
ENSCAFG00845029444	0	0	0	0

ENSCAFG00845029441	0	0	0	0
ENSCAFG00845029442	10	5	15	6
ENSCAFG00845029440	0	0	0	0
ENSCAFG00845030460	5	2	1	5
ENSCAFG00845030461	1	0	0	0
ENSCAFG00845030462	0	0	7	0
ENSCAFG00845030463	0	0	0	0
ENSCAFG00845030464	1655	1739	1509	1478
ENSCAFG00845017469	1388	1408	1527	1638
ENSCAFG00845030465	15	15	16	16
ENSCAFG00845017468	8	4	6	7
ENSCAFG00845030466	1	3	2	2
ENSCAFG00845017456	0	0	3	0
ENSCAFG00845017455	1971	1915	1746	1837
ENSCAFG00845017454	0	0	0	4
ENSCAFG00845017453	0	0	1	0
ENSCAFG00845017452	0	0	0	0
ENSCAFG00845017451	0	0	2	0
ENSCAFG00845017450	516	534	535	463
ENSCAFG00845005470	2	0	2	6
ENSCAFG00845005471	0	0	0	0
ENSCAFG00845005472	704	647	703	714
ENSCAFG00845005473	292	244	285	256
ENSCAFG00845005474	17	25	29	15
ENSCAFG00845029438	95	79	68	65
ENSCAFG00845005475	1785	1866	1539	1498
ENSCAFG00845029439	0	1	2	1
ENSCAFG00845005476	2060	2052	1798	1848
ENSCAFG00845029436	4	2	1	2
ENSCAFG00845005477	0	3	0	0
ENSCAFG00845029437	24	33	18	25
ENSCAFG00845005478	0	0	0	0
ENSCAFG00845029434	858	825	840	810
ENSCAFG00845030478	2059	1860	2001	2156
ENSCAFG00845005479	0	0	0	0
ENSCAFG00845029435	1308	1168	1301	1257
ENSCAFG00845030479	698	668	761	807
ENSCAFG00845029432	0	0	0	0
ENSCAFG00845029433	931	930	889	914
ENSCAFG00845029430	0	0	0	0
ENSCAFG00845029431	0	0	0	0

ENSCAFG00845030470	0	0	0	0
ENSCAFG00845030471	19537	18743	18681	19229
ENSCAFG00845030472	70	36	41	25
ENSCAFG00845030473	150	145	180	143
ENSCAFG00845030474	1638	1600	1499	1487
ENSCAFG00845017459	1	1	1	0
ENSCAFG00845030475	226	239	228	218
ENSCAFG00845017458	0	0	1	0
ENSCAFG00845030476	0	0	0	0
ENSCAFG00845017457	0	0	0	0
ENSCAFG00845030477	622	587	632	662
ENSCAFG00845017445	0	0	0	0
ENSCAFG00845017444	0	0	0	0
ENSCAFG00845017443	0	0	0	0
ENSCAFG00845017442	463	453	600	538
ENSCAFG00845017441	0	0	0	0
ENSCAFG00845017440	7	0	1	4
ENSCAFG00845030480	13	12	12	5
ENSCAFG00845005460	0	0	0	0
ENSCAFG00845005461	137	166	163	163
ENSCAFG00845029429	0	0	0	0
ENSCAFG00845005462	1	0	0	0
ENSCAFG00845005463	5827	5672	5360	5241
ENSCAFG00845029427	1934	1937	1758	1770
ENSCAFG00845005464	368	364	384	397
ENSCAFG00845029428	0	0	1	0
ENSCAFG00845005465	0	0	0	0
ENSCAFG00845029425	1734	1779	1795	1800
ENSCAFG00845005466	851	806	865	978
ENSCAFG00845029426	331	336	317	316
ENSCAFG00845005467	1	4	2	1
ENSCAFG00845029423	5	5	6	2
ENSCAFG00845030489	0	0	0	0
ENSCAFG00845005468	4	5	6	3
ENSCAFG00845029424	687	713	576	628
ENSCAFG00845005469	0	0	0	0
ENSCAFG00845029421	0	0	0	0
ENSCAFG00845029422	0	0	0	0
ENSCAFG00845029420	0	0	0	0
ENSCAFG00845030481	427	377	370	404
ENSCAFG00845030482	0	0	0	0

ENSCAFG00845030483	0	0	0	0
ENSCAFG00845030484	1	3	0	1
ENSCAFG00845017449	555	555	581	564
ENSCAFG00845030485	0	3	0	0
ENSCAFG00845017448	76	65	98	96
ENSCAFG00845030486	0	0	0	0
ENSCAFG00845017447	0	0	0	0
ENSCAFG00845030487	0	0	0	0
ENSCAFG00845017446	0	0	0	0
ENSCAFG00845030488	1312	1297	1032	1038
ENSCAFG00845017434	5047	4898	5214	5018
ENSCAFG00845017433	162	157	152	187
ENSCAFG00845017432	0	0	0	0
ENSCAFG00845017431	0	1	1	0
ENSCAFG00845017430	0	0	0	0
ENSCAFG00845030490	0	0	0	0
ENSCAFG00845030491	2815	2649	2586	2562
ENSCAFG00845005450	0	0	0	0
ENSCAFG00845029418	0	0	0	0
ENSCAFG00845005451	47	67	57	53
ENSCAFG00845029419	0	0	0	0
ENSCAFG00845005452	39	37	33	51
ENSCAFG00845029416	10	17	17	8
ENSCAFG00845005453	1056	978	881	865
ENSCAFG00845029417	2432	2402	2251	2269
ENSCAFG00845005454	22404	21569	20218	20041
ENSCAFG00845029414	0	0	0	0
ENSCAFG00845005455	1	0	1	0
ENSCAFG00845029415	0	0	0	0
ENSCAFG00845005456	1	1	1	0
ENSCAFG00845029412	1	4	0	0
ENSCAFG00845005457	0	0	0	0
ENSCAFG00845029413	23	15	10	11
ENSCAFG00845005458	13	27	13	21
ENSCAFG00845029410	58	47	46	36
ENSCAFG00845005459	1196	1313	1072	1259
ENSCAFG00845029411	8	7	14	13
ENSCAFG00845030492	285	242	267	249
ENSCAFG00845030493	0	0	0	0
ENSCAFG00845030494	0	0	0	0
ENSCAFG00845017439	2	2	1	1

ENSCAFG00845030495	1866	1706	2041	1950
ENSCAFG00845017438	61	76	78	81
ENSCAFG00845030496	0	0	0	0
ENSCAFG00845017437	243	221	272	260
ENSCAFG00845030497	0	0	0	0
ENSCAFG00845017436	17	20	25	34
ENSCAFG00845030498	3042	2843	2848	2875
ENSCAFG00845017435	0	0	0	0
ENSCAFG00845030499	0	0	0	0
ENSCAFG00845017423	1	8	9	7
ENSCAFG00845017422	0	0	0	0
ENSCAFG00845017421	824	784	756	776
ENSCAFG00845017420	278	308	299	220
ENSCAFG00845029409	2	0	1	0
ENSCAFG00845029407	0	0	0	0
ENSCAFG00845005440	731	762	690	748
ENSCAFG00845029408	885	870	775	789
ENSCAFG00845005441	6	1	14	10
ENSCAFG00845029405	24	22	48	31
ENSCAFG00845005442	166	146	161	196
ENSCAFG00845029406	0	0	0	0
ENSCAFG00845005443	0	0	0	0
ENSCAFG00845029403	0	0	0	0
ENSCAFG00845005444	0	1	1	0
ENSCAFG00845029404	910	788	878	877
ENSCAFG00845005445	249	259	232	264
ENSCAFG00845029401	0	1	1	0
ENSCAFG00845005446	1	0	0	1
ENSCAFG00845029402	1	1	0	0
ENSCAFG00845005447	0	0	0	3
ENSCAFG00845005448	0	1	0	0
ENSCAFG00845029400	25	27	10	23
ENSCAFG00845005449	0	0	0	1
ENSCAFG00845017429	0	2	0	0
ENSCAFG00845017428	0	0	0	0
ENSCAFG00845017427	113	107	81	95
ENSCAFG00845017426	6671	6415	5938	5748
ENSCAFG00845017425	0	0	0	0
ENSCAFG00845017424	0	0	0	0
ENSCAFG00845017412	0	0	0	0
ENSCAFG00845017411	1768	1750	1894	1843

ENSCAFG00845017410	1026	998	1000	922
ENSCAFG00845005430	0	0	0	0
ENSCAFG00845005431	307	272	308	313
ENSCAFG00845005432	4	0	2	4
ENSCAFG00845005433	10	7	14	12
ENSCAFG00845005434	0	0	0	0
ENSCAFG00845005435	276	227	271	245
ENSCAFG00845005436	450	381	528	516
ENSCAFG00845005437	0	0	0	0
ENSCAFG00845005438	0	3	0	1
ENSCAFG00845005439	3	0	2	1
ENSCAFG00845017419	0	0	0	0
ENSCAFG00845017418	0	0	0	0
ENSCAFG00845017417	0	0	0	0
ENSCAFG00845017416	0	0	1	0
ENSCAFG00845017415	37	30	36	36
ENSCAFG00845017414	1383	1298	1125	1317
ENSCAFG00845017413	0	0	0	0
ENSCAFG00845017401	0	0	0	0
ENSCAFG00845017400	1334	1295	1339	1291
ENSCAFG00845005420	0	0	0	0
ENSCAFG00845005421	0	0	0	0
ENSCAFG00845005422	349	344	403	409
ENSCAFG00845005423	0	0	0	0
ENSCAFG00845005424	5	8	16	12
ENSCAFG00845005425	37	37	47	32
ENSCAFG00845005426	0	0	0	0
ENSCAFG00845005427	0	0	0	0
ENSCAFG00845005428	302	294	407	380
ENSCAFG00845005429	0	0	0	0
ENSCAFG00845017409	0	0	0	1
ENSCAFG00845017408	0	0	0	0
ENSCAFG00845017407	0	0	0	0
ENSCAFG00845017406	0	0	0	0
ENSCAFG00845017405	0	0	0	0
ENSCAFG00845017404	0	0	0	0
ENSCAFG00845017403	299	331	230	255
ENSCAFG00845017402	0	0	0	0
ENSCAFG00845030401	0	0	0	0
ENSCAFG00845030402	1	0	0	0
ENSCAFG00845030403	2	0	0	0

ENSCAFG00845030404	553	511	402	483
ENSCAFG00845030405	694	696	631	685
ENSCAFG00845030406	0	0	0	0
ENSCAFG00845030407	772	710	644	712
ENSCAFG00845030408	0	0	0	0
ENSCAFG00845030400	0	0	0	0
ENSCAFG00845030409	0	0	0	0
ENSCAFG00845030412	795	766	784	772
ENSCAFG00845030413	0	0	0	0
ENSCAFG00845029498	0	0	0	0
ENSCAFG00845030414	93	82	94	105
ENSCAFG00845029499	232	188	189	184
ENSCAFG00845030415	3	6	1	4
ENSCAFG00845029496	24	11	13	26
ENSCAFG00845030416	12	16	13	12
ENSCAFG00845029497	904	754	740	836
ENSCAFG00845030417	0	0	0	0
ENSCAFG00845029494	668	694	662	701
ENSCAFG00845030418	1	0	2	3
ENSCAFG00845029495	0	0	0	0
ENSCAFG00845030419	0	0	0	0
ENSCAFG00845029492	63	47	54	65
ENSCAFG00845029493	559	541	568	602
ENSCAFG00845029490	0	0	0	0
ENSCAFG00845029491	408	395	363	356
ENSCAFG00845030410	0	0	0	1
ENSCAFG00845030411	0	0	0	0
ENSCAFG00845029489	1679	1543	1618	1672
ENSCAFG00845030423	26	22	17	49
ENSCAFG00845030424	0	0	0	0
ENSCAFG00845029487	5	0	1	0
ENSCAFG00845030425	10	6	9	18
ENSCAFG00845029488	276	249	408	454
ENSCAFG00845030426	0	4	3	1
ENSCAFG00845029485	0	1	0	0
ENSCAFG00845030427	43	66	54	68
ENSCAFG00845029486	0	0	0	0
ENSCAFG00845030428	0	0	0	0
ENSCAFG00845029483	0	0	0	0
ENSCAFG00845030429	0	0	0	0
ENSCAFG00845029484	40	36	32	46

ENSCAFG00845029481	902	866	1068	1067
ENSCAFG00845029482	34	26	30	21
ENSCAFG00845029480	7	3	4	0
ENSCAFG00845030420	2	0	1	2
ENSCAFG00845030421	0	0	0	0
ENSCAFG00845030422	0	0	0	0
ENSCAFG00845017499	0	0	0	0
ENSCAFG00845017498	1	0	0	0
ENSCAFG00845017497	4	6	3	2
ENSCAFG00845017496	1	2	11	8
ENSCAFG00845017495	34	29	28	28
ENSCAFG00845017494	137	148	168	179
ENSCAFG00845017493	81	68	49	58
ENSCAFG00845017492	0	0	0	0
ENSCAFG00845017491	737	730	798	848
ENSCAFG00845017490	1	3	0	1
ENSCAFG00845029478	13	7	6	17
ENSCAFG00845030434	352	321	385	417
ENSCAFG00845029479	0	0	0	0
ENSCAFG00845030435	284	281	278	243
ENSCAFG00845029476	0	0	0	0
ENSCAFG00845030436	0	1	0	0
ENSCAFG00845029477	0	0	0	0
ENSCAFG00845030437	0	0	0	0
ENSCAFG00845029474	2398	2361	2048	1948
ENSCAFG00845030438	0	0	0	0
ENSCAFG00845029475	82	111	77	74
ENSCAFG00845030439	27	28	10	22
ENSCAFG00845029472	0	0	0	0
ENSCAFG00845029473	408	399	505	473
ENSCAFG00845029470	0	0	0	0
ENSCAFG00845029471	0	0	0	0
ENSCAFG00845030430	4	0	5	2
ENSCAFG00845030431	154	160	102	105
ENSCAFG00845030432	0	0	0	0
ENSCAFG00845030433	0	0	0	1
ENSCAFG00845017489	0	0	0	1
ENSCAFG00845017488	0	0	0	0
ENSCAFG00845017487	0	0	0	0
ENSCAFG00845017486	11	1	14	7
ENSCAFG00845017485	0	0	0	0

ENSCAFG00845017484	493	436	443	435
ENSCAFG00845017483	0	0	0	0
ENSCAFG00845017482	28	29	37	33
ENSCAFG00845017481	0	0	0	0
ENSCAFG00845017480	1	0	1	1
ENSCAFG00845029469	0	0	0	0
ENSCAFG00845029467	567	512	549	512
ENSCAFG00845030445	1280	1322	1448	1329
ENSCAFG00845029468	7	9	11	10
ENSCAFG00845030446	0	0	0	0
ENSCAFG00845029465	0	0	0	0
ENSCAFG00845030447	0	0	0	0
ENSCAFG00845029466	12	6	5	6
ENSCAFG00845030448	46	50	43	37
ENSCAFG00845029463	0	0	0	0
ENSCAFG00845030449	0	0	0	0
ENSCAFG00845029464	0	0	0	0
ENSCAFG00845029461	4599	4572	4560	4372
ENSCAFG00845029462	0	0	0	0
ENSCAFG00845029460	0	0	0	0
ENSCAFG00845030440	0	0	0	0
ENSCAFG00845030441	0	0	0	0
ENSCAFG00845030442	0	0	0	0
ENSCAFG00845030443	0	0	0	0
ENSCAFG00845030444	1	8	4	2
ENSCAFG00845005410	0	0	0	0
ENSCAFG00845005411	473	475	462	410
ENSCAFG00845005412	0	0	0	0
ENSCAFG00845005413	0	0	0	0
ENSCAFG00845005414	76	50	80	49
ENSCAFG00845005415	2038	2029	2005	2114
ENSCAFG00845005416	21	15	32	12
ENSCAFG00845005417	0	0	0	0
ENSCAFG00845005418	0	0	0	1
ENSCAFG00845005419	368	397	349	337
ENSCAFG00845005400	139	156	132	150
ENSCAFG00845005401	1	2	0	1
ENSCAFG00845005402	0	0	0	0
ENSCAFG00845005403	0	0	0	0
ENSCAFG00845005404	0	0	0	0
ENSCAFG00845005405	92	98	99	87

ENSCAFG00845005406	2314	2206	2260	2353
ENSCAFG00845005407	0	0	0	0
ENSCAFG00845005408	0	0	0	0
ENSCAFG00845005409	0	0	0	0
ENSCAFG00845017599	387	307	369	374
ENSCAFG00845017598	0	0	0	0
ENSCAFG00845017597	0	0	0	0
ENSCAFG00845017596	0	0	0	0
ENSCAFG00845017595	0	0	0	0
ENSCAFG00845017594	0	2	1	0
ENSCAFG00845017593	2	3	4	2
ENSCAFG00845017592	0	0	1	2
ENSCAFG00845017591	0	0	0	0
ENSCAFG00845017590	793	800	767	898
ENSCAFG00845029579	1	0	0	0
ENSCAFG00845029577	18	4	31	39
ENSCAFG00845030577	1	0	0	0
ENSCAFG00845029578	2	5	3	5
ENSCAFG00845030578	4	1	2	2
ENSCAFG00845029575	402	381	364	341
ENSCAFG00845030579	0	0	0	0
ENSCAFG00845029576	988	1028	856	905
ENSCAFG00845029573	441	454	454	433
ENSCAFG00845029574	0	0	0	0
ENSCAFG00845029571	85	82	85	75
ENSCAFG00845029572	289	261	241	275
ENSCAFG00845029570	0	0	0	0
ENSCAFG00845030570	144	115	167	125
ENSCAFG00845030571	0	0	0	0
ENSCAFG00845030572	932	954	963	995
ENSCAFG00845030573	0	0	0	0
ENSCAFG00845030574	427	517	541	493
ENSCAFG00845030575	1318	1257	1231	1312
ENSCAFG00845030576	41	50	60	42
ENSCAFG00845017588	0	0	0	0
ENSCAFG00845017587	1520	1503	1667	1594
ENSCAFG00845017586	3	3	3	4
ENSCAFG00845017585	25	29	14	27
ENSCAFG00845017584	3	0	1	0
ENSCAFG00845017583	0	0	0	0
ENSCAFG00845017582	0	0	0	0

ENSCAFG00845017581	3	1	1	0
ENSCAFG00845017580	0	0	0	0
ENSCAFG00845029568	39	39	21	37
ENSCAFG00845029569	140	148	135	170
ENSCAFG00845029566	394	400	485	483
ENSCAFG00845030588	0	0	0	0
ENSCAFG00845029567	0	0	0	1
ENSCAFG00845030589	32	30	18	27
ENSCAFG00845029564	17	9	11	10
ENSCAFG00845029565	1	0	1	0
ENSCAFG00845029562	646	590	569	587
ENSCAFG00845029563	171	167	233	227
ENSCAFG00845029560	0	0	0	0
ENSCAFG00845029561	261	226	212	168
ENSCAFG00845030580	0	0	0	0
ENSCAFG00845030581	0	0	0	0
ENSCAFG00845030582	578	590	578	596
ENSCAFG00845030583	1	2	3	0
ENSCAFG00845030584	74	57	84	74
ENSCAFG00845030585	0	0	0	0
ENSCAFG00845030586	0	0	0	0
ENSCAFG00845017589	37	52	38	56
ENSCAFG00845030587	0	0	0	0
ENSCAFG00845017577	0	0	0	0
ENSCAFG00845017576	0	0	0	0
ENSCAFG00845017575	0	0	0	0
ENSCAFG00845017574	0	0	0	0
ENSCAFG00845017573	106	103	75	107
ENSCAFG00845017572	118	132	82	62
ENSCAFG00845017571	0	0	0	0
ENSCAFG00845005590	2	0	0	1
ENSCAFG00845017570	0	0	0	0
ENSCAFG00845030590	516	515	447	498
ENSCAFG00845005591	0	0	0	0
ENSCAFG00845005592	7	1	0	3
ENSCAFG00845005593	0	0	0	0
ENSCAFG00845005594	0	0	0	0
ENSCAFG00845005595	69	79	63	66
ENSCAFG00845029559	1	0	3	0
ENSCAFG00845005596	512	433	377	383
ENSCAFG00845005597	18	12	25	8

ENSCAFG00845029557	0	0	0	0
ENSCAFG00845005598	5845	5651	5799	5871
ENSCAFG00845029558	0	0	0	0
ENSCAFG00845005599	0	0	0	0
ENSCAFG00845029555	4	2	0	4
ENSCAFG00845030599	1571	1501	1534	1517
ENSCAFG00845029556	0	0	0	0
ENSCAFG00845029553	0	0	0	0
ENSCAFG00845029554	0	0	1	2
ENSCAFG00845029551	0	0	0	0
ENSCAFG00845029552	3	2	1	6
ENSCAFG00845029550	1729	1825	1656	1683
ENSCAFG00845030591	196	247	226	269
ENSCAFG00845030592	0	0	0	0
ENSCAFG00845030593	0	0	0	5
ENSCAFG00845030594	0	0	0	0
ENSCAFG00845030595	0	0	0	0
ENSCAFG00845030596	5	1	3	1
ENSCAFG00845017579	11	14	8	8
ENSCAFG00845030597	148	141	187	222
ENSCAFG00845017578	9	7	3	2
ENSCAFG00845030598	3	0	2	0
ENSCAFG00845017566	5	0	0	1
ENSCAFG00845017565	0	0	0	0
ENSCAFG00845017564	752	727	761	760
ENSCAFG00845017563	0	0	0	0
ENSCAFG00845017562	0	0	0	0
ENSCAFG00845017561	6747	6777	6767	7207
ENSCAFG00845017560	0	0	0	0
ENSCAFG00845005580	2	7	1	1
ENSCAFG00845005581	5250	5189	4539	4504
ENSCAFG00845005582	0	0	0	0
ENSCAFG00845005583	0	0	0	0
ENSCAFG00845005584	0	0	0	0
ENSCAFG00845029548	0	0	0	0
ENSCAFG00845005585	0	0	0	0
ENSCAFG00845029549	88	109	95	90
ENSCAFG00845005586	399	364	433	447
ENSCAFG00845029546	794	806	732	775
ENSCAFG00845005587	380	342	309	374
ENSCAFG00845029547	28	33	17	14

ENSCAFG00845005588	0	0	0	0
ENSCAFG00845029544	0	0	0	0
ENSCAFG00845005589	0	0	0	0
ENSCAFG00845029545	0	0	0	0
ENSCAFG00845029542	2092	2034	1959	2105
ENSCAFG00845029543	696	656	619	707
ENSCAFG00845029540	10	7	12	8
ENSCAFG00845029541	987	1001	872	856
ENSCAFG00845017569	0	0	0	0
ENSCAFG00845017568	0	0	0	0
ENSCAFG00845017567	333	324	265	281
ENSCAFG00845017555	0	1	0	0
ENSCAFG00845017554	4	2	7	6
ENSCAFG00845017553	0	0	0	0
ENSCAFG00845017552	7	12	5	4
ENSCAFG00845017551	23	30	19	31
ENSCAFG00845017550	3	1	0	1
ENSCAFG00845005570	0	0	0	0
ENSCAFG00845005571	593	603	598	477
ENSCAFG00845029539	0	0	0	0
ENSCAFG00845005572	0	0	0	0
ENSCAFG00845005573	5	1	2	1
ENSCAFG00845029537	620	580	527	505
ENSCAFG00845005574	46	46	33	66
ENSCAFG00845029538	0	0	1	0
ENSCAFG00845005575	2	3	3	4
ENSCAFG00845029535	1274	1281	1217	1163
ENSCAFG00845005576	0	0	0	0
ENSCAFG00845029536	1160	1152	1209	1178
ENSCAFG00845005577	0	0	0	0
ENSCAFG00845029533	0	0	0	0
ENSCAFG00845005578	4	4	3	4
ENSCAFG00845029534	1058	1109	1086	1165
ENSCAFG00845005579	2	1	1	1
ENSCAFG00845029531	797	779	808	770
ENSCAFG00845029532	0	0	0	0
ENSCAFG00845029530	1252	1186	1226	1316
ENSCAFG00845017559	332	339	343	323
ENSCAFG00845017558	0	0	0	0
ENSCAFG00845017557	0	0	0	0
ENSCAFG00845017556	2208	2112	1886	2068

ENSCAFG00845017544	0	0	0	0
ENSCAFG00845017543	12	9	12	10
ENSCAFG00845017542	0	0	0	0
ENSCAFG00845017541	0	0	0	0
ENSCAFG00845017540	0	3	3	5
ENSCAFG00845005560	0	0	0	0
ENSCAFG00845029528	2	2	2	0
ENSCAFG00845005561	0	0	0	0
ENSCAFG00845029529	2	0	4	1
ENSCAFG00845005562	57	48	61	59
ENSCAFG00845029526	0	0	0	0
ENSCAFG00845005563	390	434	375	400
ENSCAFG00845029527	1439	1402	1527	1527
ENSCAFG00845005564	418	392	341	425
ENSCAFG00845029524	0	0	0	0
ENSCAFG00845005565	0	0	0	0
ENSCAFG00845029525	1171	1175	1095	1114
ENSCAFG00845005566	0	0	0	0
ENSCAFG00845029522	0	0	0	0
ENSCAFG00845005567	0	0	0	0
ENSCAFG00845029523	20065	20006	20647	20805
ENSCAFG00845005568	39	43	42	41
ENSCAFG00845029520	96	93	97	96
ENSCAFG00845005569	0	0	0	0
ENSCAFG00845029521	0	0	0	0
ENSCAFG00845017549	0	0	0	0
ENSCAFG00845017548	0	1	0	0
ENSCAFG00845017547	31	17	27	36
ENSCAFG00845017546	1070	1039	1040	1045
ENSCAFG00845017545	2	1	2	6
ENSCAFG00845017533	190	169	169	222
ENSCAFG00845017532	0	0	0	0
ENSCAFG00845017531	695	636	663	631
ENSCAFG00845017530	0	0	0	0
ENSCAFG00845029519	0	0	0	0
ENSCAFG00845029517	0	0	0	0
ENSCAFG00845005550	1204	1151	1238	1333
ENSCAFG00845029518	3	0	0	0
ENSCAFG00845005551	0	0	0	0
ENSCAFG00845029515	871	799	730	878
ENSCAFG00845005552	5	3	4	1

ENSCAFG00845029516	145	126	170	169
ENSCAFG00845005553	0	0	0	0
ENSCAFG00845029513	125	98	119	140
ENSCAFG00845005554	0	0	0	0
ENSCAFG00845029514	0	0	0	0
ENSCAFG00845005555	0	0	0	0
ENSCAFG00845029511	0	0	0	0
ENSCAFG00845005556	6	5	10	12
ENSCAFG00845029512	22	24	38	31
ENSCAFG00845005557	44	48	42	32
ENSCAFG00845005558	582	636	482	502
ENSCAFG00845029510	31	18	25	31
ENSCAFG00845005559	54	60	23	32
ENSCAFG00845017539	157	141	102	101
ENSCAFG00845017538	0	0	0	0
ENSCAFG00845017537	2	1	1	4
ENSCAFG00845017536	1311	1267	1233	1291
ENSCAFG00845017535	0	0	0	0
ENSCAFG00845017534	4	0	1	2
ENSCAFG00845017522	10	11	13	17
ENSCAFG00845017521	0	0	0	0
ENSCAFG00845017520	0	0	0	0
ENSCAFG00845029508	320	277	339	291
ENSCAFG00845029509	2499	2354	2345	2313
ENSCAFG00845029506	1	6	2	3
ENSCAFG00845029507	26	33	35	27
ENSCAFG00845005540	33	15	26	20
ENSCAFG00845029504	2	2	1	0
ENSCAFG00845005541	211	230	232	229
ENSCAFG00845029505	15	12	26	12
ENSCAFG00845005542	0	0	0	0
ENSCAFG00845029502	30	19	21	28
ENSCAFG00845005543	0	0	0	1
ENSCAFG00845029503	0	0	0	0
ENSCAFG00845005544	19	10	7	2
ENSCAFG00845029500	0	0	0	0
ENSCAFG00845005545	3	0	1	0
ENSCAFG00845029501	1027	961	1132	1197
ENSCAFG00845005546	521	608	582	568
ENSCAFG00845005547	0	0	0	0
ENSCAFG00845005548	24	19	10	20

ENSCAFG00845005549	0	0	0	0
ENSCAFG00845017529	0	1	0	0
ENSCAFG00845017528	71	58	87	82
ENSCAFG00845017527	3	4	1	3
ENSCAFG00845017526	1909	1767	1985	2086
ENSCAFG00845017525	4425	4312	3500	3275
ENSCAFG00845017524	0	0	0	0
ENSCAFG00845017523	1218	1214	1110	1165
ENSCAFG00845030500	173	161	153	95
ENSCAFG00845030501	0	0	0	0
ENSCAFG00845030502	1400	1302	1271	1277
ENSCAFG00845030503	0	0	0	0
ENSCAFG00845030504	0	0	0	0
ENSCAFG00845030505	0	4	4	0
ENSCAFG00845030506	0	0	0	0
ENSCAFG00845030507	10	7	3	7
ENSCAFG00845030508	0	0	0	0
ENSCAFG00845030509	0	0	0	0
ENSCAFG00845030511	0	0	0	0
ENSCAFG00845030512	0	0	0	0
ENSCAFG00845030513	58	61	46	53
ENSCAFG00845030514	2715	2652	2630	2540
ENSCAFG00845030515	0	0	0	0
ENSCAFG00845030516	10	10	7	7
ENSCAFG00845030517	243	281	261	251
ENSCAFG00845030518	0	0	0	0
ENSCAFG00845030510	0	0	0	2
ENSCAFG00845030519	0	0	0	0
ENSCAFG00845030522	56	42	41	58
ENSCAFG00845030523	2520	2513	2468	2457
ENSCAFG00845030524	0	0	0	0
ENSCAFG00845030525	214	207	177	190
ENSCAFG00845030526	0	0	0	0
ENSCAFG00845030527	2	0	0	0
ENSCAFG00845030528	0	0	0	0
ENSCAFG00845030529	0	0	0	0
ENSCAFG00845030520	7	2	7	6
ENSCAFG00845030521	968	877	1079	1034
ENSCAFG00845030533	42	31	36	17
ENSCAFG00845030534	4	6	8	5
ENSCAFG00845030535	0	0	0	0

ENSCAFG00845030536	0	0	0	0
ENSCAFG00845030537	0	0	0	0
ENSCAFG00845030538	669	554	589	571
ENSCAFG00845030539	0	0	0	0
ENSCAFG00845030530	1527	1561	1455	1542
ENSCAFG00845030531	11	2	11	9
ENSCAFG00845030532	0	0	0	0
ENSCAFG00845030544	0	0	0	0
ENSCAFG00845030545	0	0	0	0
ENSCAFG00845030546	69	62	78	32
ENSCAFG00845030547	3	2	1	1
ENSCAFG00845030548	2554	2331	2401	2525
ENSCAFG00845030549	487	418	565	591
ENSCAFG00845030540	1477	1375	1418	1414
ENSCAFG00845030541	368	373	338	355
ENSCAFG00845030542	0	0	0	0
ENSCAFG00845030543	975	976	783	844
ENSCAFG00845029599	4	3	0	2
ENSCAFG00845030555	0	0	0	0
ENSCAFG00845030556	246	246	233	264
ENSCAFG00845029597	4650	4605	4382	4621
ENSCAFG00845030557	0	0	0	0
ENSCAFG00845029598	0	0	0	0
ENSCAFG00845030558	2482	2522	2341	2330
ENSCAFG00845029595	40	53	31	31
ENSCAFG00845030559	0	1	0	0
ENSCAFG00845029596	0	0	0	0
ENSCAFG00845029593	0	0	0	0
ENSCAFG00845029594	1353	1306	1559	1590
ENSCAFG00845029591	417	401	397	379
ENSCAFG00845029592	2	5	2	1
ENSCAFG00845029590	687	626	972	906
ENSCAFG00845030550	0	0	0	0
ENSCAFG00845030551	0	0	0	0
ENSCAFG00845030552	0	0	0	0
ENSCAFG00845030553	0	0	0	0
ENSCAFG00845030554	537	541	604	667
ENSCAFG00845029588	11	4	10	12
ENSCAFG00845030566	0	0	0	0
ENSCAFG00845029589	0	0	0	0
ENSCAFG00845030567	6	5	11	14

ENSCAFG00845029586	3334	3429	3228	3194
ENSCAFG00845030568	0	0	0	0
ENSCAFG00845029587	251	274	267	285
ENSCAFG00845030569	0	0	0	0
ENSCAFG00845029584	0	0	0	0
ENSCAFG00845029585	5	11	11	14
ENSCAFG00845029582	5576	5310	5210	5368
ENSCAFG00845029583	158	193	143	163
ENSCAFG00845029580	348	365	281	272
ENSCAFG00845029581	148	131	118	133
ENSCAFG00845030560	0	0	0	0
ENSCAFG00845030561	0	0	0	0
ENSCAFG00845030562	25	21	27	23
ENSCAFG00845030563	0	0	0	0
ENSCAFG00845030564	0	0	0	0
ENSCAFG00845030565	879	784	706	723
ENSCAFG00845017511	1	0	1	2
ENSCAFG00845017510	0	0	0	0
ENSCAFG00845005530	67	40	65	71
ENSCAFG00845005531	312	323	267	310
ENSCAFG00845005532	24	20	18	19
ENSCAFG00845005533	42	41	41	37
ENSCAFG00845005534	0	0	0	0
ENSCAFG00845005535	0	0	0	0
ENSCAFG00845005536	14	12	20	18
ENSCAFG00845005537	1694	1555	1906	1988
ENSCAFG00845005538	0	0	0	0
ENSCAFG00845005539	1	2	8	11
ENSCAFG00845017519	0	0	0	0
ENSCAFG00845017518	912	898	828	846
ENSCAFG00845017517	0	0	0	0
ENSCAFG00845017516	44	44	23	31
ENSCAFG00845017515	1043	1037	1079	1082
ENSCAFG00845017514	373	327	289	352
ENSCAFG00845017513	0	0	0	0
ENSCAFG00845017512	1	1	2	0
ENSCAFG00845017500	0	0	0	0
ENSCAFG00845005520	4	2	0	1
ENSCAFG00845005521	0	0	0	0
ENSCAFG00845005522	52	42	65	49
ENSCAFG00845005523	0	0	0	0

ENSCAFG00845005524	11	2	9	14
ENSCAFG00845005525	28	11	28	31
ENSCAFG00845005526	0	4	2	0
ENSCAFG00845005527	0	0	0	0
ENSCAFG00845005528	391	378	339	305
ENSCAFG00845005529	0	0	0	0
ENSCAFG00845017509	1762	1802	1727	1853
ENSCAFG00845017508	0	0	0	0
ENSCAFG00845017507	0	0	0	0
ENSCAFG00845017506	0	0	0	0
ENSCAFG00845017505	54	45	43	69
ENSCAFG00845017504	486	461	366	382
ENSCAFG00845017503	804	792	912	848
ENSCAFG00845017502	0	0	0	0
ENSCAFG00845017501	4	7	6	4
ENSCAFG00845005510	423	483	452	467
ENSCAFG00845005511	229	203	236	220
ENSCAFG00845005512	0	0	0	0
ENSCAFG00845005513	0	0	0	0
ENSCAFG00845005514	532	449	518	643
ENSCAFG00845005515	99	99	41	61
ENSCAFG00845005516	52	40	53	55
ENSCAFG00845005517	0	0	0	0
ENSCAFG00845005518	15	19	22	24
ENSCAFG00845005519	0	0	0	0
ENSCAFG00845005500	549	514	508	456
ENSCAFG00845005501	1	2	0	3
ENSCAFG00845005502	22	13	35	30
ENSCAFG00845005503	955	802	859	789
ENSCAFG00845005504	3	0	2	2
ENSCAFG00845005505	0	0	0	0
ENSCAFG00845005506	2	5	2	0
ENSCAFG00845005507	0	0	0	0
ENSCAFG00845005508	0	0	0	0
ENSCAFG00845005509	12557	12023	11396	11853
ENSCAFG00845015057	58	52	52	63
ENSCAFG00845015058	17	8	30	13
ENSCAFG00845015055	90	102	60	66
ENSCAFG00845015056	0	0	0	1
ENSCAFG00845015053	0	0	0	0
ENSCAFG00845015054	52	55	68	62

ENSCAFG00845015051	52	42	38	47
ENSCAFG00845015052	0	0	0	0
ENSCAFG00845015050	4	3	3	6
ENSCAFG00845027039	2932	2976	2922	3065
ENSCAFG00845027038	0	0	0	0
ENSCAFG00845003069	311	324	251	261
ENSCAFG00845003068	1380	1387	1396	1380
ENSCAFG00845027035	0	0	0	0
ENSCAFG00845029698	4	1	3	1
ENSCAFG00845030698	1414	1490	1380	1318
ENSCAFG00845003067	0	0	0	0
ENSCAFG00845027034	43	52	62	36
ENSCAFG00845029699	339	347	312	306
ENSCAFG00845030699	0	0	0	0
ENSCAFG00845003066	1	0	0	0
ENSCAFG00845027037	1	0	1	3
ENSCAFG00845029696	0	0	2	0
ENSCAFG00845003065	9	19	14	11
ENSCAFG00845027036	893	880	889	973
ENSCAFG00845029697	0	0	0	0
ENSCAFG00845003064	0	0	0	0
ENSCAFG00845027031	0	0	0	0
ENSCAFG00845029694	281	295	279	291
ENSCAFG00845003063	1	2	2	1
ENSCAFG00845027030	0	0	0	0
ENSCAFG00845029695	184	228	295	264
ENSCAFG00845003062	3837	3573	2751	2956
ENSCAFG00845027033	523	536	613	495
ENSCAFG00845029692	8	11	7	2
ENSCAFG00845003061	192	218	182	186
ENSCAFG00845027032	1	0	0	0
ENSCAFG00845029693	1006	964	972	1031
ENSCAFG00845003060	6	8	13	4
ENSCAFG00845029690	175	191	200	185
ENSCAFG00845030690	213	240	189	184
ENSCAFG00845029691	296	255	176	195
ENSCAFG00845030691	0	0	0	0
ENSCAFG00845030692	776	719	768	803
ENSCAFG00845030694	0	6	1	2
ENSCAFG00845030695	1483	1631	1549	1632
ENSCAFG00845015059	53	54	53	69

ENSCAFG00845030696	1	0	0	0
ENSCAFG00845030697	442	411	392	452
ENSCAFG00845015046	0	0	0	0
ENSCAFG00845015047	0	0	0	0
ENSCAFG00845015044	283	310	253	301
ENSCAFG00845015045	0	0	0	0
ENSCAFG00845015042	0	0	0	0
ENSCAFG00845015043	0	0	0	1
ENSCAFG00845015040	399	412	393	433
ENSCAFG00845015041	0	0	0	0
ENSCAFG00845027028	2	7	20	7
ENSCAFG00845027027	3084	2849	3037	3067
ENSCAFG00845029689	31	43	32	34
ENSCAFG00845027029	1964	1946	2044	2095
ENSCAFG00845003079	0	0	0	0
ENSCAFG00845027024	0	4	0	1
ENSCAFG00845029687	0	0	0	3
ENSCAFG00845003078	281	215	234	244
ENSCAFG00845027023	0	0	0	0
ENSCAFG00845029688	0	0	0	0
ENSCAFG00845003077	0	0	0	0
ENSCAFG00845027026	407	345	312	387
ENSCAFG00845029685	116	101	77	69
ENSCAFG00845003076	12	10	5	13
ENSCAFG00845027025	0	0	0	0
ENSCAFG00845029686	0	0	0	0
ENSCAFG00845003075	10	7	2	2
ENSCAFG00845027020	0	0	0	2
ENSCAFG00845029683	17	19	12	13
ENSCAFG00845003074	0	0	0	0
ENSCAFG00845029684	243	220	249	242
ENSCAFG00845003073	0	0	0	0
ENSCAFG00845027022	6023	6034	5869	5459
ENSCAFG00845029681	67	73	76	66
ENSCAFG00845003072	71	55	56	67
ENSCAFG00845027021	0	0	0	0
ENSCAFG00845029682	0	0	0	0
ENSCAFG00845003071	0	0	0	0
ENSCAFG00845003070	9	9	5	4
ENSCAFG00845029680	1	1	0	0
ENSCAFG00845015048	1	0	2	0

ENSCAFG00845015049	30	31	34	33
ENSCAFG00845015035	5	16	22	16
ENSCAFG00845017698	0	0	0	0
ENSCAFG00845015036	2	6	9	7
ENSCAFG00845017697	3	2	0	0
ENSCAFG00845015033	0	0	0	0
ENSCAFG00845017696	0	0	0	0
ENSCAFG00845015034	0	0	0	0
ENSCAFG00845017695	201	182	163	189
ENSCAFG00845015031	0	0	0	0
ENSCAFG00845017694	0	0	0	0
ENSCAFG00845015032	150	118	120	149
ENSCAFG00845017693	0	0	0	0
ENSCAFG00845017692	20	21	27	26
ENSCAFG00845015030	240	221	223	230
ENSCAFG00845017691	1624	1512	1529	1577
ENSCAFG00845017690	0	0	0	0
ENSCAFG00845027017	10	14	13	27
ENSCAFG00845003049	0	0	0	0
ENSCAFG00845027016	22	16	20	30
ENSCAFG00845003048	1	2	0	3
ENSCAFG00845027019	182	182	143	143
ENSCAFG00845029678	0	0	0	0
ENSCAFG00845003047	2	15	6	13
ENSCAFG00845027018	0	1	0	0
ENSCAFG00845029679	1	4	8	4
ENSCAFG00845003046	0	0	0	0
ENSCAFG00845027013	302	377	339	402
ENSCAFG00845029676	246	218	253	205
ENSCAFG00845003045	0	0	0	0
ENSCAFG00845027012	17	16	16	16
ENSCAFG00845029677	221	183	220	295
ENSCAFG00845003044	0	0	0	0
ENSCAFG00845027015	130	128	144	141
ENSCAFG00845029674	553	562	557	588
ENSCAFG00845003043	0	0	0	0
ENSCAFG00845027014	2014	1979	1872	1780
ENSCAFG00845029675	0	1	1	3
ENSCAFG00845003042	332	347	336	286
ENSCAFG00845029672	893	823	869	868
ENSCAFG00845003041	1054	1020	1090	1065

ENSCAFG00845029673	0	0	0	0
ENSCAFG00845003040	0	0	0	0
ENSCAFG00845027011	3	2	0	1
ENSCAFG00845029670	257	253	320	265
ENSCAFG00845027010	55	60	82	67
ENSCAFG00845029671	0	0	0	0
ENSCAFG00845015039	15	12	7	11
ENSCAFG00845015037	0	0	0	0
ENSCAFG00845015038	0	0	0	0
ENSCAFG00845017699	0	0	0	0
ENSCAFG00845015024	13	3	5	20
ENSCAFG00845017687	9	8	10	10
ENSCAFG00845015025	132	98	146	162
ENSCAFG00845017686	0	0	0	0
ENSCAFG00845015022	0	0	0	0
ENSCAFG00845017685	1432	1469	1370	1233
ENSCAFG00845015023	14	5	17	13
ENSCAFG00845017684	1	2	2	1
ENSCAFG00845015020	10	6	6	6
ENSCAFG00845017683	863	792	709	687
ENSCAFG00845015021	0	0	0	0
ENSCAFG00845017682	0	0	0	0
ENSCAFG00845017681	0	0	0	0
ENSCAFG00845017680	7	10	4	14
ENSCAFG00845027009	112	105	103	95
ENSCAFG00845027006	212	218	214	201
ENSCAFG00845029669	1113	1058	978	1105
ENSCAFG00845027005	0	0	0	0
ENSCAFG00845003059	0	0	0	0
ENSCAFG00845029667	22	18	16	6
ENSCAFG00845003058	0	0	0	0
ENSCAFG00845027007	143	130	145	159
ENSCAFG00845029668	385	479	384	421
ENSCAFG00845003057	730	662	666	719
ENSCAFG00845027002	602	563	532	519
ENSCAFG00845029665	0	0	0	0
ENSCAFG00845003056	0	0	0	0
ENSCAFG00845027001	930	947	921	950
ENSCAFG00845029666	1	7	3	0
ENSCAFG00845003055	0	0	0	0
ENSCAFG00845027004	125	136	132	112

ENSCAFG00845029663	0	0	0	0
ENSCAFG00845003054	0	0	0	0
ENSCAFG00845027003	6960	6959	6490	6353
ENSCAFG00845029664	3	0	0	0
ENSCAFG00845003053	0	0	0	0
ENSCAFG00845029661	511	464	443	432
ENSCAFG00845003052	1949	1746	2128	2064
ENSCAFG00845029662	45	38	72	36
ENSCAFG00845003051	0	0	0	0
ENSCAFG00845027000	252	223	217	278
ENSCAFG00845003050	0	0	0	0
ENSCAFG00845029660	1009	891	941	927
ENSCAFG00845015028	0	0	0	0
ENSCAFG00845015029	0	3	1	0
ENSCAFG00845015026	0	0	0	0
ENSCAFG00845017689	3335	3346	2249	2129
ENSCAFG00845015027	5	2	2	3
ENSCAFG00845017688	3063	2999	2967	3106
ENSCAFG00845015013	0	0	0	0
ENSCAFG00845017676	403	404	354	323
ENSCAFG00845015014	0	0	0	0
ENSCAFG00845017675	0	0	0	0
ENSCAFG00845015011	6683	6449	6444	6541
ENSCAFG00845017674	30	43	37	64
ENSCAFG00845015012	3	3	5	4
ENSCAFG00845017673	0	0	0	0
ENSCAFG00845017672	0	0	0	0
ENSCAFG00845015010	0	0	0	0
ENSCAFG00845017671	690	694	674	738
ENSCAFG00845017670	4	9	11	9
ENSCAFG00845005690	1364	1307	1431	1378
ENSCAFG00845005691	45	64	66	67
ENSCAFG00845005692	352	374	436	444
ENSCAFG00845003029	483	575	414	463
ENSCAFG00845005693	0	0	0	0
ENSCAFG00845003028	1515	1523	1620	1778
ENSCAFG00845005694	18	12	7	26
ENSCAFG00845029658	34	25	42	33
ENSCAFG00845003027	0	0	0	0
ENSCAFG00845005695	9	10	9	7
ENSCAFG00845029659	107	138	109	115

ENSCAFG00845003026	423	379	465	488
ENSCAFG00845005696	0	0	0	0
ENSCAFG00845029656	0	0	0	0
ENSCAFG00845003025	194	208	198	240
ENSCAFG00845005697	65	78	61	49
ENSCAFG00845029657	1862	1809	2013	2005
ENSCAFG00845003024	0	0	0	0
ENSCAFG00845005698	50	31	48	36
ENSCAFG00845029654	49	60	42	59
ENSCAFG00845003023	295	283	224	293
ENSCAFG00845005699	324	227	278	293
ENSCAFG00845029655	6	4	7	13
ENSCAFG00845003022	352	357	362	343
ENSCAFG00845029652	751	704	674	675
ENSCAFG00845003021	240	255	230	232
ENSCAFG00845029653	370	344	406	412
ENSCAFG00845003020	56	62	39	47
ENSCAFG00845029650	0	0	0	0
ENSCAFG00845029651	0	0	0	0
ENSCAFG00845015019	0	0	0	0
ENSCAFG00845015017	1393	1443	1448	1386
ENSCAFG00845015018	0	0	0	0
ENSCAFG00845017679	0	1	0	0
ENSCAFG00845015015	2	0	4	0
ENSCAFG00845017678	2019	2055	1885	1960
ENSCAFG00845015016	0	0	0	0
ENSCAFG00845017677	1	0	0	0
ENSCAFG00845015002	17	13	19	19
ENSCAFG00845017665	0	0	0	0
ENSCAFG00845015003	252	275	298	284
ENSCAFG00845017664	0	0	0	0
ENSCAFG00845015000	306	318	270	285
ENSCAFG00845017663	0	0	0	0
ENSCAFG00845015001	0	0	0	0
ENSCAFG00845017662	0	0	0	0
ENSCAFG00845017661	0	0	0	0
ENSCAFG00845017660	0	0	0	0
ENSCAFG00845005680	106	103	77	60
ENSCAFG00845005681	0	0	0	0
ENSCAFG00845029649	0	0	0	0
ENSCAFG00845005682	9	9	5	10

ENSCAFG00845003039	304	251	211	206
ENSCAFG00845005683	0	0	0	0
ENSCAFG00845029647	0	0	0	0
ENSCAFG00845003038	0	0	0	0
ENSCAFG00845005684	176	163	180	163
ENSCAFG00845029648	4	1	3	0
ENSCAFG00845003037	16	14	16	23
ENSCAFG00845005685	0	0	0	1
ENSCAFG00845029645	102	98	124	132
ENSCAFG00845003036	0	0	0	0
ENSCAFG00845005686	0	0	0	0
ENSCAFG00845029646	0	0	0	0
ENSCAFG00845003035	145	86	117	127
ENSCAFG00845005687	50	53	35	47
ENSCAFG00845029643	262	234	262	312
ENSCAFG00845003034	105	102	132	99
ENSCAFG00845005688	17	27	20	21
ENSCAFG00845029644	0	1	0	0
ENSCAFG00845003033	2	0	0	0
ENSCAFG00845005689	2	1	1	0
ENSCAFG00845029641	234	203	215	238
ENSCAFG00845003032	0	0	0	0
ENSCAFG00845029642	0	0	0	0
ENSCAFG00845003031	0	0	0	0
ENSCAFG00845003030	1	5	4	5
ENSCAFG00845029640	1	1	4	8
ENSCAFG00845015008	0	0	0	0
ENSCAFG00845015009	0	0	0	0
ENSCAFG00845015006	435	447	395	340
ENSCAFG00845017669	751	790	755	687
ENSCAFG00845015007	15	4	4	6
ENSCAFG00845017668	27	28	30	33
ENSCAFG00845015004	0	0	0	0
ENSCAFG00845017667	103	89	111	79
ENSCAFG00845015005	1	5	4	4
ENSCAFG00845017666	0	0	0	0
ENSCAFG00845017654	0	0	0	0
ENSCAFG00845017653	13	19	20	18
ENSCAFG00845017652	0	0	0	0
ENSCAFG00845017651	0	0	0	0
ENSCAFG00845017650	0	0	0	0

ENSCAFG00845003009	521	440	476	514
ENSCAFG00845003008	772	698	683	736
ENSCAFG00845005670	833	786	816	808
ENSCAFG00845029638	442	441	428	426
ENSCAFG00845003007	640	610	511	520
ENSCAFG00845005671	2276	2196	2229	2267
ENSCAFG00845029639	0	2	0	0
ENSCAFG00845003006	87	63	74	67
ENSCAFG00845005672	0	0	0	0
ENSCAFG00845029636	265	229	338	285
ENSCAFG00845003005	3830	3794	4211	4172
ENSCAFG00845005673	0	0	0	0
ENSCAFG00845029637	0	0	0	0
ENSCAFG00845003004	3328	3239	3508	3665
ENSCAFG00845005674	105	67	86	84
ENSCAFG00845029634	665	707	628	696
ENSCAFG00845003003	1	0	0	0
ENSCAFG00845005675	517	463	530	507
ENSCAFG00845029635	0	3	1	1
ENSCAFG00845003002	0	0	0	4
ENSCAFG00845005676	2	2	10	3
ENSCAFG00845029632	440	382	407	372
ENSCAFG00845003001	664	630	665	586
ENSCAFG00845005677	0	0	0	0
ENSCAFG00845029633	0	0	0	1
ENSCAFG00845003000	0	0	0	0
ENSCAFG00845005678	4119	4082	3930	3848
ENSCAFG00845029630	698	541	515	568
ENSCAFG00845005679	31	27	18	35
ENSCAFG00845029631	2	2	2	2
ENSCAFG00845017659	0	0	0	0
ENSCAFG00845017658	0	0	0	0
ENSCAFG00845017657	101	66	76	91
ENSCAFG00845017656	4197	4211	4175	4194
ENSCAFG00845017655	22	22	32	37
ENSCAFG00845017643	0	0	0	0
ENSCAFG00845017642	293	247	302	265
ENSCAFG00845017641	508	468	494	446
ENSCAFG00845017640	847	830	826	817
ENSCAFG00845029629	1913	1954	1970	1969
ENSCAFG00845003019	0	0	0	0

ENSCAFG00845029627	0	0	0	0
ENSCAFG00845003018	66	76	73	69
ENSCAFG00845005660	3123	2892	2947	2780
ENSCAFG00845029628	926	848	916	947
ENSCAFG00845003017	80	67	90	75
ENSCAFG00845005661	590	613	502	593
ENSCAFG00845029625	714	761	780	776
ENSCAFG00845003016	11	13	17	24
ENSCAFG00845005662	561	551	581	489
ENSCAFG00845029626	802	761	695	758
ENSCAFG00845003015	25	47	27	31
ENSCAFG00845005663	0	0	0	0
ENSCAFG00845029623	1614	1481	1754	1761
ENSCAFG00845003014	1	0	1	1
ENSCAFG00845005664	802	720	796	805
ENSCAFG00845029624	0	0	0	0
ENSCAFG00845003013	0	0	0	0
ENSCAFG00845005665	0	0	0	0
ENSCAFG00845029621	18651	18062	16823	17380
ENSCAFG00845003012	159	137	103	138
ENSCAFG00845005666	0	0	0	0
ENSCAFG00845029622	167	142	160	146
ENSCAFG00845003011	0	0	0	0
ENSCAFG00845005667	1	0	2	0
ENSCAFG00845003010	0	0	0	0
ENSCAFG00845005668	100	65	71	71
ENSCAFG00845029620	0	0	0	0
ENSCAFG00845005669	0	0	0	0
ENSCAFG00845017649	0	1	0	0
ENSCAFG00845017648	145	160	114	164
ENSCAFG00845017647	0	0	0	0
ENSCAFG00845017646	11	12	2	13
ENSCAFG00845017645	0	0	0	2
ENSCAFG00845017644	11	20	14	16
ENSCAFG00845030607	1438	1441	1437	1343
ENSCAFG00845030608	0	0	0	0
ENSCAFG00845030609	685	663	721	709
ENSCAFG00845030610	510	490	506	529
ENSCAFG00845030611	0	1	1	0
ENSCAFG00845030612	451	417	397	403
ENSCAFG00845030613	151	128	101	121

ENSCAFG00845030614	2	0	0	1
ENSCAFG00845030615	1	3	1	3
ENSCAFG00845030616	824	775	815	780
ENSCAFG00845030617	1233	1214	1162	1152
ENSCAFG00845030618	2	0	1	0
ENSCAFG00845030619	1	0	0	0
ENSCAFG00845030621	895	812	868	791
ENSCAFG00845030622	0	0	0	0
ENSCAFG00845030623	65	53	63	42
ENSCAFG00845030624	708	610	617	617
ENSCAFG00845030625	762	710	710	807
ENSCAFG00845030626	14	11	16	5
ENSCAFG00845030627	0	0	0	0
ENSCAFG00845030628	5006	4993	5170	4994
ENSCAFG00845030620	19	15	20	25
ENSCAFG00845030629	0	9	2	1
ENSCAFG00845030632	169	149	202	184
ENSCAFG00845030633	0	0	0	1
ENSCAFG00845030634	6	4	6	4
ENSCAFG00845030635	0	0	0	0
ENSCAFG00845027097	65	85	69	88
ENSCAFG00845030636	2493	2490	2291	2283
ENSCAFG00845027096	0	0	0	0
ENSCAFG00845030637	0	0	0	0
ENSCAFG00845027099	0	0	0	0
ENSCAFG00845030638	43	40	34	32
ENSCAFG00845027098	0	0	0	1
ENSCAFG00845030639	5	11	5	1
ENSCAFG00845027093	0	0	0	0
ENSCAFG00845027092	0	1	0	0
ENSCAFG00845027095	825	783	766	794
ENSCAFG00845027094	0	0	0	0
ENSCAFG00845027091	409	367	353	371
ENSCAFG00845030630	0	0	0	0
ENSCAFG00845027090	118	102	113	104
ENSCAFG00845030631	1	0	2	3
ENSCAFG00845030643	402	385	398	399
ENSCAFG00845027089	0	0	0	0
ENSCAFG00845030644	0	2	0	0
ENSCAFG00845030645	0	2	0	2
ENSCAFG00845030646	150	127	144	129

ENSCAFG00845027086	0	0	0	0
ENSCAFG00845030647	3	3	2	1
ENSCAFG00845027085	2466	2304	2454	2547
ENSCAFG00845030648	894	936	942	974
ENSCAFG00845027088	0	0	0	0
ENSCAFG00845030649	240	283	229	230
ENSCAFG00845027087	279	296	285	236
ENSCAFG00845027082	2	7	10	2
ENSCAFG00845027081	0	1	0	0
ENSCAFG00845027084	470	482	501	519
ENSCAFG00845027083	4216	4158	4214	4172
ENSCAFG00845030640	3081	2874	2889	2903
ENSCAFG00845027080	1306	1247	1270	1285
ENSCAFG00845030641	1	1	1	1
ENSCAFG00845030642	1082	1092	1039	1044
ENSCAFG00845015099	0	0	0	0
ENSCAFG00845015097	0	0	0	0
ENSCAFG00845015098	0	0	0	0
ENSCAFG00845015095	643	586	612	648
ENSCAFG00845015096	447	409	435	419
ENSCAFG00845015093	866	820	887	916
ENSCAFG00845015094	0	0	0	0
ENSCAFG00845015091	0	0	0	0
ENSCAFG00845015092	1433	1346	1511	1601
ENSCAFG00845015090	166	132	112	129
ENSCAFG00845027079	238	219	322	359
ENSCAFG00845030654	0	0	0	0
ENSCAFG00845027078	186	191	78	90
ENSCAFG00845030655	0	0	0	0
ENSCAFG00845030656	409	360	365	342
ENSCAFG00845030657	32	39	32	34
ENSCAFG00845027075	11986	11296	12097	12873
ENSCAFG00845030658	280	314	244	277
ENSCAFG00845027074	254	256	286	326
ENSCAFG00845030659	3	1	0	3
ENSCAFG00845027077	110	143	130	113
ENSCAFG00845027076	1677	1667	1671	1745
ENSCAFG00845027071	18	21	26	22
ENSCAFG00845027070	105	82	79	95
ENSCAFG00845027073	748	742	747	715
ENSCAFG00845027072	102	124	73	83

ENSCAFG00845030650	1	1	1	1
ENSCAFG00845030651	55	55	69	58
ENSCAFG00845030652	2802	2574	2710	2608
ENSCAFG00845030653	2	10	9	3
ENSCAFG00845015088	0	0	0	0
ENSCAFG00845015089	116	104	98	95
ENSCAFG00845015086	0	0	0	0
ENSCAFG00845015087	0	0	0	0
ENSCAFG00845015084	0	0	0	0
ENSCAFG00845015085	1268	1182	2087	2003
ENSCAFG00845015082	2262	2223	2555	2772
ENSCAFG00845015083	0	0	0	0
ENSCAFG00845015080	1	0	3	0
ENSCAFG00845015081	0	1	0	0
ENSCAFG00845027068	1131	1163	1162	1125
ENSCAFG00845030665	1411	1316	1240	1342
ENSCAFG00845027067	0	0	0	1
ENSCAFG00845030666	74	67	65	64
ENSCAFG00845030667	1138	1087	1373	1358
ENSCAFG00845027069	2597	2524	2407	2343
ENSCAFG00845030668	2	10	3	6
ENSCAFG00845027064	0	0	1	0
ENSCAFG00845030669	757	732	682	694
ENSCAFG00845027063	123	121	92	94
ENSCAFG00845027066	404	452	435	456
ENSCAFG00845027065	233	215	211	238
ENSCAFG00845027060	0	0	0	0
ENSCAFG00845027061	0	0	0	0
ENSCAFG00845030660	0	0	0	0
ENSCAFG00845030661	0	1	0	2
ENSCAFG00845030662	0	0	0	0
ENSCAFG00845030663	0	0	0	0
ENSCAFG00845030664	0	0	0	0
ENSCAFG00845015079	0	0	0	0
ENSCAFG00845015077	98	134	70	83
ENSCAFG00845015078	0	0	0	0
ENSCAFG00845015075	0	0	0	0
ENSCAFG00845015076	1903	1791	1915	1926
ENSCAFG00845015073	0	0	0	0
ENSCAFG00845015074	1	3	3	2
ENSCAFG00845015071	0	0	0	0

ENSCAFG00845015072	9	21	20	14
ENSCAFG00845015070	2698	2504	2669	2900
ENSCAFG00845027057	1	1	2	0
ENSCAFG00845030676	661	573	534	507
ENSCAFG00845003089	0	0	0	0
ENSCAFG00845027056	1273	1170	1368	1471
ENSCAFG00845030677	0	0	0	0
ENSCAFG00845003088	0	0	0	0
ENSCAFG00845027059	0	0	0	0
ENSCAFG00845030678	75	63	101	61
ENSCAFG00845003087	0	1	0	0
ENSCAFG00845027058	12	5	14	8
ENSCAFG00845030679	10	7	13	10
ENSCAFG00845003086	2981	2960	2456	2590
ENSCAFG00845027053	5211	5049	5256	5480
ENSCAFG00845003085	0	0	0	0
ENSCAFG00845027052	844	761	753	795
ENSCAFG00845003084	38	36	40	61
ENSCAFG00845027055	0	0	1	0
ENSCAFG00845003083	2111	2044	2238	2348
ENSCAFG00845027054	0	0	0	0
ENSCAFG00845003082	2	0	3	7
ENSCAFG00845003081	0	0	0	0
ENSCAFG00845003080	1002	931	812	890
ENSCAFG00845027051	1	0	0	0
ENSCAFG00845030670	1437	1440	1316	1355
ENSCAFG00845027050	0	0	0	0
ENSCAFG00845030671	2	1	1	0
ENSCAFG00845030672	0	0	0	0
ENSCAFG00845030673	33	39	31	43
ENSCAFG00845030674	0	0	0	0
ENSCAFG00845030675	538	571	599	573
ENSCAFG00845015068	0	0	0	0
ENSCAFG00845015069	429	415	369	375
ENSCAFG00845015066	0	0	0	0
ENSCAFG00845015067	19	26	34	34
ENSCAFG00845015064	1762	1795	1524	1581
ENSCAFG00845015065	0	0	0	0
ENSCAFG00845015062	0	0	0	0
ENSCAFG00845015063	2736	2624	2833	2760
ENSCAFG00845015060	0	0	0	0

ENSCAFG00845015061	0	0	0	0
ENSCAFG00845027049	972	890	971	1012
ENSCAFG00845027046	797	734	670	741
ENSCAFG00845027045	4434	4215	4507	4621
ENSCAFG00845030688	0	0	0	0
ENSCAFG00845003099	0	0	0	0
ENSCAFG00845027048	2	0	4	5
ENSCAFG00845003098	4	2	4	1
ENSCAFG00845027047	0	0	0	1
ENSCAFG00845003097	0	0	0	0
ENSCAFG00845027042	0	0	0	0
ENSCAFG00845003096	90	94	73	90
ENSCAFG00845027041	0	0	0	0
ENSCAFG00845003095	1097	1111	1033	1005
ENSCAFG00845027044	620	619	684	574
ENSCAFG00845003094	0	0	0	0
ENSCAFG00845027043	0	0	0	0
ENSCAFG00845003093	108	95	87	80
ENSCAFG00845003092	0	0	0	0
ENSCAFG00845030680	4	13	14	13
ENSCAFG00845003091	0	0	0	0
ENSCAFG00845027040	0	0	0	0
ENSCAFG00845030681	2082	1875	2336	2472
ENSCAFG00845003090	0	0	0	0
ENSCAFG00845030682	1653	1701	1620	1535
ENSCAFG00845030683	1	9	4	8
ENSCAFG00845030684	23	29	40	52
ENSCAFG00845030685	3	3	6	9
ENSCAFG00845030686	0	3	1	0
ENSCAFG00845030600	8606	7943	8149	8227
ENSCAFG00845030601	0	0	0	0
ENSCAFG00845030602	0	0	0	0
ENSCAFG00845030603	2	8	5	1
ENSCAFG00845030604	1934	1910	1910	1936
ENSCAFG00845030605	218	193	210	230
ENSCAFG00845030606	863	858	778	714
ENSCAFG00845017632	93	79	82	96
ENSCAFG00845017631	979	946	880	931
ENSCAFG00845017630	0	0	0	0
ENSCAFG00845029618	0	0	0	0
ENSCAFG00845029619	0	0	0	0

ENSCAFG00845029616	0	0	0	0
ENSCAFG00845029617	7672	7604	7596	7785
ENSCAFG00845005650	0	0	0	0
ENSCAFG00845029614	0	0	0	0
ENSCAFG00845005651	41	54	37	41
ENSCAFG00845029615	382	352	414	415
ENSCAFG00845005652	0	0	0	0
ENSCAFG00845029612	0	0	0	0
ENSCAFG00845005653	126	96	67	100
ENSCAFG00845029613	1712	1695	1599	1616
ENSCAFG00845005654	3218	2943	3268	3441
ENSCAFG00845029610	332	348	314	363
ENSCAFG00845005655	0	0	0	0
ENSCAFG00845029611	816	813	948	841
ENSCAFG00845005656	3	0	0	0
ENSCAFG00845005657	1300	1247	1238	1275
ENSCAFG00845005658	0	0	0	0
ENSCAFG00845005659	575	635	574	673
ENSCAFG00845017639	2022	1990	2207	2207
ENSCAFG00845017638	520	481	525	584
ENSCAFG00845017637	0	0	0	0
ENSCAFG00845017636	243	185	115	112
ENSCAFG00845017635	261	282	220	286
ENSCAFG00845017634	379	341	337	398
ENSCAFG00845017633	0	0	0	0
ENSCAFG00845017621	0	0	0	0
ENSCAFG00845017620	0	0	0	0
ENSCAFG00845029609	1	1	7	2
ENSCAFG00845029607	1025	917	966	958
ENSCAFG00845029608	0	0	0	2
ENSCAFG00845029605	17	1	4	2
ENSCAFG00845029606	283	267	302	344
ENSCAFG00845029603	354	281	351	344
ENSCAFG00845005640	588	459	509	569
ENSCAFG00845029604	0	0	0	0
ENSCAFG00845005641	0	2	1	3
ENSCAFG00845029601	101	89	121	103
ENSCAFG00845005642	114	120	112	116
ENSCAFG00845029602	3	7	0	3
ENSCAFG00845005643	0	0	0	0
ENSCAFG00845005644	138	118	110	120

ENSCAFG00845029600	1	0	0	0
ENSCAFG00845005645	89	115	109	105
ENSCAFG00845005646	36	20	16	25
ENSCAFG00845005647	11903	11492	11366	11692
ENSCAFG00845005648	971	955	955	870
ENSCAFG00845005649	0	0	0	0
ENSCAFG00845017629	0	0	0	0
ENSCAFG00845017628	57	45	39	59
ENSCAFG00845017627	0	0	0	0
ENSCAFG00845017626	0	0	0	0
ENSCAFG00845017625	0	0	0	0
ENSCAFG00845017624	0	0	0	0
ENSCAFG00845017623	0	0	0	0
ENSCAFG00845017622	0	0	0	0
ENSCAFG00845017610	0	0	0	0
ENSCAFG00845005630	3609	3479	3120	2990
ENSCAFG00845005631	800	775	674	706
ENSCAFG00845005632	214	185	160	146
ENSCAFG00845005633	0	0	0	0
ENSCAFG00845005634	425	453	425	429
ENSCAFG00845005635	0	0	0	0
ENSCAFG00845005636	1	5	1	3
ENSCAFG00845005637	61	82	75	59
ENSCAFG00845005638	0	0	0	1
ENSCAFG00845005639	13	10	18	14
ENSCAFG00845017619	0	0	0	0
ENSCAFG00845017618	1	5	1	3
ENSCAFG00845017617	0	0	0	0
ENSCAFG00845017616	0	0	0	0
ENSCAFG00845017615	0	0	1	0
ENSCAFG00845017614	0	0	0	0
ENSCAFG00845017613	0	0	0	0
ENSCAFG00845017612	0	0	0	0
ENSCAFG00845017611	2	0	8	1
ENSCAFG00845005620	668	616	699	632
ENSCAFG00845005621	1	1	0	0
ENSCAFG00845005622	1	1	2	5
ENSCAFG00845005623	26	38	36	23
ENSCAFG00845005624	5	2	3	0
ENSCAFG00845005625	19	16	17	12
ENSCAFG00845005626	27	22	31	19

ENSCAFG00845005627	370	304	311	350
ENSCAFG00845017609	6716	6612	6322	6383
ENSCAFG00845005628	0	0	0	0
ENSCAFG00845017608	3	2	2	0
ENSCAFG00845005629	1	0	0	0
ENSCAFG00845017607	0	2	0	0
ENSCAFG00845017606	5208	5052	5173	5301
ENSCAFG00845017605	563	522	561	601
ENSCAFG00845017604	2	1	0	0
ENSCAFG00845017603	0	0	0	0
ENSCAFG00845017602	133	124	114	153
ENSCAFG00845017601	12	4	8	2
ENSCAFG00845017600	0	0	0	0
ENSCAFG00845005610	848	799	805	921
ENSCAFG00845005611	0	1	0	1
ENSCAFG00845005612	0	0	0	0
ENSCAFG00845005613	39	32	37	32
ENSCAFG00845005614	1441	1338	1289	1348
ENSCAFG00845005615	0	0	0	0
ENSCAFG00845005616	1663	1595	1602	1677
ENSCAFG00845005617	251	257	243	236
ENSCAFG00845005618	0	0	0	0
ENSCAFG00845005619	32	37	47	29
ENSCAFG00845005600	606	629	469	494
ENSCAFG00845005601	0	0	0	0
ENSCAFG00845005602	0	1	0	0
ENSCAFG00845005603	1	0	0	0
ENSCAFG00845005604	0	0	0	0
ENSCAFG00845005605	0	0	0	0
ENSCAFG00845005606	0	0	1	0
ENSCAFG00845005607	1012	897	1009	949
ENSCAFG00845005608	2	0	1	2
ENSCAFG00845005609	0	0	0	0
ENSCAFG00845019536	0	0	0	0
ENSCAFG00845020525	1501	1502	1463	1453
ENSCAFG00845019537	0	0	0	0
ENSCAFG00845020526	0	0	0	0
ENSCAFG00845019538	1002	1047	1141	1179
ENSCAFG00845020523	0	0	0	0
ENSCAFG00845019539	0	0	0	0
ENSCAFG00845020524	0	0	0	0

ENSCAFG00845020529	0	1	2	6
ENSCAFG00845020527	522	457	499	490
ENSCAFG00845020528	0	2	1	0
ENSCAFG00845007550	1502	1398	1429	1512
ENSCAFG00845007552	0	1	0	0
ENSCAFG00845007551	1498	1435	1394	1424
ENSCAFG00845007554	0	0	0	0
ENSCAFG00845020521	0	0	0	0
ENSCAFG00845007553	0	0	0	0
ENSCAFG00845020522	577	567	662	683
ENSCAFG00845007556	5	1	1	4
ENSCAFG00845007555	0	0	0	0
ENSCAFG00845020520	273	281	278	260
ENSCAFG00845007558	4	2	7	1
ENSCAFG00845007557	59	77	69	85
ENSCAFG00845007559	0	0	0	0
ENSCAFG00845019530	0	0	0	0
ENSCAFG00845019531	0	0	0	0
ENSCAFG00845019532	1	1	0	2
ENSCAFG00845019533	1	1	4	4
ENSCAFG00845019534	695	621	640	680
ENSCAFG00845019535	0	0	0	0
ENSCAFG00845019525	11	4	7	10
ENSCAFG00845020514	12667	12024	12986	13288
ENSCAFG00845019526	267	273	254	214
ENSCAFG00845020515	0	0	2	0
ENSCAFG00845019527	0	0	0	0
ENSCAFG00845020512	0	0	0	0
ENSCAFG00845019528	78	32	34	55
ENSCAFG00845020513	71	74	73	78
ENSCAFG00845019529	1267	1152	1039	938
ENSCAFG00845020519	1142	1069	914	947
ENSCAFG00845020516	0	4	0	0
ENSCAFG00845020517	0	0	0	0
ENSCAFG00845007541	0	0	0	0
ENSCAFG00845007540	0	0	0	0
ENSCAFG00845007543	0	0	0	0
ENSCAFG00845020510	36	32	28	39
ENSCAFG00845007542	481	492	482	510
ENSCAFG00845020511	2687	2448	2745	2627
ENSCAFG00845007545	0	2	0	0

ENSCAFG00845007544	6	5	1	6
ENSCAFG00845007547	0	0	0	0
ENSCAFG00845007546	0	0	0	0
ENSCAFG00845007549	1	1	1	0
ENSCAFG00845007548	5	8	8	8
ENSCAFG00845020509	0	0	0	0
ENSCAFG00845019520	27	63	32	38
ENSCAFG00845019521	0	1	0	0
ENSCAFG00845019522	0	0	0	0
ENSCAFG00845019523	0	0	0	0
ENSCAFG00845019524	0	0	0	0
ENSCAFG00845019514	18	18	18	26
ENSCAFG00845019515	7	6	13	7
ENSCAFG00845020504	0	0	0	0
ENSCAFG00845019516	242	256	247	205
ENSCAFG00845020501	0	0	0	0
ENSCAFG00845019517	0	0	0	0
ENSCAFG00845020502	3558	3417	3223	3292
ENSCAFG00845019518	176	221	188	233
ENSCAFG00845020507	2	7	20	4
ENSCAFG00845019519	26	15	18	11
ENSCAFG00845020508	0	0	0	0
ENSCAFG00845020505	1	1	0	0
ENSCAFG00845020506	73	81	94	123
ENSCAFG00845007530	1899	1866	1914	1946
ENSCAFG00845007532	4	1	6	8
ENSCAFG00845007531	2	0	2	0
ENSCAFG00845020500	7	3	13	6
ENSCAFG00845007534	0	0	0	0
ENSCAFG00845007533	0	0	0	0
ENSCAFG00845007536	0	0	0	0
ENSCAFG00845007535	31	33	44	30
ENSCAFG00845007538	7	5	4	2
ENSCAFG00845007537	0	0	0	0
ENSCAFG00845007539	0	0	0	0
ENSCAFG00845019510	597	599	611	588
ENSCAFG00845019511	0	0	0	0
ENSCAFG00845019512	10	10	0	2
ENSCAFG00845019513	125	145	130	134
ENSCAFG00845019503	397	393	452	481
ENSCAFG00845019504	14	15	26	25

ENSCAFG00845019505	0	0	0	0
ENSCAFG00845019506	6	0	0	2
ENSCAFG00845019507	0	0	0	0
ENSCAFG00845019508	443	442	522	501
ENSCAFG00845019509	5959	5569	5393	5380
ENSCAFG00845007521	15	10	8	21
ENSCAFG00845007520	0	0	0	0
ENSCAFG00845007523	2	1	0	3
ENSCAFG00845007522	0	0	0	0
ENSCAFG00845007525	0	0	0	0
ENSCAFG00845007524	12773	12434	7621	7343
ENSCAFG00845007527	0	0	0	0
ENSCAFG00845007526	0	0	0	0
ENSCAFG00845007529	146	164	278	243
ENSCAFG00845007528	25	27	10	23
ENSCAFG00845019500	328	353	424	363
ENSCAFG00845019501	0	0	0	0
ENSCAFG00845019502	3198	3174	2709	2826
ENSCAFG00845007510	1360	1382	1203	1238
ENSCAFG00845007512	803	804	701	737
ENSCAFG00845007511	0	0	0	0
ENSCAFG00845007514	0	0	0	0
ENSCAFG00845007513	0	0	0	0
ENSCAFG00845007516	0	0	0	0
ENSCAFG00845007515	3	7	3	6
ENSCAFG00845007518	2	0	1	2
ENSCAFG00845007517	0	0	0	0
ENSCAFG00845007519	0	0	0	0
ENSCAFG00845007501	12	25	44	26
ENSCAFG00845007500	371	333	350	393
ENSCAFG00845007503	0	0	0	0
ENSCAFG00845007502	0	0	1	0
ENSCAFG00845007505	1668	1512	1526	1590
ENSCAFG00845007504	0	0	0	0
ENSCAFG00845007507	5	4	0	1
ENSCAFG00845007506	0	0	0	1
ENSCAFG00845007509	0	0	0	0
ENSCAFG00845007508	23	19	26	21
ENSCAFG00845020594	0	0	0	0
ENSCAFG00845020595	2536	2291	2344	2378
ENSCAFG00845020592	0	0	0	0

ENSCAFG00845020593	0	0	0	0
ENSCAFG00845020598	0	0	0	0
ENSCAFG00845020599	450	462	463	487
ENSCAFG00845020596	1376	1199	1356	1376
ENSCAFG00845020597	0	0	0	0
ENSCAFG00845020590	1216	1128	1062	1092
ENSCAFG00845020591	0	0	0	0
ENSCAFG00845020589	139	144	145	153
ENSCAFG00845020583	0	1	0	3
ENSCAFG00845020584	1	3	0	0
ENSCAFG00845020581	0	0	0	0
ENSCAFG00845020582	0	0	0	0
ENSCAFG00845020587	451	423	362	378
ENSCAFG00845020588	0	0	0	0
ENSCAFG00845020585	183	145	198	208
ENSCAFG00845020586	0	0	0	0
ENSCAFG00845019590	55	55	41	37
ENSCAFG00845019591	0	0	0	0
ENSCAFG00845020580	427	402	388	440
ENSCAFG00845019592	182	176	493	440
ENSCAFG00845019593	433	470	389	367
ENSCAFG00845019594	0	0	0	3
ENSCAFG00845019595	0	0	0	0
ENSCAFG00845019596	0	0	0	0
ENSCAFG00845019597	145	143	121	101
ENSCAFG00845019598	377	349	369	351
ENSCAFG00845019599	0	0	0	0
ENSCAFG00845020578	661	596	535	550
ENSCAFG00845020579	4	6	5	2
ENSCAFG00845020572	0	0	0	0
ENSCAFG00845020573	1511	1523	1408	1387
ENSCAFG00845020570	0	2	3	1
ENSCAFG00845020571	1059	975	1039	1187
ENSCAFG00845020576	789	734	815	741
ENSCAFG00845020577	0	0	0	0
ENSCAFG00845020574	6	0	1	6
ENSCAFG00845020575	42	36	37	45
ENSCAFG00845019580	99	121	115	99
ENSCAFG00845019581	0	0	0	0
ENSCAFG00845019582	0	1	0	0
ENSCAFG00845019583	0	0	0	0

ENSCAFG00845019584	0	0	0	0
ENSCAFG00845019585	0	0	0	0
ENSCAFG00845019586	0	0	0	0
ENSCAFG00845019587	33	33	22	42
ENSCAFG00845019588	1	1	0	2
ENSCAFG00845019589	0	1	0	0
ENSCAFG00845020569	235	257	275	252
ENSCAFG00845020567	46	42	53	51
ENSCAFG00845020568	23	26	15	12
ENSCAFG00845007590	0	4	3	4
ENSCAFG00845007592	0	0	0	0
ENSCAFG00845007591	730	723	640	674
ENSCAFG00845007594	1011	909	962	967
ENSCAFG00845020561	0	0	0	0
ENSCAFG00845007593	758	794	861	827
ENSCAFG00845020562	803	769	771	754
ENSCAFG00845007596	2	6	4	3
ENSCAFG00845007595	0	0	0	0
ENSCAFG00845020560	24	25	31	24
ENSCAFG00845007598	0	0	0	0
ENSCAFG00845020565	25	27	29	40
ENSCAFG00845007597	0	0	0	0
ENSCAFG00845020566	0	0	0	0
ENSCAFG00845020563	0	0	0	0
ENSCAFG00845007599	3428	3145	3310	3300
ENSCAFG00845019570	0	0	0	0
ENSCAFG00845019571	1441	1566	1391	1482
ENSCAFG00845019572	4	4	2	1
ENSCAFG00845019573	406	339	349	339
ENSCAFG00845019574	277	254	260	284
ENSCAFG00845019575	26	32	40	37
ENSCAFG00845019576	403	416	468	445
ENSCAFG00845019577	0	0	0	0
ENSCAFG00845019578	0	0	0	0
ENSCAFG00845019579	0	0	0	0
ENSCAFG00845019569	0	0	0	0
ENSCAFG00845020558	734	782	562	615
ENSCAFG00845020559	0	0	0	0
ENSCAFG00845020556	598	556	645	620
ENSCAFG00845020557	0	0	0	0
ENSCAFG00845007581	692	706	634	709

ENSCAFG00845007580	0	0	0	0
ENSCAFG00845007583	0	0	0	0
ENSCAFG00845020550	0	0	0	0
ENSCAFG00845007582	0	0	0	0
ENSCAFG00845020551	497	445	430	418
ENSCAFG00845007585	0	0	0	0
ENSCAFG00845007584	0	1	0	0
ENSCAFG00845007587	8	5	5	3
ENSCAFG00845020554	0	0	0	0
ENSCAFG00845007586	0	0	0	0
ENSCAFG00845020555	0	0	0	0
ENSCAFG00845007589	56	51	62	69
ENSCAFG00845020552	511	515	423	400
ENSCAFG00845007588	577	594	534	595
ENSCAFG00845020553	0	0	0	0
ENSCAFG00845019560	0	0	0	0
ENSCAFG00845019561	0	0	1	1
ENSCAFG00845019562	0	0	0	0
ENSCAFG00845019563	409	352	371	400
ENSCAFG00845019564	17	12	11	14
ENSCAFG00845019565	774	708	684	832
ENSCAFG00845019566	1376	1336	1278	1268
ENSCAFG00845019567	990	924	924	994
ENSCAFG00845019568	0	0	0	0
ENSCAFG00845019558	0	0	0	0
ENSCAFG00845020547	17	8	15	7
ENSCAFG00845020548	0	0	0	0
ENSCAFG00845020545	373	305	325	368
ENSCAFG00845020546	6	6	2	4
ENSCAFG00845007570	0	0	0	0
ENSCAFG00845020549	185	198	167	199
ENSCAFG00845007572	120	146	110	116
ENSCAFG00845007571	4	1	2	1
ENSCAFG00845020540	0	3	0	1
ENSCAFG00845007574	379	389	357	376
ENSCAFG00845007573	277	261	339	313
ENSCAFG00845007576	377	333	398	402
ENSCAFG00845020543	0	1	0	1
ENSCAFG00845007575	9316	9001	9868	9777
ENSCAFG00845020544	990	939	1026	1028
ENSCAFG00845007578	1	1	0	0

ENSCAFG00845020541	1139	1118	1150	1252
ENSCAFG00845007577	532	566	522	463
ENSCAFG00845020542	1555	1443	1608	1701
ENSCAFG00845007579	0	0	0	0
ENSCAFG00845019550	718	675	673	665
ENSCAFG00845019551	460	411	433	438
ENSCAFG00845019552	0	0	0	0
ENSCAFG00845019553	754	790	766	705
ENSCAFG00845019554	8	1	2	2
ENSCAFG00845019555	0	0	0	0
ENSCAFG00845019556	3489	3479	3301	3294
ENSCAFG00845019557	99	92	80	84
ENSCAFG00845019547	0	0	0	0
ENSCAFG00845020536	2874	2826	2593	2760
ENSCAFG00845019548	0	0	0	0
ENSCAFG00845020537	371	379	401	370
ENSCAFG00845019549	1597	1498	1529	1601
ENSCAFG00845020534	0	0	0	0
ENSCAFG00845020535	1	4	0	0
ENSCAFG00845020538	5	3	4	4
ENSCAFG00845020539	3	4	8	6
ENSCAFG00845007561	0	0	0	0
ENSCAFG00845007560	0	0	0	0
ENSCAFG00845007563	0	0	0	0
ENSCAFG00845007562	0	0	0	0
ENSCAFG00845007565	0	0	0	2
ENSCAFG00845020532	0	0	0	0
ENSCAFG00845007564	0	0	3	1
ENSCAFG00845020533	0	0	0	0
ENSCAFG00845007567	0	0	1	0
ENSCAFG00845020530	751	773	789	798
ENSCAFG00845007566	0	0	0	0
ENSCAFG00845007569	0	0	0	0
ENSCAFG00845007568	0	0	0	0
ENSCAFG00845019540	0	0	0	0
ENSCAFG00845019541	526	552	524	508
ENSCAFG00845019542	0	0	0	0
ENSCAFG00845019543	1177	1205	1160	1333
ENSCAFG00845019544	1107	1023	1240	1321
ENSCAFG00845019545	0	0	0	0
ENSCAFG00845019546	15	14	8	3

ENSCAFG00845020495	49	57	57	44
ENSCAFG00845020496	0	0	0	0
ENSCAFG00845020493	61	66	68	61
ENSCAFG00845020494	0	0	0	0
ENSCAFG00845020499	0	1	0	1
ENSCAFG00845020497	513	458	553	580
ENSCAFG00845020498	409	379	338	348
ENSCAFG00845020491	1052	969	1025	957
ENSCAFG00845020492	0	0	0	0
ENSCAFG00845020490	0	0	0	0
ENSCAFG00845019657	69	65	37	47
ENSCAFG00845020646	0	0	0	0
ENSCAFG00845019658	1	1	0	0
ENSCAFG00845020647	872	806	808	863
ENSCAFG00845019659	568	510	478	472
ENSCAFG00845020644	4	0	0	0
ENSCAFG00845020645	182	241	154	158
ENSCAFG00845020648	0	0	0	0
ENSCAFG00845020649	2719	2615	2821	2766
ENSCAFG00845007671	109	106	127	122
ENSCAFG00845007670	0	0	0	0
ENSCAFG00845005010	10685	10272	10399	10168
ENSCAFG00845007673	516	588	569	541
ENSCAFG00845005011	0	0	0	0
ENSCAFG00845007672	828	755	614	649
ENSCAFG00845005012	2	4	0	0
ENSCAFG00845007675	423	439	307	289
ENSCAFG00845020642	0	0	0	0
ENSCAFG00845005013	19	25	20	27
ENSCAFG00845007674	1	5	11	17
ENSCAFG00845020643	0	0	0	0
ENSCAFG00845005014	0	0	0	0
ENSCAFG00845007677	0	0	0	0
ENSCAFG00845020640	1	5	3	0
ENSCAFG00845005015	1582	1538	1187	1233
ENSCAFG00845007676	0	0	0	0
ENSCAFG00845020641	0	0	0	0
ENSCAFG00845005016	1638	1568	1600	1607
ENSCAFG00845007679	0	3	10	3
ENSCAFG00845005017	0	0	0	0
ENSCAFG00845007678	3745	3602	3425	3573

ENSCAFG00845005018	0	0	0	0
ENSCAFG00845005019	26	9	18	14
ENSCAFG00845019650	0	0	1	0
ENSCAFG00845019651	293	271	237	262
ENSCAFG00845019652	506	515	455	491
ENSCAFG00845019653	0	2	0	0
ENSCAFG00845019654	2	3	4	3
ENSCAFG00845019655	0	0	0	0
ENSCAFG00845019656	28	36	20	10
ENSCAFG00845019646	7	1	1	0
ENSCAFG00845020635	2212	2091	2152	2271
ENSCAFG00845019647	0	0	0	0
ENSCAFG00845020636	0	0	0	0
ENSCAFG00845019648	185	196	174	210
ENSCAFG00845020633	641	512	562	625
ENSCAFG00845019649	0	0	0	0
ENSCAFG00845020634	2	1	0	8
ENSCAFG00845020639	0	0	0	0
ENSCAFG00845020637	1080	963	1227	1255
ENSCAFG00845020638	507	627	470	482
ENSCAFG00845007660	341	323	384	340
ENSCAFG00845007662	260	262	133	135
ENSCAFG00845005000	5	8	7	4
ENSCAFG00845007661	0	0	0	0
ENSCAFG00845005001	0	1	3	3
ENSCAFG00845007664	0	0	0	0
ENSCAFG00845020631	1409	1394	1372	1447
ENSCAFG00845005002	444	439	401	373
ENSCAFG00845007663	0	0	0	0
ENSCAFG00845020632	31	27	27	47
ENSCAFG00845005003	4	7	9	3
ENSCAFG00845007666	0	0	2	0
ENSCAFG00845005004	353	296	339	357
ENSCAFG00845007665	329	267	289	251
ENSCAFG00845020630	22	37	31	25
ENSCAFG00845005005	1729	1598	1641	1590
ENSCAFG00845007668	884	705	763	768
ENSCAFG00845005006	889	916	856	897
ENSCAFG00845007667	1683	1698	1679	1905
ENSCAFG00845005007	2	2	0	1
ENSCAFG00845005008	462	424	478	430

ENSCAFG00845007669	305	303	351	368
ENSCAFG00845005009	1	3	3	1
ENSCAFG00845019640	3	0	0	1
ENSCAFG00845019641	0	0	0	0
ENSCAFG00845019642	0	0	0	0
ENSCAFG00845019643	719	657	598	588
ENSCAFG00845019644	474	447	460	440
ENSCAFG00845019645	0	0	0	0
ENSCAFG00845019635	0	0	0	0
ENSCAFG00845020624	1484	1349	1695	1613
ENSCAFG00845019636	280	288	222	247
ENSCAFG00845020625	1	0	0	4
ENSCAFG00845019637	4	9	5	9
ENSCAFG00845020622	0	0	0	0
ENSCAFG00845019638	0	0	0	0
ENSCAFG00845020623	2	2	1	3
ENSCAFG00845019639	553	617	564	576
ENSCAFG00845020628	14	7	13	15
ENSCAFG00845020629	0	0	0	0
ENSCAFG00845020626	0	0	0	0
ENSCAFG00845020627	137	122	121	145
ENSCAFG00845007651	5	7	5	3
ENSCAFG00845007650	0	0	0	0
ENSCAFG00845007653	17	9	16	16
ENSCAFG00845020620	8	0	7	6
ENSCAFG00845007652	2666	2678	2463	2386
ENSCAFG00845020621	386	372	390	434
ENSCAFG00845007655	0	0	0	0
ENSCAFG00845007654	0	4	0	5
ENSCAFG00845007657	0	0	0	0
ENSCAFG00845007656	1	2	0	3
ENSCAFG00845007659	3269	3197	2973	3209
ENSCAFG00845007658	554	517	436	452
ENSCAFG00845020619	755	731	740	767
ENSCAFG00845019630	3	2	3	3
ENSCAFG00845019631	0	0	0	0
ENSCAFG00845019632	1	1	2	6
ENSCAFG00845019633	0	0	0	0
ENSCAFG00845019634	548	541	517	506
ENSCAFG00845019624	1095	933	1085	1019
ENSCAFG00845020613	966	1018	946	1000

ENSCAFG00845019625	5	3	1	0
ENSCAFG00845020614	56	58	48	45
ENSCAFG00845019626	0	0	0	0
ENSCAFG00845020611	202	193	177	183
ENSCAFG00845019627	2	3	2	1
ENSCAFG00845020612	1	0	0	0
ENSCAFG00845019628	1	0	4	0
ENSCAFG00845020617	629	575	542	538
ENSCAFG00845019629	0	0	0	0
ENSCAFG00845020618	72	66	55	57
ENSCAFG00845020615	0	0	0	0
ENSCAFG00845020616	0	0	0	1
ENSCAFG00845007640	0	0	0	0
ENSCAFG00845007642	179	166	169	188
ENSCAFG00845007641	0	0	0	0
ENSCAFG00845020610	114	76	80	96
ENSCAFG00845007644	7264	6786	7091	7296
ENSCAFG00845007643	551	520	487	432
ENSCAFG00845007646	0	0	0	1
ENSCAFG00845030005	0	0	0	0
ENSCAFG00845007645	0	0	0	0
ENSCAFG00845030006	51	35	40	29
ENSCAFG00845007648	1080	1066	1078	1079
ENSCAFG00845030007	0	0	0	0
ENSCAFG00845007647	0	0	0	0
ENSCAFG00845030008	1	0	0	0
ENSCAFG00845030009	0	0	0	0
ENSCAFG00845007649	0	0	0	0
ENSCAFG00845020608	1426	1474	1372	1347
ENSCAFG00845020609	0	0	2	0
ENSCAFG00845030000	0	0	0	0
ENSCAFG00845019620	0	0	0	0
ENSCAFG00845030001	0	0	0	0
ENSCAFG00845019621	0	0	0	0
ENSCAFG00845030002	0	0	0	0
ENSCAFG00845019622	1	2	4	7
ENSCAFG00845030003	593	554	549	523
ENSCAFG00845019623	21	14	9	10
ENSCAFG00845030004	6	2	4	7
ENSCAFG00845019613	2807	2955	3302	3267
ENSCAFG00845020602	3	1	1	0

ENSCAFG00845019614	0	0	0	0
ENSCAFG00845020603	298	314	290	278
ENSCAFG00845019615	1	0	0	1
ENSCAFG00845020600	410	418	423	494
ENSCAFG00845019616	0	0	0	0
ENSCAFG00845020601	0	0	0	0
ENSCAFG00845019617	305	255	156	191
ENSCAFG00845020606	0	0	0	0
ENSCAFG00845019618	0	0	0	0
ENSCAFG00845020607	0	0	0	0
ENSCAFG00845019619	2058	1925	2102	2157
ENSCAFG00845020604	1	0	0	0
ENSCAFG00845020605	122	139	107	114
ENSCAFG00845007631	38	44	55	43
ENSCAFG00845007630	0	0	0	0
ENSCAFG00845007633	9	8	17	3
ENSCAFG00845007632	839	846	983	985
ENSCAFG00845007635	661	716	694	760
ENSCAFG00845030016	435	405	431	458
ENSCAFG00845007634	0	0	0	0
ENSCAFG00845030017	0	0	0	0
ENSCAFG00845007637	1	0	0	1
ENSCAFG00845030018	347	346	330	292
ENSCAFG00845007636	203	188	191	203
ENSCAFG00845030019	7360	6985	6794	6599
ENSCAFG00845007639	7	2	10	5
ENSCAFG00845007638	581	660	593	660
ENSCAFG00845030010	3	1	0	1
ENSCAFG00845030011	0	0	0	0
ENSCAFG00845030012	0	3	0	0
ENSCAFG00845019610	30	49	38	37
ENSCAFG00845030013	3	2	4	4
ENSCAFG00845019611	0	0	2	0
ENSCAFG00845030014	11	11	5	11
ENSCAFG00845019612	0	0	0	0
ENSCAFG00845030015	0	0	0	0
ENSCAFG00845019602	508	535	468	477
ENSCAFG00845019603	3261	3142	3081	3100
ENSCAFG00845019604	217	240	184	233
ENSCAFG00845019605	0	0	0	0
ENSCAFG00845019606	266	277	240	238

ENSCAFG00845019607	0	0	0	0
ENSCAFG00845019608	0	0	0	0
ENSCAFG00845019609	149	119	114	105
ENSCAFG00845007620	112	93	112	102
ENSCAFG00845007622	256	275	264	277
ENSCAFG00845007621	0	3	0	2
ENSCAFG00845007624	0	0	0	0
ENSCAFG00845030027	1041	925	869	938
ENSCAFG00845007623	620	585	511	569
ENSCAFG00845030028	0	0	0	0
ENSCAFG00845007626	18	19	10	21
ENSCAFG00845030029	0	0	0	0
ENSCAFG00845007625	84	95	93	82
ENSCAFG00845007628	202	193	206	237
ENSCAFG00845007627	100	136	129	101
ENSCAFG00845007629	2503	2427	2484	2479
ENSCAFG00845030020	819	766	888	938
ENSCAFG00845030021	0	0	0	0
ENSCAFG00845030022	5	0	2	2
ENSCAFG00845030023	315	297	259	288
ENSCAFG00845030024	1109	1042	1041	1132
ENSCAFG00845019600	45	46	50	62
ENSCAFG00845030025	114	131	134	158
ENSCAFG00845019601	2681	2779	2613	2635
ENSCAFG00845030026	509	465	429	416
ENSCAFG00845007611	4	3	3	0
ENSCAFG00845007610	8	7	6	6
ENSCAFG00845007613	0	0	0	0
ENSCAFG00845030038	889	812	1018	1012
ENSCAFG00845007612	7	12	5	3
ENSCAFG00845030039	343	338	342	361
ENSCAFG00845007615	2	3	0	1
ENSCAFG00845007614	0	0	0	2
ENSCAFG00845007617	404	383	431	439
ENSCAFG00845007616	0	0	0	0
ENSCAFG00845007619	0	0	0	0
ENSCAFG00845007618	0	0	0	0
ENSCAFG00845030030	1261	1301	1440	1453
ENSCAFG00845030031	2222	2196	2020	2106
ENSCAFG00845030032	5	2	2	3
ENSCAFG00845030033	7	6	2	7

ENSCAFG00845030034	0	0	0	0
ENSCAFG00845030035	5730	5560	5426	5719
ENSCAFG00845030036	0	0	0	0
ENSCAFG00845030037	470	426	357	431
ENSCAFG00845030040	51	48	52	73
ENSCAFG00845007600	0	0	0	0
ENSCAFG00845007602	0	0	0	0
ENSCAFG00845030049	233	245	180	247
ENSCAFG00845007601	1	0	0	0
ENSCAFG00845007604	207	227	196	195
ENSCAFG00845007603	0	0	0	0
ENSCAFG00845007606	0	0	0	0
ENSCAFG00845007605	0	0	0	0
ENSCAFG00845007608	24	21	12	17
ENSCAFG00845007607	0	0	0	0
ENSCAFG00845030041	0	0	0	0
ENSCAFG00845007609	17	22	13	12
ENSCAFG00845030042	0	0	0	0
ENSCAFG00845030043	354	298	362	418
ENSCAFG00845030044	0	0	0	0
ENSCAFG00845030045	471	466	449	476
ENSCAFG00845030046	0	0	0	1
ENSCAFG00845030047	792	751	750	827
ENSCAFG00845030048	167	182	169	150
ENSCAFG00845005090	0	0	0	0
ENSCAFG00845005091	6	3	8	5
ENSCAFG00845017079	649	691	695	687
ENSCAFG00845005092	3	1	0	5
ENSCAFG00845017078	0	0	0	1
ENSCAFG00845005093	1719	1563	1697	1681
ENSCAFG00845017077	0	1	1	3
ENSCAFG00845005094	749	721	734	758
ENSCAFG00845017076	1584	1611	1511	1595
ENSCAFG00845005095	0	0	0	0
ENSCAFG00845017075	0	3	0	5
ENSCAFG00845005096	0	0	0	0
ENSCAFG00845017074	0	0	0	0
ENSCAFG00845005097	0	0	0	0
ENSCAFG00845017073	937	895	958	1044
ENSCAFG00845005098	102	82	109	103
ENSCAFG00845017072	6	12	17	29

ENSCAFG00845005099	0	0	0	0
ENSCAFG00845017071	0	1	0	0
ENSCAFG00845017070	87	95	95	108
ENSCAFG00845029058	0	0	0	0
ENSCAFG00845029059	1501	1453	1326	1401
ENSCAFG00845029056	0	0	0	0
ENSCAFG00845029057	8	2	4	2
ENSCAFG00845029054	0	0	0	0
ENSCAFG00845029055	21	26	30	34
ENSCAFG00845029052	192	182	179	188
ENSCAFG00845029053	0	0	0	0
ENSCAFG00845029050	0	0	0	0
ENSCAFG00845029051	0	0	0	0
ENSCAFG00845017069	1	3	0	1
ENSCAFG00845005080	0	3	1	1
ENSCAFG00845017068	737	775	671	752
ENSCAFG00845005081	3599	3449	3233	3396
ENSCAFG00845017067	0	0	0	0
ENSCAFG00845005082	44	58	36	48
ENSCAFG00845017066	0	0	0	0
ENSCAFG00845005083	15	6	5	13
ENSCAFG00845017065	1155	1178	1145	1147
ENSCAFG00845005084	1561	1566	1381	1450
ENSCAFG00845017064	10	3	0	11
ENSCAFG00845005085	16	9	2	10
ENSCAFG00845017063	3	1	6	2
ENSCAFG00845005086	2	0	0	0
ENSCAFG00845017062	357	386	326	341
ENSCAFG00845005087	0	0	0	0
ENSCAFG00845017061	1199	1194	1015	1073
ENSCAFG00845005088	0	0	0	0
ENSCAFG00845017060	90	101	57	57
ENSCAFG00845005089	0	0	0	0
ENSCAFG00845029049	2542	2548	2586	2505
ENSCAFG00845029047	105	120	104	86
ENSCAFG00845029048	918	894	921	933
ENSCAFG00845029045	0	0	0	0
ENSCAFG00845029046	346	321	323	279
ENSCAFG00845029043	1	0	0	0
ENSCAFG00845029044	0	0	0	0
ENSCAFG00845029041	913	948	851	942

ENSCAFG00845029042	80	48	73	84
ENSCAFG00845029040	0	0	0	0
ENSCAFG00845017059	0	0	0	0
ENSCAFG00845017058	50	42	37	54
ENSCAFG00845017057	0	1	3	0
ENSCAFG00845005070	0	2	0	0
ENSCAFG00845017056	0	0	0	0
ENSCAFG00845005071	8	3	4	0
ENSCAFG00845017055	0	0	0	0
ENSCAFG00845005072	1	0	0	0
ENSCAFG00845017054	0	0	1	0
ENSCAFG00845005073	640	589	650	655
ENSCAFG00845017053	492	445	535	464
ENSCAFG00845005074	0	0	0	0
ENSCAFG00845017052	0	0	0	0
ENSCAFG00845005075	0	0	0	0
ENSCAFG00845017051	1	1	0	0
ENSCAFG00845005076	114	122	95	80
ENSCAFG00845017050	2562	2483	2538	2809
ENSCAFG00845005077	0	1	0	0
ENSCAFG00845005078	0	0	0	0
ENSCAFG00845005079	0	0	0	0
ENSCAFG00845029038	87	73	78	62
ENSCAFG00845029039	0	0	0	0
ENSCAFG00845029036	6	1	8	1
ENSCAFG00845029037	9	6	6	9
ENSCAFG00845029034	252	232	229	262
ENSCAFG00845029035	43	52	45	35
ENSCAFG00845029032	0	0	0	0
ENSCAFG00845029033	53	34	34	51
ENSCAFG00845029030	2565	2762	2356	2397
ENSCAFG00845029031	4711	4278	4529	4552
ENSCAFG00845017049	0	0	0	1
ENSCAFG00845017048	0	3	0	0
ENSCAFG00845017047	0	0	0	0
ENSCAFG00845020699	521	517	469	439
ENSCAFG00845017046	0	0	0	0
ENSCAFG00845017045	0	0	0	0
ENSCAFG00845005060	1857	1759	1723	1666
ENSCAFG00845017044	48	33	51	35
ENSCAFG00845005061	0	0	0	0

ENSCAFG00845017043	357	399	405	330
ENSCAFG00845005062	0	0	0	0
ENSCAFG00845017042	468	461	357	378
ENSCAFG00845005063	173	180	135	154
ENSCAFG00845017041	0	0	0	0
ENSCAFG00845020693	30	36	35	36
ENSCAFG00845005064	1497	1508	1474	1479
ENSCAFG00845017040	335	402	349	360
ENSCAFG00845020694	932	943	999	1098
ENSCAFG00845005065	0	0	0	0
ENSCAFG00845020691	2224	2235	1995	2066
ENSCAFG00845005066	63	72	64	47
ENSCAFG00845020692	0	0	0	0
ENSCAFG00845005067	0	0	0	0
ENSCAFG00845020697	2	0	3	2
ENSCAFG00845005068	0	0	0	0
ENSCAFG00845020698	3	0	0	0
ENSCAFG00845005069	3	9	0	1
ENSCAFG00845020695	0	2	0	0
ENSCAFG00845029029	2	2	0	1
ENSCAFG00845020696	1489	1484	1394	1411
ENSCAFG00845029027	0	0	0	0
ENSCAFG00845029028	277	272	270	320
ENSCAFG00845029025	12	7	10	15
ENSCAFG00845029026	8	14	7	6
ENSCAFG00845029023	30	20	35	33
ENSCAFG00845020690	855	781	787	857
ENSCAFG00845029024	0	0	0	0
ENSCAFG00845029021	0	0	0	0
ENSCAFG00845029022	203	213	200	179
ENSCAFG00845029020	0	0	0	0
ENSCAFG00845017038	38373	37379	32196	32179
ENSCAFG00845017037	8	5	4	6
ENSCAFG00845017036	363	409	416	440
ENSCAFG00845020688	1	3	0	3
ENSCAFG00845017035	0	0	0	0
ENSCAFG00845020689	0	0	0	0
ENSCAFG00845017034	800	810	881	884
ENSCAFG00845017033	27	32	37	36
ENSCAFG00845005050	14	24	22	22
ENSCAFG00845017032	1177	1123	939	1043

ENSCAFG00845005051	99	96	77	86
ENSCAFG00845017031	229	305	286	323
ENSCAFG00845005052	0	0	0	0
ENSCAFG00845017030	0	0	0	0
ENSCAFG00845020682	1	1	1	0
ENSCAFG00845005053	1	0	1	1
ENSCAFG00845020683	0	0	0	0
ENSCAFG00845005054	3124	3025	2817	2974
ENSCAFG00845020680	731	660	720	714
ENSCAFG00845005055	45	40	32	34
ENSCAFG00845020681	645	617	578	574
ENSCAFG00845005056	578	530	432	514
ENSCAFG00845020686	0	0	0	0
ENSCAFG00845005057	2473	2226	2342	2367
ENSCAFG00845020687	1082	1061	956	997
ENSCAFG00845005058	290	222	277	311
ENSCAFG00845020684	88	81	105	104
ENSCAFG00845029018	804	755	712	834
ENSCAFG00845005059	1206	1106	1210	1215
ENSCAFG00845020685	210	192	138	180
ENSCAFG00845029019	780	708	736	740
ENSCAFG00845029016	0	0	0	0
ENSCAFG00845029017	721	678	745	762
ENSCAFG00845029014	2264	2101	2235	2156
ENSCAFG00845029015	1808	1792	1655	1606
ENSCAFG00845029012	29	29	13	18
ENSCAFG00845019690	0	0	2	0
ENSCAFG00845029013	19	11	10	10
ENSCAFG00845019691	964	904	931	925
ENSCAFG00845029010	0	2	2	4
ENSCAFG00845019692	4	3	0	3
ENSCAFG00845029011	0	0	0	0
ENSCAFG00845019693	285	261	270	274
ENSCAFG00845019694	516	516	394	393
ENSCAFG00845019695	0	0	0	0
ENSCAFG00845019696	0	0	0	0
ENSCAFG00845019697	0	0	0	0
ENSCAFG00845019698	1082	989	962	959
ENSCAFG00845019699	0	0	0	0
ENSCAFG00845017039	24	13	10	26
ENSCAFG00845017027	0	0	0	0

ENSCAFG00845020679	586	539	618	591
ENSCAFG00845017026	3654	3322	3412	3502
ENSCAFG00845017025	0	0	0	1
ENSCAFG00845020677	0	0	0	0
ENSCAFG00845017024	250	260	238	225
ENSCAFG00845020678	0	0	0	1
ENSCAFG00845017023	0	0	0	0
ENSCAFG00845017022	1	5	1	4
ENSCAFG00845017021	4499	4111	4795	4922
ENSCAFG00845005040	0	0	0	0
ENSCAFG00845017020	0	0	0	0
ENSCAFG00845005041	385	353	381	310
ENSCAFG00845020671	0	0	0	0
ENSCAFG00845005042	496	452	340	290
ENSCAFG00845020672	932	939	671	643
ENSCAFG00845005043	0	0	0	0
ENSCAFG00845005044	0	0	0	0
ENSCAFG00845020670	2	0	3	3
ENSCAFG00845005045	10261	9851	9576	9752
ENSCAFG00845020675	0	0	2	3
ENSCAFG00845029009	1275	1171	1160	1223
ENSCAFG00845005046	0	0	0	0
ENSCAFG00845020676	0	3	1	0
ENSCAFG00845005047	0	0	0	0
ENSCAFG00845020673	0	0	0	0
ENSCAFG00845029007	4	2	0	2
ENSCAFG00845005048	0	0	0	0
ENSCAFG00845020674	0	0	0	0
ENSCAFG00845029008	227	253	233	250
ENSCAFG00845005049	0	0	3	0
ENSCAFG00845029005	1386	1346	1243	1245
ENSCAFG00845029006	274	259	290	248
ENSCAFG00845029003	0	0	0	0
ENSCAFG00845029004	1816	1841	1879	1869
ENSCAFG00845029001	3	2	1	1
ENSCAFG00845029002	1193	1271	1012	1053
ENSCAFG00845019680	0	0	0	0
ENSCAFG00845019681	0	0	0	0
ENSCAFG00845029000	2	0	0	0
ENSCAFG00845019682	5	4	7	8
ENSCAFG00845019683	11	9	12	12

ENSCAFG00845019684	0	0	0	0
ENSCAFG00845019685	0	3	0	0
ENSCAFG00845019686	387	386	330	336
ENSCAFG00845019687	2283	2227	3308	3345
ENSCAFG00845017029	0	0	0	0
ENSCAFG00845019688	0	0	0	0
ENSCAFG00845017028	315	301	315	366
ENSCAFG00845019689	0	0	0	0
ENSCAFG00845017016	578	455	462	493
ENSCAFG00845019679	1210	1122	1216	1225
ENSCAFG00845020668	940	887	909	847
ENSCAFG00845017015	0	0	0	0
ENSCAFG00845020669	246	269	282	270
ENSCAFG00845017014	783	753	773	717
ENSCAFG00845017013	0	0	0	0
ENSCAFG00845020667	24	26	46	44
ENSCAFG00845017012	2381	2451	2629	2789
ENSCAFG00845017011	525	439	383	391
ENSCAFG00845007691	139	164	137	147
ENSCAFG00845017010	0	0	0	0
ENSCAFG00845007690	0	0	0	0
ENSCAFG00845005030	2	1	0	0
ENSCAFG00845007693	10478	10223	9664	10090
ENSCAFG00845020660	673	664	663	686
ENSCAFG00845005031	0	0	0	0
ENSCAFG00845007692	0	0	0	0
ENSCAFG00845020661	0	1	6	0
ENSCAFG00845005032	317	285	268	247
ENSCAFG00845007695	2350	2419	2531	2601
ENSCAFG00845005033	0	0	0	0
ENSCAFG00845007694	3536	3296	3101	3130
ENSCAFG00845005034	0	0	0	0
ENSCAFG00845007697	3562	3365	3745	3782
ENSCAFG00845020664	365	385	365	348
ENSCAFG00845005035	26	27	21	24
ENSCAFG00845007696	124	94	106	116
ENSCAFG00845020665	1759	1756	1736	1753
ENSCAFG00845005036	0	1	0	0
ENSCAFG00845007699	21	36	38	24
ENSCAFG00845020662	837	836	942	911
ENSCAFG00845005037	13	4	7	5

ENSCAFG00845007698	2	2	2	8
ENSCAFG00845020663	0	0	0	0
ENSCAFG00845005038	206	183	215	234
ENSCAFG00845005039	17	15	10	12
ENSCAFG00845019670	1698	1638	1469	1553
ENSCAFG00845019671	514	468	450	511
ENSCAFG00845019672	0	0	0	0
ENSCAFG00845019673	4	2	4	2
ENSCAFG00845019674	919	858	941	971
ENSCAFG00845019675	0	0	0	0
ENSCAFG00845017019	9325	8986	10155	10505
ENSCAFG00845019676	316	284	331	383
ENSCAFG00845017018	302	280	325	378
ENSCAFG00845019677	46	59	43	46
ENSCAFG00845017017	204	170	236	188
ENSCAFG00845019678	565	500	472	468
ENSCAFG00845017005	1	0	2	1
ENSCAFG00845019668	0	0	0	0
ENSCAFG00845020657	0	0	0	1
ENSCAFG00845017004	17	11	13	24
ENSCAFG00845019669	9	18	10	7
ENSCAFG00845020658	0	0	0	0
ENSCAFG00845017003	3	2	1	0
ENSCAFG00845020655	0	0	1	0
ENSCAFG00845017002	12	20	11	16
ENSCAFG00845020656	0	0	0	0
ENSCAFG00845017001	1517	1405	1390	1402
ENSCAFG00845017000	77	78	65	41
ENSCAFG00845007680	846	891	885	843
ENSCAFG00845020659	2306	2175	2107	2196
ENSCAFG00845007682	0	0	0	0
ENSCAFG00845005020	12	7	2	4
ENSCAFG00845007681	184	166	193	200
ENSCAFG00845020650	0	0	0	0
ENSCAFG00845005021	57	82	103	106
ENSCAFG00845007684	0	0	0	0
ENSCAFG00845005022	0	0	0	0
ENSCAFG00845007683	0	0	0	0
ENSCAFG00845005023	123	107	128	152
ENSCAFG00845007686	0	0	0	0
ENSCAFG00845020653	632	586	568	504

ENSCAFG00845005024	15	10	6	14
ENSCAFG00845007685	72	44	55	44
ENSCAFG00845020654	4	5	4	4
ENSCAFG00845005025	1436	1355	1412	1451
ENSCAFG00845007688	982	847	919	885
ENSCAFG00845020651	0	0	0	0
ENSCAFG00845005026	9	10	9	11
ENSCAFG00845007687	48	49	54	53
ENSCAFG00845020652	0	0	0	0
ENSCAFG00845005027	9	2	1	1
ENSCAFG00845005028	869	776	868	811
ENSCAFG00845007689	896	809	888	964
ENSCAFG00845005029	1783	1679	1655	1779
ENSCAFG00845019660	2	3	2	0
ENSCAFG00845019661	580	630	599	643
ENSCAFG00845019662	117	121	112	92
ENSCAFG00845019663	944	1033	928	897
ENSCAFG00845017009	0	0	0	0
ENSCAFG00845019664	0	0	2	1
ENSCAFG00845017008	1	0	0	0
ENSCAFG00845019665	0	0	0	0
ENSCAFG00845017007	25	18	14	8
ENSCAFG00845019666	1	4	0	2
ENSCAFG00845017006	0	0	0	0
ENSCAFG00845019667	0	2	0	5
ENSCAFG00845017115	7946	7839	8293	8666
ENSCAFG00845019778	0	0	0	0
ENSCAFG00845020767	182	151	136	125
ENSCAFG00845017114	33	11	32	29
ENSCAFG00845019779	0	0	0	0
ENSCAFG00845020768	0	0	0	0
ENSCAFG00845017113	0	0	0	0
ENSCAFG00845020765	437	452	443	482
ENSCAFG00845017112	0	2	0	0
ENSCAFG00845020766	0	0	0	0
ENSCAFG00845017111	2131	2284	2190	2281
ENSCAFG00845017110	21	20	17	10
ENSCAFG00845007790	501	506	528	480
ENSCAFG00845020769	1	0	1	0
ENSCAFG00845007792	35	29	33	28
ENSCAFG00845005130	0	0	0	0

ENSCAFG00845007791	0	0	0	0
ENSCAFG00845020760	2	0	1	0
ENSCAFG00845005131	0	0	0	0
ENSCAFG00845007794	0	0	0	0
ENSCAFG00845005132	211	237	192	250
ENSCAFG00845007793	0	1	0	1
ENSCAFG00845005133	3	1	1	6
ENSCAFG00845007796	0	0	0	0
ENSCAFG00845020763	0	0	0	0
ENSCAFG00845005134	0	0	0	0
ENSCAFG00845007795	100	74	69	79
ENSCAFG00845020764	0	0	0	0
ENSCAFG00845005135	0	0	0	0
ENSCAFG00845007798	64	70	63	87
ENSCAFG00845020761	0	0	0	1
ENSCAFG00845005136	2	0	0	0
ENSCAFG00845007797	659	637	700	700
ENSCAFG00845020762	452	384	353	509
ENSCAFG00845005137	0	0	0	0
ENSCAFG00845005138	634	550	606	576
ENSCAFG00845007799	1794	1889	1451	1460
ENSCAFG00845005139	0	0	0	0
ENSCAFG00845019770	0	0	0	0
ENSCAFG00845019771	0	0	0	1
ENSCAFG00845019772	4289	4133	4107	4371
ENSCAFG00845019773	1587	1557	1386	1367
ENSCAFG00845017119	4	2	4	7
ENSCAFG00845019774	41	46	32	36
ENSCAFG00845017118	179	150	184	190
ENSCAFG00845019775	0	0	0	0
ENSCAFG00845017117	0	1	0	0
ENSCAFG00845019776	0	0	0	0
ENSCAFG00845017116	2199	2159	2404	2456
ENSCAFG00845019777	136	156	115	108
ENSCAFG00845017104	0	0	0	0
ENSCAFG00845019767	917	894	918	871
ENSCAFG00845020756	432	388	372	347
ENSCAFG00845017103	0	0	2	0
ENSCAFG00845019768	3	4	5	7
ENSCAFG00845020757	1273	1166	1320	1295
ENSCAFG00845017102	0	0	1	0

ENSCAFG00845019769	0	0	0	0
ENSCAFG00845020754	0	0	0	0
ENSCAFG00845017101	0	0	0	0
ENSCAFG00845020755	557	539	471	526
ENSCAFG00845017100	6	2	3	4
ENSCAFG00845020758	3294	3259	3538	3704
ENSCAFG00845020759	373	386	447	452
ENSCAFG00845007781	3898	3762	3383	3483
ENSCAFG00845007780	0	0	0	0
ENSCAFG00845005120	4	1	1	1
ENSCAFG00845007783	349	308	301	314
ENSCAFG00845005121	441	464	429	403
ENSCAFG00845007782	58	35	70	54
ENSCAFG00845005122	0	0	0	0
ENSCAFG00845007785	425	504	463	480
ENSCAFG00845020752	1930	1761	1743	1710
ENSCAFG00845005123	0	0	0	0
ENSCAFG00845007784	3996	3919	3622	3879
ENSCAFG00845020753	0	0	0	0
ENSCAFG00845005124	0	0	0	0
ENSCAFG00845007787	0	0	0	0
ENSCAFG00845020750	301	241	239	283
ENSCAFG00845005125	3477	3425	3089	3229
ENSCAFG00845007786	1497	1299	1213	1255
ENSCAFG00845020751	2307	2305	2056	2258
ENSCAFG00845005126	217	219	240	213
ENSCAFG00845007789	0	0	0	0
ENSCAFG00845030104	6	4	3	8
ENSCAFG00845005127	1850	1725	1612	1703
ENSCAFG00845007788	0	0	0	0
ENSCAFG00845030105	1881	1792	1697	1793
ENSCAFG00845005128	0	0	0	0
ENSCAFG00845030106	1678	1741	1453	1346
ENSCAFG00845005129	71	103	84	120
ENSCAFG00845030107	893	802	770	751
ENSCAFG00845030108	0	1	0	0
ENSCAFG00845030109	0	0	0	0
ENSCAFG00845019760	100	91	98	82
ENSCAFG00845019761	3662	3631	3196	3359
ENSCAFG00845017109	0	0	0	0
ENSCAFG00845019762	0	0	0	0

ENSCAFG00845017108	0	0	0	0
ENSCAFG00845019763	3	4	4	6
ENSCAFG00845030100	656	619	632	620
ENSCAFG00845017107	10	4	2	4
ENSCAFG00845019764	0	1	0	0
ENSCAFG00845030101	96	75	55	68
ENSCAFG00845017106	0	0	0	0
ENSCAFG00845019765	0	0	0	0
ENSCAFG00845030102	143	182	194	183
ENSCAFG00845017105	0	0	0	0
ENSCAFG00845019766	292	267	200	201
ENSCAFG00845030103	722	737	734	798
ENSCAFG00845019756	1719	1666	1579	1638
ENSCAFG00845020745	720	724	659	688
ENSCAFG00845019757	368	371	308	353
ENSCAFG00845020746	0	0	0	0
ENSCAFG00845019758	2407	2384	2377	2508
ENSCAFG00845020743	0	0	0	0
ENSCAFG00845019759	0	0	0	0
ENSCAFG00845020744	0	0	0	2
ENSCAFG00845020749	9	7	7	9
ENSCAFG00845020747	1	0	0	0
ENSCAFG00845020748	0	0	0	0
ENSCAFG00845007770	0	0	0	0
ENSCAFG00845007772	0	0	0	0
ENSCAFG00845005110	0	0	0	0
ENSCAFG00845007771	0	0	0	0
ENSCAFG00845005111	0	0	0	0
ENSCAFG00845007774	2012	1893	1691	1772
ENSCAFG00845020741	84	86	88	100
ENSCAFG00845005112	0	0	0	0
ENSCAFG00845007773	1090	1217	1226	1226
ENSCAFG00845020742	9	16	13	6
ENSCAFG00845005113	0	0	0	0
ENSCAFG00845007776	0	0	0	0
ENSCAFG00845005114	1	0	0	0
ENSCAFG00845007775	281	287	275	297
ENSCAFG00845020740	0	0	0	2
ENSCAFG00845005115	1547	1488	1203	1245
ENSCAFG00845007778	1561	1354	1209	1185
ENSCAFG00845030115	630	646	567	611

ENSCAFG00845005116	0	0	0	0
ENSCAFG00845007777	745	792	705	711
ENSCAFG00845030116	12	10	13	10
ENSCAFG00845005117	1	0	0	0
ENSCAFG00845030117	0	0	0	0
ENSCAFG00845005118	414	381	370	386
ENSCAFG00845007779	0	0	0	0
ENSCAFG00845030118	0	0	0	0
ENSCAFG00845005119	4463	4272	4050	4254
ENSCAFG00845030119	0	0	0	0
ENSCAFG00845019750	0	0	0	0
ENSCAFG00845019751	1811	1713	1766	1707
ENSCAFG00845030110	0	0	0	0
ENSCAFG00845019752	205	191	161	185
ENSCAFG00845030111	11	11	7	8
ENSCAFG00845019753	543	488	522	460
ENSCAFG00845030112	1328	1268	1288	1336
ENSCAFG00845019754	1166	1034	1110	1102
ENSCAFG00845030113	0	2	3	0
ENSCAFG00845019755	46	46	34	31
ENSCAFG00845030114	11	6	18	20
ENSCAFG00845019745	2	0	1	0
ENSCAFG00845020734	1893	1749	1417	1412
ENSCAFG00845019746	0	0	0	0
ENSCAFG00845020735	0	0	0	0
ENSCAFG00845019747	1016	907	903	824
ENSCAFG00845020732	173	209	190	147
ENSCAFG00845019748	0	0	0	0
ENSCAFG00845020733	7	5	7	6
ENSCAFG00845019749	559	548	493	572
ENSCAFG00845020738	0	0	0	0
ENSCAFG00845020739	4	2	1	0
ENSCAFG00845020736	743	710	699	693
ENSCAFG00845020737	0	0	0	0
ENSCAFG00845007761	3	0	2	3
ENSCAFG00845007760	0	0	0	0
ENSCAFG00845005100	1	1	0	0
ENSCAFG00845007763	985	1049	1075	999
ENSCAFG00845020730	853	774	811	845
ENSCAFG00845005101	1987	1770	2132	2238
ENSCAFG00845007762	512	534	508	501

ENSCAFG00845020731	39	39	47	37
ENSCAFG00845005102	277	296	277	241
ENSCAFG00845007765	995	872	910	909
ENSCAFG00845005103	0	0	0	0
ENSCAFG00845007764	0	0	0	0
ENSCAFG00845005104	6	11	10	8
ENSCAFG00845007767	593	500	459	547
ENSCAFG00845030126	2	2	1	0
ENSCAFG00845005105	17	23	31	48
ENSCAFG00845007766	0	1	0	0
ENSCAFG00845030127	307	328	364	310
ENSCAFG00845005106	0	0	0	0
ENSCAFG00845007769	680	664	799	698
ENSCAFG00845030128	4	6	3	5
ENSCAFG00845005107	0	0	0	0
ENSCAFG00845007768	0	0	0	0
ENSCAFG00845030129	1335	1200	1404	1491
ENSCAFG00845005108	1080	1119	1116	1161
ENSCAFG00845005109	0	0	0	0
ENSCAFG00845020729	0	0	0	0
ENSCAFG00845030120	1	0	0	0
ENSCAFG00845019740	176	188	159	186
ENSCAFG00845030121	0	0	1	0
ENSCAFG00845019741	0	0	0	0
ENSCAFG00845030122	0	0	1	0
ENSCAFG00845019742	0	0	0	1
ENSCAFG00845030123	0	0	0	0
ENSCAFG00845019743	1383	1331	1260	1199
ENSCAFG00845030124	1509	1496	1368	1355
ENSCAFG00845019744	0	0	0	0
ENSCAFG00845030125	1113	949	1147	1105
ENSCAFG00845019734	0	0	0	0
ENSCAFG00845020723	0	0	0	0
ENSCAFG00845019735	20	25	15	9
ENSCAFG00845020724	26	19	12	12
ENSCAFG00845019736	0	0	0	0
ENSCAFG00845020721	13262	13061	14113	14138
ENSCAFG00845019737	922	823	916	823
ENSCAFG00845020722	0	0	0	0
ENSCAFG00845019738	273	228	202	209
ENSCAFG00845020727	0	0	0	0

ENSCAFG00845019739	1	0	0	0
ENSCAFG00845020728	1197	1177	985	989
ENSCAFG00845020725	4	0	0	0
ENSCAFG00845020726	176	151	142	139
ENSCAFG00845007750	0	0	0	0
ENSCAFG00845007752	0	0	0	0
ENSCAFG00845007751	345	300	339	388
ENSCAFG00845020720	0	0	0	0
ENSCAFG00845007754	0	2	2	0
ENSCAFG00845007753	2	0	3	3
ENSCAFG00845007756	327	300	322	372
ENSCAFG00845030137	147	121	148	141
ENSCAFG00845007755	0	0	0	0
ENSCAFG00845030138	0	0	0	0
ENSCAFG00845007758	0	0	0	0
ENSCAFG00845030139	5	4	10	3
ENSCAFG00845007757	2240	2141	2306	2490
ENSCAFG00845007759	1014	920	973	898
ENSCAFG00845030130	0	0	0	0
ENSCAFG00845020718	93	112	95	87
ENSCAFG00845030131	54	60	54	46
ENSCAFG00845020719	0	0	0	0
ENSCAFG00845030132	320	241	218	267
ENSCAFG00845019730	351	336	300	313
ENSCAFG00845030133	465	505	454	456
ENSCAFG00845019731	844	837	667	673
ENSCAFG00845030134	0	0	0	0
ENSCAFG00845019732	35	27	49	44
ENSCAFG00845030135	2	3	11	4
ENSCAFG00845019733	20	29	18	23
ENSCAFG00845030136	4	1	4	1
ENSCAFG00845019723	487	488	503	465
ENSCAFG00845020712	11	8	24	15
ENSCAFG00845019724	15	11	22	18
ENSCAFG00845020713	1	0	1	3
ENSCAFG00845019725	1782	1690	1667	1673
ENSCAFG00845020710	1726	1670	1480	1660
ENSCAFG00845019726	0	0	0	0
ENSCAFG00845020711	0	0	0	0
ENSCAFG00845019727	163	125	157	147
ENSCAFG00845020716	2	0	0	0

ENSCAFG00845019728	0	0	0	0
ENSCAFG00845020717	0	0	0	0
ENSCAFG00845019729	9349	9405	10039	9977
ENSCAFG00845020714	1684	1609	1488	1531
ENSCAFG00845020715	0	0	0	0
ENSCAFG00845007741	240	276	223	224
ENSCAFG00845007740	11	6	7	8
ENSCAFG00845007743	0	0	0	0
ENSCAFG00845007742	46	41	65	49
ENSCAFG00845007745	341	334	340	271
ENSCAFG00845030148	0	0	0	0
ENSCAFG00845007744	43	38	59	57
ENSCAFG00845030149	776	693	856	850
ENSCAFG00845007747	206	192	245	261
ENSCAFG00845007746	330	313	395	426
ENSCAFG00845007749	0	0	0	0
ENSCAFG00845007748	2466	2306	2311	2244
ENSCAFG00845020709	0	0	0	0
ENSCAFG00845030140	1162	1087	1078	1127
ENSCAFG00845030141	315	302	324	304
ENSCAFG00845020707	0	0	0	0
ENSCAFG00845030142	257	298	293	310
ENSCAFG00845020708	0	0	1	1
ENSCAFG00845030143	1	1	1	0
ENSCAFG00845030144	0	0	0	2
ENSCAFG00845019720	0	0	0	0
ENSCAFG00845030145	247	279	259	250
ENSCAFG00845019721	106	111	89	116
ENSCAFG00845030146	0	0	0	0
ENSCAFG00845019722	52	32	47	69
ENSCAFG00845030147	115	128	103	154
ENSCAFG00845019712	0	1	2	1
ENSCAFG00845020701	0	0	0	0
ENSCAFG00845019713	93	78	129	120
ENSCAFG00845020702	59	61	79	79
ENSCAFG00845019714	5217	5187	4663	4676
ENSCAFG00845019715	157	173	138	134
ENSCAFG00845020700	0	0	0	0
ENSCAFG00845019716	0	0	0	0
ENSCAFG00845020705	0	0	0	0
ENSCAFG00845019717	0	0	0	0

ENSCAFG00845020706	0	0	0	0
ENSCAFG00845019718	0	0	0	0
ENSCAFG00845020703	4	0	0	0
ENSCAFG00845019719	318	275	277	250
ENSCAFG00845020704	8	6	11	8
ENSCAFG00845030150	0	0	0	0
ENSCAFG00845007730	12	15	15	25
ENSCAFG00845007732	68	74	93	95
ENSCAFG00845007731	0	0	0	0
ENSCAFG00845007734	571	576	619	609
ENSCAFG00845030159	0	0	0	0
ENSCAFG00845007733	0	0	0	0
ENSCAFG00845007736	0	0	0	0
ENSCAFG00845007735	0	0	0	0
ENSCAFG00845007738	1	0	0	0
ENSCAFG00845007737	0	0	2	1
ENSCAFG00845007739	0	0	0	0
ENSCAFG00845030151	8	5	7	19
ENSCAFG00845030152	36	24	30	27
ENSCAFG00845030153	0	0	0	0
ENSCAFG00845030154	0	0	0	0
ENSCAFG00845030155	0	0	0	0
ENSCAFG00845030156	0	0	0	0
ENSCAFG00845019710	1195	1210	1260	1328
ENSCAFG00845030157	2907	2753	3125	3066
ENSCAFG00845019711	0	0	0	0
ENSCAFG00845030158	0	0	0	0
ENSCAFG00845019701	4315	4192	4176	4498
ENSCAFG00845019702	0	0	0	0
ENSCAFG00845019703	1278	1147	1141	1162
ENSCAFG00845019705	290	341	347	395
ENSCAFG00845019706	0	0	0	0
ENSCAFG00845019707	10	18	12	15
ENSCAFG00845030160	1264	1159	1251	1253
ENSCAFG00845019708	0	0	0	0
ENSCAFG00845030161	208	195	258	255
ENSCAFG00845019709	0	0	0	0
ENSCAFG00845007721	0	0	0	0
ENSCAFG00845007720	5264	4873	4805	4822
ENSCAFG00845007723	1574	1518	1490	1567
ENSCAFG00845007722	126	127	107	89

ENSCAFG00845007725	573	560	506	463
ENSCAFG00845007724	36	24	60	43
ENSCAFG00845007727	4	4	5	7
ENSCAFG00845007726	1	7	2	2
ENSCAFG00845007729	147	137	202	168
ENSCAFG00845007728	0	0	0	0
ENSCAFG00845030162	0	0	0	0
ENSCAFG00845030163	4	0	0	0
ENSCAFG00845030164	374	363	322	377
ENSCAFG00845030165	7	6	20	13
ENSCAFG00845030166	288	291	266	332
ENSCAFG00845030167	0	0	0	0
ENSCAFG00845030168	1723	1720	1362	1491
ENSCAFG00845019700	0	0	0	1
ENSCAFG00845030169	0	0	0	3
ENSCAFG00845017199	101	97	117	102
ENSCAFG00845017198	0	0	0	0
ENSCAFG00845017197	1	4	0	2
ENSCAFG00845017196	899	873	791	816
ENSCAFG00845017195	667	555	605	642
ENSCAFG00845017194	0	0	0	0
ENSCAFG00845017193	174	157	151	125
ENSCAFG00845017192	77	82	54	81
ENSCAFG00845017191	1224	1284	1353	1449
ENSCAFG00845017190	0	0	0	0
ENSCAFG00845029179	0	1	1	0
ENSCAFG00845029177	0	0	2	0
ENSCAFG00845029178	0	5	0	0
ENSCAFG00845029175	2	0	3	2
ENSCAFG00845029176	1	4	0	8
ENSCAFG00845029173	5	0	0	0
ENSCAFG00845029174	0	3	0	0
ENSCAFG00845029171	2395	2263	2289	2246
ENSCAFG00845029172	0	0	0	0
ENSCAFG00845029170	0	0	0	0
ENSCAFG00845017189	3102	3110	2937	2872
ENSCAFG00845017188	154	185	111	163
ENSCAFG00845017187	0	0	0	0
ENSCAFG00845017186	0	0	0	0
ENSCAFG00845017185	2	1	2	6
ENSCAFG00845017184	3	2	0	1

ENSCAFG00845017183	749	739	766	819
ENSCAFG00845017182	28	25	19	10
ENSCAFG00845017181	4	1	4	3
ENSCAFG00845017180	5	2	7	9
ENSCAFG00845029168	520	491	551	496
ENSCAFG00845029169	944	770	911	984
ENSCAFG00845029166	3	2	1	2
ENSCAFG00845029167	0	0	0	0
ENSCAFG00845029164	251	212	176	215
ENSCAFG00845029165	30	29	41	36
ENSCAFG00845029162	126	107	102	81
ENSCAFG00845029163	672	661	578	647
ENSCAFG00845029160	3	2	7	1
ENSCAFG00845029161	565	480	628	642
ENSCAFG00845017179	0	0	0	0
ENSCAFG00845005190	33	64	57	57
ENSCAFG00845017178	0	0	4	3
ENSCAFG00845005191	22	31	25	26
ENSCAFG00845017177	0	0	0	0
ENSCAFG00845005192	0	0	0	0
ENSCAFG00845017176	36	34	42	29
ENSCAFG00845005193	0	0	0	0
ENSCAFG00845017175	0	0	0	0
ENSCAFG00845005194	0	0	0	0
ENSCAFG00845017174	2077	1997	1727	1799
ENSCAFG00845005195	0	0	0	0
ENSCAFG00845017173	588	601	621	633
ENSCAFG00845005196	0	0	0	0
ENSCAFG00845017172	0	2	0	0
ENSCAFG00845005197	0	0	0	0
ENSCAFG00845017171	29323	28433	30079	32115
ENSCAFG00845005198	1565	1515	1394	1391
ENSCAFG00845017170	0	0	0	0
ENSCAFG00845005199	1	2	0	0
ENSCAFG00845029159	0	0	0	0
ENSCAFG00845029157	496	456	593	565
ENSCAFG00845029158	3	4	11	10
ENSCAFG00845029155	57	68	42	60
ENSCAFG00845029156	1	0	0	0
ENSCAFG00845029153	335	230	256	204
ENSCAFG00845029154	8	8	4	7

ENSCAFG00845029151	0	0	1	0
ENSCAFG00845029152	0	0	0	0
ENSCAFG00845029150	216	239	212	242
ENSCAFG00845017169	900	862	798	814
ENSCAFG00845017168	0	0	0	0
ENSCAFG00845017167	0	0	0	0
ENSCAFG00845005180	2	0	1	0
ENSCAFG00845017166	946	908	894	926
ENSCAFG00845005181	2	0	0	0
ENSCAFG00845017165	7	1	2	3
ENSCAFG00845005182	27	13	11	18
ENSCAFG00845017164	0	0	1	0
ENSCAFG00845005183	3	1	0	2
ENSCAFG00845017163	932	830	751	800
ENSCAFG00845005184	0	0	0	0
ENSCAFG00845017162	29	22	30	29
ENSCAFG00845005185	3	0	0	0
ENSCAFG00845017161	0	0	0	0
ENSCAFG00845005186	567	546	460	479
ENSCAFG00845017160	189	207	239	210
ENSCAFG00845005187	2	0	1	1
ENSCAFG00845005188	0	0	0	0
ENSCAFG00845005189	1	0	0	0
ENSCAFG00845029148	0	0	0	0
ENSCAFG00845029149	550	522	500	450
ENSCAFG00845029146	1392	1388	1477	1646
ENSCAFG00845029147	1700	1580	1900	1967
ENSCAFG00845029144	0	0	0	0
ENSCAFG00845029145	1	0	0	1
ENSCAFG00845029142	0	0	0	0
ENSCAFG00845029143	2262	2203	2216	2349
ENSCAFG00845029140	0	0	0	0
ENSCAFG00845029141	0	0	0	0
ENSCAFG00845017159	1222	1121	1083	1147
ENSCAFG00845017158	18	14	0	1
ENSCAFG00845017157	653	604	550	640
ENSCAFG00845017156	1136	1018	985	1048
ENSCAFG00845017155	0	0	0	0
ENSCAFG00845005170	0	0	0	0
ENSCAFG00845017154	969	1023	907	975
ENSCAFG00845005171	353	328	247	218

ENSCAFG00845017153	0	0	0	0
ENSCAFG00845005172	554	504	509	534
ENSCAFG00845017152	2	2	1	6
ENSCAFG00845005173	0	0	0	0
ENSCAFG00845017151	0	0	0	0
ENSCAFG00845005174	1149	1068	1044	1075
ENSCAFG00845017150	0	0	0	0
ENSCAFG00845005175	6	8	11	11
ENSCAFG00845005176	0	0	0	0
ENSCAFG00845005177	0	0	0	0
ENSCAFG00845005178	0	0	0	0
ENSCAFG00845005179	421	469	404	423
ENSCAFG00845029139	78	92	80	60
ENSCAFG00845029137	7	10	14	9
ENSCAFG00845029138	11	11	12	7
ENSCAFG00845029135	0	0	0	0
ENSCAFG00845029136	254	235	249	242
ENSCAFG00845029133	0	0	0	0
ENSCAFG00845029134	276	286	248	260
ENSCAFG00845029131	0	0	0	0
ENSCAFG00845029132	1804	1669	1584	1557
ENSCAFG00845029130	299	240	243	302
ENSCAFG00845017148	0	0	0	0
ENSCAFG00845017147	485	455	432	500
ENSCAFG00845017146	4	9	7	7
ENSCAFG00845020798	889	786	653	667
ENSCAFG00845017145	0	0	0	0
ENSCAFG00845020799	0	0	0	0
ENSCAFG00845017144	3	0	6	3
ENSCAFG00845017143	0	0	0	0
ENSCAFG00845005160	47	49	28	18
ENSCAFG00845017142	87	71	66	77
ENSCAFG00845005161	0	0	0	1
ENSCAFG00845017141	0	0	0	0
ENSCAFG00845005162	0	0	0	0
ENSCAFG00845017140	62	53	50	68
ENSCAFG00845020792	0	0	0	0
ENSCAFG00845005163	0	0	0	0
ENSCAFG00845020793	0	0	0	0
ENSCAFG00845005164	1	0	0	0
ENSCAFG00845020790	1	0	0	0

ENSCAFG00845005165	1	3	9	4
ENSCAFG00845020791	544	586	498	520
ENSCAFG00845005166	20216	19502	17443	18439
ENSCAFG00845020796	974	1016	815	813
ENSCAFG00845005167	163	183	166	200
ENSCAFG00845020797	260	244	286	317
ENSCAFG00845005168	36	44	27	35
ENSCAFG00845020794	0	0	0	0
ENSCAFG00845029128	288	220	263	247
ENSCAFG00845005169	0	0	0	0
ENSCAFG00845020795	0	0	0	0
ENSCAFG00845029129	1688	1576	1824	1680
ENSCAFG00845029126	0	0	0	0
ENSCAFG00845029127	1221	1152	1091	1088
ENSCAFG00845029124	1231	1203	986	960
ENSCAFG00845029125	0	0	0	3
ENSCAFG00845029122	1012	1019	1012	994
ENSCAFG00845029123	0	0	0	0
ENSCAFG00845029120	0	0	0	0
ENSCAFG00845029121	267	278	254	328
ENSCAFG00845017149	255	232	171	172
ENSCAFG00845017137	1296	1241	1202	1290
ENSCAFG00845020789	956	875	986	1003
ENSCAFG00845017136	0	0	0	0
ENSCAFG00845017135	0	0	0	0
ENSCAFG00845020787	0	0	0	0
ENSCAFG00845017134	211	209	215	254
ENSCAFG00845020788	237	190	213	206
ENSCAFG00845017133	0	0	0	0
ENSCAFG00845017132	0	0	0	0
ENSCAFG00845017131	0	0	0	0
ENSCAFG00845005150	0	0	0	0
ENSCAFG00845017130	0	0	0	0
ENSCAFG00845005151	3160	3140	2903	3218
ENSCAFG00845020781	67	74	38	55
ENSCAFG00845005152	2683	2443	2693	2837
ENSCAFG00845005153	101	74	77	79
ENSCAFG00845005154	0	0	0	0
ENSCAFG00845020780	0	0	0	0
ENSCAFG00845005155	0	0	0	1
ENSCAFG00845020785	377	361	336	357

ENSCAFG00845029119	31	28	23	37
ENSCAFG00845005156	0	0	0	0
ENSCAFG00845020786	1760	1683	2134	2160
ENSCAFG00845005157	128	110	112	121
ENSCAFG00845020783	1405	1305	1526	1559
ENSCAFG00845029117	0	0	0	0
ENSCAFG00845005158	5	6	4	12
ENSCAFG00845020784	0	0	0	0
ENSCAFG00845029118	144	148	170	163
ENSCAFG00845005159	2158	1989	2105	2070
ENSCAFG00845029115	0	0	0	0
ENSCAFG00845029116	741	745	905	914
ENSCAFG00845029113	17	10	8	3
ENSCAFG00845029114	1951	1843	1862	1920
ENSCAFG00845029111	0	0	0	0
ENSCAFG00845029112	0	0	0	0
ENSCAFG00845019790	0	0	0	0
ENSCAFG00845019791	0	0	0	0
ENSCAFG00845029110	788	774	675	762
ENSCAFG00845019792	2	0	1	0
ENSCAFG00845019793	418	366	361	348
ENSCAFG00845019794	0	0	0	0
ENSCAFG00845019795	0	1	0	0
ENSCAFG00845019796	0	0	0	0
ENSCAFG00845019797	467	441	541	501
ENSCAFG00845017139	1238	1139	1167	1143
ENSCAFG00845019798	38	53	59	67
ENSCAFG00845017138	0	0	0	0
ENSCAFG00845019799	0	0	0	0
ENSCAFG00845017126	9510	9235	10015	9863
ENSCAFG00845019789	0	0	0	0
ENSCAFG00845020778	0	0	0	1
ENSCAFG00845017125	1807	1725	1753	1740
ENSCAFG00845020779	113	114	91	82
ENSCAFG00845017124	0	0	0	0
ENSCAFG00845020776	2735	2578	2623	2868
ENSCAFG00845017123	0	0	0	1
ENSCAFG00845020777	5	6	12	8
ENSCAFG00845017122	0	0	0	0
ENSCAFG00845017121	2196	2093	2080	1968
ENSCAFG00845017120	0	0	0	0

ENSCAFG00845005140	0	0	1	0
ENSCAFG00845020770	0	0	0	0
ENSCAFG00845005141	475	472	479	499
ENSCAFG00845020771	0	0	0	0
ENSCAFG00845005142	0	0	0	0
ENSCAFG00845005143	1	6	0	2
ENSCAFG00845005144	3	6	2	1
ENSCAFG00845020774	26	19	15	21
ENSCAFG00845029108	128	160	125	108
ENSCAFG00845005145	1	0	0	0
ENSCAFG00845020775	42	70	71	76
ENSCAFG00845029109	5	3	1	3
ENSCAFG00845005146	3	2	0	4
ENSCAFG00845020772	0	0	0	1
ENSCAFG00845029106	73	110	84	100
ENSCAFG00845005147	0	0	0	0
ENSCAFG00845020773	1651	1634	1610	1563
ENSCAFG00845029107	6	5	10	6
ENSCAFG00845005148	2707	2656	2452	2765
ENSCAFG00845029104	108	89	82	82
ENSCAFG00845005149	0	0	0	0
ENSCAFG00845029105	644	595	611	613
ENSCAFG00845029102	2	0	1	0
ENSCAFG00845029103	1750	1626	1598	1656
ENSCAFG00845029100	1045	1014	952	895
ENSCAFG00845029101	0	0	0	0
ENSCAFG00845019780	0	1	1	0
ENSCAFG00845019781	0	0	0	0
ENSCAFG00845019782	19	17	27	21
ENSCAFG00845019783	0	0	0	0
ENSCAFG00845019784	1261	1303	1247	1126
ENSCAFG00845019785	133	116	96	181
ENSCAFG00845017129	368	317	390	379
ENSCAFG00845019786	46	51	42	39
ENSCAFG00845017128	8002	7920	7784	8291
ENSCAFG00845019787	0	0	0	0
ENSCAFG00845017127	6	0	0	0
ENSCAFG00845019788	0	0	0	0
ENSCAFG00845029098	405	365	429	384
ENSCAFG00845029099	0	0	0	0
ENSCAFG00845029096	0	0	0	2

ENSCAFG00845029097	0	0	0	0
ENSCAFG00845029094	0	0	0	0
ENSCAFG00845029095	2044	1869	1852	1969
ENSCAFG00845029092	990	942	906	873
ENSCAFG00845029093	38	19	22	23
ENSCAFG00845029090	191	176	185	216
ENSCAFG00845029091	0	0	0	0
ENSCAFG00845029089	1021	1087	919	1011
ENSCAFG00845029087	0	0	0	0
ENSCAFG00845029088	11643	11555	13191	13347
ENSCAFG00845029085	402	318	375	334
ENSCAFG00845029086	353	329	270	292
ENSCAFG00845029083	1872	1705	1733	1737
ENSCAFG00845029084	0	0	0	0
ENSCAFG00845029081	284	233	261	278
ENSCAFG00845029082	0	0	0	0
ENSCAFG00845029080	0	0	0	0
ENSCAFG00845017099	44	45	41	40
ENSCAFG00845017098	351	244	247	288
ENSCAFG00845017097	1222	1243	1270	1182
ENSCAFG00845017096	2	0	0	0
ENSCAFG00845017095	954	920	838	848
ENSCAFG00845017094	500	486	460	476
ENSCAFG00845017093	218	146	166	156
ENSCAFG00845017092	0	0	1	0
ENSCAFG00845017091	3	2	0	1
ENSCAFG00845017090	0	0	0	0
ENSCAFG00845029078	0	0	0	0
ENSCAFG00845029079	0	0	0	0
ENSCAFG00845029076	6	7	15	7
ENSCAFG00845029077	404	381	410	413
ENSCAFG00845029074	0	0	1	0
ENSCAFG00845029075	0	0	0	0
ENSCAFG00845029072	4	8	5	5
ENSCAFG00845029073	0	0	2	0
ENSCAFG00845029070	30	25	24	15
ENSCAFG00845029071	1	2	5	8
ENSCAFG00845017089	3347	3275	2835	2967
ENSCAFG00845017088	1	0	6	1
ENSCAFG00845017087	769	750	737	771
ENSCAFG00845017086	0	0	0	0

ENSCAFG00845017085	1	0	0	0
ENSCAFG00845017084	0	0	0	0
ENSCAFG00845017083	423	394	449	479
ENSCAFG00845017082	0	0	0	0
ENSCAFG00845017081	619	592	724	719
ENSCAFG00845017080	0	0	0	0
ENSCAFG00845029069	469	367	431	410
ENSCAFG00845029067	593	632	627	570
ENSCAFG00845029068	3	3	1	0
ENSCAFG00845029065	0	0	0	0
ENSCAFG00845029066	0	0	0	0
ENSCAFG00845029063	1	0	1	0
ENSCAFG00845029064	0	0	0	0
ENSCAFG00845029061	117	118	102	133
ENSCAFG00845029062	0	0	0	0
ENSCAFG00845029060	3631	3582	3386	3530
ENSCAFG00845030050	654	746	680	704
ENSCAFG00845030051	0	0	0	0
ENSCAFG00845007710	287	343	269	299
ENSCAFG00845007712	0	0	0	0
ENSCAFG00845007711	1451	1339	1288	1334
ENSCAFG00845007714	0	0	0	0
ENSCAFG00845007713	3	2	0	3
ENSCAFG00845007716	0	0	0	0
ENSCAFG00845007715	0	0	0	0
ENSCAFG00845007718	0	0	0	0
ENSCAFG00845007717	304	331	295	274
ENSCAFG00845030052	0	0	0	0
ENSCAFG00845007719	6802	6703	6239	6299
ENSCAFG00845030053	39	24	20	43
ENSCAFG00845030054	0	0	1	0
ENSCAFG00845030055	0	0	0	0
ENSCAFG00845030056	604	538	685	780
ENSCAFG00845030057	0	0	0	0
ENSCAFG00845030058	2885	2880	2771	2809
ENSCAFG00845030059	0	0	0	0
ENSCAFG00845030060	820	744	770	627
ENSCAFG00845030061	1	1	2	4
ENSCAFG00845030062	1274	1216	1225	1341
ENSCAFG00845007701	453	401	362	427
ENSCAFG00845007700	1	0	2	1

ENSCAFG00845007703	2	1	3	1
ENSCAFG00845007702	374	386	397	406
ENSCAFG00845007705	2962	2972	2946	2890
ENSCAFG00845007704	0	0	1	0
ENSCAFG00845007707	2708	2670	2408	2398
ENSCAFG00845007706	0	0	0	0
ENSCAFG00845007709	742	769	709	724
ENSCAFG00845030063	2	0	1	0
ENSCAFG00845007708	4086	3906	4280	4245
ENSCAFG00845030064	259	240	276	322
ENSCAFG00845030065	251	272	230	207
ENSCAFG00845030066	385	382	407	430
ENSCAFG00845030067	0	0	0	0
ENSCAFG00845030068	0	0	3	2
ENSCAFG00845030069	541	488	463	451
ENSCAFG00845030070	2007	1969	1898	2041
ENSCAFG00845030071	0	0	0	0
ENSCAFG00845030072	431	407	432	465
ENSCAFG00845030073	0	0	0	0
ENSCAFG00845030074	4	4	3	4
ENSCAFG00845030075	754	752	618	648
ENSCAFG00845030076	202	230	137	157
ENSCAFG00845030077	5	5	3	9
ENSCAFG00845030078	0	2	0	2
ENSCAFG00845030079	0	1	1	0
ENSCAFG00845030080	2	1	2	2
ENSCAFG00845030081	573	615	591	568
ENSCAFG00845030082	416	396	469	476
ENSCAFG00845030083	754	739	732	796
ENSCAFG00845030084	0	0	0	0
ENSCAFG00845030085	0	0	0	0
ENSCAFG00845030086	727	760	668	727
ENSCAFG00845030087	0	0	0	0
ENSCAFG00845030088	0	0	0	0
ENSCAFG00845030089	711	644	635	747
ENSCAFG00845030090	0	0	0	0
ENSCAFG00845030091	11	11	9	6
ENSCAFG00845030092	1170	1101	1019	1014
ENSCAFG00845030093	0	0	0	0
ENSCAFG00845030094	505	437	515	493
ENSCAFG00845030095	117	111	121	135

ENSCAFG00845030096	5	4	8	3
ENSCAFG00845030097	15	15	13	22
ENSCAFG00845030098	0	0	0	1
ENSCAFG00845030099	149	140	92	75
ENSCAFG00845017236	13	26	7	8
ENSCAFG00845019899	0	0	0	0
ENSCAFG00845020888	2135	2015	2412	2382
ENSCAFG00845017235	0	0	5	0
ENSCAFG00845020889	0	0	0	0
ENSCAFG00845017234	0	0	0	0
ENSCAFG00845020886	7	6	0	6
ENSCAFG00845017233	2458	2330	2647	2609
ENSCAFG00845020887	1550	1517	1367	1368
ENSCAFG00845017232	264	257	238	245
ENSCAFG00845017231	0	0	0	0
ENSCAFG00845017230	0	0	0	0
ENSCAFG00845005250	1534	1532	1809	1766
ENSCAFG00845020880	0	0	0	0
ENSCAFG00845005251	666	676	694	649
ENSCAFG00845020881	0	0	0	0
ENSCAFG00845005252	1	3	0	0
ENSCAFG00845005253	0	0	0	0
ENSCAFG00845005254	0	0	0	0
ENSCAFG00845020884	4	2	3	0
ENSCAFG00845029218	1	0	6	3
ENSCAFG00845005255	2	2	0	0
ENSCAFG00845020885	0	0	0	0
ENSCAFG00845029219	1408	1396	1412	1474
ENSCAFG00845005256	2508	2402	2735	2785
ENSCAFG00845020882	4010	3850	3836	3666
ENSCAFG00845029216	13	4	4	5
ENSCAFG00845005257	0	0	0	0
ENSCAFG00845020883	3	5	3	0
ENSCAFG00845029217	126	108	121	114
ENSCAFG00845005258	6	4	7	12
ENSCAFG00845029214	0	0	0	0
ENSCAFG00845030214	0	5	7	5
ENSCAFG00845005259	0	0	0	0
ENSCAFG00845029215	1	5	0	1
ENSCAFG00845030215	223	189	189	189
ENSCAFG00845029212	0	0	0	0

ENSCAFG00845030216	0	0	0	0
ENSCAFG00845029213	0	0	0	0
ENSCAFG00845030217	0	0	0	0
ENSCAFG00845029210	472	470	535	535
ENSCAFG00845030218	0	0	0	0
ENSCAFG00845029211	0	0	0	0
ENSCAFG00845030219	105	96	138	118
ENSCAFG00845019890	7	10	9	8
ENSCAFG00845019891	505	451	499	471
ENSCAFG00845019892	358	343	364	346
ENSCAFG00845019893	0	0	0	0
ENSCAFG00845019894	0	0	0	0
ENSCAFG00845019895	0	0	0	0
ENSCAFG00845030210	3	1	0	1
ENSCAFG00845017239	695	668	560	599
ENSCAFG00845019896	0	0	0	0
ENSCAFG00845030211	2057	1957	1830	1922
ENSCAFG00845017238	11	4	3	3
ENSCAFG00845019897	0	0	0	0
ENSCAFG00845030212	0	0	0	2
ENSCAFG00845017237	1177	1078	1093	1122
ENSCAFG00845019898	2	4	1	1
ENSCAFG00845030213	0	0	0	0
ENSCAFG00845017225	370	333	323	354
ENSCAFG00845019888	615	546	595	584
ENSCAFG00845020877	0	0	0	0
ENSCAFG00845017224	165	168	161	177
ENSCAFG00845019889	0	0	0	0
ENSCAFG00845020878	200	243	174	186
ENSCAFG00845017223	0	0	0	0
ENSCAFG00845020875	32	25	55	44
ENSCAFG00845017222	1	0	0	0
ENSCAFG00845020876	7	2	6	6
ENSCAFG00845017221	10	12	15	11
ENSCAFG00845017220	0	0	0	0
ENSCAFG00845020879	744	672	699	692
ENSCAFG00845005240	0	0	0	0
ENSCAFG00845020870	308	283	270	303
ENSCAFG00845005241	231	184	184	160
ENSCAFG00845029209	1	2	0	2
ENSCAFG00845005242	0	0	0	0

ENSCAFG00845005243	0	0	0	0
ENSCAFG00845020873	45	49	77	50
ENSCAFG00845029207	1243	1205	1159	1176
ENSCAFG00845005244	0	0	0	0
ENSCAFG00845020874	0	0	0	1
ENSCAFG00845029208	1368	1367	1223	1299
ENSCAFG00845005245	0	0	0	0
ENSCAFG00845020871	11	5	6	1
ENSCAFG00845029205	2	0	0	3
ENSCAFG00845005246	0	0	0	0
ENSCAFG00845020872	181	172	199	206
ENSCAFG00845029206	0	0	0	0
ENSCAFG00845005247	65	29	66	53
ENSCAFG00845029203	0	0	0	0
ENSCAFG00845030225	65	47	68	85
ENSCAFG00845005248	2	0	0	0
ENSCAFG00845029204	0	0	0	0
ENSCAFG00845030226	19	20	5	11
ENSCAFG00845005249	0	0	0	0
ENSCAFG00845029201	192	169	138	141
ENSCAFG00845030227	51	43	51	54
ENSCAFG00845029202	0	0	0	0
ENSCAFG00845030228	0	0	0	0
ENSCAFG00845030229	67	75	58	54
ENSCAFG00845029200	2432	2445	2211	2350
ENSCAFG00845019880	0	0	0	0
ENSCAFG00845019881	1286	1305	1268	1279
ENSCAFG00845019882	0	0	0	0
ENSCAFG00845019883	0	0	0	0
ENSCAFG00845030220	0	0	0	0
ENSCAFG00845017229	0	0	0	0
ENSCAFG00845019884	0	0	0	0
ENSCAFG00845030221	0	3	2	1
ENSCAFG00845017228	617	573	531	476
ENSCAFG00845019885	1260	1118	1088	1167
ENSCAFG00845030222	4	4	8	3
ENSCAFG00845017227	377	375	595	561
ENSCAFG00845019886	0	0	0	0
ENSCAFG00845030223	2	6	7	8
ENSCAFG00845017226	0	0	0	0
ENSCAFG00845019887	0	0	0	0

ENSCAFG00845030224	942	910	676	627
ENSCAFG00845017214	0	0	0	0
ENSCAFG00845019877	4	0	1	1
ENSCAFG00845020866	0	0	0	0
ENSCAFG00845017213	3	7	7	6
ENSCAFG00845019878	271	249	217	201
ENSCAFG00845020867	0	0	0	0
ENSCAFG00845017212	189	152	132	129
ENSCAFG00845019879	0	0	0	0
ENSCAFG00845020864	0	3	0	0
ENSCAFG00845017211	777	708	720	776
ENSCAFG00845020865	315	314	248	309
ENSCAFG00845017210	0	0	0	0
ENSCAFG00845020868	514	477	492	467
ENSCAFG00845020869	1426	1381	1417	1516
ENSCAFG00845007891	2	1	0	0
ENSCAFG00845007890	0	0	0	0
ENSCAFG00845005230	6541	6276	5962	5803
ENSCAFG00845007893	0	0	0	0
ENSCAFG00845005231	1252	1267	1214	1318
ENSCAFG00845007892	1	1	0	0
ENSCAFG00845005232	0	0	0	0
ENSCAFG00845007895	0	0	0	0
ENSCAFG00845020862	579	487	580	561
ENSCAFG00845005233	0	0	0	0
ENSCAFG00845007894	284	260	257	280
ENSCAFG00845020863	0	0	0	0
ENSCAFG00845005234	0	0	0	0
ENSCAFG00845007897	1753	1784	1712	1644
ENSCAFG00845020860	4607	4574	4079	3834
ENSCAFG00845005235	0	0	0	0
ENSCAFG00845007896	0	0	0	0
ENSCAFG00845020861	12	4	8	6
ENSCAFG00845005236	0	0	0	0
ENSCAFG00845007899	695	640	704	634
ENSCAFG00845030236	0	0	0	0
ENSCAFG00845005237	0	0	0	0
ENSCAFG00845007898	57	71	53	52
ENSCAFG00845030237	532	537	485	562
ENSCAFG00845005238	0	0	0	0
ENSCAFG00845030238	0	0	0	0

ENSCAFG00845005239	0	0	0	0
ENSCAFG00845030239	377	348	317	317
ENSCAFG00845019870	0	0	0	0
ENSCAFG00845019871	0	0	0	0
ENSCAFG00845030230	0	0	0	0
ENSCAFG00845017219	1785	1804	1714	1640
ENSCAFG00845019872	0	0	0	0
ENSCAFG00845030231	0	0	0	0
ENSCAFG00845017218	874	887	886	839
ENSCAFG00845019873	118	112	108	104
ENSCAFG00845030232	3	0	1	0
ENSCAFG00845017217	1353	1293	1086	1085
ENSCAFG00845019874	4	4	1	1
ENSCAFG00845030233	0	0	0	0
ENSCAFG00845017216	697	738	768	749
ENSCAFG00845019875	2	0	0	0
ENSCAFG00845030234	0	0	0	0
ENSCAFG00845017215	19	21	20	10
ENSCAFG00845019876	1399	1342	1406	1507
ENSCAFG00845030235	0	0	0	0
ENSCAFG00845017203	1106	979	755	833
ENSCAFG00845019866	2447	2300	2346	2353
ENSCAFG00845020855	0	0	0	0
ENSCAFG00845017202	628	534	652	644
ENSCAFG00845019867	7570	7263	7485	7438
ENSCAFG00845020856	573	610	507	509
ENSCAFG00845017201	2571	2373	2225	2195
ENSCAFG00845019868	0	0	0	0
ENSCAFG00845020853	3469	3324	4068	4138
ENSCAFG00845017200	0	0	0	0
ENSCAFG00845019869	0	0	0	0
ENSCAFG00845020854	1103	1116	1047	1097
ENSCAFG00845020859	4	4	9	3
ENSCAFG00845020857	1	1	0	0
ENSCAFG00845020858	607	568	532	561
ENSCAFG00845007880	0	0	0	0
ENSCAFG00845007882	0	1	0	2
ENSCAFG00845005220	2759	2662	2334	2439
ENSCAFG00845007881	0	0	0	0
ENSCAFG00845005221	0	0	0	0
ENSCAFG00845007884	0	0	0	0

ENSCAFG00845020851	0	0	0	0
ENSCAFG00845005222	0	0	0	0
ENSCAFG00845007883	0	1	0	0
ENSCAFG00845020852	2	5	4	1
ENSCAFG00845005223	0	0	0	0
ENSCAFG00845007886	12	12	10	14
ENSCAFG00845005224	13	11	13	9
ENSCAFG00845007885	41	46	43	21
ENSCAFG00845020850	0	0	0	0
ENSCAFG00845005225	0	1	5	3
ENSCAFG00845007888	0	0	0	0
ENSCAFG00845030247	0	0	0	0
ENSCAFG00845005226	1	3	2	0
ENSCAFG00845007887	281	270	295	306
ENSCAFG00845030248	0	0	0	0
ENSCAFG00845005227	10406	10527	12584	12626
ENSCAFG00845030249	0	0	0	0
ENSCAFG00845005228	0	0	0	0
ENSCAFG00845007889	0	0	0	0
ENSCAFG00845005229	0	0	1	1
ENSCAFG00845030240	222	233	211	224
ENSCAFG00845017209	4	3	5	3
ENSCAFG00845019860	0	0	0	0
ENSCAFG00845030241	3	2	2	2
ENSCAFG00845017208	4054	3726	3590	3732
ENSCAFG00845019861	0	0	0	0
ENSCAFG00845030242	131	123	124	116
ENSCAFG00845017207	1788	1859	1729	1727
ENSCAFG00845019862	4	1	0	0
ENSCAFG00845030243	8	9	7	8
ENSCAFG00845017206	3	5	2	4
ENSCAFG00845019863	0	0	0	0
ENSCAFG00845030244	5	7	1	4
ENSCAFG00845017205	3	2	6	2
ENSCAFG00845019864	4876	4587	4744	4941
ENSCAFG00845030245	2160	2053	2045	1989
ENSCAFG00845017204	3	0	2	10
ENSCAFG00845019865	0	0	0	0
ENSCAFG00845030246	7	6	4	6
ENSCAFG00845019855	0	0	0	0
ENSCAFG00845020844	28	28	35	28

ENSCAFG00845019856	0	0	0	0
ENSCAFG00845020845	5639	5623	5673	6025
ENSCAFG00845019857	0	0	0	0
ENSCAFG00845020842	2	1	3	0
ENSCAFG00845019858	1	0	0	0
ENSCAFG00845020843	0	0	0	0
ENSCAFG00845019859	0	1	0	1
ENSCAFG00845020848	129	123	109	139
ENSCAFG00845020849	0	0	0	0
ENSCAFG00845020846	0	0	0	0
ENSCAFG00845020847	133	129	135	116
ENSCAFG00845007871	341	388	386	418
ENSCAFG00845007870	2241	2271	2277	2419
ENSCAFG00845005210	0	0	0	0
ENSCAFG00845007873	0	0	0	0
ENSCAFG00845020840	0	0	0	0
ENSCAFG00845005211	2	1	3	1
ENSCAFG00845007872	0	0	0	1
ENSCAFG00845020841	22	33	24	29
ENSCAFG00845005212	0	0	0	0
ENSCAFG00845007875	0	0	0	0
ENSCAFG00845005213	0	0	0	2
ENSCAFG00845007874	19	30	28	22
ENSCAFG00845005214	0	0	0	0
ENSCAFG00845007877	863	808	808	782
ENSCAFG00845030258	109	150	129	97
ENSCAFG00845005215	0	0	0	0
ENSCAFG00845007876	306	326	306	288
ENSCAFG00845030259	5	0	5	9
ENSCAFG00845005216	1	4	1	5
ENSCAFG00845007879	1038	1078	1071	1066
ENSCAFG00845005217	0	0	0	0
ENSCAFG00845007878	0	0	0	0
ENSCAFG00845005218	649	661	670	660
ENSCAFG00845005219	0	0	0	0
ENSCAFG00845030250	0	0	0	0
ENSCAFG00845030251	7	9	6	3
ENSCAFG00845020839	15	8	7	9
ENSCAFG00845030252	0	1	1	0
ENSCAFG00845019850	0	0	0	0
ENSCAFG00845030253	1	3	2	5

ENSCAFG00845019851	7	6	7	14
ENSCAFG00845030254	4	1	3	0
ENSCAFG00845019852	0	0	0	0
ENSCAFG00845030255	252	205	144	155
ENSCAFG00845019853	0	0	0	0
ENSCAFG00845030256	0	0	0	0
ENSCAFG00845019854	0	0	0	0
ENSCAFG00845030257	0	0	0	1
ENSCAFG00845019844	0	0	0	0
ENSCAFG00845020833	0	0	0	2
ENSCAFG00845019845	0	0	0	0
ENSCAFG00845020834	1591	1572	1561	1535
ENSCAFG00845019846	444	549	524	476
ENSCAFG00845020831	5	3	4	4
ENSCAFG00845019847	0	0	0	0
ENSCAFG00845020832	0	0	0	0
ENSCAFG00845019848	944	984	878	876
ENSCAFG00845020837	0	0	0	0
ENSCAFG00845019849	2	3	0	0
ENSCAFG00845020838	360	414	419	409
ENSCAFG00845020835	38	24	48	45
ENSCAFG00845020836	0	0	0	0
ENSCAFG00845030260	0	3	1	1
ENSCAFG00845007860	0	0	0	0
ENSCAFG00845007862	1	0	1	0
ENSCAFG00845005200	0	0	0	0
ENSCAFG00845007861	637	626	528	502
ENSCAFG00845020830	17	17	12	16
ENSCAFG00845005201	1241	1149	1097	1134
ENSCAFG00845007864	35	57	39	54
ENSCAFG00845005202	0	0	0	1
ENSCAFG00845007863	17	19	8	19
ENSCAFG00845005203	440	415	476	491
ENSCAFG00845007866	0	0	0	0
ENSCAFG00845030269	0	0	2	0
ENSCAFG00845005204	1106	1139	998	1029
ENSCAFG00845007865	2605	2379	2486	2318
ENSCAFG00845005205	530	493	513	501
ENSCAFG00845007868	0	0	0	0
ENSCAFG00845005206	0	0	0	0
ENSCAFG00845007867	16	21	9	24

ENSCAFG00845005207	0	0	0	0
ENSCAFG00845005208	0	0	0	0
ENSCAFG00845007869	0	0	0	0
ENSCAFG00845005209	0	0	0	0
ENSCAFG00845030261	1027	1008	1186	1192
ENSCAFG00845030262	0	0	5	0
ENSCAFG00845020828	0	0	0	0
ENSCAFG00845030263	819	798	805	854
ENSCAFG00845020829	952	875	942	915
ENSCAFG00845030264	1622	1591	1613	1797
ENSCAFG00845019840	0	0	0	0
ENSCAFG00845030265	0	0	1	0
ENSCAFG00845019841	165	157	155	207
ENSCAFG00845030266	105	102	99	71
ENSCAFG00845019842	0	0	0	0
ENSCAFG00845030267	2300	2294	1979	1864
ENSCAFG00845019843	0	0	0	0
ENSCAFG00845030268	608	604	630	583
ENSCAFG00845019833	0	0	0	0
ENSCAFG00845020822	3713	3640	3574	3863
ENSCAFG00845019834	2	7	4	9
ENSCAFG00845020823	0	0	0	0
ENSCAFG00845019835	0	0	0	0
ENSCAFG00845020820	2	0	0	0
ENSCAFG00845019836	0	0	0	0
ENSCAFG00845020821	353	347	321	370
ENSCAFG00845019837	0	0	0	0
ENSCAFG00845020826	0	0	0	0
ENSCAFG00845019838	0	0	0	1
ENSCAFG00845020827	0	0	0	0
ENSCAFG00845019839	4	1	0	2
ENSCAFG00845020824	446	426	377	429
ENSCAFG00845030270	372	318	302	307
ENSCAFG00845020825	189	232	207	207
ENSCAFG00845030271	36	28	43	37
ENSCAFG00845007851	0	0	0	0
ENSCAFG00845007850	1051	1119	1016	984
ENSCAFG00845007853	0	0	0	1
ENSCAFG00845007852	0	0	0	0
ENSCAFG00845007855	0	0	0	0
ENSCAFG00845007854	0	0	0	0

ENSCAFG00845007857	0	0	0	0
ENSCAFG00845007856	23	23	24	51
ENSCAFG00845007859	0	0	0	0
ENSCAFG00845007858	5	3	1	5
ENSCAFG00845020819	5347	5412	5825	5832
ENSCAFG00845030272	0	0	0	0
ENSCAFG00845030273	113	118	115	118
ENSCAFG00845020817	0	0	0	1
ENSCAFG00845030274	1109	1125	1035	1049
ENSCAFG00845020818	182	189	186	198
ENSCAFG00845030275	0	0	0	1
ENSCAFG00845030276	589	519	514	566
ENSCAFG00845019830	0	0	0	0
ENSCAFG00845030277	1196	1088	1167	1119
ENSCAFG00845019831	0	2	2	1
ENSCAFG00845030278	396	406	409	380
ENSCAFG00845019832	4	9	15	6
ENSCAFG00845030279	2510	2372	2306	2292
ENSCAFG00845019822	23	27	23	18
ENSCAFG00845020811	1284	1265	1374	1397
ENSCAFG00845019823	856	903	1248	1314
ENSCAFG00845020812	0	0	0	0
ENSCAFG00845019824	0	0	0	0
ENSCAFG00845019825	0	0	0	0
ENSCAFG00845020810	732	768	684	718
ENSCAFG00845019826	0	0	0	0
ENSCAFG00845020815	357	352	383	383
ENSCAFG00845019827	0	0	0	0
ENSCAFG00845020816	3315	3316	2971	2967
ENSCAFG00845030280	0	0	0	0
ENSCAFG00845019828	102	126	116	83
ENSCAFG00845020813	15	13	22	18
ENSCAFG00845030281	1	0	2	2
ENSCAFG00845019829	6	4	2	1
ENSCAFG00845020814	0	0	0	0
ENSCAFG00845030282	460	441	423	369
ENSCAFG00845007840	0	0	0	0
ENSCAFG00845007842	0	0	0	3
ENSCAFG00845007841	0	0	0	0
ENSCAFG00845007844	13	7	7	11
ENSCAFG00845007843	1265	1207	1183	1322

ENSCAFG00845007846	45	42	20	20
ENSCAFG00845007845	2	5	5	1
ENSCAFG00845007848	2	0	0	1
ENSCAFG00845007847	0	0	0	0
ENSCAFG00845007849	0	0	0	0
ENSCAFG00845020808	720	664	697	700
ENSCAFG00845030283	0	0	0	0
ENSCAFG00845020809	0	0	1	2
ENSCAFG00845030284	3391	2974	3275	3283
ENSCAFG00845020806	0	0	0	0
ENSCAFG00845030285	5	10	0	1
ENSCAFG00845020807	4	0	0	6
ENSCAFG00845030286	429	408	378	354
ENSCAFG00845030287	505	484	521	605
ENSCAFG00845030288	680	617	527	651
ENSCAFG00845019820	277	277	263	260
ENSCAFG00845030289	0	0	0	0
ENSCAFG00845019821	2793	2761	2870	2851
ENSCAFG00845029298	0	0	1	0
ENSCAFG00845029299	0	0	0	0
ENSCAFG00845029296	1	0	1	0
ENSCAFG00845029297	445	483	521	509
ENSCAFG00845029294	0	0	0	0
ENSCAFG00845029295	0	0	0	0
ENSCAFG00845029292	0	0	0	0
ENSCAFG00845029293	0	0	0	0
ENSCAFG00845029290	1	0	1	1
ENSCAFG00845029291	0	1	0	1
ENSCAFG00845029289	7	10	13	16
ENSCAFG00845029287	199	208	179	169
ENSCAFG00845029288	1	0	0	0
ENSCAFG00845029285	2	2	3	1
ENSCAFG00845029286	0	0	0	0
ENSCAFG00845029283	6	5	2	3
ENSCAFG00845029284	0	0	0	0
ENSCAFG00845029281	1	2	0	0
ENSCAFG00845029282	35	46	47	39
ENSCAFG00845029280	0	0	0	1
ENSCAFG00845017299	2166	2018	1467	1600
ENSCAFG00845017298	1007	1007	988	1010
ENSCAFG00845017297	798	789	700	752

ENSCAFG00845017296	574	584	572	583
ENSCAFG00845017295	0	0	0	0
ENSCAFG00845017294	341	316	314	353
ENSCAFG00845017293	172	165	204	138
ENSCAFG00845017292	577	596	558	607
ENSCAFG00845017291	0	0	0	0
ENSCAFG00845017290	3	3	0	0
ENSCAFG00845029278	0	0	0	0
ENSCAFG00845029279	0	0	0	0
ENSCAFG00845029276	2352	2240	2052	2367
ENSCAFG00845029277	1	2	1	0
ENSCAFG00845029274	0	0	0	0
ENSCAFG00845029275	904	890	742	742
ENSCAFG00845029272	0	0	0	0
ENSCAFG00845029273	5	6	5	0
ENSCAFG00845029270	0	0	0	0
ENSCAFG00845029271	695	756	716	709
ENSCAFG00845017289	12	6	7	3
ENSCAFG00845017288	0	1	2	1
ENSCAFG00845017287	1888	1905	2035	2157
ENSCAFG00845017286	1935	1956	2080	2222
ENSCAFG00845017285	0	0	0	0
ENSCAFG00845017284	0	0	0	1
ENSCAFG00845017283	750	614	726	696
ENSCAFG00845017282	0	0	0	0
ENSCAFG00845017281	0	0	0	0
ENSCAFG00845017280	21	18	12	25
ENSCAFG00845029269	0	0	0	0
ENSCAFG00845029267	14	5	6	8
ENSCAFG00845029268	2140	1992	1997	2048
ENSCAFG00845029265	0	0	0	0
ENSCAFG00845029266	0	0	0	0
ENSCAFG00845029263	39	17	10	12
ENSCAFG00845029264	0	0	0	0
ENSCAFG00845029261	0	0	0	0
ENSCAFG00845029262	125	162	192	192
ENSCAFG00845029260	14	13	4	3
ENSCAFG00845017279	0	0	0	0
ENSCAFG00845017278	109	82	107	107
ENSCAFG00845017277	14	14	19	8
ENSCAFG00845005290	0	0	0	0

ENSCAFG00845017276	9	5	9	8
ENSCAFG00845005291	2403	2248	2147	2190
ENSCAFG00845017275	0	0	0	0
ENSCAFG00845005292	0	0	0	0
ENSCAFG00845017274	253	227	261	287
ENSCAFG00845005293	2582	2444	2410	2342
ENSCAFG00845017273	918	851	766	767
ENSCAFG00845005294	0	0	0	0
ENSCAFG00845005295	400	403	384	397
ENSCAFG00845017271	0	0	2	3
ENSCAFG00845005296	74	93	80	93
ENSCAFG00845017270	0	0	0	0
ENSCAFG00845005297	7	2	1	7
ENSCAFG00845005298	0	0	0	0
ENSCAFG00845005299	0	0	0	0
ENSCAFG00845029258	1	0	0	0
ENSCAFG00845029259	526	498	442	466
ENSCAFG00845029256	1997	1937	1864	1887
ENSCAFG00845029257	0	0	0	0
ENSCAFG00845029254	1	1	0	0
ENSCAFG00845029255	188	154	160	155
ENSCAFG00845029252	0	0	0	0
ENSCAFG00845029253	771	695	777	844
ENSCAFG00845029250	236	251	176	243
ENSCAFG00845029251	0	0	0	0
ENSCAFG00845017269	0	0	0	0
ENSCAFG00845017268	0	0	0	0
ENSCAFG00845017267	2	2	3	3
ENSCAFG00845017266	327	299	399	317
ENSCAFG00845017265	1433	1430	1572	1548
ENSCAFG00845005280	1	1	0	2
ENSCAFG00845017264	84	58	67	72
ENSCAFG00845005281	0	0	0	2
ENSCAFG00845017263	275	227	272	298
ENSCAFG00845005282	0	0	0	0
ENSCAFG00845017262	288	299	280	289
ENSCAFG00845005283	0	0	0	0
ENSCAFG00845017261	10	8	6	6
ENSCAFG00845005284	678	657	665	669
ENSCAFG00845017260	489	441	413	463
ENSCAFG00845005285	39	36	64	29

ENSCAFG00845005286	244	237	257	243
ENSCAFG00845005287	0	0	0	0
ENSCAFG00845005288	0	0	0	0
ENSCAFG00845005289	0	0	0	0
ENSCAFG00845029249	4	5	5	6
ENSCAFG00845029247	0	0	0	0
ENSCAFG00845029248	233	211	179	154
ENSCAFG00845029245	2	0	4	1
ENSCAFG00845029246	171	170	182	168
ENSCAFG00845029243	0	0	0	0
ENSCAFG00845029244	3872	3715	4037	4362
ENSCAFG00845029241	0	0	0	0
ENSCAFG00845029242	1709	1763	1661	1726
ENSCAFG00845029240	539	522	545	550
ENSCAFG00845017258	5	1	1	1
ENSCAFG00845017257	390	340	306	339
ENSCAFG00845017256	0	0	0	0
ENSCAFG00845017255	3	5	5	1
ENSCAFG00845017254	0	0	0	0
ENSCAFG00845017253	1035	1042	1027	995
ENSCAFG00845005270	0	0	0	0
ENSCAFG00845017252	283	337	339	330
ENSCAFG00845005271	39	25	43	45
ENSCAFG00845017251	1514	1398	1501	1587
ENSCAFG00845005272	32	32	19	15
ENSCAFG00845017250	0	0	0	0
ENSCAFG00845005273	0	1	2	1
ENSCAFG00845005274	0	0	0	0
ENSCAFG00845005275	36	52	79	48
ENSCAFG00845005276	470	479	462	435
ENSCAFG00845005277	375	378	417	407
ENSCAFG00845005278	0	0	0	0
ENSCAFG00845029238	423	438	371	372
ENSCAFG00845005279	0	0	3	0
ENSCAFG00845029239	213	212	315	289
ENSCAFG00845029236	4	9	5	5
ENSCAFG00845029237	30	25	28	31
ENSCAFG00845029234	2	3	5	1
ENSCAFG00845029235	20	17	13	17
ENSCAFG00845029232	854	809	865	935
ENSCAFG00845029233	635	589	1505	1628

ENSCAFG00845029230	1	0	5	1
ENSCAFG00845029231	21	29	17	27
ENSCAFG00845017259	7	8	6	5
ENSCAFG00845017247	10	6	5	8
ENSCAFG00845020899	0	0	0	0
ENSCAFG00845017246	0	0	0	0
ENSCAFG00845017245	261	243	244	232
ENSCAFG00845020897	290	287	308	302
ENSCAFG00845017244	1010	946	942	902
ENSCAFG00845020898	0	0	0	0
ENSCAFG00845017243	633	561	596	550
ENSCAFG00845017242	0	0	0	1
ENSCAFG00845017241	0	0	0	0
ENSCAFG00845005260	0	0	0	0
ENSCAFG00845017240	1409	1493	1305	1309
ENSCAFG00845005261	0	0	0	0
ENSCAFG00845020891	11	3	7	4
ENSCAFG00845005262	0	0	0	0
ENSCAFG00845020892	0	0	2	0
ENSCAFG00845005263	67	38	57	65
ENSCAFG00845005264	0	0	0	0
ENSCAFG00845020890	711	611	654	643
ENSCAFG00845005265	449	412	477	486
ENSCAFG00845020895	1	0	2	0
ENSCAFG00845029229	95	61	113	78
ENSCAFG00845005266	3	4	5	7
ENSCAFG00845020896	0	0	0	0
ENSCAFG00845005267	3	0	1	1
ENSCAFG00845020893	0	2	1	1
ENSCAFG00845029227	0	0	0	0
ENSCAFG00845005268	0	0	0	0
ENSCAFG00845020894	76	98	77	71
ENSCAFG00845029228	0	0	0	0
ENSCAFG00845005269	757	726	799	771
ENSCAFG00845029225	7020	6635	7559	7860
ENSCAFG00845030203	0	0	0	0
ENSCAFG00845029226	73	77	57	58
ENSCAFG00845030204	0	2	0	2
ENSCAFG00845029223	164	160	161	167
ENSCAFG00845030205	7	14	13	20
ENSCAFG00845029224	1	0	0	0

ENSCAFG00845030206	92	67	101	128
ENSCAFG00845029221	1481	1519	1380	1466
ENSCAFG00845030207	248	225	217	250
ENSCAFG00845029222	148	156	110	119
ENSCAFG00845030208	0	0	0	1
ENSCAFG00845030209	2	0	3	3
ENSCAFG00845029220	0	0	0	0
ENSCAFG00845030200	0	0	0	0
ENSCAFG00845017249	2	0	0	0
ENSCAFG00845030201	0	1	0	1
ENSCAFG00845017248	1123	1052	1182	1236
ENSCAFG00845030202	0	0	0	1
ENSCAFG00845029199	0	0	0	0
ENSCAFG00845029197	0	0	0	0
ENSCAFG00845029198	0	1	0	0
ENSCAFG00845029195	0	0	0	0
ENSCAFG00845029196	2952	2989	3152	3319
ENSCAFG00845029193	1	0	0	0
ENSCAFG00845029194	707	653	732	731
ENSCAFG00845029191	0	0	0	0
ENSCAFG00845029192	3	0	0	0
ENSCAFG00845029190	6	1	4	3
ENSCAFG00845029188	0	0	0	0
ENSCAFG00845029189	5	4	5	6
ENSCAFG00845029186	0	0	0	0
ENSCAFG00845029187	0	0	0	0
ENSCAFG00845029184	1265	1128	1169	1158
ENSCAFG00845029185	274	270	243	237
ENSCAFG00845029182	0	0	0	0
ENSCAFG00845029183	101	85	61	76
ENSCAFG00845029180	0	0	0	0
ENSCAFG00845029181	257	318	282	273
ENSCAFG00845019811	955	941	834	954
ENSCAFG00845020800	818	765	738	644
ENSCAFG00845019812	131	158	176	136
ENSCAFG00845020801	0	0	0	0
ENSCAFG00845019813	292	261	226	251
ENSCAFG00845019814	137	125	126	154
ENSCAFG00845019815	843	807	893	794
ENSCAFG00845020804	6	11	0	1
ENSCAFG00845019816	2482	2118	2300	2251

ENSCAFG00845020805	23	22	9	21
ENSCAFG00845030170	30	28	21	19
ENSCAFG00845019817	678	688	594	588
ENSCAFG00845020802	1	0	0	1
ENSCAFG00845030171	0	0	0	1
ENSCAFG00845019818	1402	1484	1499	1498
ENSCAFG00845020803	0	0	0	0
ENSCAFG00845030172	220	254	235	248
ENSCAFG00845019819	29085	28694	24696	25029
ENSCAFG00845007831	1	1	0	0
ENSCAFG00845007830	14	12	14	12
ENSCAFG00845007833	1639	1676	1625	1717
ENSCAFG00845007832	0	0	0	0
ENSCAFG00845007835	0	1	0	0
ENSCAFG00845007834	346	388	365	385
ENSCAFG00845007837	0	0	0	0
ENSCAFG00845007836	1412	1399	1330	1341
ENSCAFG00845007839	0	0	0	0
ENSCAFG00845007838	0	0	0	0
ENSCAFG00845030173	0	0	0	0
ENSCAFG00845030174	2158	2128	1960	1906
ENSCAFG00845030175	3	1	0	2
ENSCAFG00845030176	173	161	204	163
ENSCAFG00845030177	961	933	736	780
ENSCAFG00845030178	1081	1046	1143	1063
ENSCAFG00845030179	703	781	712	641
ENSCAFG00845019810	5	0	1	0
ENSCAFG00845019800	1334	1262	1095	1301
ENSCAFG00845019801	156	155	108	118
ENSCAFG00845019802	0	0	0	0
ENSCAFG00845019803	0	0	5	0
ENSCAFG00845019804	459	445	439	428
ENSCAFG00845030180	1136	1049	971	1108
ENSCAFG00845019805	0	0	0	0
ENSCAFG00845030181	4	4	2	1
ENSCAFG00845019806	0	0	0	0
ENSCAFG00845030182	4310	4264	4086	3983
ENSCAFG00845030183	463	446	476	458
ENSCAFG00845019808	591	601	549	606
ENSCAFG00845019809	0	0	0	0
ENSCAFG00845007820	169	147	171	165

ENSCAFG00845007822	0	0	0	0
ENSCAFG00845007821	3415	3326	3271	3539
ENSCAFG00845007824	3	3	3	3
ENSCAFG00845007823	0	0	0	0
ENSCAFG00845007826	21	7	14	15
ENSCAFG00845007825	1	5	0	1
ENSCAFG00845007828	688	687	555	592
ENSCAFG00845007827	0	0	0	0
ENSCAFG00845030184	0	0	0	0
ENSCAFG00845007829	0	0	0	0
ENSCAFG00845030185	47	37	28	23
ENSCAFG00845030186	266	241	254	223
ENSCAFG00845030187	265	265	265	250
ENSCAFG00845030188	2	2	9	4
ENSCAFG00845030189	1	1	5	3
ENSCAFG00845030190	0	0	0	0
ENSCAFG00845030191	0	0	0	0
ENSCAFG00845030192	0	0	0	0
ENSCAFG00845030193	32	29	25	40
ENSCAFG00845030194	2921	2854	2979	2939
ENSCAFG00845007811	360	354	368	345
ENSCAFG00845007810	0	0	0	0
ENSCAFG00845007813	0	2	0	0
ENSCAFG00845007812	18	19	16	22
ENSCAFG00845007815	2539	2419	2385	2482
ENSCAFG00845007814	0	0	0	0
ENSCAFG00845007817	346	322	348	323
ENSCAFG00845007816	23	29	15	13
ENSCAFG00845007819	305	301	300	293
ENSCAFG00845030195	3	0	2	0
ENSCAFG00845007818	127	150	88	99
ENSCAFG00845030196	4	4	5	0
ENSCAFG00845030197	306	288	291	340
ENSCAFG00845030198	0	0	0	0
ENSCAFG00845030199	89	87	87	76
ENSCAFG00845007800	14	21	8	9
ENSCAFG00845007802	112	103	125	99
ENSCAFG00845007801	0	0	0	0
ENSCAFG00845007804	322	321	311	329
ENSCAFG00845007803	643	671	593	555
ENSCAFG00845007806	0	0	0	0

ENSCAFG00845007805	0	0	0	0
ENSCAFG00845007808	0	1	0	0
ENSCAFG00845007807	0	0	0	0
ENSCAFG00845007809	261	247	210	187
ENSCAFG00845022704	0	6	1	0
ENSCAFG00845022703	6	9	11	17
ENSCAFG00845022702	3	9	11	4
ENSCAFG00845022701	0	1	0	4
ENSCAFG00845022708	2384	2220	2035	1923
ENSCAFG00845022707	0	0	0	0
ENSCAFG00845022706	1	0	0	2
ENSCAFG00845022705	0	0	0	0
ENSCAFG00845009730	861	879	813	762
ENSCAFG00845009733	1152	1135	1263	1236
ENSCAFG00845010719	76	68	40	25
ENSCAFG00845022700	0	0	0	0
ENSCAFG00845009734	0	0	0	0
ENSCAFG00845009731	0	0	0	0
ENSCAFG00845009732	0	0	0	0
ENSCAFG00845009737	10	2	4	7
ENSCAFG00845010726	2725	2633	2248	2296
ENSCAFG00845009738	0	0	0	0
ENSCAFG00845010727	2511	2347	2130	2201
ENSCAFG00845009735	1068	1103	983	909
ENSCAFG00845010728	0	0	0	0
ENSCAFG00845009736	0	0	0	0
ENSCAFG00845010729	1252	1134	1154	1195
ENSCAFG00845010722	586	608	686	643
ENSCAFG00845010723	1200	1146	866	883
ENSCAFG00845009739	0	0	0	0
ENSCAFG00845010724	5	11	16	8
ENSCAFG00845010725	0	0	0	0
ENSCAFG00845010720	402	351	292	329
ENSCAFG00845010721	79	74	77	78
ENSCAFG00845009722	0	0	0	0
ENSCAFG00845010708	1	0	0	0
ENSCAFG00845009723	61	53	55	65
ENSCAFG00845010709	2196	2239	2229	2320
ENSCAFG00845009720	0	1	0	0
ENSCAFG00845009721	0	0	0	0
ENSCAFG00845009726	0	0	0	0

ENSCAFG00845010715	0	0	0	0
ENSCAFG00845009727	2262	2170	2043	2118
ENSCAFG00845010716	261	288	223	250
ENSCAFG00845009724	0	0	0	0
ENSCAFG00845010717	51	50	49	63
ENSCAFG00845009725	1247	1203	1245	1267
ENSCAFG00845010718	318	306	329	313
ENSCAFG00845010711	1151	1046	1245	1240
ENSCAFG00845010712	0	0	0	0
ENSCAFG00845009728	403	431	394	407
ENSCAFG00845010713	3157	3043	3413	3511
ENSCAFG00845009729	4	2	4	5
ENSCAFG00845010714	581	558	583	566
ENSCAFG00845010710	34	35	32	60
ENSCAFG00845009711	252	280	222	233
ENSCAFG00845009712	444	510	479	484
ENSCAFG00845009710	0	0	0	0
ENSCAFG00845009715	0	0	0	0
ENSCAFG00845010704	25	24	16	12
ENSCAFG00845009716	86	76	69	88
ENSCAFG00845010705	9	7	15	8
ENSCAFG00845009713	0	0	0	0
ENSCAFG00845010706	7658	7574	7126	7134
ENSCAFG00845009714	0	0	0	0
ENSCAFG00845010707	4	1	2	1
ENSCAFG00845009719	1711	1618	1414	1529
ENSCAFG00845010700	0	0	0	0
ENSCAFG00845010701	2090	1918	1838	2044
ENSCAFG00845009717	14	9	6	13
ENSCAFG00845010702	17	1	3	9
ENSCAFG00845009718	2	0	0	0
ENSCAFG00845010703	0	0	0	0
ENSCAFG00845009700	0	0	0	0
ENSCAFG00845009701	726	706	708	713
ENSCAFG00845009704	0	0	0	0
ENSCAFG00845009705	0	0	0	0
ENSCAFG00845009702	339	360	342	349
ENSCAFG00845009703	528	615	499	603
ENSCAFG00845009708	258	247	189	204
ENSCAFG00845009709	569	588	536	530
ENSCAFG00845009706	643	626	659	610

ENSCAFG00845009707	0	0	0	0
ENSCAFG00845020129	0	0	1	0
ENSCAFG00845020127	0	0	0	0
ENSCAFG00845020128	275	231	222	254
ENSCAFG00845022789	108	104	106	155
ENSCAFG00845007150	0	0	0	0
ENSCAFG00845007152	5755	5397	5684	5664
ENSCAFG00845007151	37	29	14	17
ENSCAFG00845007154	0	0	0	0
ENSCAFG00845020121	1085	1085	1087	1063
ENSCAFG00845022784	1939	2099	1784	1692
ENSCAFG00845007153	39	36	42	35
ENSCAFG00845020122	0	0	0	0
ENSCAFG00845022783	44	57	61	42
ENSCAFG00845007156	1	0	1	0
ENSCAFG00845022782	4	4	6	5
ENSCAFG00845007155	0	1	0	3
ENSCAFG00845020120	3587	3515	3221	3129
ENSCAFG00845022781	1132	1109	1127	1183
ENSCAFG00845007158	89	112	113	120
ENSCAFG00845020125	1153	1094	1070	1081
ENSCAFG00845022788	0	0	0	0
ENSCAFG00845007157	0	0	0	0
ENSCAFG00845020126	4	1	3	4
ENSCAFG00845020123	1719	1612	1602	1669
ENSCAFG00845022786	1	3	5	0
ENSCAFG00845007159	0	0	0	0
ENSCAFG00845020124	401	356	350	378
ENSCAFG00845022785	381	379	347	439
ENSCAFG00845022780	3295	3239	3383	3341
ENSCAFG00845019130	0	0	0	0
ENSCAFG00845019131	7	0	3	2
ENSCAFG00845019132	0	0	2	2
ENSCAFG00845019133	0	0	0	0
ENSCAFG00845019134	0	1	0	0
ENSCAFG00845019135	2076	2062	1785	1809
ENSCAFG00845019136	1	4	1	2
ENSCAFG00845019137	0	0	0	0
ENSCAFG00845019139	0	0	0	0
ENSCAFG00845019129	0	0	0	0
ENSCAFG00845020118	0	0	0	0

ENSCAFG00845020119	42	41	40	38
ENSCAFG00845020116	2680	2718	2900	2841
ENSCAFG00845010790	133	114	132	159
ENSCAFG00845020117	0	0	0	0
ENSCAFG00845022778	0	2	0	0
ENSCAFG00845007141	0	0	0	0
ENSCAFG00845007140	5	3	6	7
ENSCAFG00845007143	211	234	256	234
ENSCAFG00845020110	0	0	0	0
ENSCAFG00845022773	594	530	610	642
ENSCAFG00845007142	16	15	11	18
ENSCAFG00845020111	0	0	0	0
ENSCAFG00845022772	1	0	0	1
ENSCAFG00845007145	397	372	327	352
ENSCAFG00845022771	0	0	0	0
ENSCAFG00845007144	4	8	17	5
ENSCAFG00845022770	561	585	480	522
ENSCAFG00845007147	0	0	0	0
ENSCAFG00845020114	5	3	4	5
ENSCAFG00845007146	265	227	314	341
ENSCAFG00845020115	0	0	1	0
ENSCAFG00845022776	467	436	501	451
ENSCAFG00845007149	826	700	633	619
ENSCAFG00845020112	539	493	537	526
ENSCAFG00845022775	0	0	0	0
ENSCAFG00845007148	0	0	0	0
ENSCAFG00845020113	0	0	0	0
ENSCAFG00845022774	0	0	0	1
ENSCAFG00845010799	1	3	1	3
ENSCAFG00845019120	1	1	0	0
ENSCAFG00845010795	1	0	0	0
ENSCAFG00845019121	0	0	0	0
ENSCAFG00845010796	0	0	0	0
ENSCAFG00845019122	0	0	0	0
ENSCAFG00845010797	2997	2736	2434	2628
ENSCAFG00845019123	0	0	0	0
ENSCAFG00845010798	0	0	0	0
ENSCAFG00845019124	0	0	0	0
ENSCAFG00845010791	0	0	2	0
ENSCAFG00845019125	0	0	0	0
ENSCAFG00845010792	5737	5946	6347	6421

ENSCAFG00845019126	0	0	0	0
ENSCAFG00845010793	354	375	340	353
ENSCAFG00845019127	0	0	0	0
ENSCAFG00845010794	0	0	0	0
ENSCAFG00845019128	0	0	0	0
ENSCAFG00845019118	487	456	494	497
ENSCAFG00845020107	0	0	0	1
ENSCAFG00845019119	0	0	0	0
ENSCAFG00845020108	19	10	11	6
ENSCAFG00845022769	2	1	1	2
ENSCAFG00845020105	0	0	0	0
ENSCAFG00845022768	1387	1366	1230	1334
ENSCAFG00845020106	0	0	0	0
ENSCAFG00845022767	199	193	201	225
ENSCAFG00845009791	1	0	0	0
ENSCAFG00845009792	0	0	0	0
ENSCAFG00845007130	109	87	122	112
ENSCAFG00845020109	0	2	1	2
ENSCAFG00845009790	2145	2012	2033	2106
ENSCAFG00845007132	62	54	83	71
ENSCAFG00845009795	0	0	0	0
ENSCAFG00845022762	584	583	592	649
ENSCAFG00845007131	0	0	0	0
ENSCAFG00845009796	0	0	0	0
ENSCAFG00845020100	0	0	0	0
ENSCAFG00845022761	219	268	192	222
ENSCAFG00845007134	0	0	0	0
ENSCAFG00845009793	85	87	106	105
ENSCAFG00845022760	9	7	10	13
ENSCAFG00845007133	0	0	0	0
ENSCAFG00845009794	3	1	3	2
ENSCAFG00845007136	149	148	158	139
ENSCAFG00845009799	209	181	172	205
ENSCAFG00845020103	0	0	0	0
ENSCAFG00845022766	914	848	888	919
ENSCAFG00845007135	0	0	1	0
ENSCAFG00845020104	30	39	25	30
ENSCAFG00845022765	1887	1909	1934	1900
ENSCAFG00845007138	218	239	212	242
ENSCAFG00845009797	2	0	2	4
ENSCAFG00845020101	357	328	302	307

ENSCAFG00845007137	8	11	8	6
ENSCAFG00845009798	149	142	152	115
ENSCAFG00845020102	0	0	0	0
ENSCAFG00845022763	15911	15133	13791	14174
ENSCAFG00845007139	3262	3235	3001	2891
ENSCAFG00845010788	0	0	0	0
ENSCAFG00845010789	5766	5545	4933	4894
ENSCAFG00845010784	112	101	101	129
ENSCAFG00845019110	0	0	0	0
ENSCAFG00845010785	0	0	0	0
ENSCAFG00845019111	0	0	0	0
ENSCAFG00845010786	545	464	509	495
ENSCAFG00845010787	0	1	6	5
ENSCAFG00845019113	344	313	374	407
ENSCAFG00845010780	0	0	0	0
ENSCAFG00845019114	0	2	0	0
ENSCAFG00845010781	1	0	1	0
ENSCAFG00845019115	648	585	518	605
ENSCAFG00845010782	403	390	437	464
ENSCAFG00845019116	0	0	0	0
ENSCAFG00845010783	0	0	2	1
ENSCAFG00845019117	39	36	37	25
ENSCAFG00845019107	0	0	0	0
ENSCAFG00845022759	0	0	0	0
ENSCAFG00845019108	1987	1985	1885	1955
ENSCAFG00845022758	0	0	0	0
ENSCAFG00845019109	0	0	0	0
ENSCAFG00845022757	0	2	4	4
ENSCAFG00845022756	708	770	901	802
ENSCAFG00845009780	12438	12087	11991	12072
ENSCAFG00845009781	0	2	0	0
ENSCAFG00845007121	270	314	262	274
ENSCAFG00845009784	5	1	0	1
ENSCAFG00845022751	0	0	0	0
ENSCAFG00845007120	464	439	227	287
ENSCAFG00845009785	0	1	0	1
ENSCAFG00845007123	0	0	0	1
ENSCAFG00845009782	0	0	0	0
ENSCAFG00845007122	0	0	0	0
ENSCAFG00845009783	28	45	24	26
ENSCAFG00845007125	231	209	225	213

ENSCAFG00845009788	1	0	2	0
ENSCAFG00845022755	0	0	0	0
ENSCAFG00845007124	0	0	0	0
ENSCAFG00845009789	0	0	0	0
ENSCAFG00845022754	0	0	0	0
ENSCAFG00845007127	0	3	0	8
ENSCAFG00845009786	0	2	1	1
ENSCAFG00845022753	561	522	510	560
ENSCAFG00845007126	0	0	0	0
ENSCAFG00845009787	0	0	0	0
ENSCAFG00845022752	593	455	471	490
ENSCAFG00845007129	0	0	0	0
ENSCAFG00845007128	0	0	0	0
ENSCAFG00845010777	289	262	229	256
ENSCAFG00845010778	587	491	523	540
ENSCAFG00845010779	0	0	0	0
ENSCAFG00845010773	6	6	9	5
ENSCAFG00845010774	5189	4831	4786	4825
ENSCAFG00845019100	0	0	0	0
ENSCAFG00845010775	1435	1339	1454	1419
ENSCAFG00845019101	2	0	2	3
ENSCAFG00845010776	802	733	799	791
ENSCAFG00845019102	347	366	338	357
ENSCAFG00845019103	1654	1668	1639	1537
ENSCAFG00845010770	0	0	0	0
ENSCAFG00845019104	1	0	1	1
ENSCAFG00845010771	73	46	68	95
ENSCAFG00845019105	0	0	0	1
ENSCAFG00845010772	95	70	29	31
ENSCAFG00845019106	0	0	0	0
ENSCAFG00845022748	0	0	0	0
ENSCAFG00845022747	79	69	96	91
ENSCAFG00845022746	0	0	0	0
ENSCAFG00845022745	3510	3378	3071	3203
ENSCAFG00845009770	358	354	368	323
ENSCAFG00845022749	315	340	518	517
ENSCAFG00845007110	3	1	7	2
ENSCAFG00845009773	0	0	0	0
ENSCAFG00845022740	0	0	0	0
ENSCAFG00845009774	0	0	0	0
ENSCAFG00845007112	13	21	12	17

ENSCAFG00845009771	0	0	0	0
ENSCAFG00845007111	0	0	0	0
ENSCAFG00845009772	8	2	2	5
ENSCAFG00845007114	0	0	0	0
ENSCAFG00845009777	1232	1156	1111	1135
ENSCAFG00845022744	370	308	355	408
ENSCAFG00845007113	0	1	0	0
ENSCAFG00845009778	361	378	386	407
ENSCAFG00845022743	62	75	83	73
ENSCAFG00845007116	0	0	0	0
ENSCAFG00845009775	334	336	344	317
ENSCAFG00845022742	1	0	0	0
ENSCAFG00845007115	375	251	304	373
ENSCAFG00845009776	382	360	350	348
ENSCAFG00845022741	0	0	0	0
ENSCAFG00845007118	0	0	0	0
ENSCAFG00845007117	0	0	0	0
ENSCAFG00845009779	120	142	126	113
ENSCAFG00845007119	16	14	14	13
ENSCAFG00845010766	0	0	0	0
ENSCAFG00845010767	0	0	0	0
ENSCAFG00845010768	76	77	88	87
ENSCAFG00845010769	0	0	0	0
ENSCAFG00845010762	141	99	143	153
ENSCAFG00845010763	1	4	1	8
ENSCAFG00845010764	0	0	1	1
ENSCAFG00845010765	0	0	0	0
ENSCAFG00845010760	0	0	0	0
ENSCAFG00845010761	384	300	430	394
ENSCAFG00845022737	0	0	0	0
ENSCAFG00845022736	1	0	0	0
ENSCAFG00845022735	3	3	3	7
ENSCAFG00845022734	899	850	904	914
ENSCAFG00845022738	2	1	4	1
ENSCAFG00845009762	17	13	14	12
ENSCAFG00845009763	0	0	0	0
ENSCAFG00845007101	0	0	0	0
ENSCAFG00845009760	114	87	99	108
ENSCAFG00845007100	0	0	0	0
ENSCAFG00845009761	299	273	268	292
ENSCAFG00845007103	172	163	145	173

ENSCAFG00845009766	402	389	407	395
ENSCAFG00845022733	2	0	2	2
ENSCAFG00845007102	96	86	73	57
ENSCAFG00845009767	761	730	705	715
ENSCAFG00845022732	321	322	267	248
ENSCAFG00845007105	3	5	0	1
ENSCAFG00845009764	2	0	3	0
ENSCAFG00845022731	0	0	0	0
ENSCAFG00845007104	140	120	120	137
ENSCAFG00845009765	1	0	0	0
ENSCAFG00845007107	187	216	179	226
ENSCAFG00845010759	5	5	1	5
ENSCAFG00845007106	11	8	1	6
ENSCAFG00845007109	579	512	517	556
ENSCAFG00845009768	776	764	656	663
ENSCAFG00845007108	17	12	16	17
ENSCAFG00845009769	1168	1044	958	858
ENSCAFG00845010755	1	2	5	2
ENSCAFG00845010756	2	0	1	0
ENSCAFG00845010757	160	149	156	154
ENSCAFG00845010758	1371	1376	1325	1348
ENSCAFG00845010751	583	528	499	535
ENSCAFG00845010752	0	0	0	0
ENSCAFG00845010753	209	185	178	157
ENSCAFG00845010754	0	0	0	0
ENSCAFG00845010750	0	0	0	0
ENSCAFG00845022726	38	29	73	59
ENSCAFG00845022725	1405	1467	1414	1491
ENSCAFG00845022724	0	0	0	0
ENSCAFG00845022723	0	0	0	0
ENSCAFG00845022729	492	446	430	527
ENSCAFG00845022728	0	0	0	0
ENSCAFG00845022727	176	164	164	145
ENSCAFG00845009751	41	59	47	38
ENSCAFG00845009752	0	0	0	0
ENSCAFG00845009750	26090	24908	24310	24435
ENSCAFG00845009755	0	0	0	0
ENSCAFG00845022722	961	1077	1039	1216
ENSCAFG00845009756	2998	3022	2999	2785
ENSCAFG00845022721	1	0	1	1
ENSCAFG00845009753	241	255	212	239

ENSCAFG00845022720	4	0	1	6
ENSCAFG00845009754	0	0	0	0
ENSCAFG00845009759	451	448	430	369
ENSCAFG00845010748	77	80	92	72
ENSCAFG00845010749	1	0	0	0
ENSCAFG00845009757	42	49	46	38
ENSCAFG00845009758	0	0	0	0
ENSCAFG00845010744	1742	1549	1538	1549
ENSCAFG00845010745	627	596	516	570
ENSCAFG00845010746	0	0	0	0
ENSCAFG00845010747	3	3	1	2
ENSCAFG00845010740	0	0	0	2
ENSCAFG00845010741	2	0	1	0
ENSCAFG00845010742	39	34	24	30
ENSCAFG00845010743	0	0	0	2
ENSCAFG00845022715	0	0	0	0
ENSCAFG00845022713	1175	1042	1010	1072
ENSCAFG00845022712	3	2	0	1
ENSCAFG00845022719	9	4	6	2
ENSCAFG00845022718	0	0	0	0
ENSCAFG00845022717	0	0	0	0
ENSCAFG00845022716	145	143	143	123
ENSCAFG00845009740	2661	2329	2594	2704
ENSCAFG00845009741	2224	2299	2131	2122
ENSCAFG00845009744	1312	1315	1307	1441
ENSCAFG00845022711	387	338	328	335
ENSCAFG00845009745	71	60	54	66
ENSCAFG00845009742	295	296	324	334
ENSCAFG00845009743	0	0	0	0
ENSCAFG00845009748	0	0	0	0
ENSCAFG00845010737	984	928	853	905
ENSCAFG00845009749	0	0	0	0
ENSCAFG00845010738	1432	1389	1508	1506
ENSCAFG00845009746	5	13	10	7
ENSCAFG00845010739	1	1	4	0
ENSCAFG00845009747	34	27	27	32
ENSCAFG00845010733	1942	1794	1820	1860
ENSCAFG00845010734	1	0	2	1
ENSCAFG00845010735	29	8	18	22
ENSCAFG00845010736	11	6	10	7
ENSCAFG00845010730	132	96	120	103

ENSCAFG00845010731	1844	1790	1867	1920
ENSCAFG00845010732	1833	1628	1573	1669
ENSCAFG00845022709	112	107	79	128
ENSCAFG00845020088	789	680	780	790
ENSCAFG00845020089	0	0	0	0
ENSCAFG00845020086	291	249	227	238
ENSCAFG00845020087	102	84	102	78
ENSCAFG00845019091	0	0	0	0
ENSCAFG00845020080	0	0	0	0
ENSCAFG00845019092	1	3	1	1
ENSCAFG00845020081	0	0	0	0
ENSCAFG00845019093	206	176	186	185
ENSCAFG00845019094	3	0	0	2
ENSCAFG00845019095	354	299	315	272
ENSCAFG00845020084	225	168	187	172
ENSCAFG00845019096	1184	1023	1117	1123
ENSCAFG00845020085	898	872	888	906
ENSCAFG00845019097	0	0	2	0
ENSCAFG00845020082	0	0	0	0
ENSCAFG00845019098	2	3	2	1
ENSCAFG00845020083	1090	1110	1126	1175
ENSCAFG00845019099	0	0	0	1
ENSCAFG00845020077	36	55	75	47
ENSCAFG00845020078	106	112	89	96
ENSCAFG00845020075	0	3	5	4
ENSCAFG00845020076	0	0	0	0
ENSCAFG00845020079	517	446	504	547
ENSCAFG00845019090	0	0	0	0
ENSCAFG00845019080	1007	941	1089	960
ENSCAFG00845019081	0	0	0	0
ENSCAFG00845020070	487	420	457	508
ENSCAFG00845019082	9	12	30	15
ENSCAFG00845019083	84	62	70	65
ENSCAFG00845020073	2	1	4	1
ENSCAFG00845019085	0	0	0	0
ENSCAFG00845020074	792	762	713	668
ENSCAFG00845019086	23	15	11	18
ENSCAFG00845020071	4058	3927	3753	3659
ENSCAFG00845019087	0	0	0	0
ENSCAFG00845020072	209	224	212	199
ENSCAFG00845019088	0	0	0	0

ENSCAFG00845019089	0	0	0	0
ENSCAFG00845007091	328	302	222	247
ENSCAFG00845007090	12	26	10	18
ENSCAFG00845007093	0	0	0	0
ENSCAFG00845007092	2633	2657	2683	2773
ENSCAFG00845007095	4	1	0	2
ENSCAFG00845007094	653	609	590	645
ENSCAFG00845007097	0	0	0	0
ENSCAFG00845007096	0	0	0	0
ENSCAFG00845007099	0	0	0	0
ENSCAFG00845020066	0	0	0	0
ENSCAFG00845007098	0	0	0	0
ENSCAFG00845020067	0	0	0	0
ENSCAFG00845020064	809	801	879	917
ENSCAFG00845020065	5	6	1	0
ENSCAFG00845020068	70	75	72	94
ENSCAFG00845020069	1906	1788	1646	1652
ENSCAFG00845019070	1725	1606	1650	1732
ENSCAFG00845019071	357	363	296	329
ENSCAFG00845019072	57	42	31	37
ENSCAFG00845019073	0	0	0	0
ENSCAFG00845020062	0	0	0	0
ENSCAFG00845019074	0	0	0	0
ENSCAFG00845020063	12	11	4	13
ENSCAFG00845019075	0	0	0	0
ENSCAFG00845020060	0	1	0	0
ENSCAFG00845019076	264	242	226	254
ENSCAFG00845020061	806	756	738	743
ENSCAFG00845019077	1	2	6	7
ENSCAFG00845019078	0	0	0	0
ENSCAFG00845019079	7	5	8	10
ENSCAFG00845007080	0	0	0	0
ENSCAFG00845007082	0	0	0	0
ENSCAFG00845007081	0	0	0	0
ENSCAFG00845007084	0	0	0	0
ENSCAFG00845007083	177	238	166	173
ENSCAFG00845007086	144	151	135	154
ENSCAFG00845007085	0	0	0	0
ENSCAFG00845007088	0	0	0	0
ENSCAFG00845020055	0	0	0	0
ENSCAFG00845007087	0	0	0	0

ENSCAFG00845020056	26	16	30	25
ENSCAFG00845020053	13	6	11	5
ENSCAFG00845007089	0	0	0	0
ENSCAFG00845020054	0	0	0	0
ENSCAFG00845020059	337	352	370	372
ENSCAFG00845020057	1166	1070	1042	1079
ENSCAFG00845020058	1279	1228	1322	1351
ENSCAFG00845019060	1144	1122	1050	985
ENSCAFG00845019061	59	55	64	61
ENSCAFG00845019062	1	0	0	0
ENSCAFG00845020051	1	0	0	3
ENSCAFG00845019063	0	0	0	0
ENSCAFG00845020052	0	0	0	0
ENSCAFG00845019064	0	0	0	0
ENSCAFG00845019065	0	0	0	0
ENSCAFG00845020050	341	329	376	344
ENSCAFG00845019066	0	1	3	1
ENSCAFG00845019067	0	0	0	0
ENSCAFG00845019068	6	21	11	13
ENSCAFG00845019069	0	0	0	0
ENSCAFG00845007071	764	770	697	732
ENSCAFG00845007070	0	0	0	0
ENSCAFG00845007073	1	0	0	0
ENSCAFG00845007072	0	0	0	0
ENSCAFG00845007075	13	14	15	12
ENSCAFG00845007074	0	0	0	0
ENSCAFG00845007077	214	188	219	154
ENSCAFG00845020044	0	0	0	0
ENSCAFG00845007076	212	184	171	163
ENSCAFG00845020045	0	0	0	0
ENSCAFG00845007079	22	20	12	23
ENSCAFG00845020042	0	0	0	0
ENSCAFG00845007078	3	2	4	6
ENSCAFG00845020043	107	87	81	105
ENSCAFG00845020048	0	0	0	0
ENSCAFG00845020049	0	0	0	0
ENSCAFG00845020046	6	0	4	4
ENSCAFG00845020047	0	0	0	0
ENSCAFG00845019051	0	0	0	0
ENSCAFG00845020040	0	0	0	0
ENSCAFG00845019052	0	0	0	0

ENSCAFG00845020041	0	0	0	0
ENSCAFG00845019053	0	0	0	0
ENSCAFG00845019054	2	3	2	2
ENSCAFG00845019055	4	1	4	4
ENSCAFG00845019056	0	0	0	0
ENSCAFG00845019057	37	35	20	29
ENSCAFG00845019058	2	3	0	1
ENSCAFG00845019059	0	0	0	0
ENSCAFG00845007060	1678	1573	1573	1487
ENSCAFG00845020039	386	397	392	436
ENSCAFG00845007062	0	0	0	0
ENSCAFG00845007061	44	28	33	43
ENSCAFG00845007064	353	400	357	376
ENSCAFG00845007063	44	47	66	45
ENSCAFG00845007066	7	0	5	6
ENSCAFG00845020033	0	0	0	0
ENSCAFG00845022696	991	983	819	829
ENSCAFG00845007065	0	0	0	0
ENSCAFG00845020034	0	0	0	0
ENSCAFG00845022695	0	1	0	0
ENSCAFG00845007068	0	0	0	0
ENSCAFG00845020031	259	238	255	265
ENSCAFG00845007067	0	0	0	0
ENSCAFG00845020032	0	0	0	0
ENSCAFG00845022693	0	0	0	0
ENSCAFG00845020037	0	0	0	0
ENSCAFG00845007069	0	0	0	0
ENSCAFG00845020038	0	0	0	0
ENSCAFG00845022699	126	131	121	116
ENSCAFG00845020035	0	0	0	0
ENSCAFG00845022698	4	2	3	7
ENSCAFG00845020036	120	140	125	163
ENSCAFG00845022697	0	0	0	0
ENSCAFG00845019040	0	0	0	0
ENSCAFG00845022692	1076	1070	1089	1057
ENSCAFG00845019041	0	0	0	0
ENSCAFG00845020030	0	0	0	0
ENSCAFG00845022691	0	0	0	0
ENSCAFG00845019042	27	36	24	49
ENSCAFG00845019043	1805	1682	1941	1796
ENSCAFG00845019044	367	378	378	363

ENSCAFG00845019045	0	0	0	0
ENSCAFG00845019046	4	6	9	12
ENSCAFG00845019047	6941	6658	6441	6359
ENSCAFG00845019048	0	0	0	1
ENSCAFG00845019049	3	4	1	0
ENSCAFG00845020028	1	0	0	0
ENSCAFG00845020029	6	2	1	1
ENSCAFG00845007051	4	2	3	5
ENSCAFG00845007050	394	397	299	312
ENSCAFG00845007053	1635	1496	1525	1584
ENSCAFG00845007052	0	0	0	0
ENSCAFG00845007055	0	0	0	0
ENSCAFG00845020022	2049	1955	2156	2186
ENSCAFG00845022685	0	0	0	0
ENSCAFG00845007054	0	0	0	0
ENSCAFG00845020023	0	3	2	0
ENSCAFG00845022684	0	0	0	0
ENSCAFG00845007057	0	0	0	0
ENSCAFG00845020020	0	0	0	0
ENSCAFG00845022683	4	11	8	3
ENSCAFG00845007056	118	95	77	93
ENSCAFG00845020021	0	0	0	0
ENSCAFG00845022682	16	19	15	14
ENSCAFG00845007059	0	0	0	0
ENSCAFG00845020026	8	3	7	5
ENSCAFG00845022689	0	0	0	0
ENSCAFG00845007058	0	0	0	0
ENSCAFG00845020027	0	0	0	0
ENSCAFG00845022688	0	0	0	0
ENSCAFG00845020024	0	0	0	0
ENSCAFG00845022687	740	750	719	696
ENSCAFG00845020025	6	10	10	7
ENSCAFG00845022686	189	179	170	195
ENSCAFG00845022681	334	380	337	409
ENSCAFG00845019030	0	0	0	0
ENSCAFG00845022680	170	125	159	133
ENSCAFG00845019031	878	845	793	815
ENSCAFG00845019032	0	8	0	4
ENSCAFG00845019033	1068	1027	1050	1069
ENSCAFG00845019034	0	0	0	0
ENSCAFG00845019035	0	0	0	0

ENSCAFG00845019036	0	0	0	0
ENSCAFG00845019037	0	0	0	0
ENSCAFG00845019038	4542	4529	4111	4388
ENSCAFG00845019039	0	0	0	0
ENSCAFG00845020019	0	0	0	0
ENSCAFG00845010690	90	65	67	75
ENSCAFG00845020017	0	0	0	0
ENSCAFG00845010691	1561	1487	1613	1710
ENSCAFG00845020018	0	0	0	0
ENSCAFG00845022679	925	998	938	921
ENSCAFG00845007040	0	0	0	0
ENSCAFG00845007042	0	0	0	0
ENSCAFG00845007041	823	710	852	744
ENSCAFG00845007044	1477	1343	1401	1420
ENSCAFG00845020011	0	0	0	0
ENSCAFG00845022674	0	0	0	0
ENSCAFG00845007043	2	3	5	6
ENSCAFG00845020012	0	0	0	0
ENSCAFG00845007046	1277	1273	1138	1092
ENSCAFG00845022672	4	0	5	0
ENSCAFG00845007045	2409	2475	2128	2295
ENSCAFG00845020010	0	0	1	0
ENSCAFG00845022671	0	0	0	0
ENSCAFG00845007048	575	578	624	605
ENSCAFG00845020015	153	154	154	127
ENSCAFG00845022678	1163	1031	1121	988
ENSCAFG00845007047	0	0	0	0
ENSCAFG00845020016	0	0	0	0
ENSCAFG00845022677	0	0	0	0
ENSCAFG00845020013	6038	5568	6494	6432
ENSCAFG00845022676	0	0	0	0
ENSCAFG00845007049	14	2	10	3
ENSCAFG00845020014	0	0	0	0
ENSCAFG00845022675	285	284	276	288
ENSCAFG00845022670	15	8	10	20
ENSCAFG00845019020	0	0	0	0
ENSCAFG00845019021	514	577	525	581
ENSCAFG00845010696	867	862	991	1045
ENSCAFG00845019022	1474	1381	1317	1315
ENSCAFG00845010697	921	850	929	863
ENSCAFG00845019023	0	0	0	0

ENSCAFG00845010698	4	1	0	1
ENSCAFG00845019024	805	784	817	839
ENSCAFG00845010699	1	1	4	0
ENSCAFG00845019025	0	0	0	0
ENSCAFG00845010692	0	0	0	1
ENSCAFG00845019026	1998	1943	1960	1977
ENSCAFG00845010693	1	3	2	0
ENSCAFG00845019027	0	0	0	0
ENSCAFG00845010694	30	21	24	14
ENSCAFG00845019028	19	15	13	11
ENSCAFG00845010695	1902	2058	1955	2031
ENSCAFG00845019029	3	0	0	0
ENSCAFG00845020099	0	0	0	0
ENSCAFG00845020097	768	781	775	744
ENSCAFG00845020098	1486	1400	2905	3055
ENSCAFG00845020091	0	0	0	0
ENSCAFG00845020092	0	0	0	0
ENSCAFG00845020090	8	5	4	4
ENSCAFG00845020095	1415	1474	1372	1253
ENSCAFG00845020096	436	427	468	375
ENSCAFG00845020093	0	0	1	0
ENSCAFG00845020094	0	0	0	1
ENSCAFG00845022825	8	1	3	5
ENSCAFG00845022824	912	758	874	847
ENSCAFG00845022822	0	0	0	0
ENSCAFG00845022829	2955	2865	3421	3337
ENSCAFG00845022828	18	20	7	16
ENSCAFG00845022827	46	37	41	48
ENSCAFG00845009850	4	3	10	8
ENSCAFG00845009851	0	0	0	1
ENSCAFG00845009854	0	0	0	0
ENSCAFG00845022821	1190	1287	1103	1148
ENSCAFG00845009855	2	0	1	1
ENSCAFG00845022820	136	132	130	158
ENSCAFG00845009852	0	0	0	0
ENSCAFG00845009853	0	0	0	0
ENSCAFG00845009858	0	1	0	0
ENSCAFG00845010847	730	698	582	718
ENSCAFG00845009859	32	21	21	25
ENSCAFG00845010848	0	0	0	1
ENSCAFG00845009856	0	0	0	1

ENSCAFG00845010849	0	0	0	0
ENSCAFG00845009857	0	0	0	0
ENSCAFG00845010843	0	0	0	0
ENSCAFG00845010844	0	0	0	0
ENSCAFG00845010845	1743	1612	1697	1581
ENSCAFG00845010846	6	0	0	4
ENSCAFG00845010840	459	395	449	450
ENSCAFG00845010841	0	0	0	0
ENSCAFG00845010842	0	0	0	0
ENSCAFG00845022819	0	0	0	0
ENSCAFG00845022814	372	329	337	359
ENSCAFG00845022813	0	0	0	0
ENSCAFG00845022812	0	0	0	0
ENSCAFG00845022811	146	152	154	162
ENSCAFG00845022818	454	412	418	423
ENSCAFG00845022817	0	1	0	1
ENSCAFG00845022816	0	0	0	4
ENSCAFG00845022815	3	2	4	5
ENSCAFG00845009840	0	0	3	1
ENSCAFG00845009843	0	0	0	0
ENSCAFG00845010829	6	7	1	0
ENSCAFG00845022810	0	0	0	0
ENSCAFG00845009844	7	2	6	4
ENSCAFG00845009841	120	141	158	179
ENSCAFG00845009842	635	645	563	596
ENSCAFG00845009847	271	273	257	265
ENSCAFG00845010836	252	311	328	282
ENSCAFG00845009848	350	355	417	415
ENSCAFG00845010837	1586	1524	1652	1571
ENSCAFG00845009845	1049	992	1113	1147
ENSCAFG00845010838	6498	6301	6306	6398
ENSCAFG00845009846	768	745	779	781
ENSCAFG00845010839	0	0	2	0
ENSCAFG00845010832	4	3	2	1
ENSCAFG00845010833	0	0	0	0
ENSCAFG00845009849	0	0	0	0
ENSCAFG00845010834	2213	2048	2238	2363
ENSCAFG00845010835	2	0	1	1
ENSCAFG00845010830	0	0	1	0
ENSCAFG00845010831	0	0	0	0
ENSCAFG00845022808	65	76	66	65

ENSCAFG00845022803	473	466	338	340
ENSCAFG00845022802	331	336	386	400
ENSCAFG00845022801	2434	2216	2470	2628
ENSCAFG00845022800	826	847	889	933
ENSCAFG00845022807	1	0	1	0
ENSCAFG00845022806	0	0	0	0
ENSCAFG00845022804	0	0	0	0
ENSCAFG00845009832	125	109	114	99
ENSCAFG00845010818	5	4	7	6
ENSCAFG00845009833	0	0	3	0
ENSCAFG00845010819	4	0	3	0
ENSCAFG00845009830	149	106	169	143
ENSCAFG00845009831	0	0	0	0
ENSCAFG00845009836	0	0	0	0
ENSCAFG00845010825	1061	1010	1093	1104
ENSCAFG00845009837	352	336	304	358
ENSCAFG00845010826	371	314	381	366
ENSCAFG00845009834	0	0	0	0
ENSCAFG00845010827	0	0	1	1
ENSCAFG00845009835	0	0	0	0
ENSCAFG00845010828	5372	5302	5660	6022
ENSCAFG00845010821	0	0	0	0
ENSCAFG00845010822	1	0	1	0
ENSCAFG00845009838	0	0	0	0
ENSCAFG00845010823	13	5	4	0
ENSCAFG00845009839	966	956	804	886
ENSCAFG00845010824	101	103	132	126
ENSCAFG00845010820	13	9	7	9
ENSCAFG00845009821	2	0	0	3
ENSCAFG00845010807	0	0	0	0
ENSCAFG00845009822	590	601	458	518
ENSCAFG00845010808	323	335	303	308
ENSCAFG00845010809	64	38	25	40
ENSCAFG00845009820	218	231	167	164
ENSCAFG00845009825	25	24	20	21
ENSCAFG00845010814	1304	1248	1190	1288
ENSCAFG00845009826	1049	1032	950	1069
ENSCAFG00845010815	0	1	0	0
ENSCAFG00845009823	1005	1005	996	1010
ENSCAFG00845010816	1280	1297	1200	1262
ENSCAFG00845009824	0	0	0	0

ENSCAFG00845010817	0	0	1	0
ENSCAFG00845009829	2	1	6	1
ENSCAFG00845010810	0	0	0	0
ENSCAFG00845010811	0	2	2	1
ENSCAFG00845009827	992	925	1089	999
ENSCAFG00845010812	0	0	2	2
ENSCAFG00845009828	1926	1852	1607	1724
ENSCAFG00845010813	1450	1322	1316	1303
ENSCAFG00845009810	0	0	0	0
ENSCAFG00845009811	0	0	0	0
ENSCAFG00845009814	959	831	784	785
ENSCAFG00845010803	0	0	0	0
ENSCAFG00845009815	0	0	0	0
ENSCAFG00845010804	0	0	0	0
ENSCAFG00845009812	0	0	0	0
ENSCAFG00845010805	886	863	864	820
ENSCAFG00845009813	3	2	7	8
ENSCAFG00845010806	0	0	0	0
ENSCAFG00845009818	212	300	302	280
ENSCAFG00845009819	0	0	0	0
ENSCAFG00845010800	0	0	0	0
ENSCAFG00845009816	0	0	0	0
ENSCAFG00845010801	0	0	4	0
ENSCAFG00845009817	0	0	1	1
ENSCAFG00845010802	0	0	0	0
ENSCAFG00845009800	0	0	0	0
ENSCAFG00845009803	7	6	3	5
ENSCAFG00845009804	0	0	0	0
ENSCAFG00845009801	0	0	0	0
ENSCAFG00845009802	0	0	2	2
ENSCAFG00845009807	8	7	4	8
ENSCAFG00845009808	2	3	1	3
ENSCAFG00845009805	1211	1133	1062	1262
ENSCAFG00845009806	413	424	432	413
ENSCAFG00845009809	137	107	181	159
ENSCAFG00845020248	882	853	883	872
ENSCAFG00845020249	1279	1325	1163	1168
ENSCAFG00845007271	1016	920	1108	999
ENSCAFG00845007270	0	0	0	0
ENSCAFG00845007273	0	0	1	0
ENSCAFG00845007272	0	0	0	0

ENSCAFG00845007275	0	0	0	0
ENSCAFG00845020242	2	0	0	0
ENSCAFG00845007274	96	117	104	92
ENSCAFG00845020243	0	0	0	0
ENSCAFG00845007277	0	0	0	0
ENSCAFG00845020240	0	0	0	0
ENSCAFG00845007276	517	526	527	495
ENSCAFG00845020241	81	55	66	79
ENSCAFG00845007279	0	0	0	0
ENSCAFG00845020246	0	1	1	2
ENSCAFG00845007278	0	0	0	0
ENSCAFG00845020247	474	445	436	494
ENSCAFG00845020244	0	0	0	0
ENSCAFG00845020245	0	0	0	0
ENSCAFG00845019250	1133	1136	883	883
ENSCAFG00845019251	0	0	0	0
ENSCAFG00845019252	606	622	638	605
ENSCAFG00845019253	290	295	322	303
ENSCAFG00845019254	314	313	333	320
ENSCAFG00845019255	0	0	0	0
ENSCAFG00845019256	0	0	0	0
ENSCAFG00845019257	409	441	327	325
ENSCAFG00845019258	793	720	767	800
ENSCAFG00845019259	0	0	2	0
ENSCAFG00845020239	176	149	138	202
ENSCAFG00845020237	0	0	0	0
ENSCAFG00845020238	158	137	159	140
ENSCAFG00845022899	4	2	2	4
ENSCAFG00845007260	23	20	17	25
ENSCAFG00845007262	0	0	0	1
ENSCAFG00845007261	1178	1126	1097	1227
ENSCAFG00845007264	0	0	1	0
ENSCAFG00845020231	0	1	0	0
ENSCAFG00845022894	1761	1605	1730	1685
ENSCAFG00845007263	639	566	603	658
ENSCAFG00845020232	145	184	194	176
ENSCAFG00845022893	7	8	3	8
ENSCAFG00845007266	0	1	0	0
ENSCAFG00845022892	0	0	0	0
ENSCAFG00845007265	1	1	0	0
ENSCAFG00845020230	0	0	0	0

ENSCAFG00845022891	0	0	0	0
ENSCAFG00845007268	0	0	0	0
ENSCAFG00845020235	394	334	412	401
ENSCAFG00845022898	0	0	0	0
ENSCAFG00845007267	0	2	4	4
ENSCAFG00845020236	179	179	202	216
ENSCAFG00845022897	223	186	267	234
ENSCAFG00845020233	1	2	1	6
ENSCAFG00845022896	0	0	0	0
ENSCAFG00845007269	50	42	29	31
ENSCAFG00845020234	0	0	0	0
ENSCAFG00845022895	18	7	7	7
ENSCAFG00845022890	0	0	0	0
ENSCAFG00845019240	2	0	2	0
ENSCAFG00845019241	17	25	18	26
ENSCAFG00845019242	2997	2912	3004	3217
ENSCAFG00845019243	820	778	1145	1210
ENSCAFG00845019245	0	0	0	0
ENSCAFG00845019246	217	192	194	203
ENSCAFG00845019247	1669	1709	1741	1673
ENSCAFG00845019248	2	3	0	2
ENSCAFG00845019249	0	1	0	0
ENSCAFG00845019239	0	0	0	0
ENSCAFG00845020228	0	0	0	0
ENSCAFG00845020229	1	0	0	1
ENSCAFG00845020226	1125	1001	1003	1135
ENSCAFG00845022889	5	7	11	6
ENSCAFG00845020227	2	1	0	1
ENSCAFG00845022888	0	0	0	0
ENSCAFG00845007251	0	0	0	0
ENSCAFG00845007250	1571	1490	1475	1431
ENSCAFG00845007253	0	0	0	0
ENSCAFG00845020220	0	0	0	0
ENSCAFG00845022883	1123	1070	1007	1035
ENSCAFG00845007252	0	0	0	0
ENSCAFG00845020221	0	0	0	0
ENSCAFG00845007255	0	0	0	0
ENSCAFG00845022881	1444	1492	1441	1471
ENSCAFG00845007254	0	5	0	1
ENSCAFG00845022880	15	9	18	14
ENSCAFG00845007257	0	0	0	0

ENSCAFG00845020224	485	437	426	453
ENSCAFG00845022887	403	380	385	450
ENSCAFG00845007256	0	1	1	0
ENSCAFG00845020225	448	394	345	435
ENSCAFG00845022886	0	0	0	0
ENSCAFG00845007259	361	301	275	315
ENSCAFG00845020222	666	654	632	692
ENSCAFG00845022885	891	918	923	961
ENSCAFG00845007258	206	199	188	211
ENSCAFG00845020223	0	0	0	0
ENSCAFG00845022884	0	0	0	0
ENSCAFG00845019230	51	88	66	49
ENSCAFG00845019231	349	309	286	343
ENSCAFG00845019232	0	0	0	0
ENSCAFG00845019233	3	7	0	8
ENSCAFG00845019234	2487	2197	3048	3176
ENSCAFG00845019235	1248	1194	1275	1262
ENSCAFG00845019236	0	0	0	0
ENSCAFG00845019237	197	191	160	182
ENSCAFG00845019238	1609	1549	1596	1651
ENSCAFG00845019228	0	0	0	0
ENSCAFG00845020217	8	0	1	0
ENSCAFG00845019229	0	0	0	0
ENSCAFG00845020218	1956	1967	2055	1964
ENSCAFG00845022879	34	15	26	19
ENSCAFG00845020215	0	0	0	0
ENSCAFG00845022878	498	548	410	451
ENSCAFG00845020216	836	812	762	824
ENSCAFG00845022877	0	0	0	0
ENSCAFG00845007240	0	0	0	0
ENSCAFG00845020219	0	0	0	0
ENSCAFG00845007242	0	0	0	0
ENSCAFG00845007241	0	0	0	0
ENSCAFG00845020210	26	21	42	29
ENSCAFG00845022871	251	223	233	208
ENSCAFG00845007244	3616	3512	4311	4133
ENSCAFG00845022870	26	28	27	17
ENSCAFG00845007243	74	61	87	59
ENSCAFG00845007246	256	254	293	278
ENSCAFG00845020213	206	203	174	163
ENSCAFG00845022876	0	0	0	0

ENSCAFG00845007245	322	297	302	301
ENSCAFG00845020214	140	149	125	106
ENSCAFG00845022875	0	0	0	0
ENSCAFG00845007248	2415	2439	2228	2302
ENSCAFG00845020211	2857	2701	2679	2858
ENSCAFG00845022874	2	0	0	0
ENSCAFG00845007247	0	0	0	0
ENSCAFG00845020212	38	39	42	51
ENSCAFG00845022873	2134	2122	2340	2435
ENSCAFG00845007249	673	646	638	677
ENSCAFG00845010898	2585	2507	2148	1954
ENSCAFG00845010899	0	0	0	0
ENSCAFG00845010894	3871	3953	3231	3230
ENSCAFG00845010895	0	0	0	0
ENSCAFG00845019221	3441	3367	3339	3317
ENSCAFG00845010896	0	0	1	0
ENSCAFG00845019222	0	0	0	0
ENSCAFG00845010897	0	0	0	0
ENSCAFG00845019223	6	3	6	2
ENSCAFG00845010890	6	4	12	10
ENSCAFG00845019224	250	222	250	230
ENSCAFG00845010891	980	913	860	843
ENSCAFG00845019225	0	0	0	0
ENSCAFG00845010892	0	12	10	6
ENSCAFG00845019226	892	826	910	888
ENSCAFG00845010893	2362	2253	1913	1843
ENSCAFG00845019227	157	135	138	140
ENSCAFG00845019217	1	0	0	0
ENSCAFG00845020206	904	796	840	816
ENSCAFG00845022869	6	4	7	5
ENSCAFG00845019218	3	16	1	3
ENSCAFG00845020207	2069	2001	1889	1927
ENSCAFG00845022868	0	0	0	0
ENSCAFG00845019219	3732	3552	3591	3523
ENSCAFG00845020204	561	515	599	569
ENSCAFG00845022867	0	0	0	0
ENSCAFG00845020205	18	9	7	10
ENSCAFG00845022866	7	7	12	15
ENSCAFG00845009890	0	0	0	0
ENSCAFG00845009891	0	0	0	0
ENSCAFG00845020208	2086	1959	1968	2001

ENSCAFG00845020209	0	0	0	0
ENSCAFG00845007231	1504	1502	1456	1492
ENSCAFG00845009894	1714	1590	1546	1475
ENSCAFG00845022861	3587	3437	3177	3266
ENSCAFG00845007230	14	0	7	5
ENSCAFG00845009895	338	287	318	318
ENSCAFG00845022860	122	109	124	119
ENSCAFG00845007233	0	0	0	0
ENSCAFG00845009892	5647	5517	5175	5201
ENSCAFG00845007232	2301	2164	2056	2184
ENSCAFG00845009893	42	50	35	39
ENSCAFG00845007235	0	0	0	0
ENSCAFG00845009898	0	0	0	0
ENSCAFG00845020202	0	0	0	0
ENSCAFG00845022865	0	0	0	0
ENSCAFG00845007234	38	39	53	59
ENSCAFG00845009899	245	302	244	236
ENSCAFG00845020203	487	522	456	506
ENSCAFG00845022864	2740	2737	2610	2502
ENSCAFG00845007237	0	0	0	0
ENSCAFG00845009896	0	0	0	0
ENSCAFG00845020200	1726	1611	1650	1854
ENSCAFG00845022863	0	0	0	0
ENSCAFG00845007236	0	0	0	0
ENSCAFG00845009897	0	0	0	0
ENSCAFG00845020201	129	124	139	159
ENSCAFG00845022862	0	0	0	0
ENSCAFG00845007239	12	20	27	20
ENSCAFG00845007238	11	13	22	28
ENSCAFG00845010887	2	0	2	3
ENSCAFG00845010888	0	0	0	0
ENSCAFG00845010889	0	0	0	0
ENSCAFG00845010883	3708	3837	3055	3134
ENSCAFG00845010884	15	21	12	10
ENSCAFG00845019210	0	0	0	0
ENSCAFG00845010885	0	0	0	0
ENSCAFG00845019211	0	1	0	0
ENSCAFG00845010886	0	0	1	0
ENSCAFG00845019212	375	335	397	339
ENSCAFG00845019213	354	338	378	385
ENSCAFG00845010880	0	0	0	0

ENSCAFG00845019214	0	0	0	0
ENSCAFG00845010881	8783	8653	8220	8331
ENSCAFG00845019215	4	6	0	2
ENSCAFG00845010882	214	239	228	264
ENSCAFG00845019216	210	211	189	206
ENSCAFG00845019206	29	16	22	16
ENSCAFG00845022858	111	98	68	81
ENSCAFG00845019207	0	0	0	0
ENSCAFG00845022857	0	1	0	1
ENSCAFG00845019208	0	0	0	0
ENSCAFG00845022856	12	11	12	4
ENSCAFG00845019209	0	0	0	0
ENSCAFG00845022855	0	0	0	0
ENSCAFG00845009880	0	0	0	0
ENSCAFG00845022859	0	0	0	0
ENSCAFG00845007220	37	16	34	37
ENSCAFG00845009883	40	29	53	57
ENSCAFG00845022850	622	579	503	506
ENSCAFG00845009884	3	1	3	4
ENSCAFG00845007222	2791	2624	2522	2762
ENSCAFG00845009881	0	0	0	0
ENSCAFG00845007221	1	0	0	0
ENSCAFG00845009882	14	9	6	8
ENSCAFG00845007224	1164	1136	1177	1156
ENSCAFG00845009887	159	110	116	143
ENSCAFG00845022854	6	2	5	3
ENSCAFG00845007223	0	0	0	0
ENSCAFG00845009888	0	0	0	0
ENSCAFG00845022853	0	0	0	0
ENSCAFG00845007226	0	0	0	0
ENSCAFG00845009885	0	0	0	0
ENSCAFG00845022852	0	3	0	0
ENSCAFG00845007225	0	0	0	0
ENSCAFG00845009886	5	1	4	0
ENSCAFG00845022851	206	180	283	261
ENSCAFG00845007228	3	6	1	5
ENSCAFG00845007227	5	4	0	1
ENSCAFG00845009889	12	16	27	16
ENSCAFG00845007229	1	0	0	0
ENSCAFG00845010876	0	0	0	0
ENSCAFG00845010877	2	1	7	2

ENSCAFG00845010878	590	633	655	667
ENSCAFG00845010879	481	480	397	415
ENSCAFG00845010872	6	11	11	8
ENSCAFG00845010873	202	155	231	223
ENSCAFG00845010874	1987	1860	1730	1957
ENSCAFG00845019200	0	0	1	0
ENSCAFG00845010875	0	0	0	2
ENSCAFG00845019201	0	0	0	0
ENSCAFG00845019202	3	0	1	3
ENSCAFG00845019203	0	0	0	0
ENSCAFG00845010870	0	0	2	5
ENSCAFG00845019204	568	515	535	537
ENSCAFG00845010871	57	98	85	86
ENSCAFG00845019205	0	0	0	0
ENSCAFG00845022847	0	0	0	0
ENSCAFG00845022846	0	0	0	0
ENSCAFG00845022845	131	137	88	107
ENSCAFG00845022844	0	0	1	0
ENSCAFG00845022849	0	0	0	0
ENSCAFG00845009872	0	0	0	1
ENSCAFG00845009873	2016	1917	2107	1934
ENSCAFG00845007211	3	0	5	0
ENSCAFG00845009870	0	0	0	0
ENSCAFG00845007210	2	1	2	2
ENSCAFG00845009871	396	392	330	364
ENSCAFG00845007213	0	0	0	0
ENSCAFG00845009876	0	0	0	0
ENSCAFG00845022843	0	0	0	0
ENSCAFG00845007212	0	0	0	0
ENSCAFG00845009877	1434	1499	1315	1337
ENSCAFG00845022842	0	0	0	0
ENSCAFG00845007215	355	384	288	340
ENSCAFG00845009874	181	174	143	153
ENSCAFG00845022841	0	0	0	0
ENSCAFG00845007214	12	7	8	9
ENSCAFG00845009875	1	6	2	1
ENSCAFG00845022840	0	0	0	0
ENSCAFG00845007217	0	0	0	0
ENSCAFG00845010869	588	554	549	503
ENSCAFG00845007216	269	271	296	298
ENSCAFG00845007219	1012	1022	979	981

ENSCAFG00845009878	0	0	0	0
ENSCAFG00845007218	0	0	0	1
ENSCAFG00845009879	0	0	0	0
ENSCAFG00845010865	2	5	6	6
ENSCAFG00845010866	259	260	340	375
ENSCAFG00845010867	0	0	0	0
ENSCAFG00845010868	2	2	1	3
ENSCAFG00845010861	388	407	480	463
ENSCAFG00845010862	14	11	9	18
ENSCAFG00845010863	965	943	826	810
ENSCAFG00845010864	0	0	0	0
ENSCAFG00845010860	0	0	0	0
ENSCAFG00845022836	0	0	0	0
ENSCAFG00845022835	1122	1046	1087	1112
ENSCAFG00845022834	0	0	0	0
ENSCAFG00845022833	598	508	495	467
ENSCAFG00845022839	0	0	0	0
ENSCAFG00845022838	594	505	478	499
ENSCAFG00845022837	10	10	8	8
ENSCAFG00845009861	1431	1341	1386	1402
ENSCAFG00845009862	0	5	0	1
ENSCAFG00845007200	35	31	29	24
ENSCAFG00845009860	0	0	0	0
ENSCAFG00845007202	3281	3048	2986	2924
ENSCAFG00845009865	2	0	3	2
ENSCAFG00845022832	69	56	71	66
ENSCAFG00845007201	0	0	0	0
ENSCAFG00845009866	0	0	0	0
ENSCAFG00845022831	10	7	4	8
ENSCAFG00845007204	2	1	6	5
ENSCAFG00845009863	9304	8930	9793	9792
ENSCAFG00845022830	0	0	0	0
ENSCAFG00845007203	0	0	0	0
ENSCAFG00845009864	0	0	0	0
ENSCAFG00845007206	0	0	0	0
ENSCAFG00845009869	732	707	741	750
ENSCAFG00845010858	0	0	0	0
ENSCAFG00845007205	605	605	553	569
ENSCAFG00845010859	388	393	358	378
ENSCAFG00845007208	567	599	613	615
ENSCAFG00845009867	859	888	922	945

ENSCAFG00845007207	675	722	777	705
ENSCAFG00845009868	0	0	0	0
ENSCAFG00845010854	0	0	0	0
ENSCAFG00845007209	0	0	0	0
ENSCAFG00845010855	134	125	121	127
ENSCAFG00845010856	19	9	19	16
ENSCAFG00845010857	0	0	0	0
ENSCAFG00845010850	6	0	4	3
ENSCAFG00845010851	1240	1172	1173	1179
ENSCAFG00845010852	398	364	317	292
ENSCAFG00845010853	237	218	194	240
ENSCAFG00845020198	23	31	18	30
ENSCAFG00845020199	451	419	442	449
ENSCAFG00845020196	0	0	0	0
ENSCAFG00845020197	279	240	291	318
ENSCAFG00845020190	1287	1241	1386	1353
ENSCAFG00845020191	45	23	35	34
ENSCAFG00845020194	0	0	0	0
ENSCAFG00845020195	312	310	263	259
ENSCAFG00845020192	2	0	0	3
ENSCAFG00845020193	47	31	67	56
ENSCAFG00845020187	849	895	777	800
ENSCAFG00845020188	0	0	0	0
ENSCAFG00845020185	0	0	0	0
ENSCAFG00845020186	558	488	535	617
ENSCAFG00845020189	0	0	0	0
ENSCAFG00845019190	142	144	134	122
ENSCAFG00845019191	0	0	0	0
ENSCAFG00845020180	0	0	0	0
ENSCAFG00845019192	1	1	2	4
ENSCAFG00845019193	0	0	0	0
ENSCAFG00845019194	16	21	12	29
ENSCAFG00845020183	0	0	0	0
ENSCAFG00845019195	1018	967	1017	1030
ENSCAFG00845020184	181	193	172	184
ENSCAFG00845019196	0	0	0	0
ENSCAFG00845020181	1304	1218	1211	1253
ENSCAFG00845019197	0	0	0	0
ENSCAFG00845020182	372	326	362	339
ENSCAFG00845019198	0	3	0	2
ENSCAFG00845019199	608	610	666	689

ENSCAFG00845020176	0	0	0	0
ENSCAFG00845020177	0	0	0	0
ENSCAFG00845020174	1	1	0	0
ENSCAFG00845020175	0	1	0	0
ENSCAFG00845020178	0	0	0	0
ENSCAFG00845020179	0	0	0	1
ENSCAFG00845019180	2422	2301	2171	2295
ENSCAFG00845019181	0	0	0	0
ENSCAFG00845019183	0	0	0	0
ENSCAFG00845020172	0	0	0	0
ENSCAFG00845019184	0	0	0	0
ENSCAFG00845020173	1790	1830	1768	1675
ENSCAFG00845019185	329	403	302	341
ENSCAFG00845020170	0	0	0	0
ENSCAFG00845019186	0	0	0	0
ENSCAFG00845020171	761	736	714	776
ENSCAFG00845019187	0	0	0	0
ENSCAFG00845019188	0	1	0	0
ENSCAFG00845019189	0	0	1	0
ENSCAFG00845007190	0	0	0	0
ENSCAFG00845007192	1141	1077	952	989
ENSCAFG00845007191	480	424	367	377
ENSCAFG00845007194	0	3	0	0
ENSCAFG00845007193	0	0	0	0
ENSCAFG00845007196	1179	1160	1019	1131
ENSCAFG00845007195	4	6	3	9
ENSCAFG00845007198	0	0	0	0
ENSCAFG00845020165	0	0	0	0
ENSCAFG00845007197	9	6	10	4
ENSCAFG00845020166	0	0	0	0
ENSCAFG00845020163	0	0	0	0
ENSCAFG00845007199	0	0	0	0
ENSCAFG00845020164	1673	1674	1695	1699
ENSCAFG00845020169	1	1	1	0
ENSCAFG00845020167	0	0	0	0
ENSCAFG00845020168	0	0	0	0
ENSCAFG00845019170	7	7	9	11
ENSCAFG00845019171	0	0	0	0
ENSCAFG00845019172	0	0	0	0
ENSCAFG00845020161	36	34	29	28
ENSCAFG00845019173	1	0	0	0

ENSCAFG00845020162	0	2	2	4
ENSCAFG00845019174	2565	2604	2306	2518
ENSCAFG00845019175	2371	2284	2355	2509
ENSCAFG00845020160	808	692	675	699
ENSCAFG00845019176	161	161	143	152
ENSCAFG00845019177	1	0	1	0
ENSCAFG00845019178	0	0	0	0
ENSCAFG00845019179	0	0	0	0
ENSCAFG00845007181	15	9	6	12
ENSCAFG00845007180	1867	1812	1570	1659
ENSCAFG00845007183	2	2	0	4
ENSCAFG00845007182	0	0	0	3
ENSCAFG00845007185	2350	2142	2112	2223
ENSCAFG00845007184	0	0	0	0
ENSCAFG00845007187	0	0	0	0
ENSCAFG00845020154	0	0	0	0
ENSCAFG00845007186	7	7	9	10
ENSCAFG00845020155	3	2	2	0
ENSCAFG00845007189	0	0	0	0
ENSCAFG00845020152	3	3	0	1
ENSCAFG00845007188	2	0	0	0
ENSCAFG00845020153	0	0	2	0
ENSCAFG00845020158	0	0	0	0
ENSCAFG00845020159	0	0	0	0
ENSCAFG00845020156	0	0	0	0
ENSCAFG00845020157	0	0	0	0
ENSCAFG00845019160	0	0	0	0
ENSCAFG00845019161	0	0	0	0
ENSCAFG00845020150	0	0	0	0
ENSCAFG00845019162	0	0	1	1
ENSCAFG00845020151	18	14	19	23
ENSCAFG00845019163	220	233	242	267
ENSCAFG00845019164	2159	2075	2012	2007
ENSCAFG00845019165	1	3	10	2
ENSCAFG00845019166	1873	1912	1912	1898
ENSCAFG00845019167	0	0	0	0
ENSCAFG00845019168	0	0	0	0
ENSCAFG00845019169	213	205	174	142
ENSCAFG00845007170	0	0	0	0
ENSCAFG00845020149	4	4	4	5
ENSCAFG00845007172	1	0	1	0

ENSCAFG00845007171	39	52	47	45
ENSCAFG00845007174	2262	2211	1981	1966
ENSCAFG00845007173	0	0	0	0
ENSCAFG00845007176	0	0	0	0
ENSCAFG00845020143	0	0	0	0
ENSCAFG00845007175	0	0	0	0
ENSCAFG00845020144	5	3	5	2
ENSCAFG00845007178	0	0	0	0
ENSCAFG00845020141	0	0	0	0
ENSCAFG00845007177	555	537	586	606
ENSCAFG00845020142	514	504	512	601
ENSCAFG00845020147	0	0	0	0
ENSCAFG00845007179	9	3	7	5
ENSCAFG00845020148	101	89	99	103
ENSCAFG00845020145	0	0	0	0
ENSCAFG00845020146	0	0	0	0
ENSCAFG00845019150	0	0	0	0
ENSCAFG00845019151	0	0	0	0
ENSCAFG00845020140	2	7	1	3
ENSCAFG00845019152	1056	1017	1036	986
ENSCAFG00845019153	1	3	1	1
ENSCAFG00845019154	661	596	602	635
ENSCAFG00845019155	2	0	0	1
ENSCAFG00845019156	0	0	0	0
ENSCAFG00845019157	0	0	1	0
ENSCAFG00845019158	0	0	0	0
ENSCAFG00845019159	0	0	0	0
ENSCAFG00845020138	253	232	248	298
ENSCAFG00845020139	9	7	6	6
ENSCAFG00845007161	0	0	0	0
ENSCAFG00845007160	0	0	0	0
ENSCAFG00845007163	0	0	0	0
ENSCAFG00845007162	0	0	0	0
ENSCAFG00845007165	2613	2582	2858	2853
ENSCAFG00845020132	0	0	0	0
ENSCAFG00845022795	4	0	0	0
ENSCAFG00845007164	0	0	0	0
ENSCAFG00845020133	0	0	0	0
ENSCAFG00845022794	3631	3752	3386	3479
ENSCAFG00845007167	0	0	0	0
ENSCAFG00845020130	0	1	0	0

ENSCAFG00845022793	1103	1049	960	1055
ENSCAFG00845007166	725	578	608	654
ENSCAFG00845020131	0	0	0	0
ENSCAFG00845022792	761	729	710	657
ENSCAFG00845007169	0	1	0	0
ENSCAFG00845020136	0	0	0	0
ENSCAFG00845022799	321	276	395	347
ENSCAFG00845007168	0	0	0	0
ENSCAFG00845020137	0	0	0	0
ENSCAFG00845022798	2	1	4	1
ENSCAFG00845020134	1	1	2	2
ENSCAFG00845022797	0	0	0	0
ENSCAFG00845020135	1	0	2	9
ENSCAFG00845022796	303	321	296	273
ENSCAFG00845022791	0	0	0	0
ENSCAFG00845019140	0	0	0	0
ENSCAFG00845022790	0	0	0	0
ENSCAFG00845019141	4	5	3	1
ENSCAFG00845019142	1	2	0	0
ENSCAFG00845019143	15	14	17	14
ENSCAFG00845019144	0	2	0	0
ENSCAFG00845019145	689	681	641	709
ENSCAFG00845019146	4	2	4	10
ENSCAFG00845019147	5	3	3	4
ENSCAFG00845019148	0	0	1	0
ENSCAFG00845019149	700	709	613	541
ENSCAFG00845022946	0	0	0	0
ENSCAFG00845022945	1	0	2	0
ENSCAFG00845022944	0	0	0	0
ENSCAFG00845022943	0	0	0	0
ENSCAFG00845022949	3	4	0	2
ENSCAFG00845022948	0	0	0	0
ENSCAFG00845009971	79	64	60	49
ENSCAFG00845009972	910	884	871	960
ENSCAFG00845007310	154	125	125	118
ENSCAFG00845009970	1168	1012	1062	1046
ENSCAFG00845007312	0	0	0	0
ENSCAFG00845009975	72	59	45	46
ENSCAFG00845022942	132	129	150	161
ENSCAFG00845007311	121	144	121	100
ENSCAFG00845009976	1	0	0	0

ENSCAFG00845022941	3	0	2	5
ENSCAFG00845007314	0	0	0	0
ENSCAFG00845009973	0	0	0	0
ENSCAFG00845022940	116	102	127	115
ENSCAFG00845007313	0	0	0	0
ENSCAFG00845009974	7979	7663	7119	7289
ENSCAFG00845007316	99	55	81	89
ENSCAFG00845009979	3	7	3	2
ENSCAFG00845010968	0	0	0	0
ENSCAFG00845007315	4305	4052	3766	3731
ENSCAFG00845010969	0	0	0	0
ENSCAFG00845007318	6113	5788	5484	5335
ENSCAFG00845009977	23	30	39	32
ENSCAFG00845007317	2	0	2	2
ENSCAFG00845009978	0	0	0	0
ENSCAFG00845010964	0	0	0	0
ENSCAFG00845007319	2289	2209	2343	2270
ENSCAFG00845010965	0	0	0	0
ENSCAFG00845010966	3030	2882	2700	2748
ENSCAFG00845010967	0	0	0	0
ENSCAFG00845010960	1442	1456	1321	1453
ENSCAFG00845010961	1951	1793	1875	1847
ENSCAFG00845010962	369	304	333	343
ENSCAFG00845010963	10	4	0	6
ENSCAFG00845022934	484	515	459	496
ENSCAFG00845022933	1575	1553	1564	1524
ENSCAFG00845022932	0	0	0	0
ENSCAFG00845022939	0	0	0	0
ENSCAFG00845022938	3	6	2	1
ENSCAFG00845022937	0	0	0	0
ENSCAFG00845022936	0	0	0	0
ENSCAFG00845009960	0	0	0	0
ENSCAFG00845009961	3158	2921	3176	3179
ENSCAFG00845007301	780	721	728	712
ENSCAFG00845009964	4	1	1	2
ENSCAFG00845022931	44	41	45	41
ENSCAFG00845007300	6	1	0	0
ENSCAFG00845009965	27	49	49	40
ENSCAFG00845022930	812	616	718	713
ENSCAFG00845007303	6	2	3	7
ENSCAFG00845009962	1	0	0	0

ENSCAFG00845007302	14	10	5	3
ENSCAFG00845009963	661	767	687	671
ENSCAFG00845007305	13	16	11	9
ENSCAFG00845009968	0	0	0	0
ENSCAFG00845010957	0	0	0	0
ENSCAFG00845007304	0	0	0	0
ENSCAFG00845009969	332	327	334	328
ENSCAFG00845010958	1026	933	954	1012
ENSCAFG00845007307	0	3	0	2
ENSCAFG00845009966	186	191	200	201
ENSCAFG00845010959	3	6	6	3
ENSCAFG00845007306	301	247	234	246
ENSCAFG00845009967	1	2	10	2
ENSCAFG00845007309	6	15	2	2
ENSCAFG00845010953	0	0	0	2
ENSCAFG00845007308	192	196	205	189
ENSCAFG00845010954	3	2	1	0
ENSCAFG00845010955	1691	1570	1849	1832
ENSCAFG00845010956	1	5	1	3
ENSCAFG00845010950	0	0	0	0
ENSCAFG00845010951	1483	1380	1373	1394
ENSCAFG00845010952	0	0	0	0
ENSCAFG00845022929	2	1	1	0
ENSCAFG00845022924	0	0	3	1
ENSCAFG00845022923	0	0	0	0
ENSCAFG00845022922	0	0	0	0
ENSCAFG00845022921	1	0	0	0
ENSCAFG00845022928	87	70	68	87
ENSCAFG00845022927	0	0	3	3
ENSCAFG00845022926	326	290	281	247
ENSCAFG00845022925	0	0	0	0
ENSCAFG00845009950	0	0	0	0
ENSCAFG00845009953	0	0	1	0
ENSCAFG00845010939	0	0	0	0
ENSCAFG00845022920	0	0	0	0
ENSCAFG00845009954	0	0	0	0
ENSCAFG00845009951	6268	5898	5682	5918
ENSCAFG00845009952	2	0	3	1
ENSCAFG00845009957	946	875	754	697
ENSCAFG00845010946	0	0	0	0
ENSCAFG00845009958	0	0	2	3

ENSCAFG00845010947	0	0	0	0
ENSCAFG00845009955	1	3	5	5
ENSCAFG00845010948	1700	1647	1719	1697
ENSCAFG00845009956	229	205	175	179
ENSCAFG00845010949	1626	1635	1575	1692
ENSCAFG00845010942	217	212	221	217
ENSCAFG00845010943	353	330	300	314
ENSCAFG00845009959	1	0	1	2
ENSCAFG00845010944	1028	1011	786	821
ENSCAFG00845010945	21	19	18	19
ENSCAFG00845010940	591	604	530	597
ENSCAFG00845022919	0	0	1	0
ENSCAFG00845010941	744	750	821	708
ENSCAFG00845022913	2870	2638	2225	2292
ENSCAFG00845022912	0	0	0	0
ENSCAFG00845022911	0	0	0	0
ENSCAFG00845022917	0	0	0	0
ENSCAFG00845022916	502	595	527	587
ENSCAFG00845022915	837	752	836	841
ENSCAFG00845022914	0	0	0	0
ENSCAFG00845009942	883	908	795	861
ENSCAFG00845010928	524	448	493	481
ENSCAFG00845009943	25	27	31	23
ENSCAFG00845010929	0	0	0	0
ENSCAFG00845009940	3	0	4	6
ENSCAFG00845009941	0	2	0	1
ENSCAFG00845009946	169	133	145	157
ENSCAFG00845010935	0	0	0	0
ENSCAFG00845009947	0	0	0	0
ENSCAFG00845010936	244	205	186	220
ENSCAFG00845009944	106	98	87	117
ENSCAFG00845010937	94	92	97	113
ENSCAFG00845009945	0	0	0	0
ENSCAFG00845010938	3	11	7	2
ENSCAFG00845010931	329	361	363	356
ENSCAFG00845010932	1092	1144	1052	975
ENSCAFG00845009948	70	91	99	80
ENSCAFG00845010933	1635	1690	1551	1638
ENSCAFG00845009949	0	0	0	0
ENSCAFG00845010934	1	1	0	2
ENSCAFG00845022909	2969	2988	3230	3255

ENSCAFG00845022908	0	0	0	0
ENSCAFG00845010930	207	158	115	152
ENSCAFG00845022907	0	0	0	0
ENSCAFG00845022902	0	0	0	0
ENSCAFG00845022901	0	0	0	0
ENSCAFG00845022900	2	0	0	0
ENSCAFG00845022906	0	0	0	0
ENSCAFG00845022905	0	0	0	0
ENSCAFG00845022904	0	0	0	0
ENSCAFG00845022903	0	0	0	0
ENSCAFG00845009931	58	54	40	52
ENSCAFG00845010917	0	0	0	0
ENSCAFG00845009932	0	3	0	0
ENSCAFG00845010918	10	16	6	7
ENSCAFG00845010919	437	404	411	429
ENSCAFG00845009930	0	0	0	0
ENSCAFG00845009935	0	0	0	0
ENSCAFG00845010924	0	4	2	1
ENSCAFG00845009936	0	0	0	0
ENSCAFG00845010925	0	0	0	0
ENSCAFG00845009933	10	25	10	8
ENSCAFG00845010926	1485	1438	1580	1491
ENSCAFG00845009934	465	440	440	461
ENSCAFG00845010927	0	0	0	0
ENSCAFG00845009939	0	0	0	0
ENSCAFG00845010920	23	29	59	30
ENSCAFG00845010921	2398	2245	2506	2689
ENSCAFG00845009937	225	238	200	266
ENSCAFG00845010922	703	751	714	714
ENSCAFG00845009938	749	771	836	855
ENSCAFG00845010923	0	0	0	0
ENSCAFG00845009920	0	0	0	0
ENSCAFG00845010906	5	4	12	11
ENSCAFG00845009921	0	3	0	0
ENSCAFG00845010907	2	0	0	1
ENSCAFG00845010908	6	5	3	1
ENSCAFG00845010909	13	20	11	8
ENSCAFG00845009924	24	11	11	20
ENSCAFG00845010913	228	257	233	283
ENSCAFG00845009925	277	226	200	237
ENSCAFG00845010914	106	113	114	112

ENSCAFG00845009922	216	191	180	220
ENSCAFG00845010915	11	4	1	16
ENSCAFG00845009923	5	1	4	0
ENSCAFG00845010916	3	2	0	2
ENSCAFG00845009928	17	12	10	7
ENSCAFG00845009929	0	0	0	0
ENSCAFG00845010910	18	11	17	21
ENSCAFG00845009926	4	1	19	8
ENSCAFG00845010911	1867	1816	1882	1764
ENSCAFG00845009927	140	147	109	164
ENSCAFG00845010912	0	0	0	0
ENSCAFG00845009910	0	0	0	0
ENSCAFG00845009913	34	29	43	28
ENSCAFG00845010902	4	2	0	3
ENSCAFG00845009914	0	0	0	0
ENSCAFG00845010903	640	733	734	664
ENSCAFG00845009911	10	8	6	0
ENSCAFG00845010904	0	0	0	0
ENSCAFG00845009912	473	444	388	414
ENSCAFG00845010905	0	0	0	0
ENSCAFG00845009917	0	0	0	0
ENSCAFG00845009918	546	512	426	456
ENSCAFG00845009915	40	56	47	65
ENSCAFG00845010900	605	634	569	524
ENSCAFG00845009916	380	384	427	407
ENSCAFG00845010901	3	6	5	6
ENSCAFG00845009919	0	0	0	0
ENSCAFG00845009902	0	0	0	0
ENSCAFG00845009903	454	400	424	388
ENSCAFG00845009900	0	0	0	0
ENSCAFG00845009901	360	375	321	336
ENSCAFG00845009906	0	0	0	0
ENSCAFG00845009907	2	13	2	5
ENSCAFG00845009904	786	778	813	801
ENSCAFG00845009905	1995	2007	2014	2024
ENSCAFG00845009908	0	0	0	0
ENSCAFG00845009909	236	204	192	206
ENSCAFG00845007390	0	0	0	0
ENSCAFG00845020369	8	12	8	10
ENSCAFG00845007392	212	202	179	188
ENSCAFG00845007391	0	0	0	0

ENSCAFG00845007394	1	0	0	1
ENSCAFG00845007393	0	0	0	0
ENSCAFG00845007396	1	0	0	0
ENSCAFG00845020363	1816	1692	1608	1631
ENSCAFG00845007395	0	0	0	0
ENSCAFG00845020364	0	0	0	0
ENSCAFG00845007398	613	559	552	606
ENSCAFG00845020361	476	412	384	408
ENSCAFG00845007397	0	0	0	0
ENSCAFG00845020362	523	412	457	457
ENSCAFG00845020367	650	627	569	623
ENSCAFG00845007399	0	0	0	0
ENSCAFG00845020368	0	0	0	0
ENSCAFG00845020365	1387	1390	1376	1364
ENSCAFG00845020366	23	27	22	26
ENSCAFG00845019370	413	400	381	416
ENSCAFG00845019371	346	356	313	317
ENSCAFG00845020360	17	12	15	7
ENSCAFG00845019372	1651	1487	1987	2027
ENSCAFG00845019373	0	0	0	0
ENSCAFG00845019374	2572	2329	2676	2764
ENSCAFG00845019375	0	0	0	0
ENSCAFG00845019376	24	27	15	25
ENSCAFG00845019377	2	0	0	1
ENSCAFG00845019378	51	46	59	60
ENSCAFG00845019379	117	130	146	141
ENSCAFG00845020358	395	347	389	368
ENSCAFG00845020359	29	17	13	8
ENSCAFG00845007381	188	191	159	174
ENSCAFG00845007380	0	0	0	0
ENSCAFG00845007383	554	539	474	455
ENSCAFG00845007382	0	0	0	0
ENSCAFG00845007385	1	1	2	1
ENSCAFG00845020352	20	10	4	11
ENSCAFG00845007384	5	4	2	0
ENSCAFG00845020353	0	0	0	0
ENSCAFG00845007387	290	291	285	284
ENSCAFG00845020350	350	348	366	367
ENSCAFG00845007386	5	2	2	3
ENSCAFG00845020351	0	3	0	0
ENSCAFG00845007389	0	0	0	0

ENSCAFG00845020356	0	0	0	0
ENSCAFG00845007388	0	0	0	0
ENSCAFG00845020357	929	889	1001	943
ENSCAFG00845020354	376	369	361	428
ENSCAFG00845020355	70	84	84	67
ENSCAFG00845019360	392	385	437	450
ENSCAFG00845019361	7	0	0	0
ENSCAFG00845019362	1	6	2	0
ENSCAFG00845019363	0	0	0	0
ENSCAFG00845019364	1	2	2	3
ENSCAFG00845019365	49	51	51	42
ENSCAFG00845019366	9	8	15	7
ENSCAFG00845019367	36	30	20	20
ENSCAFG00845019368	0	0	0	0
ENSCAFG00845019369	952	788	857	861
ENSCAFG00845020349	0	0	2	0
ENSCAFG00845020347	0	1	0	0
ENSCAFG00845020348	0	0	0	0
ENSCAFG00845007370	0	0	0	0
ENSCAFG00845007372	0	0	0	0
ENSCAFG00845007371	0	0	0	0
ENSCAFG00845007374	1348	1316	1389	1400
ENSCAFG00845020341	5249	4909	4851	5071
ENSCAFG00845007373	748	723	647	704
ENSCAFG00845020342	0	0	0	0
ENSCAFG00845007376	0	0	0	0
ENSCAFG00845007375	482	441	521	487
ENSCAFG00845020340	43	22	46	41
ENSCAFG00845007378	158	127	116	123
ENSCAFG00845020345	0	0	0	0
ENSCAFG00845007377	0	0	0	0
ENSCAFG00845020346	999	918	846	986
ENSCAFG00845020343	2	1	4	0
ENSCAFG00845007379	756	704	698	732
ENSCAFG00845020344	0	0	0	1
ENSCAFG00845019350	1149	1200	1142	1247
ENSCAFG00845019351	0	0	0	0
ENSCAFG00845019352	0	0	0	0
ENSCAFG00845019353	190	206	191	200
ENSCAFG00845019354	349	280	309	342
ENSCAFG00845019355	0	0	0	0

ENSCAFG00845019356	0	0	0	0
ENSCAFG00845019357	2728	2733	2643	2606
ENSCAFG00845019358	0	0	0	0
ENSCAFG00845019359	1072	1070	999	1068
ENSCAFG00845019349	1073	984	1097	1123
ENSCAFG00845020338	186	209	174	212
ENSCAFG00845020339	412	439	442	456
ENSCAFG00845022999	719	774	630	647
ENSCAFG00845020337	257	211	258	202
ENSCAFG00845022998	141	121	126	144
ENSCAFG00845007361	0	0	0	0
ENSCAFG00845007360	12	8	6	9
ENSCAFG00845007363	0	0	0	0
ENSCAFG00845020330	5	5	2	6
ENSCAFG00845022993	0	0	0	0
ENSCAFG00845007362	0	0	0	0
ENSCAFG00845020331	0	0	0	0
ENSCAFG00845022992	2112	2229	2141	2075
ENSCAFG00845007365	1	0	0	0
ENSCAFG00845022991	0	0	0	0
ENSCAFG00845007364	8	16	16	15
ENSCAFG00845022990	0	0	0	0
ENSCAFG00845007367	0	0	0	0
ENSCAFG00845020334	1	0	0	1
ENSCAFG00845022997	0	0	0	0
ENSCAFG00845007366	599	595	569	598
ENSCAFG00845020335	739	739	657	687
ENSCAFG00845022996	53	38	68	82
ENSCAFG00845007369	0	0	0	0
ENSCAFG00845020332	99	104	84	89
ENSCAFG00845022995	0	0	0	0
ENSCAFG00845007368	0	0	0	0
ENSCAFG00845020333	0	0	0	0
ENSCAFG00845022994	1596	1372	1659	1638
ENSCAFG00845019340	1231	1106	1160	1160
ENSCAFG00845019341	3160	3044	2877	3026
ENSCAFG00845019342	590	565	468	451
ENSCAFG00845019343	488	429	469	485
ENSCAFG00845019344	0	0	0	0
ENSCAFG00845019345	7	3	2	2
ENSCAFG00845019346	726	713	848	809

ENSCAFG00845019347	0	0	0	0
ENSCAFG00845019348	38	52	57	48
ENSCAFG00845019338	0	0	0	0
ENSCAFG00845020327	0	1	2	1
ENSCAFG00845019339	16	23	28	37
ENSCAFG00845020328	794	859	851	842
ENSCAFG00845022989	0	0	0	0
ENSCAFG00845020325	0	0	0	0
ENSCAFG00845022988	0	0	0	0
ENSCAFG00845020326	276	231	215	205
ENSCAFG00845007350	0	0	0	0
ENSCAFG00845020329	0	0	1	0
ENSCAFG00845007352	0	0	0	0
ENSCAFG00845022982	1	8	2	10
ENSCAFG00845007351	1	0	0	0
ENSCAFG00845020320	516	513	489	590
ENSCAFG00845022981	0	0	0	0
ENSCAFG00845007354	969	890	895	907
ENSCAFG00845022980	108	113	106	125
ENSCAFG00845007353	1944	1887	1859	1981
ENSCAFG00845007356	1	0	1	1
ENSCAFG00845020323	0	0	1	0
ENSCAFG00845022986	0	0	0	0
ENSCAFG00845007355	4	0	0	2
ENSCAFG00845020324	773	660	548	576
ENSCAFG00845022985	0	0	0	0
ENSCAFG00845007358	0	2	0	0
ENSCAFG00845020321	0	1	1	0
ENSCAFG00845022984	2	5	1	6
ENSCAFG00845007357	0	0	0	0
ENSCAFG00845020322	1973	1816	1889	1837
ENSCAFG00845022983	1151	1206	1275	1273
ENSCAFG00845007359	614	618	604	652
ENSCAFG00845019330	0	2	7	0
ENSCAFG00845019331	0	0	0	0
ENSCAFG00845019332	0	0	0	0
ENSCAFG00845019333	4928	4743	4645	4554
ENSCAFG00845019334	3005	2999	2838	2910
ENSCAFG00845019335	51	48	52	48
ENSCAFG00845019336	0	0	0	0
ENSCAFG00845019337	103	86	91	120

ENSCAFG00845019327	0	0	0	0
ENSCAFG00845020316	20	12	15	9
ENSCAFG00845022979	0	0	0	0
ENSCAFG00845019328	904	960	922	930
ENSCAFG00845020317	0	0	0	0
ENSCAFG00845022978	0	3	2	1
ENSCAFG00845019329	2	0	1	0
ENSCAFG00845020314	50	63	55	61
ENSCAFG00845022977	2886	2878	2764	2924
ENSCAFG00845020315	0	4	12	6
ENSCAFG00845022976	0	0	0	0
ENSCAFG00845020318	4	1	0	0
ENSCAFG00845020319	1	0	0	0
ENSCAFG00845007341	19	8	14	21
ENSCAFG00845022971	1232	1139	1238	1199
ENSCAFG00845007340	145	121	89	87
ENSCAFG00845022970	0	1	3	3
ENSCAFG00845007343	0	0	0	0
ENSCAFG00845007342	0	0	0	0
ENSCAFG00845007345	1651	1514	1466	1520
ENSCAFG00845022975	0	0	0	0
ENSCAFG00845007344	0	0	0	0
ENSCAFG00845020313	928	863	887	922
ENSCAFG00845022974	0	0	0	0
ENSCAFG00845007347	4	8	5	1
ENSCAFG00845020310	3	6	0	2
ENSCAFG00845022973	0	0	0	0
ENSCAFG00845007346	3	3	1	1
ENSCAFG00845020311	636	621	699	631
ENSCAFG00845022972	275	240	258	275
ENSCAFG00845007349	0	0	0	0
ENSCAFG00845007348	1	0	3	2
ENSCAFG00845010997	11	21	14	16
ENSCAFG00845010998	1068	1021	822	687
ENSCAFG00845010999	21	12	18	12
ENSCAFG00845010993	21	28	36	32
ENSCAFG00845010994	0	0	0	0
ENSCAFG00845019320	0	0	0	0
ENSCAFG00845010995	15	11	6	7
ENSCAFG00845019321	0	1	0	0
ENSCAFG00845010996	46	53	41	51

ENSCAFG00845019322	0	0	0	0
ENSCAFG00845019323	479	504	587	518
ENSCAFG00845010990	0	0	0	0
ENSCAFG00845019324	255	284	325	300
ENSCAFG00845010991	0	0	0	0
ENSCAFG00845019325	2	2	1	2
ENSCAFG00845010992	0	0	0	0
ENSCAFG00845019326	413	365	337	361
ENSCAFG00845019316	183	214	174	200
ENSCAFG00845020305	0	0	0	0
ENSCAFG00845022968	0	0	0	0
ENSCAFG00845019317	317	306	287	251
ENSCAFG00845020306	0	0	0	0
ENSCAFG00845022967	0	0	0	0
ENSCAFG00845019318	36	31	32	31
ENSCAFG00845020303	0	0	0	0
ENSCAFG00845022966	2	0	0	0
ENSCAFG00845019319	0	0	0	0
ENSCAFG00845020304	0	0	0	0
ENSCAFG00845022965	231	201	167	196
ENSCAFG00845020309	1	0	0	0
ENSCAFG00845009990	0	0	0	0
ENSCAFG00845020307	89	89	89	86
ENSCAFG00845020308	0	0	0	0
ENSCAFG00845022969	0	0	1	1
ENSCAFG00845007330	0	4	0	2
ENSCAFG00845009993	6	6	1	2
ENSCAFG00845022960	0	0	0	0
ENSCAFG00845009994	1	1	0	1
ENSCAFG00845007332	1	0	0	0
ENSCAFG00845009991	13439	12841	13592	14049
ENSCAFG00845007331	13314	12810	13254	13155
ENSCAFG00845009992	226	242	172	201
ENSCAFG00845007334	173	177	169	166
ENSCAFG00845009997	0	4	0	1
ENSCAFG00845020301	186	185	159	124
ENSCAFG00845022964	0	0	0	0
ENSCAFG00845007333	0	0	0	0
ENSCAFG00845009998	0	0	0	0
ENSCAFG00845020302	1	0	1	0
ENSCAFG00845007336	115	129	111	75

ENSCAFG00845009995	6	12	2	13
ENSCAFG00845022962	0	0	0	0
ENSCAFG00845007335	2643	2485	2743	2685
ENSCAFG00845009996	1714	1563	1807	1717
ENSCAFG00845020300	1517	1516	1476	1440
ENSCAFG00845022961	0	1	0	0
ENSCAFG00845007338	623	614	701	677
ENSCAFG00845007337	2	1	1	0
ENSCAFG00845009999	0	0	0	0
ENSCAFG00845007339	814	819	781	829
ENSCAFG00845010986	0	0	0	0
ENSCAFG00845010987	15	3	7	4
ENSCAFG00845010988	3	2	3	0
ENSCAFG00845010989	92	78	79	95
ENSCAFG00845010982	550	540	642	677
ENSCAFG00845010983	628	589	600	652
ENSCAFG00845010984	273	281	297	314
ENSCAFG00845019310	0	0	0	0
ENSCAFG00845010985	42	32	21	26
ENSCAFG00845019311	0	0	0	0
ENSCAFG00845019312	0	0	0	0
ENSCAFG00845019313	1	0	0	3
ENSCAFG00845010980	3711	3602	3196	3167
ENSCAFG00845019314	0	0	0	0
ENSCAFG00845010981	1	0	1	3
ENSCAFG00845019315	276	213	241	267
ENSCAFG00845019305	1021	1033	907	1002
ENSCAFG00845022957	24	7	12	11
ENSCAFG00845019306	28	31	50	49
ENSCAFG00845022956	0	0	0	0
ENSCAFG00845019307	174	154	139	138
ENSCAFG00845022955	0	0	0	0
ENSCAFG00845019308	0	0	0	0
ENSCAFG00845022954	1827	1749	1712	1807
ENSCAFG00845019309	836	829	849	955
ENSCAFG00845022959	0	0	0	0
ENSCAFG00845022958	0	0	0	0
ENSCAFG00845009982	1074	994	1065	950
ENSCAFG00845009983	0	0	0	0
ENSCAFG00845007321	89	86	62	83
ENSCAFG00845009980	1	0	0	0

ENSCAFG00845007320	1883	1750	1751	1814
ENSCAFG00845009981	210	236	181	236
ENSCAFG00845007323	0	0	0	0
ENSCAFG00845009986	0	0	0	0
ENSCAFG00845022953	2010	1950	1983	1955
ENSCAFG00845007322	0	0	0	0
ENSCAFG00845009987	4	3	6	0
ENSCAFG00845022952	0	0	0	0
ENSCAFG00845007325	35	33	27	36
ENSCAFG00845009984	0	0	0	2
ENSCAFG00845022951	168	184	172	161
ENSCAFG00845007324	0	0	0	0
ENSCAFG00845009985	0	0	0	0
ENSCAFG00845022950	0	1	1	0
ENSCAFG00845007327	33	26	9	15
ENSCAFG00845010979	1761	1644	1659	1623
ENSCAFG00845007326	1	0	1	2
ENSCAFG00845007329	215	271	168	218
ENSCAFG00845009988	0	0	0	0
ENSCAFG00845007328	2	0	3	2
ENSCAFG00845009989	42	32	27	51
ENSCAFG00845010975	126	106	95	115
ENSCAFG00845010976	13192	12259	12855	13885
ENSCAFG00845010977	3316	3436	3481	3542
ENSCAFG00845010978	239	199	211	192
ENSCAFG00845010971	4822	4775	4588	4695
ENSCAFG00845010972	109	102	59	86
ENSCAFG00845010973	0	0	0	0
ENSCAFG00845010974	0	1	0	2
ENSCAFG00845019300	0	0	0	0
ENSCAFG00845019301	0	0	0	0
ENSCAFG00845019302	8	5	11	7
ENSCAFG00845019303	18	15	6	12
ENSCAFG00845010970	0	0	0	0
ENSCAFG00845019304	646	642	601	692
ENSCAFG00845020297	0	0	0	0
ENSCAFG00845020298	6	1	4	4
ENSCAFG00845020295	0	2	1	0
ENSCAFG00845020296	1688	1795	1572	1630
ENSCAFG00845020299	172	168	207	182
ENSCAFG00845020290	978	980	953	986

ENSCAFG00845020293	0	0	0	2
ENSCAFG00845020294	0	0	0	0
ENSCAFG00845020291	0	4	1	3
ENSCAFG00845020292	378	418	404	438
ENSCAFG00845020286	676	655	735	771
ENSCAFG00845020287	25	24	40	35
ENSCAFG00845020284	295	303	259	288
ENSCAFG00845020285	0	0	0	0
ENSCAFG00845020288	2215	2206	1947	2130
ENSCAFG00845020289	8	17	14	18
ENSCAFG00845019290	12	2	7	2
ENSCAFG00845019292	0	0	0	0
ENSCAFG00845019293	0	0	0	0
ENSCAFG00845020282	40	28	20	24
ENSCAFG00845019294	2455	2458	2316	2271
ENSCAFG00845020283	0	0	0	0
ENSCAFG00845019295	560	603	596	614
ENSCAFG00845020280	312	267	279	262
ENSCAFG00845019296	0	0	0	0
ENSCAFG00845020281	3	8	10	2
ENSCAFG00845019297	0	0	0	0
ENSCAFG00845019298	508	503	460	470
ENSCAFG00845019299	908	866	805	818
ENSCAFG00845020275	4173	3904	3929	4102
ENSCAFG00845020276	431	424	428	439
ENSCAFG00845020273	1	5	1	0
ENSCAFG00845020274	0	0	0	0
ENSCAFG00845020279	234	284	218	196
ENSCAFG00845020277	17	10	16	14
ENSCAFG00845020278	33	23	19	24
ENSCAFG00845019280	1	0	0	0
ENSCAFG00845019281	0	0	0	0
ENSCAFG00845019282	1230	1171	1283	1294
ENSCAFG00845020271	0	0	0	0
ENSCAFG00845019283	2	1	3	2
ENSCAFG00845020272	1352	1290	1388	1487
ENSCAFG00845019284	0	0	0	0
ENSCAFG00845019285	2	1	6	1
ENSCAFG00845020270	0	0	0	0
ENSCAFG00845019286	1	1	0	0
ENSCAFG00845019287	1	2	1	2

ENSCAFG00845019288	1	5	2	2
ENSCAFG00845019289	2	0	0	0
ENSCAFG00845007291	0	0	0	0
ENSCAFG00845007290	0	0	0	0
ENSCAFG00845007293	0	0	0	0
ENSCAFG00845007292	1815	1782	1665	1836
ENSCAFG00845007295	1	0	0	3
ENSCAFG00845007294	1186	1168	1051	1088
ENSCAFG00845007297	0	0	0	0
ENSCAFG00845020264	389	364	294	355
ENSCAFG00845007296	527	522	461	424
ENSCAFG00845020265	191	183	256	277
ENSCAFG00845007299	0	0	0	0
ENSCAFG00845020262	0	0	0	0
ENSCAFG00845007298	0	0	0	0
ENSCAFG00845020263	0	0	0	0
ENSCAFG00845020268	0	0	0	0
ENSCAFG00845020269	0	0	0	0
ENSCAFG00845020266	1274	1256	1328	1370
ENSCAFG00845020267	0	0	0	0
ENSCAFG00845019270	0	3	5	3
ENSCAFG00845019271	94	104	96	138
ENSCAFG00845020260	668	604	638	609
ENSCAFG00845019272	0	0	0	0
ENSCAFG00845020261	0	0	0	0
ENSCAFG00845019273	1158	1086	1185	1119
ENSCAFG00845019274	205	244	242	241
ENSCAFG00845019275	50	34	40	36
ENSCAFG00845019276	952	1005	1188	1191
ENSCAFG00845019277	1849	1761	1936	2223
ENSCAFG00845019278	7	5	12	6
ENSCAFG00845019279	0	0	0	0
ENSCAFG00845007280	2104	1818	2011	2086
ENSCAFG00845020259	0	0	0	0
ENSCAFG00845007282	660	752	560	618
ENSCAFG00845007281	0	0	0	0
ENSCAFG00845007284	6	7	0	3
ENSCAFG00845007283	266	261	264	274
ENSCAFG00845007286	0	0	0	0
ENSCAFG00845020253	675	639	694	666
ENSCAFG00845007285	0	0	0	0

ENSCAFG00845020254	0	0	0	0
ENSCAFG00845007288	44	41	55	79
ENSCAFG00845020251	1438	1508	1269	1369
ENSCAFG00845007287	0	0	0	0
ENSCAFG00845020252	2674	2490	2172	2343
ENSCAFG00845020257	0	3	3	0
ENSCAFG00845007289	135	130	150	111
ENSCAFG00845020258	0	1	0	2
ENSCAFG00845020255	1197	1156	1105	1153
ENSCAFG00845020256	572	563	528	481
ENSCAFG00845019260	0	0	0	0
ENSCAFG00845019261	0	0	0	0
ENSCAFG00845020250	0	0	0	0
ENSCAFG00845019262	0	0	0	0
ENSCAFG00845019263	1059	1086	863	827
ENSCAFG00845019264	9	10	17	9
ENSCAFG00845019265	732	747	703	597
ENSCAFG00845019266	396	384	391	392
ENSCAFG00845019267	43	26	43	42
ENSCAFG00845019268	1069	1068	975	989
ENSCAFG00845019269	903	873	917	1083
ENSCAFG00845019415	279	312	271	297
ENSCAFG00845020404	447	437	531	461
ENSCAFG00845019416	769	741	687	747
ENSCAFG00845020405	2	0	11	3
ENSCAFG00845019417	10	22	2	9
ENSCAFG00845020402	0	0	0	0
ENSCAFG00845019418	0	0	0	0
ENSCAFG00845020403	18	25	18	16
ENSCAFG00845019419	0	0	0	0
ENSCAFG00845020408	2808	2695	2634	2537
ENSCAFG00845020409	0	0	0	0
ENSCAFG00845020406	21	19	21	11
ENSCAFG00845020407	721	814	702	723
ENSCAFG00845007431	0	0	0	0
ENSCAFG00845007430	0	0	0	0
ENSCAFG00845007433	0	0	0	0
ENSCAFG00845020400	372	322	425	335
ENSCAFG00845007432	22	26	24	32
ENSCAFG00845020401	0	0	0	0
ENSCAFG00845007435	1	0	0	0

ENSCAFG00845007434	11	10	21	20
ENSCAFG00845007437	1876	1785	1768	1904
ENSCAFG00845007436	0	0	0	0
ENSCAFG00845007439	59	61	70	71
ENSCAFG00845007438	717	651	652	706
ENSCAFG00845019410	32	32	13	7
ENSCAFG00845019411	1	0	0	0
ENSCAFG00845019412	31	31	15	24
ENSCAFG00845019413	0	0	0	0
ENSCAFG00845019414	4	5	2	3
ENSCAFG00845019404	0	0	0	0
ENSCAFG00845019405	0	0	0	0
ENSCAFG00845019406	950	981	971	996
ENSCAFG00845019407	0	0	0	0
ENSCAFG00845019408	3	4	4	4
ENSCAFG00845019409	973	932	945	928
ENSCAFG00845007420	28	25	40	40
ENSCAFG00845007422	0	0	0	1
ENSCAFG00845007421	1042	1038	1072	1088
ENSCAFG00845007424	0	0	0	0
ENSCAFG00845007423	0	0	0	0
ENSCAFG00845007426	722	713	704	663
ENSCAFG00845007425	0	0	0	0
ENSCAFG00845007428	7	3	10	1
ENSCAFG00845007427	0	0	0	0
ENSCAFG00845007429	0	0	0	0
ENSCAFG00845019400	2033	1997	1767	1826
ENSCAFG00845019401	0	0	2	0
ENSCAFG00845019402	97	112	95	102
ENSCAFG00845019403	40	27	31	38
ENSCAFG00845007411	0	0	0	0
ENSCAFG00845007410	0	0	0	0
ENSCAFG00845007413	0	1	0	0
ENSCAFG00845007412	383	353	384	344
ENSCAFG00845007415	2	4	0	3
ENSCAFG00845007414	0	1	5	1
ENSCAFG00845007417	0	0	0	0
ENSCAFG00845007416	0	0	0	0
ENSCAFG00845007419	0	0	0	0
ENSCAFG00845007418	0	2	3	4
ENSCAFG00845007400	0	2	1	2

ENSCAFG00845007402	0	0	0	0
ENSCAFG00845007401	0	0	0	0
ENSCAFG00845007404	0	0	1	0
ENSCAFG00845007403	0	0	0	0
ENSCAFG00845007406	1	0	0	0
ENSCAFG00845007405	661	640	643	696
ENSCAFG00845007408	21	17	22	26
ENSCAFG00845007407	257	230	265	264
ENSCAFG00845007409	0	0	0	0
ENSCAFG00845020484	499	533	497	499
ENSCAFG00845020485	0	0	0	0
ENSCAFG00845020482	381	376	364	373
ENSCAFG00845020483	33	41	54	40
ENSCAFG00845020488	39	44	28	11
ENSCAFG00845020489	1	2	4	1
ENSCAFG00845020486	0	0	0	0
ENSCAFG00845020487	0	0	0	0
ENSCAFG00845019490	146	111	96	91
ENSCAFG00845019491	1571	1391	1432	1517
ENSCAFG00845020480	0	1	0	0
ENSCAFG00845019492	0	0	0	0
ENSCAFG00845020481	1325	1331	1383	1417
ENSCAFG00845019493	0	4	0	0
ENSCAFG00845019494	5	2	4	3
ENSCAFG00845019495	26	39	40	38
ENSCAFG00845019496	690	734	969	937
ENSCAFG00845019497	767	775	804	699
ENSCAFG00845019498	907	928	998	992
ENSCAFG00845019499	0	0	0	0
ENSCAFG00845020479	0	0	0	0
ENSCAFG00845020473	0	0	0	0
ENSCAFG00845020474	0	0	0	0
ENSCAFG00845020471	35	25	22	14
ENSCAFG00845020472	0	0	0	0
ENSCAFG00845020477	40	45	38	50
ENSCAFG00845020478	0	0	0	0
ENSCAFG00845020475	9	10	8	8
ENSCAFG00845020476	33	52	38	41
ENSCAFG00845019480	0	0	0	0
ENSCAFG00845020470	885	883	811	888
ENSCAFG00845019482	0	0	0	0

ENSCAFG00845019483	1376	1362	1198	1323
ENSCAFG00845019484	2	0	1	1
ENSCAFG00845019485	16	18	20	12
ENSCAFG00845019486	473	437	461	463
ENSCAFG00845019487	0	0	0	0
ENSCAFG00845019488	373	332	406	369
ENSCAFG00845019489	32	27	14	13
ENSCAFG00845020468	490	432	361	294
ENSCAFG00845020469	0	0	0	0
ENSCAFG00845007491	0	0	0	0
ENSCAFG00845007490	0	0	0	0
ENSCAFG00845007493	0	0	0	0
ENSCAFG00845007492	47	34	39	35
ENSCAFG00845007495	0	0	0	0
ENSCAFG00845020462	239	215	166	144
ENSCAFG00845007494	519	546	425	401
ENSCAFG00845020463	1196	1140	1314	1248
ENSCAFG00845007497	0	0	0	0
ENSCAFG00845020460	0	0	0	0
ENSCAFG00845007496	0	0	0	0
ENSCAFG00845020461	0	0	1	2
ENSCAFG00845007499	177	165	130	176
ENSCAFG00845020466	1	0	1	0
ENSCAFG00845007498	0	0	0	0
ENSCAFG00845020467	23	23	31	21
ENSCAFG00845020464	12	8	15	6
ENSCAFG00845020465	0	0	0	0
ENSCAFG00845019470	35	37	35	27
ENSCAFG00845019471	2183	2070	1745	1654
ENSCAFG00845019472	0	0	0	0
ENSCAFG00845019473	0	2	0	0
ENSCAFG00845019474	15	14	10	12
ENSCAFG00845019475	1151	1137	1158	1138
ENSCAFG00845019476	920	878	849	897
ENSCAFG00845019477	0	0	1	2
ENSCAFG00845019478	0	0	0	0
ENSCAFG00845019479	0	0	1	0
ENSCAFG00845020459	2305	2342	2395	2338
ENSCAFG00845020457	9	6	10	5
ENSCAFG00845020458	0	1	0	1
ENSCAFG00845007480	5	4	6	4

ENSCAFG00845007482	0	0	0	0
ENSCAFG00845007481	1	0	0	0
ENSCAFG00845007484	725	690	660	675
ENSCAFG00845020451	0	0	0	0
ENSCAFG00845007483	0	0	0	0
ENSCAFG00845020452	0	0	0	0
ENSCAFG00845007486	107	83	100	80
ENSCAFG00845007485	4	0	1	2
ENSCAFG00845020450	0	0	0	0
ENSCAFG00845007488	82	52	87	86
ENSCAFG00845020455	357	331	337	351
ENSCAFG00845007487	0	0	0	0
ENSCAFG00845020456	96	72	60	80
ENSCAFG00845020453	1449	1494	1473	1505
ENSCAFG00845007489	599	590	652	662
ENSCAFG00845020454	0	0	0	0
ENSCAFG00845019460	0	0	0	0
ENSCAFG00845019461	793	733	1132	1087
ENSCAFG00845019462	17	20	19	12
ENSCAFG00845019463	2316	2176	2127	1973
ENSCAFG00845019464	4161	4239	4194	4181
ENSCAFG00845019465	0	0	1	0
ENSCAFG00845019466	0	0	0	0
ENSCAFG00845019467	172	171	164	226
ENSCAFG00845019468	0	0	0	0
ENSCAFG00845019469	822	838	842	839
ENSCAFG00845019459	900	968	816	796
ENSCAFG00845020448	1523	1473	1439	1397
ENSCAFG00845020449	2474	2289	2267	2209
ENSCAFG00845020446	241	206	200	211
ENSCAFG00845020447	409	383	350	367
ENSCAFG00845007471	0	0	0	0
ENSCAFG00845007470	0	0	0	0
ENSCAFG00845007473	0	1	0	0
ENSCAFG00845020440	33	48	36	27
ENSCAFG00845007472	0	0	0	0
ENSCAFG00845020441	3913	3695	3696	3827
ENSCAFG00845007475	2095	2008	1519	1488
ENSCAFG00845007474	2631	2484	2630	2643
ENSCAFG00845007477	0	0	0	0
ENSCAFG00845020444	0	0	0	0

ENSCAFG00845007476	0	0	0	0
ENSCAFG00845020445	0	0	0	0
ENSCAFG00845007479	0	0	0	0
ENSCAFG00845020442	2943	2986	3015	3054
ENSCAFG00845007478	0	0	0	0
ENSCAFG00845020443	675	642	679	656
ENSCAFG00845019450	1	1	0	1
ENSCAFG00845019451	1025	1041	939	959
ENSCAFG00845019452	0	0	0	0
ENSCAFG00845019453	0	0	0	0
ENSCAFG00845019454	0	0	0	0
ENSCAFG00845019455	628	652	649	654
ENSCAFG00845019456	0	0	0	0
ENSCAFG00845019457	0	0	0	0
ENSCAFG00845019458	0	0	0	0
ENSCAFG00845019448	1923	1814	1780	1836
ENSCAFG00845020437	2221	2013	1828	2056
ENSCAFG00845019449	0	5	0	0
ENSCAFG00845020438	1462	1473	1584	1498
ENSCAFG00845020435	0	0	0	1
ENSCAFG00845020436	1	0	0	0
ENSCAFG00845007460	38	47	33	48
ENSCAFG00845020439	0	0	0	0
ENSCAFG00845007462	980	949	965	895
ENSCAFG00845007461	0	0	0	0
ENSCAFG00845020430	11	10	8	11
ENSCAFG00845007464	1384	1263	1403	1265
ENSCAFG00845007463	7	6	0	1
ENSCAFG00845007466	138	136	163	159
ENSCAFG00845020433	558	553	630	692
ENSCAFG00845007465	0	0	0	0
ENSCAFG00845020434	0	0	0	0
ENSCAFG00845007468	0	0	0	0
ENSCAFG00845020431	65	51	50	47
ENSCAFG00845007467	303	319	281	326
ENSCAFG00845020432	863	865	732	749
ENSCAFG00845007469	0	0	0	0
ENSCAFG00845019440	14	11	3	9
ENSCAFG00845019441	1131	1067	1100	1148
ENSCAFG00845019442	1126	1038	1203	1211
ENSCAFG00845019443	60	53	23	27

ENSCAFG00845019444	0	0	0	0
ENSCAFG00845019445	0	0	0	0
ENSCAFG00845019446	812	840	814	833
ENSCAFG00845019447	4	7	4	6
ENSCAFG00845019437	28	30	31	24
ENSCAFG00845020426	976	1048	1150	1067
ENSCAFG00845019438	0	0	0	0
ENSCAFG00845020427	0	0	0	1
ENSCAFG00845019439	0	0	0	0
ENSCAFG00845020424	0	0	0	0
ENSCAFG00845020425	1469	1478	1224	1182
ENSCAFG00845020428	187	192	198	218
ENSCAFG00845020429	5924	5702	6418	6674
ENSCAFG00845007451	0	0	0	0
ENSCAFG00845007450	0	0	0	1
ENSCAFG00845007453	0	0	0	0
ENSCAFG00845007452	0	0	0	1
ENSCAFG00845007455	0	0	0	0
ENSCAFG00845020422	0	0	0	0
ENSCAFG00845007454	6	0	2	1
ENSCAFG00845020423	4630	4393	4694	4680
ENSCAFG00845007457	5	0	2	2
ENSCAFG00845020420	1	0	0	0
ENSCAFG00845007456	0	0	0	0
ENSCAFG00845020421	0	0	0	0
ENSCAFG00845007459	0	0	0	0
ENSCAFG00845007458	1	0	0	0
ENSCAFG00845019430	447	366	397	421
ENSCAFG00845019431	0	0	0	0
ENSCAFG00845019432	0	0	0	0
ENSCAFG00845019433	3	5	2	3
ENSCAFG00845019434	2	0	5	3
ENSCAFG00845019435	20	18	23	21
ENSCAFG00845019436	0	0	0	0
ENSCAFG00845019426	0	0	0	0
ENSCAFG00845020415	691	594	668	661
ENSCAFG00845019427	114	109	95	97
ENSCAFG00845020416	24	28	33	27
ENSCAFG00845019428	339	333	447	406
ENSCAFG00845020413	1211	1254	1222	1215
ENSCAFG00845019429	0	0	0	0

ENSCAFG00845020414	1	0	1	1
ENSCAFG00845020419	51	56	23	30
ENSCAFG00845020417	1	5	5	4
ENSCAFG00845020418	1266	1328	1228	1247
ENSCAFG00845007440	38	42	43	40
ENSCAFG00845007442	0	0	0	0
ENSCAFG00845007441	0	0	0	0
ENSCAFG00845007444	20	17	16	7
ENSCAFG00845020411	2757	2702	2843	2911
ENSCAFG00845007443	72	81	92	126
ENSCAFG00845020412	0	0	0	0
ENSCAFG00845007446	0	0	0	0
ENSCAFG00845007445	0	0	0	0
ENSCAFG00845020410	0	0	0	0
ENSCAFG00845007448	0	0	0	0
ENSCAFG00845007447	1937	1812	1737	1796
ENSCAFG00845007449	0	0	0	0
ENSCAFG00845019420	212	199	177	176
ENSCAFG00845019421	244	241	276	289
ENSCAFG00845019422	0	0	0	0
ENSCAFG00845019423	5	3	0	1
ENSCAFG00845019424	33	42	25	28
ENSCAFG00845019425	1061	1041	948	1001
ENSCAFG00845020396	105	115	164	124
ENSCAFG00845020397	0	0	0	0
ENSCAFG00845020394	0	0	0	0
ENSCAFG00845020395	193	172	215	183
ENSCAFG00845020398	1	3	3	0
ENSCAFG00845020399	1237	1143	1199	1141
ENSCAFG00845020392	86	101	75	122
ENSCAFG00845020393	598	550	525	524
ENSCAFG00845020390	0	0	0	0
ENSCAFG00845020391	640	602	526	485
ENSCAFG00845020385	64	58	55	50
ENSCAFG00845020386	360	353	364	405
ENSCAFG00845020383	0	0	0	0
ENSCAFG00845020389	1889	1885	1870	1984
ENSCAFG00845020387	193	148	186	146
ENSCAFG00845020388	215	197	182	213
ENSCAFG00845019390	17	16	14	16
ENSCAFG00845019391	1	0	0	0

ENSCAFG00845019392	994	926	1029	1013
ENSCAFG00845020381	13	12	1	2
ENSCAFG00845019393	4281	4013	3945	4142
ENSCAFG00845020382	1	1	0	3
ENSCAFG00845019394	0	0	0	0
ENSCAFG00845019395	2	5	5	4
ENSCAFG00845020380	4	7	6	5
ENSCAFG00845019396	0	0	0	0
ENSCAFG00845019397	0	0	0	0
ENSCAFG00845019398	0	1	4	0
ENSCAFG00845019399	0	0	0	0
ENSCAFG00845020374	341	369	380	379
ENSCAFG00845020375	291	256	260	269
ENSCAFG00845020372	104	109	102	106
ENSCAFG00845020373	341	355	344	371
ENSCAFG00845020378	312	332	319	353
ENSCAFG00845020379	0	0	0	0
ENSCAFG00845020376	0	0	0	0
ENSCAFG00845020377	0	0	0	0
ENSCAFG00845019380	1743	1675	2076	2005
ENSCAFG00845019381	825	754	786	785
ENSCAFG00845020370	0	0	0	0
ENSCAFG00845019382	2018	1762	1572	1660
ENSCAFG00845020371	10	11	6	8
ENSCAFG00845019383	0	0	5	0
ENSCAFG00845019384	0	0	0	0
ENSCAFG00845019385	133	110	103	143
ENSCAFG00845019386	0	1	1	0
ENSCAFG00845019387	0	0	0	0
ENSCAFG00845019388	17	17	15	15
ENSCAFG00845019389	0	4	1	2
ENSCAFG00845000916	0	1	0	2
ENSCAFG00845000917	18	12	10	12
ENSCAFG00845000914	0	0	0	0
ENSCAFG00845000915	1266	1116	1629	1530
ENSCAFG00845000912	357	333	292	326
ENSCAFG00845000913	15	12	7	6
ENSCAFG00845000910	1439	1532	1688	1685
ENSCAFG00845000911	0	0	1	0
ENSCAFG00845012905	510	448	559	517
ENSCAFG00845012904	139	153	143	166

ENSCAFG00845012907	0	1	0	0
ENSCAFG00845012906	101	86	82	108
ENSCAFG00845012901	132	133	140	152
ENSCAFG00845012900	502	453	469	536
ENSCAFG00845012903	1	0	0	2
ENSCAFG00845012902	0	0	0	0
ENSCAFG00845000909	2075	2148	1924	2048
ENSCAFG00845000907	0	1	2	1
ENSCAFG00845000908	0	5	2	8
ENSCAFG00845000927	195	147	182	212
ENSCAFG00845000928	0	0	0	0
ENSCAFG00845000925	26	24	15	31
ENSCAFG00845000926	646	699	850	889
ENSCAFG00845000923	1212	1051	1105	1108
ENSCAFG00845000924	4294	4223	4188	4326
ENSCAFG00845000921	197	174	188	205
ENSCAFG00845000922	8	8	3	6
ENSCAFG00845000920	0	0	0	0
ENSCAFG00845000918	123	115	79	99
ENSCAFG00845000919	0	0	0	0
ENSCAFG00845000905	0	0	2	0
ENSCAFG00845000906	0	0	0	0
ENSCAFG00845000903	9	22	19	21
ENSCAFG00845000904	0	1	3	0
ENSCAFG00845000901	118	117	116	136
ENSCAFG00845000902	2552	2488	2582	2631
ENSCAFG00845000900	71	65	59	61
ENSCAFG00845022308	15	25	11	21
ENSCAFG00845022307	1040	982	987	969
ENSCAFG00845022306	0	0	0	0
ENSCAFG00845022305	1592	1585	1810	1730
ENSCAFG00845024967	446	374	410	462
ENSCAFG00845009330	0	0	0	0
ENSCAFG00845024968	0	0	0	0
ENSCAFG00845000998	0	0	0	0
ENSCAFG00845024969	257	218	270	264
ENSCAFG00845000999	0	3	0	2
ENSCAFG00845022309	0	0	0	0
ENSCAFG00845000996	16	4	6	7
ENSCAFG00845009333	2	3	8	9
ENSCAFG00845022300	0	0	0	0

ENSCAFG00845024963	561	525	490	490
ENSCAFG00845000997	1	0	0	0
ENSCAFG00845009334	0	0	1	0
ENSCAFG00845024964	0	0	0	0
ENSCAFG00845000994	0	1	0	0
ENSCAFG00845009331	0	0	0	0
ENSCAFG00845024965	1	0	1	0
ENSCAFG00845000995	1279	1322	1166	1161
ENSCAFG00845009332	0	0	0	0
ENSCAFG00845024966	24	23	22	27
ENSCAFG00845000992	0	0	0	0
ENSCAFG00845009337	0	0	2	0
ENSCAFG00845022304	0	0	0	0
ENSCAFG00845000993	501	464	443	480
ENSCAFG00845009338	1182	1185	997	1057
ENSCAFG00845022303	619	676	473	584
ENSCAFG00845024960	14	8	15	10
ENSCAFG00845000990	216	203	195	197
ENSCAFG00845009335	0	2	2	0
ENSCAFG00845022302	0	0	2	0
ENSCAFG00845024961	175	140	146	124
ENSCAFG00845000991	4256	4193	4368	4202
ENSCAFG00845009336	40	39	33	38
ENSCAFG00845022301	31	34	22	22
ENSCAFG00845024962	0	0	0	0
ENSCAFG00845009339	0	0	0	0
ENSCAFG00845010326	1800	1636	1696	1820
ENSCAFG00845012989	670	606	663	593
ENSCAFG00845010327	0	0	0	0
ENSCAFG00845012988	0	0	0	0
ENSCAFG00845010328	1331	1274	1130	1229
ENSCAFG00845010329	414	439	351	347
ENSCAFG00845010322	0	0	0	0
ENSCAFG00845012985	16468	15645	16927	17184
ENSCAFG00845010323	51	40	45	48
ENSCAFG00845012984	6384	6098	5854	5833
ENSCAFG00845010324	0	0	0	0
ENSCAFG00845012987	1	1	0	4
ENSCAFG00845010325	461	405	443	491
ENSCAFG00845012986	0	0	0	0
ENSCAFG00845012981	0	0	0	0

ENSCAFG00845012980	460	405	490	441
ENSCAFG00845010320	213	203	238	202
ENSCAFG00845012983	5	6	13	12
ENSCAFG00845010321	61	77	34	29
ENSCAFG00845012982	151	142	154	138
ENSCAFG00845024956	529	478	520	540
ENSCAFG00845024957	0	0	0	0
ENSCAFG00845024958	16	13	5	19
ENSCAFG00845024959	0	0	0	0
ENSCAFG00845009322	1391	1283	1288	1290
ENSCAFG00845024952	200	242	193	175
ENSCAFG00845009323	40	47	25	34
ENSCAFG00845024953	74	70	57	55
ENSCAFG00845009320	0	0	0	0
ENSCAFG00845009321	1633	1601	1534	1499
ENSCAFG00845024955	122	111	110	112
ENSCAFG00845009326	529	486	518	514
ENSCAFG00845009327	0	0	0	0
ENSCAFG00845009324	0	0	0	0
ENSCAFG00845024950	0	0	0	0
ENSCAFG00845009325	0	0	0	0
ENSCAFG00845024951	0	0	0	0
ENSCAFG00845010319	0	1	1	6
ENSCAFG00845009328	304	335	353	318
ENSCAFG00845009329	792	648	720	822
ENSCAFG00845010315	4	2	3	3
ENSCAFG00845012978	6	4	2	7
ENSCAFG00845010316	3	2	1	1
ENSCAFG00845012977	0	0	0	0
ENSCAFG00845010317	1553	1486	1274	1336
ENSCAFG00845010318	2	2	2	0
ENSCAFG00845012979	0	0	0	0
ENSCAFG00845010311	4	5	3	1
ENSCAFG00845012974	0	0	0	0
ENSCAFG00845010312	735	758	641	709
ENSCAFG00845012973	228	221	268	306
ENSCAFG00845010313	7361	7077	8359	8328
ENSCAFG00845012976	2	0	1	1
ENSCAFG00845010314	474	434	467	424
ENSCAFG00845012975	0	0	0	0
ENSCAFG00845012970	0	0	0	0

ENSCAFG00845012972	51	44	16	12
ENSCAFG00845010310	0	0	0	0
ENSCAFG00845012971	505	445	463	421
ENSCAFG00845024949	1	1	0	0
ENSCAFG00845000978	820	844	841	816
ENSCAFG00845024945	10	5	6	11
ENSCAFG00845000979	0	0	0	0
ENSCAFG00845024946	6974	6712	6247	6148
ENSCAFG00845000976	202	255	156	157
ENSCAFG00845024947	1	3	1	4
ENSCAFG00845000977	576	513	470	526
ENSCAFG00845024948	7	7	4	6
ENSCAFG00845000974	0	0	0	0
ENSCAFG00845009311	0	0	0	0
ENSCAFG00845024941	0	0	0	0
ENSCAFG00845000975	10	5	1	5
ENSCAFG00845009312	0	0	0	0
ENSCAFG00845024942	0	0	0	0
ENSCAFG00845000972	552	563	519	651
ENSCAFG00845024943	37	34	32	32
ENSCAFG00845000973	436	488	443	423
ENSCAFG00845009310	650	700	629	607
ENSCAFG00845024944	7	7	9	3
ENSCAFG00845000970	101	87	95	77
ENSCAFG00845009315	93	79	147	150
ENSCAFG00845000971	1	4	2	2
ENSCAFG00845009316	0	0	0	0
ENSCAFG00845009313	6	1	3	7
ENSCAFG00845009314	957	872	880	807
ENSCAFG00845024940	0	0	0	0
ENSCAFG00845009319	0	0	0	0
ENSCAFG00845010308	0	0	0	0
ENSCAFG00845010309	0	0	0	0
ENSCAFG00845009317	0	0	0	0
ENSCAFG00845009318	0	0	0	0
ENSCAFG00845010304	11	3	4	7
ENSCAFG00845012967	4117	4069	4039	4187
ENSCAFG00845010305	2968	2836	2958	3049
ENSCAFG00845012966	2650	2566	2516	2610
ENSCAFG00845010306	36	40	46	55
ENSCAFG00845012969	4	3	3	4

ENSCAFG00845010307	286	309	354	348
ENSCAFG00845012968	1163	1020	924	930
ENSCAFG00845010300	908	953	986	1033
ENSCAFG00845012963	0	0	0	0
ENSCAFG00845010301	0	4	1	1
ENSCAFG00845012962	0	0	0	3
ENSCAFG00845010302	439	433	415	458
ENSCAFG00845012965	21	11	18	16
ENSCAFG00845010303	45	57	61	58
ENSCAFG00845012964	535	462	537	544
ENSCAFG00845012961	0	0	0	0
ENSCAFG00845012960	885	959	874	891
ENSCAFG00845024938	0	2	4	2
ENSCAFG00845024939	306	292	244	261
ENSCAFG00845000989	25	36	21	23
ENSCAFG00845024934	3	0	2	3
ENSCAFG00845024935	3	2	0	2
ENSCAFG00845000987	0	0	0	0
ENSCAFG00845024936	24	23	18	20
ENSCAFG00845000988	0	0	0	0
ENSCAFG00845024937	1	0	0	0
ENSCAFG00845000985	0	0	0	0
ENSCAFG00845009300	0	2	1	1
ENSCAFG00845024930	306	276	398	372
ENSCAFG00845000986	5	4	3	5
ENSCAFG00845009301	6	1	7	8
ENSCAFG00845024931	607	603	533	573
ENSCAFG00845000983	43	18	20	21
ENSCAFG00845024932	1125	980	941	1052
ENSCAFG00845000984	2	1	3	0
ENSCAFG00845024933	0	0	0	0
ENSCAFG00845000981	1390	1313	1317	1300
ENSCAFG00845009304	0	0	0	0
ENSCAFG00845000982	277	315	349	332
ENSCAFG00845009305	832	823	836	830
ENSCAFG00845009302	0	0	0	0
ENSCAFG00845000980	642	522	568	523
ENSCAFG00845009303	654	643	637	598
ENSCAFG00845009308	751	777	763	777
ENSCAFG00845009309	0	0	0	0
ENSCAFG00845012959	0	3	0	0

ENSCAFG00845009306	0	0	0	0
ENSCAFG00845009307	0	0	0	0
ENSCAFG00845012956	2	0	0	0
ENSCAFG00845012955	1	4	3	3
ENSCAFG00845012958	0	0	0	0
ENSCAFG00845012957	0	0	0	0
ENSCAFG00845012952	574	524	443	496
ENSCAFG00845012951	0	0	1	1
ENSCAFG00845012954	17	13	9	13
ENSCAFG00845012953	0	0	0	0
ENSCAFG00845012950	182	165	128	147
ENSCAFG00845024927	56	28	27	15
ENSCAFG00845024928	16	26	25	20
ENSCAFG00845000958	20	14	22	20
ENSCAFG00845024929	3	5	1	1
ENSCAFG00845000959	2	0	0	0
ENSCAFG00845000956	2	1	1	0
ENSCAFG00845024923	0	0	0	0
ENSCAFG00845000957	0	0	0	0
ENSCAFG00845024924	820	945	877	970
ENSCAFG00845000954	0	0	0	0
ENSCAFG00845024925	0	0	0	0
ENSCAFG00845000955	0	0	0	0
ENSCAFG00845024926	0	0	0	0
ENSCAFG00845000952	1	5	3	5
ENSCAFG00845000953	719	735	1026	987
ENSCAFG00845024920	35	27	26	33
ENSCAFG00845000950	0	0	0	0
ENSCAFG00845000951	0	0	0	0
ENSCAFG00845024922	16	5	11	11
ENSCAFG00845012949	1550	1594	1659	1568
ENSCAFG00845012948	70	65	91	81
ENSCAFG00845012945	383	363	334	390
ENSCAFG00845012944	4387	3975	3805	4021
ENSCAFG00845012947	0	0	0	0
ENSCAFG00845012946	1	0	0	0
ENSCAFG00845012941	0	0	0	0
ENSCAFG00845012940	664	585	584	571
ENSCAFG00845012943	0	0	0	0
ENSCAFG00845012942	14	9	33	21
ENSCAFG00845024916	16	18	20	35

ENSCAFG00845024917	0	0	0	0
ENSCAFG00845000969	14	18	25	22
ENSCAFG00845024918	0	0	0	0
ENSCAFG00845024919	2473	2365	1301	1359
ENSCAFG00845000967	0	0	0	0
ENSCAFG00845024912	1146	1210	1000	1119
ENSCAFG00845000968	21	32	13	35
ENSCAFG00845024913	1	0	4	0
ENSCAFG00845000965	3	0	0	3
ENSCAFG00845024914	0	0	0	0
ENSCAFG00845000966	0	0	0	0
ENSCAFG00845024915	1	0	0	2
ENSCAFG00845000963	29	31	21	22
ENSCAFG00845000964	77	88	78	67
ENSCAFG00845000961	9	2	3	3
ENSCAFG00845024910	1001	1009	1012	914
ENSCAFG00845000962	4	2	1	4
ENSCAFG00845024911	1179	1186	1152	1294
ENSCAFG00845000960	0	0	0	0
ENSCAFG00845012938	0	0	0	0
ENSCAFG00845012937	400	369	472	517
ENSCAFG00845012939	1212	1230	1027	1264
ENSCAFG00845012934	0	0	0	0
ENSCAFG00845012933	1635	1525	1425	1340
ENSCAFG00845012936	76	60	96	76
ENSCAFG00845012935	121	91	108	90
ENSCAFG00845012930	0	0	0	0
ENSCAFG00845012932	413	436	390	404
ENSCAFG00845012931	0	0	0	0
ENSCAFG00845024909	0	0	0	3
ENSCAFG00845000938	0	0	0	0
ENSCAFG00845024905	7	12	5	13
ENSCAFG00845000939	0	0	0	1
ENSCAFG00845024906	7	3	3	0
ENSCAFG00845000936	9	6	4	2
ENSCAFG00845024907	0	0	0	0
ENSCAFG00845000937	100	67	59	80
ENSCAFG00845024908	29	17	29	26
ENSCAFG00845000934	350	294	285	327
ENSCAFG00845024901	0	0	0	0
ENSCAFG00845000935	0	0	1	0

ENSCAFG00845024902	1	3	1	9
ENSCAFG00845000932	188	154	154	159
ENSCAFG00845024903	360	333	337	342
ENSCAFG00845000933	0	0	0	0
ENSCAFG00845024904	13	11	12	3
ENSCAFG00845000930	20	20	30	22
ENSCAFG00845000931	0	0	0	0
ENSCAFG00845024900	33	18	27	37
ENSCAFG00845012919	0	0	0	0
ENSCAFG00845012927	18	12	16	21
ENSCAFG00845012926	0	0	0	0
ENSCAFG00845012929	123	104	106	104
ENSCAFG00845012928	30	38	63	52
ENSCAFG00845012923	0	2	0	0
ENSCAFG00845012922	961	857	857	863
ENSCAFG00845012925	467	448	397	345
ENSCAFG00845012924	704	715	577	612
ENSCAFG00845012921	33	33	42	39
ENSCAFG00845012920	43	38	34	35
ENSCAFG00845000929	99	92	80	75
ENSCAFG00845000949	10	3	9	1
ENSCAFG00845000947	422	473	391	366
ENSCAFG00845000948	0	0	0	0
ENSCAFG00845000945	1	1	1	1
ENSCAFG00845000946	1	0	0	0
ENSCAFG00845000943	57	88	77	66
ENSCAFG00845000944	302	319	327	323
ENSCAFG00845000941	40	34	12	27
ENSCAFG00845000942	4	0	0	0
ENSCAFG00845000940	0	0	0	0
ENSCAFG00845012909	118	120	138	92
ENSCAFG00845012908	148	189	211	190
ENSCAFG00845012916	1228	1246	1182	1199
ENSCAFG00845012915	1849	1812	1934	1879
ENSCAFG00845012918	0	0	0	0
ENSCAFG00845012917	1209	1142	1126	1084
ENSCAFG00845012912	3	1	0	0
ENSCAFG00845012911	0	0	0	0
ENSCAFG00845012914	63	92	73	98
ENSCAFG00845012913	0	1	0	1
ENSCAFG00845012910	51	69	39	53

ENSCAFG00845009292	0	0	0	0
ENSCAFG00845010281	218	234	172	166
ENSCAFG00845009293	31	37	41	27
ENSCAFG00845010282	447	445	412	365
ENSCAFG00845009290	0	0	0	0
ENSCAFG00845010283	450	378	376	388
ENSCAFG00845009291	0	0	0	0
ENSCAFG00845010284	5	8	5	7
ENSCAFG00845009296	0	0	0	0
ENSCAFG00845009297	13339	12851	13059	13170
ENSCAFG00845009294	0	0	0	0
ENSCAFG00845009295	0	0	0	0
ENSCAFG00845010280	787	814	877	932
ENSCAFG00845022267	0	0	0	0
ENSCAFG00845022266	4	1	0	4
ENSCAFG00845009298	615	581	454	504
ENSCAFG00845022265	80	83	68	53
ENSCAFG00845009299	47	39	34	25
ENSCAFG00845022264	164	170	159	187
ENSCAFG00845022269	949	886	1026	888
ENSCAFG00845022268	893	863	897	874
ENSCAFG00845022263	0	0	0	0
ENSCAFG00845022262	510	480	482	434
ENSCAFG00845022261	12	18	11	13
ENSCAFG00845022260	0	0	3	10
ENSCAFG00845010289	1	0	1	0
ENSCAFG00845010285	0	2	2	1
ENSCAFG00845010286	6	13	8	7
ENSCAFG00845010287	1030	1003	1004	991
ENSCAFG00845010288	916	886	958	876
ENSCAFG00845009281	0	0	0	0
ENSCAFG00845010270	163	184	132	136
ENSCAFG00845009282	1	2	2	0
ENSCAFG00845010271	0	0	0	0
ENSCAFG00845010272	306	235	240	246
ENSCAFG00845009280	170	150	179	196
ENSCAFG00845010273	0	0	0	0
ENSCAFG00845009285	0	0	0	0
ENSCAFG00845009286	8	4	7	5
ENSCAFG00845009283	369	383	395	403
ENSCAFG00845009284	33	35	18	35

ENSCAFG00845009289	0	0	1	2
ENSCAFG00845022256	10	11	19	21
ENSCAFG00845022255	3262	3031	3176	3368
ENSCAFG00845009287	0	0	0	0
ENSCAFG00845022254	0	0	0	0
ENSCAFG00845009288	0	0	0	0
ENSCAFG00845022253	44	37	35	33
ENSCAFG00845022259	298	308	308	363
ENSCAFG00845022258	142	121	108	145
ENSCAFG00845022257	0	0	1	0
ENSCAFG00845022251	55	52	47	39
ENSCAFG00845022250	6840	6730	6191	6310
ENSCAFG00845010278	6205	5987	6176	6429
ENSCAFG00845010279	11	5	11	9
ENSCAFG00845010274	0	0	0	0
ENSCAFG00845010275	0	0	0	0
ENSCAFG00845010276	1372	1310	1316	1311
ENSCAFG00845010277	0	0	0	0
ENSCAFG00845009270	2951	2796	2700	2820
ENSCAFG00845009271	2	0	0	0
ENSCAFG00845010260	0	3	0	2
ENSCAFG00845010261	813	839	774	765
ENSCAFG00845010262	395	386	369	342
ENSCAFG00845009274	38	33	41	27
ENSCAFG00845009275	0	0	0	0
ENSCAFG00845009272	0	0	0	0
ENSCAFG00845009273	1279	1240	1469	1528
ENSCAFG00845009278	73	55	47	46
ENSCAFG00845022245	0	0	0	0
ENSCAFG00845009279	0	0	0	0
ENSCAFG00845022244	147	98	148	140
ENSCAFG00845009276	3	0	1	1
ENSCAFG00845022243	678	592	544	644
ENSCAFG00845009277	0	0	0	0
ENSCAFG00845022242	0	0	0	0
ENSCAFG00845022249	0	0	0	0
ENSCAFG00845022248	0	0	0	0
ENSCAFG00845022247	1	0	0	0
ENSCAFG00845022246	11	11	5	13
ENSCAFG00845022241	698	689	592	551
ENSCAFG00845022240	32	18	28	29

ENSCAFG00845010267	1132	1057	1166	1290
ENSCAFG00845010268	1	1	0	0
ENSCAFG00845010269	2369	2321	2088	2133
ENSCAFG00845010263	2422	2227	2075	2237
ENSCAFG00845010264	0	0	0	0
ENSCAFG00845010265	14	7	5	8
ENSCAFG00845010266	2	3	2	4
ENSCAFG00845009260	0	2	1	0
ENSCAFG00845010250	5	4	0	0
ENSCAFG00845010251	0	0	0	1
ENSCAFG00845022239	0	0	0	0
ENSCAFG00845009263	0	1	0	0
ENSCAFG00845009264	0	0	0	0
ENSCAFG00845009261	5	9	11	6
ENSCAFG00845009262	14709	14477	14507	14226
ENSCAFG00845009267	415	412	425	441
ENSCAFG00845022234	1	0	0	1
ENSCAFG00845024897	4	16	12	12
ENSCAFG00845009268	0	0	0	2
ENSCAFG00845022233	0	0	0	0
ENSCAFG00845009265	0	0	0	0
ENSCAFG00845022232	381	378	97	108
ENSCAFG00845024899	8	5	6	9
ENSCAFG00845009266	0	0	0	0
ENSCAFG00845022231	582	573	637	629
ENSCAFG00845022238	13	11	7	2
ENSCAFG00845024893	0	0	0	0
ENSCAFG00845022237	0	0	0	0
ENSCAFG00845024894	63	56	43	34
ENSCAFG00845009269	734	683	650	640
ENSCAFG00845022236	1126	1020	1045	1042
ENSCAFG00845024895	9	4	4	6
ENSCAFG00845022235	1520	1467	1392	1483
ENSCAFG00845024896	1	0	0	0
ENSCAFG00845024890	498	498	538	591
ENSCAFG00845024891	1	0	0	1
ENSCAFG00845024892	0	0	0	0
ENSCAFG00845022230	0	0	0	0
ENSCAFG00845010256	0	3	0	0
ENSCAFG00845010257	4	2	0	1
ENSCAFG00845010258	1088	999	1078	1060

ENSCAFG00845010259	267	252	275	288
ENSCAFG00845010252	26	24	20	4
ENSCAFG00845010253	4	1	0	4
ENSCAFG00845010254	1774	1723	1818	1723
ENSCAFG00845010255	5125	5001	4941	5175
ENSCAFG00845010240	2045	1783	1776	1790
ENSCAFG00845022228	0	0	0	0
ENSCAFG00845009252	276	261	241	248
ENSCAFG00845009253	7	1	0	1
ENSCAFG00845009250	905	904	870	860
ENSCAFG00845009251	224	229	272	235
ENSCAFG00845009256	0	0	0	0
ENSCAFG00845022223	1778	1635	1924	1865
ENSCAFG00845024886	1614	1590	1602	1568
ENSCAFG00845009257	807	811	865	898
ENSCAFG00845022222	10261	9859	10730	10629
ENSCAFG00845024887	73	69	95	84
ENSCAFG00845009254	0	0	0	0
ENSCAFG00845022221	0	2	0	0
ENSCAFG00845024888	0	0	0	0
ENSCAFG00845009255	0	0	0	0
ENSCAFG00845022220	0	0	0	0
ENSCAFG00845024889	662	553	558	494
ENSCAFG00845022227	125	135	141	124
ENSCAFG00845024882	31	22	22	26
ENSCAFG00845022226	848	859	705	746
ENSCAFG00845024883	226	213	230	267
ENSCAFG00845009258	15786	15567	15299	15480
ENSCAFG00845022225	0	0	0	0
ENSCAFG00845024884	0	0	0	0
ENSCAFG00845009259	0	0	0	0
ENSCAFG00845022224	0	3	0	1
ENSCAFG00845024885	6	3	6	2
ENSCAFG00845024880	1	5	0	1
ENSCAFG00845024881	0	0	0	0
ENSCAFG00845010249	0	0	0	0
ENSCAFG00845010245	0	0	1	0
ENSCAFG00845010246	541	468	533	550
ENSCAFG00845010247	0	0	0	0
ENSCAFG00845010248	0	0	0	0
ENSCAFG00845010241	0	0	0	0

ENSCAFG00845010242	0	4	0	2
ENSCAFG00845010243	769	774	686	675
ENSCAFG00845010244	0	0	1	1
ENSCAFG00845022219	6	6	3	6
ENSCAFG00845012891	0	0	0	0
ENSCAFG00845022218	0	0	0	0
ENSCAFG00845012890	0	2	10	0
ENSCAFG00845022217	2222	2117	2099	2057
ENSCAFG00845009241	414	413	367	374
ENSCAFG00845009242	0	0	0	0
ENSCAFG00845009240	87	75	83	49
ENSCAFG00845009245	4	8	4	9
ENSCAFG00845022212	0	0	0	0
ENSCAFG00845024875	125	116	99	109
ENSCAFG00845009246	142	157	152	172
ENSCAFG00845022211	2	0	4	2
ENSCAFG00845024876	18	16	21	12
ENSCAFG00845009243	1093	1045	1090	1087
ENSCAFG00845022210	85	76	73	95
ENSCAFG00845024877	544	454	468	492
ENSCAFG00845009244	1768	1611	1487	1513
ENSCAFG00845024878	0	0	0	0
ENSCAFG00845009249	0	0	0	0
ENSCAFG00845022216	31	22	27	18
ENSCAFG00845024871	109	98	126	152
ENSCAFG00845022215	0	0	0	0
ENSCAFG00845024872	4	3	7	2
ENSCAFG00845009247	0	0	0	0
ENSCAFG00845022214	1298	1321	1313	1259
ENSCAFG00845024873	0	0	0	0
ENSCAFG00845009248	799	712	793	858
ENSCAFG00845022213	84	82	71	85
ENSCAFG00845024874	0	0	0	0
ENSCAFG00845024870	621	516	550	602
ENSCAFG00845010238	1320	1348	888	917
ENSCAFG00845010239	0	0	0	0
ENSCAFG00845010234	1	5	3	0
ENSCAFG00845012897	54	44	57	38
ENSCAFG00845010235	445	415	438	443
ENSCAFG00845012896	319	318	368	369
ENSCAFG00845010236	3873	3736	3544	3389

ENSCAFG00845012899	508	480	445	501
ENSCAFG00845010237	3	0	1	0
ENSCAFG00845012898	689	660	728	732
ENSCAFG00845010230	0	0	2	0
ENSCAFG00845012893	224	235	239	236
ENSCAFG00845010231	603	547	566	616
ENSCAFG00845012892	0	0	0	0
ENSCAFG00845010232	0	0	0	0
ENSCAFG00845012895	1084	989	916	906
ENSCAFG00845010233	0	0	0	0
ENSCAFG00845012894	0	0	0	0
ENSCAFG00845022209	0	0	0	0
ENSCAFG00845022208	818	753	797	816
ENSCAFG00845012880	0	0	0	0
ENSCAFG00845022206	4175	3899	3949	4224
ENSCAFG00845009230	817	700	589	649
ENSCAFG00845024868	1975	1788	1775	1787
ENSCAFG00845009231	0	0	0	0
ENSCAFG00845000899	0	0	2	0
ENSCAFG00845000897	0	0	0	0
ENSCAFG00845009234	474	381	392	434
ENSCAFG00845024864	208	192	220	199
ENSCAFG00845000898	10	8	8	5
ENSCAFG00845009235	2	0	2	1
ENSCAFG00845022200	1475	1330	1594	1613
ENSCAFG00845024865	0	0	0	0
ENSCAFG00845000895	14	23	16	8
ENSCAFG00845009232	254	217	215	219
ENSCAFG00845024866	2	2	2	0
ENSCAFG00845000896	4822	5062	4674	4911
ENSCAFG00845009233	13164	12903	11687	11463
ENSCAFG00845024867	515	493	580	517
ENSCAFG00845000893	812	779	755	825
ENSCAFG00845009238	60	61	59	79
ENSCAFG00845022205	119	127	114	111
ENSCAFG00845024860	0	0	0	0
ENSCAFG00845000894	0	0	1	1
ENSCAFG00845009239	0	0	0	0
ENSCAFG00845022204	0	0	0	0
ENSCAFG00845024861	13	9	17	13
ENSCAFG00845000891	1446	1419	1337	1251

ENSCAFG00845009236	0	0	0	0
ENSCAFG00845022203	0	0	0	0
ENSCAFG00845024862	1	1	0	1
ENSCAFG00845000892	550	553	635	637
ENSCAFG00845009237	1	0	2	0
ENSCAFG00845022202	1	0	1	1
ENSCAFG00845024863	58	61	17	17
ENSCAFG00845000890	0	0	0	0
ENSCAFG00845010227	31	19	34	25
ENSCAFG00845010228	1	2	0	3
ENSCAFG00845012889	0	0	0	0
ENSCAFG00845010229	251	267	265	217
ENSCAFG00845010223	184	183	119	124
ENSCAFG00845012886	603	562	607	620
ENSCAFG00845010224	0	2	3	1
ENSCAFG00845012885	0	0	0	0
ENSCAFG00845010225	146	107	150	149
ENSCAFG00845012888	51	37	32	44
ENSCAFG00845010226	0	0	0	0
ENSCAFG00845012887	27291	26573	28229	29493
ENSCAFG00845012882	3	0	0	0
ENSCAFG00845010220	771	739	654	706
ENSCAFG00845012881	407	358	365	337
ENSCAFG00845010221	0	1	0	0
ENSCAFG00845012884	0	0	0	0
ENSCAFG00845010222	658	716	737	703
ENSCAFG00845012883	0	0	2	0
ENSCAFG00845024857	0	0	0	0
ENSCAFG00845009220	0	0	0	0
ENSCAFG00845024858	1	0	0	0
ENSCAFG00845024859	974	907	1085	1141
ENSCAFG00845009223	93	86	69	103
ENSCAFG00845024853	801	812	713	741
ENSCAFG00845009224	420	439	373	413
ENSCAFG00845024854	851	775	800	750
ENSCAFG00845009221	4	6	1	3
ENSCAFG00845024855	1	0	0	0
ENSCAFG00845009222	464	374	425	388
ENSCAFG00845024856	69	85	82	91
ENSCAFG00845009227	0	0	0	0
ENSCAFG00845009228	0	0	0	0

ENSCAFG00845024850	1766	1694	1631	1573
ENSCAFG00845009225	0	0	2	0
ENSCAFG00845024851	87	95	99	91
ENSCAFG00845009226	717	735	693	706
ENSCAFG00845024852	2503	2463	2613	2311
ENSCAFG00845009229	0	0	0	0
ENSCAFG00845010216	8	7	7	6
ENSCAFG00845012879	511	439	427	476
ENSCAFG00845010217	468	431	399	489
ENSCAFG00845012878	829	810	745	778
ENSCAFG00845010218	1180	1145	1053	1067
ENSCAFG00845010219	745	729	787	784
ENSCAFG00845010212	0	0	0	0
ENSCAFG00845012875	0	0	0	0
ENSCAFG00845010213	0	0	0	0
ENSCAFG00845012874	0	0	0	0
ENSCAFG00845010214	0	0	0	2
ENSCAFG00845012877	1	0	0	0
ENSCAFG00845010215	52	57	70	71
ENSCAFG00845012876	0	0	0	0
ENSCAFG00845012871	855	806	856	804
ENSCAFG00845012870	503	489	464	475
ENSCAFG00845010210	0	0	0	0
ENSCAFG00845012873	833	819	703	660
ENSCAFG00845010211	268	249	237	251
ENSCAFG00845012872	222	217	204	205
ENSCAFG00845022299	0	0	0	0
ENSCAFG00845022298	0	0	0	0
ENSCAFG00845022297	657	700	648	683
ENSCAFG00845022292	0	0	0	0
ENSCAFG00845022291	0	0	0	0
ENSCAFG00845022290	1	0	4	0
ENSCAFG00845022295	1958	1974	1734	1766
ENSCAFG00845022294	659	707	624	670
ENSCAFG00845022293	15	22	14	15
ENSCAFG00845022289	84	85	89	103
ENSCAFG00845022288	0	0	0	0
ENSCAFG00845022287	0	0	0	0
ENSCAFG00845022286	498	475	481	521
ENSCAFG00845022281	99	84	100	123
ENSCAFG00845022280	2	0	0	0

ENSCAFG00845022285	0	0	0	0
ENSCAFG00845022284	50	38	44	46
ENSCAFG00845022283	1745	1725	1810	1730
ENSCAFG00845022282	0	0	1	1
ENSCAFG00845010292	0	0	0	0
ENSCAFG00845010293	1649	1524	1479	1544
ENSCAFG00845010294	23	20	26	28
ENSCAFG00845010295	0	0	0	0
ENSCAFG00845010290	0	0	0	0
ENSCAFG00845010291	115	105	90	78
ENSCAFG00845022278	1477	1320	1494	1503
ENSCAFG00845022277	1	3	2	1
ENSCAFG00845022276	0	0	0	0
ENSCAFG00845022275	443	402	464	429
ENSCAFG00845022270	3	0	1	8
ENSCAFG00845022274	865	781	1091	1079
ENSCAFG00845022273	148	130	139	125
ENSCAFG00845022272	0	0	0	0
ENSCAFG00845022271	120	135	160	143
ENSCAFG00845010296	6	7	1	1
ENSCAFG00845010297	0	0	0	0
ENSCAFG00845010298	105	93	103	103
ENSCAFG00845010299	0	0	0	0
ENSCAFG00845022429	3	6	12	6
ENSCAFG00845022428	0	0	0	0
ENSCAFG00845022427	0	0	0	0
ENSCAFG00845022426	89	63	92	83
ENSCAFG00845009450	703	741	843	870
ENSCAFG00845009451	0	0	0	0
ENSCAFG00845009454	20	50	73	70
ENSCAFG00845022421	374	309	315	330
ENSCAFG00845009455	0	5	2	7
ENSCAFG00845022420	0	0	0	0
ENSCAFG00845009452	0	0	0	0
ENSCAFG00845009453	2	0	3	2
ENSCAFG00845009458	664	702	771	715
ENSCAFG00845022425	0	0	0	0
ENSCAFG00845009459	2561	2428	2176	2184
ENSCAFG00845022424	44	31	30	41
ENSCAFG00845009456	0	0	0	0
ENSCAFG00845022423	1	0	0	0

ENSCAFG00845009457	64	61	55	79
ENSCAFG00845022422	0	0	0	0
ENSCAFG00845010447	0	0	0	0
ENSCAFG00845010448	3	3	7	10
ENSCAFG00845010449	0	0	0	0
ENSCAFG00845010443	365	416	349	339
ENSCAFG00845010444	155	164	243	210
ENSCAFG00845010445	85	72	70	86
ENSCAFG00845010446	1	1	0	0
ENSCAFG00845010440	70	73	97	70
ENSCAFG00845010441	0	0	0	1
ENSCAFG00845010442	195	206	187	151
ENSCAFG00845022418	0	0	2	0
ENSCAFG00845022417	331	353	271	267
ENSCAFG00845022416	0	0	0	0
ENSCAFG00845022415	3	1	1	4
ENSCAFG00845009440	4	14	11	7
ENSCAFG00845022419	0	0	0	0
ENSCAFG00845009443	511	484	496	530
ENSCAFG00845022410	0	0	0	0
ENSCAFG00845009444	0	0	0	0
ENSCAFG00845009441	96	71	86	99
ENSCAFG00845009442	23	11	14	9
ENSCAFG00845009447	0	0	0	0
ENSCAFG00845022414	0	0	0	0
ENSCAFG00845009448	765	682	707	774
ENSCAFG00845009445	11	3	2	3
ENSCAFG00845022412	0	0	0	0
ENSCAFG00845009446	554	504	430	490
ENSCAFG00845022411	0	5	3	5
ENSCAFG00845009449	0	0	0	0
ENSCAFG00845010436	0	0	0	0
ENSCAFG00845010437	3490	3354	3377	3508
ENSCAFG00845010438	0	0	0	0
ENSCAFG00845010439	1	0	4	1
ENSCAFG00845010432	1893	1824	1645	1756
ENSCAFG00845010433	657	590	727	738
ENSCAFG00845010434	2	0	3	1
ENSCAFG00845010435	0	0	0	0
ENSCAFG00845010430	1	0	0	2
ENSCAFG00845010431	3	6	6	12

ENSCAFG00845022407	162	182	165	169
ENSCAFG00845022406	14	20	14	11
ENSCAFG00845022405	0	0	0	4
ENSCAFG00845022404	0	0	0	0
ENSCAFG00845022409	553	540	428	445
ENSCAFG00845022408	0	0	0	0
ENSCAFG00845009432	0	0	0	0
ENSCAFG00845009433	8	7	1	6
ENSCAFG00845009430	1208	1165	1111	1135
ENSCAFG00845009431	0	0	0	1
ENSCAFG00845009436	2	1	0	4
ENSCAFG00845022403	1101	1008	904	925
ENSCAFG00845009437	720	645	709	716
ENSCAFG00845022402	0	0	0	0
ENSCAFG00845009434	0	0	0	0
ENSCAFG00845022401	0	0	0	0
ENSCAFG00845009435	5538	5395	5337	5470
ENSCAFG00845022400	0	0	0	0
ENSCAFG00845010429	0	0	0	0
ENSCAFG00845009438	0	0	0	0
ENSCAFG00845009439	0	0	0	0
ENSCAFG00845010425	744	803	715	713
ENSCAFG00845010426	578	598	524	579
ENSCAFG00845010427	6	13	9	14
ENSCAFG00845010428	32	37	31	38
ENSCAFG00845010421	14938	14412	15661	16259
ENSCAFG00845010422	1	2	1	2
ENSCAFG00845010423	48	29	36	41
ENSCAFG00845010424	654	601	639	566
ENSCAFG00845010420	0	0	0	0
ENSCAFG00845009421	0	0	0	0
ENSCAFG00845009422	1	1	4	4
ENSCAFG00845009420	0	0	0	0
ENSCAFG00845009425	7	14	3	7
ENSCAFG00845009426	3	4	3	1
ENSCAFG00845009423	63	47	73	67
ENSCAFG00845009424	0	4	2	0
ENSCAFG00845009429	5	3	1	8
ENSCAFG00845010418	0	0	1	0
ENSCAFG00845010419	424	392	387	391
ENSCAFG00845009427	895	817	768	797

ENSCAFG00845009428	0	0	0	0
ENSCAFG00845010414	299	294	308	261
ENSCAFG00845010415	410	446	430	446
ENSCAFG00845010416	26	32	22	30
ENSCAFG00845010417	2894	2828	2648	2649
ENSCAFG00845010410	43	45	30	24
ENSCAFG00845010411	532	590	585	567
ENSCAFG00845010412	27	32	15	16
ENSCAFG00845010413	2237	2250	2197	2277
ENSCAFG00845009410	167	189	185	184
ENSCAFG00845009411	245	231	202	215
ENSCAFG00845009414	3	0	3	3
ENSCAFG00845009415	0	0	0	0
ENSCAFG00845009412	2566	2469	2525	2800
ENSCAFG00845009413	18	9	6	9
ENSCAFG00845009418	1082	1059	1008	983
ENSCAFG00845010407	1859	1773	1843	1924
ENSCAFG00845009419	0	0	0	0
ENSCAFG00845010408	0	0	0	0
ENSCAFG00845009416	0	0	0	0
ENSCAFG00845010409	0	0	1	0
ENSCAFG00845009417	0	0	0	0
ENSCAFG00845010403	0	0	0	0
ENSCAFG00845010404	8	9	12	14
ENSCAFG00845010405	4	2	3	10
ENSCAFG00845010406	34	27	30	17
ENSCAFG00845010400	38	25	38	22
ENSCAFG00845010401	274	227	271	261
ENSCAFG00845010402	0	0	0	0
ENSCAFG00845009400	0	0	0	1
ENSCAFG00845009403	391	366	402	400
ENSCAFG00845009404	1259	1206	1109	1136
ENSCAFG00845009401	0	0	0	0
ENSCAFG00845009402	16	17	24	8
ENSCAFG00845009407	286	249	264	271
ENSCAFG00845009408	32	28	36	30
ENSCAFG00845009405	64	57	51	42
ENSCAFG00845009406	29	30	8	30
ENSCAFG00845009409	4	2	13	19
ENSCAFG00845022388	608	648	643	656
ENSCAFG00845022387	0	0	0	0

ENSCAFG00845022386	133	149	145	141
ENSCAFG00845022385	2	2	0	2
ENSCAFG00845022389	181	149	160	168
ENSCAFG00845022380	0	0	0	1
ENSCAFG00845022384	1468	1490	1506	1468
ENSCAFG00845022383	85	79	109	107
ENSCAFG00845022382	28	20	9	20
ENSCAFG00845022381	0	0	0	0
ENSCAFG00845010391	0	0	0	0
ENSCAFG00845010392	0	0	0	2
ENSCAFG00845010393	3	3	2	3
ENSCAFG00845010394	0	0	0	0
ENSCAFG00845010390	4	0	0	4
ENSCAFG00845022377	0	1	0	0
ENSCAFG00845022376	0	0	0	0
ENSCAFG00845022375	7	4	7	8
ENSCAFG00845022374	28	35	45	40
ENSCAFG00845022379	0	0	0	0
ENSCAFG00845022373	0	0	0	0
ENSCAFG00845022372	4	3	7	4
ENSCAFG00845022371	1135	1116	1181	1179
ENSCAFG00845022370	1	0	0	2
ENSCAFG00845010399	0	0	0	0
ENSCAFG00845010395	2602	2446	2385	2361
ENSCAFG00845010396	614	622	484	526
ENSCAFG00845010397	198	179	225	215
ENSCAFG00845010398	2	0	0	0
ENSCAFG00845009391	82	82	109	79
ENSCAFG00845010380	6515	6287	6570	6365
ENSCAFG00845009392	0	2	2	3
ENSCAFG00845010381	5338	5110	6047	6090
ENSCAFG00845010382	0	0	0	1
ENSCAFG00845009390	51	47	35	48
ENSCAFG00845010383	1	0	1	2
ENSCAFG00845009395	0	0	0	0
ENSCAFG00845009396	8	6	14	12
ENSCAFG00845009393	3	0	2	0
ENSCAFG00845009394	3	1	5	4
ENSCAFG00845009399	1	0	0	2
ENSCAFG00845022366	1119	1143	1261	1331
ENSCAFG00845022365	0	0	0	0

ENSCAFG00845009397	373	383	350	339
ENSCAFG00845022364	0	0	0	0
ENSCAFG00845009398	140	182	132	112
ENSCAFG00845022363	2	5	3	1
ENSCAFG00845022369	0	0	0	0
ENSCAFG00845022368	279	278	209	243
ENSCAFG00845022367	0	0	0	0
ENSCAFG00845022362	199	172	121	143
ENSCAFG00845022361	0	0	0	0
ENSCAFG00845022360	542	502	528	534
ENSCAFG00845010388	89	103	84	109
ENSCAFG00845010389	3190	2950	3191	3101
ENSCAFG00845010384	0	0	0	0
ENSCAFG00845010385	0	0	0	0
ENSCAFG00845010386	2	1	1	2
ENSCAFG00845010387	2	3	5	4
ENSCAFG00845009380	724	695	723	756
ENSCAFG00845009381	0	0	2	0
ENSCAFG00845010370	0	0	0	0
ENSCAFG00845010371	0	0	0	0
ENSCAFG00845010372	368	329	373	392
ENSCAFG00845009384	5	3	1	0
ENSCAFG00845009385	163	161	163	163
ENSCAFG00845009382	4976	5004	5126	4838
ENSCAFG00845009383	211	187	189	198
ENSCAFG00845009388	3	1	2	1
ENSCAFG00845022355	354	322	345	320
ENSCAFG00845009389	0	0	0	0
ENSCAFG00845022354	0	0	0	0
ENSCAFG00845009386	399	387	399	423
ENSCAFG00845022353	0	0	0	0
ENSCAFG00845009387	263	246	285	304
ENSCAFG00845022352	0	4	0	1
ENSCAFG00845022359	573	582	621	649
ENSCAFG00845022358	29	18	23	22
ENSCAFG00845022357	4298	3992	4881	4762
ENSCAFG00845022356	629	683	941	997
ENSCAFG00845022351	0	0	0	0
ENSCAFG00845022350	0	0	0	0
ENSCAFG00845010377	0	0	0	0
ENSCAFG00845010378	0	2	0	0

ENSCAFG00845010379	0	0	0	1
ENSCAFG00845010373	0	0	0	0
ENSCAFG00845010374	3388	3260	3349	3352
ENSCAFG00845010375	485	485	460	482
ENSCAFG00845010376	0	0	1	0
ENSCAFG00845009370	201	232	183	178
ENSCAFG00845010360	0	0	0	0
ENSCAFG00845010361	1625	1557	1595	1738
ENSCAFG00845022349	0	0	0	0
ENSCAFG00845009373	1	1	0	4
ENSCAFG00845009374	520	495	471	469
ENSCAFG00845009371	731	692	697	735
ENSCAFG00845009372	23	21	12	23
ENSCAFG00845009377	1728	1650	1552	1625
ENSCAFG00845022344	0	0	0	0
ENSCAFG00845009378	0	0	0	0
ENSCAFG00845009375	140	149	125	147
ENSCAFG00845022342	516	501	526	486
ENSCAFG00845009376	1	0	0	1
ENSCAFG00845022341	9	5	13	18
ENSCAFG00845022348	19	18	21	18
ENSCAFG00845022347	0	0	0	0
ENSCAFG00845009379	108	125	115	110
ENSCAFG00845022346	5	2	4	10
ENSCAFG00845022345	3	0	0	0
ENSCAFG00845022340	0	0	0	0
ENSCAFG00845010366	63	80	57	53
ENSCAFG00845010367	0	0	0	0
ENSCAFG00845010368	1098	967	996	962
ENSCAFG00845010369	315	302	354	381
ENSCAFG00845010362	8	4	7	7
ENSCAFG00845010363	423	437	465	477
ENSCAFG00845010364	0	0	0	0
ENSCAFG00845010365	5023	4879	5060	5129
ENSCAFG00845022339	0	0	0	0
ENSCAFG00845010350	33	42	24	28
ENSCAFG00845022338	0	0	0	0
ENSCAFG00845009362	0	0	0	0
ENSCAFG00845009363	19	20	14	10
ENSCAFG00845009360	7	3	6	9
ENSCAFG00845009361	1	0	0	2

ENSCAFG00845009366	0	0	0	0
ENSCAFG00845022333	494	472	502	550
ENSCAFG00845024996	509	463	381	374
ENSCAFG00845009367	907	838	876	974
ENSCAFG00845022332	634	576	542	584
ENSCAFG00845024997	1948	1888	1728	1724
ENSCAFG00845009364	0	0	1	0
ENSCAFG00845022331	0	0	0	0
ENSCAFG00845024998	63	63	85	80
ENSCAFG00845009365	6	1	0	3
ENSCAFG00845022330	3	0	0	0
ENSCAFG00845024999	0	0	0	0
ENSCAFG00845022337	1037	1062	954	1014
ENSCAFG00845024992	0	0	0	0
ENSCAFG00845022336	1061	1090	923	883
ENSCAFG00845024993	973	893	916	953
ENSCAFG00845009368	0	2	0	5
ENSCAFG00845022335	1159	1196	1140	1161
ENSCAFG00845024994	566	548	600	671
ENSCAFG00845009369	31	30	21	18
ENSCAFG00845022334	963	909	926	963
ENSCAFG00845024995	0	0	3	0
ENSCAFG00845024990	0	1	4	2
ENSCAFG00845024991	0	0	0	0
ENSCAFG00845010359	248	227	250	247
ENSCAFG00845010355	0	0	0	0
ENSCAFG00845010356	0	0	0	0
ENSCAFG00845010357	0	0	2	0
ENSCAFG00845010358	0	0	0	0
ENSCAFG00845010351	0	0	0	0
ENSCAFG00845010352	9	6	4	9
ENSCAFG00845010353	24	13	24	10
ENSCAFG00845010354	1853	1812	1811	1873
ENSCAFG00845022329	20	18	15	18
ENSCAFG00845022328	0	0	0	0
ENSCAFG00845022327	207	261	193	206
ENSCAFG00845009351	16	14	12	12
ENSCAFG00845024989	283	255	316	316
ENSCAFG00845009352	0	0	0	0
ENSCAFG00845009350	10	5	5	4
ENSCAFG00845009355	1	0	3	2

ENSCAFG00845022322	0	0	0	0
ENSCAFG00845024985	10	10	7	10
ENSCAFG00845009356	0	3	0	2
ENSCAFG00845022321	0	0	0	0
ENSCAFG00845024986	0	0	0	0
ENSCAFG00845009353	19	26	13	22
ENSCAFG00845022320	0	0	0	0
ENSCAFG00845024987	1910	1832	1873	1793
ENSCAFG00845009354	4	13	2	5
ENSCAFG00845024988	290	231	225	239
ENSCAFG00845009359	0	0	0	0
ENSCAFG00845022326	770	617	749	716
ENSCAFG00845024981	2800	2688	2862	3072
ENSCAFG00845022325	275	210	242	219
ENSCAFG00845024982	8	6	4	4
ENSCAFG00845009357	0	0	0	0
ENSCAFG00845022324	719	750	752	663
ENSCAFG00845024983	62	67	50	42
ENSCAFG00845009358	0	0	0	0
ENSCAFG00845022323	1	0	1	6
ENSCAFG00845024984	0	0	0	0
ENSCAFG00845024980	661	605	708	618
ENSCAFG00845010348	0	0	0	0
ENSCAFG00845010349	0	0	0	0
ENSCAFG00845010344	103	102	81	84
ENSCAFG00845010345	185	181	247	209
ENSCAFG00845010346	0	0	0	0
ENSCAFG00845010347	0	0	0	0
ENSCAFG00845010340	287	305	268	282
ENSCAFG00845010341	0	0	0	0
ENSCAFG00845010342	128	92	126	83
ENSCAFG00845010343	2587	2314	2361	2424
ENSCAFG00845022319	478	478	606	525
ENSCAFG00845012990	1	0	0	0
ENSCAFG00845022317	782	682	719	811
ENSCAFG00845022316	3	0	0	0
ENSCAFG00845009340	0	0	0	0
ENSCAFG00845024978	1042	1012	937	921
ENSCAFG00845009341	442	435	468	425
ENSCAFG00845024979	555	441	520	526
ENSCAFG00845009344	0	0	0	0

ENSCAFG00845022311	0	0	0	0
ENSCAFG00845024974	1441	1380	1325	1247
ENSCAFG00845009345	16	5	9	5
ENSCAFG00845022310	213	219	166	181
ENSCAFG00845009342	0	4	0	1
ENSCAFG00845024976	0	0	0	0
ENSCAFG00845009343	0	0	0	0
ENSCAFG00845024977	0	0	0	0
ENSCAFG00845009348	0	0	0	1
ENSCAFG00845022315	0	0	0	0
ENSCAFG00845024970	0	0	0	0
ENSCAFG00845009349	0	0	0	0
ENSCAFG00845022314	3865	3571	3382	3578
ENSCAFG00845024971	0	0	0	0
ENSCAFG00845009346	645	681	617	644
ENSCAFG00845022313	487	444	339	367
ENSCAFG00845024972	0	0	0	0
ENSCAFG00845009347	791	664	698	719
ENSCAFG00845022312	20	42	24	20
ENSCAFG00845024973	792	714	777	833
ENSCAFG00845010337	10	11	2	8
ENSCAFG00845010338	0	0	0	0
ENSCAFG00845012999	663	641	624	637
ENSCAFG00845010339	0	0	0	0
ENSCAFG00845010333	0	0	0	0
ENSCAFG00845012996	0	0	2	0
ENSCAFG00845010334	4467	4389	4311	4032
ENSCAFG00845012995	232	281	245	224
ENSCAFG00845010335	126	112	134	126
ENSCAFG00845012998	0	0	0	0
ENSCAFG00845010336	1677	1665	1513	1643
ENSCAFG00845012997	2	0	0	2
ENSCAFG00845012992	434	410	382	368
ENSCAFG00845010330	1334	1292	1297	1412
ENSCAFG00845012991	99	105	99	88
ENSCAFG00845010331	0	0	0	0
ENSCAFG00845012994	0	0	0	0
ENSCAFG00845010332	0	0	0	0
ENSCAFG00845012993	2747	2891	812	877
ENSCAFG00845022399	0	0	0	0
ENSCAFG00845022398	690	731	698	736

ENSCAFG00845022397	5960	5880	5723	5711
ENSCAFG00845022396	267	267	273	286
ENSCAFG00845022391	2	1	1	9
ENSCAFG00845022390	1	0	0	0
ENSCAFG00845022395	182	176	154	154
ENSCAFG00845022394	0	0	0	0
ENSCAFG00845022393	228	235	166	160
ENSCAFG00845022392	10	1	1	0
ENSCAFG00845022549	0	0	2	0
ENSCAFG00845022548	139	152	128	149
ENSCAFG00845022547	267	212	228	227
ENSCAFG00845009571	0	0	0	0
ENSCAFG00845009572	16	18	22	16
ENSCAFG00845009570	0	0	0	0
ENSCAFG00845009575	1327	1424	1258	1274
ENSCAFG00845022542	46	32	34	31
ENSCAFG00845009576	4259	4214	4225	4258
ENSCAFG00845022541	0	0	0	0
ENSCAFG00845009573	0	0	3	0
ENSCAFG00845022540	0	0	0	0
ENSCAFG00845009574	1363	1257	1396	1365
ENSCAFG00845009579	1	0	0	0
ENSCAFG00845022546	0	2	0	0
ENSCAFG00845022545	0	0	0	0
ENSCAFG00845009577	319	305	410	395
ENSCAFG00845022544	810	820	791	764
ENSCAFG00845009578	317	288	251	287
ENSCAFG00845022543	0	0	2	0
ENSCAFG00845010568	9	6	8	11
ENSCAFG00845010569	70	47	61	56
ENSCAFG00845010564	0	0	0	0
ENSCAFG00845010565	4951	4771	4781	4888
ENSCAFG00845010566	9	7	1	3
ENSCAFG00845010567	0	0	0	0
ENSCAFG00845010560	3	0	0	0
ENSCAFG00845010561	0	0	0	0
ENSCAFG00845010562	2	0	0	0
ENSCAFG00845010563	0	0	0	0
ENSCAFG00845022539	559	622	562	627
ENSCAFG00845022538	0	0	0	1
ENSCAFG00845022537	429	329	395	381

ENSCAFG00845022536	0	0	0	0
ENSCAFG00845009560	0	0	0	0
ENSCAFG00845009561	729	674	630	636
ENSCAFG00845009564	1451	1339	1325	1322
ENSCAFG00845022531	2	6	6	7
ENSCAFG00845009565	2562	2498	2576	2717
ENSCAFG00845022530	666	611	705	713
ENSCAFG00845009562	0	0	0	0
ENSCAFG00845009563	0	0	0	0
ENSCAFG00845009568	6587	6262	6238	6555
ENSCAFG00845022535	5	4	0	4
ENSCAFG00845009569	0	0	0	0
ENSCAFG00845022534	0	0	0	0
ENSCAFG00845009566	0	1	0	1
ENSCAFG00845022533	0	0	0	0
ENSCAFG00845009567	7440	7238	6847	7102
ENSCAFG00845022532	63	60	65	45
ENSCAFG00845010557	0	0	0	0
ENSCAFG00845010558	1	0	0	0
ENSCAFG00845010559	44	37	60	52
ENSCAFG00845010553	1740	1583	1825	1834
ENSCAFG00845010554	0	0	0	0
ENSCAFG00845010555	108	102	135	112
ENSCAFG00845010556	1070	1001	1043	1209
ENSCAFG00845010550	0	0	0	0
ENSCAFG00845010551	0	0	0	0
ENSCAFG00845010552	0	0	0	0
ENSCAFG00845022528	1	1	4	0
ENSCAFG00845022527	0	0	0	0
ENSCAFG00845022526	0	0	0	0
ENSCAFG00845022525	0	1	0	0
ENSCAFG00845009550	0	0	0	0
ENSCAFG00845022529	2	2	2	3
ENSCAFG00845009553	0	0	0	0
ENSCAFG00845022520	1	0	0	0
ENSCAFG00845009554	423	468	422	434
ENSCAFG00845009551	0	0	0	0
ENSCAFG00845009552	37	34	36	43
ENSCAFG00845009557	0	0	0	0
ENSCAFG00845022524	1108	1029	1024	1014
ENSCAFG00845009558	712	814	838	797

ENSCAFG00845022523	366	302	353	370
ENSCAFG00845009555	0	0	0	0
ENSCAFG00845022522	0	0	0	0
ENSCAFG00845009556	6625	6306	5974	6091
ENSCAFG00845022521	95	93	81	69
ENSCAFG00845009559	0	0	0	0
ENSCAFG00845010546	0	0	0	0
ENSCAFG00845010547	6	6	4	9
ENSCAFG00845010548	0	0	0	0
ENSCAFG00845010549	1	4	2	3
ENSCAFG00845010542	0	6	0	2
ENSCAFG00845010543	0	0	0	0
ENSCAFG00845010544	22	24	21	32
ENSCAFG00845010545	3	2	3	1
ENSCAFG00845010540	19	28	19	25
ENSCAFG00845010541	0	0	0	0
ENSCAFG00845022517	15	11	18	11
ENSCAFG00845022516	16	17	23	16
ENSCAFG00845022515	1071	970	889	950
ENSCAFG00845022519	5	0	7	0
ENSCAFG00845022518	1	3	4	3
ENSCAFG00845009542	678	602	707	686
ENSCAFG00845009543	7	5	7	14
ENSCAFG00845009540	0	0	0	0
ENSCAFG00845009541	640	709	659	646
ENSCAFG00845009546	0	0	0	0
ENSCAFG00845022513	9	13	5	5
ENSCAFG00845009547	14	14	9	24
ENSCAFG00845022512	0	0	0	0
ENSCAFG00845009544	0	0	2	0
ENSCAFG00845022511	1	0	1	0
ENSCAFG00845009545	0	0	0	0
ENSCAFG00845022510	740	707	605	628
ENSCAFG00845010539	0	0	0	0
ENSCAFG00845009548	3	2	8	4
ENSCAFG00845009549	0	0	0	0
ENSCAFG00845010535	44	33	26	38
ENSCAFG00845010536	0	0	0	0
ENSCAFG00845010537	0	2	1	0
ENSCAFG00845010538	2869	2795	2376	2412
ENSCAFG00845010531	1	0	0	0

ENSCAFG00845010532	2	6	0	0
ENSCAFG00845010533	883	895	873	852
ENSCAFG00845010534	1	0	0	1
ENSCAFG00845010530	3194	3108	2880	3049
ENSCAFG00845022506	728	650	523	609
ENSCAFG00845022505	0	0	0	0
ENSCAFG00845022504	65	82	72	66
ENSCAFG00845022503	0	1	3	0
ENSCAFG00845022509	0	0	0	0
ENSCAFG00845022508	0	0	0	0
ENSCAFG00845022507	1829	1956	1749	1803
ENSCAFG00845009531	995	948	1054	985
ENSCAFG00845009532	4	0	4	5
ENSCAFG00845009530	1368	1312	1295	1372
ENSCAFG00845009535	6	2	7	4
ENSCAFG00845022502	0	0	0	0
ENSCAFG00845009536	1	0	1	1
ENSCAFG00845022501	404	371	336	330
ENSCAFG00845009533	186	187	155	184
ENSCAFG00845022500	0	0	0	0
ENSCAFG00845009534	2303	2193	2294	2218
ENSCAFG00845009539	28	27	22	21
ENSCAFG00845010528	1	1	0	0
ENSCAFG00845010529	0	0	0	0
ENSCAFG00845009537	0	0	0	0
ENSCAFG00845009538	0	1	0	0
ENSCAFG00845010524	0	0	0	0
ENSCAFG00845010525	138	137	133	124
ENSCAFG00845010526	27	26	18	40
ENSCAFG00845010527	808	804	814	824
ENSCAFG00845010520	0	1	2	5
ENSCAFG00845010521	0	0	0	0
ENSCAFG00845010522	0	0	0	0
ENSCAFG00845010523	2	2	8	5
ENSCAFG00845009520	6	11	6	3
ENSCAFG00845009521	271	271	264	298
ENSCAFG00845009524	0	0	0	0
ENSCAFG00845009525	5	4	4	2
ENSCAFG00845009522	0	0	0	0
ENSCAFG00845009523	0	0	0	0
ENSCAFG00845009528	0	0	0	0

ENSCAFG00845010517	0	0	0	0
ENSCAFG00845009529	1667	1586	1526	1600
ENSCAFG00845010518	471	421	481	436
ENSCAFG00845009526	0	0	0	0
ENSCAFG00845010519	2	1	0	0
ENSCAFG00845009527	0	0	0	0
ENSCAFG00845010513	385	318	350	392
ENSCAFG00845010514	0	0	0	0
ENSCAFG00845010515	0	0	0	0
ENSCAFG00845010516	0	2	0	0
ENSCAFG00845010510	316	304	300	291
ENSCAFG00845010511	0	0	0	0
ENSCAFG00845010512	1	1	3	0
ENSCAFG00845009510	49	56	58	62
ENSCAFG00845009513	0	0	0	0
ENSCAFG00845009514	0	0	0	0
ENSCAFG00845009511	41	34	38	19
ENSCAFG00845009512	471	519	486	479
ENSCAFG00845009517	0	0	0	0
ENSCAFG00845010506	4	1	8	5
ENSCAFG00845009518	13256	12517	13200	13582
ENSCAFG00845010507	621	581	584	589
ENSCAFG00845009515	0	0	0	0
ENSCAFG00845010508	1	0	0	0
ENSCAFG00845009516	0	0	0	0
ENSCAFG00845010509	0	0	0	0
ENSCAFG00845010502	4522	4509	4197	4226
ENSCAFG00845010503	0	0	0	0
ENSCAFG00845009519	5	0	3	2
ENSCAFG00845010504	1	0	0	0
ENSCAFG00845010505	1	2	1	0
ENSCAFG00845010500	0	0	3	3
ENSCAFG00845010501	114	88	114	133
ENSCAFG00845009502	0	0	0	0
ENSCAFG00845009503	397	383	402	401
ENSCAFG00845009500	0	0	0	0
ENSCAFG00845009501	0	0	0	0
ENSCAFG00845009506	690	738	693	692
ENSCAFG00845009507	0	0	0	0
ENSCAFG00845009504	0	0	0	0
ENSCAFG00845009505	370	325	365	424

ENSCAFG00845009508	1147	1183	1050	1095
ENSCAFG00845009509	250	287	191	258
ENSCAFG00845022498	105	96	74	94
ENSCAFG00845022497	1	0	0	0
ENSCAFG00845022495	0	1	0	0
ENSCAFG00845022499	273	305	305	319
ENSCAFG00845022490	0	0	0	0
ENSCAFG00845022494	1	2	0	1
ENSCAFG00845022493	0	0	0	0
ENSCAFG00845022492	178	151	196	180
ENSCAFG00845022491	12	8	7	11
ENSCAFG00845022487	0	0	0	0
ENSCAFG00845022486	762	676	680	652
ENSCAFG00845022485	332	303	305	329
ENSCAFG00845022484	16	24	23	10
ENSCAFG00845022489	31	40	44	34
ENSCAFG00845022488	26	36	21	25
ENSCAFG00845022483	321	288	247	285
ENSCAFG00845022482	0	0	0	0
ENSCAFG00845022481	0	0	0	0
ENSCAFG00845022480	0	0	0	0
ENSCAFG00845010490	0	0	0	0
ENSCAFG00845010491	2	3	1	4
ENSCAFG00845010492	0	0	0	0
ENSCAFG00845010493	6	7	9	23
ENSCAFG00845022476	0	0	0	0
ENSCAFG00845022475	55	68	61	48
ENSCAFG00845022474	363	349	298	325
ENSCAFG00845022473	0	0	0	0
ENSCAFG00845022479	0	0	0	0
ENSCAFG00845022478	0	0	1	0
ENSCAFG00845022477	45	30	41	67
ENSCAFG00845022472	596	584	501	474
ENSCAFG00845022471	2852	2699	2507	2425
ENSCAFG00845022470	0	0	0	0
ENSCAFG00845010498	15	7	11	17
ENSCAFG00845010499	0	0	0	0
ENSCAFG00845010494	0	0	2	0
ENSCAFG00845010495	5	8	8	6
ENSCAFG00845010496	1	0	1	0
ENSCAFG00845010497	2	0	0	0

ENSCAFG00845009490	0	0	0	0
ENSCAFG00845009491	14	11	15	25
ENSCAFG00845010480	135	140	127	146
ENSCAFG00845010481	134	167	122	169
ENSCAFG00845010482	0	0	0	0
ENSCAFG00845009494	0	0	1	0
ENSCAFG00845009495	594	564	659	583
ENSCAFG00845009492	0	0	0	0
ENSCAFG00845009493	0	1	0	0
ENSCAFG00845009498	3089	3061	2805	2946
ENSCAFG00845022465	212	223	225	208
ENSCAFG00845009499	0	0	0	0
ENSCAFG00845022464	11	10	17	8
ENSCAFG00845009496	1	2	1	0
ENSCAFG00845022463	429	438	384	396
ENSCAFG00845009497	16	13	7	14
ENSCAFG00845022462	2	1	2	1
ENSCAFG00845022469	61	41	32	40
ENSCAFG00845022468	1	0	0	0
ENSCAFG00845022467	35	47	54	40
ENSCAFG00845022466	0	0	0	0
ENSCAFG00845022461	208	195	208	239
ENSCAFG00845022460	1	1	0	0
ENSCAFG00845010487	0	0	0	0
ENSCAFG00845010488	0	0	0	0
ENSCAFG00845010489	319	304	238	266
ENSCAFG00845010483	9	7	7	5
ENSCAFG00845010484	1	0	2	0
ENSCAFG00845010485	226	205	250	231
ENSCAFG00845010486	319	346	646	676
ENSCAFG00845009480	8	7	1	2
ENSCAFG00845010470	0	0	0	0
ENSCAFG00845010471	0	3	1	0
ENSCAFG00845022459	0	0	0	0
ENSCAFG00845009483	0	0	0	0
ENSCAFG00845009484	0	1	0	0
ENSCAFG00845009481	162	171	152	113
ENSCAFG00845009482	0	0	0	0
ENSCAFG00845009487	0	0	0	0
ENSCAFG00845022454	0	0	0	0
ENSCAFG00845009488	0	0	0	0

ENSCAFG00845022453	2	4	0	0
ENSCAFG00845009485	0	0	0	0
ENSCAFG00845022452	0	0	0	0
ENSCAFG00845009486	0	0	0	0
ENSCAFG00845022451	1698	1555	1474	1611
ENSCAFG00845022458	0	0	0	0
ENSCAFG00845022457	1377	1339	1224	1188
ENSCAFG00845009489	26	12	22	16
ENSCAFG00845022456	0	0	0	0
ENSCAFG00845022455	966	957	1053	956
ENSCAFG00845022450	4	1	0	3
ENSCAFG00845010476	0	0	0	0
ENSCAFG00845010477	2	0	5	2
ENSCAFG00845010478	0	0	0	0
ENSCAFG00845010479	0	1	0	0
ENSCAFG00845010472	1	0	0	0
ENSCAFG00845010473	0	0	0	0
ENSCAFG00845010474	422	399	385	411
ENSCAFG00845010475	3	6	4	2
ENSCAFG00845022449	2	1	0	1
ENSCAFG00845010460	0	0	0	0
ENSCAFG00845022448	0	2	2	1
ENSCAFG00845009472	577	512	569	538
ENSCAFG00845009473	0	0	0	0
ENSCAFG00845009470	0	0	0	0
ENSCAFG00845009471	37	46	54	43
ENSCAFG00845009476	0	0	0	0
ENSCAFG00845022443	0	0	0	0
ENSCAFG00845009477	1015	916	1054	1057
ENSCAFG00845022442	0	0	0	0
ENSCAFG00845009474	1	0	0	0
ENSCAFG00845022441	0	0	0	0
ENSCAFG00845009475	809	698	700	676
ENSCAFG00845022440	0	0	0	0
ENSCAFG00845022447	347	287	165	207
ENSCAFG00845022446	0	0	0	0
ENSCAFG00845009478	0	0	0	0
ENSCAFG00845022445	8	13	9	3
ENSCAFG00845009479	2	3	0	4
ENSCAFG00845022444	0	0	0	0
ENSCAFG00845010469	511	515	498	491

ENSCAFG00845010465	463	412	407	400
ENSCAFG00845010466	2018	2049	1782	1822
ENSCAFG00845010467	0	0	0	0
ENSCAFG00845010468	0	0	0	0
ENSCAFG00845010461	588	482	556	606
ENSCAFG00845010462	0	0	0	0
ENSCAFG00845010463	0	0	0	0
ENSCAFG00845010464	3	1	2	0
ENSCAFG00845022439	9	8	7	17
ENSCAFG00845022438	2	0	3	0
ENSCAFG00845022437	0	0	0	0
ENSCAFG00845009461	1	1	2	3
ENSCAFG00845009462	12	13	15	11
ENSCAFG00845009460	0	0	0	0
ENSCAFG00845009465	4	4	7	5
ENSCAFG00845022432	19	17	19	21
ENSCAFG00845009466	272	267	238	223
ENSCAFG00845022431	0	0	0	0
ENSCAFG00845009463	871	804	854	823
ENSCAFG00845022430	3	0	0	0
ENSCAFG00845009464	0	1	3	1
ENSCAFG00845009469	440	468	419	437
ENSCAFG00845022436	5882	5707	6216	6352
ENSCAFG00845022435	40	37	28	42
ENSCAFG00845009467	12	8	5	13
ENSCAFG00845022434	0	0	0	0
ENSCAFG00845009468	0	0	1	0
ENSCAFG00845022433	0	0	0	0
ENSCAFG00845010458	14	24	21	26
ENSCAFG00845010459	1139	1019	1059	1125
ENSCAFG00845010454	0	0	0	0
ENSCAFG00845010455	549	517	484	505
ENSCAFG00845010456	1017	976	897	829
ENSCAFG00845010457	0	0	0	0
ENSCAFG00845010450	0	0	0	0
ENSCAFG00845010451	0	0	0	0
ENSCAFG00845010452	366	337	312	302
ENSCAFG00845010453	0	0	0	0
ENSCAFG00845009612	1457	1491	1824	1813
ENSCAFG00845009613	9013	8957	8968	9393
ENSCAFG00845009610	0	0	0	0

ENSCAFG00845009611	0	0	0	0
ENSCAFG00845009616	0	0	0	0
ENSCAFG00845010605	0	0	0	1
ENSCAFG00845009617	372	330	445	421
ENSCAFG00845010606	4	3	1	1
ENSCAFG00845009614	5118	5060	4149	4376
ENSCAFG00845010607	967	912	1047	1062
ENSCAFG00845009615	307	298	295	267
ENSCAFG00845010608	0	0	0	0
ENSCAFG00845010601	769	720	701	691
ENSCAFG00845010602	0	0	0	0
ENSCAFG00845009618	0	0	0	0
ENSCAFG00845010603	0	0	0	0
ENSCAFG00845009619	66	95	57	63
ENSCAFG00845010604	0	0	0	0
ENSCAFG00845010600	0	4	0	1
ENSCAFG00845009601	0	0	0	0
ENSCAFG00845009602	398	352	430	392
ENSCAFG00845009600	542	545	496	437
ENSCAFG00845009605	0	0	0	0
ENSCAFG00845009606	1760	1810	2408	2445
ENSCAFG00845009603	3	1	4	0
ENSCAFG00845009604	0	0	0	0
ENSCAFG00845009609	155	137	124	147
ENSCAFG00845009607	600	596	595	559
ENSCAFG00845009608	0	0	0	0
ENSCAFG00845019019	0	0	0	0
ENSCAFG00845020008	0	0	0	0
ENSCAFG00845020009	0	0	0	0
ENSCAFG00845020006	128	99	97	79
ENSCAFG00845022669	0	0	0	0
ENSCAFG00845010680	508	503	515	517
ENSCAFG00845020007	4	1	0	1
ENSCAFG00845022668	553	587	705	734
ENSCAFG00845009692	0	0	0	0
ENSCAFG00845009693	0	0	0	0
ENSCAFG00845007031	0	0	0	0
ENSCAFG00845009690	158	193	186	179
ENSCAFG00845007030	0	0	5	0
ENSCAFG00845009691	0	0	0	0
ENSCAFG00845007033	78	81	32	15

ENSCAFG00845009696	557	488	502	452
ENSCAFG00845020000	2641	2559	2467	2398
ENSCAFG00845007032	24	17	14	28
ENSCAFG00845009697	980	979	1061	950
ENSCAFG00845020001	3	8	5	5
ENSCAFG00845022662	73	80	69	98
ENSCAFG00845007035	1	0	0	0
ENSCAFG00845009694	299	324	333	312
ENSCAFG00845022661	0	0	0	0
ENSCAFG00845007034	10	3	2	5
ENSCAFG00845009695	22	18	3	10
ENSCAFG00845022660	3	0	0	1
ENSCAFG00845007037	309	306	244	247
ENSCAFG00845020004	8	9	14	5
ENSCAFG00845022667	1171	1089	1126	1131
ENSCAFG00845007036	0	3	0	0
ENSCAFG00845020005	9620	9251	9838	10201
ENSCAFG00845022666	750	698	690	711
ENSCAFG00845007039	0	0	0	0
ENSCAFG00845009698	0	0	0	0
ENSCAFG00845020002	0	0	0	0
ENSCAFG00845022665	3192	3058	2980	3084
ENSCAFG00845007038	40	16	25	30
ENSCAFG00845009699	0	0	6	1
ENSCAFG00845020003	0	0	0	0
ENSCAFG00845022664	0	0	0	0
ENSCAFG00845010689	2035	1930	1861	1928
ENSCAFG00845019010	40	34	29	53
ENSCAFG00845010685	0	0	0	0
ENSCAFG00845019011	506	514	559	631
ENSCAFG00845010686	0	3	0	0
ENSCAFG00845019012	5	2	1	3
ENSCAFG00845010687	0	0	0	0
ENSCAFG00845019013	72	49	50	49
ENSCAFG00845010688	11	12	7	10
ENSCAFG00845019014	0	0	0	0
ENSCAFG00845010681	718	642	579	711
ENSCAFG00845010682	1276	1267	1447	1546
ENSCAFG00845019016	1972	1919	1807	1847
ENSCAFG00845010683	1218	1101	1037	1140
ENSCAFG00845019017	0	0	0	0

ENSCAFG00845010684	1069	1121	989	1023
ENSCAFG00845019018	324	313	357	347
ENSCAFG00845019008	1	0	0	1
ENSCAFG00845019009	0	0	0	0
ENSCAFG00845022659	0	0	0	0
ENSCAFG00845022658	0	0	0	0
ENSCAFG00845022657	0	0	0	0
ENSCAFG00845009681	0	0	0	0
ENSCAFG00845009682	442	459	463	522
ENSCAFG00845007020	167	129	123	145
ENSCAFG00845009680	0	0	0	0
ENSCAFG00845007022	1542	1452	1548	1579
ENSCAFG00845009685	0	0	0	0
ENSCAFG00845022652	23	28	31	38
ENSCAFG00845007021	0	0	0	0
ENSCAFG00845009686	374	383	377	317
ENSCAFG00845022651	0	2	1	0
ENSCAFG00845007024	0	0	0	1
ENSCAFG00845009683	1338	1211	1180	1269
ENSCAFG00845022650	0	0	0	0
ENSCAFG00845007023	881	861	805	780
ENSCAFG00845009684	26	16	24	26
ENSCAFG00845007026	0	0	0	0
ENSCAFG00845009689	6	11	7	6
ENSCAFG00845022656	0	1	0	0
ENSCAFG00845007025	0	0	0	0
ENSCAFG00845022655	0	0	0	0
ENSCAFG00845007028	5	1	0	0
ENSCAFG00845009687	137	149	155	192
ENSCAFG00845022654	234	210	272	240
ENSCAFG00845007027	0	0	0	0
ENSCAFG00845009688	732	699	654	708
ENSCAFG00845022653	40	41	41	27
ENSCAFG00845007029	74	61	57	84
ENSCAFG00845010678	1324	1251	1118	1250
ENSCAFG00845010679	545	524	467	475
ENSCAFG00845010674	0	0	0	0
ENSCAFG00845010675	25	23	29	21
ENSCAFG00845019001	29	13	31	20
ENSCAFG00845010676	1346	1355	1287	1430
ENSCAFG00845019002	1024	1076	886	863

ENSCAFG00845010677	10	6	7	6
ENSCAFG00845019003	388	374	430	424
ENSCAFG00845010670	0	0	0	0
ENSCAFG00845019004	1641	1464	1356	1330
ENSCAFG00845010671	532	514	415	437
ENSCAFG00845019005	0	0	0	0
ENSCAFG00845010672	0	0	0	0
ENSCAFG00845019006	0	0	0	0
ENSCAFG00845010673	0	0	0	0
ENSCAFG00845019007	0	0	0	0
ENSCAFG00845022649	0	0	0	0
ENSCAFG00845022648	0	0	0	0
ENSCAFG00845022647	38	39	33	49
ENSCAFG00845022646	1077	1189	1161	1148
ENSCAFG00845009670	0	0	0	0
ENSCAFG00845009671	1	0	0	0
ENSCAFG00845007011	0	0	0	0
ENSCAFG00845009674	26	31	20	27
ENSCAFG00845022641	0	0	0	0
ENSCAFG00845007010	1184	1090	1242	1280
ENSCAFG00845009675	9	13	5	9
ENSCAFG00845022640	0	0	0	0
ENSCAFG00845007013	0	0	0	0
ENSCAFG00845009672	11	36	33	20
ENSCAFG00845007012	0	4	1	1
ENSCAFG00845009673	493	575	493	506
ENSCAFG00845007015	0	0	0	0
ENSCAFG00845009678	0	0	0	0
ENSCAFG00845022645	0	0	0	0
ENSCAFG00845007014	2981	2952	2786	2818
ENSCAFG00845009679	0	0	0	0
ENSCAFG00845022644	0	0	0	0
ENSCAFG00845007017	0	0	0	0
ENSCAFG00845009676	0	0	0	0
ENSCAFG00845022643	0	0	0	0
ENSCAFG00845007016	2822	2791	2789	2868
ENSCAFG00845009677	0	0	0	0
ENSCAFG00845022642	1176	1291	1197	1252
ENSCAFG00845007019	0	0	0	0
ENSCAFG00845007018	0	0	0	0
ENSCAFG00845010667	0	0	0	0

ENSCAFG00845010668	0	0	0	0
ENSCAFG00845010669	1	1	0	1
ENSCAFG00845010663	26	19	12	8
ENSCAFG00845010664	0	0	0	0
ENSCAFG00845010665	0	1	0	0
ENSCAFG00845010666	10	9	5	9
ENSCAFG00845010660	0	0	0	0
ENSCAFG00845010661	3	1	0	2
ENSCAFG00845010662	0	0	0	0
ENSCAFG00845022638	184	156	142	182
ENSCAFG00845022637	0	0	0	0
ENSCAFG00845022636	0	0	0	0
ENSCAFG00845022635	0	0	0	0
ENSCAFG00845009660	1317	1184	1158	1244
ENSCAFG00845022639	0	0	0	0
ENSCAFG00845007000	232	217	199	200
ENSCAFG00845009663	0	0	0	0
ENSCAFG00845022630	6	6	8	9
ENSCAFG00845009664	0	0	0	0
ENSCAFG00845007002	0	0	0	0
ENSCAFG00845009661	0	0	0	0
ENSCAFG00845007001	0	0	0	0
ENSCAFG00845009662	3178	3226	3009	2946
ENSCAFG00845007004	0	0	0	0
ENSCAFG00845009667	0	0	0	0
ENSCAFG00845022634	555	517	535	493
ENSCAFG00845007003	38071	37403	38185	38430
ENSCAFG00845009668	0	0	1	0
ENSCAFG00845022633	291	252	223	245
ENSCAFG00845007006	0	0	0	0
ENSCAFG00845009665	0	0	0	0
ENSCAFG00845022632	2763	2829	3014	3082
ENSCAFG00845007005	25	18	19	24
ENSCAFG00845009666	158	168	187	204
ENSCAFG00845022631	2	0	1	5
ENSCAFG00845007008	0	0	0	0
ENSCAFG00845007007	3239	3176	2497	2578
ENSCAFG00845009669	516	520	501	534
ENSCAFG00845007009	0	0	0	0
ENSCAFG00845010656	28	32	19	25
ENSCAFG00845010657	0	0	0	0

ENSCAFG00845010658	709	717	716	741
ENSCAFG00845010659	50	62	44	30
ENSCAFG00845010652	1	10	15	11
ENSCAFG00845010653	258	238	279	285
ENSCAFG00845010654	1	1	0	1
ENSCAFG00845010655	0	0	0	0
ENSCAFG00845010650	241	231	289	264
ENSCAFG00845010651	1	0	1	0
ENSCAFG00845022627	0	1	0	1
ENSCAFG00845022626	0	1	0	0
ENSCAFG00845022625	587	525	503	476
ENSCAFG00845022624	266	248	261	261
ENSCAFG00845022628	276	258	278	283
ENSCAFG00845009652	2012	1967	1754	1879
ENSCAFG00845009653	1832	1678	2002	1947
ENSCAFG00845009650	0	1	0	2
ENSCAFG00845009651	0	0	0	0
ENSCAFG00845009656	0	0	0	0
ENSCAFG00845022623	0	0	0	0
ENSCAFG00845009657	503	514	496	511
ENSCAFG00845022622	7660	7183	6682	6649
ENSCAFG00845009654	0	0	0	0
ENSCAFG00845022621	307	289	293	300
ENSCAFG00845009655	122	129	117	155
ENSCAFG00845022620	27	35	34	24
ENSCAFG00845010649	0	0	0	0
ENSCAFG00845009658	68	56	37	36
ENSCAFG00845009659	472	521	440	401
ENSCAFG00845010645	0	0	0	0
ENSCAFG00845010646	0	0	0	0
ENSCAFG00845010647	10	5	4	5
ENSCAFG00845010648	1	0	2	0
ENSCAFG00845010641	0	0	0	0
ENSCAFG00845010642	0	0	0	0
ENSCAFG00845010643	0	0	0	0
ENSCAFG00845010644	0	0	0	0
ENSCAFG00845010640	2	2	0	0
ENSCAFG00845022616	0	0	0	0
ENSCAFG00845022615	0	0	0	0
ENSCAFG00845022614	397	440	420	464
ENSCAFG00845022613	0	0	0	0

ENSCAFG00845022619	13	5	12	4
ENSCAFG00845022618	0	0	0	0
ENSCAFG00845022617	4156	4057	3776	3932
ENSCAFG00845009641	0	0	0	0
ENSCAFG00845009642	169	143	184	175
ENSCAFG00845009640	0	0	0	0
ENSCAFG00845009645	0	0	0	0
ENSCAFG00845022612	1	2	0	0
ENSCAFG00845009646	746	719	830	798
ENSCAFG00845022611	0	0	0	0
ENSCAFG00845009643	0	0	0	0
ENSCAFG00845022610	0	0	0	0
ENSCAFG00845009644	14	23	13	10
ENSCAFG00845009649	0	0	0	0
ENSCAFG00845010638	0	0	0	0
ENSCAFG00845010639	882	918	938	875
ENSCAFG00845009647	85	92	111	89
ENSCAFG00845009648	0	0	0	0
ENSCAFG00845010634	10	6	4	7
ENSCAFG00845010635	185	210	183	201
ENSCAFG00845010636	87	59	82	69
ENSCAFG00845010637	88771	87447	96335	95552
ENSCAFG00845010630	0	0	0	0
ENSCAFG00845010631	0	0	0	0
ENSCAFG00845010632	99	73	101	95
ENSCAFG00845010633	2	1	5	1
ENSCAFG00845022605	2146	2099	2129	2049
ENSCAFG00845022604	994	949	692	608
ENSCAFG00845022603	0	0	0	0
ENSCAFG00845022602	0	0	0	0
ENSCAFG00845022609	5390	5109	4633	4875
ENSCAFG00845022608	0	0	0	0
ENSCAFG00845022607	1066	973	1012	987
ENSCAFG00845022606	6	0	0	1
ENSCAFG00845009630	305	271	270	266
ENSCAFG00845009631	3	0	0	3
ENSCAFG00845009634	66	87	62	68
ENSCAFG00845022601	973	902	836	949
ENSCAFG00845009635	0	0	0	0
ENSCAFG00845022600	0	0	0	0
ENSCAFG00845009632	555	510	533	453

ENSCAFG00845009633	1068	1075	1103	1125
ENSCAFG00845009638	1	0	7	1
ENSCAFG00845010627	0	0	0	0
ENSCAFG00845009639	0	0	0	0
ENSCAFG00845010628	518	426	496	477
ENSCAFG00845009636	623	668	692	665
ENSCAFG00845010629	6400	6217	6536	6814
ENSCAFG00845009637	163	167	153	147
ENSCAFG00845010623	1356	1427	1377	1421
ENSCAFG00845010624	0	0	0	0
ENSCAFG00845010625	11	12	5	11
ENSCAFG00845010626	0	0	0	0
ENSCAFG00845010620	5	3	5	5
ENSCAFG00845010621	0	1	2	1
ENSCAFG00845010622	168	198	159	159
ENSCAFG00845009620	428	459	416	431
ENSCAFG00845009623	675	608	587	609
ENSCAFG00845010609	241	263	170	137
ENSCAFG00845009624	276	336	299	323
ENSCAFG00845009621	84	84	103	97
ENSCAFG00845009622	0	0	0	1
ENSCAFG00845009627	0	0	0	0
ENSCAFG00845010616	0	0	0	0
ENSCAFG00845009628	284	226	213	175
ENSCAFG00845010617	759	676	691	658
ENSCAFG00845009625	0	0	0	0
ENSCAFG00845010618	8	9	12	20
ENSCAFG00845009626	0	0	0	0
ENSCAFG00845010619	1	0	0	0
ENSCAFG00845010612	2572	2676	2482	2522
ENSCAFG00845010613	6	3	1	2
ENSCAFG00845009629	5	5	3	4
ENSCAFG00845010614	0	0	0	0
ENSCAFG00845010615	0	0	0	0
ENSCAFG00845010610	0	0	0	0
ENSCAFG00845010611	327	302	281	262
ENSCAFG00845022597	19	16	17	13
ENSCAFG00845022596	885	834	803	872
ENSCAFG00845022595	15	8	11	9
ENSCAFG00845022594	1	0	0	2
ENSCAFG00845022599	1539	1468	1562	1466

ENSCAFG00845022598	0	0	2	0
ENSCAFG00845022593	7649	7448	8231	8480
ENSCAFG00845022592	0	0	0	0
ENSCAFG00845022591	0	0	0	0
ENSCAFG00845022590	314	291	297	309
ENSCAFG00845022586	0	0	0	0
ENSCAFG00845022585	21	36	38	33
ENSCAFG00845022584	290	290	307	286
ENSCAFG00845022583	2	2	6	1
ENSCAFG00845022589	0	0	0	0
ENSCAFG00845022588	0	0	0	0
ENSCAFG00845022587	0	0	0	0
ENSCAFG00845022582	0	0	0	0
ENSCAFG00845022581	29	26	25	39
ENSCAFG00845022580	284	340	275	314
ENSCAFG00845010590	74	71	66	75
ENSCAFG00845010591	255	238	259	252
ENSCAFG00845010592	0	1	0	1
ENSCAFG00845022575	22	43	34	34
ENSCAFG00845022574	0	0	0	0
ENSCAFG00845022573	0	0	0	2
ENSCAFG00845022572	2044	2056	1966	1928
ENSCAFG00845022579	2	2	3	0
ENSCAFG00845022578	109	120	95	124
ENSCAFG00845022577	385	371	361	417
ENSCAFG00845022576	0	0	0	0
ENSCAFG00845022571	0	0	0	2
ENSCAFG00845022570	0	0	0	0
ENSCAFG00845010597	1	1	4	1
ENSCAFG00845010598	479	528	455	489
ENSCAFG00845010599	0	0	0	5
ENSCAFG00845010593	0	0	0	0
ENSCAFG00845010594	581	573	473	405
ENSCAFG00845010595	0	0	0	0
ENSCAFG00845010596	1943	1858	1640	1637
ENSCAFG00845009590	0	0	0	0
ENSCAFG00845010580	20	24	15	15
ENSCAFG00845010581	0	0	0	0
ENSCAFG00845022569	0	0	0	0
ENSCAFG00845009593	1624	1492	1507	1535
ENSCAFG00845009594	0	0	0	0

ENSCAFG00845009591	4	1	1	2
ENSCAFG00845009592	0	0	0	0
ENSCAFG00845009597	1	2	3	0
ENSCAFG00845022564	7	6	7	7
ENSCAFG00845009598	0	0	0	0
ENSCAFG00845022563	3	2	2	1
ENSCAFG00845009595	0	0	1	0
ENSCAFG00845022562	0	0	0	0
ENSCAFG00845009596	0	0	0	0
ENSCAFG00845022561	0	0	1	1
ENSCAFG00845022568	0	0	2	0
ENSCAFG00845022567	235	215	250	256
ENSCAFG00845009599	1	0	1	5
ENSCAFG00845022566	14	19	40	35
ENSCAFG00845022565	1	2	1	1
ENSCAFG00845022560	0	3	1	6
ENSCAFG00845010586	101	103	125	134
ENSCAFG00845010587	1207	1148	1099	1025
ENSCAFG00845010588	0	0	0	3
ENSCAFG00845010589	503	554	447	448
ENSCAFG00845010582	1310	1288	645	673
ENSCAFG00845010583	0	0	0	0
ENSCAFG00845010584	5	1	7	2
ENSCAFG00845010585	39	43	21	31
ENSCAFG00845022559	465	482	433	574
ENSCAFG00845010570	380	375	425	365
ENSCAFG00845022558	787	841	719	746
ENSCAFG00845009582	0	0	0	0
ENSCAFG00845009583	711	639	658	694
ENSCAFG00845009580	0	0	0	0
ENSCAFG00845009581	5	1	1	1
ENSCAFG00845009586	1145	1159	1061	1099
ENSCAFG00845022553	15	22	12	9
ENSCAFG00845009587	9	14	6	2
ENSCAFG00845022552	3	13	7	8
ENSCAFG00845009584	225	179	118	105
ENSCAFG00845022551	86	93	116	108
ENSCAFG00845009585	87	69	68	75
ENSCAFG00845022550	124	102	96	108
ENSCAFG00845022557	3	2	1	0
ENSCAFG00845022556	0	0	0	0

ENSCAFG00845009588	0	0	0	0
ENSCAFG00845022555	0	3	0	0
ENSCAFG00845009589	12	16	15	19
ENSCAFG00845022554	14111	13410	13669	13435
ENSCAFG00845010579	674	661	596	594
ENSCAFG00845010575	618	612	677	705
ENSCAFG00845010576	145	159	151	147
ENSCAFG00845010577	0	0	0	0
ENSCAFG00845010578	1	0	0	0
ENSCAFG00845010571	1114	1115	1039	1045
ENSCAFG00845010572	0	0	0	0
ENSCAFG00845010573	1716	1544	1730	1667
ENSCAFG00845010574	0	0	1	5
ENSCAFG00845000518	696	649	684	743
ENSCAFG00845000519	0	0	0	1
ENSCAFG00845000516	331	336	399	387
ENSCAFG00845000517	1717	1508	1536	1559
ENSCAFG00845000514	1120	1038	1051	991
ENSCAFG00845000515	59	56	48	46
ENSCAFG00845000512	78	53	55	62
ENSCAFG00845000513	202	199	189	184
ENSCAFG00845000510	1521	1551	1406	1359
ENSCAFG00845000511	179	130	157	131
ENSCAFG00845012509	336	397	426	448
ENSCAFG00845012508	0	0	0	0
ENSCAFG00845012505	2	3	0	1
ENSCAFG00845012504	2E+05	2E+05	162380	162877
ENSCAFG00845012507	1	0	0	0
ENSCAFG00845012506	2340	2301	2023	2111
ENSCAFG00845012501	0	1	1	0
ENSCAFG00845012500	271	292	309	309
ENSCAFG00845012503	0	0	0	0
ENSCAFG00845012502	0	0	0	0
ENSCAFG00845000529	0	0	1	0
ENSCAFG00845000527	0	0	0	0
ENSCAFG00845000528	1	0	0	0
ENSCAFG00845000525	0	0	0	0
ENSCAFG00845000526	20	14	21	24
ENSCAFG00845000523	688	697	597	619
ENSCAFG00845000524	0	0	0	0
ENSCAFG00845000521	851	885	1001	1009

ENSCAFG00845000522	0	0	0	0
ENSCAFG00845000520	0	0	0	0
ENSCAFG00845000509	0	2	1	0
ENSCAFG00845000507	305	315	293	303
ENSCAFG00845000508	779	694	776	772
ENSCAFG00845000505	873	808	871	894
ENSCAFG00845000506	0	0	0	0
ENSCAFG00845000503	583	607	543	485
ENSCAFG00845000504	585	538	485	477
ENSCAFG00845000501	0	1	0	0
ENSCAFG00845000502	200	179	225	205
ENSCAFG00845000500	296	349	318	372
ENSCAFG00845012460	149	141	147	129
ENSCAFG00845012462	6	1	5	8
ENSCAFG00845012461	0	0	0	0
ENSCAFG00845000479	755	692	738	800
ENSCAFG00845024446	0	1	0	1
ENSCAFG00845024447	0	0	0	0
ENSCAFG00845000477	0	0	0	0
ENSCAFG00845024448	2	2	0	1
ENSCAFG00845000478	119	131	132	125
ENSCAFG00845024449	1173	1128	1008	1010
ENSCAFG00845000475	148	126	117	115
ENSCAFG00845024442	0	2	4	0
ENSCAFG00845000476	0	0	0	0
ENSCAFG00845024443	1546	1455	1545	1563
ENSCAFG00845000473	5	1	6	3
ENSCAFG00845000474	0	0	0	0
ENSCAFG00845024445	0	1	0	2
ENSCAFG00845000471	22	16	41	49
ENSCAFG00845000472	0	0	0	0
ENSCAFG00845024440	186	186	173	199
ENSCAFG00845000470	0	0	1	1
ENSCAFG00845024441	528	544	538	583
ENSCAFG00845012468	14	14	15	19
ENSCAFG00845012467	0	0	0	0
ENSCAFG00845012469	0	1	1	1
ENSCAFG00845012464	0	2	0	0
ENSCAFG00845012463	8	7	6	11
ENSCAFG00845012466	10	4	21	9
ENSCAFG00845012465	0	3	0	0

ENSCAFG00845012451	1	1	0	0
ENSCAFG00845012450	14	9	21	18
ENSCAFG00845024439	3	3	10	5
ENSCAFG00845024435	0	0	0	0
ENSCAFG00845024436	0	0	0	0
ENSCAFG00845000488	899	798	862	845
ENSCAFG00845024437	0	0	0	0
ENSCAFG00845000489	594	546	528	487
ENSCAFG00845024438	179	215	167	184
ENSCAFG00845000486	3	10	11	8
ENSCAFG00845024431	1	5	0	1
ENSCAFG00845000487	0	0	0	0
ENSCAFG00845024432	36	36	28	19
ENSCAFG00845000484	66	58	51	61
ENSCAFG00845024433	3	4	5	4
ENSCAFG00845000485	0	0	0	3
ENSCAFG00845024434	4	14	16	16
ENSCAFG00845000482	0	2	0	1
ENSCAFG00845000483	0	0	0	0
ENSCAFG00845000480	4095	3775	3634	3648
ENSCAFG00845000481	3769	3903	4471	4358
ENSCAFG00845024430	0	0	0	0
ENSCAFG00845012457	0	0	0	0
ENSCAFG00845012456	1927	1978	1770	1913
ENSCAFG00845012459	0	0	0	0
ENSCAFG00845012458	0	0	0	0
ENSCAFG00845012453	0	0	0	0
ENSCAFG00845012452	645	628	541	531
ENSCAFG00845012455	3243	3106	2974	2996
ENSCAFG00845012454	1451	1446	1452	1488
ENSCAFG00845012440	0	0	0	0
ENSCAFG00845024429	271	273	228	255
ENSCAFG00845000459	793	764	656	770
ENSCAFG00845000457	1591	1589	1530	1570
ENSCAFG00845024424	0	0	1	0
ENSCAFG00845000458	547	531	528	525
ENSCAFG00845024425	6	1	3	0
ENSCAFG00845000455	0	0	0	0
ENSCAFG00845024426	408	382	382	377
ENSCAFG00845000456	537	547	532	527
ENSCAFG00845024427	0	0	0	0

ENSCAFG00845000453	0	0	0	1
ENSCAFG00845024420	26	23	17	14
ENSCAFG00845000454	186	184	154	188
ENSCAFG00845024421	0	0	0	0
ENSCAFG00845000451	0	0	0	0
ENSCAFG00845024422	638	648	663	717
ENSCAFG00845000452	0	0	0	0
ENSCAFG00845024423	0	0	1	0
ENSCAFG00845000450	61	79	67	89
ENSCAFG00845012449	0	0	0	0
ENSCAFG00845012446	0	3	0	3
ENSCAFG00845012445	1309	1183	1398	1328
ENSCAFG00845012448	0	0	0	0
ENSCAFG00845012447	465	489	404	479
ENSCAFG00845012442	166	146	173	175
ENSCAFG00845012441	0	0	0	0
ENSCAFG00845012444	0	0	0	0
ENSCAFG00845012443	0	0	0	0
ENSCAFG00845024417	9	10	7	5
ENSCAFG00845024418	0	0	0	1
ENSCAFG00845024419	525	446	410	390
ENSCAFG00845000468	0	0	0	0
ENSCAFG00845024413	0	0	0	0
ENSCAFG00845000469	64	86	78	62
ENSCAFG00845024414	1648	1634	1633	1706
ENSCAFG00845000466	16	20	16	21
ENSCAFG00845024415	2002	1869	1995	1969
ENSCAFG00845000467	0	0	0	0
ENSCAFG00845024416	0	1	1	0
ENSCAFG00845000464	201	253	223	223
ENSCAFG00845000465	590	512	571	605
ENSCAFG00845024410	0	0	0	0
ENSCAFG00845000462	0	0	0	0
ENSCAFG00845024411	0	0	0	0
ENSCAFG00845000463	419	369	388	381
ENSCAFG00845024412	71	56	67	55
ENSCAFG00845000460	0	0	1	0
ENSCAFG00845000461	1193	1124	1376	1289
ENSCAFG00845012439	0	1	1	0
ENSCAFG00845012438	637	605	663	623
ENSCAFG00845012435	0	0	0	0

ENSCAFG00845012434	2489	2422	2323	2306
ENSCAFG00845012437	1993	1962	1845	1765
ENSCAFG00845012436	21	33	41	22
ENSCAFG00845012431	3	2	1	1
ENSCAFG00845012430	0	0	0	0
ENSCAFG00845012433	110	141	125	115
ENSCAFG00845012432	3000	3079	2912	3127
ENSCAFG00845000439	1227	1201	1241	1261
ENSCAFG00845024406	1995	1867	2088	2069
ENSCAFG00845024407	3168	3039	2919	2932
ENSCAFG00845000437	1228	1195	1140	1195
ENSCAFG00845000438	0	0	0	0
ENSCAFG00845024409	2	2	3	9
ENSCAFG00845000435	0	6	10	6
ENSCAFG00845024402	0	0	0	0
ENSCAFG00845000436	2	0	1	2
ENSCAFG00845024403	618	648	586	609
ENSCAFG00845000433	0	0	0	0
ENSCAFG00845024404	0	0	0	0
ENSCAFG00845000434	54	33	37	46
ENSCAFG00845024405	0	0	0	0
ENSCAFG00845000431	0	0	1	0
ENSCAFG00845000432	348	351	564	519
ENSCAFG00845024400	0	0	0	0
ENSCAFG00845000430	852	754	844	777
ENSCAFG00845024401	1137	1027	1056	1120
ENSCAFG00845012428	26	40	22	33
ENSCAFG00845012427	601	528	551	590
ENSCAFG00845012429	6	3	10	1
ENSCAFG00845012424	7	2	8	9
ENSCAFG00845012423	617	597	633	634
ENSCAFG00845012426	603	597	540	582
ENSCAFG00845012425	1176	1060	1153	1217
ENSCAFG00845012420	0	0	0	0
ENSCAFG00845012422	0	0	0	0
ENSCAFG00845012421	248	287	230	290
ENSCAFG00845000448	491	476	440	467
ENSCAFG00845000449	7	4	3	0
ENSCAFG00845000446	0	0	0	0
ENSCAFG00845000447	0	0	0	0
ENSCAFG00845000444	0	0	0	2

ENSCAFG00845000445	4573	4408	4454	4610
ENSCAFG00845000442	0	0	0	0
ENSCAFG00845000443	2369	2241	2519	2518
ENSCAFG00845000440	161	144	141	135
ENSCAFG00845000441	0	0	0	0
ENSCAFG00845012417	5	4	16	8
ENSCAFG00845012416	1102	1096	905	925
ENSCAFG00845012419	0	0	0	0
ENSCAFG00845012418	0	0	0	0
ENSCAFG00845012413	9	13	14	7
ENSCAFG00845012412	77	86	43	58
ENSCAFG00845012415	3436	3496	3145	3137
ENSCAFG00845012414	1838	1738	1699	1867
ENSCAFG00845012411	0	0	0	0
ENSCAFG00845012410	142	113	144	135
ENSCAFG00845000419	51	49	48	46
ENSCAFG00845000417	215	207	218	216
ENSCAFG00845000418	1	0	0	0
ENSCAFG00845000415	97	101	97	86
ENSCAFG00845000416	2	3	1	1
ENSCAFG00845000413	1829	1833	1893	1966
ENSCAFG00845000414	0	2	1	1
ENSCAFG00845000411	429	391	480	481
ENSCAFG00845000412	93	78	49	84
ENSCAFG00845000410	30	27	10	30
ENSCAFG00845012409	0	0	0	0
ENSCAFG00845012406	0	0	0	0
ENSCAFG00845012405	0	1	0	1
ENSCAFG00845012408	70	78	56	60
ENSCAFG00845012407	0	0	0	0
ENSCAFG00845012402	0	0	0	0
ENSCAFG00845012401	0	0	0	0
ENSCAFG00845012404	1878	1864	1979	2032
ENSCAFG00845012403	7	4	5	7
ENSCAFG00845012400	4006	3863	3665	3804
ENSCAFG00845000428	1	0	0	0
ENSCAFG00845000429	1092	1097	954	981
ENSCAFG00845000426	622	658	657	736
ENSCAFG00845000427	6	1	3	3
ENSCAFG00845000424	251	238	239	197
ENSCAFG00845000425	6	0	7	10

ENSCAFG00845000422	245	248	233	254
ENSCAFG00845000423	4784	4526	4853	4883
ENSCAFG00845000420	30	26	29	27
ENSCAFG00845000421	0	0	0	0
ENSCAFG00845024497	290	291	206	200
ENSCAFG00845024498	0	0	0	0
ENSCAFG00845024499	0	0	0	2
ENSCAFG00845024493	503	480	512	485
ENSCAFG00845024494	882	785	899	799
ENSCAFG00845024495	4114	3977	3839	3854
ENSCAFG00845024496	1	0	0	0
ENSCAFG00845024490	0	0	0	0
ENSCAFG00845024491	1394	1168	1320	1327
ENSCAFG00845024486	0	0	1	2
ENSCAFG00845024488	247	254	207	244
ENSCAFG00845024489	3077	3156	3396	3438
ENSCAFG00845024482	117	87	140	135
ENSCAFG00845024483	0	0	1	0
ENSCAFG00845024484	182	151	167	181
ENSCAFG00845024485	346	341	375	357
ENSCAFG00845024480	1	2	1	2
ENSCAFG00845024481	91	113	89	118
ENSCAFG00845012493	1	0	4	6
ENSCAFG00845012492	903	944	992	929
ENSCAFG00845012495	1074	1083	1079	1126
ENSCAFG00845012494	0	0	0	0
ENSCAFG00845012491	0	0	0	0
ENSCAFG00845012490	0	0	0	0
ENSCAFG00845024479	0	0	0	0
ENSCAFG00845024475	18	6	19	27
ENSCAFG00845024476	1735	1494	1653	1628
ENSCAFG00845024477	0	0	0	0
ENSCAFG00845024478	0	0	0	0
ENSCAFG00845024471	0	0	0	0
ENSCAFG00845024472	25	26	24	25
ENSCAFG00845024473	0	0	1	0
ENSCAFG00845024474	281	257	258	302
ENSCAFG00845024470	2	0	0	0
ENSCAFG00845012497	222	203	231	189
ENSCAFG00845012496	615	621	729	796
ENSCAFG00845012499	1698	1681	1037	1132

ENSCAFG00845012498	0	0	0	0
ENSCAFG00845012482	0	0	0	0
ENSCAFG00845012481	2	0	0	0
ENSCAFG00845012484	0	0	0	0
ENSCAFG00845012483	840	886	822	770
ENSCAFG00845012480	0	0	0	0
ENSCAFG00845024468	0	0	0	0
ENSCAFG00845024469	0	0	0	0
ENSCAFG00845000499	31	43	49	42
ENSCAFG00845000497	1225	1326	1251	1174
ENSCAFG00845024464	0	0	0	0
ENSCAFG00845000498	567	490	549	578
ENSCAFG00845024465	0	0	0	0
ENSCAFG00845000495	1868	1807	1803	1773
ENSCAFG00845024466	0	4	2	1
ENSCAFG00845000496	0	0	0	0
ENSCAFG00845024467	433	425	440	408
ENSCAFG00845000493	0	1	1	0
ENSCAFG00845024460	1	4	1	0
ENSCAFG00845000494	0	0	0	0
ENSCAFG00845024461	0	0	0	0
ENSCAFG00845000491	273	262	296	270
ENSCAFG00845000492	2271	2037	2164	2159
ENSCAFG00845024463	0	0	0	0
ENSCAFG00845000490	7	16	6	10
ENSCAFG00845012489	7	9	5	3
ENSCAFG00845012486	236	270	246	224
ENSCAFG00845012485	355	356	444	404
ENSCAFG00845012488	0	0	0	0
ENSCAFG00845012487	1100	1084	931	997
ENSCAFG00845012471	652	683	577	565
ENSCAFG00845012470	1	0	0	0
ENSCAFG00845012473	0	0	1	1
ENSCAFG00845012472	0	0	0	0
ENSCAFG00845024457	1394	1418	1511	1403
ENSCAFG00845024458	0	2	0	0
ENSCAFG00845024459	70	55	46	57
ENSCAFG00845024453	0	0	2	0
ENSCAFG00845024455	0	0	3	3
ENSCAFG00845024456	1	0	0	0
ENSCAFG00845024451	341	323	276	301

ENSCAFG00845024452	29	18	19	19
ENSCAFG00845012479	0	0	0	0
ENSCAFG00845012478	0	0	0	0
ENSCAFG00845012475	1079	1103	1172	1170
ENSCAFG00845012474	61	51	69	77
ENSCAFG00845012477	508	567	534	550
ENSCAFG00845012476	0	0	0	0
ENSCAFG00845024608	0	0	0	0
ENSCAFG00845024609	1084	1042	897	1001
ENSCAFG00845000639	38	52	72	60
ENSCAFG00845000637	0	0	0	0
ENSCAFG00845024604	0	0	0	0
ENSCAFG00845000638	517	472	437	459
ENSCAFG00845024605	10	3	6	11
ENSCAFG00845000635	655	679	539	560
ENSCAFG00845024606	356	280	299	317
ENSCAFG00845000636	1168	1196	1071	1117
ENSCAFG00845024607	135	108	113	99
ENSCAFG00845000633	0	1	0	0
ENSCAFG00845024600	0	0	0	0
ENSCAFG00845000634	114	108	110	118
ENSCAFG00845024601	4	2	0	0
ENSCAFG00845000631	747	764	717	680
ENSCAFG00845024602	43	66	71	63
ENSCAFG00845000632	0	0	0	1
ENSCAFG00845024603	0	0	2	0
ENSCAFG00845000630	0	0	0	0
ENSCAFG00845012629	184	212	109	133
ENSCAFG00845012626	4	1	4	2
ENSCAFG00845012625	0	0	0	0
ENSCAFG00845012628	0	0	0	0
ENSCAFG00845012627	0	0	0	0
ENSCAFG00845012622	0	0	0	0
ENSCAFG00845012621	844	820	855	916
ENSCAFG00845012624	395	336	465	376
ENSCAFG00845012623	0	0	0	0
ENSCAFG00845012620	2	4	0	1
ENSCAFG00845000648	17	9	18	13
ENSCAFG00845000649	4	0	0	0
ENSCAFG00845000646	0	0	0	0
ENSCAFG00845000647	1	4	3	0

ENSCAFG00845000644	0	0	0	0
ENSCAFG00845000645	41	35	33	37
ENSCAFG00845000642	888	847	715	860
ENSCAFG00845000643	0	0	0	0
ENSCAFG00845000640	0	1	0	0
ENSCAFG00845000641	0	0	0	0
ENSCAFG00845012619	0	0	0	2
ENSCAFG00845012618	1215	1215	1070	1093
ENSCAFG00845012615	852	772	875	951
ENSCAFG00845012614	0	1	0	0
ENSCAFG00845012617	10	16	15	11
ENSCAFG00845012616	117	124	135	124
ENSCAFG00845012611	0	0	0	0
ENSCAFG00845012610	330	306	320	246
ENSCAFG00845012613	0	0	0	0
ENSCAFG00845012612	0	0	0	0
ENSCAFG00845000619	3	3	4	3
ENSCAFG00845000617	0	0	0	0
ENSCAFG00845000618	0	0	0	0
ENSCAFG00845000615	0	0	0	0
ENSCAFG00845000616	507	400	351	334
ENSCAFG00845000613	0	0	0	0
ENSCAFG00845000614	1	0	3	0
ENSCAFG00845000611	0	0	0	0
ENSCAFG00845000612	0	0	0	0
ENSCAFG00845000610	0	0	0	0
ENSCAFG00845012608	0	0	0	0
ENSCAFG00845012607	0	0	0	0
ENSCAFG00845012609	78	89	62	53
ENSCAFG00845012604	0	0	0	0
ENSCAFG00845012603	0	1	0	1
ENSCAFG00845012606	0	0	2	0
ENSCAFG00845012605	218	205	241	217
ENSCAFG00845012600	78	75	110	77
ENSCAFG00845012602	2299	2308	2155	2089
ENSCAFG00845012601	480	461	479	450
ENSCAFG00845000628	28	32	18	13
ENSCAFG00845000629	1806	1703	1624	1675
ENSCAFG00845000626	0	0	0	0
ENSCAFG00845000627	5	4	3	4
ENSCAFG00845000624	0	1	1	0

ENSCAFG00845000625	5146	5122	4930	5327
ENSCAFG00845000622	0	0	1	0
ENSCAFG00845000623	39	48	72	52
ENSCAFG00845000620	0	0	0	0
ENSCAFG00845000621	93	77	60	68
ENSCAFG00845000608	234	239	300	252
ENSCAFG00845000609	3	1	3	0
ENSCAFG00845000606	0	0	0	0
ENSCAFG00845000607	5864	5925	13528	13514
ENSCAFG00845000604	229	217	210	217
ENSCAFG00845000605	202	200	187	206
ENSCAFG00845000602	0	0	0	1
ENSCAFG00845000603	0	0	0	0
ENSCAFG00845000600	736	742	712	749
ENSCAFG00845000601	0	0	0	0
ENSCAFG00845012581	0	0	0	0
ENSCAFG00845012580	0	0	0	0
ENSCAFG00845012583	560	628	514	520
ENSCAFG00845012582	138	146	184	168
ENSCAFG00845024567	0	0	2	2
ENSCAFG00845024568	75	47	46	55
ENSCAFG00845000598	11	8	12	23
ENSCAFG00845024569	0	0	0	0
ENSCAFG00845000599	336	340	309	352
ENSCAFG00845000596	0	0	0	0
ENSCAFG00845024563	0	0	0	0
ENSCAFG00845000597	563	482	469	470
ENSCAFG00845024564	0	0	0	0
ENSCAFG00845000594	0	0	0	0
ENSCAFG00845024565	0	0	0	1
ENSCAFG00845000595	6	17	18	10
ENSCAFG00845024566	3	2	2	3
ENSCAFG00845000592	0	2	2	3
ENSCAFG00845000593	90	107	97	87
ENSCAFG00845024560	21	23	37	40
ENSCAFG00845000590	514	474	366	389
ENSCAFG00845024561	0	0	0	1
ENSCAFG00845000591	0	0	0	0
ENSCAFG00845024562	1247	1163	1158	1147
ENSCAFG00845012589	918	824	921	915
ENSCAFG00845012588	0	0	0	0

ENSCAFG00845012585	13	1	8	8
ENSCAFG00845012584	0	0	0	0
ENSCAFG00845012587	921	818	867	874
ENSCAFG00845012586	632	645	573	687
ENSCAFG00845012570	0	0	0	0
ENSCAFG00845012572	0	0	0	0
ENSCAFG00845012571	0	0	0	0
ENSCAFG00845024556	394	390	397	413
ENSCAFG00845024557	22	21	18	34
ENSCAFG00845024558	0	0	0	0
ENSCAFG00845024559	18	11	26	23
ENSCAFG00845024554	9048	8641	8337	8486
ENSCAFG00845024550	0	0	0	0
ENSCAFG00845024551	0	0	0	0
ENSCAFG00845012578	0	0	0	0
ENSCAFG00845012577	66	55	86	82
ENSCAFG00845012579	0	0	0	0
ENSCAFG00845012574	207	171	161	147
ENSCAFG00845012573	0	1	0	1
ENSCAFG00845012576	0	0	0	0
ENSCAFG00845012575	1525	1535	1622	1589
ENSCAFG00845012561	0	0	0	0
ENSCAFG00845012560	1055	1026	1129	1086
ENSCAFG00845024549	2	0	0	0
ENSCAFG00845000578	340	330	313	268
ENSCAFG00845024545	0	0	0	1
ENSCAFG00845000579	1135	1135	985	1094
ENSCAFG00845024546	0	0	0	0
ENSCAFG00845000576	342	331	273	312
ENSCAFG00845024547	623	540	538	481
ENSCAFG00845000577	57	75	70	63
ENSCAFG00845024548	0	0	1	0
ENSCAFG00845000574	0	0	0	0
ENSCAFG00845024541	1	0	0	0
ENSCAFG00845000575	1	0	1	0
ENSCAFG00845024542	0	0	0	0
ENSCAFG00845000572	300	284	303	268
ENSCAFG00845024543	880	952	811	857
ENSCAFG00845000573	97	87	106	70
ENSCAFG00845024544	1233	1317	1342	1412
ENSCAFG00845000570	0	0	0	1

ENSCAFG00845000571	1	1	1	0
ENSCAFG00845024540	0	1	0	0
ENSCAFG00845012567	0	0	0	1
ENSCAFG00845012566	0	0	0	1
ENSCAFG00845012569	0	1	0	0
ENSCAFG00845012568	0	0	0	0
ENSCAFG00845012563	0	0	0	0
ENSCAFG00845012562	967	876	994	971
ENSCAFG00845012565	0	0	0	0
ENSCAFG00845012564	2	4	0	0
ENSCAFG00845012550	0	0	1	0
ENSCAFG00845024538	18	7	13	6
ENSCAFG00845024539	0	0	0	0
ENSCAFG00845000589	1304	1170	1185	1201
ENSCAFG00845024534	0	0	0	0
ENSCAFG00845024535	5	2	0	0
ENSCAFG00845000587	5	1	7	9
ENSCAFG00845024536	10	20	29	16
ENSCAFG00845000588	0	0	0	0
ENSCAFG00845024537	0	0	0	0
ENSCAFG00845000585	65	67	84	77
ENSCAFG00845024530	0	0	0	0
ENSCAFG00845000586	659	639	618	644
ENSCAFG00845000583	0	2	1	0
ENSCAFG00845024532	0	0	0	0
ENSCAFG00845000584	0	0	0	0
ENSCAFG00845024533	1	2	2	1
ENSCAFG00845000581	13	6	4	12
ENSCAFG00845000582	0	0	0	0
ENSCAFG00845000580	967	894	798	821
ENSCAFG00845012559	2	0	0	1
ENSCAFG00845012556	0	0	0	0
ENSCAFG00845012555	2295	2244	2246	2182
ENSCAFG00845012558	12	24	9	28
ENSCAFG00845012557	0	0	0	0
ENSCAFG00845012552	7	20	11	20
ENSCAFG00845012551	0	0	0	0
ENSCAFG00845012554	0	2	0	0
ENSCAFG00845012553	0	0	0	0
ENSCAFG00845024527	0	0	0	0
ENSCAFG00845024528	0	1	0	0

ENSCAFG00845000558	0	0	0	0
ENSCAFG00845024529	2	4	4	3
ENSCAFG00845000559	0	0	0	0
ENSCAFG00845000556	1	1	0	0
ENSCAFG00845024523	0	0	0	3
ENSCAFG00845000557	0	0	0	0
ENSCAFG00845024524	568	581	577	557
ENSCAFG00845000554	0	0	0	0
ENSCAFG00845024525	0	0	0	0
ENSCAFG00845000555	13	3	6	4
ENSCAFG00845024526	0	0	0	0
ENSCAFG00845000552	0	0	0	0
ENSCAFG00845000553	1925	1860	1782	1769
ENSCAFG00845024520	0	0	0	0
ENSCAFG00845000550	2	0	3	1
ENSCAFG00845024521	0	0	0	0
ENSCAFG00845000551	0	0	0	0
ENSCAFG00845024522	954	918	1052	999
ENSCAFG00845012549	20	25	36	26
ENSCAFG00845012548	86	58	78	67
ENSCAFG00845012545	0	0	0	0
ENSCAFG00845012544	2	2	1	0
ENSCAFG00845012547	0	0	0	0
ENSCAFG00845012546	0	0	0	0
ENSCAFG00845012541	697	615	730	771
ENSCAFG00845012540	0	0	0	0
ENSCAFG00845012543	44	37	32	41
ENSCAFG00845012542	0	0	0	0
ENSCAFG00845024516	0	0	0	0
ENSCAFG00845024517	665	723	717	670
ENSCAFG00845000569	874	885	796	796
ENSCAFG00845024518	0	0	0	0
ENSCAFG00845000567	0	0	0	0
ENSCAFG00845024512	1	0	0	1
ENSCAFG00845000568	28	24	31	49
ENSCAFG00845024513	0	0	0	0
ENSCAFG00845000565	0	0	0	0
ENSCAFG00845024514	378	302	332	336
ENSCAFG00845000566	2	2	0	1
ENSCAFG00845024515	1	0	0	0
ENSCAFG00845000563	116	104	104	90

ENSCAFG00845000564	543	474	499	538
ENSCAFG00845000561	19	16	17	13
ENSCAFG00845024510	781	788	708	685
ENSCAFG00845000562	0	2	3	0
ENSCAFG00845024511	0	0	0	0
ENSCAFG00845000560	2135	2099	1954	1939
ENSCAFG00845012538	0	0	0	0
ENSCAFG00845012537	650	605	512	511
ENSCAFG00845012539	0	0	0	0
ENSCAFG00845012534	0	0	0	0
ENSCAFG00845012533	0	0	0	0
ENSCAFG00845012536	304	254	245	256
ENSCAFG00845012535	0	0	0	0
ENSCAFG00845012530	0	0	0	1
ENSCAFG00845012532	2177	2123	2272	2461
ENSCAFG00845012531	380	350	398	438
ENSCAFG00845024509	0	0	0	0
ENSCAFG00845000538	0	0	0	0
ENSCAFG00845000539	10	18	11	13
ENSCAFG00845000536	0	0	0	0
ENSCAFG00845024507	115	102	122	106
ENSCAFG00845000537	1	0	2	0
ENSCAFG00845024508	453	442	553	518
ENSCAFG00845000534	15	30	12	9
ENSCAFG00845024501	5	3	12	12
ENSCAFG00845000535	24	31	14	22
ENSCAFG00845024502	0	0	0	0
ENSCAFG00845000532	2	0	0	2
ENSCAFG00845024503	0	0	0	0
ENSCAFG00845000533	54	61	52	60
ENSCAFG00845024504	0	0	0	0
ENSCAFG00845000530	0	0	1	1
ENSCAFG00845000531	4584	4365	5414	5508
ENSCAFG00845024500	1	3	0	2
ENSCAFG00845012527	298	344	312	309
ENSCAFG00845012526	0	0	0	0
ENSCAFG00845012529	1	1	3	1
ENSCAFG00845012528	0	0	0	0
ENSCAFG00845012523	0	0	0	0
ENSCAFG00845012522	0	0	0	0
ENSCAFG00845012525	0	0	0	2

ENSCAFG00845012524	2214	2259	1880	1943
ENSCAFG00845012521	0	0	0	0
ENSCAFG00845012520	6	2	3	0
ENSCAFG00845000549	0	0	0	0
ENSCAFG00845000547	16	7	7	7
ENSCAFG00845000548	17	24	28	17
ENSCAFG00845000545	0	0	0	0
ENSCAFG00845000546	118	103	85	96
ENSCAFG00845000543	448	506	572	492
ENSCAFG00845000544	1	0	0	0
ENSCAFG00845000541	1	0	0	3
ENSCAFG00845000542	0	0	0	0
ENSCAFG00845000540	0	0	0	1
ENSCAFG00845012519	1132	1123	1234	1188
ENSCAFG00845012516	0	0	0	0
ENSCAFG00845012515	0	0	0	0
ENSCAFG00845012518	0	0	0	0
ENSCAFG00845012517	0	0	0	0
ENSCAFG00845012512	0	0	0	0
ENSCAFG00845012511	453	491	370	437
ENSCAFG00845012514	450	476	468	483
ENSCAFG00845012513	2	26	9	19
ENSCAFG00845012510	108	89	103	118
ENSCAFG00845024596	3	8	3	6
ENSCAFG00845024597	773	733	859	759
ENSCAFG00845024598	0	0	0	0
ENSCAFG00845024599	0	0	0	0
ENSCAFG00845024592	0	0	0	0
ENSCAFG00845024593	0	0	0	0
ENSCAFG00845024594	1811	1785	1262	1355
ENSCAFG00845024595	0	0	1	1
ENSCAFG00845024590	50	74	60	81
ENSCAFG00845024591	0	0	0	0
ENSCAFG00845024589	8	12	11	9
ENSCAFG00845024585	2	2	0	2
ENSCAFG00845024586	2770	2532	2876	2989
ENSCAFG00845024587	0	0	0	0
ENSCAFG00845024581	2	1	0	3
ENSCAFG00845024582	1571	1695	1593	1565
ENSCAFG00845024583	1417	1337	1337	1268
ENSCAFG00845024584	0	0	0	0

ENSCAFG00845024580	72	102	91	104
ENSCAFG00845012592	0	2	3	0
ENSCAFG00845012591	0	0	0	0
ENSCAFG00845012594	330	302	332	351
ENSCAFG00845012593	0	0	0	0
ENSCAFG00845012590	0	4	0	0
ENSCAFG00845024578	521	558	510	449
ENSCAFG00845024579	2	0	3	1
ENSCAFG00845024574	0	5	0	3
ENSCAFG00845024575	0	1	0	0
ENSCAFG00845024576	866	904	767	834
ENSCAFG00845024577	351	382	351	320
ENSCAFG00845024571	86	77	76	90
ENSCAFG00845024572	0	1	1	0
ENSCAFG00845024573	480	490	597	616
ENSCAFG00845012599	235	270	227	233
ENSCAFG00845012596	26	30	6	11
ENSCAFG00845012595	0	0	0	0
ENSCAFG00845012598	0	0	0	0
ENSCAFG00845012597	0	0	0	0
ENSCAFG00845024729	5	3	0	1
ENSCAFG00845000758	829	837	778	824
ENSCAFG00845024725	207	199	202	240
ENSCAFG00845000759	1063	985	1187	1157
ENSCAFG00845024726	0	0	0	0
ENSCAFG00845000756	589	604	605	610
ENSCAFG00845024727	1	0	0	0
ENSCAFG00845000757	1302	1287	1267	1255
ENSCAFG00845024728	1554	1442	1367	1388
ENSCAFG00845000754	8	13	10	15
ENSCAFG00845024721	0	0	0	0
ENSCAFG00845000755	13	15	12	19
ENSCAFG00845024722	0	0	1	1
ENSCAFG00845000752	0	0	0	0
ENSCAFG00845024723	1215	1126	1033	1064
ENSCAFG00845000753	3099	2969	3232	3222
ENSCAFG00845024724	2177	2313	2143	2236
ENSCAFG00845000750	2	0	1	1
ENSCAFG00845000751	7	8	4	5
ENSCAFG00845024720	8	9	22	19
ENSCAFG00845012747	134	132	139	139

ENSCAFG00845012746	1057	1050	1164	1059
ENSCAFG00845012749	0	0	0	0
ENSCAFG00845012748	0	0	0	0
ENSCAFG00845012743	395	383	403	336
ENSCAFG00845012742	0	0	0	0
ENSCAFG00845012745	0	0	0	0
ENSCAFG00845012744	36533	35615	31564	31886
ENSCAFG00845012741	0	0	0	0
ENSCAFG00845012740	0	0	0	0
ENSCAFG00845024718	2122	1944	2128	2155
ENSCAFG00845024719	0	0	0	0
ENSCAFG00845000769	323	283	261	263
ENSCAFG00845024714	0	0	0	0
ENSCAFG00845024715	0	0	0	0
ENSCAFG00845000767	10	4	4	2
ENSCAFG00845024716	1877	1857	1737	1741
ENSCAFG00845000768	0	0	0	0
ENSCAFG00845000765	342	368	386	299
ENSCAFG00845024710	1137	992	1019	1136
ENSCAFG00845000766	90	100	78	57
ENSCAFG00845024711	0	1	0	1
ENSCAFG00845000763	27	33	20	22
ENSCAFG00845000764	21	34	13	18
ENSCAFG00845024713	380	380	342	334
ENSCAFG00845000761	1	1	0	7
ENSCAFG00845000762	4	1	3	2
ENSCAFG00845000760	746	747	697	748
ENSCAFG00845012739	0	0	0	0
ENSCAFG00845012736	0	0	0	0
ENSCAFG00845012735	0	0	0	0
ENSCAFG00845012738	12	15	12	14
ENSCAFG00845012737	0	0	0	0
ENSCAFG00845012732	0	0	0	0
ENSCAFG00845012731	0	0	0	0
ENSCAFG00845012734	310	300	336	355
ENSCAFG00845012733	0	0	0	0
ENSCAFG00845012730	0	0	0	0
ENSCAFG00845024707	0	0	0	0
ENSCAFG00845024708	10307	10096	13183	13049
ENSCAFG00845000738	0	0	0	0
ENSCAFG00845024709	211	222	238	232

ENSCAFG00845000739	0	0	0	0
ENSCAFG00845000736	3311	3172	3223	3303
ENSCAFG00845024703	194	180	130	147
ENSCAFG00845000737	7	9	4	3
ENSCAFG00845024704	1519	1434	1391	1471
ENSCAFG00845000734	0	2	0	0
ENSCAFG00845024705	4197	4049	4117	3788
ENSCAFG00845000735	102	128	104	101
ENSCAFG00845024706	0	0	0	0
ENSCAFG00845000732	561	563	570	630
ENSCAFG00845000733	4	2	1	4
ENSCAFG00845024700	2	3	1	4
ENSCAFG00845000730	0	0	0	1
ENSCAFG00845024701	166	168	149	173
ENSCAFG00845000731	661	604	652	615
ENSCAFG00845024702	298	299	304	328
ENSCAFG00845012729	0	1	0	1
ENSCAFG00845012728	0	0	0	0
ENSCAFG00845012725	23	13	10	15
ENSCAFG00845012724	5	0	2	0
ENSCAFG00845012727	303	285	273	316
ENSCAFG00845012726	1062	1031	1200	1222
ENSCAFG00845012721	0	0	0	0
ENSCAFG00845012720	77	93	95	105
ENSCAFG00845012723	0	0	0	0
ENSCAFG00845012722	0	0	0	0
ENSCAFG00845000749	1	0	1	3
ENSCAFG00845000747	0	0	0	0
ENSCAFG00845000748	11	13	12	13
ENSCAFG00845000745	0	0	0	0
ENSCAFG00845000746	0	0	0	0
ENSCAFG00845000743	479	450	527	549
ENSCAFG00845000744	475	409	379	398
ENSCAFG00845000741	242	227	216	218
ENSCAFG00845000742	791	825	959	1014
ENSCAFG00845000740	0	8	2	3
ENSCAFG00845012718	1699	1627	1666	1697
ENSCAFG00845012717	409	378	418	446
ENSCAFG00845012719	0	0	0	0
ENSCAFG00845012714	2	0	2	0
ENSCAFG00845012713	1	4	3	2

ENSCAFG00845012716	288	304	261	289
ENSCAFG00845012715	0	0	0	0
ENSCAFG00845012710	2	0	2	0
ENSCAFG00845012712	0	0	0	0
ENSCAFG00845012711	1622	1454	1576	1623
ENSCAFG00845000718	0	0	0	0
ENSCAFG00845000719	0	0	0	2
ENSCAFG00845000716	1	1	1	1
ENSCAFG00845000717	2418	2238	2355	2416
ENSCAFG00845000714	906	844	923	998
ENSCAFG00845000715	531	433	452	429
ENSCAFG00845000712	288	274	200	268
ENSCAFG00845000713	0	1	0	4
ENSCAFG00845000710	1	0	0	4
ENSCAFG00845000711	50	36	39	37
ENSCAFG00845012707	0	0	0	0
ENSCAFG00845012706	0	0	0	0
ENSCAFG00845012709	0	0	0	0
ENSCAFG00845012708	0	0	0	0
ENSCAFG00845012703	0	0	0	0
ENSCAFG00845012702	0	0	0	0
ENSCAFG00845012705	0	0	0	0
ENSCAFG00845012704	6093	5902	5904	5799
ENSCAFG00845012701	2	0	0	2
ENSCAFG00845012700	4	6	4	2
ENSCAFG00845000709	0	0	3	2
ENSCAFG00845000729	0	0	0	0
ENSCAFG00845000727	84	63	82	81
ENSCAFG00845000728	0	0	0	0
ENSCAFG00845000725	0	0	0	0
ENSCAFG00845000726	0	0	0	0
ENSCAFG00845000723	0	0	0	0
ENSCAFG00845000724	3	0	1	0
ENSCAFG00845000721	256	236	242	224
ENSCAFG00845000722	203	200	200	254
ENSCAFG00845000720	0	0	0	0
ENSCAFG00845000707	166	175	92	81
ENSCAFG00845000708	512	463	600	651
ENSCAFG00845000705	0	0	2	1
ENSCAFG00845000706	374	374	419	405
ENSCAFG00845000703	2587	2459	2250	2254

ENSCAFG00845000704	0	0	2	2
ENSCAFG00845000701	0	0	1	1
ENSCAFG00845000702	6	3	2	2
ENSCAFG00845000700	0	0	0	0
ENSCAFG00845009050	0	0	0	0
ENSCAFG00845009051	252	287	238	203
ENSCAFG00845010040	0	0	0	0
ENSCAFG00845010041	5255	5239	4864	4958
ENSCAFG00845010042	0	0	0	0
ENSCAFG00845009054	0	0	0	0
ENSCAFG00845009055	0	0	0	0
ENSCAFG00845009052	0	0	0	0
ENSCAFG00845009053	3	4	1	1
ENSCAFG00845009058	1596	1503	1620	1483
ENSCAFG00845022025	0	0	0	0
ENSCAFG00845024688	189	183	167	134
ENSCAFG00845009059	1	1	3	6
ENSCAFG00845022024	0	0	0	0
ENSCAFG00845024689	1	2	1	0
ENSCAFG00845009056	2645	2525	2145	2209
ENSCAFG00845022023	306	319	272	312
ENSCAFG00845009057	0	0	0	0
ENSCAFG00845022022	543	643	674	555
ENSCAFG00845022029	5023	4785	5179	5314
ENSCAFG00845024684	1	0	0	0
ENSCAFG00845022028	33	17	7	12
ENSCAFG00845024685	8	2	2	1
ENSCAFG00845022027	0	0	0	0
ENSCAFG00845022026	0	0	1	1
ENSCAFG00845024687	2357	2243	2184	2321
ENSCAFG00845024680	0	0	0	0
ENSCAFG00845024681	0	0	0	0
ENSCAFG00845024682	1	0	1	0
ENSCAFG00845024683	279	236	230	262
ENSCAFG00845022021	475	474	464	515
ENSCAFG00845022020	640	636	632	652
ENSCAFG00845010047	0	4	0	2
ENSCAFG00845010048	0	0	0	0
ENSCAFG00845010049	0	0	1	0
ENSCAFG00845010043	2402	2228	2178	2149
ENSCAFG00845010044	0	0	0	0

ENSCAFG00845010045	21	28	34	16
ENSCAFG00845010046	670	647	644	641
ENSCAFG00845012691	112	118	140	116
ENSCAFG00845009040	464	469	544	557
ENSCAFG00845012690	0	0	0	0
ENSCAFG00845010030	569	543	568	538
ENSCAFG00845012693	0	0	0	0
ENSCAFG00845010031	0	0	0	0
ENSCAFG00845012692	989	885	856	882
ENSCAFG00845022019	545	503	546	523
ENSCAFG00845009043	0	1	2	2
ENSCAFG00845009044	0	0	0	0
ENSCAFG00845009041	0	0	0	0
ENSCAFG00845009042	58	69	44	40
ENSCAFG00845009047	0	0	0	0
ENSCAFG00845022014	315	291	355	301
ENSCAFG00845024677	539	518	505	496
ENSCAFG00845009048	0	0	0	0
ENSCAFG00845022013	0	0	0	0
ENSCAFG00845024678	1	1	0	2
ENSCAFG00845009045	1492	1572	1583	1576
ENSCAFG00845022012	295	255	278	304
ENSCAFG00845024679	0	0	0	0
ENSCAFG00845009046	25	14	26	20
ENSCAFG00845022011	4208	4262	3300	3139
ENSCAFG00845022018	720	757	744	774
ENSCAFG00845024673	13	10	20	19
ENSCAFG00845022017	1823	2004	2006	1918
ENSCAFG00845024674	0	0	0	0
ENSCAFG00845009049	0	0	0	0
ENSCAFG00845022016	585	523	474	492
ENSCAFG00845024675	209	224	291	267
ENSCAFG00845022015	1	0	4	1
ENSCAFG00845024676	0	0	0	0
ENSCAFG00845024670	0	0	0	0
ENSCAFG00845024672	99	86	72	90
ENSCAFG00845022010	0	0	0	0
ENSCAFG00845010036	0	0	0	0
ENSCAFG00845012699	169	154	157	182
ENSCAFG00845010037	0	0	0	0
ENSCAFG00845012698	90	88	92	83

ENSCAFG00845010038	1282	1120	1349	1300
ENSCAFG00845010039	0	0	0	0
ENSCAFG00845010032	2	2	1	1
ENSCAFG00845012695	0	0	0	0
ENSCAFG00845010033	348	369	300	370
ENSCAFG00845012694	295	299	270	304
ENSCAFG00845010034	0	0	0	0
ENSCAFG00845012697	1	2	2	0
ENSCAFG00845010035	0	0	0	1
ENSCAFG00845012696	631	556	562	560
ENSCAFG00845012680	0	1	0	4
ENSCAFG00845012682	808	756	901	843
ENSCAFG00845022009	2	0	0	0
ENSCAFG00845010020	2	1	3	0
ENSCAFG00845012681	0	0	0	0
ENSCAFG00845022008	0	0	0	0
ENSCAFG00845009032	0	0	0	0
ENSCAFG00845009033	10	8	5	7
ENSCAFG00845009030	363	369	348	348
ENSCAFG00845009031	1	1	7	1
ENSCAFG00845000699	0	0	0	0
ENSCAFG00845009036	0	0	3	4
ENSCAFG00845022003	1388	1339	1317	1264
ENSCAFG00845024666	2	2	2	2
ENSCAFG00845009037	1	0	0	0
ENSCAFG00845022002	285	286	278	240
ENSCAFG00845024667	2376	2399	2117	2172
ENSCAFG00845000697	51	73	59	39
ENSCAFG00845009034	1088	1096	1133	1080
ENSCAFG00845022001	6	10	10	6
ENSCAFG00845024668	1	0	0	0
ENSCAFG00845000698	447	461	422	441
ENSCAFG00845009035	9	6	13	7
ENSCAFG00845022000	192	181	166	200
ENSCAFG00845024669	2	2	0	1
ENSCAFG00845000695	234	218	212	187
ENSCAFG00845022007	1164	1148	1032	1008
ENSCAFG00845024662	726	637	668	691
ENSCAFG00845000696	4335	4290	4482	4801
ENSCAFG00845022006	0	0	0	0
ENSCAFG00845024663	485	519	457	495

ENSCAFG00845000693	326	321	302	379
ENSCAFG00845009038	22	11	9	22
ENSCAFG00845024664	318	329	328	366
ENSCAFG00845000694	1	0	0	1
ENSCAFG00845009039	138	99	122	132
ENSCAFG00845022004	0	0	0	1
ENSCAFG00845024665	0	4	0	1
ENSCAFG00845000691	21	5	9	13
ENSCAFG00845000692	689	636	649	691
ENSCAFG00845024660	0	0	0	0
ENSCAFG00845000690	2	6	1	1
ENSCAFG00845024661	1171	1050	1001	1142
ENSCAFG00845010029	0	0	0	0
ENSCAFG00845010025	0	0	0	0
ENSCAFG00845012688	0	0	0	0
ENSCAFG00845010026	1	0	2	1
ENSCAFG00845012687	0	0	0	0
ENSCAFG00845010027	12	9	13	12
ENSCAFG00845010028	0	0	0	0
ENSCAFG00845012689	0	0	0	0
ENSCAFG00845010021	0	7	1	5
ENSCAFG00845012684	85	79	118	103
ENSCAFG00845010022	90	73	78	125
ENSCAFG00845012683	0	0	0	0
ENSCAFG00845010023	36	34	47	33
ENSCAFG00845012686	0	0	0	0
ENSCAFG00845010024	1	3	3	3
ENSCAFG00845012685	420	390	420	414
ENSCAFG00845012671	14	9	12	6
ENSCAFG00845012670	0	0	0	0
ENSCAFG00845009021	2	2	2	5
ENSCAFG00845024659	2	0	5	0
ENSCAFG00845009022	520	374	492	475
ENSCAFG00845009020	0	0	0	0
ENSCAFG00845009025	252	271	212	232
ENSCAFG00845024655	102	103	84	111
ENSCAFG00845009026	5	1	0	1
ENSCAFG00845024656	280	296	248	304
ENSCAFG00845009023	3	1	1	4
ENSCAFG00845024657	0	0	0	0
ENSCAFG00845009024	3154	3309	3440	3435

ENSCAFG00845024658	31	47	21	42
ENSCAFG00845009029	266	273	262	306
ENSCAFG00845024651	1845	1852	1935	1860
ENSCAFG00845024652	0	4	5	3
ENSCAFG00845009027	546	575	546	524
ENSCAFG00845009028	0	0	0	0
ENSCAFG00845024654	173	134	165	157
ENSCAFG00845024650	380	383	447	389
ENSCAFG00845010018	215	177	121	173
ENSCAFG00845010019	594	521	552	589
ENSCAFG00845010014	0	0	0	0
ENSCAFG00845012677	76	73	108	97
ENSCAFG00845010015	1379	1410	1506	1525
ENSCAFG00845012676	0	0	0	0
ENSCAFG00845010016	2038	1900	1797	1909
ENSCAFG00845012679	0	0	0	0
ENSCAFG00845010017	1	2	0	0
ENSCAFG00845012678	0	0	0	0
ENSCAFG00845010010	517	492	569	505
ENSCAFG00845012673	0	0	0	0
ENSCAFG00845010011	3	4	2	2
ENSCAFG00845012672	0	1	0	0
ENSCAFG00845010012	0	0	0	0
ENSCAFG00845012675	0	0	0	0
ENSCAFG00845010013	0	1	0	0
ENSCAFG00845012674	0	0	0	0
ENSCAFG00845012660	1145	1177	1126	1048
ENSCAFG00845009010	1038	939	1017	1070
ENSCAFG00845024648	415	304	281	246
ENSCAFG00845009011	401	460	383	321
ENSCAFG00845024649	40	31	50	36
ENSCAFG00845000679	2	0	2	0
ENSCAFG00845000677	10	5	1	4
ENSCAFG00845009014	3475	3309	3237	2933
ENSCAFG00845024644	713	676	621	661
ENSCAFG00845000678	7	4	10	9
ENSCAFG00845009015	817	727	756	706
ENSCAFG00845024645	757	731	751	717
ENSCAFG00845000675	3	1	0	0
ENSCAFG00845009012	0	0	0	0
ENSCAFG00845024646	0	0	0	0

ENSCAFG00845000676	0	0	0	2
ENSCAFG00845009013	10	9	12	11
ENSCAFG00845024647	2382	2416	2217	2267
ENSCAFG00845000673	933	768	901	869
ENSCAFG00845009018	0	0	2	0
ENSCAFG00845024640	17	13	20	20
ENSCAFG00845000674	0	0	0	0
ENSCAFG00845009019	0	2	0	0
ENSCAFG00845024641	0	0	0	1
ENSCAFG00845000671	0	0	0	0
ENSCAFG00845009016	0	0	0	0
ENSCAFG00845024642	0	0	0	0
ENSCAFG00845000672	0	0	0	0
ENSCAFG00845009017	1	8	6	5
ENSCAFG00845024643	0	0	0	0
ENSCAFG00845000670	1224	1190	1182	1188
ENSCAFG00845010007	853	820	784	802
ENSCAFG00845010008	0	0	0	0
ENSCAFG00845012669	1	1	0	0
ENSCAFG00845010009	0	0	0	0
ENSCAFG00845010003	279	281	252	270
ENSCAFG00845012666	0	0	0	0
ENSCAFG00845010004	0	0	0	0
ENSCAFG00845012665	0	0	0	0
ENSCAFG00845010005	1	7	13	10
ENSCAFG00845012668	672	603	693	694
ENSCAFG00845010006	0	0	0	0
ENSCAFG00845012667	159	163	155	170
ENSCAFG00845012662	11270	10855	11358	11439
ENSCAFG00845010000	821	751	804	744
ENSCAFG00845012661	286	256	228	243
ENSCAFG00845010001	690	621	653	657
ENSCAFG00845012664	0	1	0	0
ENSCAFG00845010002	257	260	286	308
ENSCAFG00845012663	14	17	16	37
ENSCAFG00845024637	0	0	0	0
ENSCAFG00845009000	0	6	8	1
ENSCAFG00845024638	0	0	0	0
ENSCAFG00845024639	2	4	4	4
ENSCAFG00845000688	117	116	175	175
ENSCAFG00845009003	49	59	46	47

ENSCAFG00845024633	80	68	71	83
ENSCAFG00845000689	4	1	5	4
ENSCAFG00845009004	8	2	8	7
ENSCAFG00845024634	0	0	0	0
ENSCAFG00845000686	779	737	719	677
ENSCAFG00845009001	6	4	13	9
ENSCAFG00845024635	426	370	305	413
ENSCAFG00845000687	3	1	2	4
ENSCAFG00845009002	1650	1599	1537	1525
ENSCAFG00845024636	13	18	22	10
ENSCAFG00845000684	0	0	0	0
ENSCAFG00845009007	928	844	809	881
ENSCAFG00845000685	3	7	3	6
ENSCAFG00845009008	21	27	32	32
ENSCAFG00845024630	612	616	578	519
ENSCAFG00845000682	0	0	0	0
ENSCAFG00845009005	144	101	122	149
ENSCAFG00845000683	27	38	36	33
ENSCAFG00845009006	554	546	562	587
ENSCAFG00845024632	25	13	17	13
ENSCAFG00845000680	526	523	504	604
ENSCAFG00845000681	0	2	2	0
ENSCAFG00845009009	779	717	645	707
ENSCAFG00845012659	0	0	0	0
ENSCAFG00845012658	0	0	0	0
ENSCAFG00845012655	0	5	2	3
ENSCAFG00845012654	183	183	185	201
ENSCAFG00845012657	0	0	0	0
ENSCAFG00845012656	824	773	745	719
ENSCAFG00845012651	174	162	144	142
ENSCAFG00845012650	0	1	1	0
ENSCAFG00845012653	0	0	0	0
ENSCAFG00845012652	1	0	0	1
ENSCAFG00845000659	1	2	2	1
ENSCAFG00845024626	0	0	0	0
ENSCAFG00845024627	449	404	431	502
ENSCAFG00845000657	255	257	250	271
ENSCAFG00845000658	689	683	659	570
ENSCAFG00845024629	0	0	0	0
ENSCAFG00845000655	0	0	0	0
ENSCAFG00845024622	0	0	1	0

ENSCAFG00845000656	2	1	2	2
ENSCAFG00845024623	0	1	3	0
ENSCAFG00845000653	9	3	3	8
ENSCAFG00845024624	0	0	0	0
ENSCAFG00845000654	45	45	45	39
ENSCAFG00845024625	66	71	44	40
ENSCAFG00845000651	19	14	17	28
ENSCAFG00845000652	2146	2000	1949	2109
ENSCAFG00845024620	68	71	76	97
ENSCAFG00845000650	0	0	0	0
ENSCAFG00845024621	0	0	0	0
ENSCAFG00845012648	33	33	30	41
ENSCAFG00845012647	820	745	743	676
ENSCAFG00845012649	10	13	11	12
ENSCAFG00845012644	259	272	270	302
ENSCAFG00845012643	0	0	0	0
ENSCAFG00845012646	1046	1014	941	865
ENSCAFG00845012645	4	2	0	0
ENSCAFG00845012640	0	0	0	0
ENSCAFG00845012642	2368	2395	2345	2287
ENSCAFG00845012641	22	17	18	17
ENSCAFG00845024619	5	7	0	3
ENSCAFG00845024615	400	337	333	371
ENSCAFG00845024616	0	0	0	0
ENSCAFG00845000668	0	0	0	0
ENSCAFG00845024617	0	0	0	0
ENSCAFG00845000669	177	160	152	183
ENSCAFG00845024618	0	0	0	0
ENSCAFG00845000666	1416	1461	1462	1472
ENSCAFG00845024611	149	169	167	183
ENSCAFG00845000667	0	0	0	0
ENSCAFG00845024612	8	4	3	1
ENSCAFG00845000664	342	313	332	327
ENSCAFG00845024613	4	1	3	3
ENSCAFG00845000665	1	0	0	0
ENSCAFG00845024614	0	0	0	0
ENSCAFG00845000662	0	0	0	0
ENSCAFG00845000663	0	0	0	2
ENSCAFG00845000660	0	0	0	0
ENSCAFG00845000661	10	8	21	8
ENSCAFG00845012637	2	5	0	2

ENSCAFG00845012636	913	950	1041	1028
ENSCAFG00845012639	4	2	3	3
ENSCAFG00845012638	0	0	0	0
ENSCAFG00845012633	0	0	0	0
ENSCAFG00845012632	0	0	0	0
ENSCAFG00845012635	941	983	955	953
ENSCAFG00845012634	8	3	3	2
ENSCAFG00845012631	11	11	15	5
ENSCAFG00845012630	0	2	0	1
ENSCAFG00845022099	2463	2299	2264	2423
ENSCAFG00845022094	191	148	162	162
ENSCAFG00845022093	194	142	195	203
ENSCAFG00845022092	1115	1076	1191	1227
ENSCAFG00845022091	0	0	0	0
ENSCAFG00845022098	2	0	0	0
ENSCAFG00845022096	0	0	0	0
ENSCAFG00845022095	204	188	148	143
ENSCAFG00845022090	1160	1177	1072	1110
ENSCAFG00845022089	0	0	0	0
ENSCAFG00845022088	183	151	178	185
ENSCAFG00845022083	0	0	0	0
ENSCAFG00845022082	0	0	0	0
ENSCAFG00845022081	32	30	43	55
ENSCAFG00845022080	106	99	68	127
ENSCAFG00845022087	0	0	0	0
ENSCAFG00845022086	0	0	0	0
ENSCAFG00845022085	5437	5304	5052	4977
ENSCAFG00845022084	708	705	752	718
ENSCAFG00845010094	1	2	5	3
ENSCAFG00845010095	153	125	148	149
ENSCAFG00845010096	0	0	0	0
ENSCAFG00845010097	308	337	329	380
ENSCAFG00845010090	0	0	0	0
ENSCAFG00845010091	1179	1190	1079	1107
ENSCAFG00845010092	1740	1658	1185	1268
ENSCAFG00845010093	505	564	469	522
ENSCAFG00845022079	707	692	665	675
ENSCAFG00845022078	254	214	208	231
ENSCAFG00845022077	0	1	0	0
ENSCAFG00845022072	0	0	0	0
ENSCAFG00845022071	0	0	0	0

ENSCAFG00845022070	0	0	0	0
ENSCAFG00845022076	3	2	5	6
ENSCAFG00845022075	9	3	5	5
ENSCAFG00845022074	551	514	496	536
ENSCAFG00845022073	1	1	7	3
ENSCAFG00845010098	3	1	0	4
ENSCAFG00845010099	1241	1201	1145	1205
ENSCAFG00845009094	72	70	73	51
ENSCAFG00845010083	8	8	3	3
ENSCAFG00845009095	763	687	823	724
ENSCAFG00845010084	0	0	0	0
ENSCAFG00845009092	25	10	7	7
ENSCAFG00845010085	1984	1884	2109	2051
ENSCAFG00845009093	0	0	0	0
ENSCAFG00845010086	0	0	0	0
ENSCAFG00845009098	136	135	132	107
ENSCAFG00845009099	4265	3979	3944	3850
ENSCAFG00845010080	867	889	930	1021
ENSCAFG00845009096	0	0	1	1
ENSCAFG00845010081	234	235	181	154
ENSCAFG00845009097	61	62	57	69
ENSCAFG00845010082	1	1	1	0
ENSCAFG00845022069	0	0	0	0
ENSCAFG00845022068	0	0	0	0
ENSCAFG00845022067	3	5	7	10
ENSCAFG00845022066	843	842	793	836
ENSCAFG00845022061	4	7	4	3
ENSCAFG00845022065	0	0	0	0
ENSCAFG00845022064	1317	1258	1317	1309
ENSCAFG00845022063	1192	1194	1098	1131
ENSCAFG00845022062	0	0	0	0
ENSCAFG00845010087	0	0	0	0
ENSCAFG00845010088	1408	1240	1357	1369
ENSCAFG00845010089	0	0	0	0
ENSCAFG00845009083	2	0	1	0
ENSCAFG00845010072	378	353	383	393
ENSCAFG00845009084	2	0	0	0
ENSCAFG00845010073	247	233	244	232
ENSCAFG00845009081	1585	1536	1597	1624
ENSCAFG00845010074	0	0	0	0
ENSCAFG00845009082	591	587	619	607

ENSCAFG00845010075	873	932	1096	1125
ENSCAFG00845009087	140	173	149	160
ENSCAFG00845009088	813	816	722	749
ENSCAFG00845009085	2	1	0	3
ENSCAFG00845010070	1	1	1	0
ENSCAFG00845009086	0	0	0	2
ENSCAFG00845010071	1619	1591	1495	1556
ENSCAFG00845022058	0	0	0	0
ENSCAFG00845022057	0	0	0	0
ENSCAFG00845009089	0	0	0	0
ENSCAFG00845022056	0	0	0	0
ENSCAFG00845022055	0	0	0	0
ENSCAFG00845022059	0	0	1	1
ENSCAFG00845022050	0	3	2	2
ENSCAFG00845022054	52	45	47	53
ENSCAFG00845022053	0	0	0	0
ENSCAFG00845022052	469	477	453	511
ENSCAFG00845022051	0	0	0	0
ENSCAFG00845009090	338	333	324	315
ENSCAFG00845010076	27	45	23	27
ENSCAFG00845009091	0	0	0	0
ENSCAFG00845010077	184	204	239	215
ENSCAFG00845010078	30	22	37	52
ENSCAFG00845010079	546	538	528	526
ENSCAFG00845009072	103	103	87	92
ENSCAFG00845010061	46	51	94	90
ENSCAFG00845009073	0	0	0	0
ENSCAFG00845010062	19	9	23	20
ENSCAFG00845009070	0	0	0	2
ENSCAFG00845010063	2258	2183	1783	1785
ENSCAFG00845009071	0	0	0	0
ENSCAFG00845010064	0	0	0	0
ENSCAFG00845009076	1	0	2	2
ENSCAFG00845009077	2	2	3	5
ENSCAFG00845009074	34	31	26	33
ENSCAFG00845009075	0	1	0	0
ENSCAFG00845010060	142	134	124	121
ENSCAFG00845022047	0	0	0	0
ENSCAFG00845022046	0	0	0	0
ENSCAFG00845009078	3	3	2	0
ENSCAFG00845022045	5575	5364	4945	5099

ENSCAFG00845009079	0	0	0	0
ENSCAFG00845022044	0	0	0	0
ENSCAFG00845022049	0	3	0	1
ENSCAFG00845022048	0	3	1	1
ENSCAFG00845022043	269	275	335	229
ENSCAFG00845022042	0	0	0	0
ENSCAFG00845022041	0	0	0	0
ENSCAFG00845022040	0	0	1	0
ENSCAFG00845010069	1	2	0	0
ENSCAFG00845010065	0	0	0	0
ENSCAFG00845009080	644	582	630	592
ENSCAFG00845010066	151	159	161	159
ENSCAFG00845010067	0	0	0	0
ENSCAFG00845010068	0	0	0	0
ENSCAFG00845009061	1069	1016	1245	1077
ENSCAFG00845010050	0	3	2	0
ENSCAFG00845009062	3120	3019	2981	2966
ENSCAFG00845010051	1	1	1	3
ENSCAFG00845010052	4	4	6	0
ENSCAFG00845009060	0	0	0	0
ENSCAFG00845010053	862	760	799	829
ENSCAFG00845009065	0	0	0	0
ENSCAFG00845009066	4	11	16	16
ENSCAFG00845009063	12	5	8	6
ENSCAFG00845009064	0	0	0	0
ENSCAFG00845009069	1	2	2	0
ENSCAFG00845022036	161	124	198	211
ENSCAFG00845024699	1	0	0	0
ENSCAFG00845022035	18	22	37	23
ENSCAFG00845009067	1197	1200	1123	1157
ENSCAFG00845022034	0	0	0	1
ENSCAFG00845009068	0	0	0	0
ENSCAFG00845022033	2066	2046	2023	2021
ENSCAFG00845024695	855	794	787	936
ENSCAFG00845022039	183	194	140	132
ENSCAFG00845024696	0	3	0	0
ENSCAFG00845022038	0	0	0	0
ENSCAFG00845024697	462	418	454	519
ENSCAFG00845022037	0	0	0	0
ENSCAFG00845024698	0	1	0	0
ENSCAFG00845024691	917	899	914	894

ENSCAFG00845024692	1782	1760	1649	1721
ENSCAFG00845024693	0	0	0	0
ENSCAFG00845024694	203	225	206	224
ENSCAFG00845022031	1	0	1	0
ENSCAFG00845022030	1	3	4	8
ENSCAFG00845024690	0	0	0	0
ENSCAFG00845010058	668	520	592	618
ENSCAFG00845010059	0	0	0	0
ENSCAFG00845010054	126	120	107	110
ENSCAFG00845010055	11	10	11	14
ENSCAFG00845010056	0	0	0	0
ENSCAFG00845010057	0	0	0	0
ENSCAFG00845000806	0	2	1	1
ENSCAFG00845000807	2	5	3	1
ENSCAFG00845000804	0	0	0	0
ENSCAFG00845000805	0	0	0	0
ENSCAFG00845000802	1156	1035	1143	1216
ENSCAFG00845000803	17	20	18	14
ENSCAFG00845000800	63	65	50	65
ENSCAFG00845000801	66	56	44	69
ENSCAFG00845000879	191	166	164	136
ENSCAFG00845024846	32	42	26	21
ENSCAFG00845024847	5	0	0	3
ENSCAFG00845000877	460	412	452	546
ENSCAFG00845024848	0	0	0	0
ENSCAFG00845000878	2280	2278	2251	2397
ENSCAFG00845024849	0	0	0	0
ENSCAFG00845000875	1	1	0	2
ENSCAFG00845009212	0	0	0	0
ENSCAFG00845024842	4	0	6	10
ENSCAFG00845000876	709	712	639	645
ENSCAFG00845009213	109	104	92	87
ENSCAFG00845024843	365	337	327	338
ENSCAFG00845000873	1378	1276	1247	1323
ENSCAFG00845009210	100	107	48	99
ENSCAFG00845024844	0	0	0	0
ENSCAFG00845000874	0	0	0	0
ENSCAFG00845009211	117	109	96	91
ENSCAFG00845000871	3	6	4	4
ENSCAFG00845009216	0	0	0	0
ENSCAFG00845000872	0	0	0	0

ENSCAFG00845009217	0	0	0	1
ENSCAFG00845009214	239	227	225	265
ENSCAFG00845024840	261	215	201	184
ENSCAFG00845000870	6	4	8	8
ENSCAFG00845009215	8	14	15	11
ENSCAFG00845024841	0	0	0	0
ENSCAFG00845010209	0	0	0	0
ENSCAFG00845009218	0	0	0	0
ENSCAFG00845009219	74	52	66	53
ENSCAFG00845010205	1	0	0	0
ENSCAFG00845012868	0	0	0	0
ENSCAFG00845010206	1	0	0	4
ENSCAFG00845012867	0	0	0	0
ENSCAFG00845010207	51	49	57	56
ENSCAFG00845010208	30	35	52	32
ENSCAFG00845012869	392	429	351	416
ENSCAFG00845010201	0	0	0	0
ENSCAFG00845012864	0	1	0	0
ENSCAFG00845010202	12	10	5	9
ENSCAFG00845012863	877	858	781	793
ENSCAFG00845010203	237	248	192	252
ENSCAFG00845012866	0	0	0	0
ENSCAFG00845010204	1253	1195	1158	1227
ENSCAFG00845012865	0	0	0	0
ENSCAFG00845012860	0	0	0	0
ENSCAFG00845012862	86	72	92	82
ENSCAFG00845010200	0	0	0	0
ENSCAFG00845012861	658	647	625	683
ENSCAFG00845024839	881	845	781	847
ENSCAFG00845024835	1	3	0	1
ENSCAFG00845024836	0	0	0	0
ENSCAFG00845000888	32176	31652	31804	30872
ENSCAFG00845024837	0	0	0	0
ENSCAFG00845000889	18	14	6	11
ENSCAFG00845024838	2	5	2	9
ENSCAFG00845000886	0	0	1	1
ENSCAFG00845009201	2	1	0	1
ENSCAFG00845024831	0	0	1	0
ENSCAFG00845000887	1752	1630	1507	1612
ENSCAFG00845009202	0	0	0	0
ENSCAFG00845000884	1001	993	1008	1032

ENSCAFG00845024833	3	6	7	3
ENSCAFG00845000885	0	0	0	0
ENSCAFG00845009200	0	2	0	0
ENSCAFG00845024834	0	0	0	0
ENSCAFG00845000882	77	89	69	51
ENSCAFG00845009205	3374	3278	3120	3314
ENSCAFG00845000883	624	646	642	641
ENSCAFG00845009206	0	0	0	0
ENSCAFG00845000880	785	757	815	795
ENSCAFG00845009203	840	715	874	844
ENSCAFG00845000881	0	0	0	0
ENSCAFG00845009204	374	356	347	347
ENSCAFG00845009209	0	0	0	0
ENSCAFG00845009207	8	5	5	3
ENSCAFG00845009208	59	47	20	29
ENSCAFG00845012857	187	174	195	163
ENSCAFG00845012856	236	188	204	209
ENSCAFG00845012859	922	816	811	822
ENSCAFG00845012858	196	171	157	218
ENSCAFG00845012853	1	0	0	0
ENSCAFG00845012852	117	125	101	138
ENSCAFG00845012855	410	401	351	397
ENSCAFG00845012854	984	781	664	733
ENSCAFG00845012851	0	0	0	0
ENSCAFG00845012850	3	3	4	2
ENSCAFG00845024828	0	0	0	0
ENSCAFG00845024829	785	629	864	953
ENSCAFG00845000859	0	3	2	0
ENSCAFG00845000857	792	727	756	794
ENSCAFG00845024824	0	0	0	0
ENSCAFG00845000858	6	8	2	6
ENSCAFG00845024825	1897	1824	1795	1796
ENSCAFG00845000855	3	0	1	1
ENSCAFG00845024826	448	399	340	396
ENSCAFG00845000856	35	42	40	40
ENSCAFG00845024827	4	0	0	1
ENSCAFG00845000853	0	0	0	0
ENSCAFG00845024820	5	2	4	3
ENSCAFG00845000854	0	0	0	0
ENSCAFG00845024821	0	2	3	1
ENSCAFG00845000851	39	42	81	40

ENSCAFG00845000852	1245	1250	1266	1251
ENSCAFG00845024823	205	177	190	201
ENSCAFG00845000850	76	45	63	55
ENSCAFG00845012849	0	0	0	0
ENSCAFG00845012846	14	21	31	19
ENSCAFG00845012845	471	441	365	393
ENSCAFG00845012848	1	1	0	0
ENSCAFG00845012847	318	271	252	265
ENSCAFG00845012842	10	7	8	17
ENSCAFG00845012841	599	649	548	580
ENSCAFG00845012844	0	0	0	0
ENSCAFG00845012843	1251	1203	1163	1116
ENSCAFG00845012840	508	411	427	417
ENSCAFG00845024817	0	0	0	0
ENSCAFG00845024818	7	4	5	2
ENSCAFG00845024819	596	408	548	514
ENSCAFG00845000868	0	0	0	3
ENSCAFG00845024813	4	8	1	5
ENSCAFG00845000869	1044	984	874	962
ENSCAFG00845024814	0	0	0	0
ENSCAFG00845000866	2053	1835	1892	1964
ENSCAFG00845024815	9	9	14	9
ENSCAFG00845000867	0	0	0	0
ENSCAFG00845024816	50	47	49	40
ENSCAFG00845000864	57	49	49	43
ENSCAFG00845000865	346	367	402	380
ENSCAFG00845024810	453	436	459	446
ENSCAFG00845000862	2	0	0	0
ENSCAFG00845024811	66	49	56	40
ENSCAFG00845000863	11	6	8	7
ENSCAFG00845000860	16	9	7	16
ENSCAFG00845000861	0	0	0	0
ENSCAFG00845012839	137	126	62	69
ENSCAFG00845012838	0	0	0	0
ENSCAFG00845012835	383	422	449	415
ENSCAFG00845012834	0	0	0	0
ENSCAFG00845012837	151	151	133	157
ENSCAFG00845012836	40	41	36	30
ENSCAFG00845012831	143	133	134	134
ENSCAFG00845012830	0	0	0	0
ENSCAFG00845012833	0	0	0	0

ENSCAFG00845012832	0	0	0	0
ENSCAFG00845000839	843	789	705	749
ENSCAFG00845024806	10	1	0	2
ENSCAFG00845024807	145	144	173	223
ENSCAFG00845000837	0	0	0	0
ENSCAFG00845024808	1103	1085	968	1181
ENSCAFG00845000838	0	0	0	0
ENSCAFG00845024809	78	68	68	86
ENSCAFG00845000835	3	1	0	3
ENSCAFG00845024802	107	106	116	116
ENSCAFG00845000836	90	98	96	102
ENSCAFG00845024803	0	0	0	0
ENSCAFG00845000833	314	300	259	249
ENSCAFG00845024804	0	1	0	0
ENSCAFG00845000834	1	0	0	0
ENSCAFG00845024805	484	477	461	469
ENSCAFG00845000831	123	133	110	123
ENSCAFG00845000832	0	0	0	2
ENSCAFG00845024800	0	0	0	0
ENSCAFG00845000830	2383	2263	2330	2450
ENSCAFG00845024801	1508	1547	2077	2054
ENSCAFG00845012828	135	150	136	181
ENSCAFG00845012827	908	893	865	905
ENSCAFG00845012829	2	6	15	7
ENSCAFG00845012824	1	5	3	3
ENSCAFG00845012823	0	0	0	0
ENSCAFG00845012826	13	26	20	23
ENSCAFG00845012825	0	0	0	0
ENSCAFG00845012820	0	2	0	0
ENSCAFG00845012822	0	0	0	0
ENSCAFG00845012821	818	846	739	859
ENSCAFG00845000848	1141	1133	1098	948
ENSCAFG00845000849	120	112	74	95
ENSCAFG00845000846	2946	2915	2348	2467
ENSCAFG00845000847	619	582	659	652
ENSCAFG00845000844	2097	1980	1762	1778
ENSCAFG00845000845	52	55	40	45
ENSCAFG00845000842	26	15	23	26
ENSCAFG00845000843	148	192	172	145
ENSCAFG00845000840	0	0	0	0
ENSCAFG00845000841	2346	2354	2274	2451

ENSCAFG00845012809	10	7	6	3
ENSCAFG00845012817	1	0	0	0
ENSCAFG00845012816	143	145	122	145
ENSCAFG00845012819	60	58	57	48
ENSCAFG00845012818	0	1	0	1
ENSCAFG00845012813	8	11	9	16
ENSCAFG00845012812	3404	3235	3770	3652
ENSCAFG00845012815	0	0	0	0
ENSCAFG00845012814	0	0	0	0
ENSCAFG00845012811	0	0	0	0
ENSCAFG00845012810	0	0	1	6
ENSCAFG00845000817	0	0	0	2
ENSCAFG00845000818	375	388	367	362
ENSCAFG00845000815	274	244	232	240
ENSCAFG00845000816	1179	1110	989	1011
ENSCAFG00845000813	0	1	3	0
ENSCAFG00845000814	2	0	1	0
ENSCAFG00845000811	727	700	734	827
ENSCAFG00845000812	159	168	149	195
ENSCAFG00845000810	0	0	0	0
ENSCAFG00845012806	0	0	0	0
ENSCAFG00845012805	0	0	0	0
ENSCAFG00845012808	710	749	648	622
ENSCAFG00845012807	0	0	1	5
ENSCAFG00845012802	1	0	0	0
ENSCAFG00845012801	122	76	117	101
ENSCAFG00845012804	1073	987	1056	1083
ENSCAFG00845012803	0	0	0	0
ENSCAFG00845012800	0	0	0	0
ENSCAFG00845000808	1047	1081	966	1052
ENSCAFG00845000809	38	35	62	68
ENSCAFG00845000828	0	6	0	2
ENSCAFG00845000829	358	406	332	336
ENSCAFG00845000826	29	9	20	11
ENSCAFG00845000827	197	186	196	191
ENSCAFG00845000824	0	0	0	0
ENSCAFG00845000825	3712	3723	3538	3588
ENSCAFG00845000822	0	0	0	0
ENSCAFG00845000823	876	900	862	909
ENSCAFG00845000820	0	0	0	0
ENSCAFG00845000821	83	63	87	103

ENSCAFG00845000819	0	1	3	0
ENSCAFG00845009171	0	3	3	1
ENSCAFG00845010160	165	155	149	163
ENSCAFG00845009172	4	3	4	0
ENSCAFG00845010161	0	0	0	0
ENSCAFG00845010162	0	0	0	0
ENSCAFG00845009170	0	0	0	0
ENSCAFG00845010163	20894	20670	19122	18749
ENSCAFG00845009175	2	1	0	3
ENSCAFG00845009176	0	0	0	0
ENSCAFG00845009173	158	176	216	176
ENSCAFG00845009174	554	503	538	510
ENSCAFG00845009179	0	0	0	0
ENSCAFG00845022146	26	30	25	20
ENSCAFG00845022145	0	0	0	0
ENSCAFG00845009177	903	840	920	901
ENSCAFG00845022144	3252	3211	2539	2814
ENSCAFG00845009178	2158	1903	1865	2037
ENSCAFG00845022143	1	0	0	0
ENSCAFG00845022149	0	0	0	0
ENSCAFG00845022148	829	789	867	949
ENSCAFG00845022147	2573	2515	2590	2771
ENSCAFG00845022142	665	628	681	699
ENSCAFG00845022141	390	337	367	369
ENSCAFG00845022140	147	133	148	131
ENSCAFG00845010168	2	0	0	0
ENSCAFG00845010169	5	1	2	1
ENSCAFG00845010164	385	344	332	332
ENSCAFG00845010165	0	0	5	0
ENSCAFG00845010166	7	6	4	4
ENSCAFG00845010167	0	0	0	0
ENSCAFG00845009160	0	0	0	0
ENSCAFG00845009161	28	24	27	32
ENSCAFG00845010150	1	1	3	4
ENSCAFG00845010151	2	2	0	2
ENSCAFG00845010152	522	498	543	589
ENSCAFG00845009164	0	0	0	0
ENSCAFG00845009165	465	472	359	460
ENSCAFG00845009162	1301	1246	1077	1105
ENSCAFG00845009163	1182	1114	1250	1218
ENSCAFG00845009168	0	0	0	0

ENSCAFG00845022135	0	0	0	0
ENSCAFG00845024798	422	462	376	361
ENSCAFG00845009169	0	0	0	0
ENSCAFG00845022134	0	0	0	0
ENSCAFG00845024799	5	1	1	6
ENSCAFG00845009166	7	9	8	7
ENSCAFG00845022133	0	0	0	0
ENSCAFG00845009167	0	0	0	0
ENSCAFG00845022132	115	91	106	100
ENSCAFG00845022139	0	0	0	0
ENSCAFG00845024794	432	412	379	449
ENSCAFG00845022138	0	0	0	0
ENSCAFG00845024795	5	0	0	0
ENSCAFG00845022137	443	433	419	452
ENSCAFG00845024796	9	6	28	10
ENSCAFG00845022136	802	701	738	754
ENSCAFG00845024797	0	2	2	0
ENSCAFG00845024790	3717	3604	3834	4070
ENSCAFG00845024791	5	2	0	3
ENSCAFG00845024792	1	0	3	0
ENSCAFG00845024793	967	1001	1024	1042
ENSCAFG00845022131	539	558	501	531
ENSCAFG00845010157	0	3	1	7
ENSCAFG00845010158	336	332	297	316
ENSCAFG00845010159	0	0	0	0
ENSCAFG00845010153	183	162	151	155
ENSCAFG00845010154	1	5	3	7
ENSCAFG00845010155	0	0	0	0
ENSCAFG00845010156	0	2	2	5
ENSCAFG00845009150	4	3	2	2
ENSCAFG00845010140	0	0	0	0
ENSCAFG00845010141	264	278	195	225
ENSCAFG00845022129	126	133	58	56
ENSCAFG00845009153	4135	3788	4063	4101
ENSCAFG00845009154	0	0	0	0
ENSCAFG00845009151	318	323	244	259
ENSCAFG00845009152	1810	1802	1609	1626
ENSCAFG00845009157	20	25	28	18
ENSCAFG00845022124	11	12	14	15
ENSCAFG00845009158	0	0	0	0
ENSCAFG00845022123	0	0	0	0

ENSCAFG00845024788	3808	3555	3854	3732
ENSCAFG00845009155	1072	990	916	936
ENSCAFG00845022122	289	227	221	206
ENSCAFG00845024789	0	0	0	0
ENSCAFG00845009156	221	202	209	235
ENSCAFG00845022121	1045	979	1241	1063
ENSCAFG00845022128	0	0	0	0
ENSCAFG00845024783	1	1	1	4
ENSCAFG00845022127	0	0	0	0
ENSCAFG00845024784	371	318	290	323
ENSCAFG00845009159	1049	1013	876	930
ENSCAFG00845022126	457	483	637	654
ENSCAFG00845024785	0	0	0	0
ENSCAFG00845022125	0	0	1	0
ENSCAFG00845024786	1223	1130	842	763
ENSCAFG00845024780	183	185	224	193
ENSCAFG00845024781	0	0	0	0
ENSCAFG00845024782	2285	2182	2164	2330
ENSCAFG00845022120	538	479	629	586
ENSCAFG00845010146	440	426	478	458
ENSCAFG00845010147	6	6	1	3
ENSCAFG00845010148	776	716	767	888
ENSCAFG00845010149	13	17	14	11
ENSCAFG00845010142	300	285	345	341
ENSCAFG00845010143	0	2	2	1
ENSCAFG00845010144	330	268	336	327
ENSCAFG00845010145	626	607	468	471
ENSCAFG00845012790	1487	1381	1396	1274
ENSCAFG00845012792	0	0	0	0
ENSCAFG00845010130	14	13	3	10
ENSCAFG00845012791	9517	9375	9055	9507
ENSCAFG00845022118	4	6	4	8
ENSCAFG00845009142	108	116	109	93
ENSCAFG00845009143	0	0	0	0
ENSCAFG00845009140	1	2	2	0
ENSCAFG00845009141	1	0	0	0
ENSCAFG00845009146	336	328	345	346
ENSCAFG00845022113	324	278	250	275
ENSCAFG00845024776	11	7	10	2
ENSCAFG00845009147	462	455	467	450
ENSCAFG00845022112	0	0	0	0

ENSCAFG00845024777	2	0	0	1
ENSCAFG00845009144	0	0	0	0
ENSCAFG00845022111	630	593	717	631
ENSCAFG00845024778	2340	2262	2071	2173
ENSCAFG00845009145	845	937	777	855
ENSCAFG00845022110	992	1131	869	930
ENSCAFG00845024779	0	0	0	0
ENSCAFG00845022117	0	0	0	0
ENSCAFG00845024772	0	4	0	6
ENSCAFG00845022116	1114	1128	1035	1054
ENSCAFG00845024773	1228	1253	1114	1147
ENSCAFG00845009148	6	10	4	7
ENSCAFG00845022115	18	15	18	27
ENSCAFG00845024774	0	0	0	0
ENSCAFG00845009149	1128	1058	1405	1493
ENSCAFG00845022114	202	190	172	172
ENSCAFG00845024775	45	32	34	42
ENSCAFG00845024770	0	0	0	0
ENSCAFG00845024771	1	0	1	0
ENSCAFG00845010139	0	0	0	0
ENSCAFG00845010135	1	0	2	0
ENSCAFG00845012798	940	841	862	844
ENSCAFG00845010136	799	773	846	858
ENSCAFG00845012797	16	16	25	16
ENSCAFG00845010137	87	65	73	96
ENSCAFG00845010138	0	0	0	0
ENSCAFG00845012799	0	1	0	0
ENSCAFG00845010131	0	0	0	0
ENSCAFG00845012794	19	14	26	17
ENSCAFG00845010132	18	13	2	6
ENSCAFG00845012793	403	371	335	370
ENSCAFG00845010133	0	0	0	0
ENSCAFG00845012796	1528	1516	1542	1407
ENSCAFG00845010134	0	0	0	0
ENSCAFG00845012795	2	2	2	1
ENSCAFG00845022109	7	1	0	0
ENSCAFG00845012781	0	0	0	0
ENSCAFG00845022108	1966	1799	2035	1966
ENSCAFG00845012780	0	0	0	0
ENSCAFG00845022107	0	0	0	0
ENSCAFG00845009131	5	2	5	7

ENSCAFG00845024769	2011	1980	1945	2024
ENSCAFG00845009132	1114	1208	1160	1238
ENSCAFG00845009130	334	315	281	294
ENSCAFG00845000798	0	0	0	1
ENSCAFG00845009135	2485	2252	2433	2415
ENSCAFG00845022102	4565	4371	4426	4402
ENSCAFG00845024765	3	0	0	3
ENSCAFG00845000799	7808	7613	7203	7340
ENSCAFG00845009136	752	630	745	818
ENSCAFG00845022101	0	0	0	0
ENSCAFG00845024766	1	0	4	3
ENSCAFG00845000796	1368	1256	1283	1335
ENSCAFG00845009133	581	609	589	604
ENSCAFG00845022100	5153	4869	4595	4698
ENSCAFG00845024767	0	0	0	1
ENSCAFG00845000797	0	0	1	1
ENSCAFG00845009134	1	0	0	0
ENSCAFG00845024768	0	0	0	0
ENSCAFG00845000794	144	166	160	172
ENSCAFG00845009139	254	239	294	299
ENSCAFG00845022106	549	571	611	587
ENSCAFG00845024761	315	329	302	320
ENSCAFG00845000795	5418	5328	4533	4546
ENSCAFG00845022105	0	0	0	0
ENSCAFG00845024762	0	0	3	1
ENSCAFG00845000792	219	180	134	209
ENSCAFG00845009137	1042	978	949	967
ENSCAFG00845022104	366	360	408	398
ENSCAFG00845024763	694	741	739	701
ENSCAFG00845000793	354	324	304	293
ENSCAFG00845009138	1838	1791	1724	1782
ENSCAFG00845022103	2	1	0	1
ENSCAFG00845024764	0	0	0	0
ENSCAFG00845000790	0	0	0	0
ENSCAFG00845000791	162	141	111	119
ENSCAFG00845024760	180	185	212	184
ENSCAFG00845010128	0	0	0	0
ENSCAFG00845010129	0	0	0	0
ENSCAFG00845010124	47	49	70	76
ENSCAFG00845012787	1	0	0	0
ENSCAFG00845010125	0	0	0	0

ENSCAFG00845012786	0	0	0	0
ENSCAFG00845010126	0	0	0	0
ENSCAFG00845012789	258	202	232	252
ENSCAFG00845010127	1983	1836	1719	1791
ENSCAFG00845012788	3	3	1	3
ENSCAFG00845010120	5	9	2	10
ENSCAFG00845012783	640	625	745	718
ENSCAFG00845010121	271	300	309	305
ENSCAFG00845012782	1	2	4	1
ENSCAFG00845010122	77	91	79	85
ENSCAFG00845012785	2165	2100	2251	2087
ENSCAFG00845010123	106	64	68	96
ENSCAFG00845012784	19	20	22	19
ENSCAFG00845012770	5	1	3	3
ENSCAFG00845009120	0	0	0	0
ENSCAFG00845024758	560	556	578	575
ENSCAFG00845009121	1533	1443	1572	1628
ENSCAFG00845024759	0	0	0	0
ENSCAFG00845009124	570	629	518	515
ENSCAFG00845024754	23	25	27	27
ENSCAFG00845009125	4	8	6	5
ENSCAFG00845024755	0	0	0	0
ENSCAFG00845009122	772	743	809	783
ENSCAFG00845024756	0	0	0	0
ENSCAFG00845009123	2	1	2	4
ENSCAFG00845024757	448	462	447	446
ENSCAFG00845009128	880	849	950	891
ENSCAFG00845024750	4114	3990	4089	4009
ENSCAFG00845009129	301	254	275	281
ENSCAFG00845024751	762	732	789	820
ENSCAFG00845009126	0	0	0	0
ENSCAFG00845024752	218	224	206	196
ENSCAFG00845009127	0	0	0	0
ENSCAFG00845024753	174	205	220	188
ENSCAFG00845010117	46	51	45	60
ENSCAFG00845010118	376	373	414	400
ENSCAFG00845012779	1234	1193	1235	1140
ENSCAFG00845010119	2338	2242	2209	2345
ENSCAFG00845010113	0	0	0	0
ENSCAFG00845012776	4562	4441	4432	4668
ENSCAFG00845010114	0	0	0	0

ENSCAFG00845012775	1683	1623	1701	1545
ENSCAFG00845010115	0	0	0	0
ENSCAFG00845012778	0	0	0	0
ENSCAFG00845010116	2	0	0	0
ENSCAFG00845012777	310	266	247	232
ENSCAFG00845012772	2	0	0	0
ENSCAFG00845010110	0	0	0	0
ENSCAFG00845012771	0	0	2	0
ENSCAFG00845010111	0	0	0	2
ENSCAFG00845012774	0	0	0	0
ENSCAFG00845010112	0	2	1	1
ENSCAFG00845012773	147	148	141	125
ENSCAFG00845009110	0	0	0	0
ENSCAFG00845024748	3	5	1	4
ENSCAFG00845000778	651	648	581	582
ENSCAFG00845024749	75	77	78	63
ENSCAFG00845000779	103	99	107	118
ENSCAFG00845000776	568	516	428	421
ENSCAFG00845009113	43	42	29	39
ENSCAFG00845024743	58	51	88	74
ENSCAFG00845000777	1144	1159	1197	1155
ENSCAFG00845009114	0	0	0	0
ENSCAFG00845024744	4090	4023	3846	4067
ENSCAFG00845000774	1	0	0	0
ENSCAFG00845009111	1	4	0	0
ENSCAFG00845024745	251	260	313	273
ENSCAFG00845000775	171	147	155	177
ENSCAFG00845009112	0	0	0	0
ENSCAFG00845024746	77	76	72	120
ENSCAFG00845000772	0	0	0	0
ENSCAFG00845009117	0	0	0	0
ENSCAFG00845000773	169	141	153	127
ENSCAFG00845009118	0	0	0	0
ENSCAFG00845024740	0	0	0	0
ENSCAFG00845000770	379	360	412	439
ENSCAFG00845009115	360	371	359	345
ENSCAFG00845024741	0	0	0	0
ENSCAFG00845000771	2279	2313	1991	1913
ENSCAFG00845009116	787	701	738	703
ENSCAFG00845024742	0	0	0	0
ENSCAFG00845009119	971	965	837	909

ENSCAFG00845010106	22168	21561	20353	19927
ENSCAFG00845012769	0	0	0	0
ENSCAFG00845010107	15	4	10	8
ENSCAFG00845012768	0	0	0	0
ENSCAFG00845010108	0	0	0	0
ENSCAFG00845010109	0	0	0	0
ENSCAFG00845010102	0	0	0	0
ENSCAFG00845012765	419	339	362	315
ENSCAFG00845010103	188	228	222	219
ENSCAFG00845012764	0	0	0	0
ENSCAFG00845010104	32	17	15	10
ENSCAFG00845012767	0	0	0	0
ENSCAFG00845010105	15	14	22	23
ENSCAFG00845012766	410	384	324	472
ENSCAFG00845012761	572	595	569	545
ENSCAFG00845012760	462	441	496	439
ENSCAFG00845010100	0	0	0	0
ENSCAFG00845012763	37	26	30	34
ENSCAFG00845010101	97	110	113	104
ENSCAFG00845012762	0	0	0	0
ENSCAFG00845024736	0	0	0	0
ENSCAFG00845024737	0	0	0	0
ENSCAFG00845000789	673	619	631	614
ENSCAFG00845024738	0	0	0	2
ENSCAFG00845024739	0	0	0	0
ENSCAFG00845000787	1110	1030	1030	1061
ENSCAFG00845009102	0	0	0	0
ENSCAFG00845024732	1627	1599	1521	1604
ENSCAFG00845000788	4	6	7	8
ENSCAFG00845009103	0	0	0	0
ENSCAFG00845024733	7564	7297	7471	7789
ENSCAFG00845000785	974	1007	932	971
ENSCAFG00845009100	18	12	10	14
ENSCAFG00845024734	12	18	9	9
ENSCAFG00845000786	1	1	2	1
ENSCAFG00845009101	467	536	527	509
ENSCAFG00845024735	3	1	2	10
ENSCAFG00845000783	446	409	400	480
ENSCAFG00845009106	0	1	0	0
ENSCAFG00845000784	436	452	494	447
ENSCAFG00845009107	52	42	44	41

ENSCAFG00845000781	0	0	0	0
ENSCAFG00845009104	130	155	159	152
ENSCAFG00845000782	274	269	312	294
ENSCAFG00845009105	0	0	0	0
ENSCAFG00845024731	1	1	0	0
ENSCAFG00845000780	181	186	189	173
ENSCAFG00845009108	7	6	1	2
ENSCAFG00845009109	3	2	6	2
ENSCAFG00845012758	75	58	99	90
ENSCAFG00845012757	386	401	476	537
ENSCAFG00845012759	386	434	383	449
ENSCAFG00845012754	264	246	228	233
ENSCAFG00845012753	6129	5804	6095	6081
ENSCAFG00845012756	0	0	0	0
ENSCAFG00845012755	0	0	0	0
ENSCAFG00845012750	1139	1191	1128	1133
ENSCAFG00845012752	395	397	396	389
ENSCAFG00845012751	219	244	181	171
ENSCAFG00845022199	7	5	6	6
ENSCAFG00845022198	4467	4334	4319	4262
ENSCAFG00845022193	0	0	0	0
ENSCAFG00845022192	0	0	0	0
ENSCAFG00845022191	2	1	0	3
ENSCAFG00845022190	0	2	2	2
ENSCAFG00845022197	1	0	0	0
ENSCAFG00845022196	1176	1276	1136	1227
ENSCAFG00845022195	0	0	0	1
ENSCAFG00845022194	0	1	0	0
ENSCAFG00845022189	0	0	0	0
ENSCAFG00845022188	497	501	543	626
ENSCAFG00845022187	0	2	0	0
ENSCAFG00845022182	1	2	3	1
ENSCAFG00845022181	5	0	4	5
ENSCAFG00845022180	2287	2172	2175	2370
ENSCAFG00845022186	797	792	752	771
ENSCAFG00845022185	95	124	108	116
ENSCAFG00845022184	2571	2392	2506	2703
ENSCAFG00845022183	0	0	0	0
ENSCAFG00845010193	0	0	0	0
ENSCAFG00845010194	0	0	1	0
ENSCAFG00845010195	0	0	0	0

ENSCAFG00845010196	290	286	358	393
ENSCAFG00845010190	928	937	985	1044
ENSCAFG00845010191	3	3	5	4
ENSCAFG00845010192	0	0	0	0
ENSCAFG00845022179	0	0	0	0
ENSCAFG00845022178	1690	1642	1637	1546
ENSCAFG00845022177	75	43	58	47
ENSCAFG00845022176	1	1	1	5
ENSCAFG00845022171	216	235	222	213
ENSCAFG00845022170	127	133	144	145
ENSCAFG00845022175	1	0	0	0
ENSCAFG00845022174	620	618	541	569
ENSCAFG00845022173	0	0	0	0
ENSCAFG00845010197	5621	5400	4636	4708
ENSCAFG00845010198	0	0	0	0
ENSCAFG00845010199	0	0	0	0
ENSCAFG00845009193	69	64	66	47
ENSCAFG00845010182	0	0	0	0
ENSCAFG00845009194	0	0	0	0
ENSCAFG00845010183	0	0	0	0
ENSCAFG00845009191	486	479	467	471
ENSCAFG00845010184	160	193	184	168
ENSCAFG00845009192	1634	1716	1625	1668
ENSCAFG00845010185	395	421	416	383
ENSCAFG00845009197	6588	6640	6013	6001
ENSCAFG00845009198	2	1	0	2
ENSCAFG00845009195	51	55	71	59
ENSCAFG00845010180	2	3	2	0
ENSCAFG00845009196	1140	1126	1133	1069
ENSCAFG00845010181	470	445	468	495
ENSCAFG00845022168	150	146	158	152
ENSCAFG00845022167	1	2	4	1
ENSCAFG00845009199	2	12	14	7
ENSCAFG00845022166	0	0	0	0
ENSCAFG00845022165	0	0	0	0
ENSCAFG00845022169	409	373	402	408
ENSCAFG00845022160	0	0	0	1
ENSCAFG00845022164	143	133	86	68
ENSCAFG00845022163	647	621	625	552
ENSCAFG00845022162	0	0	0	0
ENSCAFG00845022161	434	354	399	408

ENSCAFG00845010186	5	16	12	12
ENSCAFG00845010187	0	0	0	0
ENSCAFG00845010188	4224	3879	3594	3734
ENSCAFG00845010189	0	0	0	0
ENSCAFG00845009182	0	0	0	2
ENSCAFG00845010171	320	308	273	277
ENSCAFG00845009183	5447	5105	5707	5711
ENSCAFG00845010172	0	0	0	0
ENSCAFG00845009180	25	29	16	21
ENSCAFG00845010173	0	0	0	0
ENSCAFG00845009181	0	0	0	0
ENSCAFG00845010174	0	0	0	0
ENSCAFG00845009186	717	658	702	736
ENSCAFG00845009187	2	6	1	3
ENSCAFG00845009184	61	36	75	66
ENSCAFG00845009185	0	0	21	28
ENSCAFG00845010170	9	1	1	3
ENSCAFG00845022157	0	0	0	0
ENSCAFG00845022156	19	18	12	13
ENSCAFG00845009188	0	0	1	1
ENSCAFG00845022155	17	10	19	20
ENSCAFG00845009189	389	307	425	403
ENSCAFG00845022154	0	0	0	0
ENSCAFG00845022159	0	0	0	0
ENSCAFG00845022158	0	0	0	0
ENSCAFG00845022153	1158	1120	878	920
ENSCAFG00845022152	5	5	5	3
ENSCAFG00845022151	0	0	0	0
ENSCAFG00845022150	676	681	525	505
ENSCAFG00845010179	0	0	0	0
ENSCAFG00845010175	685	612	657	676
ENSCAFG00845009190	1	1	0	0
ENSCAFG00845010176	1691	1644	1521	1621
ENSCAFG00845010177	0	0	0	0
ENSCAFG00845010178	272	193	252	267
ENSCAFG00845024097	0	0	1	1
ENSCAFG00845024098	938	906	916	870
ENSCAFG00845024099	0	0	0	0
ENSCAFG00845024093	10	6	3	6
ENSCAFG00845024094	843	798	763	866
ENSCAFG00845024095	0	0	0	1

ENSCAFG00845024096	0	0	1	2
ENSCAFG00845024090	41	54	37	37
ENSCAFG00845024091	39	47	32	42
ENSCAFG00845024092	1097	998	1056	1158
ENSCAFG00845024086	11895	11608	11201	11760
ENSCAFG00845024087	844	759	832	795
ENSCAFG00845024089	7599	7435	7387	7364
ENSCAFG00845024082	0	2	2	0
ENSCAFG00845024083	1217	1168	1241	1333
ENSCAFG00845024084	0	2	0	0
ENSCAFG00845024085	238	221	252	239
ENSCAFG00845024080	1183	1197	1243	1275
ENSCAFG00845024081	937	1012	919	987
ENSCAFG00845012097	4	2	0	0
ENSCAFG00845012096	1181	1094	1042	1071
ENSCAFG00845012099	15	11	17	12
ENSCAFG00845012098	0	0	0	0
ENSCAFG00845012093	112	125	114	111
ENSCAFG00845012092	6	2	8	5
ENSCAFG00845012095	0	0	0	0
ENSCAFG00845012094	0	1	0	0
ENSCAFG00845012091	0	0	0	0
ENSCAFG00845012090	687	739	754	715
ENSCAFG00845024079	92	97	117	125
ENSCAFG00845024075	0	0	0	0
ENSCAFG00845024076	179	153	156	150
ENSCAFG00845024077	0	0	0	0
ENSCAFG00845024078	3	4	3	2
ENSCAFG00845024071	0	0	0	0
ENSCAFG00845024072	5	4	4	4
ENSCAFG00845024073	0	0	0	0
ENSCAFG00845024074	0	1	0	7
ENSCAFG00845024070	0	0	0	0
ENSCAFG00845012086	0	0	1	0
ENSCAFG00845012085	3784	3850	3646	3751
ENSCAFG00845012088	4	2	1	0
ENSCAFG00845012087	5349	5161	4525	4460
ENSCAFG00845012082	2	7	9	7
ENSCAFG00845012081	0	0	0	0
ENSCAFG00845012084	1013	956	747	698
ENSCAFG00845012083	251	218	257	262

ENSCAFG00845012080	421	379	395	453
ENSCAFG00845024069	69	79	110	103
ENSCAFG00845000099	49	47	58	60
ENSCAFG00845000097	992	945	873	926
ENSCAFG00845024064	622	524	553	534
ENSCAFG00845000098	156	135	125	153
ENSCAFG00845024065	0	0	0	0
ENSCAFG00845000095	0	0	0	0
ENSCAFG00845024066	0	0	0	0
ENSCAFG00845000096	10	7	4	9
ENSCAFG00845024067	10	4	6	3
ENSCAFG00845000093	0	0	0	0
ENSCAFG00845024060	1	0	3	4
ENSCAFG00845000094	11	7	1	7
ENSCAFG00845024061	0	0	0	0
ENSCAFG00845000091	0	0	0	0
ENSCAFG00845024062	0	0	0	0
ENSCAFG00845000092	3952	3733	3664	3715
ENSCAFG00845024063	0	0	0	0
ENSCAFG00845000090	26	16	12	12
ENSCAFG00845012089	0	0	0	0
ENSCAFG00845012075	26	38	52	32
ENSCAFG00845012074	420	403	345	334
ENSCAFG00845012077	1	0	0	0
ENSCAFG00845012076	50	52	52	37
ENSCAFG00845012071	553	522	569	604
ENSCAFG00845012070	0	0	0	0
ENSCAFG00845012073	0	0	0	0
ENSCAFG00845012072	0	0	0	0
ENSCAFG00845024057	1	0	0	1
ENSCAFG00845024058	0	0	1	0
ENSCAFG00845024059	0	1	1	0
ENSCAFG00845024053	7479	7142	6615	6794
ENSCAFG00845024054	1194	1054	1346	1341
ENSCAFG00845024055	0	0	0	0
ENSCAFG00845024056	0	0	0	0
ENSCAFG00845024050	0	0	0	0
ENSCAFG00845024051	1	0	0	0
ENSCAFG00845024052	3	3	3	8
ENSCAFG00845012079	0	0	0	0
ENSCAFG00845012078	19	17	23	27

ENSCAFG00845002709	109	105	127	128
ENSCAFG00845002708	35	21	33	34
ENSCAFG00845002707	0	0	0	0
ENSCAFG00845002706	0	0	0	0
ENSCAFG00845002705	0	0	0	0
ENSCAFG00845002704	0	0	0	0
ENSCAFG00845002703	0	0	0	0
ENSCAFG00845002702	0	0	0	0
ENSCAFG00845002701	0	0	0	0
ENSCAFG00845002700	4479	4372	4123	4411
ENSCAFG00845014640	722	669	679	724
ENSCAFG00845026629	9	5	2	11
ENSCAFG00845026628	2	2	2	1
ENSCAFG00845002659	0	0	0	0
ENSCAFG00845002658	5	5	1	3
ENSCAFG00845026625	2251	2289	2255	2188
ENSCAFG00845002657	1	1	0	0
ENSCAFG00845026624	678	618	606	660
ENSCAFG00845002656	0	0	1	0
ENSCAFG00845026627	755	667	757	763
ENSCAFG00845002655	2081	1869	1882	1960
ENSCAFG00845026626	2747	2765	2875	2961
ENSCAFG00845002654	5	11	16	10
ENSCAFG00845026621	145	106	101	117
ENSCAFG00845002653	0	0	0	0
ENSCAFG00845026620	5225	4880	4893	4797
ENSCAFG00845002652	975	930	911	908
ENSCAFG00845026623	3	5	5	7
ENSCAFG00845002651	55	60	52	52
ENSCAFG00845026622	33	40	42	39
ENSCAFG00845002650	0	0	0	0
ENSCAFG00845014649	22	21	3	3
ENSCAFG00845014647	0	0	0	0
ENSCAFG00845014648	0	0	0	0
ENSCAFG00845014645	294	254	252	334
ENSCAFG00845014646	4717	4461	4211	4213
ENSCAFG00845014643	0	0	0	0
ENSCAFG00845014644	4	0	0	0
ENSCAFG00845014641	0	0	0	0
ENSCAFG00845014642	855	847	817	921
ENSCAFG00845026618	30	33	23	26

ENSCAFG00845026617	0	0	0	0
ENSCAFG00845000008	145	148	158	130
ENSCAFG00845000009	0	0	0	0
ENSCAFG00845026619	28	21	49	42
ENSCAFG00845000006	0	0	0	0
ENSCAFG00845002669	43	43	42	77
ENSCAFG00845026614	162	167	168	207
ENSCAFG00845000007	175	136	190	176
ENSCAFG00845002668	0	0	0	0
ENSCAFG00845026613	306	283	335	320
ENSCAFG00845000004	0	0	0	0
ENSCAFG00845002667	987	1001	929	997
ENSCAFG00845026616	192	174	95	126
ENSCAFG00845000005	0	0	0	0
ENSCAFG00845002666	404	345	367	415
ENSCAFG00845026615	1240	1216	1273	1243
ENSCAFG00845000002	1386	1338	1261	1221
ENSCAFG00845002665	0	0	0	0
ENSCAFG00845026610	1296	1280	1293	1259
ENSCAFG00845000003	32	28	28	29
ENSCAFG00845002664	478	469	464	519
ENSCAFG00845002663	1000	1059	1093	1032
ENSCAFG00845026612	0	0	0	0
ENSCAFG00845002662	40	29	31	23
ENSCAFG00845026611	235	202	175	166
ENSCAFG00845002661	0	0	0	0
ENSCAFG00845002660	930	920	854	850
ENSCAFG00845014638	502	558	586	611
ENSCAFG00845014639	0	0	0	0
ENSCAFG00845014636	1859	1725	1779	1802
ENSCAFG00845014637	2477	2423	2363	2357
ENSCAFG00845014634	202	235	177	212
ENSCAFG00845014635	5	22	9	13
ENSCAFG00845014632	2	10	19	11
ENSCAFG00845014633	340	323	383	365
ENSCAFG00845014630	1989	1888	1871	1858
ENSCAFG00845014631	2284	2265	2107	2116
ENSCAFG00845026607	1	4	5	4
ENSCAFG00845002639	92	77	103	112
ENSCAFG00845026606	0	0	0	0
ENSCAFG00845002638	0	0	0	0

ENSCAFG00845026609	0	0	0	0
ENSCAFG00845002637	1	0	1	2
ENSCAFG00845026608	0	0	0	0
ENSCAFG00845002636	0	0	0	0
ENSCAFG00845026603	0	0	0	0
ENSCAFG00845002635	0	0	0	0
ENSCAFG00845026602	2657	2371	2449	2570
ENSCAFG00845002634	2	4	0	2
ENSCAFG00845026605	394	400	355	356
ENSCAFG00845002633	0	0	0	0
ENSCAFG00845026604	3	2	1	8
ENSCAFG00845002632	19	20	6	15
ENSCAFG00845002631	0	0	0	0
ENSCAFG00845002630	0	1	1	0
ENSCAFG00845026601	1185	1054	1209	1205
ENSCAFG00845026600	3085	2927	3399	3370
ENSCAFG00845014629	1570	1452	1398	1389
ENSCAFG00845014627	728	730	610	632
ENSCAFG00845014628	1400	1317	1351	1408
ENSCAFG00845014625	0	0	3	1
ENSCAFG00845014626	814	790	753	732
ENSCAFG00845014623	0	0	0	0
ENSCAFG00845014624	129	134	115	92
ENSCAFG00845014621	217	186	151	247
ENSCAFG00845014622	26	24	42	44
ENSCAFG00845014620	0	0	0	0
ENSCAFG00845002649	1294	1290	1262	1316
ENSCAFG00845002648	0	0	0	0
ENSCAFG00845002647	3257	3151	3018	3108
ENSCAFG00845002646	691	698	610	615
ENSCAFG00845002645	0	0	0	0
ENSCAFG00845002644	1919	1780	1912	2000
ENSCAFG00845002643	0	3	2	0
ENSCAFG00845002642	0	0	0	0
ENSCAFG00845002641	226	171	160	169
ENSCAFG00845002640	34	33	41	33
ENSCAFG00845014618	57	56	63	49
ENSCAFG00845014619	9893	9538	8967	9122
ENSCAFG00845014616	3	1	8	4
ENSCAFG00845014617	13	16	6	20
ENSCAFG00845014614	0	0	1	0

ENSCAFG00845014615	3	5	1	3
ENSCAFG00845014612	639	655	717	649
ENSCAFG00845014613	1	0	0	0
ENSCAFG00845014610	1	0	0	0
ENSCAFG00845014611	2	3	0	2
ENSCAFG00845002619	0	0	0	0
ENSCAFG00845002618	0	0	2	0
ENSCAFG00845002617	3190	3086	3241	3325
ENSCAFG00845002616	0	0	0	0
ENSCAFG00845002615	0	0	0	0
ENSCAFG00845002614	0	0	0	0
ENSCAFG00845002613	0	0	0	0
ENSCAFG00845002612	8	8	6	2
ENSCAFG00845002611	0	0	0	0
ENSCAFG00845002610	0	0	0	0
ENSCAFG00845014609	91	92	98	81
ENSCAFG00845014607	40	39	26	34
ENSCAFG00845014608	45	57	68	48
ENSCAFG00845014605	0	0	0	0
ENSCAFG00845014606	0	0	0	0
ENSCAFG00845014603	0	0	0	0
ENSCAFG00845014604	238	259	193	165
ENSCAFG00845014601	0	0	1	1
ENSCAFG00845014602	2252	2066	2218	2220
ENSCAFG00845014600	2197	2256	2266	2378
ENSCAFG00845002629	1761	1790	1602	1543
ENSCAFG00845002628	868	851	995	886
ENSCAFG00845002627	0	0	1	0
ENSCAFG00845002626	0	0	0	0
ENSCAFG00845002625	206	177	179	183
ENSCAFG00845002624	0	0	0	0
ENSCAFG00845002623	0	0	0	0
ENSCAFG00845002622	0	0	0	0
ENSCAFG00845002621	0	0	0	0
ENSCAFG00845002620	0	0	0	0
ENSCAFG00845002609	1983	2020	1922	2080
ENSCAFG00845002608	6	0	2	1
ENSCAFG00845002607	0	0	0	0
ENSCAFG00845002606	0	0	0	0
ENSCAFG00845002605	63	77	52	46
ENSCAFG00845002604	1	0	0	0

ENSCAFG00845002603	0	0	0	1
ENSCAFG00845002602	386	370	387	429
ENSCAFG00845002601	2629	2559	2563	2607
ENSCAFG00845002600	0	0	0	0
ENSCAFG00845012064	47	49	66	41
ENSCAFG00845012063	0	0	0	0
ENSCAFG00845012066	2319	2248	2006	2114
ENSCAFG00845012065	44	41	40	58
ENSCAFG00845012060	4525	4364	2771	2932
ENSCAFG00845012062	0	0	0	0
ENSCAFG00845012061	0	0	0	0
ENSCAFG00845000079	0	0	0	0
ENSCAFG00845024046	0	0	0	0
ENSCAFG00845024047	0	0	0	0
ENSCAFG00845000077	0	0	0	0
ENSCAFG00845024048	535	478	487	566
ENSCAFG00845000078	51	42	31	51
ENSCAFG00845024049	0	0	0	0
ENSCAFG00845000075	0	0	0	0
ENSCAFG00845024042	1	5	6	6
ENSCAFG00845000076	0	0	0	0
ENSCAFG00845024043	0	0	0	0
ENSCAFG00845000073	1216	1165	1075	999
ENSCAFG00845000074	0	0	0	0
ENSCAFG00845024045	0	0	1	0
ENSCAFG00845000071	0	0	6	3
ENSCAFG00845000072	0	0	0	0
ENSCAFG00845024040	0	0	0	0
ENSCAFG00845000070	0	0	0	0
ENSCAFG00845024041	371	332	284	325
ENSCAFG00845012068	482	500	575	390
ENSCAFG00845012067	0	0	0	0
ENSCAFG00845012069	0	0	0	0
ENSCAFG00845012053	650	628	629	644
ENSCAFG00845012052	34	26	20	18
ENSCAFG00845012055	118	112	103	117
ENSCAFG00845012054	1	3	1	2
ENSCAFG00845012051	0	0	0	0
ENSCAFG00845012050	39	56	34	24
ENSCAFG00845024039	728	689	529	523
ENSCAFG00845024035	4	0	0	0

ENSCAFG00845026698	378	405	428	393
ENSCAFG00845024036	0	1	0	0
ENSCAFG00845026697	661	638	578	707
ENSCAFG00845000088	252	219	214	245
ENSCAFG00845024037	194	223	242	242
ENSCAFG00845000089	0	1	0	0
ENSCAFG00845024038	2081	2013	1961	1884
ENSCAFG00845026699	1	7	0	3
ENSCAFG00845000086	223	231	272	235
ENSCAFG00845024031	5950	5918	6015	5845
ENSCAFG00845026694	11366	10929	10904	11484
ENSCAFG00845000087	0	0	0	0
ENSCAFG00845024032	7281	6957	6688	7164
ENSCAFG00845026693	291	288	267	247
ENSCAFG00845000084	7	2	8	10
ENSCAFG00845024033	429	449	394	454
ENSCAFG00845026696	1	0	0	0
ENSCAFG00845000085	0	0	0	0
ENSCAFG00845024034	0	0	0	0
ENSCAFG00845026695	15	7	14	10
ENSCAFG00845000082	0	0	0	0
ENSCAFG00845026690	0	0	0	0
ENSCAFG00845000083	0	0	0	0
ENSCAFG00845000080	2	3	0	0
ENSCAFG00845026692	7	2	4	19
ENSCAFG00845000081	0	0	0	0
ENSCAFG00845024030	0	0	0	0
ENSCAFG00845026691	3	2	0	1
ENSCAFG00845012057	389	376	323	262
ENSCAFG00845012056	0	0	0	0
ENSCAFG00845012059	1	2	6	4
ENSCAFG00845012058	0	0	1	0
ENSCAFG00845012042	0	0	0	0
ENSCAFG00845012041	0	0	0	0
ENSCAFG00845012044	5	15	9	8
ENSCAFG00845012043	2	1	1	0
ENSCAFG00845012040	2196	2083	2237	2115
ENSCAFG00845024028	1	0	2	0
ENSCAFG00845024029	14	18	13	11
ENSCAFG00845000059	5	8	1	3
ENSCAFG00845000057	0	0	0	0

ENSCAFG00845024024	2	1	5	2
ENSCAFG00845026687	218	220	224	279
ENSCAFG00845000058	2	2	2	1
ENSCAFG00845024025	281	262	261	272
ENSCAFG00845026686	14	10	5	11
ENSCAFG00845000055	33	29	14	24
ENSCAFG00845024026	0	0	0	0
ENSCAFG00845026689	0	1	0	0
ENSCAFG00845000056	469	430	395	425
ENSCAFG00845026688	1262	1286	1167	1133
ENSCAFG00845000053	1	1	3	3
ENSCAFG00845024020	0	0	0	0
ENSCAFG00845026683	5	6	6	2
ENSCAFG00845000054	166	188	195	143
ENSCAFG00845024021	3	0	0	2
ENSCAFG00845026682	2	1	3	2
ENSCAFG00845000051	1	1	0	1
ENSCAFG00845024022	217	177	165	137
ENSCAFG00845000052	0	0	0	0
ENSCAFG00845024023	125	137	84	76
ENSCAFG00845026684	603	640	594	578
ENSCAFG00845000050	0	3	5	0
ENSCAFG00845026680	102	114	115	108
ENSCAFG00845012049	0	0	3	0
ENSCAFG00845012046	0	0	0	0
ENSCAFG00845012045	1395	1288	1385	1377
ENSCAFG00845012048	15	10	4	13
ENSCAFG00845012047	1519	1378	1302	1390
ENSCAFG00845012031	0	0	0	0
ENSCAFG00845014694	0	0	0	0
ENSCAFG00845012030	2	2	0	1
ENSCAFG00845014695	209	201	222	187
ENSCAFG00845012033	5	2	5	4
ENSCAFG00845014692	1	9	6	3
ENSCAFG00845012032	0	0	0	0
ENSCAFG00845014693	35	29	46	41
ENSCAFG00845014690	2	2	3	0
ENSCAFG00845014691	4	0	0	0
ENSCAFG00845024017	1118	1019	1105	1085
ENSCAFG00845024018	0	0	0	0
ENSCAFG00845026679	0	0	1	1

ENSCAFG00845024019	8404	8255	7537	8255
ENSCAFG00845000068	372	373	388	445
ENSCAFG00845024013	0	0	1	2
ENSCAFG00845026676	0	0	0	0
ENSCAFG00845000069	6	3	10	4
ENSCAFG00845024014	171	131	129	106
ENSCAFG00845026675	1500	1450	1475	1518
ENSCAFG00845000066	435	426	418	426
ENSCAFG00845024015	1092	1097	944	948
ENSCAFG00845026678	0	0	0	0
ENSCAFG00845000067	0	0	0	0
ENSCAFG00845024016	594	606	555	541
ENSCAFG00845026677	1068	994	1040	1112
ENSCAFG00845000064	1529	1426	1320	1436
ENSCAFG00845026672	114	129	87	108
ENSCAFG00845000065	0	0	0	0
ENSCAFG00845024010	147	174	132	183
ENSCAFG00845026671	0	0	0	0
ENSCAFG00845000062	12	11	10	1
ENSCAFG00845024011	0	0	2	3
ENSCAFG00845026674	619	508	592	629
ENSCAFG00845000063	0	0	0	1
ENSCAFG00845024012	0	0	0	0
ENSCAFG00845026673	126	145	142	143
ENSCAFG00845000060	0	0	0	0
ENSCAFG00845000061	0	0	0	0
ENSCAFG00845026670	808	793	892	828
ENSCAFG00845012039	12	16	15	13
ENSCAFG00845012038	2449	2166	2426	2706
ENSCAFG00845012035	188	216	208	185
ENSCAFG00845014698	4	0	4	1
ENSCAFG00845012034	0	0	0	0
ENSCAFG00845014699	1104	1037	1022	997
ENSCAFG00845012037	0	0	0	0
ENSCAFG00845014696	2300	2193	2304	2328
ENSCAFG00845012036	2246	2204	1099	1142
ENSCAFG00845014697	5	9	7	13
ENSCAFG00845012020	3	1	1	2
ENSCAFG00845014683	0	0	0	0
ENSCAFG00845014684	2	0	3	1
ENSCAFG00845012022	0	0	0	0

ENSCAFG00845014681	0	1	1	0
ENSCAFG00845012021	3215	3341	3596	3797
ENSCAFG00845014682	5	0	1	5
ENSCAFG00845014680	1367	1339	1486	1465
ENSCAFG00845000039	0	0	0	0
ENSCAFG00845024006	439	415	467	476
ENSCAFG00845026669	31	20	34	20
ENSCAFG00845024007	1	0	0	2
ENSCAFG00845000037	1	0	0	0
ENSCAFG00845024008	1066	1045	1058	961
ENSCAFG00845000038	0	0	0	0
ENSCAFG00845002699	481	496	538	519
ENSCAFG00845024009	372	437	369	373
ENSCAFG00845000035	0	0	0	0
ENSCAFG00845002698	0	0	0	0
ENSCAFG00845024002	871	892	782	811
ENSCAFG00845026665	3	1	2	3
ENSCAFG00845000036	699	685	751	710
ENSCAFG00845002697	43	40	52	52
ENSCAFG00845024003	911	878	892	949
ENSCAFG00845026664	0	0	0	3
ENSCAFG00845000033	881	847	825	771
ENSCAFG00845002696	4698	4578	4148	4293
ENSCAFG00845024004	1373	1284	1551	1549
ENSCAFG00845026667	1067	908	942	860
ENSCAFG00845000034	0	0	0	0
ENSCAFG00845002695	1791	1825	1802	1748
ENSCAFG00845024005	495	484	525	466
ENSCAFG00845026666	0	0	0	0
ENSCAFG00845000031	0	0	0	0
ENSCAFG00845002694	4	2	4	2
ENSCAFG00845026661	0	0	0	0
ENSCAFG00845000032	55	37	36	37
ENSCAFG00845002693	97	85	91	80
ENSCAFG00845026660	0	0	0	0
ENSCAFG00845002692	199	154	226	197
ENSCAFG00845024000	1	0	0	1
ENSCAFG00845026663	0	1	1	0
ENSCAFG00845000030	57	50	41	37
ENSCAFG00845002691	182	191	165	195
ENSCAFG00845024001	1	0	0	0

ENSCAFG00845026662	0	0	0	0
ENSCAFG00845002690	71	85	78	76
ENSCAFG00845012028	0	0	0	0
ENSCAFG00845012027	0	0	0	0
ENSCAFG00845014689	1	1	2	0
ENSCAFG00845012029	0	0	0	0
ENSCAFG00845012024	3	6	7	2
ENSCAFG00845014687	6	10	6	2
ENSCAFG00845012023	0	0	0	0
ENSCAFG00845014688	196	199	285	264
ENSCAFG00845012026	0	0	0	0
ENSCAFG00845014685	0	0	0	0
ENSCAFG00845012025	0	0	0	0
ENSCAFG00845014686	157	190	171	170
ENSCAFG00845014672	2	1	0	4
ENSCAFG00845014673	1	0	0	0
ENSCAFG00845012011	67	46	46	65
ENSCAFG00845014670	21	20	13	29
ENSCAFG00845012010	0	0	0	0
ENSCAFG00845014671	1089	1073	953	1020
ENSCAFG00845026658	356	357	325	335
ENSCAFG00845026657	25	29	44	55
ENSCAFG00845000048	21	19	16	23
ENSCAFG00845000049	0	2	0	0
ENSCAFG00845026659	29	36	35	40
ENSCAFG00845000046	0	0	0	0
ENSCAFG00845026654	132	144	112	103
ENSCAFG00845000047	18	15	8	11
ENSCAFG00845026653	2434	2438	2192	2178
ENSCAFG00845000044	429	370	360	354
ENSCAFG00845026656	0	0	0	0
ENSCAFG00845000045	1047	985	890	899
ENSCAFG00845026655	981	994	954	1034
ENSCAFG00845000042	6	4	2	2
ENSCAFG00845026650	127	142	101	112
ENSCAFG00845000043	2076	1913	1999	2105
ENSCAFG00845000040	5264	5145	5817	6071
ENSCAFG00845026652	0	0	0	0
ENSCAFG00845000041	0	0	0	0
ENSCAFG00845026651	1009	1067	1159	1176
ENSCAFG00845012017	249	246	197	205

ENSCAFG00845012016	1788	1628	1737	1705
ENSCAFG00845012019	0	0	0	0
ENSCAFG00845014678	1355	1318	1195	1293
ENSCAFG00845012018	0	0	0	0
ENSCAFG00845014679	0	0	0	0
ENSCAFG00845012013	70	41	83	74
ENSCAFG00845014676	314	324	317	336
ENSCAFG00845012012	8	3	11	20
ENSCAFG00845014677	810	727	789	793
ENSCAFG00845012015	3873	3674	3598	3738
ENSCAFG00845014674	2	5	4	6
ENSCAFG00845012014	0	0	0	0
ENSCAFG00845014675	7	3	7	2
ENSCAFG00845014661	465	366	321	368
ENSCAFG00845014662	0	0	0	1
ENSCAFG00845012000	0	0	0	0
ENSCAFG00845014660	1	1	0	0
ENSCAFG00845000019	173	187	169	129
ENSCAFG00845000017	0	0	0	0
ENSCAFG00845026647	0	0	0	0
ENSCAFG00845000018	1	0	0	1
ENSCAFG00845002679	3	1	1	0
ENSCAFG00845026646	0	0	0	0
ENSCAFG00845000015	0	0	0	0
ENSCAFG00845002678	74	63	37	33
ENSCAFG00845026649	0	0	0	0
ENSCAFG00845000016	653	517	556	524
ENSCAFG00845002677	0	1	2	2
ENSCAFG00845026648	384	323	356	366
ENSCAFG00845000013	0	0	0	0
ENSCAFG00845002676	13	9	6	6
ENSCAFG00845026643	147	147	47	56
ENSCAFG00845000014	389	342	344	362
ENSCAFG00845002675	70	66	70	39
ENSCAFG00845026642	76	56	71	71
ENSCAFG00845000011	0	0	0	0
ENSCAFG00845002674	229	254	256	264
ENSCAFG00845026645	0	5	2	1
ENSCAFG00845000012	0	0	0	0
ENSCAFG00845002673	1514	1511	1555	1436
ENSCAFG00845026644	19	19	10	5

ENSCAFG00845002672	0	3	3	4
ENSCAFG00845000010	0	0	0	0
ENSCAFG00845002671	0	1	0	2
ENSCAFG00845002670	0	0	0	0
ENSCAFG00845026641	424	371	395	383
ENSCAFG00845026640	298	220	281	332
ENSCAFG00845012009	31	23	33	39
ENSCAFG00845012006	0	0	0	0
ENSCAFG00845014669	21	11	17	29
ENSCAFG00845012005	0	0	0	0
ENSCAFG00845012008	0	0	0	0
ENSCAFG00845014667	1	3	2	3
ENSCAFG00845012007	410	402	478	421
ENSCAFG00845014668	0	0	0	1
ENSCAFG00845012002	3	0	0	0
ENSCAFG00845014665	0	0	0	1
ENSCAFG00845012001	804	793	639	634
ENSCAFG00845014666	666	608	620	616
ENSCAFG00845012004	0	0	0	0
ENSCAFG00845014663	0	1	0	1
ENSCAFG00845012003	2	4	0	4
ENSCAFG00845014664	0	0	0	0
ENSCAFG00845014650	0	1	3	0
ENSCAFG00845014651	637	598	543	618
ENSCAFG00845026639	1305	1342	1287	1363
ENSCAFG00845000028	2	1	2	1
ENSCAFG00845026636	0	0	0	0
ENSCAFG00845000029	0	0	0	0
ENSCAFG00845026635	0	2	0	0
ENSCAFG00845000026	716	671	706	712
ENSCAFG00845002689	253	234	230	239
ENSCAFG00845026638	2587	2412	2250	2374
ENSCAFG00845000027	19	15	19	22
ENSCAFG00845002688	33	47	46	45
ENSCAFG00845026637	0	0	0	0
ENSCAFG00845000024	634	574	680	627
ENSCAFG00845002687	0	0	0	0
ENSCAFG00845026632	6702	6563	6432	6743
ENSCAFG00845000025	0	0	0	0
ENSCAFG00845002686	3608	3445	3539	3730
ENSCAFG00845026631	214	247	243	212

ENSCAFG00845000022	0	0	0	0
ENSCAFG00845002685	295	278	243	270
ENSCAFG00845026634	190	191	224	215
ENSCAFG00845000023	2	2	1	1
ENSCAFG00845002684	0	0	0	0
ENSCAFG00845026633	1143	963	978	956
ENSCAFG00845000020	3	2	0	2
ENSCAFG00845002683	0	0	0	0
ENSCAFG00845000021	798	674	683	769
ENSCAFG00845002682	0	0	0	0
ENSCAFG00845002681	0	6	4	4
ENSCAFG00845026630	1636	1526	1635	1623
ENSCAFG00845002680	5	5	6	6
ENSCAFG00845014658	88	75	74	78
ENSCAFG00845014659	4	1	3	3
ENSCAFG00845014656	0	0	0	0
ENSCAFG00845014657	49	44	49	64
ENSCAFG00845014654	0	0	0	1
ENSCAFG00845014655	409	489	420	370
ENSCAFG00845014652	0	0	0	0
ENSCAFG00845014653	96	85	83	73
ENSCAFG00845024196	646	600	596	579
ENSCAFG00845024197	18	14	22	21
ENSCAFG00845024198	0	0	0	0
ENSCAFG00845024199	1	2	3	4
ENSCAFG00845024192	5135	5301	4858	4864
ENSCAFG00845024193	0	0	0	0
ENSCAFG00845024195	2053	2072	2122	2020
ENSCAFG00845024190	0	0	0	0
ENSCAFG00845024191	0	0	3	0
ENSCAFG00845024189	51	54	71	63
ENSCAFG00845024185	85	53	59	57
ENSCAFG00845024186	6	0	4	5
ENSCAFG00845024187	0	0	0	0
ENSCAFG00845024188	0	0	1	0
ENSCAFG00845024181	30	29	36	19
ENSCAFG00845024182	1115	971	1025	1081
ENSCAFG00845024183	1	0	1	1
ENSCAFG00845024184	0	0	0	0
ENSCAFG00845024180	0	0	0	0
ENSCAFG00845012196	4092	3893	3958	4127

ENSCAFG00845012195	851	851	920	884
ENSCAFG00845012198	269	256	219	241
ENSCAFG00845012197	764	653	657	623
ENSCAFG00845012192	0	0	0	0
ENSCAFG00845012191	1115	1097	889	961
ENSCAFG00845012194	0	0	0	0
ENSCAFG00845012193	124	132	100	105
ENSCAFG00845012190	0	0	0	0
ENSCAFG00845024178	1140	1157	1087	1121
ENSCAFG00845024179	0	0	0	0
ENSCAFG00845024174	0	0	0	0
ENSCAFG00845024175	0	0	2	1
ENSCAFG00845024176	1	2	0	2
ENSCAFG00845024177	441	473	591	546
ENSCAFG00845024170	162	149	151	171
ENSCAFG00845024171	12	16	19	18
ENSCAFG00845024172	0	0	1	0
ENSCAFG00845024173	47	27	22	21
ENSCAFG00845012199	12	11	29	26
ENSCAFG00845002819	117	95	102	106
ENSCAFG00845002818	77	73	83	81
ENSCAFG00845002817	263	259	285	278
ENSCAFG00845002816	0	0	0	3
ENSCAFG00845002815	70	101	99	94
ENSCAFG00845002814	305	287	279	232
ENSCAFG00845002813	1602	1480	1564	1665
ENSCAFG00845002812	896	865	852	810
ENSCAFG00845002811	7	2	3	4
ENSCAFG00845002810	1469	1454	1289	1235
ENSCAFG00845014809	0	0	0	0
ENSCAFG00845014807	10	12	9	18
ENSCAFG00845014808	0	0	0	0
ENSCAFG00845014805	0	0	0	0
ENSCAFG00845014806	10	19	26	24
ENSCAFG00845014803	0	0	0	0
ENSCAFG00845014804	546	448	426	418
ENSCAFG00845014801	0	0	1	1
ENSCAFG00845014802	0	0	0	0
ENSCAFG00845014800	2115	2154	2033	1952
ENSCAFG00845002829	41	38	35	65
ENSCAFG00845002828	0	0	0	0

ENSCAFG00845002827	1762	1607	1715	1689
ENSCAFG00845002826	0	0	0	0
ENSCAFG00845002825	272	229	234	271
ENSCAFG00845002824	0	0	0	0
ENSCAFG00845002823	191	182	178	182
ENSCAFG00845002822	0	0	0	0
ENSCAFG00845002821	5	3	5	5
ENSCAFG00845002820	2	6	1	6
ENSCAFG00845002809	0	0	0	0
ENSCAFG00845002808	0	0	0	0
ENSCAFG00845002807	0	0	0	0
ENSCAFG00845002806	0	1	0	2
ENSCAFG00845002805	9	8	11	14
ENSCAFG00845002804	0	0	0	0
ENSCAFG00845002803	512	438	463	474
ENSCAFG00845002802	0	0	0	0
ENSCAFG00845002801	0	3	0	4
ENSCAFG00845002800	315	307	286	265
ENSCAFG00845014760	4078	3996	4649	4405
ENSCAFG00845014761	0	0	0	0
ENSCAFG00845026749	529	524	438	402
ENSCAFG00845000118	1	5	1	3
ENSCAFG00845000119	3	0	2	7
ENSCAFG00845000116	4	1	3	2
ENSCAFG00845002779	2	2	0	0
ENSCAFG00845026746	0	0	1	0
ENSCAFG00845000117	1	1	0	0
ENSCAFG00845002778	5	11	5	4
ENSCAFG00845026745	2	0	0	0
ENSCAFG00845000114	0	2	0	0
ENSCAFG00845002777	46	80	61	47
ENSCAFG00845026748	2	0	0	1
ENSCAFG00845000115	0	1	0	0
ENSCAFG00845002776	0	0	0	0
ENSCAFG00845026747	0	0	0	0
ENSCAFG00845000112	0	0	0	0
ENSCAFG00845002775	0	0	0	0
ENSCAFG00845026742	0	0	0	0
ENSCAFG00845000113	158	132	87	149
ENSCAFG00845002774	0	0	0	0
ENSCAFG00845026741	0	0	0	0

ENSCAFG00845000110	0	0	0	0
ENSCAFG00845002773	78	80	80	90
ENSCAFG00845026744	603	586	682	603
ENSCAFG00845000111	462	406	324	296
ENSCAFG00845002772	0	0	0	0
ENSCAFG00845026743	0	0	0	0
ENSCAFG00845002771	299	287	288	206
ENSCAFG00845002770	0	0	0	0
ENSCAFG00845026740	731	686	714	712
ENSCAFG00845012109	508	513	470	565
ENSCAFG00845012108	7	7	7	5
ENSCAFG00845012105	920	834	835	797
ENSCAFG00845014768	1545	1554	1528	1603
ENSCAFG00845012104	1103	919	1043	1026
ENSCAFG00845014769	0	0	0	0
ENSCAFG00845012107	6	1	1	0
ENSCAFG00845014766	0	0	0	0
ENSCAFG00845012106	1341	1327	1435	1489
ENSCAFG00845014767	63	66	92	88
ENSCAFG00845012101	92	104	105	119
ENSCAFG00845014764	0	1	0	0
ENSCAFG00845012100	0	0	0	0
ENSCAFG00845014765	0	0	0	0
ENSCAFG00845012103	105	101	76	66
ENSCAFG00845014762	345	264	326	301
ENSCAFG00845012102	0	0	0	0
ENSCAFG00845014763	45	45	34	37
ENSCAFG00845014750	1941	1726	2151	2059
ENSCAFG00845026739	2	4	1	1
ENSCAFG00845000129	0	0	0	0
ENSCAFG00845000127	0	2	0	1
ENSCAFG00845026735	0	0	0	0
ENSCAFG00845000128	835	847	781	764
ENSCAFG00845002789	0	0	0	0
ENSCAFG00845026734	0	0	0	0
ENSCAFG00845000125	0	0	0	0
ENSCAFG00845002788	0	0	0	0
ENSCAFG00845026737	1	0	0	1
ENSCAFG00845000126	46	43	57	77
ENSCAFG00845002787	2583	2654	2702	2700
ENSCAFG00845026736	0	0	0	1

ENSCAFG00845000123	331	370	378	344
ENSCAFG00845002786	2	1	0	0
ENSCAFG00845026731	0	0	2	0
ENSCAFG00845000124	0	2	0	0
ENSCAFG00845002785	0	0	0	0
ENSCAFG00845026730	0	0	0	0
ENSCAFG00845000121	0	0	0	0
ENSCAFG00845002784	90	103	101	52
ENSCAFG00845026733	0	8	0	3
ENSCAFG00845000122	1	1	2	0
ENSCAFG00845002783	3679	3444	3828	3778
ENSCAFG00845026732	0	2	5	0
ENSCAFG00845002782	0	0	0	0
ENSCAFG00845000120	123	121	106	131
ENSCAFG00845002781	561	510	510	568
ENSCAFG00845002780	0	0	1	0
ENSCAFG00845014759	0	0	0	0
ENSCAFG00845014757	1394	1274	1831	1944
ENSCAFG00845014758	0	0	0	0
ENSCAFG00845014755	1005	862	808	788
ENSCAFG00845014756	0	0	0	0
ENSCAFG00845014753	2425	2232	2286	2312
ENSCAFG00845014754	1994	1947	1817	1841
ENSCAFG00845014751	0	0	0	0
ENSCAFG00845014752	122	108	124	101
ENSCAFG00845026728	3107	3020	3192	3474
ENSCAFG00845026727	0	0	0	0
ENSCAFG00845002759	331	341	280	314
ENSCAFG00845002758	0	0	0	0
ENSCAFG00845002757	612	584	516	476
ENSCAFG00845026724	1	2	1	4
ENSCAFG00845002756	135	136	125	123
ENSCAFG00845026723	0	0	0	0
ENSCAFG00845002755	0	0	0	0
ENSCAFG00845026726	0	0	0	0
ENSCAFG00845002754	216	214	204	159
ENSCAFG00845026725	0	0	0	0
ENSCAFG00845002753	62	46	75	52
ENSCAFG00845026720	1487	1339	1609	1637
ENSCAFG00845002752	0	0	0	0
ENSCAFG00845002751	0	0	0	0

ENSCAFG00845026722	0	0	0	1
ENSCAFG00845002750	60	48	31	28
ENSCAFG00845026721	34	35	49	33
ENSCAFG00845014748	0	0	0	0
ENSCAFG00845014749	1	1	1	1
ENSCAFG00845014746	0	0	0	0
ENSCAFG00845014747	200	221	180	236
ENSCAFG00845014744	5	1	4	6
ENSCAFG00845014745	1180	1096	1186	1256
ENSCAFG00845014742	0	0	0	0
ENSCAFG00845014743	12	5	15	4
ENSCAFG00845014740	5	5	2	3
ENSCAFG00845014741	0	0	0	0
ENSCAFG00845000109	0	0	0	0
ENSCAFG00845026717	1591	1472	1416	1471
ENSCAFG00845000107	0	0	0	0
ENSCAFG00845026719	460	427	471	479
ENSCAFG00845000108	1	3	3	5
ENSCAFG00845002769	0	0	0	0
ENSCAFG00845026718	2	5	0	2
ENSCAFG00845000105	2	3	0	0
ENSCAFG00845002768	1045	1016	1052	1034
ENSCAFG00845026713	57	78	55	65
ENSCAFG00845000106	0	0	0	0
ENSCAFG00845002767	0	0	0	0
ENSCAFG00845026712	556	548	548	585
ENSCAFG00845000103	278	324	223	250
ENSCAFG00845002766	1	0	0	1
ENSCAFG00845026715	1268	1317	1307	1318
ENSCAFG00845000104	678	639	694	733
ENSCAFG00845002765	0	0	0	0
ENSCAFG00845026714	1	0	1	2
ENSCAFG00845000101	0	0	0	0
ENSCAFG00845002764	0	0	0	0
ENSCAFG00845000102	0	0	0	0
ENSCAFG00845002763	162	166	118	131
ENSCAFG00845002762	4254	4081	3987	4323
ENSCAFG00845026711	643	619	570	635
ENSCAFG00845000100	0	0	0	0
ENSCAFG00845002761	2	0	0	0
ENSCAFG00845026710	16	8	17	19

ENSCAFG00845002760	115	124	138	163
ENSCAFG00845014739	1035	1105	1081	1185
ENSCAFG00845014737	280	241	192	233
ENSCAFG00845014738	1120	1023	1076	956
ENSCAFG00845014735	2449	2405	2377	2354
ENSCAFG00845014736	0	0	0	0
ENSCAFG00845014733	258	239	262	223
ENSCAFG00845014734	0	0	0	0
ENSCAFG00845014731	0	1	3	0
ENSCAFG00845014732	1136	1185	1065	1111
ENSCAFG00845014730	0	0	0	0
ENSCAFG00845026709	255	173	217	270
ENSCAFG00845002739	7	3	2	4
ENSCAFG00845026706	0	0	0	0
ENSCAFG00845002738	0	0	0	0
ENSCAFG00845026705	6	0	1	1
ENSCAFG00845002737	1	0	0	0
ENSCAFG00845026708	27	20	39	30
ENSCAFG00845002736	0	2	1	4
ENSCAFG00845026707	377	298	363	402
ENSCAFG00845002735	0	0	0	0
ENSCAFG00845026702	9	6	8	9
ENSCAFG00845002734	114	96	107	99
ENSCAFG00845026701	0	0	0	1
ENSCAFG00845002733	4	4	8	6
ENSCAFG00845026704	177	192	194	185
ENSCAFG00845002732	5	3	2	1
ENSCAFG00845026703	3	0	4	2
ENSCAFG00845002731	570	538	642	600
ENSCAFG00845002730	1	1	0	0
ENSCAFG00845026700	1029	931	846	890
ENSCAFG00845014728	0	0	0	0
ENSCAFG00845014729	0	0	0	0
ENSCAFG00845014726	3	2	2	1
ENSCAFG00845014727	181	199	213	195
ENSCAFG00845014724	117	130	123	101
ENSCAFG00845014725	2182	2069	2031	2148
ENSCAFG00845014722	1928	1836	1641	1738
ENSCAFG00845014723	0	0	0	0
ENSCAFG00845014720	0	1	0	2
ENSCAFG00845014721	18	20	17	18

ENSCAFG00845002749	0	0	0	0
ENSCAFG00845002748	0	0	0	0
ENSCAFG00845002747	7745	7385	7447	7570
ENSCAFG00845002746	3339	3216	3291	3058
ENSCAFG00845002745	69	69	98	102
ENSCAFG00845002744	0	0	0	3
ENSCAFG00845002743	0	0	0	0
ENSCAFG00845002742	106	96	95	114
ENSCAFG00845002741	8	5	2	3
ENSCAFG00845002740	1	0	0	0
ENSCAFG00845014719	5269	5125	4691	4772
ENSCAFG00845014717	39	36	30	42
ENSCAFG00845014718	3	4	0	8
ENSCAFG00845014715	0	0	0	1
ENSCAFG00845014716	0	0	0	0
ENSCAFG00845014713	0	1	0	0
ENSCAFG00845014714	0	0	0	0
ENSCAFG00845014711	2255	2213	2025	2055
ENSCAFG00845014712	111	93	208	216
ENSCAFG00845014710	432	450	350	346
ENSCAFG00845002719	0	0	0	0
ENSCAFG00845002718	87	110	84	83
ENSCAFG00845002717	0	0	0	0
ENSCAFG00845002716	0	0	0	0
ENSCAFG00845002715	0	0	0	0
ENSCAFG00845002714	17	17	8	20
ENSCAFG00845002713	1	0	0	1
ENSCAFG00845002712	150	156	133	133
ENSCAFG00845002711	0	0	0	0
ENSCAFG00845002710	341	389	365	332
ENSCAFG00845014708	3820	3968	3721	3878
ENSCAFG00845014709	0	0	1	0
ENSCAFG00845014706	1	3	0	0
ENSCAFG00845014707	776	727	711	766
ENSCAFG00845014704	3	8	1	6
ENSCAFG00845014705	722	757	827	761
ENSCAFG00845014702	195	215	235	263
ENSCAFG00845014703	670	617	733	745
ENSCAFG00845014700	0	0	0	0
ENSCAFG00845014701	0	0	0	0
ENSCAFG00845002729	0	0	0	0

ENSCAFG00845002728	0	0	0	0
ENSCAFG00845002727	0	0	0	0
ENSCAFG00845002726	2040	2094	2081	2036
ENSCAFG00845002725	46	59	40	47
ENSCAFG00845002724	3065	2927	2918	2915
ENSCAFG00845002723	1	2	0	1
ENSCAFG00845002722	5871	5432	5213	5672
ENSCAFG00845002721	7	0	6	3
ENSCAFG00845002720	261	268	269	275
ENSCAFG00845012185	1	1	0	0
ENSCAFG00845012184	1407	1320	1173	1219
ENSCAFG00845012187	0	0	0	0
ENSCAFG00845012186	7	1	3	1
ENSCAFG00845012181	0	0	0	0
ENSCAFG00845012180	332	323	324	348
ENSCAFG00845012183	1	1	1	4
ENSCAFG00845012182	0	0	0	1
ENSCAFG00845024167	2	0	0	2
ENSCAFG00845024168	512	466	422	479
ENSCAFG00845000198	0	1	0	0
ENSCAFG00845024169	0	0	0	0
ENSCAFG00845000199	0	0	0	0
ENSCAFG00845000196	0	0	0	0
ENSCAFG00845024163	19	16	17	30
ENSCAFG00845000197	0	0	0	2
ENSCAFG00845024164	1	0	0	0
ENSCAFG00845000194	557	607	613	596
ENSCAFG00845024165	0	0	0	0
ENSCAFG00845000195	317	276	318	274
ENSCAFG00845024166	16	7	15	12
ENSCAFG00845000192	0	0	0	0
ENSCAFG00845000193	3022	2823	2798	2865
ENSCAFG00845024160	0	0	0	2
ENSCAFG00845000190	25	39	30	28
ENSCAFG00845024161	349	379	343	403
ENSCAFG00845000191	0	0	0	0
ENSCAFG00845024162	0	0	0	0
ENSCAFG00845012189	0	0	0	0
ENSCAFG00845012188	0	4	0	0
ENSCAFG00845012174	147	146	187	162
ENSCAFG00845012173	406	402	399	389

ENSCAFG00845012176	0	0	0	0
ENSCAFG00845012175	759	784	788	799
ENSCAFG00845012170	0	0	0	0
ENSCAFG00845012172	5675	5383	4741	4923
ENSCAFG00845012171	7	5	1	1
ENSCAFG00845024156	49	61	39	41
ENSCAFG00845024157	2583	2458	2358	2404
ENSCAFG00845024158	1070	1067	1065	998
ENSCAFG00845024159	170	172	210	164
ENSCAFG00845024152	0	0	0	0
ENSCAFG00845024153	0	1	0	0
ENSCAFG00845024154	349	334	387	397
ENSCAFG00845024155	0	0	0	0
ENSCAFG00845024150	531	522	534	536
ENSCAFG00845012178	731	748	685	735
ENSCAFG00845012177	0	0	1	0
ENSCAFG00845012179	7	11	13	18
ENSCAFG00845012163	449	475	409	405
ENSCAFG00845012162	2	6	3	5
ENSCAFG00845012165	111	121	100	113
ENSCAFG00845012164	2414	2376	2494	2464
ENSCAFG00845012161	0	1	0	4
ENSCAFG00845012160	1173	1135	1126	1106
ENSCAFG00845024149	2202	2175	2214	2423
ENSCAFG00845000178	136	80	90	110
ENSCAFG00845024145	0	1	0	1
ENSCAFG00845000179	0	0	0	0
ENSCAFG00845024146	1667	1580	1582	1620
ENSCAFG00845000176	0	0	0	0
ENSCAFG00845024147	151	145	148	152
ENSCAFG00845000177	1761	1749	1697	1729
ENSCAFG00845024148	0	0	0	0
ENSCAFG00845000174	0	0	0	0
ENSCAFG00845024141	1	2	1	2
ENSCAFG00845000175	116	118	137	128
ENSCAFG00845024142	0	0	0	0
ENSCAFG00845000172	0	0	0	0
ENSCAFG00845024143	2	6	3	6
ENSCAFG00845000173	0	2	2	1
ENSCAFG00845024144	753	630	602	578
ENSCAFG00845000170	0	0	0	0

ENSCAFG00845000171	3	0	2	0
ENSCAFG00845024140	1	0	1	2
ENSCAFG00845012167	0	0	2	0
ENSCAFG00845012166	1064	987	951	962
ENSCAFG00845012169	0	2	0	0
ENSCAFG00845012168	0	0	0	0
ENSCAFG00845012152	319	325	243	264
ENSCAFG00845012151	2	0	0	0
ENSCAFG00845012154	7	1	5	7
ENSCAFG00845012153	0	0	0	0
ENSCAFG00845012150	2582	2381	2531	2983
ENSCAFG00845024138	0	0	0	0
ENSCAFG00845024139	36	24	18	38
ENSCAFG00845000189	24	7	20	28
ENSCAFG00845024134	180	199	182	170
ENSCAFG00845026797	0	0	0	0
ENSCAFG00845024135	408	402	367	374
ENSCAFG00845026796	641	602	546	495
ENSCAFG00845000187	353	280	319	315
ENSCAFG00845024136	138	115	131	128
ENSCAFG00845026799	2349	2170	2319	2264
ENSCAFG00845000188	4	2	2	2
ENSCAFG00845024137	339	332	374	343
ENSCAFG00845026798	1	0	0	1
ENSCAFG00845000185	0	0	0	0
ENSCAFG00845024130	339	305	326	296
ENSCAFG00845026793	0	0	0	0
ENSCAFG00845000186	0	0	0	0
ENSCAFG00845024131	11	8	10	10
ENSCAFG00845026792	12	12	19	8
ENSCAFG00845000183	0	0	0	0
ENSCAFG00845024132	0	0	0	0
ENSCAFG00845026795	5803	5801	5737	5889
ENSCAFG00845000184	0	0	0	0
ENSCAFG00845024133	0	0	0	0
ENSCAFG00845026794	0	0	0	0
ENSCAFG00845000181	135	97	119	127
ENSCAFG00845000182	0	0	0	0
ENSCAFG00845026791	0	0	2	0
ENSCAFG00845000180	251	219	201	207
ENSCAFG00845026790	5417	5086	4890	4890

ENSCAFG00845012159	557	551	508	568
ENSCAFG00845012156	1	0	2	0
ENSCAFG00845012155	4	7	0	8
ENSCAFG00845012158	6	8	4	4
ENSCAFG00845012157	3	0	2	2
ENSCAFG00845012141	7	8	4	5
ENSCAFG00845012140	248	213	200	206
ENSCAFG00845012143	0	0	0	1
ENSCAFG00845012142	467	458	451	464
ENSCAFG00845024127	0	0	0	0
ENSCAFG00845024128	0	0	0	0
ENSCAFG00845026789	0	0	0	0
ENSCAFG00845000158	313	291	236	255
ENSCAFG00845024129	0	0	0	0
ENSCAFG00845000159	0	0	0	0
ENSCAFG00845000156	0	0	0	0
ENSCAFG00845000157	63	41	58	50
ENSCAFG00845024124	2440	2376	2251	2279
ENSCAFG00845026785	0	0	0	0
ENSCAFG00845000154	1058	903	793	881
ENSCAFG00845024125	0	0	0	0
ENSCAFG00845026788	3	0	0	0
ENSCAFG00845000155	16	9	10	4
ENSCAFG00845024126	0	0	0	0
ENSCAFG00845026787	0	0	0	0
ENSCAFG00845000152	268	257	246	247
ENSCAFG00845026782	1	1	0	0
ENSCAFG00845000153	0	0	0	0
ENSCAFG00845024120	0	0	0	0
ENSCAFG00845026781	54	64	33	30
ENSCAFG00845000150	4704	4563	4403	4344
ENSCAFG00845024121	339	299	328	314
ENSCAFG00845026784	0	0	0	0
ENSCAFG00845000151	0	0	0	0
ENSCAFG00845024122	6	11	4	6
ENSCAFG00845026783	837	726	649	703
ENSCAFG00845026780	0	0	0	0
ENSCAFG00845012149	0	0	0	0
ENSCAFG00845012148	0	0	0	0
ENSCAFG00845012145	0	0	0	0
ENSCAFG00845012144	0	0	0	0

ENSCAFG00845012147	239	227	251	265
ENSCAFG00845012146	1456	1560	1446	1335
ENSCAFG00845012130	0	0	0	0
ENSCAFG00845014793	0	0	0	0
ENSCAFG00845014794	0	0	0	0
ENSCAFG00845012132	15	3	4	5
ENSCAFG00845014791	10	0	6	5
ENSCAFG00845012131	0	0	0	0
ENSCAFG00845014792	0	1	1	0
ENSCAFG00845014790	0	0	0	0
ENSCAFG00845024116	1287	1337	1083	1109
ENSCAFG00845026779	271	306	309	311
ENSCAFG00845024117	13	0	8	12
ENSCAFG00845026778	1217	1224	1186	1289
ENSCAFG00845000169	0	0	0	0
ENSCAFG00845024118	371	373	419	511
ENSCAFG00845024119	0	0	0	0
ENSCAFG00845000167	91	80	69	75
ENSCAFG00845024112	1297	1125	1345	1352
ENSCAFG00845026775	76	97	107	74
ENSCAFG00845000168	0	1	2	0
ENSCAFG00845024113	3741	3599	3426	3292
ENSCAFG00845026774	2	2	1	0
ENSCAFG00845000165	815	800	714	640
ENSCAFG00845024114	80	80	99	71
ENSCAFG00845026777	835	817	938	971
ENSCAFG00845000166	0	1	1	2
ENSCAFG00845024115	915	963	1633	1675
ENSCAFG00845026776	49	59	62	63
ENSCAFG00845000163	0	0	0	0
ENSCAFG00845026771	1718	1578	1658	1608
ENSCAFG00845000164	0	0	0	0
ENSCAFG00845026770	106	95	77	60
ENSCAFG00845000161	0	1	0	0
ENSCAFG00845026773	1	0	0	1
ENSCAFG00845000162	71	62	56	59
ENSCAFG00845024111	1255	1295	998	981
ENSCAFG00845026772	0	0	0	0
ENSCAFG00845000160	0	1	0	0
ENSCAFG00845012138	15226	15586	13659	14058
ENSCAFG00845012137	1	0	0	0

ENSCAFG00845014799	0	4	0	0
ENSCAFG00845012139	2	1	1	0
ENSCAFG00845012134	918	824	847	960
ENSCAFG00845014797	0	0	0	0
ENSCAFG00845012133	87	91	78	87
ENSCAFG00845014798	10	19	17	6
ENSCAFG00845012136	477	462	457	473
ENSCAFG00845014795	58	32	39	32
ENSCAFG00845012135	2992	2754	2748	2767
ENSCAFG00845014796	269	242	234	226
ENSCAFG00845014782	0	0	0	0
ENSCAFG00845014783	0	0	0	0
ENSCAFG00845012121	667	606	614	651
ENSCAFG00845014780	0	0	0	0
ENSCAFG00845012120	1135	1093	991	1025
ENSCAFG00845014781	0	0	0	0
ENSCAFG00845024109	331	345	265	317
ENSCAFG00845000138	5	5	1	8
ENSCAFG00845024105	21	20	47	37
ENSCAFG00845026768	0	0	0	0
ENSCAFG00845000139	524	478	546	492
ENSCAFG00845024106	6	2	3	5
ENSCAFG00845026767	857	876	1038	870
ENSCAFG00845000136	122	110	107	95
ENSCAFG00845002799	1064	904	877	870
ENSCAFG00845024107	2	0	1	0
ENSCAFG00845000137	0	0	0	1
ENSCAFG00845002798	1	0	3	0
ENSCAFG00845024108	108	71	107	99
ENSCAFG00845026769	834	784	686	809
ENSCAFG00845000134	352	385	377	362
ENSCAFG00845002797	0	0	0	0
ENSCAFG00845024101	280	307	255	273
ENSCAFG00845026764	666	684	635	637
ENSCAFG00845000135	782	773	776	757
ENSCAFG00845002796	0	0	0	0
ENSCAFG00845024102	1	1	6	2
ENSCAFG00845026763	809	799	701	739
ENSCAFG00845000132	75	60	91	80
ENSCAFG00845002795	0	0	0	0
ENSCAFG00845024103	1743	1508	1438	1430

ENSCAFG00845026766	671	773	582	651
ENSCAFG00845000133	182	110	149	130
ENSCAFG00845002794	2	10	0	0
ENSCAFG00845024104	1045	966	1065	1026
ENSCAFG00845026765	517	455	533	528
ENSCAFG00845000130	0	0	0	0
ENSCAFG00845002793	0	0	0	0
ENSCAFG00845026760	0	0	0	0
ENSCAFG00845000131	1	0	0	0
ENSCAFG00845002792	0	0	0	0
ENSCAFG00845002791	0	0	0	0
ENSCAFG00845026762	2	1	4	3
ENSCAFG00845002790	5	0	3	0
ENSCAFG00845024100	6	1	5	3
ENSCAFG00845026761	2872	2874	2840	2841
ENSCAFG00845012127	2549	2313	2388	2359
ENSCAFG00845012126	4	2	0	2
ENSCAFG00845012129	120	88	96	109
ENSCAFG00845014788	0	0	0	0
ENSCAFG00845012128	5161	5184	4974	5050
ENSCAFG00845014789	684	570	625	614
ENSCAFG00845012123	0	0	0	0
ENSCAFG00845014786	0	0	0	0
ENSCAFG00845012122	5	6	6	1
ENSCAFG00845014787	25	13	19	18
ENSCAFG00845012125	0	0	0	0
ENSCAFG00845014784	0	0	0	0
ENSCAFG00845012124	0	0	0	0
ENSCAFG00845014785	1064	1130	1020	1007
ENSCAFG00845014771	3493	3434	3343	3438
ENSCAFG00845014772	1529	1530	1482	1402
ENSCAFG00845012110	1	1	4	2
ENSCAFG00845014770	53	54	49	41
ENSCAFG00845000149	1	1	1	0
ENSCAFG00845026757	265	280	302	310
ENSCAFG00845026756	0	0	0	0
ENSCAFG00845000147	0	0	0	0
ENSCAFG00845026759	0	0	0	6
ENSCAFG00845000148	0	0	0	0
ENSCAFG00845026758	7	3	11	3
ENSCAFG00845000145	1969	1928	1852	1916

ENSCAFG00845026753	679	745	677	663
ENSCAFG00845000146	0	0	0	0
ENSCAFG00845026752	0	0	0	0
ENSCAFG00845000143	38	44	28	26
ENSCAFG00845026755	0	0	0	0
ENSCAFG00845000144	0	0	0	0
ENSCAFG00845026754	231	291	281	268
ENSCAFG00845000141	224	197	278	311
ENSCAFG00845000142	0	1	0	0
ENSCAFG00845026751	57	47	50	49
ENSCAFG00845000140	4	2	4	5
ENSCAFG00845012119	501	497	502	491
ENSCAFG00845012116	6060	6080	6920	7132
ENSCAFG00845014779	52	30	39	46
ENSCAFG00845012115	0	0	0	0
ENSCAFG00845012118	169	148	165	189
ENSCAFG00845014777	3	0	2	0
ENSCAFG00845012117	0	0	0	0
ENSCAFG00845014778	0	2	0	0
ENSCAFG00845012112	184	224	223	222
ENSCAFG00845014775	162	145	148	137
ENSCAFG00845012111	307	321	279	302
ENSCAFG00845014776	0	0	3	0
ENSCAFG00845012114	5054	4753	5122	5029
ENSCAFG00845014773	0	0	0	0
ENSCAFG00845012113	0	0	2	2
ENSCAFG00845014774	0	0	0	0
ENSCAFG00845024299	1669	1561	1338	1398
ENSCAFG00845024296	0	0	0	0
ENSCAFG00845024297	60	76	79	72
ENSCAFG00845024298	0	0	0	0
ENSCAFG00845024291	0	1	0	0
ENSCAFG00845024292	0	0	0	0
ENSCAFG00845024293	0	0	0	0
ENSCAFG00845024294	2	0	0	0
ENSCAFG00845024290	1939	1830	1672	1740
ENSCAFG00845026908	0	0	0	0
ENSCAFG00845026907	466	415	526	510
ENSCAFG00845002939	1	0	0	0
ENSCAFG00845002938	0	2	0	0
ENSCAFG00845026909	0	0	0	0

ENSCAFG00845002937	0	0	0	0
ENSCAFG00845026904	0	0	0	0
ENSCAFG00845002936	0	2	2	2
ENSCAFG00845026903	0	1	0	0
ENSCAFG00845002935	5	9	2	2
ENSCAFG00845026906	0	0	0	0
ENSCAFG00845002934	0	0	2	0
ENSCAFG00845026905	0	0	0	0
ENSCAFG00845002933	617	604	550	634
ENSCAFG00845026900	267	239	292	294
ENSCAFG00845002932	12	7	13	8
ENSCAFG00845002931	0	0	0	0
ENSCAFG00845026902	0	0	0	0
ENSCAFG00845002930	1609	1595	1354	1356
ENSCAFG00845026901	0	0	0	0
ENSCAFG00845014928	0	0	0	0
ENSCAFG00845014929	1	4	4	5
ENSCAFG00845014926	0	0	1	1
ENSCAFG00845014927	413	366	397	335
ENSCAFG00845014924	0	0	0	0
ENSCAFG00845014925	6575	6266	6218	6700
ENSCAFG00845014922	1349	1274	1509	1402
ENSCAFG00845014923	0	0	0	0
ENSCAFG00845014920	0	0	1	0
ENSCAFG00845014921	0	0	0	0
ENSCAFG00845002949	267	309	263	280
ENSCAFG00845002948	0	0	0	0
ENSCAFG00845002947	15967	15562	16309	17707
ENSCAFG00845002946	0	0	0	0
ENSCAFG00845002945	6	1	0	8
ENSCAFG00845002944	45	43	48	43
ENSCAFG00845002943	685	649	615	670
ENSCAFG00845002942	1035	954	1035	1061
ENSCAFG00845002941	0	0	0	0
ENSCAFG00845002940	0	0	0	0
ENSCAFG00845014919	553	548	498	532
ENSCAFG00845014917	2018	1976	1921	1892
ENSCAFG00845014918	5	10	7	8
ENSCAFG00845014915	0	0	0	0
ENSCAFG00845014916	0	0	0	0
ENSCAFG00845014913	0	0	0	0

ENSCAFG00845014914	9	23	8	15
ENSCAFG00845014911	656	635	726	781
ENSCAFG00845014912	5	1	6	10
ENSCAFG00845014910	1328	1252	1197	1200
ENSCAFG00845002919	1	0	0	0
ENSCAFG00845002918	1	0	0	0
ENSCAFG00845002917	0	0	0	2
ENSCAFG00845002916	97	75	79	76
ENSCAFG00845002915	100	112	98	80
ENSCAFG00845002914	9	1	0	2
ENSCAFG00845002913	0	0	0	0
ENSCAFG00845002912	32	22	25	23
ENSCAFG00845002911	0	0	0	0
ENSCAFG00845002910	167	167	89	110
ENSCAFG00845014908	0	0	0	0
ENSCAFG00845014909	0	0	0	0
ENSCAFG00845014906	1656	1479	1627	1614
ENSCAFG00845014907	1660	1543	1552	1479
ENSCAFG00845014904	0	0	0	0
ENSCAFG00845014905	289	303	257	291
ENSCAFG00845014902	0	0	0	0
ENSCAFG00845014903	104	112	77	75
ENSCAFG00845014900	0	0	0	0
ENSCAFG00845014901	835	840	844	848
ENSCAFG00845002909	1	0	0	0
ENSCAFG00845002929	0	0	0	0
ENSCAFG00845002928	0	0	0	0
ENSCAFG00845002927	0	0	0	0
ENSCAFG00845002926	0	0	0	0
ENSCAFG00845002925	124	143	120	144
ENSCAFG00845002924	2	2	0	0
ENSCAFG00845002923	1	1	0	7
ENSCAFG00845002922	291	344	251	234
ENSCAFG00845002921	0	0	0	0
ENSCAFG00845002920	3	1	1	2
ENSCAFG00845002908	0	0	0	0
ENSCAFG00845002907	0	0	0	0
ENSCAFG00845002906	3	14	14	14
ENSCAFG00845002905	1630	1554	1591	1595
ENSCAFG00845002904	0	1	1	0
ENSCAFG00845002903	0	0	0	0

ENSCAFG00845002902	2255	2297	2088	2202
ENSCAFG00845002901	0	0	0	0
ENSCAFG00845002900	0	0	0	0
ENSCAFG00845014881	0	0	0	0
ENSCAFG00845014882	5	9	15	5
ENSCAFG00845012220	0	0	0	0
ENSCAFG00845014880	1543	1560	1418	1346
ENSCAFG00845024208	0	0	0	0
ENSCAFG00845024209	0	0	1	1
ENSCAFG00845000239	107	81	87	78
ENSCAFG00845000237	0	0	0	0
ENSCAFG00845024204	131	95	74	109
ENSCAFG00845026867	0	1	0	0
ENSCAFG00845000238	0	0	0	0
ENSCAFG00845002899	945	852	934	925
ENSCAFG00845024205	0	0	0	0
ENSCAFG00845026866	0	0	0	0
ENSCAFG00845000235	0	0	0	0
ENSCAFG00845002898	0	2	0	2
ENSCAFG00845024206	157	138	166	168
ENSCAFG00845026869	18	20	6	15
ENSCAFG00845000236	0	0	0	0
ENSCAFG00845002897	0	0	0	0
ENSCAFG00845024207	0	0	0	0
ENSCAFG00845026868	3	3	2	0
ENSCAFG00845000233	977	946	842	912
ENSCAFG00845002896	7	13	9	9
ENSCAFG00845024200	0	0	0	0
ENSCAFG00845026863	91	79	78	72
ENSCAFG00845000234	38	22	22	31
ENSCAFG00845002895	0	0	0	0
ENSCAFG00845024201	0	0	0	0
ENSCAFG00845026862	449	437	412	470
ENSCAFG00845000231	0	0	0	0
ENSCAFG00845002894	0	3	0	0
ENSCAFG00845024202	0	0	0	0
ENSCAFG00845026865	0	0	0	0
ENSCAFG00845000232	0	0	0	0
ENSCAFG00845002893	0	0	0	0
ENSCAFG00845024203	33	33	31	26
ENSCAFG00845026864	633	703	654	630

ENSCAFG00845002892	0	0	0	0
ENSCAFG00845000230	61	80	60	89
ENSCAFG00845002891	0	0	0	0
ENSCAFG00845002890	0	0	1	2
ENSCAFG00845026860	0	0	0	0
ENSCAFG00845012229	47	49	55	53
ENSCAFG00845012226	213	211	195	200
ENSCAFG00845014889	1480	1394	1320	1194
ENSCAFG00845012225	34	39	39	57
ENSCAFG00845012228	731	652	504	499
ENSCAFG00845014887	608	513	526	501
ENSCAFG00845012227	0	0	0	0
ENSCAFG00845014888	0	0	0	0
ENSCAFG00845012222	0	0	0	0
ENSCAFG00845014885	27	7	18	11
ENSCAFG00845012221	551	541	485	455
ENSCAFG00845014886	1413	1475	1318	1364
ENSCAFG00845012224	0	0	0	0
ENSCAFG00845014883	677	704	758	679
ENSCAFG00845012223	3	3	3	4
ENSCAFG00845014884	11	0	8	13
ENSCAFG00845014870	1	1	1	0
ENSCAFG00845014871	4	1	0	1
ENSCAFG00845026859	0	0	0	0
ENSCAFG00845000248	0	0	0	0
ENSCAFG00845026856	673	634	737	726
ENSCAFG00845000249	0	0	0	1
ENSCAFG00845026855	1	0	1	0
ENSCAFG00845000246	0	0	0	0
ENSCAFG00845026858	0	0	0	0
ENSCAFG00845000247	0	1	1	0
ENSCAFG00845026857	16	16	24	13
ENSCAFG00845000244	0	0	1	1
ENSCAFG00845026852	191	226	205	247
ENSCAFG00845000245	0	0	1	0
ENSCAFG00845026851	0	0	0	0
ENSCAFG00845000242	1055	1076	1074	1001
ENSCAFG00845026854	0	0	0	0
ENSCAFG00845000243	0	0	0	0
ENSCAFG00845026853	0	0	0	0
ENSCAFG00845000240	974	906	922	984

ENSCAFG00845000241	0	0	0	0
ENSCAFG00845026850	0	1	3	0
ENSCAFG00845012219	463	465	489	505
ENSCAFG00845012218	0	0	0	0
ENSCAFG00845012215	2	2	0	0
ENSCAFG00845014878	0	0	3	3
ENSCAFG00845012214	1	0	0	0
ENSCAFG00845014879	1	0	0	0
ENSCAFG00845012217	0	0	0	0
ENSCAFG00845014876	0	0	0	0
ENSCAFG00845012216	0	0	0	0
ENSCAFG00845014877	0	1	0	1
ENSCAFG00845012211	610	579	505	560
ENSCAFG00845014874	409	449	436	478
ENSCAFG00845012210	210	178	152	153
ENSCAFG00845014875	1	0	0	0
ENSCAFG00845012213	150	113	127	108
ENSCAFG00845014872	0	0	0	0
ENSCAFG00845012212	46	61	55	43
ENSCAFG00845014873	0	0	0	0
ENSCAFG00845014860	119	104	109	129
ENSCAFG00845000219	63	52	52	57
ENSCAFG00845026849	0	0	0	0
ENSCAFG00845026848	74	65	75	83
ENSCAFG00845000217	12	9	8	17
ENSCAFG00845000218	0	0	0	0
ENSCAFG00845002879	0	0	0	2
ENSCAFG00845000215	146	157	148	176
ENSCAFG00845002878	8	11	27	19
ENSCAFG00845026845	0	0	0	0
ENSCAFG00845000216	0	0	0	0
ENSCAFG00845002877	0	0	0	0
ENSCAFG00845026844	0	0	0	0
ENSCAFG00845000213	877	829	904	841
ENSCAFG00845002876	269	284	256	246
ENSCAFG00845026847	769	764	721	769
ENSCAFG00845000214	0	0	0	0
ENSCAFG00845002875	0	0	0	0
ENSCAFG00845026846	0	0	0	0
ENSCAFG00845000211	188	166	159	142
ENSCAFG00845002874	103	105	88	94

ENSCAFG00845026841	0	3	0	0
ENSCAFG00845000212	1638	1500	1546	1508
ENSCAFG00845002873	0	0	0	0
ENSCAFG00845026840	0	0	0	0
ENSCAFG00845002872	0	0	0	0
ENSCAFG00845026843	3	2	6	4
ENSCAFG00845000210	1021	938	938	981
ENSCAFG00845002871	276	296	313	342
ENSCAFG00845026842	0	0	0	0
ENSCAFG00845002870	1921	1970	1988	2008
ENSCAFG00845012208	0	0	0	0
ENSCAFG00845012207	536	473	455	519
ENSCAFG00845014869	1068	1080	1044	967
ENSCAFG00845012209	0	1	0	0
ENSCAFG00845012204	5	1	2	2
ENSCAFG00845014867	627	679	803	863
ENSCAFG00845012203	3543	3463	3536	3639
ENSCAFG00845014868	0	0	0	0
ENSCAFG00845012206	220	252	208	226
ENSCAFG00845014865	214	218	227	201
ENSCAFG00845012205	0	0	0	0
ENSCAFG00845014866	2	5	11	3
ENSCAFG00845012200	0	0	0	0
ENSCAFG00845014863	148	162	153	128
ENSCAFG00845014864	1	0	3	0
ENSCAFG00845012202	0	0	0	1
ENSCAFG00845014861	0	0	0	0
ENSCAFG00845012201	497	462	456	546
ENSCAFG00845014862	100	82	82	103
ENSCAFG00845026838	0	0	0	0
ENSCAFG00845026837	8	10	9	5
ENSCAFG00845000228	0	0	0	0
ENSCAFG00845000229	0	0	0	0
ENSCAFG00845026839	0	0	0	0
ENSCAFG00845000226	7137	6810	7491	7533
ENSCAFG00845002889	8	2	2	5
ENSCAFG00845026834	0	2	0	0
ENSCAFG00845000227	0	0	0	0
ENSCAFG00845002888	0	0	0	0
ENSCAFG00845026833	502	499	502	515
ENSCAFG00845000224	0	0	0	0

ENSCAFG00845002887	235	209	229	242
ENSCAFG00845000225	0	0	0	0
ENSCAFG00845002886	538	578	578	609
ENSCAFG00845026835	1	1	3	2
ENSCAFG00845000222	557	552	596	542
ENSCAFG00845002885	194	172	159	182
ENSCAFG00845026830	0	0	0	0
ENSCAFG00845000223	10189	10118	9464	9651
ENSCAFG00845002884	369	373	350	326
ENSCAFG00845000220	1	0	0	0
ENSCAFG00845002883	119	121	126	114
ENSCAFG00845026832	372	315	378	408
ENSCAFG00845000221	0	0	0	0
ENSCAFG00845002882	0	0	0	0
ENSCAFG00845026831	256	260	240	219
ENSCAFG00845002881	77	82	73	91
ENSCAFG00845002880	8	5	1	4
ENSCAFG00845014858	419	340	323	354
ENSCAFG00845014859	1	0	1	0
ENSCAFG00845014856	69	62	45	40
ENSCAFG00845014857	0	0	0	0
ENSCAFG00845014854	0	0	0	0
ENSCAFG00845014855	4	7	2	3
ENSCAFG00845014852	874	906	821	927
ENSCAFG00845014853	0	1	0	3
ENSCAFG00845014850	0	0	0	0
ENSCAFG00845014851	0	0	1	0
ENSCAFG00845026827	1	3	0	1
ENSCAFG00845002859	0	1	1	0
ENSCAFG00845026826	1	0	7	2
ENSCAFG00845002858	1	0	0	0
ENSCAFG00845026829	1105	1067	1133	1171
ENSCAFG00845002857	0	0	2	2
ENSCAFG00845026828	0	1	1	0
ENSCAFG00845002856	24	9	13	8
ENSCAFG00845026823	0	0	0	0
ENSCAFG00845002855	0	0	0	0
ENSCAFG00845026822	6	1	2	1
ENSCAFG00845002854	0	0	0	0
ENSCAFG00845026825	1	0	0	0
ENSCAFG00845002853	10	4	6	6

ENSCAFG00845026824	0	0	0	0
ENSCAFG00845002852	14	14	22	33
ENSCAFG00845002851	0	0	0	0
ENSCAFG00845002850	0	0	0	0
ENSCAFG00845026821	1	1	2	0
ENSCAFG00845026820	3	1	0	1
ENSCAFG00845014849	5	2	1	3
ENSCAFG00845014847	1	0	1	2
ENSCAFG00845014848	2430	2250	2298	2485
ENSCAFG00845014845	411	433	544	524
ENSCAFG00845014846	3	7	7	17
ENSCAFG00845014843	55	44	56	75
ENSCAFG00845014844	982	979	899	919
ENSCAFG00845014841	49	59	53	78
ENSCAFG00845014842	101	108	91	75
ENSCAFG00845014840	600	601	575	572
ENSCAFG00845026819	0	0	0	0
ENSCAFG00845000208	36	55	50	52
ENSCAFG00845000209	3	1	2	4
ENSCAFG00845026815	38	39	33	20
ENSCAFG00845000206	462	447	387	425
ENSCAFG00845002869	0	0	0	0
ENSCAFG00845026818	0	0	0	0
ENSCAFG00845000207	0	0	0	0
ENSCAFG00845002868	119	108	91	119
ENSCAFG00845026817	0	0	0	2
ENSCAFG00845000204	940	886	1030	1124
ENSCAFG00845002867	0	0	0	0
ENSCAFG00845026812	462	468	394	449
ENSCAFG00845000205	266	242	275	242
ENSCAFG00845002866	1231	1208	1214	1198
ENSCAFG00845026811	0	0	0	0
ENSCAFG00845000202	0	0	0	0
ENSCAFG00845002865	0	0	0	1
ENSCAFG00845026814	32	34	39	45
ENSCAFG00845000203	0	3	0	0
ENSCAFG00845002864	271	264	323	315
ENSCAFG00845026813	75	69	73	85
ENSCAFG00845000200	2288	2128	2124	1999
ENSCAFG00845002863	2	1	1	4
ENSCAFG00845000201	0	0	0	0

ENSCAFG00845002862	12	6	10	4
ENSCAFG00845002861	0	0	0	0
ENSCAFG00845026810	604	579	700	697
ENSCAFG00845002860	0	0	0	0
ENSCAFG00845014838	1526	1517	1632	1745
ENSCAFG00845014839	2	4	6	7
ENSCAFG00845014836	1185	1074	1149	1164
ENSCAFG00845014837	542	558	556	515
ENSCAFG00845014834	0	0	0	0
ENSCAFG00845014835	285	209	253	250
ENSCAFG00845014832	264	251	231	280
ENSCAFG00845014833	0	0	0	0
ENSCAFG00845014830	0	0	0	0
ENSCAFG00845014831	0	0	0	0
ENSCAFG00845026809	2487	2542	2338	2212
ENSCAFG00845026808	459	417	408	397
ENSCAFG00845002839	0	0	0	0
ENSCAFG00845002838	0	0	0	0
ENSCAFG00845026805	0	0	0	0
ENSCAFG00845002837	0	0	0	0
ENSCAFG00845026804	426	479	431	429
ENSCAFG00845002836	0	0	0	0
ENSCAFG00845026807	160	114	117	121
ENSCAFG00845002835	31	45	35	36
ENSCAFG00845026806	907	804	908	861
ENSCAFG00845002834	5	3	4	3
ENSCAFG00845026801	2	2	3	2
ENSCAFG00845002833	261	249	233	285
ENSCAFG00845002832	13	17	13	12
ENSCAFG00845026803	7	6	13	16
ENSCAFG00845002831	0	0	0	0
ENSCAFG00845026802	0	1	1	0
ENSCAFG00845002830	0	0	0	0
ENSCAFG00845014829	0	0	0	0
ENSCAFG00845014827	247	260	210	234
ENSCAFG00845014828	10	10	10	15
ENSCAFG00845014825	1101	1070	1041	923
ENSCAFG00845014826	11	23	10	8
ENSCAFG00845014823	96	81	50	58
ENSCAFG00845014824	0	0	0	0
ENSCAFG00845014821	106	92	94	88

ENSCAFG00845014822	0	0	0	0
ENSCAFG00845014820	44	43	19	32
ENSCAFG00845002849	860	782	851	877
ENSCAFG00845002848	0	0	0	0
ENSCAFG00845002847	0	0	0	0
ENSCAFG00845002846	0	0	0	0
ENSCAFG00845002845	0	0	0	0
ENSCAFG00845002844	0	0	0	0
ENSCAFG00845002843	0	0	0	0
ENSCAFG00845002842	105	115	83	104
ENSCAFG00845002841	201	183	194	191
ENSCAFG00845002840	128	142	136	141
ENSCAFG00845014818	6	9	5	3
ENSCAFG00845014819	0	0	0	0
ENSCAFG00845014816	0	0	0	0
ENSCAFG00845014817	117	152	130	130
ENSCAFG00845014814	0	0	0	0
ENSCAFG00845014815	0	0	0	0
ENSCAFG00845014812	542	563	509	534
ENSCAFG00845014813	638	594	628	675
ENSCAFG00845014810	1606	1540	1512	1591
ENSCAFG00845014811	0	0	0	0
ENSCAFG00845024288	0	0	2	0
ENSCAFG00845024289	646	584	702	783
ENSCAFG00845024284	0	0	1	2
ENSCAFG00845024285	22	20	23	26
ENSCAFG00845024286	0	0	0	0
ENSCAFG00845024287	116	99	175	157
ENSCAFG00845024280	1	0	0	0
ENSCAFG00845024281	485	463	467	370
ENSCAFG00845024282	3	1	0	0
ENSCAFG00845024283	558	638	571	558
ENSCAFG00845012295	0	0	0	0
ENSCAFG00845012294	102	113	110	128
ENSCAFG00845012297	2	0	0	0
ENSCAFG00845012296	562	542	428	447
ENSCAFG00845012291	0	0	0	0
ENSCAFG00845012290	3	5	2	6
ENSCAFG00845012293	425	335	406	341
ENSCAFG00845012292	0	0	1	0
ENSCAFG00845024277	113	91	114	114

ENSCAFG00845024278	0	0	0	0
ENSCAFG00845024279	39	50	26	33
ENSCAFG00845024273	53	60	66	59
ENSCAFG00845024274	0	3	1	2
ENSCAFG00845024275	1	4	0	0
ENSCAFG00845024276	0	0	2	0
ENSCAFG00845024270	0	0	0	0
ENSCAFG00845024271	120	131	85	70
ENSCAFG00845024272	1	0	0	1
ENSCAFG00845012299	656	686	599	615
ENSCAFG00845012298	1966	1923	1810	1804
ENSCAFG00845012284	0	0	0	0
ENSCAFG00845012283	1734	1475	1400	1293
ENSCAFG00845012286	1691	1693	1636	1567
ENSCAFG00845012285	0	0	0	0
ENSCAFG00845012280	0	0	0	0
ENSCAFG00845012282	603	605	607	651
ENSCAFG00845012281	839	786	806	837
ENSCAFG00845000299	0	0	0	0
ENSCAFG00845024266	0	0	0	0
ENSCAFG00845024267	1150	1124	1081	1111
ENSCAFG00845000297	2271	2136	1228	1293
ENSCAFG00845024268	2402	2315	2141	2141
ENSCAFG00845000298	0	2	0	0
ENSCAFG00845000295	2	1	2	0
ENSCAFG00845024262	0	0	0	1
ENSCAFG00845000296	0	0	0	0
ENSCAFG00845024263	214	227	253	294
ENSCAFG00845000293	0	0	0	0
ENSCAFG00845024264	0	4	1	3
ENSCAFG00845000294	1113	1069	1056	1077
ENSCAFG00845024265	1027	952	964	1015
ENSCAFG00845000291	0	0	0	0
ENSCAFG00845000292	0	0	0	0
ENSCAFG00845024260	465	477	382	413
ENSCAFG00845000290	2	1	0	0
ENSCAFG00845024261	0	0	0	0
ENSCAFG00845012288	0	0	0	0
ENSCAFG00845012287	3	0	0	0
ENSCAFG00845012289	192	248	179	196
ENSCAFG00845012273	0	0	0	0

ENSCAFG00845012272	0	0	0	0
ENSCAFG00845012275	63	51	58	52
ENSCAFG00845012274	69	73	74	65
ENSCAFG00845012271	0	0	0	0
ENSCAFG00845012270	11	4	8	6
ENSCAFG00845024259	0	0	0	0
ENSCAFG00845024255	887	808	897	806
ENSCAFG00845024256	0	0	0	0
ENSCAFG00845024257	1309	1226	1142	1175
ENSCAFG00845024258	0	0	0	0
ENSCAFG00845024251	22	20	25	16
ENSCAFG00845024252	439	415	447	431
ENSCAFG00845024253	87	80	100	85
ENSCAFG00845024254	0	0	0	0
ENSCAFG00845024250	0	0	0	0
ENSCAFG00845012277	0	3	0	0
ENSCAFG00845012276	69	59	53	43
ENSCAFG00845012279	0	0	0	0
ENSCAFG00845012278	0	0	0	0
ENSCAFG00845012262	65	70	88	89
ENSCAFG00845012261	0	0	0	0
ENSCAFG00845012264	0	1	0	0
ENSCAFG00845012263	0	0	0	0
ENSCAFG00845012260	0	0	0	0
ENSCAFG00845024248	6	1	1	7
ENSCAFG00845024249	33	19	29	26
ENSCAFG00845000279	0	0	0	0
ENSCAFG00845000277	0	0	0	3
ENSCAFG00845024244	1214	1199	1128	1137
ENSCAFG00845000278	5747	5515	5849	5566
ENSCAFG00845024245	0	0	0	0
ENSCAFG00845000275	0	0	0	0
ENSCAFG00845024246	662	687	681	635
ENSCAFG00845000276	0	0	0	0
ENSCAFG00845024247	0	0	0	0
ENSCAFG00845000273	1134	1059	987	1097
ENSCAFG00845024240	0	0	0	0
ENSCAFG00845000274	0	0	0	0
ENSCAFG00845024241	0	1	0	1
ENSCAFG00845000271	0	0	0	0
ENSCAFG00845024242	2293	2115	2021	1991

ENSCAFG00845000272	0	0	0	0
ENSCAFG00845024243	292	260	300	256
ENSCAFG00845000270	0	0	0	0
ENSCAFG00845012269	1352	1322	1177	1182
ENSCAFG00845012266	377	358	324	376
ENSCAFG00845012265	920	847	790	894
ENSCAFG00845012268	40	32	42	29
ENSCAFG00845012267	3207	2955	3210	3156
ENSCAFG00845012251	1036	1019	1009	1135
ENSCAFG00845012250	0	0	0	0
ENSCAFG00845012253	358	292	353	447
ENSCAFG00845012252	17	45	14	22
ENSCAFG00845024237	0	0	0	0
ENSCAFG00845024238	0	0	0	0
ENSCAFG00845026899	402	313	275	316
ENSCAFG00845024239	467	467	494	487
ENSCAFG00845000288	2465	2472	2495	2439
ENSCAFG00845024233	613	541	575	591
ENSCAFG00845026896	0	0	0	0
ENSCAFG00845000289	3	5	7	6
ENSCAFG00845024234	346	337	309	352
ENSCAFG00845026895	9	8	15	8
ENSCAFG00845000286	48	37	53	53
ENSCAFG00845024235	191	150	255	294
ENSCAFG00845026898	1	1	0	1
ENSCAFG00845000287	172	227	135	145
ENSCAFG00845024236	0	0	2	0
ENSCAFG00845026897	3	0	0	1
ENSCAFG00845000284	0	0	0	0
ENSCAFG00845026892	3802	3656	3280	3321
ENSCAFG00845000285	0	0	0	0
ENSCAFG00845024230	1526	1451	1379	1512
ENSCAFG00845026891	0	0	0	0
ENSCAFG00845000282	0	0	0	0
ENSCAFG00845024231	1090	1032	1204	1173
ENSCAFG00845026894	118	120	64	72
ENSCAFG00845000283	0	0	0	3
ENSCAFG00845024232	0	0	0	0
ENSCAFG00845000280	0	0	0	0
ENSCAFG00845000281	3	0	2	3
ENSCAFG00845026890	0	0	1	0

ENSCAFG00845012259	0	0	0	0
ENSCAFG00845012258	0	0	0	0
ENSCAFG00845012255	1039	956	849	938
ENSCAFG00845012254	0	0	0	0
ENSCAFG00845012257	2	3	0	0
ENSCAFG00845012256	1261	1220	1207	1181
ENSCAFG00845012240	12	10	11	9
ENSCAFG00845012242	60	61	114	97
ENSCAFG00845012241	121	111	126	121
ENSCAFG00845000259	1	1	1	4
ENSCAFG00845024226	0	0	0	0
ENSCAFG00845026889	2	0	1	0
ENSCAFG00845024227	909	848	867	923
ENSCAFG00845026888	0	0	0	0
ENSCAFG00845000257	197	188	195	214
ENSCAFG00845024228	575	534	408	437
ENSCAFG00845000258	0	0	0	0
ENSCAFG00845024229	0	0	0	0
ENSCAFG00845000255	0	0	0	0
ENSCAFG00845024222	0	0	0	0
ENSCAFG00845026885	84	89	102	66
ENSCAFG00845000256	473	425	499	509
ENSCAFG00845024223	0	0	2	0
ENSCAFG00845026884	388	330	329	351
ENSCAFG00845000253	0	0	0	0
ENSCAFG00845024224	1752	1716	1520	1640
ENSCAFG00845026887	0	0	0	0
ENSCAFG00845000254	0	0	0	0
ENSCAFG00845024225	0	0	0	0
ENSCAFG00845026886	1584	1515	1617	1588
ENSCAFG00845000251	0	0	0	0
ENSCAFG00845026881	111	118	106	108
ENSCAFG00845000252	0	0	0	0
ENSCAFG00845026880	547	580	526	492
ENSCAFG00845026883	9	3	4	3
ENSCAFG00845000250	0	0	0	0
ENSCAFG00845024221	478	431	528	582
ENSCAFG00845026882	0	0	0	0
ENSCAFG00845012248	2	2	0	1
ENSCAFG00845012247	0	0	0	0
ENSCAFG00845012249	33	46	36	34

ENSCAFG00845012244	0	0	0	0
ENSCAFG00845012243	75	56	64	67
ENSCAFG00845012246	3	2	0	1
ENSCAFG00845012245	4075	3895	3624	3705
ENSCAFG00845014892	108	135	136	142
ENSCAFG00845014893	248	304	329	267
ENSCAFG00845012231	2263	2173	2280	2368
ENSCAFG00845014890	0	0	0	0
ENSCAFG00845012230	0	0	0	0
ENSCAFG00845014891	5	10	12	8
ENSCAFG00845024219	0	0	0	0
ENSCAFG00845024215	2838	2690	2727	2435
ENSCAFG00845026878	0	0	0	0
ENSCAFG00845024216	8	13	12	10
ENSCAFG00845026877	0	0	0	0
ENSCAFG00845000268	4	4	5	2
ENSCAFG00845024217	24	32	22	12
ENSCAFG00845000269	0	0	0	0
ENSCAFG00845024218	989	954	910	946
ENSCAFG00845026879	0	4	0	0
ENSCAFG00845000266	213	216	193	193
ENSCAFG00845024211	0	0	0	0
ENSCAFG00845026874	6	10	11	7
ENSCAFG00845000267	662	629	631	621
ENSCAFG00845024212	0	0	0	0
ENSCAFG00845026873	485	418	417	426
ENSCAFG00845000264	0	0	0	0
ENSCAFG00845024213	2355	2255	2308	2211
ENSCAFG00845026876	12	13	14	23
ENSCAFG00845000265	0	0	0	0
ENSCAFG00845024214	0	0	0	0
ENSCAFG00845026875	0	0	0	0
ENSCAFG00845000262	0	0	0	0
ENSCAFG00845026870	818	842	919	869
ENSCAFG00845000263	0	0	0	0
ENSCAFG00845000260	0	0	0	0
ENSCAFG00845026872	3	0	2	5
ENSCAFG00845000261	1395	1393	1305	1265
ENSCAFG00845024210	123	112	124	95
ENSCAFG00845026871	0	0	0	0
ENSCAFG00845012237	0	0	0	0

ENSCAFG00845012236	561	489	664	628
ENSCAFG00845012239	0	0	0	0
ENSCAFG00845014898	0	1	1	0
ENSCAFG00845012238	0	0	0	0
ENSCAFG00845014899	171	200	138	168
ENSCAFG00845012233	310	251	240	278
ENSCAFG00845014896	0	0	0	0
ENSCAFG00845012232	41	39	43	36
ENSCAFG00845014897	690	755	651	690
ENSCAFG00845012235	0	0	0	0
ENSCAFG00845014894	521	490	440	497
ENSCAFG00845012234	2416	2401	2074	2255
ENSCAFG00845014895	2	1	0	0
ENSCAFG00845000408	0	0	0	0
ENSCAFG00845000409	23	21	19	14
ENSCAFG00845000406	0	0	0	0
ENSCAFG00845000407	974	917	989	942
ENSCAFG00845000404	268	285	302	323
ENSCAFG00845000405	0	0	0	0
ENSCAFG00845000402	10	7	6	4
ENSCAFG00845000403	0	0	0	0
ENSCAFG00845000400	0	0	0	0
ENSCAFG00845000401	410	394	335	376
ENSCAFG00845012341	0	0	0	0
ENSCAFG00845012340	0	0	0	0
ENSCAFG00845024329	654	624	663	679
ENSCAFG00845000358	0	0	0	1
ENSCAFG00845024325	0	0	0	0
ENSCAFG00845026988	441	441	421	428
ENSCAFG00845000359	0	0	0	0
ENSCAFG00845026987	1780	1773	1815	1622
ENSCAFG00845000356	1	0	0	0
ENSCAFG00845024327	673	762	562	545
ENSCAFG00845000357	31	33	69	48
ENSCAFG00845024328	1	1	1	3
ENSCAFG00845026989	0	0	0	0
ENSCAFG00845000354	0	0	0	0
ENSCAFG00845024321	6	1	0	3
ENSCAFG00845026984	0	0	0	0
ENSCAFG00845000355	0	0	0	0
ENSCAFG00845024322	0	0	0	0

ENSCAFG00845026983	3	3	9	8
ENSCAFG00845000352	0	0	0	0
ENSCAFG00845024323	2	2	0	2
ENSCAFG00845026986	1	4	1	2
ENSCAFG00845000353	11	11	4	2
ENSCAFG00845024324	43	33	32	39
ENSCAFG00845026985	0	0	0	0
ENSCAFG00845000350	0	0	0	0
ENSCAFG00845026980	308	270	284	290
ENSCAFG00845000351	0	0	0	0
ENSCAFG00845026982	287	283	221	213
ENSCAFG00845024320	593	711	804	749
ENSCAFG00845026981	449	493	419	488
ENSCAFG00845012347	0	0	0	0
ENSCAFG00845012346	1	4	1	1
ENSCAFG00845012349	3	2	7	1
ENSCAFG00845012348	0	0	0	0
ENSCAFG00845012343	0	0	0	0
ENSCAFG00845012342	188	185	154	182
ENSCAFG00845012345	0	0	0	0
ENSCAFG00845012344	0	0	2	1
ENSCAFG00845014991	99	88	98	72
ENSCAFG00845014992	0	0	0	0
ENSCAFG00845012330	3	6	2	11
ENSCAFG00845014990	0	0	0	0
ENSCAFG00845024318	0	0	0	0
ENSCAFG00845024319	3475	3317	3205	3169
ENSCAFG00845000369	0	0	0	0
ENSCAFG00845024314	0	0	0	0
ENSCAFG00845026977	424	391	436	415
ENSCAFG00845024315	0	0	0	0
ENSCAFG00845026976	2	5	5	3
ENSCAFG00845000367	1069	969	832	866
ENSCAFG00845024316	1838	1851	1840	1788
ENSCAFG00845026979	767	783	752	765
ENSCAFG00845000368	0	0	1	0
ENSCAFG00845024317	1985	1939	2070	2063
ENSCAFG00845026978	2	2	3	4
ENSCAFG00845000365	0	0	0	0
ENSCAFG00845026973	2841	2819	3105	3089
ENSCAFG00845000366	2	14	0	2

ENSCAFG00845024311	0	1	0	0
ENSCAFG00845000363	1	1	1	0
ENSCAFG00845024312	3	5	2	7
ENSCAFG00845026975	0	0	0	0
ENSCAFG00845000364	0	0	0	0
ENSCAFG00845024313	6	9	1	5
ENSCAFG00845026974	0	0	0	0
ENSCAFG00845000361	0	0	0	0
ENSCAFG00845000362	2	0	0	0
ENSCAFG00845026971	17	7	11	9
ENSCAFG00845000360	0	0	0	0
ENSCAFG00845026970	1	1	3	0
ENSCAFG00845012339	0	0	0	0
ENSCAFG00845012336	1978	1846	1928	2021
ENSCAFG00845014999	0	0	0	0
ENSCAFG00845012335	299	279	228	307
ENSCAFG00845012338	531	537	576	553
ENSCAFG00845014997	0	0	0	0
ENSCAFG00845012337	1054	988	1080	1075
ENSCAFG00845014998	0	0	0	0
ENSCAFG00845012332	0	0	0	0
ENSCAFG00845014995	0	0	0	0
ENSCAFG00845012331	8	4	6	10
ENSCAFG00845014996	0	0	0	0
ENSCAFG00845012334	1	0	2	0
ENSCAFG00845014993	1340	1300	1262	1312
ENSCAFG00845012333	0	0	0	0
ENSCAFG00845014994	0	0	0	0
ENSCAFG00845014980	471	426	446	493
ENSCAFG00845014981	1	0	0	0
ENSCAFG00845024307	832	750	830	946
ENSCAFG00845024308	62	55	74	79
ENSCAFG00845026969	1	2	3	1
ENSCAFG00845000338	387	475	392	387
ENSCAFG00845024309	0	0	0	0
ENSCAFG00845000339	345	346	381	373
ENSCAFG00845000336	0	0	0	0
ENSCAFG00845002999	1	3	1	0
ENSCAFG00845024303	404	394	371	359
ENSCAFG00845026966	333	328	278	315
ENSCAFG00845000337	0	0	0	0

ENSCAFG00845002998	3181	3176	2991	3028
ENSCAFG00845024304	0	0	0	0
ENSCAFG00845026965	0	0	0	0
ENSCAFG00845000334	251	239	209	206
ENSCAFG00845002997	0	0	0	0
ENSCAFG00845024305	0	0	0	0
ENSCAFG00845026968	481	425	551	566
ENSCAFG00845000335	1	1	0	1
ENSCAFG00845002996	3120	3155	2575	2479
ENSCAFG00845024306	3	8	0	1
ENSCAFG00845026967	447	405	379	394
ENSCAFG00845000332	2	10	0	3
ENSCAFG00845002995	2590	2619	2774	2902
ENSCAFG00845026962	212	245	226	227
ENSCAFG00845000333	0	0	0	0
ENSCAFG00845002994	0	0	0	0
ENSCAFG00845024300	1	0	4	6
ENSCAFG00845026961	0	0	0	0
ENSCAFG00845000330	457	472	465	490
ENSCAFG00845002993	0	0	0	0
ENSCAFG00845024301	4	3	3	1
ENSCAFG00845026964	1235	1190	1272	1299
ENSCAFG00845000331	476	494	462	485
ENSCAFG00845002992	1233	1205	1008	1077
ENSCAFG00845024302	2	3	6	4
ENSCAFG00845026963	4886	4710	4613	5063
ENSCAFG00845002991	0	0	0	0
ENSCAFG00845002990	0	0	2	0
ENSCAFG00845026960	8	8	0	5
ENSCAFG00845012329	165	105	163	195
ENSCAFG00845012328	0	0	0	0
ENSCAFG00845012325	0	0	0	1
ENSCAFG00845014988	0	0	0	0
ENSCAFG00845012324	0	0	0	0
ENSCAFG00845014989	0	0	0	0
ENSCAFG00845012327	1135	1081	1156	1067
ENSCAFG00845014986	0	0	0	0
ENSCAFG00845012326	40	30	33	25
ENSCAFG00845014987	0	0	0	0
ENSCAFG00845012321	165	209	179	191
ENSCAFG00845014984	0	0	0	0

ENSCAFG00845012320	0	0	0	0
ENSCAFG00845014985	0	0	0	0
ENSCAFG00845012323	0	0	0	0
ENSCAFG00845014982	57	33	38	50
ENSCAFG00845012322	0	0	0	3
ENSCAFG00845014983	0	0	0	0
ENSCAFG00845014970	0	0	0	0
ENSCAFG00845026959	0	0	0	0
ENSCAFG00845000349	2	1	3	1
ENSCAFG00845000347	0	0	0	2
ENSCAFG00845026955	0	6	1	0
ENSCAFG00845000348	282	267	295	288
ENSCAFG00845026954	6640	6536	6658	6927
ENSCAFG00845000345	0	0	0	0
ENSCAFG00845026957	2	2	1	0
ENSCAFG00845000346	0	0	0	0
ENSCAFG00845026956	0	0	0	0
ENSCAFG00845000343	0	0	0	0
ENSCAFG00845026951	2053	1885	1899	1880
ENSCAFG00845000344	455	422	398	400
ENSCAFG00845026950	0	0	0	0
ENSCAFG00845000341	851	792	696	768
ENSCAFG00845026953	0	0	0	0
ENSCAFG00845000342	0	0	0	0
ENSCAFG00845026952	0	0	3	0
ENSCAFG00845000340	83	53	60	56
ENSCAFG00845012318	0	0	0	0
ENSCAFG00845012317	20	17	10	11
ENSCAFG00845014979	1	0	0	0
ENSCAFG00845012319	0	0	0	0
ENSCAFG00845012314	49	34	62	53
ENSCAFG00845014977	0	0	0	0
ENSCAFG00845012313	2	3	1	0
ENSCAFG00845014978	0	0	0	0
ENSCAFG00845012316	0	0	0	0
ENSCAFG00845014975	837	811	814	803
ENSCAFG00845012315	1034	984	885	970
ENSCAFG00845014976	0	0	0	1
ENSCAFG00845012310	0	0	0	0
ENSCAFG00845014973	0	0	0	0
ENSCAFG00845014974	0	0	0	0

ENSCAFG00845012312	0	0	0	0
ENSCAFG00845014971	0	0	0	0
ENSCAFG00845012311	196	173	199	196
ENSCAFG00845014972	3	3	1	6
ENSCAFG00845000318	32	30	46	36
ENSCAFG00845026948	437	409	373	465
ENSCAFG00845000319	13	11	9	5
ENSCAFG00845026947	6	2	4	3
ENSCAFG00845000316	30	18	15	23
ENSCAFG00845002979	0	0	0	0
ENSCAFG00845000317	0	0	0	0
ENSCAFG00845002978	0	0	0	0
ENSCAFG00845026949	0	0	0	0
ENSCAFG00845000314	0	0	0	0
ENSCAFG00845002977	0	0	0	0
ENSCAFG00845026944	0	0	0	0
ENSCAFG00845000315	668	673	653	652
ENSCAFG00845002976	0	0	0	0
ENSCAFG00845026943	6	4	2	3
ENSCAFG00845000312	1	1	0	0
ENSCAFG00845002975	2	1	2	0
ENSCAFG00845026946	35	27	30	38
ENSCAFG00845000313	0	0	0	0
ENSCAFG00845002974	0	0	0	0
ENSCAFG00845026945	0	0	0	0
ENSCAFG00845000310	27	46	36	24
ENSCAFG00845002973	2007	1946	2104	2231
ENSCAFG00845026940	0	0	0	0
ENSCAFG00845000311	0	0	0	0
ENSCAFG00845002972	6347	6025	6091	6348
ENSCAFG00845002971	1163	1109	1056	990
ENSCAFG00845026942	5	10	14	5
ENSCAFG00845002970	30	25	15	12
ENSCAFG00845026941	0	0	0	0
ENSCAFG00845012307	0	0	0	0
ENSCAFG00845012306	41	56	36	50
ENSCAFG00845012309	178	140	157	185
ENSCAFG00845014968	0	0	0	0
ENSCAFG00845012308	0	0	0	0
ENSCAFG00845014969	2082	1957	1925	1928
ENSCAFG00845012303	633	559	537	574

ENSCAFG00845014966	7	2	1	6
ENSCAFG00845012302	1699	1645	1508	1576
ENSCAFG00845014967	1600	1451	1356	1486
ENSCAFG00845012305	0	0	0	0
ENSCAFG00845014964	407	352	704	713
ENSCAFG00845012304	1760	1752	1490	1562
ENSCAFG00845014965	0	0	0	0
ENSCAFG00845014962	0	0	1	0
ENSCAFG00845014963	2	5	3	4
ENSCAFG00845012301	0	1	0	0
ENSCAFG00845014960	0	0	0	0
ENSCAFG00845012300	0	0	0	0
ENSCAFG00845014961	0	1	0	3
ENSCAFG00845000329	418	395	327	372
ENSCAFG00845026937	0	0	0	0
ENSCAFG00845026936	491	437	366	382
ENSCAFG00845000327	486	449	448	413
ENSCAFG00845026939	0	0	0	0
ENSCAFG00845000328	162	142	173	157
ENSCAFG00845002989	2370	2509	2625	2584
ENSCAFG00845026938	1479	1389	1370	1320
ENSCAFG00845000325	0	0	0	0
ENSCAFG00845002988	0	0	0	0
ENSCAFG00845026933	375	334	317	309
ENSCAFG00845000326	7	4	6	5
ENSCAFG00845002987	0	0	0	0
ENSCAFG00845026932	0	0	0	0
ENSCAFG00845000323	817	819	796	739
ENSCAFG00845002986	661	648	618	629
ENSCAFG00845026935	998	997	908	989
ENSCAFG00845000324	0	0	0	0
ENSCAFG00845002985	521	582	508	539
ENSCAFG00845026934	963	964	1091	1101
ENSCAFG00845000321	1640	1589	1588	1731
ENSCAFG00845002984	903	868	889	954
ENSCAFG00845000322	0	0	0	0
ENSCAFG00845002983	2	2	2	1
ENSCAFG00845002982	4	7	8	3
ENSCAFG00845026931	1	1	0	1
ENSCAFG00845000320	21	22	34	16
ENSCAFG00845002981	0	0	0	0

ENSCAFG00845026930	923	813	849	1052
ENSCAFG00845002980	0	0	3	1
ENSCAFG00845014959	277	291	256	248
ENSCAFG00845014957	0	0	0	0
ENSCAFG00845014958	1123	1045	1178	1115
ENSCAFG00845014955	2	1	3	7
ENSCAFG00845014956	19	33	16	31
ENSCAFG00845014953	0	0	0	0
ENSCAFG00845014954	0	0	0	0
ENSCAFG00845014951	347	291	309	320
ENSCAFG00845014952	5157	4926	5110	5382
ENSCAFG00845014950	248	243	216	196
ENSCAFG00845026929	1347	1308	1257	1283
ENSCAFG00845002959	7	15	5	4
ENSCAFG00845026926	458	490	529	482
ENSCAFG00845002958	0	0	0	0
ENSCAFG00845026925	0	0	0	0
ENSCAFG00845002957	0	0	0	0
ENSCAFG00845026928	868	856	947	991
ENSCAFG00845002956	78	79	43	69
ENSCAFG00845002955	518	496	481	430
ENSCAFG00845026922	20	14	23	18
ENSCAFG00845002954	0	0	0	0
ENSCAFG00845026921	0	0	0	0
ENSCAFG00845002953	189	205	201	192
ENSCAFG00845026924	485	496	498	479
ENSCAFG00845002952	0	0	0	0
ENSCAFG00845026923	2005	1833	1847	2029
ENSCAFG00845002951	3323	3264	3062	3069
ENSCAFG00845002950	0	0	0	0
ENSCAFG00845026920	0	0	0	0
ENSCAFG00845014948	0	0	0	0
ENSCAFG00845014949	0	0	0	0
ENSCAFG00845014946	0	0	0	0
ENSCAFG00845014947	0	0	0	0
ENSCAFG00845014944	325	303	330	334
ENSCAFG00845014945	0	0	0	0
ENSCAFG00845014942	1	1	4	4
ENSCAFG00845014943	60	37	36	30
ENSCAFG00845014940	0	0	0	0
ENSCAFG00845014941	0	0	0	0

ENSCAFG00845026919	2476	2437	2588	2634
ENSCAFG00845026918	447	426	400	365
ENSCAFG00845000309	0	0	0	0
ENSCAFG00845000307	315	257	271	299
ENSCAFG00845026915	0	0	0	0
ENSCAFG00845000308	310	318	316	274
ENSCAFG00845002969	0	0	0	0
ENSCAFG00845026914	87	86	95	85
ENSCAFG00845000305	0	0	0	1
ENSCAFG00845002968	1736	1706	1727	1712
ENSCAFG00845026917	5	7	11	4
ENSCAFG00845000306	0	0	0	0
ENSCAFG00845002967	264	238	300	291
ENSCAFG00845026916	8064	7748	8037	8244
ENSCAFG00845000303	0	0	0	0
ENSCAFG00845002966	181	194	185	202
ENSCAFG00845026911	29	31	22	31
ENSCAFG00845000304	231	201	209	196
ENSCAFG00845002965	0	0	0	0
ENSCAFG00845026910	8853	8704	8551	8838
ENSCAFG00845000301	0	0	0	0
ENSCAFG00845002964	2	2	1	3
ENSCAFG00845026913	1	0	0	0
ENSCAFG00845000302	20	10	9	8
ENSCAFG00845002963	0	0	0	0
ENSCAFG00845026912	1069	1160	1094	1125
ENSCAFG00845002962	263	235	254	305
ENSCAFG00845000300	0	0	0	0
ENSCAFG00845002961	7	12	11	12
ENSCAFG00845002960	0	0	0	0
ENSCAFG00845014939	0	0	0	0
ENSCAFG00845014937	0	0	0	0
ENSCAFG00845014938	0	0	0	0
ENSCAFG00845014935	0	0	0	0
ENSCAFG00845014936	0	0	0	0
ENSCAFG00845014933	0	0	0	0
ENSCAFG00845014934	2	6	2	5
ENSCAFG00845014931	1699	1529	1659	1695
ENSCAFG00845014932	0	0	0	0
ENSCAFG00845014930	3	6	2	9
ENSCAFG00845024398	670	593	652	706

ENSCAFG00845024399	25	7	16	18
ENSCAFG00845024394	2	2	4	2
ENSCAFG00845024396	0	0	0	0
ENSCAFG00845024397	0	0	0	0
ENSCAFG00845024390	0	0	0	0
ENSCAFG00845024391	1085	1042	1050	1030
ENSCAFG00845024392	0	0	0	0
ENSCAFG00845024393	2	3	2	3
ENSCAFG00845024387	0	0	0	2
ENSCAFG00845024388	1439	1311	1425	1413
ENSCAFG00845024389	0	0	0	0
ENSCAFG00845024383	0	0	0	0
ENSCAFG00845024384	0	0	0	0
ENSCAFG00845024385	2	1	1	0
ENSCAFG00845024386	491	455	559	545
ENSCAFG00845024380	3	1	0	2
ENSCAFG00845024382	0	0	0	0
ENSCAFG00845012394	2	2	1	0
ENSCAFG00845012393	163	203	153	197
ENSCAFG00845012396	0	0	0	0
ENSCAFG00845012395	0	0	0	0
ENSCAFG00845012390	0	0	0	0
ENSCAFG00845012392	0	0	0	0
ENSCAFG00845012391	94	81	91	55
ENSCAFG00845024376	34	18	29	36
ENSCAFG00845024377	649	626	674	655
ENSCAFG00845024378	322	332	343	327
ENSCAFG00845024379	0	0	0	0
ENSCAFG00845024372	0	0	0	0
ENSCAFG00845024373	0	0	0	0
ENSCAFG00845024374	0	0	0	0
ENSCAFG00845024375	118	98	68	62
ENSCAFG00845024370	5	4	7	9
ENSCAFG00845024371	0	0	0	0
ENSCAFG00845012398	19	16	15	22
ENSCAFG00845012397	52	43	69	66
ENSCAFG00845012399	0	0	0	0
ENSCAFG00845012383	7067	6736	6761	6563
ENSCAFG00845012382	2002	2059	1890	1905
ENSCAFG00845012385	0	0	0	0
ENSCAFG00845012384	7431	7188	6753	6602

ENSCAFG00845012381	8	19	22	14
ENSCAFG00845012380	0	0	0	2
ENSCAFG00845024369	0	0	0	0
ENSCAFG00845000398	1995	1885	1855	1818
ENSCAFG00845024365	0	0	0	0
ENSCAFG00845000399	0	0	1	0
ENSCAFG00845024366	3165	3027	3084	3027
ENSCAFG00845000396	0	0	0	0
ENSCAFG00845024367	220	197	236	248
ENSCAFG00845000397	0	0	0	0
ENSCAFG00845024368	2	0	0	1
ENSCAFG00845000394	739	705	726	660
ENSCAFG00845024361	485	550	504	547
ENSCAFG00845000395	392	372	400	420
ENSCAFG00845024362	0	0	0	0
ENSCAFG00845000392	704	658	721	760
ENSCAFG00845024363	2386	2284	2223	2315
ENSCAFG00845000393	1	2	1	3
ENSCAFG00845024364	36	25	40	33
ENSCAFG00845000390	331	336	451	462
ENSCAFG00845000391	478	435	399	475
ENSCAFG00845024360	938	916	813	892
ENSCAFG00845012387	0	0	0	0
ENSCAFG00845012386	88	53	64	57
ENSCAFG00845012389	7	15	7	8
ENSCAFG00845012388	1417	1282	1317	1355
ENSCAFG00845012372	4	3	3	1
ENSCAFG00845012371	3469	3187	3573	3396
ENSCAFG00845012374	32	21	44	31
ENSCAFG00845012373	1158	1030	1093	1009
ENSCAFG00845012370	0	0	0	0
ENSCAFG00845024358	0	0	0	0
ENSCAFG00845024359	266	284	279	288
ENSCAFG00845024354	0	0	0	0
ENSCAFG00845024355	0	0	0	0
ENSCAFG00845024356	646	685	586	602
ENSCAFG00845024357	712	741	654	770
ENSCAFG00845024350	344	346	251	195
ENSCAFG00845024352	1391	1379	1222	1207
ENSCAFG00845024353	0	0	0	0
ENSCAFG00845012379	0	0	0	0

ENSCAFG00845012376	0	0	0	0
ENSCAFG00845012375	202	238	205	253
ENSCAFG00845012378	0	0	0	0
ENSCAFG00845012377	1	4	0	1
ENSCAFG00845012361	0	0	0	0
ENSCAFG00845012360	0	0	0	0
ENSCAFG00845012363	1468	1468	1490	1404
ENSCAFG00845012362	301	298	286	322
ENSCAFG00845024347	1376	1416	1508	1419
ENSCAFG00845024348	0	0	0	0
ENSCAFG00845000378	1	0	0	0
ENSCAFG00845024349	7	0	7	2
ENSCAFG00845000379	437	446	390	370
ENSCAFG00845000376	0	0	0	0
ENSCAFG00845024343	1717	1672	1543	1464
ENSCAFG00845000377	0	0	7	0
ENSCAFG00845024344	0	2	0	0
ENSCAFG00845000374	0	0	0	0
ENSCAFG00845024345	2	0	0	0
ENSCAFG00845000375	0	0	0	1
ENSCAFG00845024346	148	137	154	159
ENSCAFG00845000372	3224	3066	3010	3101
ENSCAFG00845000373	1	4	2	6
ENSCAFG00845024340	41	40	54	38
ENSCAFG00845000370	29	15	20	19
ENSCAFG00845024341	0	0	0	0
ENSCAFG00845000371	543	525	534	512
ENSCAFG00845024342	0	0	0	0
ENSCAFG00845012369	992	992	1180	1142
ENSCAFG00845012368	0	0	0	0
ENSCAFG00845012365	6	11	7	12
ENSCAFG00845012364	540	517	578	589
ENSCAFG00845012367	795	732	792	866
ENSCAFG00845012366	21	16	31	28
ENSCAFG00845012350	0	0	0	0
ENSCAFG00845012352	0	0	0	0
ENSCAFG00845012351	1	1	0	0
ENSCAFG00845024336	3151	2872	3024	3060
ENSCAFG00845026999	291	272	271	263
ENSCAFG00845024337	0	0	1	0
ENSCAFG00845026998	0	0	0	0

ENSCAFG00845000389	761	840	768	759
ENSCAFG00845024338	1102	1038	1066	1067
ENSCAFG00845024339	505	461	446	473
ENSCAFG00845000387	25	17	15	25
ENSCAFG00845024332	719	725	745	689
ENSCAFG00845026995	0	0	2	2
ENSCAFG00845000388	1	2	4	1
ENSCAFG00845024333	1454	1553	1576	1537
ENSCAFG00845026994	2	1	0	0
ENSCAFG00845000385	0	0	0	0
ENSCAFG00845024334	439	379	447	405
ENSCAFG00845026997	229	250	246	197
ENSCAFG00845000386	3	0	0	2
ENSCAFG00845024335	291	304	326	321
ENSCAFG00845026996	0	0	0	0
ENSCAFG00845000383	2	6	5	4
ENSCAFG00845026991	0	0	0	0
ENSCAFG00845000384	5395	4914	5160	5569
ENSCAFG00845026990	62	76	69	56
ENSCAFG00845000381	18	16	9	6
ENSCAFG00845024330	1	1	0	1
ENSCAFG00845026993	0	0	0	0
ENSCAFG00845000382	359	367	399	349
ENSCAFG00845024331	0	1	4	7
ENSCAFG00845026992	613	567	579	628
ENSCAFG00845000380	0	0	0	0
ENSCAFG00845012358	0	1	0	0
ENSCAFG00845012357	356	370	361	364
ENSCAFG00845012359	0	4	0	0
ENSCAFG00845012354	1262	1215	1244	1246
ENSCAFG00845012353	14	13	14	23
ENSCAFG00845012356	0	0	0	0
ENSCAFG00845012355	4	7	4	5
ENSCAFG00845026298	29	29	18	34
ENSCAFG00845026297	0	0	0	0
ENSCAFG00845026299	0	0	0	0
ENSCAFG00845026294	0	0	0	0
ENSCAFG00845026293	0	0	0	0
ENSCAFG00845026296	75	64	72	71
ENSCAFG00845026295	0	0	0	0
ENSCAFG00845026290	15	17	11	23

ENSCAFG00845026292	0	0	0	0
ENSCAFG00845026291	0	0	0	0
ENSCAFG00845026287	0	0	0	0
ENSCAFG00845026289	2483	2565	2637	2730
ENSCAFG00845026288	71	72	69	62
ENSCAFG00845026283	0	0	0	5
ENSCAFG00845026282	421	400	497	427
ENSCAFG00845026285	460	457	496	491
ENSCAFG00845026284	0	0	0	0
ENSCAFG00845026281	1	3	0	1
ENSCAFG00845026280	0	0	0	0
ENSCAFG00845014298	1439	1323	1298	1340
ENSCAFG00845014299	355	316	284	388
ENSCAFG00845014296	6	7	18	12
ENSCAFG00845014297	0	0	0	0
ENSCAFG00845014294	53	53	59	47
ENSCAFG00845014295	5	7	17	15
ENSCAFG00845014292	1	1	0	0
ENSCAFG00845014293	0	0	0	0
ENSCAFG00845014290	511	553	649	678
ENSCAFG00845014291	564	497	502	487
ENSCAFG00845026279	0	0	0	0
ENSCAFG00845026276	0	0	0	0
ENSCAFG00845026278	0	0	0	0
ENSCAFG00845026277	3	0	2	0
ENSCAFG00845026272	0	0	0	0
ENSCAFG00845026271	198	181	242	196
ENSCAFG00845026274	0	0	1	0
ENSCAFG00845026273	0	0	0	0
ENSCAFG00845026270	1872	1820	1928	2085
ENSCAFG00845014287	8474	8311	7328	7158
ENSCAFG00845014288	0	0	0	2
ENSCAFG00845014285	11	20	5	9
ENSCAFG00845014286	927	924	882	833
ENSCAFG00845014283	4	3	3	5
ENSCAFG00845014284	297	311	317	299
ENSCAFG00845014281	144	115	130	121
ENSCAFG00845014282	8	5	6	9
ENSCAFG00845014280	1347	1351	1358	1157
ENSCAFG00845026269	0	0	0	0
ENSCAFG00845026268	0	0	0	0

ENSCAFG00845002299	0	0	0	0
ENSCAFG00845002298	0	0	0	0
ENSCAFG00845026265	0	0	0	0
ENSCAFG00845002297	0	0	0	0
ENSCAFG00845026264	0	0	0	0
ENSCAFG00845002296	0	0	0	0
ENSCAFG00845026267	7	6	5	2
ENSCAFG00845002295	449	406	383	400
ENSCAFG00845026266	419	387	424	463
ENSCAFG00845002294	9393	9692	8259	7998
ENSCAFG00845026261	147	162	128	127
ENSCAFG00845002293	0	0	3	0
ENSCAFG00845026260	0	1	0	2
ENSCAFG00845002292	174	200	161	152
ENSCAFG00845026263	0	0	0	0
ENSCAFG00845002291	16	20	15	22
ENSCAFG00845026262	0	2	1	2
ENSCAFG00845002290	0	0	0	0
ENSCAFG00845014289	1503	1401	1534	1545
ENSCAFG00845014276	0	2	3	2
ENSCAFG00845014277	0	0	0	0
ENSCAFG00845014274	0	0	0	0
ENSCAFG00845014275	14	6	11	21
ENSCAFG00845014272	1166	1196	1084	1132
ENSCAFG00845014273	196	208	262	247
ENSCAFG00845014270	0	0	0	0
ENSCAFG00845014271	5	3	11	10
ENSCAFG00845026258	0	0	0	0
ENSCAFG00845026257	1	0	0	0
ENSCAFG00845026259	1466	1408	1294	1318
ENSCAFG00845026254	0	0	0	0
ENSCAFG00845026253	0	0	0	0
ENSCAFG00845026256	4	4	1	3
ENSCAFG00845026255	4681	4569	4083	4078
ENSCAFG00845026250	0	0	0	0
ENSCAFG00845026252	0	0	0	0
ENSCAFG00845026251	17	7	18	22
ENSCAFG00845014278	404	393	329	363
ENSCAFG00845014279	3747	3659	3307	3281
ENSCAFG00845014265	0	0	0	0
ENSCAFG00845014266	1729	1628	1389	1429

ENSCAFG00845014263	3	0	0	0
ENSCAFG00845014264	0	0	4	6
ENSCAFG00845014261	0	0	0	0
ENSCAFG00845014262	0	0	0	0
ENSCAFG00845014260	2	4	0	0
ENSCAFG00845026247	0	0	0	0
ENSCAFG00845002279	2	7	6	11
ENSCAFG00845026246	0	0	0	0
ENSCAFG00845002278	0	0	0	0
ENSCAFG00845002277	17	22	13	28
ENSCAFG00845026248	0	0	0	0
ENSCAFG00845002276	0	0	0	0
ENSCAFG00845026243	0	0	0	0
ENSCAFG00845002275	626	589	601	637
ENSCAFG00845026242	0	0	0	0
ENSCAFG00845002274	1	2	5	0
ENSCAFG00845026245	340	297	273	308
ENSCAFG00845002273	4	1	2	0
ENSCAFG00845026244	0	0	0	0
ENSCAFG00845002272	0	0	0	0
ENSCAFG00845002271	459	472	526	472
ENSCAFG00845002270	594	575	594	551
ENSCAFG00845026241	4	3	4	8
ENSCAFG00845026240	0	0	0	0
ENSCAFG00845014269	24	28	31	15
ENSCAFG00845014267	0	0	0	0
ENSCAFG00845014268	0	0	0	0
ENSCAFG00845014254	0	0	0	0
ENSCAFG00845014255	0	0	0	0
ENSCAFG00845014252	0	0	0	0
ENSCAFG00845014253	675	647	613	612
ENSCAFG00845014250	1	1	6	2
ENSCAFG00845014251	1618	1546	1543	1512
ENSCAFG00845026236	843	748	788	918
ENSCAFG00845028899	278	244	258	255
ENSCAFG00845026235	0	2	2	5
ENSCAFG00845002289	183	175	97	145
ENSCAFG00845026238	0	0	0	0
ENSCAFG00845028897	158	167	131	166
ENSCAFG00845002288	7198	7136	7666	7558
ENSCAFG00845026237	16	10	11	5

ENSCAFG00845028898	1	0	0	0
ENSCAFG00845002287	56	82	57	63
ENSCAFG00845026232	1161	1038	943	949
ENSCAFG00845028895	0	4	5	2
ENSCAFG00845002286	0	0	0	0
ENSCAFG00845028896	76	72	81	76
ENSCAFG00845002285	1098	981	986	1078
ENSCAFG00845026234	0	0	0	0
ENSCAFG00845028893	3	1	3	5
ENSCAFG00845002284	0	2	2	2
ENSCAFG00845026233	469	492	292	315
ENSCAFG00845028894	0	0	0	0
ENSCAFG00845002283	3	2	0	1
ENSCAFG00845028891	1	0	0	0
ENSCAFG00845002282	1174	1182	1105	1145
ENSCAFG00845028892	3090	2856	3280	3217
ENSCAFG00845002281	0	4	3	2
ENSCAFG00845026230	0	0	0	0
ENSCAFG00845002280	1	1	0	0
ENSCAFG00845028890	469	440	430	381
ENSCAFG00845014258	0	2	1	6
ENSCAFG00845014259	0	0	0	0
ENSCAFG00845014256	14	4	4	5
ENSCAFG00845014257	0	0	1	0
ENSCAFG00845028808	1	0	0	0
ENSCAFG00845028809	3878	3701	4604	4490
ENSCAFG00845028806	517	515	528	486
ENSCAFG00845028807	0	0	0	0
ENSCAFG00845028804	232	232	221	243
ENSCAFG00845028805	68	36	59	67
ENSCAFG00845028802	0	0	0	0
ENSCAFG00845028803	755	673	715	765
ENSCAFG00845028800	0	0	0	0
ENSCAFG00845028801	154	140	166	192
ENSCAFG00845004840	792	743	722	738
ENSCAFG00845004841	0	0	0	0
ENSCAFG00845004842	0	0	0	0
ENSCAFG00845004843	45	31	41	40
ENSCAFG00845004844	0	0	0	0
ENSCAFG00845004845	0	0	0	0
ENSCAFG00845016829	0	0	0	0

ENSCAFG00845004846	267	298	279	382
ENSCAFG00845004847	52	45	40	69
ENSCAFG00845016827	88	66	74	106
ENSCAFG00845004848	226	262	238	249
ENSCAFG00845016826	0	0	0	0
ENSCAFG00845004849	403	409	480	451
ENSCAFG00845016825	349	364	361	295
ENSCAFG00845016824	0	1	1	0
ENSCAFG00845016823	139	108	102	139
ENSCAFG00845016822	90	57	61	85
ENSCAFG00845016821	402	413	399	409
ENSCAFG00845016820	0	1	3	1
ENSCAFG00845004830	0	0	1	1
ENSCAFG00845004831	2500	2459	2287	2440
ENSCAFG00845004832	11	9	10	5
ENSCAFG00845004833	864	861	829	855
ENSCAFG00845016819	0	0	0	0
ENSCAFG00845004834	742	681	752	878
ENSCAFG00845016818	1	4	0	3
ENSCAFG00845004835	4	4	4	4
ENSCAFG00845016817	0	0	0	0
ENSCAFG00845004836	1	0	0	0
ENSCAFG00845016816	5187	4931	5135	5409
ENSCAFG00845004837	2	2	0	1
ENSCAFG00845016815	2	1	0	0
ENSCAFG00845004838	0	0	0	0
ENSCAFG00845016814	219	220	164	189
ENSCAFG00845004839	2	0	0	0
ENSCAFG00845016813	11	13	9	14
ENSCAFG00845016812	2	0	1	1
ENSCAFG00845016811	0	1	0	0
ENSCAFG00845016810	18	26	7	22
ENSCAFG00845004820	117	114	163	134
ENSCAFG00845004821	82	120	77	67
ENSCAFG00845016809	0	0	0	0
ENSCAFG00845004822	573	571	566	491
ENSCAFG00845016808	617	535	578	506
ENSCAFG00845004823	27	15	26	26
ENSCAFG00845016807	1772	1664	1683	1802
ENSCAFG00845004824	0	0	0	0
ENSCAFG00845016806	840	717	755	714

ENSCAFG00845004825	14	11	21	25
ENSCAFG00845016805	0	1	0	0
ENSCAFG00845004826	1	1	0	1
ENSCAFG00845016804	1464	1434	1204	1352
ENSCAFG00845004827	74	73	74	84
ENSCAFG00845016803	2	0	0	1
ENSCAFG00845004828	642	630	615	660
ENSCAFG00845016802	0	0	0	0
ENSCAFG00845004829	0	3	3	1
ENSCAFG00845016801	27	43	23	35
ENSCAFG00845016800	0	0	0	0
ENSCAFG00845004810	0	1	0	1
ENSCAFG00845004811	26	26	18	27
ENSCAFG00845004812	0	0	0	0
ENSCAFG00845004813	0	0	0	0
ENSCAFG00845004814	3	6	2	3
ENSCAFG00845004815	382	405	388	382
ENSCAFG00845004816	0	0	0	0
ENSCAFG00845004817	63	78	86	83
ENSCAFG00845004818	1	2	0	5
ENSCAFG00845004819	70	47	52	65
ENSCAFG00845004800	0	0	0	0
ENSCAFG00845004801	676	618	698	721
ENSCAFG00845004802	0	1	0	2
ENSCAFG00845004803	528	491	527	566
ENSCAFG00845004804	189	206	171	192
ENSCAFG00845004805	387	387	464	426
ENSCAFG00845004806	1763	1790	2007	2080
ENSCAFG00845004807	1059	1006	1053	1063
ENSCAFG00845004808	375	398	357	429
ENSCAFG00845004809	381	340	410	354
ENSCAFG00845014243	272	242	266	306
ENSCAFG00845014244	3971	3885	4348	4467
ENSCAFG00845014241	0	0	0	0
ENSCAFG00845014242	0	0	0	0
ENSCAFG00845014240	2293	2137	1847	1864
ENSCAFG00845026229	0	0	0	0
ENSCAFG00845026228	0	0	0	0
ENSCAFG00845002259	13	13	45	33
ENSCAFG00845002258	240	224	222	210
ENSCAFG00845026225	0	0	0	1

ENSCAFG00845028888	10	9	9	7
ENSCAFG00845002257	1544	1452	1381	1449
ENSCAFG00845026224	4	7	8	5
ENSCAFG00845028889	222	246	218	219
ENSCAFG00845002256	102	85	89	77
ENSCAFG00845026227	0	0	0	0
ENSCAFG00845028886	0	0	0	0
ENSCAFG00845002255	148	112	115	109
ENSCAFG00845028887	2	6	9	3
ENSCAFG00845002254	0	0	0	0
ENSCAFG00845026221	585	593	513	634
ENSCAFG00845028884	11	9	6	5
ENSCAFG00845002253	1821	1829	1599	1679
ENSCAFG00845026220	3006	3145	2907	2822
ENSCAFG00845028885	0	3	2	0
ENSCAFG00845002252	156	120	114	124
ENSCAFG00845026223	0	0	0	0
ENSCAFG00845028882	0	0	0	0
ENSCAFG00845002251	1573	1611	1096	1165
ENSCAFG00845026222	1	0	0	0
ENSCAFG00845028883	0	0	0	1
ENSCAFG00845002250	0	0	0	1
ENSCAFG00845028880	7	5	2	3
ENSCAFG00845028881	0	0	0	0
ENSCAFG00845014249	0	0	0	0
ENSCAFG00845014247	2993	2821	1118	1129
ENSCAFG00845014248	798	801	715	785
ENSCAFG00845014245	865	782	1427	1523
ENSCAFG00845014246	1002	964	829	777
ENSCAFG00845014232	276	295	112	113
ENSCAFG00845016895	0	0	0	0
ENSCAFG00845014233	2405	2371	2308	2464
ENSCAFG00845016894	7	0	0	8
ENSCAFG00845014230	60	68	75	58
ENSCAFG00845016893	473	540	516	528
ENSCAFG00845014231	32	34	44	45
ENSCAFG00845016892	2	0	1	2
ENSCAFG00845016891	109	148	117	134
ENSCAFG00845016890	0	0	0	0
ENSCAFG00845026218	762	837	766	774
ENSCAFG00845026217	2	2	0	2

ENSCAFG00845028879	5	3	0	3
ENSCAFG00845026219	0	0	0	0
ENSCAFG00845002269	1548	1470	1414	1566
ENSCAFG00845026214	0	0	0	0
ENSCAFG00845028877	563	585	527	570
ENSCAFG00845002268	0	2	1	2
ENSCAFG00845026213	0	0	0	0
ENSCAFG00845028878	0	0	0	0
ENSCAFG00845002267	901	865	600	616
ENSCAFG00845026216	259	242	246	296
ENSCAFG00845028875	3562	3452	3591	3534
ENSCAFG00845002266	268	285	288	250
ENSCAFG00845028876	2	0	1	0
ENSCAFG00845002265	9	31	15	24
ENSCAFG00845026210	0	0	0	0
ENSCAFG00845028873	0	0	0	0
ENSCAFG00845002264	0	4	0	0
ENSCAFG00845028874	2	0	0	0
ENSCAFG00845002263	3935	3739	3487	3530
ENSCAFG00845026212	1	2	1	0
ENSCAFG00845028871	509	423	477	498
ENSCAFG00845002262	3	2	0	1
ENSCAFG00845026211	0	0	0	0
ENSCAFG00845028872	2322	2260	2260	2369
ENSCAFG00845002261	0	0	0	0
ENSCAFG00845002260	968	975	1027	1057
ENSCAFG00845028870	49	37	62	67
ENSCAFG00845014238	884	760	773	833
ENSCAFG00845014239	553	541	446	464
ENSCAFG00845014236	3	6	2	2
ENSCAFG00845016899	0	0	0	0
ENSCAFG00845014237	387	447	395	393
ENSCAFG00845016898	9	6	9	4
ENSCAFG00845014234	2206	2124	2194	2135
ENSCAFG00845016897	4	5	19	11
ENSCAFG00845014235	1608	1589	1507	1538
ENSCAFG00845016896	0	0	0	0
ENSCAFG00845014221	0	0	0	0
ENSCAFG00845016884	13	2	1	6
ENSCAFG00845014222	0	0	0	0
ENSCAFG00845016883	0	0	0	0

ENSCAFG00845016882	0	0	0	0
ENSCAFG00845014220	1030	965	887	1006
ENSCAFG00845016881	17	12	2	16
ENSCAFG00845016880	0	0	0	0
ENSCAFG00845026207	4	2	0	1
ENSCAFG00845002239	0	1	2	1
ENSCAFG00845026206	0	0	0	0
ENSCAFG00845002238	0	0	0	0
ENSCAFG00845026209	0	0	0	0
ENSCAFG00845028868	0	0	0	3
ENSCAFG00845002237	4	7	12	13
ENSCAFG00845028869	292	283	298	277
ENSCAFG00845002236	945	898	939	1009
ENSCAFG00845026203	519	514	606	562
ENSCAFG00845028866	6	3	2	1
ENSCAFG00845002235	4	1	0	1
ENSCAFG00845026202	21	33	41	18
ENSCAFG00845028867	257	183	210	288
ENSCAFG00845002234	6	1	7	4
ENSCAFG00845026205	0	0	0	0
ENSCAFG00845028864	0	0	0	0
ENSCAFG00845002233	927	902	862	926
ENSCAFG00845026204	0	0	0	0
ENSCAFG00845028865	1	2	0	0
ENSCAFG00845002232	6	2	10	2
ENSCAFG00845028862	9	2	2	1
ENSCAFG00845002231	7	10	9	13
ENSCAFG00845028863	0	0	0	0
ENSCAFG00845002230	0	0	0	0
ENSCAFG00845026201	0	0	0	0
ENSCAFG00845028860	21	17	10	14
ENSCAFG00845026200	1024	1017	1021	1014
ENSCAFG00845028861	63	62	72	102
ENSCAFG00845014229	14	17	8	7
ENSCAFG00845014227	1	0	1	1
ENSCAFG00845014228	0	0	0	0
ENSCAFG00845016889	6520	6366	6293	6747
ENSCAFG00845014225	1570	1655	1474	1571
ENSCAFG00845016888	0	0	0	0
ENSCAFG00845014226	2557	2469	2546	2428
ENSCAFG00845016887	0	0	0	0

ENSCAFG00845014223	3	1	4	2
ENSCAFG00845016886	725	693	690	721
ENSCAFG00845014224	43	27	26	19
ENSCAFG00845016885	0	0	0	0
ENSCAFG00845014210	143	173	141	204
ENSCAFG00845016873	677	743	654	634
ENSCAFG00845014211	0	2	4	0
ENSCAFG00845016872	857	814	751	704
ENSCAFG00845016871	0	0	0	2
ENSCAFG00845016870	0	0	0	0
ENSCAFG00845028859	169	178	167	132
ENSCAFG00845002249	222	239	175	149
ENSCAFG00845028857	14	11	13	7
ENSCAFG00845002248	369	300	282	304
ENSCAFG00845004890	11	6	8	7
ENSCAFG00845028858	2	3	0	0
ENSCAFG00845002247	936	965	870	887
ENSCAFG00845004891	120	106	111	104
ENSCAFG00845028855	54	63	100	75
ENSCAFG00845002246	195	215	178	188
ENSCAFG00845004892	0	0	0	0
ENSCAFG00845028856	1613	1626	1571	1669
ENSCAFG00845002245	7	1	4	8
ENSCAFG00845004893	70	54	66	59
ENSCAFG00845028853	278	306	242	308
ENSCAFG00845002244	264	252	230	236
ENSCAFG00845004894	41	21	43	27
ENSCAFG00845028854	1668	1588	1836	2013
ENSCAFG00845002243	231	197	242	219
ENSCAFG00845004895	527	537	498	505
ENSCAFG00845028851	238	223	178	188
ENSCAFG00845002242	5	0	2	2
ENSCAFG00845004896	225	200	408	342
ENSCAFG00845028852	9	15	14	15
ENSCAFG00845002241	2	0	2	2
ENSCAFG00845004897	0	0	0	4
ENSCAFG00845002240	3	5	1	1
ENSCAFG00845004898	1503	1423	1202	1243
ENSCAFG00845028850	120	112	143	150
ENSCAFG00845004899	0	0	0	0
ENSCAFG00845014218	10	9	20	11

ENSCAFG00845014219	5	1	7	4
ENSCAFG00845014216	22	25	32	30
ENSCAFG00845016879	192	179	189	176
ENSCAFG00845014217	0	0	0	0
ENSCAFG00845016878	0	0	0	0
ENSCAFG00845014214	591	505	562	589
ENSCAFG00845016877	16	18	12	14
ENSCAFG00845014215	0	0	0	0
ENSCAFG00845016876	0	0	0	1
ENSCAFG00845014212	1	0	1	0
ENSCAFG00845016875	323	347	309	355
ENSCAFG00845014213	426	378	459	393
ENSCAFG00845016874	140	164	239	222
ENSCAFG00845016862	103	98	81	94
ENSCAFG00845014200	2	0	3	0
ENSCAFG00845016861	0	0	0	0
ENSCAFG00845016860	385	361	278	329
ENSCAFG00845002219	1344	1287	1306	1290
ENSCAFG00845002218	26	28	24	24
ENSCAFG00845028848	0	0	0	2
ENSCAFG00845002217	3081	3041	2966	2994
ENSCAFG00845028849	0	2	0	0
ENSCAFG00845002216	0	0	0	0
ENSCAFG00845028846	0	0	0	1
ENSCAFG00845002215	14	8	15	11
ENSCAFG00845028847	0	0	0	0
ENSCAFG00845002214	1349	1250	1354	1452
ENSCAFG00845004880	1230	1201	1267	1199
ENSCAFG00845028844	0	0	0	0
ENSCAFG00845002213	4	1	1	1
ENSCAFG00845004881	1433	1457	1402	1506
ENSCAFG00845028845	5	7	7	7
ENSCAFG00845002212	23	20	22	8
ENSCAFG00845004882	2	1	2	1
ENSCAFG00845028842	0	0	0	0
ENSCAFG00845002211	910	868	819	1002
ENSCAFG00845004883	186	172	167	172
ENSCAFG00845028843	4	6	5	5
ENSCAFG00845002210	1057	919	1034	1027
ENSCAFG00845004884	0	0	0	0
ENSCAFG00845028840	0	0	0	0

ENSCAFG00845004885	3670	3292	3466	3464
ENSCAFG00845028841	0	0	0	0
ENSCAFG00845004886	0	0	0	1
ENSCAFG00845004887	0	0	0	0
ENSCAFG00845004888	0	0	0	0
ENSCAFG00845004889	3	9	9	8
ENSCAFG00845014209	8	3	9	4
ENSCAFG00845014207	2	8	2	7
ENSCAFG00845014208	958	962	1089	1144
ENSCAFG00845016869	419	409	380	431
ENSCAFG00845014205	1019	911	858	834
ENSCAFG00845016868	0	0	0	0
ENSCAFG00845014206	7	5	2	3
ENSCAFG00845016867	1322	1206	1319	1321
ENSCAFG00845014203	0	0	0	0
ENSCAFG00845016866	247	263	203	205
ENSCAFG00845014204	251	243	232	210
ENSCAFG00845016865	0	0	0	0
ENSCAFG00845014201	152	112	103	103
ENSCAFG00845016864	0	0	0	0
ENSCAFG00845014202	1423	1343	1455	1407
ENSCAFG00845016863	0	0	0	0
ENSCAFG00845016851	0	0	0	0
ENSCAFG00845016850	265	274	286	269
ENSCAFG00845028839	1745	1541	1627	1618
ENSCAFG00845002229	3	11	1	4
ENSCAFG00845028837	4	3	5	3
ENSCAFG00845002228	0	0	0	0
ENSCAFG00845028838	0	0	0	0
ENSCAFG00845002227	28	33	28	26
ENSCAFG00845028835	940	973	987	1029
ENSCAFG00845002226	3	0	0	5
ENSCAFG00845028836	0	0	0	0
ENSCAFG00845002225	1	0	2	2
ENSCAFG00845028833	0	0	0	0
ENSCAFG00845002224	155	146	111	143
ENSCAFG00845004870	20	20	29	29
ENSCAFG00845028834	0	3	2	0
ENSCAFG00845002223	982	1011	908	932
ENSCAFG00845004871	0	0	0	0
ENSCAFG00845028831	0	0	0	0

ENSCAFG00845002222	1	2	2	0
ENSCAFG00845004872	0	0	0	0
ENSCAFG00845028832	887	844	993	950
ENSCAFG00845002221	0	0	3	1
ENSCAFG00845004873	27	11	20	29
ENSCAFG00845002220	382	377	288	302
ENSCAFG00845004874	0	0	0	0
ENSCAFG00845028830	2	0	0	1
ENSCAFG00845004875	1602	1578	3161	3186
ENSCAFG00845004876	1	0	2	2
ENSCAFG00845004877	0	1	0	0
ENSCAFG00845004878	0	0	0	0
ENSCAFG00845004879	449	547	437	436
ENSCAFG00845016859	0	0	0	0
ENSCAFG00845016858	0	0	0	0
ENSCAFG00845016857	0	0	0	2
ENSCAFG00845016856	2	4	0	4
ENSCAFG00845016855	12	15	8	11
ENSCAFG00845016854	0	0	0	0
ENSCAFG00845016853	0	0	0	0
ENSCAFG00845016852	243	220	226	193
ENSCAFG00845016840	386	393	443	415
ENSCAFG00845028828	0	0	1	0
ENSCAFG00845028829	1652	1606	1682	1702
ENSCAFG00845028826	953	917	869	889
ENSCAFG00845028827	0	2	0	0
ENSCAFG00845028824	0	1	1	4
ENSCAFG00845028825	0	0	0	0
ENSCAFG00845028822	0	0	0	0
ENSCAFG00845028823	0	0	0	0
ENSCAFG00845004860	0	0	0	0
ENSCAFG00845028820	175	166	128	126
ENSCAFG00845004861	0	0	0	0
ENSCAFG00845028821	0	0	0	0
ENSCAFG00845004862	5397	5591	6018	6060
ENSCAFG00845004863	1470	1347	1465	1488
ENSCAFG00845004864	12	15	15	12
ENSCAFG00845004865	0	0	0	0
ENSCAFG00845004866	404	437	433	417
ENSCAFG00845004867	32	34	25	22
ENSCAFG00845004868	3	7	6	1

ENSCAFG00845004869	0	0	0	0
ENSCAFG00845016849	16	19	28	18
ENSCAFG00845016848	0	0	0	0
ENSCAFG00845016847	0	1	5	1
ENSCAFG00845016846	0	0	0	0
ENSCAFG00845016845	55	45	41	28
ENSCAFG00845016844	0	0	0	0
ENSCAFG00845016843	0	0	0	0
ENSCAFG00845016842	3159	2940	3565	3538
ENSCAFG00845016841	330	293	325	319
ENSCAFG00845028819	118	105	84	116
ENSCAFG00845002209	1	0	1	0
ENSCAFG00845028817	998	961	897	920
ENSCAFG00845002208	0	0	0	0
ENSCAFG00845028818	0	0	0	0
ENSCAFG00845002207	0	0	0	0
ENSCAFG00845028815	348	342	307	312
ENSCAFG00845002206	7	5	6	5
ENSCAFG00845028816	667	549	551	556
ENSCAFG00845002205	1810	1572	1914	1968
ENSCAFG00845028813	1400	1289	1267	1277
ENSCAFG00845002204	161	159	94	116
ENSCAFG00845028814	689	703	654	681
ENSCAFG00845002203	0	0	0	0
ENSCAFG00845028811	0	0	0	0
ENSCAFG00845002202	1270	1117	1181	1141
ENSCAFG00845028812	0	0	0	0
ENSCAFG00845002201	0	0	0	0
ENSCAFG00845002200	6	6	9	3
ENSCAFG00845004850	81	107	99	86
ENSCAFG00845028810	0	0	0	0
ENSCAFG00845004851	4	5	5	6
ENSCAFG00845004852	0	0	0	0
ENSCAFG00845004853	0	0	0	0
ENSCAFG00845004854	139	154	162	181
ENSCAFG00845004855	0	0	0	0
ENSCAFG00845004856	0	0	0	0
ENSCAFG00845004857	237	212	208	217
ENSCAFG00845016839	0	0	0	0
ENSCAFG00845004858	991	950	1056	1039
ENSCAFG00845016838	0	0	0	0

ENSCAFG00845004859	2	0	0	0
ENSCAFG00845016837	5	0	2	3
ENSCAFG00845016836	0	1	0	0
ENSCAFG00845016835	909	843	959	886
ENSCAFG00845016834	32	32	26	33
ENSCAFG00845016833	0	0	0	0
ENSCAFG00845016832	132	109	99	124
ENSCAFG00845016831	1	2	5	1
ENSCAFG00845016830	0	0	0	0
ENSCAFG00845026397	0	0	0	0
ENSCAFG00845026396	736	687	790	774
ENSCAFG00845026399	0	0	0	0
ENSCAFG00845026398	0	0	0	0
ENSCAFG00845026393	5	8	6	9
ENSCAFG00845026392	1	2	0	0
ENSCAFG00845026395	0	0	0	0
ENSCAFG00845026394	487	447	458	460
ENSCAFG00845026391	0	1	1	2
ENSCAFG00845026390	10163	10002	9722	10532
ENSCAFG00845026389	3469	3165	3058	3102
ENSCAFG00845026386	0	0	0	0
ENSCAFG00845026385	0	0	0	0
ENSCAFG00845026388	0	0	0	0
ENSCAFG00845026387	11	12	7	1
ENSCAFG00845026382	197	163	204	176
ENSCAFG00845026381	0	0	0	0
ENSCAFG00845026384	1024	934	1066	1072
ENSCAFG00845026383	17	6	5	9
ENSCAFG00845026380	2967	2869	2989	2986
ENSCAFG00845014397	37	36	30	39
ENSCAFG00845014398	0	0	1	0
ENSCAFG00845014395	0	0	0	0
ENSCAFG00845014396	0	0	0	0
ENSCAFG00845014393	4	0	2	1
ENSCAFG00845014394	764	814	691	716
ENSCAFG00845014391	0	0	1	0
ENSCAFG00845014392	0	0	0	0
ENSCAFG00845014390	0	0	0	0
ENSCAFG00845026379	536	495	472	411
ENSCAFG00845026378	1	1	0	0
ENSCAFG00845026375	0	0	0	0

ENSCAFG00845026374	0	0	0	0
ENSCAFG00845026377	0	0	0	0
ENSCAFG00845026376	789	759	757	818
ENSCAFG00845026371	0	0	0	0
ENSCAFG00845026370	0	0	0	0
ENSCAFG00845026373	3890	3773	3955	3954
ENSCAFG00845026372	0	0	0	0
ENSCAFG00845014399	249	240	265	293
ENSCAFG00845014386	0	0	0	0
ENSCAFG00845014387	0	0	0	0
ENSCAFG00845014384	59	62	88	55
ENSCAFG00845014385	0	0	0	0
ENSCAFG00845014382	0	0	0	0
ENSCAFG00845014383	2046	1823	2007	1914
ENSCAFG00845014380	971	984	723	730
ENSCAFG00845014381	0	0	0	0
ENSCAFG00845026368	708	631	661	575
ENSCAFG00845026367	0	0	0	0
ENSCAFG00845002399	71	53	68	60
ENSCAFG00845002398	6	5	3	3
ENSCAFG00845026369	407	488	482	503
ENSCAFG00845002397	17	13	5	18
ENSCAFG00845026364	0	1	3	0
ENSCAFG00845002396	5	10	17	24
ENSCAFG00845026363	0	1	0	0
ENSCAFG00845002395	704	693	633	627
ENSCAFG00845026366	50	40	31	28
ENSCAFG00845002394	0	0	0	0
ENSCAFG00845026365	1742	1679	1798	1800
ENSCAFG00845002393	0	0	1	0
ENSCAFG00845026360	0	0	0	0
ENSCAFG00845002392	9	12	9	6
ENSCAFG00845002391	816	823	741	765
ENSCAFG00845026362	763	683	794	799
ENSCAFG00845002390	0	0	0	0
ENSCAFG00845026361	605	532	552	503
ENSCAFG00845014388	566	535	509	593
ENSCAFG00845014389	83	75	90	57
ENSCAFG00845014375	16	12	24	6
ENSCAFG00845014376	0	0	0	0
ENSCAFG00845014373	0	0	0	0

ENSCAFG00845014374	0	0	0	0
ENSCAFG00845014371	2	3	0	4
ENSCAFG00845014372	3	0	1	1
ENSCAFG00845014370	270	264	250	298
ENSCAFG00845026357	554	587	574	608
ENSCAFG00845026356	26	32	45	21
ENSCAFG00845026359	0	0	0	0
ENSCAFG00845026358	2730	2707	2853	2901
ENSCAFG00845026353	1039	1059	1010	1016
ENSCAFG00845026352	6	4	7	3
ENSCAFG00845026355	0	0	0	0
ENSCAFG00845026354	0	1	2	0
ENSCAFG00845026351	0	0	0	0
ENSCAFG00845026350	0	0	0	1
ENSCAFG00845014379	0	0	0	0
ENSCAFG00845014377	1186	1095	1029	995
ENSCAFG00845014378	38	27	26	48
ENSCAFG00845028929	105	108	122	133
ENSCAFG00845028927	6	6	21	8
ENSCAFG00845028928	53	43	62	73
ENSCAFG00845028925	7	10	5	6
ENSCAFG00845028926	3	2	2	2
ENSCAFG00845028923	597	514	568	584
ENSCAFG00845028924	1559	1578	1397	1505
ENSCAFG00845028921	3537	3588	3169	3402
ENSCAFG00845028922	33	28	22	34
ENSCAFG00845004960	386	323	377	389
ENSCAFG00845028920	2515	2214	2132	2313
ENSCAFG00845004961	0	0	0	0
ENSCAFG00845004962	323	289	260	236
ENSCAFG00845004963	4	1	1	3
ENSCAFG00845004964	166	180	124	195
ENSCAFG00845004965	5	0	3	5
ENSCAFG00845004966	1090	1073	1050	970
ENSCAFG00845004967	0	0	0	0
ENSCAFG00845016949	228	207	161	199
ENSCAFG00845004968	1	0	0	0
ENSCAFG00845016948	0	0	0	0
ENSCAFG00845004969	0	0	0	0
ENSCAFG00845016947	93	123	136	92
ENSCAFG00845016946	552	497	441	512

ENSCAFG00845016945	260	328	310	264
ENSCAFG00845016944	191	194	159	184
ENSCAFG00845016943	1084	970	1018	994
ENSCAFG00845016942	187	218	179	235
ENSCAFG00845016941	0	0	0	0
ENSCAFG00845016940	13	14	6	9
ENSCAFG00845028918	16528	16132	16243	16535
ENSCAFG00845002309	749	700	758	742
ENSCAFG00845028919	274	248	149	220
ENSCAFG00845002308	0	1	0	0
ENSCAFG00845028916	4177	4164	3784	3869
ENSCAFG00845002307	0	0	0	0
ENSCAFG00845028917	0	0	0	0
ENSCAFG00845002306	0	0	0	0
ENSCAFG00845028914	766	799	803	886
ENSCAFG00845002305	0	0	0	0
ENSCAFG00845028915	0	0	0	0
ENSCAFG00845002304	225	227	211	191
ENSCAFG00845028912	95	122	100	126
ENSCAFG00845002303	45	50	11	17
ENSCAFG00845028913	710	693	811	737
ENSCAFG00845002302	0	0	0	0
ENSCAFG00845028910	385	380	435	410
ENSCAFG00845002301	1	0	1	0
ENSCAFG00845028911	78	51	47	52
ENSCAFG00845002300	1308	1401	1260	1255
ENSCAFG00845004950	9	1	7	4
ENSCAFG00845004951	61	45	62	54
ENSCAFG00845004952	3	13	6	5
ENSCAFG00845004953	415	445	507	530
ENSCAFG00845004954	0	0	0	0
ENSCAFG00845004955	1116	1050	1032	1140
ENSCAFG00845016939	0	0	0	0
ENSCAFG00845004956	15	10	19	21
ENSCAFG00845016938	96	104	115	113
ENSCAFG00845004957	659	601	435	497
ENSCAFG00845016937	0	0	0	0
ENSCAFG00845004958	0	0	0	0
ENSCAFG00845016936	354	285	204	252
ENSCAFG00845004959	0	0	0	0
ENSCAFG00845016935	0	0	0	0

ENSCAFG00845016934	634	590	533	532
ENSCAFG00845016933	840	730	877	828
ENSCAFG00845016932	260	259	292	256
ENSCAFG00845016931	0	0	0	0
ENSCAFG00845016930	0	0	0	0
ENSCAFG00845028909	31	21	23	39
ENSCAFG00845028907	215	214	253	201
ENSCAFG00845028908	1233	1148	1220	1173
ENSCAFG00845028905	384	430	418	418
ENSCAFG00845028906	17	15	22	25
ENSCAFG00845028903	12	8	19	18
ENSCAFG00845028904	215	195	190	222
ENSCAFG00845028901	2	0	0	2
ENSCAFG00845028902	13	6	12	9
ENSCAFG00845028900	27	18	43	32
ENSCAFG00845004940	1	1	1	2
ENSCAFG00845004941	618	614	655	720
ENSCAFG00845004942	194	203	244	224
ENSCAFG00845004943	13	15	12	16
ENSCAFG00845016929	0	0	0	0
ENSCAFG00845004944	9	7	17	5
ENSCAFG00845016928	584	549	625	588
ENSCAFG00845004945	0	1	0	0
ENSCAFG00845016927	0	0	0	0
ENSCAFG00845004946	38	45	50	49
ENSCAFG00845016926	0	0	0	0
ENSCAFG00845004947	0	0	0	0
ENSCAFG00845016925	1584	1654	1501	1629
ENSCAFG00845004948	188	160	150	168
ENSCAFG00845016924	1389	1425	1448	1426
ENSCAFG00845004949	0	0	0	1
ENSCAFG00845016923	0	0	0	0
ENSCAFG00845016922	10	6	8	1
ENSCAFG00845016921	2672	2578	2402	2422
ENSCAFG00845016920	0	0	0	0
ENSCAFG00845004930	716	811	693	770
ENSCAFG00845004931	1	3	2	0
ENSCAFG00845016919	0	0	0	0
ENSCAFG00845004932	248	281	233	222
ENSCAFG00845016918	0	0	0	0
ENSCAFG00845004933	0	0	0	0

ENSCAFG00845016917	1	0	2	0
ENSCAFG00845004934	0	0	0	0
ENSCAFG00845016916	0	0	0	1
ENSCAFG00845004935	0	0	3	0
ENSCAFG00845016915	0	1	0	0
ENSCAFG00845004936	68	64	94	75
ENSCAFG00845016914	0	0	0	0
ENSCAFG00845004937	0	0	0	0
ENSCAFG00845016913	402	384	421	399
ENSCAFG00845004938	3	0	0	0
ENSCAFG00845016912	33	29	45	26
ENSCAFG00845004939	197	176	147	158
ENSCAFG00845016911	1500	1452	1621	1611
ENSCAFG00845016910	50	36	46	46
ENSCAFG00845016909	1	0	2	1
ENSCAFG00845004920	21	19	21	39
ENSCAFG00845016908	0	0	0	0
ENSCAFG00845004921	277	236	253	294
ENSCAFG00845016907	15	23	25	30
ENSCAFG00845004922	12	14	26	21
ENSCAFG00845016906	1	1	2	1
ENSCAFG00845004923	933	873	961	961
ENSCAFG00845016905	0	0	5	3
ENSCAFG00845004924	112	96	83	97
ENSCAFG00845016904	515	505	528	537
ENSCAFG00845004925	0	0	0	0
ENSCAFG00845016903	0	0	0	0
ENSCAFG00845004926	4	1	1	1
ENSCAFG00845016902	20	23	23	35
ENSCAFG00845004927	0	0	0	0
ENSCAFG00845016901	7	1	5	15
ENSCAFG00845004928	2011	1833	1836	1774
ENSCAFG00845016900	0	1	0	0
ENSCAFG00845004929	2	3	2	9
ENSCAFG00845004910	0	0	0	0
ENSCAFG00845004911	117	115	145	115
ENSCAFG00845004912	387	415	387	361
ENSCAFG00845004913	5	1	1	1
ENSCAFG00845004914	0	1	0	0
ENSCAFG00845004915	173	167	133	134
ENSCAFG00845004916	0	2	2	0

ENSCAFG00845004917	0	0	0	0
ENSCAFG00845004918	0	0	0	0
ENSCAFG00845004919	7306	6944	6651	6867
ENSCAFG00845004900	2797	2686	2479	2765
ENSCAFG00845004901	970	875	947	939
ENSCAFG00845004902	0	0	0	0
ENSCAFG00845004903	1	0	0	4
ENSCAFG00845004904	634	528	590	530
ENSCAFG00845004905	1431	1450	1396	1456
ENSCAFG00845004906	0	0	0	0
ENSCAFG00845004907	0	0	0	0
ENSCAFG00845004908	0	0	0	0
ENSCAFG00845004909	1	0	3	2
ENSCAFG00845014364	150	144	140	159
ENSCAFG00845014365	207	194	185	218
ENSCAFG00845014362	23	10	14	9
ENSCAFG00845014363	0	0	0	0
ENSCAFG00845014360	1594	1559	1618	1561
ENSCAFG00845014361	0	0	0	0
ENSCAFG00845026349	473	444	473	451
ENSCAFG00845002379	664	715	625	620
ENSCAFG00845026346	0	0	0	0
ENSCAFG00845002378	25	25	27	18
ENSCAFG00845026345	3650	3704	3609	3639
ENSCAFG00845002377	0	2	3	0
ENSCAFG00845026348	0	0	0	0
ENSCAFG00845002376	146	136	192	151
ENSCAFG00845026347	0	0	0	0
ENSCAFG00845002375	0	1	0	3
ENSCAFG00845026342	0	0	0	0
ENSCAFG00845002374	443	389	380	412
ENSCAFG00845026341	1836	1809	1746	1848
ENSCAFG00845002373	0	0	0	0
ENSCAFG00845026344	0	0	0	0
ENSCAFG00845002372	0	0	0	0
ENSCAFG00845026343	0	0	0	0
ENSCAFG00845002371	694	713	702	737
ENSCAFG00845002370	1142	1090	1074	1125
ENSCAFG00845026340	2050	1792	1996	2009
ENSCAFG00845014368	0	0	0	0
ENSCAFG00845014369	263	247	250	242

ENSCAFG00845014366	0	0	0	0
ENSCAFG00845014367	0	2	0	0
ENSCAFG00845014353	0	0	0	0
ENSCAFG00845014354	1	2	1	0
ENSCAFG00845014351	0	0	0	0
ENSCAFG00845014352	0	0	0	0
ENSCAFG00845014350	1226	1180	1247	1198
ENSCAFG00845026339	2	2	2	3
ENSCAFG00845026338	0	0	0	1
ENSCAFG00845026335	1	0	0	0
ENSCAFG00845028998	0	1	1	0
ENSCAFG00845002389	29	30	55	30
ENSCAFG00845026334	472	443	466	470
ENSCAFG00845028999	0	0	1	0
ENSCAFG00845002388	0	1	0	0
ENSCAFG00845026337	990	925	960	1010
ENSCAFG00845028996	0	0	0	0
ENSCAFG00845002387	620	620	629	603
ENSCAFG00845026336	427	386	379	393
ENSCAFG00845028997	3526	3365	3239	3269
ENSCAFG00845002386	3	0	0	7
ENSCAFG00845026331	3	5	3	1
ENSCAFG00845028994	45	57	42	39
ENSCAFG00845002385	0	0	0	0
ENSCAFG00845026330	0	0	0	0
ENSCAFG00845028995	93	64	79	86
ENSCAFG00845002384	1003	1017	958	919
ENSCAFG00845026333	0	0	0	0
ENSCAFG00845028992	795	824	732	717
ENSCAFG00845002383	2	1	1	4
ENSCAFG00845026332	1300	1201	1261	1284
ENSCAFG00845028993	0	0	0	0
ENSCAFG00845002382	10	4	5	5
ENSCAFG00845028990	0	0	0	0
ENSCAFG00845002381	0	2	0	0
ENSCAFG00845028991	0	0	0	0
ENSCAFG00845002380	497	480	403	388
ENSCAFG00845014359	0	0	0	0
ENSCAFG00845014357	0	0	0	0
ENSCAFG00845014358	524	466	498	505
ENSCAFG00845014355	0	0	0	0

ENSCAFG00845014356	1	1	1	3
ENSCAFG00845014342	0	0	0	0
ENSCAFG00845014343	1	0	0	0
ENSCAFG00845014340	0	0	0	0
ENSCAFG00845014341	0	0	0	0
ENSCAFG00845026327	565	591	556	489
ENSCAFG00845002359	20	19	13	17
ENSCAFG00845028989	0	0	0	2
ENSCAFG00845002358	279	306	317	306
ENSCAFG00845026329	0	0	0	0
ENSCAFG00845002357	0	0	0	0
ENSCAFG00845026324	80	70	55	73
ENSCAFG00845028987	0	0	0	0
ENSCAFG00845002356	193	179	135	160
ENSCAFG00845026323	0	0	0	1
ENSCAFG00845028988	158	157	150	124
ENSCAFG00845002355	0	0	0	0
ENSCAFG00845026326	0	0	0	0
ENSCAFG00845028985	0	0	0	0
ENSCAFG00845002354	0	0	0	0
ENSCAFG00845026325	367	436	472	369
ENSCAFG00845028986	1	0	0	0
ENSCAFG00845002353	30	36	53	38
ENSCAFG00845026320	0	0	0	0
ENSCAFG00845028983	13	12	13	18
ENSCAFG00845002352	3	2	1	2
ENSCAFG00845028984	0	0	0	0
ENSCAFG00845002351	0	0	2	0
ENSCAFG00845026322	0	0	0	0
ENSCAFG00845028981	13	19	22	20
ENSCAFG00845002350	3	5	1	3
ENSCAFG00845026321	0	0	0	0
ENSCAFG00845028982	1590	1439	1462	1486
ENSCAFG00845028980	0	0	0	0
ENSCAFG00845014348	1052	1007	1099	996
ENSCAFG00845014349	10	5	6	1
ENSCAFG00845014346	0	0	0	0
ENSCAFG00845014347	192	199	194	235
ENSCAFG00845014344	0	0	0	0
ENSCAFG00845014345	1375	1434	1347	1428
ENSCAFG00845014331	4233	4143	3975	3935

ENSCAFG00845016994	1	0	0	1
ENSCAFG00845014332	1240	1132	1031	1122
ENSCAFG00845016993	6	8	11	3
ENSCAFG00845016992	540	489	560	518
ENSCAFG00845014330	0	0	0	0
ENSCAFG00845016991	230	238	198	212
ENSCAFG00845016990	0	0	0	0
ENSCAFG00845026317	0	0	0	0
ENSCAFG00845026316	48	48	34	49
ENSCAFG00845026319	0	0	0	0
ENSCAFG00845028978	1	0	0	0
ENSCAFG00845002369	0	0	0	0
ENSCAFG00845026318	5	3	7	4
ENSCAFG00845028979	0	1	2	2
ENSCAFG00845002368	0	0	0	3
ENSCAFG00845026313	0	0	0	0
ENSCAFG00845028976	0	0	0	0
ENSCAFG00845002367	812	780	908	978
ENSCAFG00845026312	10	8	1	8
ENSCAFG00845028977	954	1060	926	989
ENSCAFG00845002366	578	529	618	620
ENSCAFG00845026315	0	0	0	0
ENSCAFG00845028974	72	58	98	75
ENSCAFG00845002365	11	13	7	20
ENSCAFG00845026314	0	0	0	0
ENSCAFG00845028975	0	0	0	0
ENSCAFG00845002364	7	10	7	9
ENSCAFG00845028972	122	153	113	122
ENSCAFG00845002363	7	6	7	16
ENSCAFG00845028973	566	517	533	577
ENSCAFG00845002362	0	0	0	0
ENSCAFG00845026311	1449	1358	1448	1415
ENSCAFG00845028970	198	250	261	231
ENSCAFG00845002361	0	0	0	0
ENSCAFG00845026310	0	0	0	0
ENSCAFG00845028971	0	0	0	0
ENSCAFG00845002360	0	0	0	0
ENSCAFG00845014339	471	471	474	408
ENSCAFG00845014337	3797	3749	5058	5216
ENSCAFG00845014338	1821	1810	1832	2063
ENSCAFG00845016999	0	0	0	0

ENSCAFG00845014335	569	520	511	592
ENSCAFG00845016998	222	235	191	181
ENSCAFG00845014336	13	18	7	13
ENSCAFG00845016997	117	87	88	82
ENSCAFG00845014333	1003	1057	887	931
ENSCAFG00845016996	1336	1255	1199	1180
ENSCAFG00845014334	10	16	17	26
ENSCAFG00845016995	3321	3464	3039	3041
ENSCAFG00845014320	0	0	0	0
ENSCAFG00845016983	0	0	0	0
ENSCAFG00845014321	0	0	0	0
ENSCAFG00845016982	367	296	280	287
ENSCAFG00845016981	568	628	646	609
ENSCAFG00845016980	5017	4807	4164	4275
ENSCAFG00845026309	0	0	0	0
ENSCAFG00845002339	0	0	0	0
ENSCAFG00845026306	0	0	0	0
ENSCAFG00845028969	1	0	0	0
ENSCAFG00845002338	809	748	752	769
ENSCAFG00845026305	244	229	288	281
ENSCAFG00845002337	175	171	153	163
ENSCAFG00845026308	0	0	0	0
ENSCAFG00845028967	611	626	589	626
ENSCAFG00845002336	14	14	13	12
ENSCAFG00845026307	0	0	0	0
ENSCAFG00845028968	221	209	193	179
ENSCAFG00845002335	2	6	5	4
ENSCAFG00845026302	0	0	0	0
ENSCAFG00845028965	0	0	0	0
ENSCAFG00845002334	0	2	1	2
ENSCAFG00845026301	6	6	0	2
ENSCAFG00845028966	49	33	51	43
ENSCAFG00845002333	2	1	1	0
ENSCAFG00845026304	8	6	9	6
ENSCAFG00845028963	0	0	0	0
ENSCAFG00845002332	0	0	0	1
ENSCAFG00845026303	0	0	0	0
ENSCAFG00845028964	0	0	0	0
ENSCAFG00845002331	8578	8736	8653	8024
ENSCAFG00845028961	650	534	589	663
ENSCAFG00845002330	0	0	0	0

ENSCAFG00845028962	662	655	616	598
ENSCAFG00845028960	2	3	1	5
ENSCAFG00845014328	1	1	2	0
ENSCAFG00845014329	278	280	279	267
ENSCAFG00845014326	0	0	0	0
ENSCAFG00845016989	2892	2808	2617	2602
ENSCAFG00845014327	0	0	0	0
ENSCAFG00845016988	84	88	60	64
ENSCAFG00845014324	0	0	0	0
ENSCAFG00845016987	0	0	0	0
ENSCAFG00845014325	836	808	715	673
ENSCAFG00845016986	25	29	31	25
ENSCAFG00845014322	6	2	2	3
ENSCAFG00845016985	0	0	0	0
ENSCAFG00845014323	0	0	0	0
ENSCAFG00845016984	0	0	0	0
ENSCAFG00845016972	28	15	5	12
ENSCAFG00845014310	2221	2034	2106	2125
ENSCAFG00845016971	12	16	14	26
ENSCAFG00845016970	325	248	261	284
ENSCAFG00845028958	2	0	1	0
ENSCAFG00845002349	12	5	10	9
ENSCAFG00845028959	3997	3909	4043	3841
ENSCAFG00845002348	3410	3215	2949	3028
ENSCAFG00845028956	700	615	612	663
ENSCAFG00845002347	1533	1466	1537	1554
ENSCAFG00845028957	0	0	0	0
ENSCAFG00845002346	0	0	2	0
ENSCAFG00845004990	0	0	0	0
ENSCAFG00845028954	11	19	14	19
ENSCAFG00845002345	1230	1254	1510	1421
ENSCAFG00845004991	133	137	148	140
ENSCAFG00845028955	1212	1222	1030	1128
ENSCAFG00845002344	595	591	577	573
ENSCAFG00845004992	799	790	647	640
ENSCAFG00845028952	263	265	253	302
ENSCAFG00845002343	0	0	0	0
ENSCAFG00845004993	0	0	0	0
ENSCAFG00845028953	47	34	35	48
ENSCAFG00845002342	0	0	0	1
ENSCAFG00845004994	3221	3220	3008	3119

ENSCAFG00845028950	64	52	46	65
ENSCAFG00845002341	27	19	10	25
ENSCAFG00845004995	176	152	248	258
ENSCAFG00845028951	0	0	0	2
ENSCAFG00845002340	707	666	769	816
ENSCAFG00845004996	1728	1610	1417	1456
ENSCAFG00845004997	0	1	0	0
ENSCAFG00845004998	0	0	0	0
ENSCAFG00845004999	7	3	5	7
ENSCAFG00845014319	0	0	0	0
ENSCAFG00845014317	476	471	488	540
ENSCAFG00845014318	2082	2011	1971	1825
ENSCAFG00845016979	0	0	0	0
ENSCAFG00845014315	473	420	449	400
ENSCAFG00845016978	0	0	0	0
ENSCAFG00845014316	0	0	0	0
ENSCAFG00845016977	123	68	120	96
ENSCAFG00845014313	409	411	341	367
ENSCAFG00845016976	36	46	37	59
ENSCAFG00845014314	1646	1565	1734	1752
ENSCAFG00845016975	645	529	548	558
ENSCAFG00845014311	12	21	9	14
ENSCAFG00845016974	457	327	433	447
ENSCAFG00845014312	0	0	0	0
ENSCAFG00845016973	1115	1058	1002	1001
ENSCAFG00845016961	0	0	0	0
ENSCAFG00845016960	0	0	0	0
ENSCAFG00845002319	0	0	0	0
ENSCAFG00845028949	272	273	235	287
ENSCAFG00845002318	18	26	16	21
ENSCAFG00845002317	0	0	0	0
ENSCAFG00845028947	0	0	0	0
ENSCAFG00845002316	22	16	47	35
ENSCAFG00845028948	1023	983	982	925
ENSCAFG00845002315	28	18	22	13
ENSCAFG00845028945	40	55	46	60
ENSCAFG00845002314	0	0	0	0
ENSCAFG00845028946	0	0	0	1
ENSCAFG00845002313	0	0	0	0
ENSCAFG00845028943	0	0	0	0
ENSCAFG00845002312	34	18	53	23

ENSCAFG00845004980	763	736	553	672
ENSCAFG00845028944	0	0	0	0
ENSCAFG00845002311	0	0	0	0
ENSCAFG00845004981	2	2	2	3
ENSCAFG00845028941	0	1	0	0
ENSCAFG00845002310	0	0	7	1
ENSCAFG00845004982	408	422	422	387
ENSCAFG00845028942	0	0	0	1
ENSCAFG00845004983	0	1	0	7
ENSCAFG00845004984	0	0	0	0
ENSCAFG00845028940	307	291	313	342
ENSCAFG00845004985	0	0	0	1
ENSCAFG00845004986	0	0	0	0
ENSCAFG00845004987	0	0	0	0
ENSCAFG00845004988	167	145	159	135
ENSCAFG00845004989	2020	2146	1944	1998
ENSCAFG00845014308	300	315	303	288
ENSCAFG00845014309	172	141	97	108
ENSCAFG00845014306	4	3	3	3
ENSCAFG00845016969	4534	4433	4154	4041
ENSCAFG00845014307	437	368	287	392
ENSCAFG00845016968	0	0	0	0
ENSCAFG00845014304	2007	2013	1851	1756
ENSCAFG00845016967	146	143	169	185
ENSCAFG00845014305	798	789	811	797
ENSCAFG00845016966	4	1	1	2
ENSCAFG00845014302	22	17	28	19
ENSCAFG00845016965	305	240	244	299
ENSCAFG00845014303	14	21	16	15
ENSCAFG00845016964	0	0	0	0
ENSCAFG00845014300	672	593	570	559
ENSCAFG00845016963	53	57	58	49
ENSCAFG00845014301	1	0	2	1
ENSCAFG00845016962	1658	1477	1714	1795
ENSCAFG00845016950	0	0	0	0
ENSCAFG00845028938	0	0	0	0
ENSCAFG00845002329	0	1	1	2
ENSCAFG00845028939	4518	4452	4228	4059
ENSCAFG00845002328	994	971	895	976
ENSCAFG00845028936	2051	1876	2162	2174
ENSCAFG00845002327	1	1	1	2

ENSCAFG00845028937	739	763	718	703
ENSCAFG00845002326	10	13	16	11
ENSCAFG00845028934	241	207	256	257
ENSCAFG00845002325	3411	3551	3431	3477
ENSCAFG00845028935	155	125	110	115
ENSCAFG00845002324	8	22	5	20
ENSCAFG00845028932	776	740	835	822
ENSCAFG00845002323	1155	1099	1191	1318
ENSCAFG00845028933	3	2	3	4
ENSCAFG00845002322	922	1028	970	1030
ENSCAFG00845004970	3010	2737	3037	3069
ENSCAFG00845028930	1	0	0	2
ENSCAFG00845002321	0	0	0	0
ENSCAFG00845004971	4401	4176	3501	3527
ENSCAFG00845028931	603	666	700	680
ENSCAFG00845002320	12	12	25	22
ENSCAFG00845004972	680	640	647	682
ENSCAFG00845004973	1	0	0	0
ENSCAFG00845004974	74	60	92	74
ENSCAFG00845004975	1	0	0	0
ENSCAFG00845004976	25	21	13	20
ENSCAFG00845004977	62	54	49	74
ENSCAFG00845004978	141	133	125	122
ENSCAFG00845004979	3899	3737	3849	3815
ENSCAFG00845016959	2	5	4	3
ENSCAFG00845016958	0	0	0	0
ENSCAFG00845016957	23	11	9	23
ENSCAFG00845016956	242	230	224	272
ENSCAFG00845016955	0	0	0	0
ENSCAFG00845016954	0	0	0	0
ENSCAFG00845016953	0	0	0	0
ENSCAFG00845016952	7	14	12	7
ENSCAFG00845016951	8	7	7	3
ENSCAFG00845026499	0	0	0	0
ENSCAFG00845026495	1	2	2	0
ENSCAFG00845026498	510	499	485	499
ENSCAFG00845026497	0	0	0	0
ENSCAFG00845026492	567	558	553	535
ENSCAFG00845026491	0	0	0	0
ENSCAFG00845026494	0	0	0	0
ENSCAFG00845026493	0	0	0	0

ENSCAFG00845026490	0	0	0	0
ENSCAFG00845026489	64	78	78	88
ENSCAFG00845026488	0	0	0	0
ENSCAFG00845026485	14	8	9	8
ENSCAFG00845026484	0	0	2	0
ENSCAFG00845026487	9	12	20	18
ENSCAFG00845026486	305	284	348	312
ENSCAFG00845026481	1	1	3	1
ENSCAFG00845026480	611	640	669	714
ENSCAFG00845026483	0	1	0	0
ENSCAFG00845026482	0	0	1	0
ENSCAFG00845014496	0	0	0	0
ENSCAFG00845014497	0	0	0	0
ENSCAFG00845014494	361	325	214	246
ENSCAFG00845014495	73	83	98	85
ENSCAFG00845014492	114	131	109	119
ENSCAFG00845014493	22	4	5	12
ENSCAFG00845014490	0	0	0	0
ENSCAFG00845014491	0	0	1	0
ENSCAFG00845026478	73	70	49	60
ENSCAFG00845026477	8	1	5	12
ENSCAFG00845026479	52	49	35	66
ENSCAFG00845026474	0	0	0	0
ENSCAFG00845026473	0	0	0	0
ENSCAFG00845026476	0	0	0	0
ENSCAFG00845026475	3168	3203	2852	2912
ENSCAFG00845026470	0	1	1	0
ENSCAFG00845026472	0	0	0	0
ENSCAFG00845026471	0	0	0	0
ENSCAFG00845014498	326	377	258	249
ENSCAFG00845014499	0	0	0	2
ENSCAFG00845002419	0	0	0	0
ENSCAFG00845002418	0	0	0	1
ENSCAFG00845002417	267	273	259	293
ENSCAFG00845002416	858	825	777	776
ENSCAFG00845002415	254	225	224	257
ENSCAFG00845002414	1	0	0	0
ENSCAFG00845002413	41	50	31	53
ENSCAFG00845002412	0	0	0	0
ENSCAFG00845002411	0	0	0	0
ENSCAFG00845002410	0	0	2	0

ENSCAFG00845014409	63	67	64	51
ENSCAFG00845014407	2	0	3	0
ENSCAFG00845014408	5	9	12	9
ENSCAFG00845014405	0	0	0	0
ENSCAFG00845014406	194	177	196	196
ENSCAFG00845014403	331	323	314	333
ENSCAFG00845014404	1	1	2	8
ENSCAFG00845014401	64	79	66	80
ENSCAFG00845014402	0	0	0	0
ENSCAFG00845014400	0	0	0	0
ENSCAFG00845002429	663	613	615	631
ENSCAFG00845002428	298	264	312	285
ENSCAFG00845002427	205	187	220	233
ENSCAFG00845002426	6853	6701	6440	6560
ENSCAFG00845002425	17	10	10	8
ENSCAFG00845002424	0	0	0	0
ENSCAFG00845002423	1071	1027	1003	1009
ENSCAFG00845002422	3199	3154	3429	3487
ENSCAFG00845002421	1	0	0	0
ENSCAFG00845002420	0	0	0	0
ENSCAFG00845002409	710	780	697	708
ENSCAFG00845002408	2	3	1	0
ENSCAFG00845002407	33	18	17	12
ENSCAFG00845002406	0	0	0	0
ENSCAFG00845002405	11	9	14	6
ENSCAFG00845002404	0	0	0	0
ENSCAFG00845002403	1064	1025	929	926
ENSCAFG00845002402	0	0	0	0
ENSCAFG00845002401	0	0	0	0
ENSCAFG00845002400	0	0	0	0
ENSCAFG00845014485	0	0	0	0
ENSCAFG00845014486	366	367	372	442
ENSCAFG00845014483	53	61	49	34
ENSCAFG00845014484	0	0	0	0
ENSCAFG00845014481	7	10	9	5
ENSCAFG00845014482	315	319	337	308
ENSCAFG00845014480	2	3	0	2
ENSCAFG00845026467	4	1	1	1
ENSCAFG00845002499	0	2	0	0
ENSCAFG00845026466	0	0	0	0
ENSCAFG00845002498	3	1	6	2

ENSCAFG00845026469	26	26	23	20
ENSCAFG00845002497	202	266	226	203
ENSCAFG00845026468	0	0	0	0
ENSCAFG00845002496	514	442	409	425
ENSCAFG00845026463	600	501	577	568
ENSCAFG00845002495	1	0	0	0
ENSCAFG00845026462	197	168	182	181
ENSCAFG00845002494	17	15	17	13
ENSCAFG00845026465	0	0	0	0
ENSCAFG00845002493	1078	934	1062	1015
ENSCAFG00845026464	1493	1500	1483	1498
ENSCAFG00845002492	0	0	0	0
ENSCAFG00845002491	0	0	0	0
ENSCAFG00845002490	0	0	0	0
ENSCAFG00845026461	0	1	0	0
ENSCAFG00845026460	6	3	7	7
ENSCAFG00845014489	0	0	0	0
ENSCAFG00845014487	1408	1382	1545	1417
ENSCAFG00845014488	1422	1426	1411	1478
ENSCAFG00845014474	0	1	1	0
ENSCAFG00845014475	100	85	75	70
ENSCAFG00845014472	0	0	0	0
ENSCAFG00845014473	714	610	727	714
ENSCAFG00845014470	964	951	928	937
ENSCAFG00845014471	9	0	0	0
ENSCAFG00845026459	0	0	0	0
ENSCAFG00845026456	6	6	9	9
ENSCAFG00845026455	1	0	0	0
ENSCAFG00845026458	0	0	0	0
ENSCAFG00845026457	0	0	0	0
ENSCAFG00845026452	417	439	315	335
ENSCAFG00845026451	0	0	0	0
ENSCAFG00845026454	4	5	1	2
ENSCAFG00845026453	5221	5070	5470	5762
ENSCAFG00845014478	261	231	271	292
ENSCAFG00845014479	0	1	0	1
ENSCAFG00845014476	23	18	18	19
ENSCAFG00845014477	0	2	3	4
ENSCAFG00845014463	0	0	1	0
ENSCAFG00845014464	0	0	0	0
ENSCAFG00845014461	121	97	115	110

ENSCAFG00845014462	0	0	0	0
ENSCAFG00845014460	123	110	128	145
ENSCAFG00845026449	0	0	0	0
ENSCAFG00845026448	64	75	74	75
ENSCAFG00845002479	486	465	449	454
ENSCAFG00845002478	0	0	3	1
ENSCAFG00845026445	0	0	0	0
ENSCAFG00845002477	0	0	0	0
ENSCAFG00845026444	0	0	0	0
ENSCAFG00845002476	1572	1505	1502	1604
ENSCAFG00845026447	0	0	0	0
ENSCAFG00845002475	803	758	864	832
ENSCAFG00845026446	12	8	5	3
ENSCAFG00845002474	25	42	34	28
ENSCAFG00845026441	1074	1126	1062	1062
ENSCAFG00845002473	0	0	0	2
ENSCAFG00845026440	0	0	0	0
ENSCAFG00845002472	294	251	275	250
ENSCAFG00845026443	0	0	0	0
ENSCAFG00845002471	0	0	0	0
ENSCAFG00845026442	590	577	529	491
ENSCAFG00845002470	503	491	535	524
ENSCAFG00845014469	9	5	10	4
ENSCAFG00845014467	438	478	411	538
ENSCAFG00845014468	72	67	52	84
ENSCAFG00845014465	1238	1219	1407	1330
ENSCAFG00845014466	0	0	0	0
ENSCAFG00845014452	371	384	340	351
ENSCAFG00845014453	0	1	0	0
ENSCAFG00845014450	2114	1926	2074	2141
ENSCAFG00845014451	2835	2671	2921	2809
ENSCAFG00845026438	0	0	0	0
ENSCAFG00845026437	92	99	146	98
ENSCAFG00845026439	1871	1755	1559	1603
ENSCAFG00845002489	978	815	887	811
ENSCAFG00845026434	1	2	4	0
ENSCAFG00845002488	27	42	36	20
ENSCAFG00845026433	889	863	787	844
ENSCAFG00845002487	0	0	0	0
ENSCAFG00845002486	354	328	327	362
ENSCAFG00845026435	0	3	0	1

ENSCAFG00845002485	338	311	374	366
ENSCAFG00845026430	147	166	178	200
ENSCAFG00845002484	865	842	807	772
ENSCAFG00845002483	0	0	4	2
ENSCAFG00845026432	0	0	0	0
ENSCAFG00845002482	361	353	423	440
ENSCAFG00845026431	792	716	780	788
ENSCAFG00845002481	0	0	0	0
ENSCAFG00845002480	0	0	0	0
ENSCAFG00845014458	972	932	1091	1048
ENSCAFG00845014459	641	638	671	696
ENSCAFG00845014456	51	55	67	62
ENSCAFG00845014457	97	83	75	66
ENSCAFG00845014454	0	0	1	2
ENSCAFG00845014455	0	0	0	0
ENSCAFG00845014441	0	0	0	0
ENSCAFG00845014442	0	0	0	0
ENSCAFG00845014440	0	0	0	0
ENSCAFG00845026427	0	0	0	0
ENSCAFG00845002459	5	6	3	5
ENSCAFG00845026426	0	0	0	0
ENSCAFG00845002458	173	176	160	179
ENSCAFG00845026429	3	0	0	0
ENSCAFG00845002457	0	0	0	0
ENSCAFG00845026428	944	974	1040	1112
ENSCAFG00845002456	0	0	0	0
ENSCAFG00845026423	313	244	217	233
ENSCAFG00845002455	86	80	114	122
ENSCAFG00845026422	0	0	0	0
ENSCAFG00845002454	855	815	833	845
ENSCAFG00845026425	4	3	5	6
ENSCAFG00845002453	22	36	18	25
ENSCAFG00845026424	4	3	4	0
ENSCAFG00845002452	0	0	3	0
ENSCAFG00845002451	72	91	61	60
ENSCAFG00845002450	0	0	0	0
ENSCAFG00845026421	0	0	1	1
ENSCAFG00845026420	3	1	5	2
ENSCAFG00845014449	0	1	1	4
ENSCAFG00845014447	0	0	0	0
ENSCAFG00845014448	323	234	271	225

ENSCAFG00845014445	1710	1610	1701	1756
ENSCAFG00845014446	44	42	46	57
ENSCAFG00845014443	10	18	11	4
ENSCAFG00845014444	2	2	2	5
ENSCAFG00845014430	0	0	0	0
ENSCAFG00845014431	0	0	0	0
ENSCAFG00845026419	0	1	2	0
ENSCAFG00845026416	995	1016	914	959
ENSCAFG00845002469	6696	6630	5765	6063
ENSCAFG00845026418	1	0	0	0
ENSCAFG00845002468	3	7	7	5
ENSCAFG00845026417	86	77	47	53
ENSCAFG00845002467	1709	1598	1466	1452
ENSCAFG00845026412	0	0	0	0
ENSCAFG00845002466	0	2	0	0
ENSCAFG00845026411	19	20	30	30
ENSCAFG00845002465	135	121	122	146
ENSCAFG00845026414	0	0	0	0
ENSCAFG00845002464	0	0	0	0
ENSCAFG00845026413	4242	4099	3865	3989
ENSCAFG00845002463	0	0	0	0
ENSCAFG00845002462	1	1	1	0
ENSCAFG00845002461	1841	1740	1744	1813
ENSCAFG00845026410	0	0	0	0
ENSCAFG00845002460	1412	1466	1415	1493
ENSCAFG00845014438	0	2	0	0
ENSCAFG00845014439	52	76	53	65
ENSCAFG00845014436	2043	2004	1541	1508
ENSCAFG00845014437	280	239	173	218
ENSCAFG00845014434	8	7	8	5
ENSCAFG00845014435	0	0	0	0
ENSCAFG00845014432	558	563	713	692
ENSCAFG00845014433	309	294	301	286
ENSCAFG00845014420	0	0	0	0
ENSCAFG00845026409	2	4	2	2
ENSCAFG00845026408	0	0	0	0
ENSCAFG00845002439	1074	1017	939	941
ENSCAFG00845002438	800	783	754	780
ENSCAFG00845026405	166	165	187	206
ENSCAFG00845002437	383	374	423	445
ENSCAFG00845026404	454	393	341	370

ENSCAFG00845002436	0	1	0	0
ENSCAFG00845026407	12	14	9	5
ENSCAFG00845002435	1	0	0	0
ENSCAFG00845026406	1495	1410	1410	1308
ENSCAFG00845002434	398	415	391	419
ENSCAFG00845026401	0	0	0	0
ENSCAFG00845002433	67	55	62	60
ENSCAFG00845026400	2	0	0	2
ENSCAFG00845002432	0	1	0	0
ENSCAFG00845026403	0	0	0	0
ENSCAFG00845002431	526	503	471	456
ENSCAFG00845026402	23	16	9	11
ENSCAFG00845002430	4340	4189	4546	4401
ENSCAFG00845014429	4	7	0	1
ENSCAFG00845014427	0	0	0	0
ENSCAFG00845014428	0	0	0	0
ENSCAFG00845014425	0	0	0	0
ENSCAFG00845014426	0	1	0	0
ENSCAFG00845014423	235	210	145	151
ENSCAFG00845014424	0	0	1	0
ENSCAFG00845014421	1	0	2	0
ENSCAFG00845014422	69	79	55	61
ENSCAFG00845002449	20	26	20	32
ENSCAFG00845002448	193	212	223	221
ENSCAFG00845002447	4	5	8	0
ENSCAFG00845002446	1993	1916	1687	1691
ENSCAFG00845002445	1138	1114	1095	1124
ENSCAFG00845002444	0	0	0	0
ENSCAFG00845002443	1	1	0	1
ENSCAFG00845002442	824	825	838	817
ENSCAFG00845002441	2011	2126	1960	1937
ENSCAFG00845002440	951	887	1019	1028
ENSCAFG00845014418	61	74	69	77
ENSCAFG00845014419	32	21	30	30
ENSCAFG00845014416	0	0	0	0
ENSCAFG00845014417	0	0	0	0
ENSCAFG00845014414	11	13	0	6
ENSCAFG00845014415	3	3	1	3
ENSCAFG00845014412	0	0	0	0
ENSCAFG00845014413	0	0	0	0
ENSCAFG00845014410	1663	1667	1657	1815

ENSCAFG00845014411	56	42	52	63
ENSCAFG00845026599	482	445	391	372
ENSCAFG00845026598	1436	1395	1392	1326
ENSCAFG00845026595	3692	3317	4090	4200
ENSCAFG00845026594	0	0	0	0
ENSCAFG00845026597	1	3	0	0
ENSCAFG00845026596	2019	2037	1781	1770
ENSCAFG00845026591	0	0	0	0
ENSCAFG00845026590	5691	5547	5770	5864
ENSCAFG00845026593	1	3	0	0
ENSCAFG00845026592	0	0	0	0
ENSCAFG00845026508	0	0	0	0
ENSCAFG00845026507	0	0	0	1
ENSCAFG00845002539	0	0	0	0
ENSCAFG00845002538	0	0	0	0
ENSCAFG00845026509	383	342	331	389
ENSCAFG00845002537	3	0	1	0
ENSCAFG00845026504	0	0	3	0
ENSCAFG00845002536	231	184	241	206
ENSCAFG00845026503	1	1	0	0
ENSCAFG00845002535	440	428	417	411
ENSCAFG00845026506	0	0	0	0
ENSCAFG00845002534	64	59	43	40
ENSCAFG00845026505	422	468	431	454
ENSCAFG00845002533	271	287	225	266
ENSCAFG00845026500	0	0	0	0
ENSCAFG00845002532	0	1	0	4
ENSCAFG00845002531	0	0	0	0
ENSCAFG00845026502	0	0	0	0
ENSCAFG00845002530	0	0	0	0
ENSCAFG00845026501	26	20	16	20
ENSCAFG00845014528	343	374	318	316
ENSCAFG00845014529	0	0	0	0
ENSCAFG00845014526	132	131	119	112
ENSCAFG00845014527	12	4	5	7
ENSCAFG00845014524	797	861	787	816
ENSCAFG00845014525	157	143	142	125
ENSCAFG00845014522	0	0	0	0
ENSCAFG00845014523	0	0	0	0
ENSCAFG00845014520	893	975	909	856
ENSCAFG00845014521	3236	3083	3595	3613

ENSCAFG00845002549	889	884	895	862
ENSCAFG00845002548	18	13	8	13
ENSCAFG00845002547	0	0	0	0
ENSCAFG00845002546	434	417	431	454
ENSCAFG00845002545	0	0	0	0
ENSCAFG00845002544	0	0	0	0
ENSCAFG00845002543	2272	2150	2227	2181
ENSCAFG00845002542	0	0	0	0
ENSCAFG00845002541	3440	3246	3125	3146
ENSCAFG00845002540	0	0	0	0
ENSCAFG00845014519	0	0	5	8
ENSCAFG00845014517	7	11	6	14
ENSCAFG00845014518	0	0	0	0
ENSCAFG00845014515	2	4	2	6
ENSCAFG00845014516	0	0	0	0
ENSCAFG00845014513	2319	2253	2037	2078
ENSCAFG00845014514	493	540	533	473
ENSCAFG00845014511	1	0	1	1
ENSCAFG00845014512	451	409	417	449
ENSCAFG00845014510	6668	6355	6847	7379
ENSCAFG00845002519	1	0	0	0
ENSCAFG00845002518	0	0	0	0
ENSCAFG00845002517	1	0	0	0
ENSCAFG00845002516	0	0	0	0
ENSCAFG00845002515	3	1	1	3
ENSCAFG00845002514	1369	1297	1357	1452
ENSCAFG00845002513	0	0	0	0
ENSCAFG00845002512	2	0	1	0
ENSCAFG00845002511	1	3	2	7
ENSCAFG00845002510	3	11	8	4
ENSCAFG00845014508	8	3	3	1
ENSCAFG00845014509	1217	1215	1140	1009
ENSCAFG00845014506	455	427	309	360
ENSCAFG00845014507	0	0	0	0
ENSCAFG00845014504	1291	1172	1109	1212
ENSCAFG00845014505	594	512	540	531
ENSCAFG00845014502	2	1	1	1
ENSCAFG00845014503	437	388	403	408
ENSCAFG00845014500	0	0	0	0
ENSCAFG00845014501	49	35	55	63
ENSCAFG00845002529	0	0	0	0

ENSCAFG00845002528	0	2	0	0
ENSCAFG00845002527	0	0	0	0
ENSCAFG00845002526	1539	1428	1418	1444
ENSCAFG00845002525	0	0	0	0
ENSCAFG00845002524	653	578	697	694
ENSCAFG00845002523	5	2	2	8
ENSCAFG00845002522	0	0	1	0
ENSCAFG00845002521	10321	9859	9438	9400
ENSCAFG00845002520	0	0	0	0
ENSCAFG00845002509	333	329	334	359
ENSCAFG00845002508	0	0	0	0
ENSCAFG00845002507	56	64	81	72
ENSCAFG00845002506	347	333	342	416
ENSCAFG00845002505	0	0	0	0
ENSCAFG00845002504	0	0	0	0
ENSCAFG00845002503	564	517	518	562
ENSCAFG00845002502	0	0	0	0
ENSCAFG00845002501	17	7	8	15
ENSCAFG00845002500	192	187	193	191
ENSCAFG00845026588	1	2	3	3
ENSCAFG00845026587	0	0	0	0
ENSCAFG00845026589	9	16	10	8
ENSCAFG00845026584	3	1	1	1
ENSCAFG00845026583	855	760	746	717
ENSCAFG00845026586	0	0	0	0
ENSCAFG00845026585	0	0	0	0
ENSCAFG00845026582	15011	15056	16673	17186
ENSCAFG00845026581	25	20	20	22
ENSCAFG00845014595	0	0	0	0
ENSCAFG00845014596	31	57	18	33
ENSCAFG00845014593	0	0	0	0
ENSCAFG00845014594	0	0	0	0
ENSCAFG00845014591	204	204	212	205
ENSCAFG00845014592	0	1	2	0
ENSCAFG00845014590	3	3	1	7
ENSCAFG00845026577	1266	1216	1300	1299
ENSCAFG00845026576	42	34	35	49
ENSCAFG00845026579	749	775	829	791
ENSCAFG00845026578	94	73	89	109
ENSCAFG00845026573	6494	6230	6231	6524
ENSCAFG00845026572	999	929	1055	1030

ENSCAFG00845026575	0	0	1	0
ENSCAFG00845026574	0	1	2	0
ENSCAFG00845026571	6	3	1	1
ENSCAFG00845026570	1	1	2	1
ENSCAFG00845014599	1	0	1	1
ENSCAFG00845014597	0	0	0	0
ENSCAFG00845014598	5	1	6	2
ENSCAFG00845014584	0	0	0	0
ENSCAFG00845014585	970	919	925	950
ENSCAFG00845014582	1373	1329	1386	1387
ENSCAFG00845014583	0	0	0	0
ENSCAFG00845014580	1191	1113	1112	1208
ENSCAFG00845014581	0	0	0	0
ENSCAFG00845026569	0	1	0	1
ENSCAFG00845002599	0	0	0	0
ENSCAFG00845026566	1	1	0	2
ENSCAFG00845002598	1535	1468	1527	1513
ENSCAFG00845026565	0	0	0	0
ENSCAFG00845002597	0	0	0	0
ENSCAFG00845026568	64	74	76	69
ENSCAFG00845002596	13	2	3	4
ENSCAFG00845026567	7	0	6	0
ENSCAFG00845002595	0	0	0	0
ENSCAFG00845026562	6442	6291	5907	5833
ENSCAFG00845002594	686	727	701	708
ENSCAFG00845026561	1	0	0	0
ENSCAFG00845002593	0	0	0	0
ENSCAFG00845026564	11	14	11	16
ENSCAFG00845002592	216	165	169	191
ENSCAFG00845026563	0	0	0	0
ENSCAFG00845002591	6093	5785	5810	5745
ENSCAFG00845002590	516	476	559	545
ENSCAFG00845026560	0	0	0	0
ENSCAFG00845014588	40	49	44	30
ENSCAFG00845014589	0	0	0	0
ENSCAFG00845014586	65	62	86	60
ENSCAFG00845014587	3	5	0	0
ENSCAFG00845014573	11	11	13	11
ENSCAFG00845014574	1	5	4	9
ENSCAFG00845014571	0	0	1	0
ENSCAFG00845014572	0	0	0	1

ENSCAFG00845014570	1281	1103	1267	1258
ENSCAFG00845026559	0	0	0	0
ENSCAFG00845026558	0	0	0	0
ENSCAFG00845026555	1001	1027	864	805
ENSCAFG00845026554	864	971	891	949
ENSCAFG00845026557	0	1	1	0
ENSCAFG00845026556	0	0	0	0
ENSCAFG00845026551	510	494	506	527
ENSCAFG00845026550	0	0	0	0
ENSCAFG00845026553	1875	1867	1875	2002
ENSCAFG00845026552	0	0	0	0
ENSCAFG00845014579	205	171	272	279
ENSCAFG00845014577	583	621	514	578
ENSCAFG00845014578	868	745	734	712
ENSCAFG00845014575	5	0	8	6
ENSCAFG00845014576	0	0	0	0
ENSCAFG00845014562	129	130	136	144
ENSCAFG00845014563	0	1	2	0
ENSCAFG00845014560	0	0	1	1
ENSCAFG00845014561	1	0	0	1
ENSCAFG00845026548	0	0	0	0
ENSCAFG00845026547	0	0	0	0
ENSCAFG00845002579	2251	2035	2304	2410
ENSCAFG00845002578	2	0	0	0
ENSCAFG00845026549	0	0	0	0
ENSCAFG00845002577	15	22	18	13
ENSCAFG00845026544	0	1	0	2
ENSCAFG00845002576	2	7	6	2
ENSCAFG00845026543	0	0	0	0
ENSCAFG00845002575	45	41	26	21
ENSCAFG00845026546	7	20	8	6
ENSCAFG00845002574	0	0	0	0
ENSCAFG00845026545	969	884	864	917
ENSCAFG00845002573	71	102	94	93
ENSCAFG00845026540	0	0	0	0
ENSCAFG00845002572	404	449	424	456
ENSCAFG00845002571	389	393	441	451
ENSCAFG00845026542	1	1	0	0
ENSCAFG00845002570	422	414	408	464
ENSCAFG00845026541	143	120	102	146
ENSCAFG00845014568	1779	1673	1577	1683

ENSCAFG00845014569	559	626	508	624
ENSCAFG00845014566	0	0	0	0
ENSCAFG00845014567	2005	1908	1735	1802
ENSCAFG00845014564	1	0	4	1
ENSCAFG00845014565	1692	1683	1689	1765
ENSCAFG00845014551	0	0	0	0
ENSCAFG00845014552	2800	2806	2676	2644
ENSCAFG00845014550	0	0	0	0
ENSCAFG00845026537	0	0	0	0
ENSCAFG00845026536	385	399	394	349
ENSCAFG00845026539	0	0	0	0
ENSCAFG00845002589	0	0	0	0
ENSCAFG00845026538	2	0	0	6
ENSCAFG00845002588	0	0	0	0
ENSCAFG00845026533	483	477	470	478
ENSCAFG00845002587	57	83	22	17
ENSCAFG00845026532	0	1	0	0
ENSCAFG00845002586	3220	3243	3063	3011
ENSCAFG00845026535	0	0	0	0
ENSCAFG00845002585	72	59	79	75
ENSCAFG00845026534	0	0	0	0
ENSCAFG00845002584	0	0	0	0
ENSCAFG00845002583	0	1	0	0
ENSCAFG00845002582	223	208	256	249
ENSCAFG00845026531	13	9	4	6
ENSCAFG00845002581	10	15	9	12
ENSCAFG00845026530	2	1	4	1
ENSCAFG00845002580	0	0	0	0
ENSCAFG00845014559	3	5	0	7
ENSCAFG00845014557	1304	1258	1190	1261
ENSCAFG00845014558	0	0	0	0
ENSCAFG00845014555	945	967	816	833
ENSCAFG00845014556	351	340	317	389
ENSCAFG00845014553	194	186	177	224
ENSCAFG00845014554	0	0	0	0
ENSCAFG00845014540	752	804	617	676
ENSCAFG00845014541	0	0	0	0
ENSCAFG00845026529	584	516	519	539
ENSCAFG00845002559	0	0	0	0
ENSCAFG00845026526	0	0	0	0
ENSCAFG00845002558	0	0	0	0

ENSCAFG00845026525	63	66	68	76
ENSCAFG00845002557	1	0	0	0
ENSCAFG00845026528	579	599	350	315
ENSCAFG00845002556	0	0	0	0
ENSCAFG00845026527	0	0	3	0
ENSCAFG00845002555	2	1	0	1
ENSCAFG00845026522	0	0	0	0
ENSCAFG00845002554	66	48	30	42
ENSCAFG00845026521	0	0	0	0
ENSCAFG00845002553	0	0	0	0
ENSCAFG00845026524	3692	3577	3852	3896
ENSCAFG00845002552	0	0	0	0
ENSCAFG00845026523	0	0	0	0
ENSCAFG00845002551	240	262	279	237
ENSCAFG00845002550	0	0	0	0
ENSCAFG00845014548	3718	3500	3726	3880
ENSCAFG00845014549	0	0	1	0
ENSCAFG00845014546	3131	3055	3030	2977
ENSCAFG00845014547	185	217	252	223
ENSCAFG00845014544	1422	1363	1237	1294
ENSCAFG00845014545	0	0	0	1
ENSCAFG00845014542	182	187	162	185
ENSCAFG00845014543	3	8	13	21
ENSCAFG00845014530	0	0	0	0
ENSCAFG00845026519	548	538	623	612
ENSCAFG00845026518	0	0	4	0
ENSCAFG00845026515	6	13	10	3
ENSCAFG00845002569	6023	5981	6408	6336
ENSCAFG00845026514	0	0	0	0
ENSCAFG00845002568	456	402	387	369
ENSCAFG00845026517	0	0	0	0
ENSCAFG00845002567	0	0	0	0
ENSCAFG00845026516	0	0	0	0
ENSCAFG00845002566	19	16	13	26
ENSCAFG00845026511	0	0	0	0
ENSCAFG00845002565	1138	1038	1038	1039
ENSCAFG00845026510	0	0	0	0
ENSCAFG00845002564	6936	6457	6440	6453
ENSCAFG00845026513	5519	5258	5033	5122
ENSCAFG00845002563	1128	1015	1049	1060
ENSCAFG00845026512	42	55	34	36

ENSCAFG00845002562	448	413	505	477
ENSCAFG00845002561	0	0	0	0
ENSCAFG00845002560	0	0	0	0
ENSCAFG00845014539	627	542	544	485
ENSCAFG00845014537	53	58	73	64
ENSCAFG00845014538	0	0	0	0
ENSCAFG00845014535	291	304	271	271
ENSCAFG00845014536	1015	851	809	837
ENSCAFG00845014533	0	0	0	0
ENSCAFG00845014534	2530	2453	2385	2504
ENSCAFG00845014531	14092	13660	12852	12695
ENSCAFG00845014532	19	6	8	14
ENSCAFG00845028488	1	0	0	0
ENSCAFG00845028489	0	0	0	0
ENSCAFG00845028486	1918	1839	2028	2151
ENSCAFG00845028487	1098	955	1095	1024
ENSCAFG00845028484	0	0	0	0
ENSCAFG00845028485	695	751	818	781
ENSCAFG00845028482	31	32	34	34
ENSCAFG00845028483	176	162	171	186
ENSCAFG00845028480	219	221	220	199
ENSCAFG00845028481	0	0	0	0
ENSCAFG00845016499	12	21	14	9
ENSCAFG00845016498	0	0	0	0
ENSCAFG00845016497	816	816	761	767
ENSCAFG00845016496	1134	1138	1131	1125
ENSCAFG00845016495	276	239	324	361
ENSCAFG00845016494	275	309	276	291
ENSCAFG00845016493	191	150	138	140
ENSCAFG00845016492	627	617	567	613
ENSCAFG00845016491	0	0	0	0
ENSCAFG00845016490	606	634	609	576
ENSCAFG00845028479	195	184	222	200
ENSCAFG00845028477	0	0	0	1
ENSCAFG00845028478	212	214	162	171
ENSCAFG00845028475	2	0	0	0
ENSCAFG00845028476	1	3	2	7
ENSCAFG00845028473	1431	1386	1348	1535
ENSCAFG00845028474	18	12	9	6
ENSCAFG00845028471	0	0	0	0
ENSCAFG00845028472	0	0	0	0

ENSCAFG00845028470	0	0	0	1
ENSCAFG00845016488	63	45	67	43
ENSCAFG00845016487	1483	1465	1415	1412
ENSCAFG00845016486	0	0	0	0
ENSCAFG00845016485	67	54	48	55
ENSCAFG00845016484	0	0	0	0
ENSCAFG00845016483	0	0	0	0
ENSCAFG00845016482	302	306	283	330
ENSCAFG00845016481	4145	4047	4225	4072
ENSCAFG00845016480	0	0	0	1
ENSCAFG00845028468	13	15	9	12
ENSCAFG00845028469	1391	1347	1128	1258
ENSCAFG00845028466	1435	1393	1350	1340
ENSCAFG00845028467	0	0	0	0
ENSCAFG00845028464	905	940	871	949
ENSCAFG00845028465	278	275	258	281
ENSCAFG00845028462	0	0	0	0
ENSCAFG00845028463	0	0	0	0
ENSCAFG00845028460	0	0	0	0
ENSCAFG00845028461	12	6	8	6
ENSCAFG00845016489	170	160	140	148
ENSCAFG00845016477	0	1	0	0
ENSCAFG00845016476	0	0	1	0
ENSCAFG00845016475	1239	1215	1297	1270
ENSCAFG00845016474	573	640	612	637
ENSCAFG00845016473	0	0	1	0
ENSCAFG00845016472	0	0	0	1
ENSCAFG00845016471	3	0	3	2
ENSCAFG00845004490	0	0	0	0
ENSCAFG00845016470	0	1	2	1
ENSCAFG00845004491	0	0	0	0
ENSCAFG00845004492	1531	1442	2169	2079
ENSCAFG00845004493	0	0	0	0
ENSCAFG00845004494	3	0	1	0
ENSCAFG00845004495	0	0	0	0
ENSCAFG00845028459	3	0	0	0
ENSCAFG00845004496	433	394	418	359
ENSCAFG00845004497	0	0	2	2
ENSCAFG00845028457	29	35	16	44
ENSCAFG00845004498	0	0	0	0
ENSCAFG00845028458	602	668	786	713

ENSCAFG00845004499	0	0	0	0
ENSCAFG00845028455	0	0	0	0
ENSCAFG00845028456	11419	10827	10533	10731
ENSCAFG00845028453	1	1	0	2
ENSCAFG00845028454	0	0	0	0
ENSCAFG00845028451	0	0	0	0
ENSCAFG00845028452	0	0	0	0
ENSCAFG00845028450	77	67	73	82
ENSCAFG00845016479	0	0	0	0
ENSCAFG00845016478	0	0	0	0
ENSCAFG00845016466	3633	3730	3278	3018
ENSCAFG00845016465	3	0	1	1
ENSCAFG00845016464	223	230	243	279
ENSCAFG00845016463	568	549	593	619
ENSCAFG00845016462	0	0	0	0
ENSCAFG00845016461	0	0	0	0
ENSCAFG00845016460	0	0	0	0
ENSCAFG00845004480	0	0	0	0
ENSCAFG00845004481	916	783	929	1044
ENSCAFG00845004482	184	181	158	153
ENSCAFG00845004483	1278	1146	1184	1294
ENSCAFG00845004484	7	4	1	6
ENSCAFG00845028448	334	371	350	349
ENSCAFG00845004485	6	1	2	0
ENSCAFG00845028449	511	490	430	432
ENSCAFG00845004486	0	0	0	0
ENSCAFG00845028446	4788	4664	5031	4944
ENSCAFG00845004487	1479	1361	1229	1343
ENSCAFG00845028447	2	6	9	5
ENSCAFG00845004488	3644	3441	3585	3483
ENSCAFG00845028444	212	155	158	127
ENSCAFG00845004489	1527	1480	1477	1484
ENSCAFG00845028445	0	0	0	0
ENSCAFG00845028442	0	0	0	0
ENSCAFG00845028443	1	0	0	1
ENSCAFG00845028440	207	248	211	230
ENSCAFG00845028441	2	0	0	0
ENSCAFG00845016469	2537	2551	2910	2895
ENSCAFG00845016468	0	0	0	0
ENSCAFG00845016467	0	0	0	0
ENSCAFG00845016455	0	0	0	0

ENSCAFG00845016454	6233	5866	6057	6126
ENSCAFG00845016453	7	6	14	9
ENSCAFG00845016452	0	0	0	0
ENSCAFG00845016451	2092	2077	1881	1969
ENSCAFG00845016450	58	45	41	30
ENSCAFG00845004470	0	0	0	0
ENSCAFG00845004471	40	23	44	41
ENSCAFG00845028439	0	0	0	0
ENSCAFG00845004472	34	27	30	26
ENSCAFG00845004473	3	2	1	7
ENSCAFG00845028437	0	2	0	0
ENSCAFG00845004474	0	0	0	0
ENSCAFG00845028438	2416	2467	2288	2236
ENSCAFG00845004475	31	40	56	44
ENSCAFG00845028435	1789	1658	1603	1687
ENSCAFG00845004476	22	29	42	43
ENSCAFG00845028436	496	469	936	954
ENSCAFG00845004477	0	0	0	0
ENSCAFG00845028433	0	0	0	0
ENSCAFG00845004478	0	0	0	0
ENSCAFG00845028434	1168	1161	1138	1158
ENSCAFG00845004479	2115	1917	2033	2184
ENSCAFG00845028431	73	56	56	40
ENSCAFG00845028432	0	0	0	0
ENSCAFG00845028430	0	0	0	0
ENSCAFG00845016459	2338	2326	2055	2122
ENSCAFG00845016458	18	14	9	9
ENSCAFG00845016457	255	266	263	192
ENSCAFG00845016456	860	819	885	866
ENSCAFG00845016444	714	695	447	478
ENSCAFG00845016443	79	71	52	52
ENSCAFG00845016442	0	0	1	0
ENSCAFG00845016441	0	0	0	0
ENSCAFG00845016440	2246	2182	2317	2199
ENSCAFG00845004460	0	0	0	0
ENSCAFG00845028428	0	0	0	0
ENSCAFG00845004461	833	830	905	947
ENSCAFG00845028429	0	0	0	0
ENSCAFG00845004462	0	0	0	0
ENSCAFG00845028426	0	0	0	0
ENSCAFG00845004463	0	0	0	0

ENSCAFG00845028427	199	184	218	201
ENSCAFG00845004464	0	0	0	0
ENSCAFG00845028424	534	552	540	576
ENSCAFG00845004465	222	189	216	197
ENSCAFG00845028425	632	623	619	588
ENSCAFG00845004466	5829	5630	5963	5844
ENSCAFG00845028422	24	27	12	9
ENSCAFG00845004467	0	0	2	0
ENSCAFG00845028423	0	0	0	0
ENSCAFG00845004468	8	15	27	11
ENSCAFG00845028420	6	0	4	2
ENSCAFG00845004469	844	768	763	850
ENSCAFG00845028421	0	0	0	0
ENSCAFG00845016449	473	412	404	410
ENSCAFG00845016448	514	452	421	448
ENSCAFG00845016447	0	0	0	0
ENSCAFG00845016446	0	0	0	0
ENSCAFG00845016445	0	0	0	0
ENSCAFG00845016433	0	0	0	0
ENSCAFG00845016432	1	3	5	7
ENSCAFG00845016431	246	214	244	228
ENSCAFG00845016430	202	213	219	205
ENSCAFG00845028419	0	0	6	0
ENSCAFG00845028417	0	0	0	0
ENSCAFG00845004450	0	0	0	0
ENSCAFG00845028418	0	4	0	0
ENSCAFG00845004451	0	1	0	0
ENSCAFG00845028415	722	672	736	696
ENSCAFG00845004452	0	0	0	0
ENSCAFG00845028416	0	0	0	0
ENSCAFG00845004453	0	0	0	0
ENSCAFG00845028413	11	7	6	12
ENSCAFG00845004454	0	0	0	0
ENSCAFG00845028414	0	0	0	0
ENSCAFG00845004455	0	1	2	4
ENSCAFG00845028411	250	242	219	258
ENSCAFG00845004456	0	3	4	0
ENSCAFG00845028412	1	0	3	3
ENSCAFG00845004457	0	0	0	0
ENSCAFG00845004458	0	0	0	0
ENSCAFG00845028410	0	0	0	0

ENSCAFG00845004459	47	56	90	91
ENSCAFG00845016439	33	35	30	30
ENSCAFG00845016438	731	657	663	678
ENSCAFG00845016437	0	0	0	0
ENSCAFG00845016436	670	544	658	666
ENSCAFG00845016435	1972	1831	1756	1822
ENSCAFG00845016434	1964	1978	1885	1988
ENSCAFG00845028499	1	3	6	0
ENSCAFG00845028497	0	0	2	3
ENSCAFG00845028498	2133	2170	2448	2333
ENSCAFG00845028495	2	1	3	2
ENSCAFG00845028496	0	0	0	0
ENSCAFG00845028493	0	0	0	0
ENSCAFG00845028494	42	19	36	29
ENSCAFG00845028491	0	0	0	0
ENSCAFG00845028492	1	6	3	1
ENSCAFG00845028490	0	0	0	0
ENSCAFG00845016422	1080	1105	1004	1058
ENSCAFG00845016421	175	151	179	136
ENSCAFG00845016420	0	0	6	0
ENSCAFG00845028408	0	0	0	0
ENSCAFG00845028409	0	0	0	0
ENSCAFG00845028406	0	0	0	0
ENSCAFG00845028407	1712	1625	1569	1604
ENSCAFG00845004440	0	0	0	0
ENSCAFG00845028404	0	0	0	0
ENSCAFG00845004441	0	0	0	0
ENSCAFG00845028405	0	0	0	0
ENSCAFG00845004442	2	0	0	2
ENSCAFG00845028402	2	6	1	1
ENSCAFG00845004443	0	0	0	0
ENSCAFG00845028403	0	0	0	0
ENSCAFG00845004444	116	112	112	79
ENSCAFG00845028400	44	49	29	38
ENSCAFG00845004445	0	0	0	0
ENSCAFG00845028401	0	0	0	0
ENSCAFG00845004446	0	0	0	1
ENSCAFG00845004447	0	0	0	0
ENSCAFG00845004448	0	0	0	0
ENSCAFG00845004449	142	112	139	116
ENSCAFG00845016429	0	0	0	0

ENSCAFG00845016428	1345	1275	1386	1409
ENSCAFG00845016427	0	0	0	0
ENSCAFG00845016426	0	0	0	0
ENSCAFG00845016425	1	0	2	4
ENSCAFG00845016424	0	0	0	0
ENSCAFG00845016423	10	25	17	21
ENSCAFG00845016411	234	242	218	217
ENSCAFG00845016410	965	889	927	947
ENSCAFG00845004430	1132	1079	901	973
ENSCAFG00845004431	0	0	0	2
ENSCAFG00845004432	0	0	0	0
ENSCAFG00845004433	0	2	0	0
ENSCAFG00845004434	0	0	0	0
ENSCAFG00845004435	1	6	0	6
ENSCAFG00845004436	312	290	240	257
ENSCAFG00845004437	1	0	0	1
ENSCAFG00845004438	0	0	0	0
ENSCAFG00845004439	117	81	67	79
ENSCAFG00845016419	0	0	0	0
ENSCAFG00845016418	7242	7068	6950	6744
ENSCAFG00845016417	763	813	758	794
ENSCAFG00845016416	1569	1555	1599	1568
ENSCAFG00845016415	582	496	773	765
ENSCAFG00845016414	0	0	1	2
ENSCAFG00845016413	6	0	1	3
ENSCAFG00845016412	0	0	0	0
ENSCAFG00845016400	537	503	483	535
ENSCAFG00845004420	0	0	0	0
ENSCAFG00845004421	15	16	7	5
ENSCAFG00845004422	70	92	64	70
ENSCAFG00845004423	0	0	0	0
ENSCAFG00845004424	53	47	44	42
ENSCAFG00845004425	0	1	0	0
ENSCAFG00845004426	0	0	0	0
ENSCAFG00845004427	1	1	0	0
ENSCAFG00845004428	0	0	0	0
ENSCAFG00845004429	0	0	0	0
ENSCAFG00845016409	0	0	0	0
ENSCAFG00845016408	3	0	0	8
ENSCAFG00845016407	0	0	0	0
ENSCAFG00845016406	0	0	0	0

ENSCAFG00845016405	0	0	0	0
ENSCAFG00845016404	309	305	340	346
ENSCAFG00845016403	3	2	0	0
ENSCAFG00845016402	178	196	119	111
ENSCAFG00845016401	1	0	0	1
ENSCAFG00845004410	1259	1215	1222	1154
ENSCAFG00845004411	0	0	0	0
ENSCAFG00845004412	1	0	0	0
ENSCAFG00845004413	1008	906	793	834
ENSCAFG00845004414	0	0	0	0
ENSCAFG00845004415	0	0	0	0
ENSCAFG00845004416	4	0	0	0
ENSCAFG00845004417	0	0	0	0
ENSCAFG00845004418	0	0	2	3
ENSCAFG00845004419	10	0	2	2
ENSCAFG00845004400	0	0	0	0
ENSCAFG00845004401	0	0	0	0
ENSCAFG00845004402	499	497	468	501
ENSCAFG00845004403	0	1	0	0
ENSCAFG00845004404	0	0	0	0
ENSCAFG00845004405	0	0	2	0
ENSCAFG00845004406	6	2	3	2
ENSCAFG00845004407	0	0	0	0
ENSCAFG00845004408	0	0	0	0
ENSCAFG00845004409	0	3	0	0
ENSCAFG00845028598	1520	1566	1486	1476
ENSCAFG00845028599	0	0	0	1
ENSCAFG00845028596	0	0	0	0
ENSCAFG00845028597	1230	1226	1246	1117
ENSCAFG00845028594	0	0	0	0
ENSCAFG00845028595	0	0	0	0
ENSCAFG00845028592	9	8	21	25
ENSCAFG00845028593	0	0	0	0
ENSCAFG00845028590	0	0	0	0
ENSCAFG00845028591	272	290	227	238
ENSCAFG00845028589	211	181	195	177
ENSCAFG00845028587	0	0	0	0
ENSCAFG00845028588	0	1	0	0
ENSCAFG00845028585	0	0	0	0
ENSCAFG00845028586	0	0	0	0
ENSCAFG00845028583	721	689	705	652

ENSCAFG00845028584	0	0	0	0
ENSCAFG00845028581	0	0	0	0
ENSCAFG00845028582	409	358	340	333
ENSCAFG00845028580	661	668	555	609
ENSCAFG00845016598	2	0	1	2
ENSCAFG00845016597	295	275	248	277
ENSCAFG00845016596	611	503	461	489
ENSCAFG00845016595	0	0	0	0
ENSCAFG00845016594	1171	1071	1013	1165
ENSCAFG00845016593	0	0	0	0
ENSCAFG00845016592	9	24	28	18
ENSCAFG00845016591	0	0	0	0
ENSCAFG00845016590	0	0	0	0
ENSCAFG00845028578	0	0	0	0
ENSCAFG00845028579	9	4	0	1
ENSCAFG00845028576	0	0	0	0
ENSCAFG00845028577	307	298	255	254
ENSCAFG00845028574	49	68	54	61
ENSCAFG00845028575	0	0	0	0
ENSCAFG00845028572	279	291	255	261
ENSCAFG00845028573	0	0	0	0
ENSCAFG00845028570	353	332	345	329
ENSCAFG00845028571	2	1	5	2
ENSCAFG00845016599	0	0	0	0
ENSCAFG00845016587	49	43	52	48
ENSCAFG00845016586	0	0	0	0
ENSCAFG00845016585	338	425	333	364
ENSCAFG00845016584	0	0	0	0
ENSCAFG00845016583	1	1	1	1
ENSCAFG00845016582	27	18	21	26
ENSCAFG00845016581	0	0	0	0
ENSCAFG00845016580	169	150	197	171
ENSCAFG00845028569	0	0	0	0
ENSCAFG00845028567	0	0	0	0
ENSCAFG00845028568	0	0	0	0
ENSCAFG00845028565	0	0	0	0
ENSCAFG00845028566	0	0	0	1
ENSCAFG00845028563	139	130	136	141
ENSCAFG00845028564	136	138	190	200
ENSCAFG00845028561	1	2	3	1
ENSCAFG00845028562	13	13	8	14

ENSCAFG00845028560	992	939	1050	1023
ENSCAFG00845016589	549	481	448	494
ENSCAFG00845016588	0	1	0	0
ENSCAFG00845016576	0	0	0	0
ENSCAFG00845016575	6	4	4	5
ENSCAFG00845016574	746	647	747	768
ENSCAFG00845016573	154	154	162	142
ENSCAFG00845016572	0	0	0	1
ENSCAFG00845016571	0	0	0	0
ENSCAFG00845016570	0	0	0	0
ENSCAFG00845004590	2919	3055	3013	3188
ENSCAFG00845004591	43	45	50	44
ENSCAFG00845004592	1	9	3	2
ENSCAFG00845004593	0	0	0	0
ENSCAFG00845004594	0	0	0	0
ENSCAFG00845028558	241	267	252	248
ENSCAFG00845004595	9	6	3	3
ENSCAFG00845028559	553	527	537	474
ENSCAFG00845004596	0	0	0	0
ENSCAFG00845028556	236	248	247	263
ENSCAFG00845004597	6	0	0	2
ENSCAFG00845028557	20	24	18	28
ENSCAFG00845004598	433	426	377	411
ENSCAFG00845028554	0	0	0	0
ENSCAFG00845004599	2941	2781	2574	2824
ENSCAFG00845028555	271	316	258	283
ENSCAFG00845028552	0	0	0	0
ENSCAFG00845028553	1	6	4	4
ENSCAFG00845028550	1	0	0	1
ENSCAFG00845028551	23	18	20	10
ENSCAFG00845016579	41	40	49	38
ENSCAFG00845016578	0	0	0	0
ENSCAFG00845016577	0	0	0	0
ENSCAFG00845016565	0	0	0	0
ENSCAFG00845016564	0	0	0	0
ENSCAFG00845016563	0	0	0	0
ENSCAFG00845016562	0	0	0	0
ENSCAFG00845016561	13	16	10	18
ENSCAFG00845016560	607	597	542	613
ENSCAFG00845004580	75	75	67	50
ENSCAFG00845004581	107	89	112	118

ENSCAFG00845028549	2193	1941	1985	1964
ENSCAFG00845004582	2	1	4	1
ENSCAFG00845004583	2	1	3	0
ENSCAFG00845028547	365	353	369	415
ENSCAFG00845004584	182	160	125	146
ENSCAFG00845028548	2073	2129	1886	2195
ENSCAFG00845004585	0	0	0	0
ENSCAFG00845028545	4	6	3	5
ENSCAFG00845004586	3	1	3	12
ENSCAFG00845028546	2	3	2	0
ENSCAFG00845004587	0	0	0	0
ENSCAFG00845028543	4	0	1	5
ENSCAFG00845004588	1176	1155	1109	1101
ENSCAFG00845028544	12	8	13	9
ENSCAFG00845004589	0	0	0	0
ENSCAFG00845028541	677	587	635	658
ENSCAFG00845028542	0	0	0	0
ENSCAFG00845028540	9	8	5	11
ENSCAFG00845016569	10	19	20	19
ENSCAFG00845016568	9	7	6	8
ENSCAFG00845016567	0	0	0	0
ENSCAFG00845016566	54	45	38	53
ENSCAFG00845016554	0	0	0	0
ENSCAFG00845016553	0	0	0	1
ENSCAFG00845016552	235	212	182	203
ENSCAFG00845016551	0	0	0	0
ENSCAFG00845016550	10	10	14	5
ENSCAFG00845004570	0	0	0	0
ENSCAFG00845028538	6518	6069	6575	6706
ENSCAFG00845004571	1	0	0	1
ENSCAFG00845028539	3290	3165	3286	3498
ENSCAFG00845004572	183	148	166	140
ENSCAFG00845028536	1	0	2	0
ENSCAFG00845004573	0	0	0	0
ENSCAFG00845028537	993	985	1024	965
ENSCAFG00845004574	3	6	0	1
ENSCAFG00845028534	637	685	683	621
ENSCAFG00845004575	602	527	533	564
ENSCAFG00845028535	1003	989	953	945
ENSCAFG00845004576	141	127	80	82
ENSCAFG00845028532	82	79	55	66

ENSCAFG00845004577	119	109	138	145
ENSCAFG00845028533	327	292	418	468
ENSCAFG00845004578	616	627	575	582
ENSCAFG00845028530	860	806	801	866
ENSCAFG00845004579	199	187	177	160
ENSCAFG00845028531	576	517	486	520
ENSCAFG00845016559	543	484	541	497
ENSCAFG00845016558	96	154	89	124
ENSCAFG00845016557	1129	1079	944	1092
ENSCAFG00845016556	1	0	0	0
ENSCAFG00845016555	12	12	28	15
ENSCAFG00845016543	40	44	25	33
ENSCAFG00845016542	13	9	4	4
ENSCAFG00845016541	2	1	5	0
ENSCAFG00845016540	0	0	0	0
ENSCAFG00845028529	0	0	0	0
ENSCAFG00845028527	245	276	351	303
ENSCAFG00845004560	367	414	377	407
ENSCAFG00845028528	0	0	0	0
ENSCAFG00845004561	438	472	451	487
ENSCAFG00845028525	275	262	230	261
ENSCAFG00845004562	9	1	3	6
ENSCAFG00845028526	375	337	326	347
ENSCAFG00845004563	0	0	0	0
ENSCAFG00845028523	874	817	792	971
ENSCAFG00845004564	0	0	0	0
ENSCAFG00845028524	154	154	138	141
ENSCAFG00845004565	195	175	206	170
ENSCAFG00845028521	24	38	33	10
ENSCAFG00845004566	0	0	0	0
ENSCAFG00845028522	0	0	0	0
ENSCAFG00845004567	1105	1078	1080	1073
ENSCAFG00845004568	33	22	28	28
ENSCAFG00845028520	613	583	549	551
ENSCAFG00845004569	0	0	0	0
ENSCAFG00845016549	0	0	0	0
ENSCAFG00845016548	324	311	316	331
ENSCAFG00845016547	2	1	0	2
ENSCAFG00845016546	0	0	0	0
ENSCAFG00845016545	2	1	0	0
ENSCAFG00845016544	693	666	700	658

ENSCAFG00845016532	12	9	18	12
ENSCAFG00845016531	4286	4163	3876	3739
ENSCAFG00845016530	0	0	0	0
ENSCAFG00845028518	0	0	0	0
ENSCAFG00845028519	1242	1221	1234	1241
ENSCAFG00845028516	0	0	1	0
ENSCAFG00845028517	0	0	1	0
ENSCAFG00845004550	514	527	519	536
ENSCAFG00845028514	9	0	6	2
ENSCAFG00845004551	25	18	16	11
ENSCAFG00845028515	0	0	0	0
ENSCAFG00845004552	0	0	0	0
ENSCAFG00845028512	0	0	0	0
ENSCAFG00845004553	3179	2975	2944	3014
ENSCAFG00845028513	0	0	0	0
ENSCAFG00845004554	104	89	95	105
ENSCAFG00845028510	408	480	501	562
ENSCAFG00845004555	33	20	13	21
ENSCAFG00845028511	32	44	41	59
ENSCAFG00845004556	261	266	260	279
ENSCAFG00845004557	0	0	0	0
ENSCAFG00845004558	50	41	31	39
ENSCAFG00845004559	0	0	0	0
ENSCAFG00845016539	0	0	0	0
ENSCAFG00845016538	2261	2334	2239	2188
ENSCAFG00845016537	3048	2816	3440	3487
ENSCAFG00845016536	343	299	328	325
ENSCAFG00845016535	1009	887	897	1009
ENSCAFG00845016534	0	0	0	0
ENSCAFG00845016533	1621	1571	1805	1826
ENSCAFG00845016521	1	1	0	0
ENSCAFG00845016520	5	1	5	1
ENSCAFG00845028509	39	40	24	45
ENSCAFG00845028507	0	0	0	0
ENSCAFG00845028508	1	0	0	0
ENSCAFG00845028505	605	661	811	843
ENSCAFG00845028506	589	611	551	539
ENSCAFG00845028503	162	169	144	132
ENSCAFG00845004540	0	0	0	0
ENSCAFG00845028504	11	6	6	7
ENSCAFG00845004541	1	2	4	2

ENSCAFG00845028501	5	9	4	12
ENSCAFG00845004542	0	0	0	0
ENSCAFG00845028502	240	243	278	296
ENSCAFG00845004543	168	124	129	125
ENSCAFG00845004544	744	748	613	635
ENSCAFG00845028500	11	15	11	13
ENSCAFG00845004545	25	41	17	18
ENSCAFG00845004546	0	0	0	0
ENSCAFG00845004547	4756	4426	4377	4378
ENSCAFG00845004548	0	0	0	0
ENSCAFG00845004549	3062	2904	3149	3281
ENSCAFG00845016529	649	681	671	757
ENSCAFG00845016528	0	0	0	0
ENSCAFG00845016527	0	0	0	0
ENSCAFG00845016526	1	0	0	1
ENSCAFG00845016525	0	0	0	0
ENSCAFG00845016524	0	1	1	2
ENSCAFG00845016523	0	0	0	0
ENSCAFG00845016522	1	0	0	1
ENSCAFG00845016510	0	0	0	0
ENSCAFG00845004530	1	1	4	4
ENSCAFG00845004531	0	0	0	0
ENSCAFG00845004532	0	0	0	0
ENSCAFG00845004533	0	0	0	0
ENSCAFG00845004534	0	0	0	0
ENSCAFG00845004535	5	12	11	9
ENSCAFG00845004536	4	6	9	5
ENSCAFG00845004537	0	0	0	0
ENSCAFG00845004538	0	1	2	0
ENSCAFG00845004539	1	0	0	0
ENSCAFG00845016519	0	0	2	0
ENSCAFG00845016518	0	0	0	0
ENSCAFG00845016517	9	7	14	5
ENSCAFG00845016516	1101	967	926	884
ENSCAFG00845016515	1980	1814	1912	2003
ENSCAFG00845016514	701	694	723	756
ENSCAFG00845016513	388	337	382	446
ENSCAFG00845016512	0	0	0	0
ENSCAFG00845016511	4	8	7	4
ENSCAFG00845004520	0	0	0	0
ENSCAFG00845004521	810	812	927	895

ENSCAFG00845004522	0	0	0	0
ENSCAFG00845004523	0	0	0	0
ENSCAFG00845004524	0	0	0	0
ENSCAFG00845004525	2714	2674	2608	2570
ENSCAFG00845004526	3	0	0	1
ENSCAFG00845004527	908	912	994	1025
ENSCAFG00845016509	830	768	730	666
ENSCAFG00845004528	0	0	0	0
ENSCAFG00845016508	0	0	0	0
ENSCAFG00845004529	0	0	0	0
ENSCAFG00845016507	0	0	0	0
ENSCAFG00845016506	6	3	6	14
ENSCAFG00845016505	1441	1327	1399	1316
ENSCAFG00845016504	1	0	0	0
ENSCAFG00845016503	3	3	4	3
ENSCAFG00845016502	0	0	0	0
ENSCAFG00845016501	0	0	0	0
ENSCAFG00845016500	32	24	11	22
ENSCAFG00845004510	84	56	46	30
ENSCAFG00845004511	0	0	0	0
ENSCAFG00845004512	0	0	0	0
ENSCAFG00845004513	2	2	1	10
ENSCAFG00845004514	0	0	0	0
ENSCAFG00845004515	0	0	0	0
ENSCAFG00845004516	12	3	2	2
ENSCAFG00845004517	1	1	0	1
ENSCAFG00845004518	0	0	0	0
ENSCAFG00845004519	0	0	0	0
ENSCAFG00845004500	0	0	0	0
ENSCAFG00845004501	0	1	0	0
ENSCAFG00845004502	0	0	0	0
ENSCAFG00845004503	0	0	0	0
ENSCAFG00845004504	0	0	0	0
ENSCAFG00845004505	1	0	2	0
ENSCAFG00845004506	0	0	0	0
ENSCAFG00845004507	0	0	0	0
ENSCAFG00845004508	0	3	1	0
ENSCAFG00845004509	0	0	0	0
ENSCAFG00845014089	0	0	0	0
ENSCAFG00845014087	726	691	673	659
ENSCAFG00845014088	3459	3243	2868	2906

ENSCAFG00845014085	9461	9016	9011	9291
ENSCAFG00845014086	0	0	0	0
ENSCAFG00845014083	748	747	761	739
ENSCAFG00845014084	1	1	1	3
ENSCAFG00845014081	0	0	0	0
ENSCAFG00845014082	2529	2554	2544	2638
ENSCAFG00845014080	0	0	0	0
ENSCAFG00845002099	535	434	532	511
ENSCAFG00845026066	7	16	11	20
ENSCAFG00845002098	0	0	0	0
ENSCAFG00845026069	0	0	1	0
ENSCAFG00845002097	3171	3076	3277	3216
ENSCAFG00845026068	0	0	3	0
ENSCAFG00845002096	17	5	2	3
ENSCAFG00845026063	0	0	0	0
ENSCAFG00845002095	456	430	418	404
ENSCAFG00845026062	185	200	164	176
ENSCAFG00845002094	1667	1649	1455	1357
ENSCAFG00845026065	2	4	1	1
ENSCAFG00845002093	63	46	39	43
ENSCAFG00845026064	480	502	458	414
ENSCAFG00845002092	1422	1348	1319	1223
ENSCAFG00845002091	0	0	0	3
ENSCAFG00845002090	922	859	863	828
ENSCAFG00845026061	1208	1058	1144	1278
ENSCAFG00845026060	308	249	230	243
ENSCAFG00845014078	0	0	0	0
ENSCAFG00845014079	0	0	2	0
ENSCAFG00845014076	4	4	2	9
ENSCAFG00845014077	0	0	0	0
ENSCAFG00845014074	1544	1530	1691	1821
ENSCAFG00845014075	0	0	0	0
ENSCAFG00845014072	35	45	42	34
ENSCAFG00845014073	959	957	912	880
ENSCAFG00845014070	1410	1356	1292	1280
ENSCAFG00845014071	7176	6718	6593	7034
ENSCAFG00845026059	12	13	10	16
ENSCAFG00845026056	0	0	0	0
ENSCAFG00845026055	13	11	12	16
ENSCAFG00845026058	4728	4496	4891	5055
ENSCAFG00845026057	0	0	0	0

ENSCAFG00845026052	214	231	165	179
ENSCAFG00845026051	349	319	396	428
ENSCAFG00845026054	1529	1565	1363	1337
ENSCAFG00845026053	1022	1001	1093	1167
ENSCAFG00845014067	0	0	0	0
ENSCAFG00845014068	0	0	0	0
ENSCAFG00845014065	1836	1762	1662	1692
ENSCAFG00845014066	1042	954	971	951
ENSCAFG00845014063	1243	1109	1159	1174
ENSCAFG00845014064	494	472	541	484
ENSCAFG00845014061	0	0	0	0
ENSCAFG00845014062	9	6	12	5
ENSCAFG00845014060	2040	1933	1933	1845
ENSCAFG00845026049	4	13	9	2
ENSCAFG00845026048	974	940	940	971
ENSCAFG00845002079	0	0	0	0
ENSCAFG00845002078	0	0	0	0
ENSCAFG00845026045	16	33	16	27
ENSCAFG00845002077	0	0	0	0
ENSCAFG00845026044	909	850	900	927
ENSCAFG00845002076	483	424	383	366
ENSCAFG00845026047	80	74	79	87
ENSCAFG00845002075	0	0	0	0
ENSCAFG00845026046	1346	1355	1444	1410
ENSCAFG00845002074	0	0	0	1
ENSCAFG00845026041	0	0	0	0
ENSCAFG00845002073	1324	1177	1267	1319
ENSCAFG00845026040	0	0	0	0
ENSCAFG00845002072	0	0	0	0
ENSCAFG00845002071	0	0	0	0
ENSCAFG00845026042	0	0	0	0
ENSCAFG00845002070	1193	1182	954	946
ENSCAFG00845014069	1548	1512	1372	1353
ENSCAFG00845014056	431	385	390	353
ENSCAFG00845014057	0	0	0	0
ENSCAFG00845014054	1	0	0	3
ENSCAFG00845014055	2066	1974	1914	2112
ENSCAFG00845014052	0	0	0	0
ENSCAFG00845014053	64	57	49	33
ENSCAFG00845014050	2541	2566	2378	2427
ENSCAFG00845014051	0	0	0	0

ENSCAFG00845026038	90	86	81	50
ENSCAFG00845026037	0	0	0	0
ENSCAFG00845028699	0	0	0	0
ENSCAFG00845026039	0	0	0	0
ENSCAFG00845002089	4657	4553	4670	4799
ENSCAFG00845026034	0	0	0	0
ENSCAFG00845028697	1427	1440	1341	1313
ENSCAFG00845002088	0	1	0	0
ENSCAFG00845026033	1	0	0	0
ENSCAFG00845028698	36	35	43	43
ENSCAFG00845002087	0	0	0	0
ENSCAFG00845026036	0	0	0	0
ENSCAFG00845028695	0	0	0	0
ENSCAFG00845002086	3	4	0	4
ENSCAFG00845026035	19	12	9	13
ENSCAFG00845028696	1188	1173	1026	925
ENSCAFG00845002085	31	23	22	15
ENSCAFG00845026030	560	542	634	566
ENSCAFG00845028693	0	0	1	1
ENSCAFG00845002084	1267	1249	1069	1165
ENSCAFG00845028694	2728	2744	2729	2857
ENSCAFG00845002083	114	118	106	85
ENSCAFG00845026032	0	0	0	0
ENSCAFG00845028691	304	277	297	316
ENSCAFG00845002082	2106	1948	2187	2211
ENSCAFG00845026031	0	1	0	0
ENSCAFG00845028692	0	0	0	0
ENSCAFG00845002081	43	36	23	26
ENSCAFG00845002080	718	696	689	671
ENSCAFG00845028690	668	695	732	688
ENSCAFG00845014058	0	0	0	0
ENSCAFG00845014059	253	235	265	262
ENSCAFG00845014045	0	0	0	0
ENSCAFG00845014046	0	0	0	0
ENSCAFG00845014043	36	25	22	14
ENSCAFG00845014044	50	61	52	38
ENSCAFG00845014041	0	0	6	9
ENSCAFG00845014042	0	0	0	0
ENSCAFG00845014040	518	474	627	705
ENSCAFG00845026027	199	192	158	201
ENSCAFG00845002059	0	0	0	0

ENSCAFG00845026026	58	72	61	62
ENSCAFG00845002058	2	0	0	0
ENSCAFG00845026029	953	944	839	856
ENSCAFG00845028688	0	0	0	0
ENSCAFG00845002057	0	0	0	0
ENSCAFG00845026028	0	0	0	0
ENSCAFG00845028689	865	869	863	936
ENSCAFG00845002056	4	8	15	6
ENSCAFG00845026023	4	3	2	3
ENSCAFG00845028686	5	2	8	7
ENSCAFG00845002055	0	0	0	0
ENSCAFG00845026022	0	0	0	0
ENSCAFG00845028687	0	0	0	0
ENSCAFG00845002054	190	192	183	211
ENSCAFG00845026025	711	824	913	877
ENSCAFG00845028684	0	0	0	0
ENSCAFG00845002053	0	0	0	0
ENSCAFG00845026024	1357	1317	1340	1346
ENSCAFG00845028685	0	0	0	0
ENSCAFG00845002052	0	0	0	0
ENSCAFG00845028682	0	0	0	0
ENSCAFG00845002051	334	311	273	293
ENSCAFG00845028683	0	0	0	0
ENSCAFG00845002050	331	343	366	317
ENSCAFG00845026021	0	0	0	0
ENSCAFG00845028680	1945	1729	2078	2112
ENSCAFG00845026020	2995	2994	2870	2771
ENSCAFG00845028681	335	308	379	379
ENSCAFG00845014049	2	3	0	3
ENSCAFG00845014047	0	0	0	0
ENSCAFG00845014048	158	143	162	139
ENSCAFG00845014034	9	14	6	5
ENSCAFG00845016697	0	0	0	0
ENSCAFG00845014035	2	5	4	1
ENSCAFG00845016696	0	0	1	0
ENSCAFG00845014032	0	0	0	0
ENSCAFG00845016695	53	29	55	32
ENSCAFG00845014033	675	679	657	685
ENSCAFG00845016694	366	365	312	373
ENSCAFG00845014030	1726	1658	1574	1657
ENSCAFG00845016693	0	0	0	0

ENSCAFG00845014031	0	0	0	0
ENSCAFG00845016692	0	0	0	0
ENSCAFG00845016691	3	0	6	2
ENSCAFG00845016690	0	0	0	0
ENSCAFG00845026019	0	0	0	0
ENSCAFG00845026016	0	0	0	0
ENSCAFG00845028679	573	525	553	582
ENSCAFG00845026015	1	2	1	0
ENSCAFG00845002069	997	907	875	854
ENSCAFG00845026018	0	0	1	0
ENSCAFG00845028677	0	0	0	0
ENSCAFG00845002068	0	0	0	0
ENSCAFG00845026017	0	0	0	0
ENSCAFG00845028678	575	587	573	696
ENSCAFG00845002067	683	677	654	637
ENSCAFG00845026012	1	0	0	0
ENSCAFG00845028675	724	742	946	780
ENSCAFG00845002066	112	116	89	81
ENSCAFG00845026011	0	0	0	0
ENSCAFG00845028676	1425	1394	1422	1434
ENSCAFG00845002065	30	22	24	35
ENSCAFG00845026014	0	0	0	0
ENSCAFG00845028673	0	0	0	0
ENSCAFG00845002064	0	0	0	0
ENSCAFG00845026013	0	0	0	0
ENSCAFG00845028674	0	0	0	0
ENSCAFG00845002063	0	1	0	0
ENSCAFG00845028671	0	0	0	0
ENSCAFG00845002062	1077	1124	960	950
ENSCAFG00845028672	221	198	192	167
ENSCAFG00845002061	557	591	468	534
ENSCAFG00845026010	30	32	23	23
ENSCAFG00845002060	6	4	6	4
ENSCAFG00845028670	260	314	254	281
ENSCAFG00845014038	0	0	0	0
ENSCAFG00845014039	612	579	549	559
ENSCAFG00845014036	8	4	2	1
ENSCAFG00845016699	0	0	0	0
ENSCAFG00845014037	0	0	0	0
ENSCAFG00845016698	0	0	0	0
ENSCAFG00845014023	2075	1955	1625	1740

ENSCAFG00845016686	275	249	331	309
ENSCAFG00845014024	3907	3854	3595	3438
ENSCAFG00845016685	726	689	718	739
ENSCAFG00845014021	7	8	3	2
ENSCAFG00845016684	31	27	31	25
ENSCAFG00845014022	1516	1433	1471	1444
ENSCAFG00845016683	1762	1836	1624	1695
ENSCAFG00845016682	0	0	0	0
ENSCAFG00845014020	1	1	4	4
ENSCAFG00845016681	0	0	0	0
ENSCAFG00845016680	0	0	0	0
ENSCAFG00845026009	158	192	129	147
ENSCAFG00845026008	7	4	8	4
ENSCAFG00845002039	583	639	524	590
ENSCAFG00845002038	0	0	0	0
ENSCAFG00845026005	3704	3578	3450	3650
ENSCAFG00845028668	0	0	0	0
ENSCAFG00845002037	969	1022	906	859
ENSCAFG00845026004	0	0	1	1
ENSCAFG00845028669	1839	1736	2149	2088
ENSCAFG00845002036	26	13	27	32
ENSCAFG00845028666	0	0	0	0
ENSCAFG00845002035	0	0	0	0
ENSCAFG00845026006	0	0	0	0
ENSCAFG00845028667	0	0	0	0
ENSCAFG00845002034	3671	3234	3208	3100
ENSCAFG00845026001	447	446	510	501
ENSCAFG00845028664	12	9	18	14
ENSCAFG00845002033	713	575	627	658
ENSCAFG00845026000	44	61	43	48
ENSCAFG00845028665	1330	1254	1137	1172
ENSCAFG00845002032	246	240	228	234
ENSCAFG00845026003	0	0	0	0
ENSCAFG00845028662	0	0	0	0
ENSCAFG00845002031	0	0	0	0
ENSCAFG00845026002	0	0	0	0
ENSCAFG00845028663	10959	10802	11878	11516
ENSCAFG00845002030	6	4	8	9
ENSCAFG00845028660	0	0	0	0
ENSCAFG00845028661	218	190	230	306
ENSCAFG00845014029	1	0	0	0

ENSCAFG00845014027	0	0	2	0
ENSCAFG00845014028	199	195	224	206
ENSCAFG00845016689	0	0	0	0
ENSCAFG00845014025	3	4	1	3
ENSCAFG00845016688	418	445	460	425
ENSCAFG00845014026	0	2	0	2
ENSCAFG00845016687	0	0	0	0
ENSCAFG00845014012	72	57	73	71
ENSCAFG00845016675	0	0	0	0
ENSCAFG00845014013	136	144	137	113
ENSCAFG00845016674	64	76	67	45
ENSCAFG00845014010	0	0	0	0
ENSCAFG00845016673	162	130	98	94
ENSCAFG00845014011	738	789	796	767
ENSCAFG00845016672	1	0	3	4
ENSCAFG00845016671	0	0	0	0
ENSCAFG00845016670	0	0	0	0
ENSCAFG00845004690	864	811	725	807
ENSCAFG00845004691	0	0	0	0
ENSCAFG00845028659	66	72	70	76
ENSCAFG00845004692	1413	1245	1377	1473
ENSCAFG00845002049	572	569	546	580
ENSCAFG00845004693	2	0	1	0
ENSCAFG00845028657	63	45	57	59
ENSCAFG00845002048	2	0	1	1
ENSCAFG00845004694	0	0	0	0
ENSCAFG00845028658	1	1	7	7
ENSCAFG00845002047	467	464	408	365
ENSCAFG00845004695	97	133	102	107
ENSCAFG00845028655	2846	2565	3047	3186
ENSCAFG00845002046	0	0	0	0
ENSCAFG00845004696	0	0	0	0
ENSCAFG00845028656	0	0	0	0
ENSCAFG00845002045	40	21	29	14
ENSCAFG00845004697	1457	1383	1349	1290
ENSCAFG00845028653	0	0	0	0
ENSCAFG00845002044	1073	1039	872	932
ENSCAFG00845004698	970	969	938	997
ENSCAFG00845028654	0	0	0	0
ENSCAFG00845002043	603	558	671	672
ENSCAFG00845004699	1717	1739	1538	1614

ENSCAFG00845028651	0	0	0	0
ENSCAFG00845002042	0	0	0	0
ENSCAFG00845028652	14	16	6	16
ENSCAFG00845002041	2	1	1	1
ENSCAFG00845002040	296	261	226	234
ENSCAFG00845028650	205	203	245	207
ENSCAFG00845014018	0	0	0	0
ENSCAFG00845014019	0	0	0	0
ENSCAFG00845014016	31	28	44	40
ENSCAFG00845016679	1257	1150	1097	1201
ENSCAFG00845014017	5	1	1	3
ENSCAFG00845016678	0	0	0	0
ENSCAFG00845014014	0	0	0	0
ENSCAFG00845014015	628	579	564	579
ENSCAFG00845016676	496	457	425	482
ENSCAFG00845026099	809	833	807	816
ENSCAFG00845026096	0	0	0	0
ENSCAFG00845026095	3	4	0	1
ENSCAFG00845026098	177	137	153	176
ENSCAFG00845026097	35	22	28	34
ENSCAFG00845026092	0	0	0	0
ENSCAFG00845026091	0	0	0	0
ENSCAFG00845026094	408	420	379	397
ENSCAFG00845026093	137	128	100	105
ENSCAFG00845026090	0	0	0	0
ENSCAFG00845026089	0	0	0	0
ENSCAFG00845026088	1743	1752	1917	1994
ENSCAFG00845026084	0	0	0	0
ENSCAFG00845026087	0	0	0	0
ENSCAFG00845026086	0	0	0	0
ENSCAFG00845026081	861	814	801	832
ENSCAFG00845026080	0	0	0	0
ENSCAFG00845026083	0	0	0	0
ENSCAFG00845026082	0	0	0	0
ENSCAFG00845014098	678	726	586	578
ENSCAFG00845014099	172	167	203	222
ENSCAFG00845014096	267	244	201	234
ENSCAFG00845014097	64	73	56	77
ENSCAFG00845014094	2	0	4	3
ENSCAFG00845014095	0	0	0	0
ENSCAFG00845014092	349	298	329	304

ENSCAFG00845014093	36	29	24	23
ENSCAFG00845014090	77	74	56	70
ENSCAFG00845014091	0	0	0	0
ENSCAFG00845026078	0	0	0	0
ENSCAFG00845026079	3	11	1	2
ENSCAFG00845026073	26	23	17	22
ENSCAFG00845026076	1656	1805	1668	1542
ENSCAFG00845026075	4	7	9	5
ENSCAFG00845026070	0	0	0	0
ENSCAFG00845026072	7	9	6	5
ENSCAFG00845026071	110	104	118	115
ENSCAFG00845004600	143	140	146	155
ENSCAFG00845004601	0	0	4	4
ENSCAFG00845004602	381	400	375	428
ENSCAFG00845004603	1648	1561	1476	1545
ENSCAFG00845004604	7379	7066	7166	7554
ENSCAFG00845004605	158	124	184	172
ENSCAFG00845004606	1031	1070	1062	1054
ENSCAFG00845004607	0	0	0	0
ENSCAFG00845004608	12	5	5	12
ENSCAFG00845004609	20	18	10	12
ENSCAFG00845014001	624	578	504	495
ENSCAFG00845016664	83	82	88	84
ENSCAFG00845014002	0	0	0	0
ENSCAFG00845016663	0	0	0	0
ENSCAFG00845016662	1	0	0	0
ENSCAFG00845014000	44	36	44	42
ENSCAFG00845016661	1016	940	854	914
ENSCAFG00845016660	0	0	0	0
ENSCAFG00845002019	0	0	0	0
ENSCAFG00845002018	42	26	42	67
ENSCAFG00845004680	0	3	1	5
ENSCAFG00845028648	181	213	205	199
ENSCAFG00845002017	0	1	0	0
ENSCAFG00845004681	1958	1926	1908	1826
ENSCAFG00845028649	1236	1109	1296	1288
ENSCAFG00845002016	0	0	0	0
ENSCAFG00845004682	334	343	307	321
ENSCAFG00845028646	6791	6492	5691	5501
ENSCAFG00845002015	330	311	258	241
ENSCAFG00845004683	0	0	0	0

ENSCAFG00845028647	0	0	0	0
ENSCAFG00845002014	6027	6049	5670	5907
ENSCAFG00845004684	0	0	0	0
ENSCAFG00845028644	1	3	1	1
ENSCAFG00845002013	1	1	3	2
ENSCAFG00845004685	8	21	4	11
ENSCAFG00845028645	0	2	0	0
ENSCAFG00845002012	0	0	0	0
ENSCAFG00845004686	4146	3898	3679	3537
ENSCAFG00845028642	30	34	29	26
ENSCAFG00845002011	1805	1764	1753	1757
ENSCAFG00845004687	1310	1280	1386	1519
ENSCAFG00845028643	0	0	0	0
ENSCAFG00845002010	0	0	0	1
ENSCAFG00845004688	1110	1080	1072	1085
ENSCAFG00845028640	979	994	905	863
ENSCAFG00845004689	0	0	0	0
ENSCAFG00845028641	0	3	0	1
ENSCAFG00845014009	30	43	35	45
ENSCAFG00845014007	6	5	7	7
ENSCAFG00845014008	1310	1268	1145	1151
ENSCAFG00845016669	338	342	355	328
ENSCAFG00845014005	0	1	2	0
ENSCAFG00845016668	293	257	246	259
ENSCAFG00845014006	1	0	0	0
ENSCAFG00845016667	0	0	1	0
ENSCAFG00845014003	434	416	371	390
ENSCAFG00845016666	1	3	2	0
ENSCAFG00845014004	5	1	1	3
ENSCAFG00845016665	0	0	0	0
ENSCAFG00845016653	1035	945	931	975
ENSCAFG00845016652	481	472	471	458
ENSCAFG00845016651	0	0	0	0
ENSCAFG00845016650	0	0	0	0
ENSCAFG00845028639	9	11	9	5
ENSCAFG00845002029	5	4	4	4
ENSCAFG00845028637	2	2	4	0
ENSCAFG00845002028	6	21	13	18
ENSCAFG00845004670	30	22	31	25
ENSCAFG00845028638	0	0	1	1
ENSCAFG00845002027	4	1	6	1

ENSCAFG00845004671	459	462	438	477
ENSCAFG00845028635	0	1	6	0
ENSCAFG00845002026	16	19	25	18
ENSCAFG00845004672	192	243	161	178
ENSCAFG00845028636	0	2	6	0
ENSCAFG00845002025	1170	1126	848	825
ENSCAFG00845004673	658	633	541	563
ENSCAFG00845028633	5	10	22	14
ENSCAFG00845002024	0	0	0	0
ENSCAFG00845004674	280	310	292	278
ENSCAFG00845028634	2	0	3	0
ENSCAFG00845002023	0	0	0	0
ENSCAFG00845004675	1219	1139	1252	1181
ENSCAFG00845028631	6	0	5	5
ENSCAFG00845002022	2	0	0	0
ENSCAFG00845004676	0	0	0	0
ENSCAFG00845028632	7670	7215	6284	6319
ENSCAFG00845002021	1322	1269	1234	1238
ENSCAFG00845004677	4	8	3	3
ENSCAFG00845002020	3	17	10	7
ENSCAFG00845004678	0	0	0	0
ENSCAFG00845028630	0	0	0	0
ENSCAFG00845004679	0	0	0	0
ENSCAFG00845016659	26	22	9	15
ENSCAFG00845016658	0	0	0	0
ENSCAFG00845016657	2	0	0	0
ENSCAFG00845016656	0	0	0	0
ENSCAFG00845016655	0	0	0	0
ENSCAFG00845016654	52	48	42	52
ENSCAFG00845016642	200	216	158	176
ENSCAFG00845016640	883	876	821	847
ENSCAFG00845028628	20	34	28	24
ENSCAFG00845028629	1	2	2	0
ENSCAFG00845028626	6394	6192	5956	6356
ENSCAFG00845028627	8864	8599	8701	9070
ENSCAFG00845004660	4	0	0	0
ENSCAFG00845028624	1434	1520	1562	1565
ENSCAFG00845004661	5	14	2	4
ENSCAFG00845028625	6	7	0	6
ENSCAFG00845004662	2237	2156	2120	1997
ENSCAFG00845028622	2557	2694	2440	2633

ENSCAFG00845004663	0	0	0	0
ENSCAFG00845028623	196	174	185	176
ENSCAFG00845004664	0	0	0	0
ENSCAFG00845028620	350	356	384	382
ENSCAFG00845004665	0	0	1	0
ENSCAFG00845028621	130	129	103	119
ENSCAFG00845004666	40	61	62	46
ENSCAFG00845004667	660	713	622	660
ENSCAFG00845004668	628	642	568	655
ENSCAFG00845004669	0	0	0	0
ENSCAFG00845016649	10713	10371	6552	6355
ENSCAFG00845016648	3	3	1	1
ENSCAFG00845016647	0	0	0	0
ENSCAFG00845016646	0	0	0	0
ENSCAFG00845016645	0	0	0	0
ENSCAFG00845016644	0	0	0	0
ENSCAFG00845016643	671	667	722	714
ENSCAFG00845016631	0	0	0	0
ENSCAFG00845016630	152	145	171	127
ENSCAFG00845028619	2	2	3	6
ENSCAFG00845002009	856	815	744	677
ENSCAFG00845028617	5	0	2	1
ENSCAFG00845002008	493	422	553	530
ENSCAFG00845028618	835	742	708	719
ENSCAFG00845002007	3	1	0	0
ENSCAFG00845028615	66	64	43	67
ENSCAFG00845002006	1327	1264	1106	1052
ENSCAFG00845028616	1	1	0	0
ENSCAFG00845002005	866	810	820	886
ENSCAFG00845028613	0	0	0	2
ENSCAFG00845002004	3	0	2	0
ENSCAFG00845004650	0	0	0	0
ENSCAFG00845028614	205	209	166	188
ENSCAFG00845002003	0	2	0	0
ENSCAFG00845004651	6927	6702	6144	6615
ENSCAFG00845028611	1	0	0	0
ENSCAFG00845002002	0	0	0	0
ENSCAFG00845004652	1868	1632	1802	1845
ENSCAFG00845028612	0	1	2	1
ENSCAFG00845002001	0	0	0	0
ENSCAFG00845004653	0	0	0	0

ENSCAFG00845002000	0	0	0	0
ENSCAFG00845004654	20	19	29	33
ENSCAFG00845028610	5	4	11	17
ENSCAFG00845004655	0	0	0	0
ENSCAFG00845004656	0	0	0	0
ENSCAFG00845004657	24	24	30	17
ENSCAFG00845004658	6	6	6	3
ENSCAFG00845004659	0	0	0	0
ENSCAFG00845016639	525	470	490	541
ENSCAFG00845016638	0	0	0	0
ENSCAFG00845016637	0	0	0	0
ENSCAFG00845016636	577	521	513	562
ENSCAFG00845016635	0	0	0	3
ENSCAFG00845016634	0	0	0	0
ENSCAFG00845016633	195	218	224	177
ENSCAFG00845016632	17	18	22	9
ENSCAFG00845016620	0	0	0	0
ENSCAFG00845028608	307	280	230	299
ENSCAFG00845028609	253	221	332	312
ENSCAFG00845028606	176	157	224	207
ENSCAFG00845028607	0	0	1	1
ENSCAFG00845028604	0	0	0	0
ENSCAFG00845028605	0	0	0	0
ENSCAFG00845028602	198	190	206	197
ENSCAFG00845028603	0	0	0	0
ENSCAFG00845004640	255	293	272	249
ENSCAFG00845028600	0	0	0	0
ENSCAFG00845004641	8	14	22	12
ENSCAFG00845028601	0	0	0	0
ENSCAFG00845004642	0	0	0	0
ENSCAFG00845004643	120	91	92	114
ENSCAFG00845004644	6	3	6	1
ENSCAFG00845004645	3	1	4	6
ENSCAFG00845004646	0	0	0	0
ENSCAFG00845004647	7607	7632	8289	8362
ENSCAFG00845004648	0	0	0	0
ENSCAFG00845004649	63	52	46	76
ENSCAFG00845016629	0	0	0	0
ENSCAFG00845016628	0	0	0	0
ENSCAFG00845016627	0	0	0	0
ENSCAFG00845016626	637	612	472	509

ENSCAFG00845016625	422	411	330	310
ENSCAFG00845016624	9	11	5	7
ENSCAFG00845016623	0	0	0	0
ENSCAFG00845016622	728	633	636	662
ENSCAFG00845016621	0	0	0	0
ENSCAFG00845004630	1	4	0	2
ENSCAFG00845004631	7	2	0	5
ENSCAFG00845004632	1162	1164	1088	1104
ENSCAFG00845004633	647	575	517	491
ENSCAFG00845004634	0	1	0	3
ENSCAFG00845004635	0	0	0	0
ENSCAFG00845004636	0	0	1	3
ENSCAFG00845004637	0	0	0	0
ENSCAFG00845016619	74	49	57	41
ENSCAFG00845004638	15	9	6	6
ENSCAFG00845016618	176	187	151	152
ENSCAFG00845004639	0	0	0	0
ENSCAFG00845016617	0	0	0	0
ENSCAFG00845016616	0	0	0	0
ENSCAFG00845016615	667	639	784	748
ENSCAFG00845016614	359	332	338	275
ENSCAFG00845016613	0	0	0	0
ENSCAFG00845016612	35	45	34	93
ENSCAFG00845016611	2	3	1	3
ENSCAFG00845016610	0	0	0	0
ENSCAFG00845004620	55	50	68	56
ENSCAFG00845004621	192	180	216	221
ENSCAFG00845004622	557	478	467	420
ENSCAFG00845004623	141	154	113	129
ENSCAFG00845004624	486	463	439	536
ENSCAFG00845004625	0	0	0	0
ENSCAFG00845016609	65	63	62	68
ENSCAFG00845004626	53	48	43	44
ENSCAFG00845016608	0	0	0	0
ENSCAFG00845004627	0	0	0	0
ENSCAFG00845016607	0	0	0	0
ENSCAFG00845004628	3257	3088	2958	2948
ENSCAFG00845016606	14	12	28	21
ENSCAFG00845004629	1337	1336	1308	1374
ENSCAFG00845016605	0	0	0	0
ENSCAFG00845016604	869	805	814	827

ENSCAFG00845016603	0	0	0	0
ENSCAFG00845016602	0	0	0	0
ENSCAFG00845016601	541	584	603	519
ENSCAFG00845016600	177	148	251	216
ENSCAFG00845004610	2	0	0	1
ENSCAFG00845004611	145	129	173	154
ENSCAFG00845004612	0	0	0	0
ENSCAFG00845004613	5	6	4	3
ENSCAFG00845004614	0	2	0	1
ENSCAFG00845004615	263	314	269	256
ENSCAFG00845004616	1183	1094	1209	1234
ENSCAFG00845004617	54	47	50	52
ENSCAFG00845004618	12	24	8	7
ENSCAFG00845004619	4	0	4	4
ENSCAFG00845026188	1515	1514	1196	1109
ENSCAFG00845026187	0	0	0	1
ENSCAFG00845026189	0	0	0	0
ENSCAFG00845026183	0	0	0	0
ENSCAFG00845026185	0	0	0	0
ENSCAFG00845026180	575	628	626	590
ENSCAFG00845026182	1318	1121	1120	1154
ENSCAFG00845026181	1387	1394	1503	1579
ENSCAFG00845014199	2042	1894	1862	1984
ENSCAFG00845014197	253	244	232	220
ENSCAFG00845014198	0	0	0	0
ENSCAFG00845014195	0	0	0	0
ENSCAFG00845014196	4425	4497	3680	3615
ENSCAFG00845014193	8	2	10	3
ENSCAFG00845014194	0	0	0	0
ENSCAFG00845014191	34	30	23	17
ENSCAFG00845014192	12251	11513	11180	11091
ENSCAFG00845014190	0	0	0	0
ENSCAFG00845026177	275	262	244	229
ENSCAFG00845026176	802	758	748	775
ENSCAFG00845026179	197	196	221	221
ENSCAFG00845026178	21	6	11	15
ENSCAFG00845026173	232	220	170	197
ENSCAFG00845026172	1	0	1	0
ENSCAFG00845026175	2936	2957	3191	3174
ENSCAFG00845026174	969	933	960	996
ENSCAFG00845026170	3882	3780	3696	3695

ENSCAFG00845014188	32	22	13	25
ENSCAFG00845014189	0	0	0	0
ENSCAFG00845014186	157	116	103	106
ENSCAFG00845014187	0	0	0	0
ENSCAFG00845014184	0	0	0	0
ENSCAFG00845014185	759	763	769	852
ENSCAFG00845014182	0	0	0	0
ENSCAFG00845014183	845	760	716	729
ENSCAFG00845014180	1650	1660	1443	1425
ENSCAFG00845014181	0	0	0	0
ENSCAFG00845026169	0	0	0	0
ENSCAFG00845002199	82	66	84	95
ENSCAFG00845026166	343	247	302	287
ENSCAFG00845002198	0	0	0	0
ENSCAFG00845026165	636	581	633	638
ENSCAFG00845002197	249	307	263	260
ENSCAFG00845026168	0	0	0	0
ENSCAFG00845002196	27	27	23	24
ENSCAFG00845026167	0	0	0	0
ENSCAFG00845002195	26	32	13	23
ENSCAFG00845026162	7	2	7	11
ENSCAFG00845002194	2	0	0	1
ENSCAFG00845026161	10	9	3	1
ENSCAFG00845002193	129	167	132	106
ENSCAFG00845026164	0	3	2	4
ENSCAFG00845002192	906	831	671	700
ENSCAFG00845026163	1	0	0	2
ENSCAFG00845002191	1088	995	981	957
ENSCAFG00845002190	24	23	36	19
ENSCAFG00845014177	1	0	0	0
ENSCAFG00845014178	0	0	0	0
ENSCAFG00845014175	1	0	0	0
ENSCAFG00845014176	0	0	0	0
ENSCAFG00845014173	190	149	153	144
ENSCAFG00845014174	0	0	0	0
ENSCAFG00845014171	1952	1814	1869	1915
ENSCAFG00845014172	0	0	0	0
ENSCAFG00845014170	0	0	0	0
ENSCAFG00845026159	0	0	0	0
ENSCAFG00845026158	15	11	32	22
ENSCAFG00845026155	179	198	205	205

ENSCAFG00845026154	0	0	0	0
ENSCAFG00845026157	11	4	6	6
ENSCAFG00845026156	0	0	0	0
ENSCAFG00845026151	0	0	0	0
ENSCAFG00845026150	313	327	261	271
ENSCAFG00845026153	430	433	537	526
ENSCAFG00845026152	78	48	58	48
ENSCAFG00845014179	1290	1169	1436	1446
ENSCAFG00845014166	700	683	676	710
ENSCAFG00845014167	917	833	823	829
ENSCAFG00845014164	14	8	12	8
ENSCAFG00845014165	6	4	14	17
ENSCAFG00845014162	474	507	506	467
ENSCAFG00845014163	833	745	786	685
ENSCAFG00845014160	0	0	0	0
ENSCAFG00845014161	2	4	2	0
ENSCAFG00845026148	32	24	24	29
ENSCAFG00845026147	0	0	0	0
ENSCAFG00845002179	1729	1633	1542	1526
ENSCAFG00845002178	1778	1775	1772	1849
ENSCAFG00845026149	0	0	0	0
ENSCAFG00845002177	811	757	865	868
ENSCAFG00845026144	0	1	0	0
ENSCAFG00845002176	0	0	0	0
ENSCAFG00845026143	0	0	0	0
ENSCAFG00845002175	0	1	0	0
ENSCAFG00845026146	3	3	9	2
ENSCAFG00845002174	650	665	715	670
ENSCAFG00845026145	36	31	28	31
ENSCAFG00845002173	29	23	34	26
ENSCAFG00845026140	0	0	0	0
ENSCAFG00845002172	499	486	586	636
ENSCAFG00845002171	0	0	0	0
ENSCAFG00845026142	2825	2942	2820	2846
ENSCAFG00845002170	149	155	214	205
ENSCAFG00845026141	3390	2909	3554	3704
ENSCAFG00845014168	0	0	0	0
ENSCAFG00845014169	2	0	1	0
ENSCAFG00845014155	17	15	13	21
ENSCAFG00845014156	59	43	53	59
ENSCAFG00845014153	0	0	0	0

ENSCAFG00845014154	269	277	253	260
ENSCAFG00845014151	1581	1515	1561	1496
ENSCAFG00845014152	1044	913	842	899
ENSCAFG00845014150	49	54	55	48
ENSCAFG00845026137	0	0	0	0
ENSCAFG00845026136	2	2	0	4
ENSCAFG00845026139	147	138	162	168
ENSCAFG00845028798	467	470	553	571
ENSCAFG00845002189	349	306	280	319
ENSCAFG00845026138	1073	962	908	885
ENSCAFG00845028799	0	0	0	0
ENSCAFG00845002188	3	0	4	1
ENSCAFG00845026133	25	26	21	20
ENSCAFG00845028796	1996	2010	1745	1814
ENSCAFG00845002187	4	0	1	1
ENSCAFG00845026132	13	15	16	9
ENSCAFG00845028797	0	0	0	0
ENSCAFG00845002186	0	0	0	0
ENSCAFG00845026135	0	0	0	0
ENSCAFG00845028794	4	12	6	8
ENSCAFG00845002185	0	0	0	0
ENSCAFG00845028795	303	327	347	341
ENSCAFG00845002184	0	0	0	0
ENSCAFG00845028792	618	596	554	568
ENSCAFG00845002183	21	33	30	25
ENSCAFG00845028793	0	0	0	0
ENSCAFG00845002182	481	527	497	467
ENSCAFG00845026131	0	0	0	0
ENSCAFG00845028790	2	1	5	0
ENSCAFG00845002181	0	0	0	0
ENSCAFG00845026130	297	298	328	339
ENSCAFG00845028791	2409	2100	2298	2539
ENSCAFG00845002180	0	0	0	0
ENSCAFG00845014159	10	10	9	13
ENSCAFG00845014157	570	604	469	537
ENSCAFG00845014158	0	0	0	0
ENSCAFG00845014144	0	0	0	0
ENSCAFG00845014145	910	900	783	765
ENSCAFG00845014142	909	856	793	837
ENSCAFG00845014143	783	766	678	768
ENSCAFG00845014140	218	230	191	207

ENSCAFG00845014141	0	0	0	1
ENSCAFG00845002159	76	85	120	89
ENSCAFG00845026126	0	0	0	0
ENSCAFG00845028789	2350	2224	2366	2397
ENSCAFG00845002158	2	3	0	0
ENSCAFG00845026125	1	0	0	0
ENSCAFG00845002157	1	0	0	0
ENSCAFG00845026128	3924	3879	3536	3654
ENSCAFG00845028787	645	630	582	565
ENSCAFG00845002156	1817	1911	1632	1738
ENSCAFG00845026127	3	0	0	0
ENSCAFG00845028788	1212	1251	1268	1313
ENSCAFG00845002155	0	1	0	4
ENSCAFG00845026122	0	0	0	0
ENSCAFG00845028785	0	0	0	0
ENSCAFG00845002154	2403	2305	2309	2058
ENSCAFG00845026121	0	0	0	0
ENSCAFG00845028786	273	234	272	272
ENSCAFG00845002153	0	1	0	0
ENSCAFG00845026124	0	1	0	0
ENSCAFG00845028783	5	1	1	3
ENSCAFG00845002152	0	0	0	0
ENSCAFG00845026123	0	0	0	0
ENSCAFG00845028784	0	0	0	0
ENSCAFG00845002151	0	2	0	4
ENSCAFG00845028781	0	0	0	0
ENSCAFG00845002150	382	372	349	347
ENSCAFG00845028782	0	0	0	0
ENSCAFG00845026120	2	5	0	3
ENSCAFG00845028780	0	0	0	0
ENSCAFG00845014148	0	0	0	0
ENSCAFG00845014149	1493	1452	1409	1417
ENSCAFG00845014146	4	7	9	8
ENSCAFG00845014147	87	87	109	111
ENSCAFG00845014133	4275	3973	3607	3784
ENSCAFG00845016796	715	708	724	760
ENSCAFG00845014134	792	790	636	721
ENSCAFG00845016795	455	437	423	498
ENSCAFG00845014131	0	0	0	0
ENSCAFG00845016794	0	0	0	0
ENSCAFG00845014132	397	363	306	334

ENSCAFG00845016793	445	429	473	534
ENSCAFG00845016792	525	496	545	463
ENSCAFG00845014130	0	0	1	2
ENSCAFG00845016791	15639	15341	15786	15781
ENSCAFG00845016790	0	0	0	0
ENSCAFG00845026119	11	5	3	14
ENSCAFG00845026118	419	373	414	366
ENSCAFG00845026115	0	0	0	0
ENSCAFG00845028778	0	0	0	0
ENSCAFG00845002169	0	0	0	0
ENSCAFG00845026114	1	0	0	0
ENSCAFG00845028779	13	21	9	8
ENSCAFG00845002168	1757	1723	1885	1894
ENSCAFG00845026117	0	0	0	0
ENSCAFG00845028776	0	0	0	0
ENSCAFG00845002167	8	3	8	13
ENSCAFG00845026116	557	526	475	498
ENSCAFG00845028777	27	25	34	57
ENSCAFG00845002166	88	87	70	96
ENSCAFG00845026111	1178	1155	1188	1144
ENSCAFG00845028774	0	0	0	0
ENSCAFG00845002165	2464	2227	2839	2981
ENSCAFG00845026110	154	192	173	159
ENSCAFG00845028775	0	0	0	0
ENSCAFG00845002164	997	1036	1072	1010
ENSCAFG00845026113	0	0	0	0
ENSCAFG00845028772	0	0	0	0
ENSCAFG00845002163	0	0	0	0
ENSCAFG00845026112	0	0	0	0
ENSCAFG00845028773	221	190	302	252
ENSCAFG00845002162	1063	1055	1041	1101
ENSCAFG00845028770	0	0	0	0
ENSCAFG00845002161	857	741	671	619
ENSCAFG00845028771	470	448	474	418
ENSCAFG00845002160	0	0	0	0
ENSCAFG00845014139	0	0	0	0
ENSCAFG00845014137	547	548	653	649
ENSCAFG00845014138	0	0	0	0
ENSCAFG00845016799	0	0	0	0
ENSCAFG00845014135	3	4	3	3
ENSCAFG00845016798	603	618	626	684

ENSCAFG00845014136	15	14	7	4
ENSCAFG00845016797	0	0	0	0
ENSCAFG00845026199	579	531	438	489
ENSCAFG00845026198	81	95	97	98
ENSCAFG00845026195	708	719	655	670
ENSCAFG00845026194	93	70	92	88
ENSCAFG00845026197	1	1	0	1
ENSCAFG00845026196	78	51	81	76
ENSCAFG00845026190	0	0	0	0
ENSCAFG00845026193	530	488	518	443
ENSCAFG00845026192	0	0	0	0
ENSCAFG00845004720	912	879	1053	1067
ENSCAFG00845004721	1	0	3	0
ENSCAFG00845004722	210	207	196	225
ENSCAFG00845004723	3	5	5	1
ENSCAFG00845016709	7	4	0	5
ENSCAFG00845004724	0	0	1	0
ENSCAFG00845016708	639	546	652	661
ENSCAFG00845004725	151	181	170	165
ENSCAFG00845016707	0	0	0	0
ENSCAFG00845004726	0	0	0	0
ENSCAFG00845016706	251	277	308	340
ENSCAFG00845004727	0	0	0	0
ENSCAFG00845016705	788	701	713	793
ENSCAFG00845004728	174	165	178	147
ENSCAFG00845016704	0	0	0	0
ENSCAFG00845004729	541	502	474	554
ENSCAFG00845016703	621	687	642	681
ENSCAFG00845016702	413	436	455	403
ENSCAFG00845016701	3031	2999	2879	2787
ENSCAFG00845016700	0	0	0	0
ENSCAFG00845004710	0	0	0	1
ENSCAFG00845004711	0	0	0	0
ENSCAFG00845004712	350	329	343	333
ENSCAFG00845004713	0	0	0	0
ENSCAFG00845004714	1188	1100	1197	1133
ENSCAFG00845004715	26	31	41	28
ENSCAFG00845004716	28	51	59	40
ENSCAFG00845004717	8	7	7	4
ENSCAFG00845004718	0	0	0	0
ENSCAFG00845004719	0	0	0	0

ENSCAFG00845004700	1097	1109	1065	1047
ENSCAFG00845004701	0	0	0	0
ENSCAFG00845004702	0	0	0	0
ENSCAFG00845004703	0	0	0	0
ENSCAFG00845004704	0	0	0	0
ENSCAFG00845004705	2720	2670	2774	2950
ENSCAFG00845004706	0	1	0	0
ENSCAFG00845004707	1	2	0	0
ENSCAFG00845004708	0	0	1	2
ENSCAFG00845004709	601	568	574	569
ENSCAFG00845014122	6062	5761	5575	5486
ENSCAFG00845016785	0	0	0	0
ENSCAFG00845014123	129	131	138	107
ENSCAFG00845016784	0	0	2	0
ENSCAFG00845014120	450	325	401	429
ENSCAFG00845016783	4	3	2	1
ENSCAFG00845014121	2816	2873	2932	3089
ENSCAFG00845016782	0	0	0	0
ENSCAFG00845016781	2	3	0	0
ENSCAFG00845016780	3554	3553	3170	3142
ENSCAFG00845026108	14	2	8	4
ENSCAFG00845026107	12	10	23	18
ENSCAFG00845002139	3	1	1	0
ENSCAFG00845028769	137	154	132	143
ENSCAFG00845002138	24	17	8	9
ENSCAFG00845026109	0	0	0	0
ENSCAFG00845002137	44	69	66	43
ENSCAFG00845028767	0	0	0	0
ENSCAFG00845002136	15	23	9	12
ENSCAFG00845026103	539	553	574	499
ENSCAFG00845028768	0	0	0	0
ENSCAFG00845002135	1	0	2	2
ENSCAFG00845026106	774	750	748	739
ENSCAFG00845028765	653	695	659	729
ENSCAFG00845002134	0	0	0	0
ENSCAFG00845026105	33	31	29	17
ENSCAFG00845028766	2	1	2	0
ENSCAFG00845002133	206	208	141	141
ENSCAFG00845026100	522	542	446	501
ENSCAFG00845028763	4	1	3	4
ENSCAFG00845002132	0	0	0	0

ENSCAFG00845028764	156	132	147	177
ENSCAFG00845002131	1	0	0	0
ENSCAFG00845028761	1	0	0	0
ENSCAFG00845002130	0	0	0	0
ENSCAFG00845026101	4	9	10	5
ENSCAFG00845028762	1049	963	923	956
ENSCAFG00845028760	17	6	7	10
ENSCAFG00845014128	1	4	3	2
ENSCAFG00845014129	750	771	821	806
ENSCAFG00845014126	0	0	0	0
ENSCAFG00845016789	712	619	718	741
ENSCAFG00845014127	0	0	0	0
ENSCAFG00845016788	0	0	0	0
ENSCAFG00845014124	0	0	1	0
ENSCAFG00845016787	49	48	36	50
ENSCAFG00845014125	981	883	693	735
ENSCAFG00845016786	0	0	0	1
ENSCAFG00845014111	2	1	3	3
ENSCAFG00845016774	0	0	0	0
ENSCAFG00845014112	7	2	6	2
ENSCAFG00845016773	260	251	254	237
ENSCAFG00845016772	1	1	3	0
ENSCAFG00845014110	392	466	381	376
ENSCAFG00845016771	3	0	0	2
ENSCAFG00845004790	0	0	0	0
ENSCAFG00845028758	0	0	0	0
ENSCAFG00845002149	1	1	4	3
ENSCAFG00845004791	0	0	0	0
ENSCAFG00845028759	1348	1321	1242	1311
ENSCAFG00845002148	0	0	0	0
ENSCAFG00845004792	1	0	0	1
ENSCAFG00845028756	0	0	0	0
ENSCAFG00845002147	0	1	0	0
ENSCAFG00845004793	82	76	49	41
ENSCAFG00845028757	421	393	395	385
ENSCAFG00845002146	17	9	17	24
ENSCAFG00845004794	4488	4115	4682	4665
ENSCAFG00845028754	0	0	0	0
ENSCAFG00845002145	185	233	214	230
ENSCAFG00845004795	0	2	1	0
ENSCAFG00845028755	32	27	34	41

ENSCAFG00845002144	200	205	224	207
ENSCAFG00845004796	0	0	0	0
ENSCAFG00845028752	544	500	526	540
ENSCAFG00845002143	0	0	0	0
ENSCAFG00845004797	0	0	0	0
ENSCAFG00845028753	1364	1250	1446	1452
ENSCAFG00845002142	5	3	3	9
ENSCAFG00845004798	27	29	23	16
ENSCAFG00845028750	0	0	0	1
ENSCAFG00845002141	3317	2967	3131	3141
ENSCAFG00845004799	79	75	67	72
ENSCAFG00845028751	650	705	569	647
ENSCAFG00845002140	20	20	13	12
ENSCAFG00845014119	1634	1507	1461	1536
ENSCAFG00845014117	3072	2920	3159	3214
ENSCAFG00845014118	17	18	23	19
ENSCAFG00845016779	3	3	1	3
ENSCAFG00845014115	0	0	0	0
ENSCAFG00845016778	1	3	0	0
ENSCAFG00845014116	1784	1688	1783	1809
ENSCAFG00845016777	3421	3274	3139	3130
ENSCAFG00845014113	77	75	51	80
ENSCAFG00845016776	0	0	2	0
ENSCAFG00845014114	84	96	107	94
ENSCAFG00845016775	0	0	0	0
ENSCAFG00845014100	0	0	0	0
ENSCAFG00845016763	514	609	588	591
ENSCAFG00845014101	3	1	0	0
ENSCAFG00845016762	0	0	0	0
ENSCAFG00845016761	15	8	0	7
ENSCAFG00845016760	1	3	1	4
ENSCAFG00845002119	0	0	2	0
ENSCAFG00845028749	4	4	0	5
ENSCAFG00845002118	0	0	0	0
ENSCAFG00845002117	10	4	16	13
ENSCAFG00845028747	0	0	0	0
ENSCAFG00845002116	151	175	185	166
ENSCAFG00845004780	0	0	0	0
ENSCAFG00845028748	3023	2719	2971	2889
ENSCAFG00845002115	0	0	0	0
ENSCAFG00845004781	0	0	0	0

ENSCAFG00845028745	745	691	705	641
ENSCAFG00845002114	413	397	362	399
ENSCAFG00845004782	0	0	0	0
ENSCAFG00845028746	0	0	0	0
ENSCAFG00845002113	0	0	0	1
ENSCAFG00845004783	0	0	0	0
ENSCAFG00845028743	1861	1763	1970	1897
ENSCAFG00845002112	8	2	6	1
ENSCAFG00845004784	0	0	0	0
ENSCAFG00845028744	0	0	0	0
ENSCAFG00845002111	0	0	0	0
ENSCAFG00845004785	2	3	3	2
ENSCAFG00845028741	52	64	48	65
ENSCAFG00845002110	0	1	0	1
ENSCAFG00845004786	0	0	0	0
ENSCAFG00845028742	311	295	330	350
ENSCAFG00845004787	0	1	0	0
ENSCAFG00845004788	0	0	1	0
ENSCAFG00845028740	138	155	124	127
ENSCAFG00845004789	860	844	644	663
ENSCAFG00845014108	0	0	0	0
ENSCAFG00845014109	0	1	0	0
ENSCAFG00845014106	1700	1438	1523	1612
ENSCAFG00845016769	2	3	2	0
ENSCAFG00845014107	0	0	0	0
ENSCAFG00845016768	0	0	0	0
ENSCAFG00845014104	12	12	1	2
ENSCAFG00845016767	531	519	528	499
ENSCAFG00845014105	343	410	365	334
ENSCAFG00845016766	1	1	3	5
ENSCAFG00845014102	0	0	0	0
ENSCAFG00845016765	0	0	0	0
ENSCAFG00845014103	3	2	2	0
ENSCAFG00845016764	176	191	211	238
ENSCAFG00845016752	3	1	4	1
ENSCAFG00845016751	1622	1729	1469	1596
ENSCAFG00845016750	0	0	0	0
ENSCAFG00845028738	0	0	0	0
ENSCAFG00845002129	0	0	0	0
ENSCAFG00845028739	678	696	645	659
ENSCAFG00845002128	0	0	0	0

ENSCAFG00845028736	221	221	140	119
ENSCAFG00845002127	0	0	0	0
ENSCAFG00845028737	64	55	66	73
ENSCAFG00845002126	4	3	10	10
ENSCAFG00845004770	85	112	94	116
ENSCAFG00845028734	728	670	773	765
ENSCAFG00845002125	0	0	0	0
ENSCAFG00845004771	2	0	0	0
ENSCAFG00845028735	1097	1031	1188	1207
ENSCAFG00845002124	0	0	0	0
ENSCAFG00845004772	0	0	0	0
ENSCAFG00845028732	0	0	0	0
ENSCAFG00845002123	173	168	158	227
ENSCAFG00845004773	0	0	0	0
ENSCAFG00845028733	73	92	108	95
ENSCAFG00845002122	4	9	7	2
ENSCAFG00845004774	608	607	604	695
ENSCAFG00845028730	994	1009	879	873
ENSCAFG00845002121	3599	3499	3157	3361
ENSCAFG00845004775	72	69	89	62
ENSCAFG00845028731	18	25	17	16
ENSCAFG00845002120	33	18	17	27
ENSCAFG00845004776	0	0	0	0
ENSCAFG00845004777	0	0	0	0
ENSCAFG00845004778	404	391	364	363
ENSCAFG00845004779	135	120	156	114
ENSCAFG00845016759	1	0	2	6
ENSCAFG00845016758	0	0	0	0
ENSCAFG00845016757	1153	1102	1057	1084
ENSCAFG00845016756	1	0	1	2
ENSCAFG00845016755	0	0	0	0
ENSCAFG00845016754	0	0	0	0
ENSCAFG00845016753	0	0	0	0
ENSCAFG00845016741	0	0	0	0
ENSCAFG00845016740	2	1	5	8
ENSCAFG00845028729	10	9	4	3
ENSCAFG00845028727	0	0	0	0
ENSCAFG00845028728	0	0	0	0
ENSCAFG00845028725	0	0	0	0
ENSCAFG00845028726	0	0	0	0
ENSCAFG00845028723	633	606	566	653

ENSCAFG00845004760	125	124	126	109
ENSCAFG00845028724	0	0	0	0
ENSCAFG00845004761	0	0	0	3
ENSCAFG00845028721	134	130	132	132
ENSCAFG00845004762	1	1	1	3
ENSCAFG00845028722	392	408	467	510
ENSCAFG00845004763	5	1	2	5
ENSCAFG00845004764	2	2	10	4
ENSCAFG00845028720	1	0	0	0
ENSCAFG00845004765	1729	1665	1472	1530
ENSCAFG00845004766	10	15	22	16
ENSCAFG00845004767	1262	1232	1187	1124
ENSCAFG00845004768	220	270	258	229
ENSCAFG00845004769	12	8	9	12
ENSCAFG00845016749	258	230	209	205
ENSCAFG00845016748	0	0	0	0
ENSCAFG00845016747	46	46	33	39
ENSCAFG00845016746	5	4	1	4
ENSCAFG00845016745	75	72	71	63
ENSCAFG00845016744	0	0	0	0
ENSCAFG00845016743	0	0	0	0
ENSCAFG00845016742	49	57	56	47
ENSCAFG00845016730	0	0	0	0
ENSCAFG00845028718	267	270	280	301
ENSCAFG00845002109	21	30	25	38
ENSCAFG00845028719	308	292	401	409
ENSCAFG00845002108	4	4	8	2
ENSCAFG00845028716	0	1	1	0
ENSCAFG00845002107	54	49	47	39
ENSCAFG00845028717	0	0	0	0
ENSCAFG00845002106	0	0	0	1
ENSCAFG00845028714	12	11	13	12
ENSCAFG00845002105	0	0	0	0
ENSCAFG00845028715	2	0	0	0
ENSCAFG00845002104	37	30	25	28
ENSCAFG00845028712	37	42	33	29
ENSCAFG00845002103	223	200	194	186
ENSCAFG00845028713	0	0	0	0
ENSCAFG00845002102	0	3	1	0
ENSCAFG00845004750	0	1	0	0
ENSCAFG00845028710	0	0	0	0

ENSCAFG00845002101	75	65	113	121
ENSCAFG00845004751	74	83	53	89
ENSCAFG00845028711	0	0	0	0
ENSCAFG00845002100	5	5	4	2
ENSCAFG00845004752	712	657	655	702
ENSCAFG00845004753	3	7	4	6
ENSCAFG00845004754	1	0	0	2
ENSCAFG00845004755	0	0	0	0
ENSCAFG00845004756	1126	1078	1298	1162
ENSCAFG00845004757	181	123	146	168
ENSCAFG00845004758	6	3	4	3
ENSCAFG00845004759	766	816	737	695
ENSCAFG00845016739	1	0	0	0
ENSCAFG00845016738	149	148	116	108
ENSCAFG00845016737	0	0	0	0
ENSCAFG00845016736	34	30	25	21
ENSCAFG00845016735	613	429	516	548
ENSCAFG00845016734	231	266	193	203
ENSCAFG00845016733	0	0	1	0
ENSCAFG00845016732	3	3	1	0
ENSCAFG00845016731	0	0	0	0
ENSCAFG00845028709	337	284	376	366
ENSCAFG00845028707	904	858	804	839
ENSCAFG00845028708	163	187	132	178
ENSCAFG00845028705	0	0	0	0
ENSCAFG00845028706	16	16	18	16
ENSCAFG00845028703	333	254	251	248
ENSCAFG00845028704	0	0	0	0
ENSCAFG00845028701	0	1	0	0
ENSCAFG00845028702	0	0	0	0
ENSCAFG00845004740	232	263	255	251
ENSCAFG00845028700	0	0	0	0
ENSCAFG00845004741	0	0	0	0
ENSCAFG00845004742	342	349	318	368
ENSCAFG00845004743	152	167	159	143
ENSCAFG00845004744	1192	1063	982	1052
ENSCAFG00845004745	0	0	0	0
ENSCAFG00845004746	203	175	184	199
ENSCAFG00845004747	0	0	0	0
ENSCAFG00845016729	8675	8494	8121	7949
ENSCAFG00845004748	246	245	228	239

ENSCAFG00845016728	0	0	0	0
ENSCAFG00845004749	373	334	360	345
ENSCAFG00845016727	46	42	38	21
ENSCAFG00845016726	55	47	28	18
ENSCAFG00845016725	0	1	0	0
ENSCAFG00845016724	0	1	0	1
ENSCAFG00845016723	414	353	439	486
ENSCAFG00845016722	4	2	0	1
ENSCAFG00845016721	106	130	98	89
ENSCAFG00845016720	370	449	359	423
ENSCAFG00845004730	438	407	365	310
ENSCAFG00845004731	94	93	83	72
ENSCAFG00845004732	0	0	0	0
ENSCAFG00845004733	0	0	0	0
ENSCAFG00845004734	0	0	0	0
ENSCAFG00845004735	1938	1900	2007	2075
ENSCAFG00845004736	0	0	0	0
ENSCAFG00845016718	204	205	185	187
ENSCAFG00845004737	1127	1172	1129	1110
ENSCAFG00845016717	1	0	0	2
ENSCAFG00845004738	0	0	0	0
ENSCAFG00845016716	1	3	5	3
ENSCAFG00845004739	0	0	4	0
ENSCAFG00845016715	5	5	6	5
ENSCAFG00845016714	0	0	0	0
ENSCAFG00845016713	128	123	121	119
ENSCAFG00845016712	2673	2677	2334	2383
ENSCAFG00845016711	659	576	651	663
ENSCAFG00845016710	64	63	35	51
ENSCAFG00845016025	0	0	0	0
ENSCAFG00845018688	0	0	0	0
ENSCAFG00845016026	0	0	0	0
ENSCAFG00845018687	113	111	108	91
ENSCAFG00845016023	682	582	673	633
ENSCAFG00845018686	0	0	0	0
ENSCAFG00845016024	2	0	1	0
ENSCAFG00845018685	1	0	0	5
ENSCAFG00845016021	0	0	0	0
ENSCAFG00845018684	67	111	62	90
ENSCAFG00845016022	50	23	61	30
ENSCAFG00845018683	633	596	571	557

ENSCAFG00845018682	891	831	834	823
ENSCAFG00845016020	0	0	1	1
ENSCAFG00845018681	0	0	0	0
ENSCAFG00845018680	7	7	9	11
ENSCAFG00845028007	509	554	468	484
ENSCAFG00845004039	0	0	0	0
ENSCAFG00845028006	0	0	0	0
ENSCAFG00845004038	0	0	0	0
ENSCAFG00845028009	1704	1590	1723	1713
ENSCAFG00845004037	0	0	0	0
ENSCAFG00845028008	1039	965	925	898
ENSCAFG00845004036	0	4	1	4
ENSCAFG00845028003	0	0	0	0
ENSCAFG00845004035	0	0	0	0
ENSCAFG00845028002	0	0	0	0
ENSCAFG00845004034	3	1	3	0
ENSCAFG00845028005	356	317	284	279
ENSCAFG00845004033	131	134	199	197
ENSCAFG00845028004	0	0	0	0
ENSCAFG00845004032	0	3	4	5
ENSCAFG00845004031	3	1	2	3
ENSCAFG00845004030	0	0	0	0
ENSCAFG00845028001	135	137	114	134
ENSCAFG00845028000	4	7	2	5
ENSCAFG00845016029	0	0	0	0
ENSCAFG00845016027	0	0	0	0
ENSCAFG00845016028	0	0	0	0
ENSCAFG00845018689	4	2	3	3
ENSCAFG00845016014	274	302	199	197
ENSCAFG00845018677	652	652	610	630
ENSCAFG00845016015	1	0	0	2
ENSCAFG00845018676	0	0	0	0
ENSCAFG00845016012	0	0	0	0
ENSCAFG00845018675	2954	2882	2618	2433
ENSCAFG00845016013	48	71	93	86
ENSCAFG00845018674	331	305	289	283
ENSCAFG00845016010	32	25	28	41
ENSCAFG00845018673	2010	1994	1988	2049
ENSCAFG00845016011	0	0	0	0
ENSCAFG00845018672	6544	6395	6770	7002
ENSCAFG00845018671	491	479	440	412

ENSCAFG00845006690	0	0	0	0
ENSCAFG00845018670	0	0	1	0
ENSCAFG00845006691	0	0	2	2
ENSCAFG00845006692	3	1	0	0
ENSCAFG00845006693	4967	4846	4881	5121
ENSCAFG00845006694	0	0	0	0
ENSCAFG00845006695	2841	2642	2854	2830
ENSCAFG00845006696	7	3	6	5
ENSCAFG00845004049	550	473	478	506
ENSCAFG00845006697	27	22	38	29
ENSCAFG00845004048	0	0	0	0
ENSCAFG00845006698	2	2	0	0
ENSCAFG00845004047	0	0	0	0
ENSCAFG00845006699	0	0	0	0
ENSCAFG00845004046	135	127	205	197
ENSCAFG00845004045	0	0	0	0
ENSCAFG00845004044	0	0	0	0
ENSCAFG00845004043	0	0	0	0
ENSCAFG00845004042	0	1	0	0
ENSCAFG00845004041	0	0	0	0
ENSCAFG00845004040	1298	1221	1079	1169
ENSCAFG00845016018	773	691	679	664
ENSCAFG00845016019	7	4	3	7
ENSCAFG00845016016	2	2	5	6
ENSCAFG00845018679	0	0	0	0
ENSCAFG00845016017	0	0	0	0
ENSCAFG00845018678	0	2	1	1
ENSCAFG00845016003	0	0	0	0
ENSCAFG00845018666	2182	2228	2169	2323
ENSCAFG00845016004	54	49	69	95
ENSCAFG00845018665	0	1	0	0
ENSCAFG00845016001	0	0	0	0
ENSCAFG00845018664	348	330	356	362
ENSCAFG00845016002	449	406	414	366
ENSCAFG00845018663	2095	1892	1887	2004
ENSCAFG00845018662	1179	1236	1066	1091
ENSCAFG00845016000	6	2	9	1
ENSCAFG00845018660	0	1	0	0
ENSCAFG00845006680	0	0	0	0
ENSCAFG00845006681	0	0	0	0
ENSCAFG00845006682	0	1	0	0

ENSCAFG00845004019	0	0	0	0
ENSCAFG00845006683	0	0	0	0
ENSCAFG00845004018	1171	1204	845	928
ENSCAFG00845006684	0	0	0	1
ENSCAFG00845004017	0	0	0	0
ENSCAFG00845006685	2	1	0	0
ENSCAFG00845004016	0	0	1	0
ENSCAFG00845006686	0	0	0	0
ENSCAFG00845004015	0	0	0	0
ENSCAFG00845006687	76	62	83	95
ENSCAFG00845004014	316	312	308	285
ENSCAFG00845006688	700	768	837	696
ENSCAFG00845004013	1083	998	994	1042
ENSCAFG00845006689	0	0	0	0
ENSCAFG00845004012	0	0	0	1
ENSCAFG00845004011	478	485	416	451
ENSCAFG00845004010	0	0	0	0
ENSCAFG00845016009	0	0	0	0
ENSCAFG00845016007	2	3	1	3
ENSCAFG00845016008	1	0	0	3
ENSCAFG00845018669	645	649	653	638
ENSCAFG00845016005	7623	7121	7594	7556
ENSCAFG00845018668	0	0	0	0
ENSCAFG00845016006	0	0	1	0
ENSCAFG00845018667	142	146	152	160
ENSCAFG00845018655	0	0	0	0
ENSCAFG00845018654	0	0	0	1
ENSCAFG00845018653	157	137	142	161
ENSCAFG00845018652	2	3	2	9
ENSCAFG00845018651	21	21	12	16
ENSCAFG00845018650	18	14	13	18
ENSCAFG00845006670	1	0	0	1
ENSCAFG00845006671	11	12	12	9
ENSCAFG00845006672	0	0	0	0
ENSCAFG00845004029	1	1	0	0
ENSCAFG00845006673	0	0	0	0
ENSCAFG00845004028	0	0	0	0
ENSCAFG00845006674	0	1	0	0
ENSCAFG00845004027	1292	1345	1270	1154
ENSCAFG00845006675	76	93	63	81
ENSCAFG00845004026	0	0	0	0

ENSCAFG00845006676	0	0	0	0
ENSCAFG00845004025	0	0	0	0
ENSCAFG00845006677	7	15	7	6
ENSCAFG00845004024	0	0	0	0
ENSCAFG00845006678	2	6	6	4
ENSCAFG00845004023	550	570	506	541
ENSCAFG00845006679	0	0	0	0
ENSCAFG00845004022	0	0	0	0
ENSCAFG00845004021	0	0	0	0
ENSCAFG00845004020	0	0	0	0
ENSCAFG00845018659	0	0	0	0
ENSCAFG00845018658	0	0	1	0
ENSCAFG00845018657	1007	956	916	873
ENSCAFG00845018656	1071	924	1010	971
ENSCAFG00845018644	102	117	130	158
ENSCAFG00845018643	0	0	0	0
ENSCAFG00845018642	0	0	0	0
ENSCAFG00845018641	1905	1755	1730	1718
ENSCAFG00845018640	0	0	0	0
ENSCAFG00845006660	1	0	1	1
ENSCAFG00845006661	1	1	0	0
ENSCAFG00845006662	0	0	0	0
ENSCAFG00845006663	0	0	0	0
ENSCAFG00845006664	0	0	0	0
ENSCAFG00845006665	4	9	4	4
ENSCAFG00845006666	37	32	29	42
ENSCAFG00845006667	1345	1404	1202	1293
ENSCAFG00845006668	4	0	0	0
ENSCAFG00845006669	0	0	0	0
ENSCAFG00845018649	0	0	0	0
ENSCAFG00845018648	0	0	0	0
ENSCAFG00845018647	737	692	601	618
ENSCAFG00845018646	699	781	1164	1130
ENSCAFG00845018645	0	0	0	0
ENSCAFG00845018633	1	0	0	0
ENSCAFG00845018632	320	316	328	402
ENSCAFG00845018631	0	0	0	0
ENSCAFG00845018630	547	544	566	559
ENSCAFG00845004009	3392	3413	3201	3425
ENSCAFG00845004008	1796	1631	1728	1703
ENSCAFG00845006650	10	28	18	25

ENSCAFG00845004007	472	432	510	587
ENSCAFG00845006651	1448	1433	1299	1424
ENSCAFG00845004006	0	0	0	0
ENSCAFG00845006652	730	753	656	681
ENSCAFG00845004005	466	424	520	519
ENSCAFG00845006653	0	0	4	1
ENSCAFG00845004004	0	6	2	5
ENSCAFG00845006654	0	0	0	0
ENSCAFG00845004003	1095	977	1025	1089
ENSCAFG00845006655	0	0	0	0
ENSCAFG00845004002	3	4	3	1
ENSCAFG00845006656	264	257	245	242
ENSCAFG00845004001	1576	1594	1806	1817
ENSCAFG00845006657	449	460	500	489
ENSCAFG00845004000	0	0	0	1
ENSCAFG00845006658	21	34	41	29
ENSCAFG00845006659	1	0	1	0
ENSCAFG00845018639	0	0	2	0
ENSCAFG00845018638	1339	1270	1356	1202
ENSCAFG00845018637	0	0	0	0
ENSCAFG00845018636	0	1	0	0
ENSCAFG00845018635	590	531	636	610
ENSCAFG00845018634	8	12	15	11
ENSCAFG00845018622	280	280	289	235
ENSCAFG00845018621	512	529	498	539
ENSCAFG00845018620	1	0	0	1
ENSCAFG00845006640	2	1	0	0
ENSCAFG00845006641	650	634	573	572
ENSCAFG00845006642	0	0	0	0
ENSCAFG00845006643	888	902	865	853
ENSCAFG00845006644	400	391	394	371
ENSCAFG00845006645	609	630	584	575
ENSCAFG00845006646	123	129	136	142
ENSCAFG00845006647	0	0	0	0
ENSCAFG00845006648	0	0	0	1
ENSCAFG00845006649	346	336	358	348
ENSCAFG00845018629	0	0	0	0
ENSCAFG00845018628	2	0	0	0
ENSCAFG00845018627	1	6	5	6
ENSCAFG00845018626	0	0	0	0
ENSCAFG00845018625	0	2	0	1

ENSCAFG00845018623	59	55	50	43
ENSCAFG00845018611	0	0	0	0
ENSCAFG00845018610	5	0	4	0
ENSCAFG00845006630	0	0	0	0
ENSCAFG00845006631	1	0	0	2
ENSCAFG00845006632	1	2	0	0
ENSCAFG00845006633	0	0	0	0
ENSCAFG00845006634	0	0	0	0
ENSCAFG00845006635	1192	1034	1173	1122
ENSCAFG00845006636	0	0	0	0
ENSCAFG00845006637	1911	1831	2613	2522
ENSCAFG00845006638	817	896	798	874
ENSCAFG00845006639	2	0	3	4
ENSCAFG00845018619	1	0	1	0
ENSCAFG00845018618	1	0	0	0
ENSCAFG00845018617	0	0	1	1
ENSCAFG00845018616	0	0	0	0
ENSCAFG00845018615	0	0	0	0
ENSCAFG00845018614	0	0	0	0
ENSCAFG00845018613	43	39	35	30
ENSCAFG00845018612	0	0	0	0
ENSCAFG00845028087	6	10	8	9
ENSCAFG00845028086	6	2	5	6
ENSCAFG00845028089	0	0	0	0
ENSCAFG00845028088	0	2	0	0
ENSCAFG00845028083	0	0	1	0
ENSCAFG00845028082	1444	1366	1226	1368
ENSCAFG00845028085	0	0	0	0
ENSCAFG00845028084	0	0	0	0
ENSCAFG00845028081	2	1	4	2
ENSCAFG00845028080	0	0	0	0
ENSCAFG00845016098	1304	1390	1248	1313
ENSCAFG00845016099	0	0	0	0
ENSCAFG00845016096	14	7	16	7
ENSCAFG00845016097	120	134	86	122
ENSCAFG00845016094	1	0	0	0
ENSCAFG00845016095	1	0	2	0
ENSCAFG00845016092	74	75	65	65
ENSCAFG00845016093	2451	2364	2085	2023
ENSCAFG00845016090	0	0	0	0
ENSCAFG00845016091	0	0	0	0

ENSCAFG00845028079	583	574	592	567
ENSCAFG00845028076	0	0	0	0
ENSCAFG00845028075	0	0	0	0
ENSCAFG00845028078	8050	7874	8530	9028
ENSCAFG00845028077	0	0	0	0
ENSCAFG00845028072	129	156	186	156
ENSCAFG00845028071	0	0	0	0
ENSCAFG00845028074	0	0	0	0
ENSCAFG00845028073	29	38	31	44
ENSCAFG00845028070	0	0	0	0
ENSCAFG00845016089	3	0	1	0
ENSCAFG00845016087	0	0	0	0
ENSCAFG00845016088	270	239	286	331
ENSCAFG00845016085	0	0	0	0
ENSCAFG00845016086	94	100	106	82
ENSCAFG00845016083	0	0	0	0
ENSCAFG00845016084	1091	1110	1161	1109
ENSCAFG00845016081	3958	3893	4060	3999
ENSCAFG00845016082	0	0	0	0
ENSCAFG00845016080	0	0	0	0
ENSCAFG00845028069	1023	872	969	1056
ENSCAFG00845028068	519	600	545	616
ENSCAFG00845004099	358	344	407	408
ENSCAFG00845004098	0	0	0	0
ENSCAFG00845028065	1106	1011	1065	1057
ENSCAFG00845004097	1	0	2	0
ENSCAFG00845028064	0	0	0	0
ENSCAFG00845004096	0	0	3	2
ENSCAFG00845028067	703	705	603	604
ENSCAFG00845004095	0	0	0	0
ENSCAFG00845028066	2222	2122	1940	2046
ENSCAFG00845004094	3	7	9	5
ENSCAFG00845028061	1585	1623	1525	1713
ENSCAFG00845004093	0	0	0	0
ENSCAFG00845028060	1132	1186	990	1008
ENSCAFG00845004092	0	0	0	0
ENSCAFG00845028063	0	0	0	0
ENSCAFG00845004091	23	32	8	31
ENSCAFG00845028062	3	0	0	2
ENSCAFG00845004090	5	1	2	0
ENSCAFG00845016078	0	0	0	0

ENSCAFG00845016079	1	1	2	1
ENSCAFG00845016076	0	0	0	0
ENSCAFG00845016077	0	0	0	0
ENSCAFG00845016074	5	4	2	3
ENSCAFG00845016075	0	0	0	0
ENSCAFG00845016072	2374	2474	2788	2765
ENSCAFG00845016073	697	564	736	683
ENSCAFG00845016070	42	53	45	53
ENSCAFG00845016071	0	0	0	0
ENSCAFG00845028058	0	0	1	0
ENSCAFG00845028057	0	0	0	0
ENSCAFG00845028059	0	0	0	0
ENSCAFG00845028054	0	0	2	2
ENSCAFG00845028053	341	307	297	317
ENSCAFG00845028056	5	2	3	1
ENSCAFG00845028055	1299	1228	1252	1170
ENSCAFG00845028050	0	0	0	0
ENSCAFG00845028052	695	660	722	730
ENSCAFG00845028051	0	0	0	0
ENSCAFG00845016069	1713	1656	1528	1458
ENSCAFG00845016067	0	0	0	1
ENSCAFG00845016068	0	0	0	0
ENSCAFG00845016065	57	46	69	65
ENSCAFG00845016066	26	22	14	11
ENSCAFG00845016063	0	0	0	0
ENSCAFG00845016064	768	845	820	857
ENSCAFG00845016061	2389	2436	2516	2586
ENSCAFG00845016062	1	1	0	0
ENSCAFG00845016060	0	0	0	0
ENSCAFG00845028047	0	0	0	0
ENSCAFG00845004079	0	0	0	0
ENSCAFG00845028046	522	593	457	517
ENSCAFG00845004078	0	0	0	0
ENSCAFG00845028049	388	456	439	436
ENSCAFG00845004077	0	0	0	0
ENSCAFG00845028048	168	205	186	193
ENSCAFG00845004076	1819	1636	1689	1721
ENSCAFG00845028043	0	0	0	0
ENSCAFG00845004075	0	0	0	0
ENSCAFG00845028042	0	0	0	0
ENSCAFG00845004074	275	231	249	260

ENSCAFG00845028045	767	732	837	796
ENSCAFG00845004073	7	5	7	7
ENSCAFG00845028044	129	109	115	130
ENSCAFG00845004072	0	0	0	0
ENSCAFG00845004071	0	0	0	0
ENSCAFG00845004070	0	0	0	0
ENSCAFG00845028041	820	750	740	700
ENSCAFG00845028040	0	0	0	0
ENSCAFG00845016058	1223	1182	1036	1060
ENSCAFG00845016059	0	0	0	0
ENSCAFG00845016056	353	370	373	372
ENSCAFG00845016057	2	1	1	0
ENSCAFG00845016054	1306	1279	1335	1433
ENSCAFG00845016055	0	0	0	0
ENSCAFG00845016052	1252	1206	1437	1476
ENSCAFG00845016053	0	0	0	0
ENSCAFG00845016050	0	0	0	0
ENSCAFG00845016051	604	578	545	556
ENSCAFG00845028039	0	0	0	0
ENSCAFG00845028036	0	0	0	0
ENSCAFG00845028035	1999	1974	2084	2056
ENSCAFG00845004089	1393	1356	1269	1261
ENSCAFG00845028038	7	8	5	3
ENSCAFG00845004088	1970	1971	1856	1933
ENSCAFG00845028037	0	0	0	0
ENSCAFG00845004087	1093	1217	1107	1175
ENSCAFG00845028032	0	0	0	0
ENSCAFG00845004086	0	0	0	0
ENSCAFG00845028031	0	0	0	0
ENSCAFG00845004085	0	0	0	0
ENSCAFG00845028034	0	0	0	0
ENSCAFG00845004084	1	4	1	10
ENSCAFG00845028033	0	0	0	0
ENSCAFG00845004083	0	0	0	0
ENSCAFG00845004082	8	8	3	6
ENSCAFG00845004081	0	0	0	0
ENSCAFG00845028030	0	0	0	0
ENSCAFG00845004080	0	0	0	0
ENSCAFG00845016047	0	0	0	0
ENSCAFG00845016048	698	609	681	715
ENSCAFG00845016045	328	328	341	346

ENSCAFG00845016046	0	0	0	0
ENSCAFG00845016043	233	242	254	237
ENSCAFG00845016044	2	1	1	0
ENSCAFG00845016041	646	655	581	621
ENSCAFG00845016042	0	0	0	0
ENSCAFG00845016040	1626	1507	1423	1448
ENSCAFG00845028029	39	33	37	35
ENSCAFG00845028028	0	0	0	0
ENSCAFG00845004059	3	2	1	6
ENSCAFG00845004058	34	31	30	44
ENSCAFG00845028025	1	0	0	0
ENSCAFG00845004057	0	0	0	0
ENSCAFG00845028024	7	9	14	10
ENSCAFG00845004056	0	0	0	0
ENSCAFG00845028027	0	0	0	0
ENSCAFG00845004055	0	0	0	0
ENSCAFG00845028026	339	341	298	259
ENSCAFG00845004054	0	0	0	0
ENSCAFG00845028021	284	268	277	278
ENSCAFG00845004053	186	201	217	217
ENSCAFG00845028020	0	0	0	0
ENSCAFG00845004052	3942	3794	3646	3759
ENSCAFG00845028023	0	0	0	0
ENSCAFG00845004051	0	0	0	0
ENSCAFG00845028022	258	258	212	191
ENSCAFG00845004050	1889	1852	1775	1773
ENSCAFG00845016049	2	1	1	0
ENSCAFG00845016036	1644	1667	1753	1837
ENSCAFG00845018699	629	534	588	679
ENSCAFG00845016037	0	0	0	0
ENSCAFG00845018698	0	0	0	0
ENSCAFG00845016034	0	0	0	0
ENSCAFG00845018697	0	0	0	0
ENSCAFG00845016035	147	117	102	118
ENSCAFG00845018696	0	0	0	0
ENSCAFG00845016032	1533	1310	1463	1370
ENSCAFG00845018695	0	0	0	0
ENSCAFG00845016033	179	139	162	174
ENSCAFG00845018694	0	0	0	0
ENSCAFG00845016030	0	0	0	0
ENSCAFG00845018693	0	1	0	0

ENSCAFG00845016031	3849	3821	3434	3634
ENSCAFG00845018692	0	0	0	1
ENSCAFG00845018691	0	0	0	0
ENSCAFG00845018690	137	130	121	126
ENSCAFG00845028018	0	0	0	0
ENSCAFG00845028017	5	2	6	9
ENSCAFG00845028019	0	0	0	0
ENSCAFG00845004069	0	0	0	0
ENSCAFG00845028014	1	0	4	1
ENSCAFG00845004068	0	0	3	0
ENSCAFG00845028013	0	0	2	2
ENSCAFG00845004067	0	0	0	1
ENSCAFG00845028016	688	670	728	712
ENSCAFG00845004066	0	0	0	0
ENSCAFG00845028015	946	905	793	921
ENSCAFG00845004065	112	96	112	114
ENSCAFG00845028010	0	0	0	0
ENSCAFG00845004064	33	40	40	34
ENSCAFG00845004063	0	0	0	0
ENSCAFG00845028012	0	0	0	0
ENSCAFG00845004062	0	0	0	0
ENSCAFG00845028011	0	0	0	0
ENSCAFG00845004061	281	253	234	212
ENSCAFG00845004060	1895	1992	1805	1871
ENSCAFG00845016038	0	0	0	0
ENSCAFG00845016039	1347	1388	1302	1352
ENSCAFG00845018600	356	368	379	332
ENSCAFG00845006620	5	6	3	5
ENSCAFG00845006621	0	0	0	0
ENSCAFG00845006622	8469	7896	8751	9158
ENSCAFG00845006623	3	1	1	0
ENSCAFG00845006624	29	15	17	10
ENSCAFG00845006625	22	16	29	17
ENSCAFG00845006626	0	0	0	0
ENSCAFG00845006627	0	0	0	0
ENSCAFG00845006628	0	2	0	1
ENSCAFG00845006629	311	309	282	331
ENSCAFG00845018609	0	0	0	0
ENSCAFG00845018608	2361	2302	2813	2674
ENSCAFG00845018607	0	0	1	0
ENSCAFG00845018606	116	135	134	143

ENSCAFG00845018605	0	0	0	0
ENSCAFG00845018604	0	0	0	0
ENSCAFG00845018603	0	0	0	0
ENSCAFG00845018602	185	177	191	205
ENSCAFG00845018601	1	2	1	2
ENSCAFG00845006610	2157	2123	1886	1800
ENSCAFG00845006611	348	306	413	427
ENSCAFG00845006612	1990	1892	1745	1697
ENSCAFG00845006613	1387	1372	1348	1318
ENSCAFG00845006614	1	1	0	0
ENSCAFG00845006615	1	0	0	0
ENSCAFG00845006616	0	0	0	0
ENSCAFG00845006617	0	0	0	0
ENSCAFG00845006618	0	0	0	0
ENSCAFG00845006619	10	12	7	8
ENSCAFG00845006600	0	0	0	0
ENSCAFG00845006601	0	0	0	0
ENSCAFG00845006602	0	1	1	0
ENSCAFG00845006603	3	9	15	10
ENSCAFG00845006604	0	0	0	0
ENSCAFG00845006605	56	45	38	44
ENSCAFG00845006606	0	0	0	3
ENSCAFG00845006607	12	14	14	14
ENSCAFG00845006608	673	649	717	729
ENSCAFG00845006609	337	350	299	282
ENSCAFG00845016146	3792	3675	4055	4412
ENSCAFG00845016147	147	116	136	126
ENSCAFG00845016144	4	5	2	3
ENSCAFG00845016145	0	0	0	0
ENSCAFG00845016142	27	30	27	29
ENSCAFG00845016143	135	142	144	151
ENSCAFG00845016140	1	1	4	1
ENSCAFG00845016141	218	200	229	250
ENSCAFG00845028128	0	0	0	0
ENSCAFG00845028127	117	106	98	136
ENSCAFG00845004159	0	0	0	0
ENSCAFG00845004158	0	0	0	0
ENSCAFG00845028129	0	0	0	1
ENSCAFG00845004157	497	490	512	552
ENSCAFG00845028124	1	4	4	2
ENSCAFG00845004156	1969	1908	1717	1775

ENSCAFG00845028123	64	58	61	61
ENSCAFG00845004155	0	0	0	0
ENSCAFG00845028126	354	374	417	458
ENSCAFG00845004154	1510	1403	1264	1365
ENSCAFG00845028125	39	44	23	43
ENSCAFG00845004153	0	0	0	0
ENSCAFG00845028120	0	0	0	0
ENSCAFG00845004152	3953	3804	4386	4280
ENSCAFG00845004151	0	0	0	0
ENSCAFG00845028122	0	0	0	0
ENSCAFG00845004150	0	0	0	0
ENSCAFG00845028121	564	565	539	620
ENSCAFG00845016148	344	376	276	327
ENSCAFG00845016149	4	7	1	0
ENSCAFG00845016135	1119	1060	1021	1050
ENSCAFG00845018798	569	587	523	623
ENSCAFG00845016136	1	2	2	1
ENSCAFG00845018797	5459	5117	5303	5429
ENSCAFG00845016133	0	0	0	0
ENSCAFG00845018796	0	0	0	0
ENSCAFG00845016134	2338	2197	2429	2342
ENSCAFG00845018795	49	62	35	48
ENSCAFG00845016131	0	3	1	4
ENSCAFG00845018794	5	3	11	2
ENSCAFG00845016132	5086	4987	4303	4340
ENSCAFG00845018793	2	0	0	0
ENSCAFG00845018792	0	0	0	0
ENSCAFG00845016130	760	703	711	749
ENSCAFG00845018791	0	0	0	0
ENSCAFG00845018790	62	42	41	37
ENSCAFG00845028117	0	0	0	0
ENSCAFG00845028116	13	12	23	29
ENSCAFG00845028119	1	5	1	0
ENSCAFG00845004169	173	175	150	122
ENSCAFG00845028118	296	337	296	365
ENSCAFG00845004168	1115	1102	1030	1053
ENSCAFG00845028113	0	0	0	0
ENSCAFG00845004167	0	1	1	0
ENSCAFG00845028112	222	228	251	240
ENSCAFG00845004166	0	0	0	0
ENSCAFG00845028115	0	0	0	1

ENSCAFG00845004165	1	0	0	0
ENSCAFG00845028114	0	0	0	0
ENSCAFG00845004164	0	0	0	0
ENSCAFG00845004163	1404	1391	1463	1487
ENSCAFG00845004162	10	2	11	7
ENSCAFG00845028111	0	0	0	0
ENSCAFG00845004161	1697	1550	1477	1496
ENSCAFG00845028110	0	0	0	0
ENSCAFG00845004160	0	0	0	0
ENSCAFG00845016139	0	0	0	0
ENSCAFG00845016137	0	1	0	0
ENSCAFG00845016138	0	0	0	0
ENSCAFG00845016124	0	0	0	0
ENSCAFG00845018787	131	116	124	97
ENSCAFG00845016125	0	0	0	0
ENSCAFG00845018786	845	767	728	771
ENSCAFG00845016122	4	1	3	2
ENSCAFG00845018785	4	1	5	0
ENSCAFG00845016123	0	1	3	1
ENSCAFG00845018784	0	0	0	0
ENSCAFG00845016120	15	16	7	10
ENSCAFG00845018783	239	281	252	281
ENSCAFG00845016121	314	282	297	328
ENSCAFG00845018782	721	620	636	645
ENSCAFG00845018781	1275	1258	1182	1035
ENSCAFG00845018780	4	3	3	5
ENSCAFG00845028109	0	0	0	0
ENSCAFG00845004139	5	3	2	0
ENSCAFG00845028106	0	0	0	0
ENSCAFG00845004138	0	0	0	0
ENSCAFG00845028105	0	0	0	0
ENSCAFG00845004137	0	0	0	0
ENSCAFG00845028108	0	0	0	0
ENSCAFG00845004136	153	172	265	271
ENSCAFG00845028107	5	2	0	2
ENSCAFG00845004135	2838	2936	2758	2844
ENSCAFG00845028102	18	19	25	24
ENSCAFG00845004134	0	0	0	0
ENSCAFG00845028101	276	305	318	300
ENSCAFG00845004133	813	840	807	764
ENSCAFG00845028104	0	0	0	0

ENSCAFG00845004132	0	0	0	0
ENSCAFG00845028103	0	0	0	0
ENSCAFG00845004131	87	92	84	77
ENSCAFG00845004130	514	482	420	449
ENSCAFG00845028100	8	3	2	0
ENSCAFG00845016128	0	0	0	0
ENSCAFG00845016129	772	727	730	790
ENSCAFG00845016126	4	1	2	1
ENSCAFG00845018789	930	784	798	823
ENSCAFG00845016127	4	9	2	3
ENSCAFG00845018788	0	0	0	0
ENSCAFG00845016113	2	1	4	1
ENSCAFG00845018776	10	16	5	6
ENSCAFG00845016114	70	60	77	54
ENSCAFG00845018775	2	0	4	0
ENSCAFG00845016111	0	0	0	0
ENSCAFG00845018774	1252	1194	1236	1155
ENSCAFG00845016112	0	0	0	0
ENSCAFG00845018773	0	0	0	0
ENSCAFG00845018772	0	0	0	0
ENSCAFG00845016110	23	18	14	12
ENSCAFG00845018771	0	0	0	0
ENSCAFG00845018770	432	486	420	463
ENSCAFG00845006790	9	9	2	1
ENSCAFG00845006791	190	180	134	160
ENSCAFG00845006792	192	236	233	309
ENSCAFG00845006793	302	290	287	264
ENSCAFG00845006794	7	6	5	8
ENSCAFG00845004149	0	0	0	0
ENSCAFG00845006795	244	204	231	233
ENSCAFG00845004148	1	0	3	0
ENSCAFG00845006796	0	0	0	0
ENSCAFG00845004147	0	0	0	0
ENSCAFG00845006797	0	0	0	0
ENSCAFG00845004146	4241	4151	4146	4059
ENSCAFG00845006798	2581	2502	2332	2448
ENSCAFG00845004145	166	141	144	162
ENSCAFG00845006799	1	0	0	2
ENSCAFG00845004144	0	0	0	0
ENSCAFG00845004143	0	0	0	0
ENSCAFG00845004142	0	0	0	0

ENSCAFG00845004141	2	4	2	1
ENSCAFG00845004140	0	0	0	0
ENSCAFG00845016119	0	0	0	0
ENSCAFG00845016117	2	0	0	0
ENSCAFG00845016118	0	0	0	0
ENSCAFG00845018779	0	0	0	0
ENSCAFG00845016115	0	0	0	0
ENSCAFG00845018778	868	753	748	724
ENSCAFG00845016116	4268	4278	3924	4028
ENSCAFG00845018777	168	167	163	182
ENSCAFG00845016102	6861	6656	6795	6913
ENSCAFG00845018765	621	660	679	697
ENSCAFG00845016103	81	74	65	69
ENSCAFG00845018764	0	0	0	0
ENSCAFG00845016100	0	0	0	0
ENSCAFG00845018763	382	313	375	341
ENSCAFG00845016101	21	37	23	19
ENSCAFG00845018762	0	0	0	0
ENSCAFG00845018761	923	935	827	836
ENSCAFG00845006780	1283	1206	1159	1106
ENSCAFG00845004119	0	0	0	0
ENSCAFG00845006781	0	0	0	0
ENSCAFG00845004118	26	31	14	18
ENSCAFG00845006782	0	0	1	2
ENSCAFG00845004117	33	53	28	33
ENSCAFG00845006783	1349	1340	1591	1678
ENSCAFG00845004116	0	0	0	0
ENSCAFG00845006784	869	871	707	766
ENSCAFG00845004115	0	0	0	0
ENSCAFG00845006785	0	0	0	0
ENSCAFG00845004114	2048	2001	2021	2050
ENSCAFG00845006786	0	0	0	0
ENSCAFG00845004113	0	0	0	0
ENSCAFG00845006787	0	0	0	0
ENSCAFG00845004112	223	232	288	274
ENSCAFG00845006788	0	0	0	0
ENSCAFG00845004111	154	150	145	157
ENSCAFG00845006789	9	12	14	8
ENSCAFG00845004110	0	1	0	0
ENSCAFG00845016108	2	0	0	2
ENSCAFG00845016109	0	0	0	0

ENSCAFG00845016106	0	0	0	0
ENSCAFG00845018769	1	0	0	0
ENSCAFG00845016107	17	31	20	22
ENSCAFG00845018768	120	95	132	153
ENSCAFG00845016104	864	872	703	853
ENSCAFG00845018767	0	0	0	0
ENSCAFG00845016105	2597	2450	2813	2819
ENSCAFG00845018766	0	0	0	1
ENSCAFG00845018754	0	0	0	0
ENSCAFG00845018753	0	0	0	0
ENSCAFG00845018752	512	536	429	522
ENSCAFG00845018751	0	0	1	2
ENSCAFG00845018750	0	0	0	2
ENSCAFG00845006770	467	446	416	419
ENSCAFG00845004129	171	192	173	139
ENSCAFG00845006771	0	0	0	0
ENSCAFG00845004128	0	0	0	0
ENSCAFG00845006772	0	0	0	0
ENSCAFG00845004127	0	0	0	0
ENSCAFG00845006773	552	526	631	617
ENSCAFG00845004126	106	62	81	87
ENSCAFG00845006774	2	6	1	2
ENSCAFG00845004125	0	0	0	0
ENSCAFG00845006775	0	0	0	0
ENSCAFG00845004124	2577	2470	2541	2482
ENSCAFG00845006776	0	0	1	0
ENSCAFG00845004123	864	878	795	739
ENSCAFG00845006777	0	1	3	1
ENSCAFG00845004122	7	2	16	19
ENSCAFG00845006778	0	0	0	0
ENSCAFG00845004121	0	0	0	0
ENSCAFG00845006779	145	117	148	146
ENSCAFG00845004120	0	0	0	0
ENSCAFG00845018759	0	0	0	0
ENSCAFG00845018758	0	0	0	0
ENSCAFG00845018757	68	61	47	68
ENSCAFG00845018756	0	0	0	0
ENSCAFG00845018755	0	0	0	0
ENSCAFG00845018743	5343	5020	4755	4993
ENSCAFG00845018742	0	0	0	0
ENSCAFG00845018741	17	23	21	18

ENSCAFG00845018740	246	265	287	293
ENSCAFG00845006760	0	0	0	0
ENSCAFG00845006761	0	2	4	1
ENSCAFG00845006762	265	173	209	215
ENSCAFG00845006763	0	0	0	0
ENSCAFG00845006764	1848	1751	1882	1847
ENSCAFG00845006765	0	0	0	0
ENSCAFG00845006766	1	3	1	1
ENSCAFG00845006767	0	0	0	0
ENSCAFG00845006768	617	604	590	618
ENSCAFG00845006769	39	42	59	50
ENSCAFG00845018749	0	0	0	0
ENSCAFG00845018748	515	525	431	449
ENSCAFG00845018747	0	0	0	0
ENSCAFG00845018746	1521	1449	1312	1290
ENSCAFG00845018745	0	0	0	0
ENSCAFG00845018744	0	0	0	0
ENSCAFG00845018732	4	1	6	4
ENSCAFG00845018731	0	0	0	0
ENSCAFG00845018730	0	0	0	0
ENSCAFG00845004109	37	55	79	63
ENSCAFG00845004108	1	0	0	2
ENSCAFG00845004107	0	0	0	0
ENSCAFG00845004106	5	9	5	10
ENSCAFG00845006750	2288	2266	2223	2302
ENSCAFG00845004105	0	0	0	0
ENSCAFG00845006751	1886	1867	1817	1754
ENSCAFG00845004104	0	0	0	0
ENSCAFG00845006752	1219	1148	1194	1122
ENSCAFG00845004103	0	2	7	0
ENSCAFG00845006753	7901	7778	7816	8207
ENSCAFG00845004102	9	6	1	7
ENSCAFG00845006754	0	0	0	0
ENSCAFG00845004101	0	0	0	0
ENSCAFG00845006755	1	0	2	0
ENSCAFG00845004100	2491	2315	2437	2540
ENSCAFG00845006756	2673	2542	2509	2636
ENSCAFG00845006757	0	0	0	0
ENSCAFG00845006758	0	0	0	0
ENSCAFG00845006759	1429	1417	1597	1613
ENSCAFG00845018739	4	10	5	4

ENSCAFG00845018738	444	440	510	463
ENSCAFG00845018737	0	0	0	0
ENSCAFG00845018736	0	2	0	0
ENSCAFG00845018735	0	0	1	0
ENSCAFG00845018734	0	0	0	0
ENSCAFG00845018733	18	15	7	5
ENSCAFG00845028197	0	0	1	1
ENSCAFG00845028196	1	0	1	1
ENSCAFG00845028199	0	0	0	0
ENSCAFG00845028198	63	58	94	73
ENSCAFG00845028193	0	0	0	0
ENSCAFG00845028192	0	0	0	0
ENSCAFG00845028195	0	0	0	0
ENSCAFG00845028194	3	1	3	0
ENSCAFG00845028191	356	319	364	388
ENSCAFG00845028190	0	0	0	0
ENSCAFG00845028189	1024	952	1053	986
ENSCAFG00845028186	0	0	0	0
ENSCAFG00845028185	45	42	36	43
ENSCAFG00845028188	443	395	387	415
ENSCAFG00845028187	301	348	185	197
ENSCAFG00845028182	0	0	0	0
ENSCAFG00845028181	0	0	0	0
ENSCAFG00845028184	531	491	498	507
ENSCAFG00845028183	1	0	0	0
ENSCAFG00845028180	23	14	12	9
ENSCAFG00845016199	0	0	0	0
ENSCAFG00845016197	0	0	0	0
ENSCAFG00845016198	0	0	0	0
ENSCAFG00845016195	1068	1006	1063	1100
ENSCAFG00845016196	828	806	845	802
ENSCAFG00845016193	228	220	224	239
ENSCAFG00845016194	1	2	3	0
ENSCAFG00845016191	73	74	76	58
ENSCAFG00845016192	5	5	0	5
ENSCAFG00845016190	3	4	2	6
ENSCAFG00845028179	0	0	0	0
ENSCAFG00845028178	0	0	0	0
ENSCAFG00845028175	26	12	35	12
ENSCAFG00845028174	1	1	1	3
ENSCAFG00845028177	1930	1914	1847	1745

ENSCAFG00845028176	1116	1031	1129	1090
ENSCAFG00845028171	1	0	2	1
ENSCAFG00845028170	1	0	1	0
ENSCAFG00845028173	1837	1858	1874	1990
ENSCAFG00845028172	0	0	0	0
ENSCAFG00845016188	495	451	489	493
ENSCAFG00845016189	0	0	0	0
ENSCAFG00845016186	1	2	0	1
ENSCAFG00845016187	0	0	0	0
ENSCAFG00845016184	0	0	0	0
ENSCAFG00845016185	120	110	202	197
ENSCAFG00845016182	131	174	152	138
ENSCAFG00845016183	312	356	311	333
ENSCAFG00845016180	6	2	2	0
ENSCAFG00845016181	1464	1369	1580	1538
ENSCAFG00845028168	0	0	0	0
ENSCAFG00845028167	0	0	0	0
ENSCAFG00845004199	0	9	2	3
ENSCAFG00845004198	0	0	0	0
ENSCAFG00845028169	0	0	0	0
ENSCAFG00845004197	613	563	634	676
ENSCAFG00845028164	2413	2334	2060	2251
ENSCAFG00845004196	2269	2093	2004	2092
ENSCAFG00845028163	0	0	1	0
ENSCAFG00845004195	1050	1053	864	905
ENSCAFG00845028166	3	4	2	1
ENSCAFG00845004194	0	0	0	0
ENSCAFG00845028165	1316	1353	1305	1264
ENSCAFG00845004193	0	0	2	2
ENSCAFG00845028160	488	441	427	465
ENSCAFG00845004192	199	189	111	144
ENSCAFG00845004191	1409	1381	1629	1694
ENSCAFG00845028162	0	0	0	0
ENSCAFG00845004190	0	0	0	0
ENSCAFG00845028161	0	0	0	0
ENSCAFG00845016179	0	0	0	0
ENSCAFG00845016177	0	0	0	0
ENSCAFG00845016178	0	0	0	0
ENSCAFG00845016175	0	1	0	0
ENSCAFG00845016176	6	5	7	9
ENSCAFG00845016173	2	1	1	7

ENSCAFG00845016174	1485	1398	1524	1528
ENSCAFG00845016171	0	0	0	0
ENSCAFG00845016172	48	50	38	40
ENSCAFG00845016170	245	226	235	223
ENSCAFG00845028157	0	0	0	1
ENSCAFG00845028156	0	0	0	0
ENSCAFG00845028159	0	0	0	0
ENSCAFG00845028158	0	0	0	0
ENSCAFG00845028153	2	8	3	7
ENSCAFG00845028152	593	551	544	559
ENSCAFG00845028155	262	247	247	257
ENSCAFG00845028154	326	338	369	353
ENSCAFG00845028151	564	579	584	617
ENSCAFG00845028150	0	0	0	0
ENSCAFG00845016168	240	207	196	176
ENSCAFG00845016169	510	433	523	420
ENSCAFG00845016166	13	14	6	11
ENSCAFG00845016167	208	193	160	189
ENSCAFG00845016164	0	0	0	0
ENSCAFG00845016165	1657	1566	1845	1928
ENSCAFG00845016162	0	0	0	0
ENSCAFG00845016163	4	1	6	3
ENSCAFG00845016160	2	0	0	0
ENSCAFG00845016161	92	106	101	101
ENSCAFG00845028149	20	20	20	21
ENSCAFG00845004179	0	0	0	0
ENSCAFG00845028146	2	4	3	2
ENSCAFG00845004178	0	0	0	0
ENSCAFG00845028145	316	317	316	373
ENSCAFG00845004177	0	0	0	0
ENSCAFG00845028148	1360	1249	1482	1371
ENSCAFG00845004176	0	0	5	4
ENSCAFG00845028147	174	190	194	169
ENSCAFG00845004175	25	27	24	34
ENSCAFG00845028142	0	0	0	0
ENSCAFG00845004174	4	0	1	4
ENSCAFG00845028141	0	0	0	0
ENSCAFG00845004173	0	0	0	0
ENSCAFG00845028144	0	0	0	0
ENSCAFG00845004172	1	2	2	2
ENSCAFG00845028143	15	6	21	26

ENSCAFG00845004171	2	3	1	2
ENSCAFG00845004170	4	4	1	2
ENSCAFG00845028140	0	0	0	0
ENSCAFG00845016157	8	10	6	8
ENSCAFG00845016158	4	2	1	1
ENSCAFG00845016155	0	0	0	1
ENSCAFG00845016156	322	354	300	325
ENSCAFG00845016153	822	739	797	820
ENSCAFG00845016154	1756	1764	1881	1885
ENSCAFG00845016151	0	0	0	0
ENSCAFG00845016152	0	0	0	0
ENSCAFG00845016150	695	600	472	437
ENSCAFG00845028139	0	0	0	0
ENSCAFG00845028138	0	0	0	0
ENSCAFG00845028135	1	1	0	2
ENSCAFG00845004189	990	1035	1133	1179
ENSCAFG00845028134	177	171	147	188
ENSCAFG00845004188	2115	2022	1931	2088
ENSCAFG00845028137	3	3	2	3
ENSCAFG00845004187	11	7	2	1
ENSCAFG00845028136	2	2	1	4
ENSCAFG00845004186	3	4	0	1
ENSCAFG00845028131	0	0	0	0
ENSCAFG00845004185	0	0	0	0
ENSCAFG00845028130	11359	11404	11555	11826
ENSCAFG00845004184	6	1	12	4
ENSCAFG00845028133	7	1	8	3
ENSCAFG00845004183	2632	2450	2663	2643
ENSCAFG00845028132	313	264	325	302
ENSCAFG00845004182	3030	2965	2918	2961
ENSCAFG00845004181	1292	1186	1191	1181
ENSCAFG00845004180	0	1	0	0
ENSCAFG00845016159	0	0	0	0
ENSCAFG00845028098	264	246	265	270
ENSCAFG00845028097	0	0	0	0
ENSCAFG00845028099	0	0	0	0
ENSCAFG00845028094	1339	1290	1321	1282
ENSCAFG00845028093	0	0	0	0
ENSCAFG00845028096	1	1	0	0
ENSCAFG00845028095	360	290	341	377
ENSCAFG00845028090	295	281	278	287

ENSCAFG00845028092	236	208	252	206
ENSCAFG00845028091	5008	4783	5017	5064
ENSCAFG00845018721	485	445	499	534
ENSCAFG00845018720	56	41	35	41
ENSCAFG00845006740	1	3	3	1
ENSCAFG00845006741	0	1	1	1
ENSCAFG00845006742	1245	1248	1424	1360
ENSCAFG00845006743	4650	4571	4323	4498
ENSCAFG00845006744	3	1	2	2
ENSCAFG00845006745	1341	1321	1368	1446
ENSCAFG00845006746	469	463	414	438
ENSCAFG00845006747	284	288	284	328
ENSCAFG00845006748	141	154	135	128
ENSCAFG00845006749	860	800	739	766
ENSCAFG00845018729	7	4	10	10
ENSCAFG00845018728	15	11	16	12
ENSCAFG00845018727	388	375	372	315
ENSCAFG00845018726	4413	4234	4219	4516
ENSCAFG00845018725	266	287	219	220
ENSCAFG00845018724	1122	1005	1048	1046
ENSCAFG00845018723	972	977	819	911
ENSCAFG00845018722	0	0	0	0
ENSCAFG00845018710	44	26	28	27
ENSCAFG00845006730	1	2	2	0
ENSCAFG00845006731	0	0	0	0
ENSCAFG00845006732	0	0	0	0
ENSCAFG00845006733	3	4	3	13
ENSCAFG00845006734	0	0	0	0
ENSCAFG00845006735	632	665	449	450
ENSCAFG00845006736	0	0	0	0
ENSCAFG00845006737	0	0	0	0
ENSCAFG00845006738	2	0	0	5
ENSCAFG00845006739	0	0	0	0
ENSCAFG00845018719	1	1	4	1
ENSCAFG00845018718	18	4	11	14
ENSCAFG00845018717	0	0	1	0
ENSCAFG00845018716	0	0	0	0
ENSCAFG00845018715	0	0	0	0
ENSCAFG00845018714	0	0	0	0
ENSCAFG00845018713	165	163	157	142
ENSCAFG00845018712	418	401	340	332

ENSCAFG00845018711	517	542	464	506
ENSCAFG00845006720	1975	1879	1806	1766
ENSCAFG00845006721	1272	1283	1263	1314
ENSCAFG00845006722	755	694	699	657
ENSCAFG00845006723	204	241	145	104
ENSCAFG00845006724	8	9	9	6
ENSCAFG00845006725	4	0	6	4
ENSCAFG00845006726	0	0	0	0
ENSCAFG00845006727	3278	3238	3356	3593
ENSCAFG00845018709	627	558	556	522
ENSCAFG00845006728	273	232	234	225
ENSCAFG00845018708	710	748	736	656
ENSCAFG00845006729	2	1	3	1
ENSCAFG00845018707	0	0	0	0
ENSCAFG00845018706	0	0	0	0
ENSCAFG00845018705	0	0	0	0
ENSCAFG00845018704	0	0	0	0
ENSCAFG00845018703	275	238	373	349
ENSCAFG00845018702	4	2	4	2
ENSCAFG00845018701	1024	1084	1066	1222
ENSCAFG00845018700	3	3	11	6
ENSCAFG00845006710	1509	1503	1378	1438
ENSCAFG00845006711	0	0	0	0
ENSCAFG00845006712	5	2	1	0
ENSCAFG00845006713	2	0	0	0
ENSCAFG00845006714	0	0	0	0
ENSCAFG00845006715	0	2	0	1
ENSCAFG00845006716	0	0	0	0
ENSCAFG00845006717	0	0	0	0
ENSCAFG00845006718	382	363	356	364
ENSCAFG00845006719	736	683	680	775
ENSCAFG00845006700	1	5	0	3
ENSCAFG00845006701	168	152	152	171
ENSCAFG00845006702	1187	1089	1079	1104
ENSCAFG00845006703	124	135	144	166
ENSCAFG00845006704	0	6	0	0
ENSCAFG00845006705	99	113	66	64
ENSCAFG00845006706	0	0	0	2
ENSCAFG00845006707	244	273	268	278
ENSCAFG00845006708	1	1	0	0
ENSCAFG00845006709	165	151	181	156

ENSCAFG00845016267	7	12	13	11
ENSCAFG00845016268	0	0	0	0
ENSCAFG00845016265	232	233	245	274
ENSCAFG00845016266	0	0	0	0
ENSCAFG00845016263	89	86	80	70
ENSCAFG00845016264	0	0	0	0
ENSCAFG00845016261	1	0	0	0
ENSCAFG00845016262	531	518	541	601
ENSCAFG00845016260	66	84	63	62
ENSCAFG00845028249	0	0	0	0
ENSCAFG00845028248	334	372	397	404
ENSCAFG00845004279	0	0	1	4
ENSCAFG00845004278	23	16	15	29
ENSCAFG00845028245	0	2	0	0
ENSCAFG00845004277	0	10	1	1
ENSCAFG00845028244	654	641	678	673
ENSCAFG00845004276	164	184	185	163
ENSCAFG00845028247	861	833	841	880
ENSCAFG00845004275	74	78	89	96
ENSCAFG00845028246	0	0	1	0
ENSCAFG00845004274	11	5	5	0
ENSCAFG00845028241	0	0	0	0
ENSCAFG00845004273	1	0	0	1
ENSCAFG00845028240	0	0	0	0
ENSCAFG00845004272	0	0	0	0
ENSCAFG00845028243	0	0	0	0
ENSCAFG00845004271	1980	1872	1798	1916
ENSCAFG00845028242	13	9	7	16
ENSCAFG00845004270	4	1	0	5
ENSCAFG00845016269	1	1	1	4
ENSCAFG00845016256	163	145	146	171
ENSCAFG00845016257	692	605	761	790
ENSCAFG00845016254	0	0	0	0
ENSCAFG00845016255	0	0	0	0
ENSCAFG00845016252	1066	1001	889	1001
ENSCAFG00845016253	737	713	701	770
ENSCAFG00845016250	231	258	204	232
ENSCAFG00845016251	0	0	0	0
ENSCAFG00845028238	310	341	271	226
ENSCAFG00845028237	1245	1217	1270	1373
ENSCAFG00845028239	47	41	45	51

ENSCAFG00845004289	223	198	191	229
ENSCAFG00845028234	37	39	31	24
ENSCAFG00845004288	13	18	13	21
ENSCAFG00845028233	292	326	305	351
ENSCAFG00845004287	0	0	0	0
ENSCAFG00845028236	1	0	1	2
ENSCAFG00845004286	1499	1425	1024	965
ENSCAFG00845028235	443	488	399	469
ENSCAFG00845004285	1043	1015	898	975
ENSCAFG00845028230	0	0	0	0
ENSCAFG00845004284	0	0	0	0
ENSCAFG00845004283	0	0	0	0
ENSCAFG00845028232	0	1	0	0
ENSCAFG00845004282	188	179	231	203
ENSCAFG00845028231	0	0	0	0
ENSCAFG00845004281	116	129	93	102
ENSCAFG00845004280	2266	2184	2220	2308
ENSCAFG00845016258	2	2	2	1
ENSCAFG00845016259	558	450	447	487
ENSCAFG00845016245	440	414	433	380
ENSCAFG00845016246	1226	1201	1224	1198
ENSCAFG00845016243	332	322	69	77
ENSCAFG00845016244	0	0	0	0
ENSCAFG00845016241	3	0	2	0
ENSCAFG00845016242	1	0	4	0
ENSCAFG00845016240	52	46	35	36
ENSCAFG00845028227	4	2	6	6
ENSCAFG00845004259	3729	3683	3713	4005
ENSCAFG00845028226	0	0	0	0
ENSCAFG00845004258	0	0	1	1
ENSCAFG00845028229	5049	4830	4501	4651
ENSCAFG00845004257	0	0	0	0
ENSCAFG00845028228	1284	1198	1229	1310
ENSCAFG00845004256	407	418	272	286
ENSCAFG00845028223	641	646	581	618
ENSCAFG00845004255	12	5	7	8
ENSCAFG00845028222	1	0	0	2
ENSCAFG00845004254	0	0	0	0
ENSCAFG00845028225	0	0	0	0
ENSCAFG00845004253	0	0	0	0
ENSCAFG00845028224	2745	2769	2627	2470

ENSCAFG00845004252	327	352	405	383
ENSCAFG00845004251	1	3	2	0
ENSCAFG00845004250	766	644	639	630
ENSCAFG00845028221	36	49	34	46
ENSCAFG00845028220	2	8	0	5
ENSCAFG00845016249	14	7	10	7
ENSCAFG00845016247	34	30	57	40
ENSCAFG00845016248	722	612	714	793
ENSCAFG00845016234	22	21	26	24
ENSCAFG00845018897	0	0	0	0
ENSCAFG00845016235	45	23	38	39
ENSCAFG00845018896	1	0	0	1
ENSCAFG00845016232	0	0	0	0
ENSCAFG00845018895	0	0	0	0
ENSCAFG00845016233	29	33	31	24
ENSCAFG00845018894	0	0	0	0
ENSCAFG00845016230	1255	1230	1237	1308
ENSCAFG00845018893	1	0	0	0
ENSCAFG00845016231	0	0	0	0
ENSCAFG00845018892	339	337	359	375
ENSCAFG00845018891	456	448	461	439
ENSCAFG00845018890	0	0	0	0
ENSCAFG00845028219	200	169	188	190
ENSCAFG00845028216	283	266	241	288
ENSCAFG00845028215	0	0	0	0
ENSCAFG00845004269	1444	1373	1029	1130
ENSCAFG00845028218	63	105	60	61
ENSCAFG00845004268	705	698	700	687
ENSCAFG00845028217	1703	1560	1562	1636
ENSCAFG00845004267	0	0	0	0
ENSCAFG00845028212	287	279	367	343
ENSCAFG00845004266	0	2	1	2
ENSCAFG00845028211	420	370	469	499
ENSCAFG00845004265	0	0	0	0
ENSCAFG00845028214	900	823	831	761
ENSCAFG00845004264	9	3	7	2
ENSCAFG00845028213	0	0	0	0
ENSCAFG00845004263	643	561	588	584
ENSCAFG00845004262	0	1	0	1
ENSCAFG00845004261	3	3	0	1
ENSCAFG00845028210	1365	1431	1345	1287

ENSCAFG00845004260	0	0	0	0
ENSCAFG00845016238	0	0	0	0
ENSCAFG00845016239	0	0	0	0
ENSCAFG00845016236	110	85	90	94
ENSCAFG00845018899	0	0	0	0
ENSCAFG00845016237	0	0	1	0
ENSCAFG00845018898	1308	1193	674	632
ENSCAFG00845016223	0	0	0	0
ENSCAFG00845018886	0	0	0	0
ENSCAFG00845016224	0	0	0	1
ENSCAFG00845018885	4	1	3	6
ENSCAFG00845016221	15	13	4	16
ENSCAFG00845018884	0	0	0	0
ENSCAFG00845016222	914	993	950	953
ENSCAFG00845018883	180	170	160	143
ENSCAFG00845018882	696	677	636	673
ENSCAFG00845016220	5	6	8	6
ENSCAFG00845018881	0	0	0	0
ENSCAFG00845018880	3426	3374	3267	3347
ENSCAFG00845028209	61	44	48	73
ENSCAFG00845028208	0	0	0	0
ENSCAFG00845004239	645	662	600	570
ENSCAFG00845004238	813	823	692	708
ENSCAFG00845028205	238	205	198	214
ENSCAFG00845004237	2	0	0	2
ENSCAFG00845028204	0	0	0	0
ENSCAFG00845004236	305	348	302	321
ENSCAFG00845028207	0	0	0	0
ENSCAFG00845004235	205	250	194	194
ENSCAFG00845028206	1	6	2	2
ENSCAFG00845004234	22	26	22	21
ENSCAFG00845028201	1164	1225	1103	1155
ENSCAFG00845004233	1	0	5	2
ENSCAFG00845028200	2	0	1	0
ENSCAFG00845004232	537	526	500	492
ENSCAFG00845028203	1772	1655	1645	1773
ENSCAFG00845004231	1	0	0	0
ENSCAFG00845028202	0	0	0	0
ENSCAFG00845004230	30	29	27	26
ENSCAFG00845016229	2297	2263	2573	2431
ENSCAFG00845016227	379	409	356	392

ENSCAFG00845016228	1	2	8	2
ENSCAFG00845018889	359	315	343	333
ENSCAFG00845016225	0	0	0	0
ENSCAFG00845018888	975	948	876	935
ENSCAFG00845016226	79	77	63	61
ENSCAFG00845018887	3215	3075	2959	2995
ENSCAFG00845016212	0	0	0	0
ENSCAFG00845018875	1527	1348	1268	1322
ENSCAFG00845016213	0	1	0	2
ENSCAFG00845018874	0	0	3	1
ENSCAFG00845016210	2188	2159	1698	1785
ENSCAFG00845018873	1265	1276	1219	1183
ENSCAFG00845016211	27	22	21	32
ENSCAFG00845018872	735	683	689	626
ENSCAFG00845018871	0	0	0	0
ENSCAFG00845018870	0	0	0	0
ENSCAFG00845006890	0	0	0	1
ENSCAFG00845006891	252	269	259	237
ENSCAFG00845006892	0	0	0	0
ENSCAFG00845004249	353	371	371	397
ENSCAFG00845006893	895	856	805	736
ENSCAFG00845004248	0	0	0	0
ENSCAFG00845006894	449	429	350	373
ENSCAFG00845004247	0	0	0	0
ENSCAFG00845006895	17	6	7	12
ENSCAFG00845004246	1360	1349	1264	1267
ENSCAFG00845006896	0	1	0	1
ENSCAFG00845004245	6	13	2	6
ENSCAFG00845006897	2268	2064	1763	1779
ENSCAFG00845004244	0	0	0	0
ENSCAFG00845006898	0	0	0	0
ENSCAFG00845004243	0	0	0	0
ENSCAFG00845006899	5	1	7	1
ENSCAFG00845004242	19	22	39	37
ENSCAFG00845004241	870	832	922	922
ENSCAFG00845004240	60	71	70	38
ENSCAFG00845016218	1576	1482	1543	1757
ENSCAFG00845016219	0	0	1	1
ENSCAFG00845016216	177	149	173	195
ENSCAFG00845018879	66	85	108	93
ENSCAFG00845016217	9	4	8	12

ENSCAFG00845018878	0	0	1	1
ENSCAFG00845016214	0	0	0	0
ENSCAFG00845018877	0	0	0	0
ENSCAFG00845016215	0	0	0	0
ENSCAFG00845018876	7	8	4	4
ENSCAFG00845016201	6	4	11	7
ENSCAFG00845018864	16	12	14	14
ENSCAFG00845016202	46	76	55	48
ENSCAFG00845018863	0	0	0	0
ENSCAFG00845018862	0	0	0	0
ENSCAFG00845016200	293	296	287	311
ENSCAFG00845018861	1469	1346	1422	1381
ENSCAFG00845018860	49	39	50	42
ENSCAFG00845004219	1245	1158	1277	1307
ENSCAFG00845004218	6	11	11	8
ENSCAFG00845006880	2	0	0	2
ENSCAFG00845004217	16	8	5	10
ENSCAFG00845006881	5	3	7	1
ENSCAFG00845004216	0	0	0	0
ENSCAFG00845006882	0	0	0	0
ENSCAFG00845004215	0	0	0	0
ENSCAFG00845006883	69	53	67	56
ENSCAFG00845004214	2	2	1	3
ENSCAFG00845006884	792	734	775	709
ENSCAFG00845004213	41	41	40	37
ENSCAFG00845006885	0	0	0	0
ENSCAFG00845004212	0	0	0	0
ENSCAFG00845006886	23	24	14	12
ENSCAFG00845004211	266	281	298	243
ENSCAFG00845006887	0	0	0	0
ENSCAFG00845004210	49	69	82	70
ENSCAFG00845006888	337	338	399	392
ENSCAFG00845006889	1077	989	977	1010
ENSCAFG00845016209	0	0	0	0
ENSCAFG00845016207	208	220	228	279
ENSCAFG00845016208	17	34	29	35
ENSCAFG00845018869	1	0	0	0
ENSCAFG00845016205	1	0	0	1
ENSCAFG00845018868	1263	1235	1196	1261
ENSCAFG00845016206	13	11	13	10
ENSCAFG00845018867	0	0	0	0

ENSCAFG00845016203	1539	1575	1268	1279
ENSCAFG00845018866	0	0	0	0
ENSCAFG00845016204	0	0	0	0
ENSCAFG00845018865	0	0	0	0
ENSCAFG00845018853	0	0	0	0
ENSCAFG00845018852	23	45	51	27
ENSCAFG00845018851	3	3	7	5
ENSCAFG00845018850	0	0	0	0
ENSCAFG00845004229	225	240	189	218
ENSCAFG00845004228	0	0	0	0
ENSCAFG00845006870	6	3	3	6
ENSCAFG00845004227	0	0	0	0
ENSCAFG00845006871	6	6	7	2
ENSCAFG00845004226	397	397	395	408
ENSCAFG00845006872	0	0	0	0
ENSCAFG00845004225	3	8	1	0
ENSCAFG00845006873	38	58	36	53
ENSCAFG00845004224	1	0	0	0
ENSCAFG00845006874	5	4	8	7
ENSCAFG00845004223	0	0	0	0
ENSCAFG00845006875	66	53	41	36
ENSCAFG00845004222	2051	2058	2012	2064
ENSCAFG00845006876	791	708	626	675
ENSCAFG00845004221	38	35	43	40
ENSCAFG00845006877	462	476	417	400
ENSCAFG00845004220	147	150	171	173
ENSCAFG00845006878	129	128	185	195
ENSCAFG00845006879	1814	1810	1682	1678
ENSCAFG00845018859	0	0	0	0
ENSCAFG00845018858	653	663	599	606
ENSCAFG00845018857	0	0	0	0
ENSCAFG00845018856	300	233	304	238
ENSCAFG00845018855	0	0	0	0
ENSCAFG00845018854	781	715	818	828
ENSCAFG00845028299	4	0	0	4
ENSCAFG00845028296	1	0	6	0
ENSCAFG00845028295	0	0	0	0
ENSCAFG00845028298	0	0	0	0
ENSCAFG00845028297	15	21	15	11
ENSCAFG00845028292	2622	2508	2557	2556
ENSCAFG00845028291	206	210	234	254

ENSCAFG00845028294	1115	1020	1198	1134
ENSCAFG00845028293	770	697	1011	990
ENSCAFG00845028290	12	14	18	15
ENSCAFG00845028289	0	0	0	0
ENSCAFG00845028288	709	707	648	679
ENSCAFG00845028285	2201	2292	2283	2251
ENSCAFG00845028284	0	1	0	0
ENSCAFG00845028287	0	0	0	0
ENSCAFG00845028286	32	26	26	27
ENSCAFG00845028281	0	0	0	0
ENSCAFG00845028280	0	0	0	0
ENSCAFG00845028283	3	8	2	6
ENSCAFG00845028282	328	298	267	347
ENSCAFG00845016298	21	26	25	20
ENSCAFG00845016299	499	478	509	467
ENSCAFG00845016296	493	446	377	453
ENSCAFG00845016297	5	2	1	4
ENSCAFG00845016294	0	0	0	0
ENSCAFG00845016295	0	0	0	0
ENSCAFG00845016292	29	27	52	47
ENSCAFG00845016293	0	0	0	0
ENSCAFG00845016290	876	947	955	1006
ENSCAFG00845016291	8	4	8	11
ENSCAFG00845028278	0	0	0	0
ENSCAFG00845028277	364	350	377	379
ENSCAFG00845028279	15369	15064	16183	16379
ENSCAFG00845028274	1285	1135	1164	1191
ENSCAFG00845028273	4	0	0	1
ENSCAFG00845028276	1	5	1	9
ENSCAFG00845028275	2	0	2	1
ENSCAFG00845028270	0	0	0	0
ENSCAFG00845028272	0	0	0	0
ENSCAFG00845028271	0	0	0	0
ENSCAFG00845016289	0	0	0	0
ENSCAFG00845016287	2	2	2	0
ENSCAFG00845016288	1219	1169	1091	1123
ENSCAFG00845016285	0	0	0	0
ENSCAFG00845016286	0	0	0	0
ENSCAFG00845016283	0	0	0	2
ENSCAFG00845016284	0	0	0	0
ENSCAFG00845016281	21	12	23	24

ENSCAFG00845016282	1047	976	1015	1017
ENSCAFG00845016280	0	0	0	0
ENSCAFG00845028267	0	0	0	0
ENSCAFG00845004299	41	49	35	32
ENSCAFG00845028266	372	424	397	435
ENSCAFG00845004298	170	154	113	91
ENSCAFG00845028269	3	2	6	4
ENSCAFG00845004297	17	21	16	13
ENSCAFG00845028268	862	753	939	948
ENSCAFG00845004296	1341	1207	1124	1180
ENSCAFG00845028263	938	878	849	997
ENSCAFG00845004295	0	0	0	0
ENSCAFG00845028262	17	25	16	16
ENSCAFG00845004294	0	0	0	0
ENSCAFG00845028265	1	0	2	5
ENSCAFG00845004293	390	416	424	401
ENSCAFG00845028264	0	0	0	0
ENSCAFG00845004292	0	0	0	0
ENSCAFG00845004291	532	498	509	543
ENSCAFG00845004290	0	0	0	0
ENSCAFG00845028261	1104	1155	1090	1149
ENSCAFG00845028260	293	334	312	326
ENSCAFG00845016278	618	504	458	394
ENSCAFG00845016279	507	446	466	440
ENSCAFG00845016276	1422	1241	1309	1307
ENSCAFG00845016277	26	24	29	15
ENSCAFG00845016274	406	417	382	370
ENSCAFG00845016275	13	8	1	4
ENSCAFG00845016272	14	11	18	14
ENSCAFG00845016273	29	34	18	27
ENSCAFG00845016270	5	12	9	10
ENSCAFG00845016271	1437	1363	1322	1383
ENSCAFG00845028259	122	160	155	145
ENSCAFG00845028256	1490	1431	1446	1476
ENSCAFG00845028255	134	117	115	121
ENSCAFG00845028258	1236	1196	1265	1257
ENSCAFG00845028257	0	0	0	0
ENSCAFG00845028252	1074	1114	1051	992
ENSCAFG00845028251	0	0	0	1
ENSCAFG00845028254	8260	8090	8146	7856
ENSCAFG00845028253	7192	6954	6650	6543

ENSCAFG00845028250	0	0	0	0
ENSCAFG00845018842	0	0	0	0
ENSCAFG00845018841	0	0	0	0
ENSCAFG00845018840	0	0	0	0
ENSCAFG00845006860	0	0	0	0
ENSCAFG00845006861	0	0	0	0
ENSCAFG00845006862	1	5	3	8
ENSCAFG00845006863	0	0	0	0
ENSCAFG00845006864	0	0	0	0
ENSCAFG00845006865	213	184	154	199
ENSCAFG00845006866	2	2	5	4
ENSCAFG00845006867	0	0	4	0
ENSCAFG00845006868	392	451	354	342
ENSCAFG00845006869	54	67	67	77
ENSCAFG00845018849	891	840	854	842
ENSCAFG00845018848	34	17	25	23
ENSCAFG00845018847	0	0	0	0
ENSCAFG00845018846	61	49	65	51
ENSCAFG00845018845	1	0	0	1
ENSCAFG00845018844	11	24	13	16
ENSCAFG00845018843	1	0	0	0
ENSCAFG00845018831	1505	1434	1445	1454
ENSCAFG00845018830	0	0	0	0
ENSCAFG00845004209	19	25	40	28
ENSCAFG00845004208	846	761	752	753
ENSCAFG00845004207	3396	3196	3373	3300
ENSCAFG00845004206	0	0	0	0
ENSCAFG00845004205	5	2	1	2
ENSCAFG00845004204	1	1	1	1
ENSCAFG00845006850	460	420	439	444
ENSCAFG00845004203	245	250	225	263
ENSCAFG00845006851	0	0	0	0
ENSCAFG00845004202	32	39	37	35
ENSCAFG00845006852	63	60	55	66
ENSCAFG00845004201	0	0	0	0
ENSCAFG00845006853	110	122	75	67
ENSCAFG00845004200	0	0	0	0
ENSCAFG00845006854	508	536	496	501
ENSCAFG00845006855	9	0	0	0
ENSCAFG00845006856	565	591	524	560
ENSCAFG00845006857	0	0	0	0

ENSCAFG00845006858	119	102	153	142
ENSCAFG00845006859	753	708	724	677
ENSCAFG00845018839	2055	1942	1828	2022
ENSCAFG00845018838	2662	2387	2257	2378
ENSCAFG00845018837	0	0	0	0
ENSCAFG00845018836	1	3	1	3
ENSCAFG00845018835	0	0	0	0
ENSCAFG00845018834	78	126	85	82
ENSCAFG00845018833	0	0	0	0
ENSCAFG00845018832	503	438	468	545
ENSCAFG00845018820	0	1	1	0
ENSCAFG00845006840	70	61	82	66
ENSCAFG00845006841	322	330	337	314
ENSCAFG00845006842	0	0	1	1
ENSCAFG00845006843	593	613	704	724
ENSCAFG00845006844	34	52	41	40
ENSCAFG00845006845	0	3	0	0
ENSCAFG00845006846	0	0	0	0
ENSCAFG00845006847	3408	3519	3567	3497
ENSCAFG00845006848	259	278	210	242
ENSCAFG00845006849	0	0	0	0
ENSCAFG00845018829	0	0	0	0
ENSCAFG00845018828	0	0	1	0
ENSCAFG00845018827	2	4	3	10
ENSCAFG00845018826	3	1	2	1
ENSCAFG00845018825	437	418	371	391
ENSCAFG00845018824	0	0	0	0
ENSCAFG00845018823	29	34	23	27
ENSCAFG00845018822	74	63	56	45
ENSCAFG00845018821	137	115	134	86
ENSCAFG00845006830	0	0	1	1
ENSCAFG00845006831	52	58	79	75
ENSCAFG00845006832	699	640	605	583
ENSCAFG00845006833	0	0	0	0
ENSCAFG00845006834	0	0	0	0
ENSCAFG00845006835	0	0	0	0
ENSCAFG00845006836	343	326	282	306
ENSCAFG00845006837	1	1	1	1
ENSCAFG00845018819	0	0	0	0
ENSCAFG00845006838	0	0	0	0
ENSCAFG00845018818	192	218	199	179

ENSCAFG00845006839	0	0	0	0
ENSCAFG00845018817	1877	1766	1747	1805
ENSCAFG00845018816	1251	1200	1059	1062
ENSCAFG00845018815	447	398	402	312
ENSCAFG00845018814	0	0	0	0
ENSCAFG00845018813	0	0	0	0
ENSCAFG00845018812	670	629	618	702
ENSCAFG00845018811	597	578	599	562
ENSCAFG00845018810	468	461	463	445
ENSCAFG00845006820	901	846	877	870
ENSCAFG00845006821	0	0	0	0
ENSCAFG00845006822	0	0	0	0
ENSCAFG00845006823	182	212	171	204
ENSCAFG00845006824	42	46	44	33
ENSCAFG00845006825	23	27	21	27
ENSCAFG00845018809	1	0	0	0
ENSCAFG00845006826	0	0	0	0
ENSCAFG00845018808	285	287	296	246
ENSCAFG00845006827	1423	1335	1347	1377
ENSCAFG00845018807	0	0	0	0
ENSCAFG00845006828	617	602	567	593
ENSCAFG00845018806	0	0	0	0
ENSCAFG00845006829	3	2	0	1
ENSCAFG00845018805	342	333	322	315
ENSCAFG00845018804	7914	7382	7719	8008
ENSCAFG00845018803	0	0	0	0
ENSCAFG00845018802	6	2	3	8
ENSCAFG00845018801	235	215	135	160
ENSCAFG00845018800	256	223	391	382
ENSCAFG00845006810	13	23	19	21
ENSCAFG00845006811	1064	968	1036	1057
ENSCAFG00845006812	0	0	0	0
ENSCAFG00845006813	0	0	0	0
ENSCAFG00845006814	0	0	0	0
ENSCAFG00845006815	1156	1204	1116	1194
ENSCAFG00845006816	0	0	0	0
ENSCAFG00845006817	0	0	0	0
ENSCAFG00845006818	17	19	14	18
ENSCAFG00845006819	2	1	4	0
ENSCAFG00845006800	0	0	0	0
ENSCAFG00845006801	104	80	82	76

ENSCAFG00845006802	2168	2107	2060	2373
ENSCAFG00845006803	1	0	1	0
ENSCAFG00845006804	3	3	2	4
ENSCAFG00845006805	4	4	2	3
ENSCAFG00845006806	565	557	845	799
ENSCAFG00845006807	495	415	499	481
ENSCAFG00845006808	0	0	0	0
ENSCAFG00845006809	869	770	779	716
ENSCAFG00845016388	2648	2538	2296	2455
ENSCAFG00845016389	410	373	404	451
ENSCAFG00845016386	116	79	78	64
ENSCAFG00845016387	1629	1444	1999	1978
ENSCAFG00845016384	1836	1851	1713	1849
ENSCAFG00845016385	0	0	0	0
ENSCAFG00845016382	0	0	0	0
ENSCAFG00845016383	0	0	0	0
ENSCAFG00845016380	335	387	337	398
ENSCAFG00845016381	0	0	0	0
ENSCAFG00845028369	234	227	213	257
ENSCAFG00845004399	0	0	0	0
ENSCAFG00845028366	1178	1059	1007	986
ENSCAFG00845004398	0	0	0	0
ENSCAFG00845028365	0	0	0	0
ENSCAFG00845004397	417	393	367	366
ENSCAFG00845028368	4242	3931	4005	4035
ENSCAFG00845004396	0	1	0	1
ENSCAFG00845028367	3905	3914	3529	3504
ENSCAFG00845004395	0	0	0	0
ENSCAFG00845028362	2581	2458	2455	2368
ENSCAFG00845004394	0	0	0	0
ENSCAFG00845028361	1070	991	1072	1207
ENSCAFG00845004393	0	0	0	0
ENSCAFG00845028364	0	0	0	1
ENSCAFG00845004392	0	0	0	0
ENSCAFG00845028363	0	0	0	0
ENSCAFG00845004391	1903	1793	2323	2286
ENSCAFG00845004390	0	0	0	0
ENSCAFG00845028360	1469	1337	1342	1310
ENSCAFG00845016377	8	10	8	4
ENSCAFG00845016378	1790	1779	1843	2012
ENSCAFG00845016375	12	16	30	22

ENSCAFG00845016376	0	0	0	0
ENSCAFG00845016373	0	0	0	0
ENSCAFG00845016374	0	0	0	0
ENSCAFG00845016371	63	35	47	61
ENSCAFG00845016372	0	0	0	0
ENSCAFG00845016370	0	0	0	0
ENSCAFG00845028359	1	0	0	6
ENSCAFG00845028358	0	0	0	0
ENSCAFG00845028355	0	0	0	0
ENSCAFG00845028354	0	0	0	1
ENSCAFG00845028357	981	898	896	862
ENSCAFG00845028356	0	0	0	0
ENSCAFG00845028351	107	89	87	106
ENSCAFG00845028350	0	0	0	0
ENSCAFG00845028353	1812	1609	1541	1611
ENSCAFG00845028352	1372	1327	1378	1480
ENSCAFG00845016379	187	199	188	199
ENSCAFG00845016366	9	7	3	8
ENSCAFG00845016367	0	0	0	0
ENSCAFG00845016364	39	45	15	39
ENSCAFG00845016365	1458	1427	1232	1170
ENSCAFG00845016362	0	0	0	0
ENSCAFG00845016363	60	65	41	39
ENSCAFG00845016360	10	7	6	0
ENSCAFG00845016361	40	45	63	38
ENSCAFG00845028348	0	0	0	0
ENSCAFG00845028347	57	48	50	47
ENSCAFG00845004379	37	42	51	42
ENSCAFG00845004378	70	62	39	40
ENSCAFG00845028349	0	0	0	0
ENSCAFG00845004377	29	27	32	45
ENSCAFG00845028344	0	0	0	0
ENSCAFG00845004376	0	0	0	0
ENSCAFG00845028343	0	0	0	0
ENSCAFG00845004375	1453	1331	1287	1226
ENSCAFG00845028346	10	17	33	27
ENSCAFG00845004374	0	0	0	0
ENSCAFG00845028345	0	0	0	0
ENSCAFG00845004373	803	709	721	741
ENSCAFG00845028340	0	0	0	0
ENSCAFG00845004372	611	582	688	698

ENSCAFG00845004371	0	0	0	0
ENSCAFG00845028342	0	0	0	0
ENSCAFG00845004370	247	231	172	170
ENSCAFG00845028341	1714	1600	1664	1624
ENSCAFG00845016368	0	0	0	0
ENSCAFG00845016369	3040	2978	2710	2766
ENSCAFG00845016355	3	2	0	0
ENSCAFG00845016356	0	0	0	0
ENSCAFG00845016353	221	233	189	187
ENSCAFG00845016354	1569	1567	1469	1436
ENSCAFG00845016351	69	94	58	48
ENSCAFG00845016352	0	0	1	1
ENSCAFG00845016350	621	599	540	548
ENSCAFG00845028337	0	1	0	0
ENSCAFG00845028336	7	6	2	9
ENSCAFG00845028339	782	683	775	786
ENSCAFG00845004389	0	0	0	0
ENSCAFG00845028338	1301	1180	1102	1186
ENSCAFG00845004388	0	0	0	0
ENSCAFG00845028333	6484	6545	6547	6471
ENSCAFG00845004387	5	2	2	3
ENSCAFG00845028332	1295	1273	1258	1215
ENSCAFG00845004386	7374	7004	6855	7215
ENSCAFG00845028335	1995	1826	1894	2045
ENSCAFG00845004385	0	0	2	0
ENSCAFG00845028334	0	0	0	0
ENSCAFG00845004384	0	0	0	0
ENSCAFG00845004383	8	1	1	5
ENSCAFG00845004382	250	285	223	247
ENSCAFG00845028331	0	0	0	0
ENSCAFG00845004381	13	4	12	6
ENSCAFG00845028330	429	488	437	431
ENSCAFG00845004380	0	0	0	0
ENSCAFG00845016359	5	18	10	5
ENSCAFG00845016357	0	0	0	0
ENSCAFG00845016358	0	0	0	0
ENSCAFG00845016344	0	0	0	0
ENSCAFG00845016345	300	299	305	278
ENSCAFG00845016342	640	675	678	606
ENSCAFG00845016343	301	307	236	246
ENSCAFG00845016340	1	2	0	2

ENSCAFG00845016341	988	1025	1024	1185
ENSCAFG00845028329	0	0	0	0
ENSCAFG00845004359	0	0	1	5
ENSCAFG00845028326	1	1	1	4
ENSCAFG00845004358	0	0	0	0
ENSCAFG00845028325	0	0	0	0
ENSCAFG00845004357	0	0	0	0
ENSCAFG00845028328	0	0	0	0
ENSCAFG00845004356	57	49	42	35
ENSCAFG00845028327	2	3	9	6
ENSCAFG00845004355	0	0	0	0
ENSCAFG00845028322	424	401	552	659
ENSCAFG00845004354	1	1	4	4
ENSCAFG00845028321	0	0	0	0
ENSCAFG00845004353	5454	5264	5427	5458
ENSCAFG00845028324	947	905	841	858
ENSCAFG00845004352	35	40	70	57
ENSCAFG00845028323	1	1	4	0
ENSCAFG00845004351	16	12	19	10
ENSCAFG00845004350	0	0	0	0
ENSCAFG00845028320	8	7	2	6
ENSCAFG00845016348	6	4	9	8
ENSCAFG00845016349	79	84	82	67
ENSCAFG00845016346	1978	1917	1825	1891
ENSCAFG00845016347	0	0	0	0
ENSCAFG00845016333	0	0	0	0
ENSCAFG00845018996	0	0	0	0
ENSCAFG00845016334	0	0	0	0
ENSCAFG00845018995	722	658	745	688
ENSCAFG00845016331	960	932	912	1055
ENSCAFG00845018994	0	0	0	0
ENSCAFG00845016332	1799	1772	1602	1684
ENSCAFG00845018993	0	0	0	0
ENSCAFG00845018992	0	0	0	0
ENSCAFG00845016330	3	0	1	4
ENSCAFG00845018991	71	73	82	68
ENSCAFG00845018990	922	916	1013	974
ENSCAFG00845028319	0	0	0	0
ENSCAFG00845028318	0	0	0	0
ENSCAFG00845028315	195	160	172	148
ENSCAFG00845004369	2	4	0	2

ENSCAFG00845028314	1249	1205	1217	1089
ENSCAFG00845004368	4	4	1	1
ENSCAFG00845028317	0	0	0	0
ENSCAFG00845004367	0	0	0	0
ENSCAFG00845028316	0	0	0	0
ENSCAFG00845004366	16	15	10	8
ENSCAFG00845028311	728	696	741	694
ENSCAFG00845004365	1491	1475	1560	1525
ENSCAFG00845028310	1	0	0	1
ENSCAFG00845004364	0	0	0	0
ENSCAFG00845028313	123	98	119	114
ENSCAFG00845004363	112	94	89	133
ENSCAFG00845028312	4	0	1	1
ENSCAFG00845004362	0	0	0	0
ENSCAFG00845004361	103	71	67	56
ENSCAFG00845004360	51	32	35	35
ENSCAFG00845016339	3	3	2	1
ENSCAFG00845016337	1326	1254	1218	1234
ENSCAFG00845016338	0	0	0	1
ENSCAFG00845018999	109	104	92	96
ENSCAFG00845016335	0	0	0	0
ENSCAFG00845018998	1119	1176	993	1020
ENSCAFG00845016336	10	8	2	7
ENSCAFG00845018997	1686	1565	1422	1521
ENSCAFG00845016322	554	556	574	598
ENSCAFG00845018985	0	0	0	0
ENSCAFG00845016323	421	421	459	425
ENSCAFG00845018984	0	0	0	0
ENSCAFG00845016320	2	0	0	0
ENSCAFG00845018983	1	0	1	2
ENSCAFG00845016321	613	594	594	583
ENSCAFG00845018982	66	91	52	83
ENSCAFG00845018981	228	244	214	253
ENSCAFG00845018980	20	32	13	23
ENSCAFG00845028308	4	10	14	13
ENSCAFG00845028307	0	0	0	0
ENSCAFG00845004339	884	863	829	819
ENSCAFG00845004338	367	307	270	349
ENSCAFG00845028309	408	348	298	353
ENSCAFG00845004337	0	0	0	0
ENSCAFG00845028304	386	398	345	377

ENSCAFG00845004336	0	0	0	0
ENSCAFG00845028303	0	0	0	0
ENSCAFG00845004335	0	2	0	0
ENSCAFG00845028306	0	0	0	0
ENSCAFG00845004334	670	597	833	781
ENSCAFG00845028305	65	67	65	62
ENSCAFG00845004333	402	353	364	378
ENSCAFG00845004332	103	114	110	107
ENSCAFG00845004331	34	19	20	26
ENSCAFG00845028302	11	14	5	15
ENSCAFG00845004330	1165	1184	1303	1190
ENSCAFG00845028301	462	420	480	436
ENSCAFG00845016328	1890	1852	1765	1752
ENSCAFG00845016329	2	4	7	4
ENSCAFG00845016326	0	0	0	0
ENSCAFG00845018989	0	0	0	0
ENSCAFG00845016327	0	0	0	0
ENSCAFG00845018988	274	229	170	202
ENSCAFG00845016324	1099	1099	1123	1159
ENSCAFG00845018987	0	0	0	0
ENSCAFG00845016325	0	0	0	0
ENSCAFG00845018986	397	424	341	451
ENSCAFG00845016311	3	3	4	3
ENSCAFG00845016312	0	0	0	0
ENSCAFG00845018973	31	45	41	47
ENSCAFG00845018972	0	0	0	0
ENSCAFG00845016310	48	42	57	58
ENSCAFG00845018971	66	55	71	75
ENSCAFG00845018970	1228	1197	1210	1234
ENSCAFG00845006990	0	0	0	0
ENSCAFG00845004349	0	0	0	0
ENSCAFG00845006991	0	0	0	0
ENSCAFG00845004348	2362	2396	2229	2390
ENSCAFG00845006992	0	0	0	0
ENSCAFG00845004347	589	535	584	553
ENSCAFG00845006993	0	0	0	0
ENSCAFG00845004346	506	462	492	469
ENSCAFG00845006994	295	302	193	235
ENSCAFG00845004345	127	108	107	110
ENSCAFG00845006995	0	0	1	0
ENSCAFG00845004344	56	47	61	53

ENSCAFG00845006996	0	0	0	0
ENSCAFG00845004343	230	232	188	185
ENSCAFG00845006997	0	0	0	0
ENSCAFG00845004342	0	0	0	0
ENSCAFG00845006998	334	353	375	371
ENSCAFG00845004341	0	1	0	0
ENSCAFG00845006999	0	0	0	0
ENSCAFG00845004340	34	30	26	29
ENSCAFG00845016319	0	0	0	0
ENSCAFG00845016317	0	0	0	0
ENSCAFG00845016318	41	56	35	44
ENSCAFG00845018979	1119	975	1146	1078
ENSCAFG00845016315	0	0	0	0
ENSCAFG00845018978	26	9	26	18
ENSCAFG00845016316	0	4	1	0
ENSCAFG00845018977	1238	1212	1301	1310
ENSCAFG00845016313	69	72	82	62
ENSCAFG00845018976	132	117	118	126
ENSCAFG00845016314	1	0	0	0
ENSCAFG00845018975	2086	2061	1797	1884
ENSCAFG00845028399	161	136	127	154
ENSCAFG00845028398	1003	942	959	970
ENSCAFG00845028395	14	16	14	11
ENSCAFG00845028394	0	0	0	0
ENSCAFG00845028397	0	0	1	0
ENSCAFG00845028396	0	0	0	0
ENSCAFG00845028391	683	756	611	513
ENSCAFG00845028390	0	0	1	0
ENSCAFG00845028393	26	30	40	47
ENSCAFG00845028392	416	396	432	472
ENSCAFG00845028388	0	0	0	0
ENSCAFG00845028387	16	18	6	11
ENSCAFG00845028389	671	664	646	696
ENSCAFG00845028384	1728	1527	1799	1906
ENSCAFG00845028383	1225	1259	1107	1181
ENSCAFG00845028386	0	0	0	0
ENSCAFG00845028385	2	3	0	0
ENSCAFG00845028380	0	0	0	0
ENSCAFG00845028382	984	940	957	1003
ENSCAFG00845028381	0	2	0	1
ENSCAFG00845016399	0	0	0	0

ENSCAFG00845016397	0	0	0	0
ENSCAFG00845016398	0	0	0	0
ENSCAFG00845016395	659	638	716	685
ENSCAFG00845016396	335	262	283	287
ENSCAFG00845016393	322	259	326	270
ENSCAFG00845016394	0	1	0	0
ENSCAFG00845016391	17	19	15	4
ENSCAFG00845016392	96	80	59	61
ENSCAFG00845016390	0	0	0	0
ENSCAFG00845028377	716	650	642	641
ENSCAFG00845028376	0	2	0	0
ENSCAFG00845028379	0	0	4	0
ENSCAFG00845028378	36	21	28	36
ENSCAFG00845028373	1763	1753	1660	1736
ENSCAFG00845028372	4	0	0	8
ENSCAFG00845028375	810	811	947	954
ENSCAFG00845028374	0	0	0	0
ENSCAFG00845028371	0	0	0	0
ENSCAFG00845028370	0	0	0	0
ENSCAFG00845006900	0	0	0	0
ENSCAFG00845006901	497	414	424	433
ENSCAFG00845006902	0	0	0	0
ENSCAFG00845006903	235	260	214	208
ENSCAFG00845006904	474	427	547	525
ENSCAFG00845006905	119	87	85	107
ENSCAFG00845006906	0	1	2	3
ENSCAFG00845006907	0	0	0	0
ENSCAFG00845006908	26	10	13	30
ENSCAFG00845006909	149	102	142	145
ENSCAFG00845016300	0	0	0	0
ENSCAFG00845018963	0	0	0	0
ENSCAFG00845016301	3	17	25	12
ENSCAFG00845018962	4348	4291	4104	4086
ENSCAFG00845018961	434	442	379	392
ENSCAFG00845018960	0	0	0	0
ENSCAFG00845004319	595	528	516	575
ENSCAFG00845004318	891	795	918	871
ENSCAFG00845004317	29	16	18	16
ENSCAFG00845004316	3159	2909	2933	3007
ENSCAFG00845006980	0	0	0	0
ENSCAFG00845004315	545	539	564	556

ENSCAFG00845006981	0	0	0	0
ENSCAFG00845004314	216	205	199	255
ENSCAFG00845006982	0	0	0	0
ENSCAFG00845004313	0	0	0	0
ENSCAFG00845006983	0	0	0	0
ENSCAFG00845004312	0	0	0	0
ENSCAFG00845006984	7	3	0	0
ENSCAFG00845004311	0	0	0	0
ENSCAFG00845006985	45	49	44	61
ENSCAFG00845004310	0	0	0	0
ENSCAFG00845006986	243	196	206	251
ENSCAFG00845006987	359	365	297	351
ENSCAFG00845006988	86	86	87	94
ENSCAFG00845006989	0	0	0	0
ENSCAFG00845016308	0	0	0	0
ENSCAFG00845016309	0	0	0	0
ENSCAFG00845016306	0	0	0	0
ENSCAFG00845018969	1E+05	1E+05	63106	51697
ENSCAFG00845016307	630	602	606	630
ENSCAFG00845018968	0	0	0	0
ENSCAFG00845016304	399	427	429	457
ENSCAFG00845018967	0	0	0	0
ENSCAFG00845016305	0	0	0	0
ENSCAFG00845018966	0	0	0	0
ENSCAFG00845016302	65	62	80	85
ENSCAFG00845018965	744	730	659	633
ENSCAFG00845016303	716	708	702	762
ENSCAFG00845018964	0	0	0	0
ENSCAFG00845018952	434	473	520	537
ENSCAFG00845018951	0	0	0	0
ENSCAFG00845018950	1	3	0	0
ENSCAFG00845004329	0	0	0	0
ENSCAFG00845004328	0	1	2	0
ENSCAFG00845004327	333	296	325	307
ENSCAFG00845004326	0	0	0	0
ENSCAFG00845006970	0	0	0	0
ENSCAFG00845004325	110	105	62	76
ENSCAFG00845006971	0	0	0	0
ENSCAFG00845004324	0	0	1	1
ENSCAFG00845006972	654	601	492	469
ENSCAFG00845004323	0	0	0	0

ENSCAFG00845006973	727	727	688	754
ENSCAFG00845004322	100	93	107	75
ENSCAFG00845006974	0	0	0	0
ENSCAFG00845004321	160	144	163	162
ENSCAFG00845006975	2562	2534	2530	2602
ENSCAFG00845004320	0	1	0	0
ENSCAFG00845006976	79	67	93	89
ENSCAFG00845006977	0	0	0	0
ENSCAFG00845006978	0	0	0	0
ENSCAFG00845006979	0	1	0	0
ENSCAFG00845018959	2	2	7	3
ENSCAFG00845018958	4203	4357	4477	4292
ENSCAFG00845018957	5201	5188	5210	5274
ENSCAFG00845018956	25	28	22	22
ENSCAFG00845018955	19	26	15	25
ENSCAFG00845018954	0	0	0	0
ENSCAFG00845018953	0	0	0	0
ENSCAFG00845018941	2	4	1	3
ENSCAFG00845018940	890	932	1092	1047
ENSCAFG00845006960	0	1	0	0
ENSCAFG00845006961	0	1	0	0
ENSCAFG00845006962	4	1	1	3
ENSCAFG00845006963	0	0	0	0
ENSCAFG00845006964	147	95	152	139
ENSCAFG00845006965	0	0	0	0
ENSCAFG00845006966	0	0	0	0
ENSCAFG00845006967	1252	1234	1284	1423
ENSCAFG00845006968	428	423	454	386
ENSCAFG00845006969	0	0	0	0
ENSCAFG00845018949	0	0	0	0
ENSCAFG00845018948	590	528	605	512
ENSCAFG00845018947	282	232	327	267
ENSCAFG00845018945	632	543	667	694
ENSCAFG00845018944	0	0	0	0
ENSCAFG00845018943	477	510	561	522
ENSCAFG00845018942	453	470	457	414
ENSCAFG00845018930	0	0	0	0
ENSCAFG00845004309	49	43	23	40
ENSCAFG00845004308	370	401	446	430
ENSCAFG00845004307	10	3	1	10
ENSCAFG00845004306	0	2	1	0

ENSCAFG00845004305	732	692	609	652
ENSCAFG00845004304	0	0	0	0
ENSCAFG00845004303	316	300	272	259
ENSCAFG00845004302	3367	3263	3073	2991
ENSCAFG00845006950	2	0	0	3
ENSCAFG00845004301	209	177	192	216
ENSCAFG00845006951	2	0	0	1
ENSCAFG00845004300	0	0	2	0
ENSCAFG00845006952	0	0	0	0
ENSCAFG00845006953	0	1	1	0
ENSCAFG00845006954	0	0	0	0
ENSCAFG00845006955	250	267	244	303
ENSCAFG00845006956	1	1	1	4
ENSCAFG00845006957	414	379	389	441
ENSCAFG00845006958	57	53	67	83
ENSCAFG00845006959	804	786	720	764
ENSCAFG00845018939	1339	1368	1283	1287
ENSCAFG00845018938	0	0	0	0
ENSCAFG00845018937	2161	2154	2032	2173
ENSCAFG00845018936	1576	1706	1610	1672
ENSCAFG00845018935	459	462	452	433
ENSCAFG00845018934	160	162	230	222
ENSCAFG00845018933	0	2	4	8
ENSCAFG00845018932	1075	1051	1004	955
ENSCAFG00845006940	547	535	544	526
ENSCAFG00845006941	3	0	2	0
ENSCAFG00845006942	0	0	0	1
ENSCAFG00845006943	238	220	185	229
ENSCAFG00845006944	0	0	0	0
ENSCAFG00845006945	8	7	7	2
ENSCAFG00845006946	8870	8596	8392	8750
ENSCAFG00845006947	0	1	0	0
ENSCAFG00845018929	66	61	40	43
ENSCAFG00845006948	13	34	17	18
ENSCAFG00845018928	395	365	354	350
ENSCAFG00845006949	3881	3562	3663	3696
ENSCAFG00845018927	1	3	3	5
ENSCAFG00845018926	1093	965	1079	1050
ENSCAFG00845018925	1294	1250	1254	1386
ENSCAFG00845018924	4107	3867	4163	4125
ENSCAFG00845018923	0	0	0	0

ENSCAFG00845018922	107	106	128	130
ENSCAFG00845018921	149	126	173	184
ENSCAFG00845018920	0	0	0	0
ENSCAFG00845006930	0	0	0	0
ENSCAFG00845006931	57	53	52	38
ENSCAFG00845006932	65	46	57	70
ENSCAFG00845006933	397	367	398	405
ENSCAFG00845006934	378	349	334	337
ENSCAFG00845006935	523	514	434	467
ENSCAFG00845018919	0	0	0	0
ENSCAFG00845006936	0	3	1	3
ENSCAFG00845018918	0	0	0	0
ENSCAFG00845006937	1	0	0	0
ENSCAFG00845018917	1138	1015	1155	1163
ENSCAFG00845006938	1640	1604	1617	1611
ENSCAFG00845018916	515	537	494	473
ENSCAFG00845006939	5	4	4	2
ENSCAFG00845018915	0	0	0	0
ENSCAFG00845018914	0	0	0	0
ENSCAFG00845018913	388	361	286	334
ENSCAFG00845018912	1718	1534	1823	1772
ENSCAFG00845018911	3	0	2	3
ENSCAFG00845018910	0	0	0	3
ENSCAFG00845006920	1	0	0	0
ENSCAFG00845006921	0	0	0	0
ENSCAFG00845006922	31	33	32	45
ENSCAFG00845006923	86	50	70	55
ENSCAFG00845018909	0	0	0	0
ENSCAFG00845006924	0	0	0	0
ENSCAFG00845018908	238	232	205	207
ENSCAFG00845006925	530	492	493	462
ENSCAFG00845018907	0	0	0	0
ENSCAFG00845006926	0	0	0	0
ENSCAFG00845018906	29	24	16	20
ENSCAFG00845006927	0	0	0	0
ENSCAFG00845018905	242	206	208	184
ENSCAFG00845006928	7	2	4	2
ENSCAFG00845018904	170	141	159	169
ENSCAFG00845006929	353	307	359	370
ENSCAFG00845018903	8	16	8	17
ENSCAFG00845018902	0	0	0	0

ENSCAFG00845018901	0	0	0	0
ENSCAFG00845018900	26	25	19	16
ENSCAFG00845006910	1216	1189	1024	1117
ENSCAFG00845006911	756	851	748	702
ENSCAFG00845006912	0	0	0	0
ENSCAFG00845006913	0	0	0	0
ENSCAFG00845006914	0	0	0	0
ENSCAFG00845006915	5	1	2	5
ENSCAFG00845006916	0	0	0	0
ENSCAFG00845006917	0	2	0	0
ENSCAFG00845006918	0	0	0	0
ENSCAFG00845006919	0	3	1	0
ENSCAFG00845018204	195	146	184	167
ENSCAFG00845021856	0	0	1	0
ENSCAFG00845018203	258	245	233	221
ENSCAFG00845021857	1519	1358	1419	1537
ENSCAFG00845018202	0	1	1	0
ENSCAFG00845021854	0	0	0	0
ENSCAFG00845018201	0	0	0	0
ENSCAFG00845021855	2	8	4	3
ENSCAFG00845018200	0	0	0	0
ENSCAFG00845021858	2472	2422	2240	2356
ENSCAFG00845021859	444	425	415	438
ENSCAFG00845008881	356	282	331	373
ENSCAFG00845008880	780	740	791	726
ENSCAFG00845006220	0	0	0	0
ENSCAFG00845008883	1487	1431	1420	1471
ENSCAFG00845006221	29	32	14	12
ENSCAFG00845008882	3	5	1	9
ENSCAFG00845006222	80	66	82	71
ENSCAFG00845008885	0	0	0	0
ENSCAFG00845021852	0	0	0	0
ENSCAFG00845006223	2886	2896	2656	2593
ENSCAFG00845008884	992	926	935	955
ENSCAFG00845021853	7	1	12	5
ENSCAFG00845006224	0	0	0	0
ENSCAFG00845008887	3	3	2	2
ENSCAFG00845021850	0	0	0	0
ENSCAFG00845006225	9	2	5	6
ENSCAFG00845008886	0	0	3	0
ENSCAFG00845021851	403	464	359	449

ENSCAFG00845006226	3	1	1	3
ENSCAFG00845008889	1859	1734	1861	1822
ENSCAFG00845006227	0	0	0	0
ENSCAFG00845008888	16	19	17	20
ENSCAFG00845006228	30	24	26	16
ENSCAFG00845006229	0	0	0	0
ENSCAFG00845018209	10662	10252	10623	10673
ENSCAFG00845018208	0	0	0	0
ENSCAFG00845018207	0	0	0	0
ENSCAFG00845018206	0	0	0	0
ENSCAFG00845018205	0	2	0	3
ENSCAFG00845021845	122	87	125	104
ENSCAFG00845021846	516	506	548	575
ENSCAFG00845021843	7851	7324	7565	7850
ENSCAFG00845021844	2	0	1	0
ENSCAFG00845021849	0	0	0	4
ENSCAFG00845021847	587	577	676	642
ENSCAFG00845021848	0	0	0	0
ENSCAFG00845008870	233	196	218	197
ENSCAFG00845008872	1619	1530	1561	1562
ENSCAFG00845006210	14	20	21	13
ENSCAFG00845008871	2	1	0	2
ENSCAFG00845006211	0	0	0	0
ENSCAFG00845008874	0	0	0	0
ENSCAFG00845021841	642	561	731	731
ENSCAFG00845006212	0	0	0	0
ENSCAFG00845008873	15	14	6	8
ENSCAFG00845021842	0	0	0	0
ENSCAFG00845006213	377	343	340	350
ENSCAFG00845008876	146	144	179	164
ENSCAFG00845006214	0	0	0	0
ENSCAFG00845008875	767	695	777	797
ENSCAFG00845021840	0	0	0	2
ENSCAFG00845006215	24	12	22	27
ENSCAFG00845008878	477	452	506	541
ENSCAFG00845006216	2091	1948	1940	1969
ENSCAFG00845008877	0	0	0	0
ENSCAFG00845006217	27	23	32	25
ENSCAFG00845006218	567	555	553	598
ENSCAFG00845008879	1591	1492	1497	1453
ENSCAFG00845006219	1013	944	925	911

ENSCAFG00845021834	3	3	1	3
ENSCAFG00845021835	0	2	0	1
ENSCAFG00845021832	7	3	7	6
ENSCAFG00845021833	89	89	99	94
ENSCAFG00845021838	0	0	1	0
ENSCAFG00845021839	615	613	569	472
ENSCAFG00845021836	377	383	464	434
ENSCAFG00845021837	838	905	941	936
ENSCAFG00845008861	754	684	795	791
ENSCAFG00845008860	1477	1506	1405	1460
ENSCAFG00845006200	3	5	3	2
ENSCAFG00845008863	796	651	703	714
ENSCAFG00845021830	37	34	39	25
ENSCAFG00845006201	931	960	924	1001
ENSCAFG00845008862	382	384	364	348
ENSCAFG00845021831	0	0	0	0
ENSCAFG00845006202	0	0	0	0
ENSCAFG00845008865	2	1	5	3
ENSCAFG00845006203	0	0	0	0
ENSCAFG00845008864	2	3	8	8
ENSCAFG00845006204	0	0	0	2
ENSCAFG00845008867	0	0	0	0
ENSCAFG00845006205	1	0	3	3
ENSCAFG00845008866	0	0	0	0
ENSCAFG00845006206	0	0	0	0
ENSCAFG00845008869	0	0	0	0
ENSCAFG00845006207	117	115	105	124
ENSCAFG00845008868	0	0	0	0
ENSCAFG00845006208	18	20	15	21
ENSCAFG00845006209	1	1	2	4
ENSCAFG00845021829	1328	1337	1507	1647
ENSCAFG00845021823	0	1	0	0
ENSCAFG00845021824	1870	1805	1704	1689
ENSCAFG00845021821	3	1	0	2
ENSCAFG00845021822	0	0	0	0
ENSCAFG00845021827	146	132	138	142
ENSCAFG00845021828	919	948	868	865
ENSCAFG00845021825	6029	6089	5640	5426
ENSCAFG00845021826	6714	6109	3581	3726
ENSCAFG00845008850	0	2	2	4
ENSCAFG00845008852	1602	1574	1571	1671

ENSCAFG00845008851	240	251	143	160
ENSCAFG00845021820	2555	2464	2769	2685
ENSCAFG00845008854	0	0	0	0
ENSCAFG00845008853	1080	992	898	946
ENSCAFG00845008856	1148	1099	1220	1244
ENSCAFG00845008855	0	0	0	0
ENSCAFG00845008858	3977	3920	3384	3468
ENSCAFG00845008857	0	1	1	0
ENSCAFG00845008859	3372	3087	3019	3156
ENSCAFG00845021818	277	241	258	249
ENSCAFG00845021819	10847	10434	9977	10156
ENSCAFG00845021812	42	29	33	29
ENSCAFG00845021813	4	3	6	8
ENSCAFG00845021810	17	4	11	11
ENSCAFG00845021811	776	741	777	705
ENSCAFG00845021816	0	0	0	0
ENSCAFG00845021817	44	39	39	49
ENSCAFG00845021814	870	840	741	831
ENSCAFG00845021815	71	53	62	46
ENSCAFG00845008841	646	670	676	626
ENSCAFG00845008840	1227	1219	1437	1273
ENSCAFG00845008843	4341	4261	4350	4242
ENSCAFG00845008842	0	0	0	0
ENSCAFG00845008845	1853	1797	1587	1605
ENSCAFG00845008844	0	0	0	0
ENSCAFG00845008847	325	322	321	369
ENSCAFG00845008846	522	448	370	363
ENSCAFG00845008849	121	76	55	66
ENSCAFG00845008848	153	221	156	178
ENSCAFG00845021809	51	42	37	44
ENSCAFG00845021807	500	504	403	400
ENSCAFG00845021808	5	2	1	3
ENSCAFG00845021802	0	2	0	0
ENSCAFG00845021800	0	0	0	2
ENSCAFG00845021805	4	6	9	8
ENSCAFG00845021806	15	9	16	12
ENSCAFG00845021803	0	0	0	0
ENSCAFG00845021804	0	0	0	1
ENSCAFG00845008830	46	41	54	47
ENSCAFG00845008832	102	102	98	85
ENSCAFG00845008831	635	585	563	577

ENSCAFG00845008834	0	0	0	0
ENSCAFG00845008833	0	0	0	0
ENSCAFG00845008836	0	2	0	0
ENSCAFG00845008835	4	5	7	4
ENSCAFG00845008838	40	20	38	51
ENSCAFG00845008837	0	0	0	0
ENSCAFG00845008839	1213	1192	1225	1174
ENSCAFG00845008821	124	112	93	99
ENSCAFG00845008820	0	0	0	0
ENSCAFG00845008823	9	11	9	10
ENSCAFG00845008822	123	101	100	123
ENSCAFG00845008825	2	1	0	1
ENSCAFG00845008824	0	0	0	0
ENSCAFG00845008827	0	0	0	0
ENSCAFG00845008826	7	22	18	18
ENSCAFG00845008829	379	316	255	319
ENSCAFG00845008828	1081	1143	1046	1037
ENSCAFG00845008810	0	0	1	0
ENSCAFG00845008812	0	0	0	0
ENSCAFG00845008811	60587	58271	57442	57254
ENSCAFG00845008814	0	0	0	1
ENSCAFG00845008813	13	26	17	8
ENSCAFG00845008816	0	0	0	0
ENSCAFG00845008815	0	0	0	0
ENSCAFG00845008818	0	0	0	0
ENSCAFG00845008817	0	0	0	0
ENSCAFG00845008819	5	10	1	8
ENSCAFG00845018289	1616	1616	1551	1551
ENSCAFG00845018288	1541	1335	1370	1432
ENSCAFG00845018287	138	135	185	171
ENSCAFG00845018286	2	4	4	0
ENSCAFG00845018285	1	1	1	1
ENSCAFG00845018284	0	0	0	0
ENSCAFG00845018283	0	0	0	0
ENSCAFG00845018282	328	306	306	237
ENSCAFG00845018281	0	0	0	0
ENSCAFG00845018280	529	461	511	530
ENSCAFG00845018279	50	46	54	53
ENSCAFG00845006290	0	0	0	0
ENSCAFG00845018278	768	681	717	787
ENSCAFG00845006291	2902	2879	2735	2798

ENSCAFG00845018277	607	538	569	574
ENSCAFG00845006292	0	0	0	0
ENSCAFG00845018276	305	329	234	255
ENSCAFG00845006293	14	14	11	7
ENSCAFG00845018275	0	0	0	0
ENSCAFG00845006294	0	0	0	0
ENSCAFG00845018274	3767	3573	3668	3893
ENSCAFG00845006295	696	681	662	657
ENSCAFG00845018273	1	1	1	4
ENSCAFG00845006296	0	0	0	0
ENSCAFG00845018272	5	1	1	0
ENSCAFG00845006297	0	0	0	0
ENSCAFG00845018271	458	483	409	425
ENSCAFG00845006298	0	0	0	0
ENSCAFG00845018270	543	590	732	721
ENSCAFG00845006299	47	30	33	36
ENSCAFG00845018268	19906	19458	18154	18387
ENSCAFG00845018267	0	0	0	0
ENSCAFG00845006280	4	2	2	2
ENSCAFG00845018266	75	65	45	63
ENSCAFG00845006281	4093	3871	3866	4095
ENSCAFG00845018265	0	0	0	0
ENSCAFG00845006282	0	0	0	0
ENSCAFG00845018264	1365	1222	1230	1426
ENSCAFG00845006283	0	0	0	0
ENSCAFG00845018263	373	371	408	381
ENSCAFG00845006284	1	2	2	3
ENSCAFG00845018262	0	0	0	0
ENSCAFG00845006285	0	0	0	0
ENSCAFG00845018261	143	140	139	112
ENSCAFG00845006286	0	0	0	0
ENSCAFG00845018260	3	2	4	5
ENSCAFG00845006287	2	1	5	1
ENSCAFG00845006288	308	288	345	322
ENSCAFG00845006289	0	0	2	1
ENSCAFG00845018259	0	0	0	0
ENSCAFG00845018258	0	1	0	0
ENSCAFG00845018257	2	0	0	2
ENSCAFG00845018256	0	0	0	0
ENSCAFG00845018255	616	707	533	631
ENSCAFG00845006270	349	354	330	368

ENSCAFG00845018254	562	553	516	579
ENSCAFG00845006271	0	0	0	0
ENSCAFG00845018253	4	4	5	7
ENSCAFG00845006272	104	98	90	69
ENSCAFG00845018252	0	0	0	1
ENSCAFG00845006273	0	0	0	0
ENSCAFG00845018251	0	0	0	2
ENSCAFG00845006274	0	0	0	0
ENSCAFG00845018250	0	0	0	0
ENSCAFG00845006275	79	85	48	54
ENSCAFG00845006276	2	4	2	0
ENSCAFG00845006277	1165	1076	1020	962
ENSCAFG00845006278	0	0	0	0
ENSCAFG00845006279	0	0	0	0
ENSCAFG00845018248	3283	3097	3195	3302
ENSCAFG00845018247	673	601	734	731
ENSCAFG00845018246	3949	4033	3758	3990
ENSCAFG00845021898	16	26	9	8
ENSCAFG00845018245	0	0	0	0
ENSCAFG00845021899	2	0	3	0
ENSCAFG00845018244	10990	10674	11167	11132
ENSCAFG00845018243	1957	2039	2167	2209
ENSCAFG00845006260	115	119	107	113
ENSCAFG00845018242	0	0	0	1
ENSCAFG00845006261	0	0	0	0
ENSCAFG00845018241	0	0	0	0
ENSCAFG00845006262	0	0	0	0
ENSCAFG00845018240	1819	1657	1597	1475
ENSCAFG00845021892	387	385	370	377
ENSCAFG00845006263	0	0	0	0
ENSCAFG00845021893	210	246	261	233
ENSCAFG00845006264	0	0	0	0
ENSCAFG00845021890	8	8	6	14
ENSCAFG00845006265	723	679	741	798
ENSCAFG00845021891	5	4	4	3
ENSCAFG00845006266	579	555	389	435
ENSCAFG00845021896	375	377	382	357
ENSCAFG00845006267	1523	1533	1760	1772
ENSCAFG00845021897	416	476	433	418
ENSCAFG00845006268	0	0	0	0
ENSCAFG00845021894	0	0	0	0

ENSCAFG00845006269	0	0	0	0
ENSCAFG00845021895	4	11	2	1
ENSCAFG00845018249	0	0	0	0
ENSCAFG00845018237	305	290	280	319
ENSCAFG00845021889	1	0	2	0
ENSCAFG00845018236	0	0	0	0
ENSCAFG00845018235	341	347	374	301
ENSCAFG00845021887	168	164	259	229
ENSCAFG00845018234	1	0	0	0
ENSCAFG00845021888	0	0	0	0
ENSCAFG00845018233	1204	1209	1186	1215
ENSCAFG00845018232	0	0	0	0
ENSCAFG00845018231	0	0	0	0
ENSCAFG00845006250	0	0	0	0
ENSCAFG00845018230	0	0	0	0
ENSCAFG00845006251	742	710	757	820
ENSCAFG00845021881	5	7	7	6
ENSCAFG00845006252	104	72	80	70
ENSCAFG00845021882	5257	4907	5043	5312
ENSCAFG00845006253	27	53	26	36
ENSCAFG00845006254	0	0	0	0
ENSCAFG00845021880	213	203	246	247
ENSCAFG00845006255	790	783	748	796
ENSCAFG00845021885	367	364	371	330
ENSCAFG00845006256	0	5	1	0
ENSCAFG00845021886	932	874	941	914
ENSCAFG00845006257	0	0	0	0
ENSCAFG00845021883	3	4	0	1
ENSCAFG00845006258	152	163	150	162
ENSCAFG00845021884	231	143	127	146
ENSCAFG00845006259	25	26	26	17
ENSCAFG00845018238	283	343	289	271
ENSCAFG00845018226	317	305	361	408
ENSCAFG00845021878	0	0	0	0
ENSCAFG00845018225	3	0	1	1
ENSCAFG00845021879	0	0	1	0
ENSCAFG00845018224	58	62	49	64
ENSCAFG00845021876	306	334	343	351
ENSCAFG00845021877	80	99	91	97
ENSCAFG00845018222	0	0	0	0
ENSCAFG00845018221	224	174	238	228

ENSCAFG00845018220	0	0	0	0
ENSCAFG00845006240	33	26	39	26
ENSCAFG00845021870	197	183	183	218
ENSCAFG00845006241	0	0	0	0
ENSCAFG00845021871	3115	2860	3013	3055
ENSCAFG00845006242	0	0	0	0
ENSCAFG00845006243	0	0	0	0
ENSCAFG00845006244	0	0	0	0
ENSCAFG00845021874	0	0	0	0
ENSCAFG00845006245	1	1	1	1
ENSCAFG00845021875	0	0	0	0
ENSCAFG00845006246	1911	1890	1848	1921
ENSCAFG00845021872	557	549	553	570
ENSCAFG00845006247	6	5	8	7
ENSCAFG00845021873	0	0	0	0
ENSCAFG00845006248	0	0	0	0
ENSCAFG00845006249	1205	1147	1218	1239
ENSCAFG00845018229	0	0	0	0
ENSCAFG00845018228	0	0	0	0
ENSCAFG00845018227	2324	2236	2758	2858
ENSCAFG00845018215	1167	1101	945	965
ENSCAFG00845021867	9	3	1	7
ENSCAFG00845018214	0	0	0	0
ENSCAFG00845021868	0	0	0	0
ENSCAFG00845018213	62	64	60	62
ENSCAFG00845021865	95	92	102	93
ENSCAFG00845018212	0	0	0	0
ENSCAFG00845021866	0	0	0	0
ENSCAFG00845018211	336	345	367	391
ENSCAFG00845018210	0	0	0	0
ENSCAFG00845008890	0	0	0	0
ENSCAFG00845021869	403	347	405	397
ENSCAFG00845008892	113	164	134	154
ENSCAFG00845006230	448	401	440	491
ENSCAFG00845008891	6	4	8	1
ENSCAFG00845021860	876	845	685	624
ENSCAFG00845006231	7	10	5	6
ENSCAFG00845008894	0	0	0	1
ENSCAFG00845006232	552	570	569	583
ENSCAFG00845008893	20918	20414	20337	20807
ENSCAFG00845006233	0	0	0	0

ENSCAFG00845008896	15	11	23	13
ENSCAFG00845021863	751	710	667	637
ENSCAFG00845006234	1	2	1	0
ENSCAFG00845008895	473	527	434	437
ENSCAFG00845021864	5644	5484	5342	5585
ENSCAFG00845006235	0	0	0	0
ENSCAFG00845008898	98	82	78	113
ENSCAFG00845021861	0	0	0	0
ENSCAFG00845006236	393	385	413	415
ENSCAFG00845008897	423	397	404	351
ENSCAFG00845021862	3	3	0	1
ENSCAFG00845006237	10	7	7	8
ENSCAFG00845006238	2	7	4	2
ENSCAFG00845008899	2	9	3	3
ENSCAFG00845006239	49	39	34	33
ENSCAFG00845018219	0	0	1	4
ENSCAFG00845018218	5	1	0	2
ENSCAFG00845018217	0	0	0	0
ENSCAFG00845018216	0	0	0	0
ENSCAFG00845018199	0	0	0	0
ENSCAFG00845018198	0	0	0	0
ENSCAFG00845018197	68	55	58	64
ENSCAFG00845018196	0	0	0	0
ENSCAFG00845018195	4	2	0	0
ENSCAFG00845018194	273	250	206	201
ENSCAFG00845018193	470	479	538	552
ENSCAFG00845018192	0	0	0	0
ENSCAFG00845018191	8	8	0	0
ENSCAFG00845018190	1	0	0	0
ENSCAFG00845018189	1	5	5	2
ENSCAFG00845018188	0	0	0	0
ENSCAFG00845018187	160	170	167	155
ENSCAFG00845018186	0	0	0	0
ENSCAFG00845018185	387	347	346	384
ENSCAFG00845018184	9	5	3	3
ENSCAFG00845018183	0	0	0	0
ENSCAFG00845018182	1431	1377	1216	1307
ENSCAFG00845018181	27	16	22	19
ENSCAFG00845018180	35	20	27	30
ENSCAFG00845006190	0	0	0	0
ENSCAFG00845006191	0	0	0	0

ENSCAFG00845018179	1657	1629	1388	1453
ENSCAFG00845006192	0	0	0	0
ENSCAFG00845018178	0	0	0	0
ENSCAFG00845006193	144	173	176	135
ENSCAFG00845018177	3	2	2	1
ENSCAFG00845006194	91	136	90	111
ENSCAFG00845018176	17	7	25	6
ENSCAFG00845006195	1	8	4	12
ENSCAFG00845018175	0	0	0	0
ENSCAFG00845006196	626	593	589	664
ENSCAFG00845018174	49	65	59	65
ENSCAFG00845006197	665	607	609	606
ENSCAFG00845018173	5	2	5	5
ENSCAFG00845006198	0	1	0	2
ENSCAFG00845018172	0	0	0	0
ENSCAFG00845006199	0	0	0	0
ENSCAFG00845018171	0	2	0	0
ENSCAFG00845018170	0	0	0	0
ENSCAFG00845008801	0	0	0	0
ENSCAFG00845008800	0	0	0	0
ENSCAFG00845008803	779	762	823	791
ENSCAFG00845008802	1150	1107	1226	1202
ENSCAFG00845008805	1	0	0	0
ENSCAFG00845008804	0	0	0	0
ENSCAFG00845008807	5	2	6	4
ENSCAFG00845008806	0	0	0	0
ENSCAFG00845008809	15	16	6	21
ENSCAFG00845008808	0	0	0	0
ENSCAFG00845018325	239	246	253	217
ENSCAFG00845021977	0	0	0	0
ENSCAFG00845018324	0	0	0	0
ENSCAFG00845021978	918	835	752	818
ENSCAFG00845018323	1821	1585	1876	1843
ENSCAFG00845021975	578	505	433	482
ENSCAFG00845018322	0	0	0	0
ENSCAFG00845018321	621	632	633	616
ENSCAFG00845018320	0	0	0	1
ENSCAFG00845021979	251	231	179	197
ENSCAFG00845006340	0	0	0	0
ENSCAFG00845021970	1	2	1	1
ENSCAFG00845006341	0	0	0	0

ENSCAFG00845006342	2725	2778	2551	2572
ENSCAFG00845006343	1467	1437	1398	1468
ENSCAFG00845021973	16	9	19	15
ENSCAFG00845006344	0	0	0	0
ENSCAFG00845021974	755	692	667	702
ENSCAFG00845006345	1	7	4	3
ENSCAFG00845021971	0	0	0	0
ENSCAFG00845006346	0	0	0	0
ENSCAFG00845021972	0	2	4	3
ENSCAFG00845006347	0	0	0	0
ENSCAFG00845006348	0	0	0	0
ENSCAFG00845006349	281	312	265	279
ENSCAFG00845018329	41	48	54	44
ENSCAFG00845018328	0	0	0	0
ENSCAFG00845018327	807	696	879	870
ENSCAFG00845018326	2054	1848	1862	1960
ENSCAFG00845018314	1	0	3	2
ENSCAFG00845021966	416	416	486	414
ENSCAFG00845018313	457	454	547	487
ENSCAFG00845021967	8441	7915	8103	8543
ENSCAFG00845018312	0	0	0	0
ENSCAFG00845021964	392	386	381	416
ENSCAFG00845018311	0	0	0	0
ENSCAFG00845021965	1	5	1	1
ENSCAFG00845021968	0	0	0	0
ENSCAFG00845021969	0	0	0	0
ENSCAFG00845008991	0	0	0	0
ENSCAFG00845008990	0	0	0	0
ENSCAFG00845006330	0	0	0	0
ENSCAFG00845008993	340	321	404	366
ENSCAFG00845006331	6721	6283	6673	6944
ENSCAFG00845008992	0	0	0	0
ENSCAFG00845006332	0	0	0	0
ENSCAFG00845008995	0	0	0	0
ENSCAFG00845021962	367	308	360	377
ENSCAFG00845006333	0	0	0	0
ENSCAFG00845008994	492	401	352	393
ENSCAFG00845021963	156	119	108	127
ENSCAFG00845006334	0	0	0	0
ENSCAFG00845008997	1	0	0	0
ENSCAFG00845021960	1	4	5	6

ENSCAFG00845006335	128	157	158	150
ENSCAFG00845008996	0	0	0	0
ENSCAFG00845021961	6	1	1	4
ENSCAFG00845006336	0	0	0	0
ENSCAFG00845008999	11	9	10	10
ENSCAFG00845006337	0	0	0	0
ENSCAFG00845008998	1	2	0	0
ENSCAFG00845006338	0	0	0	0
ENSCAFG00845006339	234	223	127	149
ENSCAFG00845018319	486	440	528	490
ENSCAFG00845018318	72	85	65	89
ENSCAFG00845018317	0	0	0	0
ENSCAFG00845018316	1161	1218	1167	1192
ENSCAFG00845018315	179	152	204	144
ENSCAFG00845018303	1	0	0	0
ENSCAFG00845021955	835	840	870	798
ENSCAFG00845018302	0	0	0	0
ENSCAFG00845021956	0	0	0	0
ENSCAFG00845018301	0	0	0	0
ENSCAFG00845021953	0	0	0	0
ENSCAFG00845018300	71	66	71	81
ENSCAFG00845021954	0	0	0	0
ENSCAFG00845021959	0	0	0	0
ENSCAFG00845021957	0	0	0	0
ENSCAFG00845021958	0	0	0	0
ENSCAFG00845008980	0	0	2	0
ENSCAFG00845008982	468	404	447	438
ENSCAFG00845006320	329	322	295	352
ENSCAFG00845008981	216	227	205	254
ENSCAFG00845006321	0	0	0	0
ENSCAFG00845008984	0	0	0	0
ENSCAFG00845021951	0	0	0	0
ENSCAFG00845006322	0	0	0	0
ENSCAFG00845008983	156	144	163	132
ENSCAFG00845021952	928	923	1025	1074
ENSCAFG00845006323	1	4	5	2
ENSCAFG00845008986	877	793	904	960
ENSCAFG00845006324	0	0	0	0
ENSCAFG00845008985	405	408	407	405
ENSCAFG00845021950	2312	2124	2231	2188
ENSCAFG00845006325	400	386	352	366

ENSCAFG00845008988	0	4	0	2
ENSCAFG00845006326	0	0	0	0
ENSCAFG00845008987	2300	2271	2122	2054
ENSCAFG00845006327	7	11	12	7
ENSCAFG00845006328	4	0	5	0
ENSCAFG00845008989	1	0	0	1
ENSCAFG00845006329	0	0	0	0
ENSCAFG00845018309	0	1	0	0
ENSCAFG00845018308	0	0	0	0
ENSCAFG00845018307	1	0	0	0
ENSCAFG00845018306	0	0	0	0
ENSCAFG00845018305	1	1	4	0
ENSCAFG00845018304	0	2	0	0
ENSCAFG00845021944	175	167	115	112
ENSCAFG00845021945	90	154	115	118
ENSCAFG00845021942	32	39	36	27
ENSCAFG00845021943	13	14	26	25
ENSCAFG00845021948	55	36	55	35
ENSCAFG00845021949	1	1	2	2
ENSCAFG00845021946	3	7	11	6
ENSCAFG00845021947	12	6	6	5
ENSCAFG00845008971	1	1	0	0
ENSCAFG00845008970	1665	1620	1704	1750
ENSCAFG00845006310	0	0	0	0
ENSCAFG00845008973	2	1	2	0
ENSCAFG00845021940	0	0	1	5
ENSCAFG00845006311	345	341	292	273
ENSCAFG00845008972	0	0	0	0
ENSCAFG00845021941	0	0	0	0
ENSCAFG00845006312	369	377	363	365
ENSCAFG00845008975	0	0	0	0
ENSCAFG00845006313	0	0	0	0
ENSCAFG00845008974	0	0	0	0
ENSCAFG00845006314	0	0	0	0
ENSCAFG00845008977	473	510	485	480
ENSCAFG00845006315	8694	8429	7898	7853
ENSCAFG00845008976	147	139	121	116
ENSCAFG00845006316	2484	2445	2237	2257
ENSCAFG00845008979	12	8	18	9
ENSCAFG00845006317	1372	1346	1258	1267
ENSCAFG00845008978	0	3	3	2

ENSCAFG00845006318	8	8	5	15
ENSCAFG00845006319	542	527	534	508
ENSCAFG00845021939	0	1	0	0
ENSCAFG00845021933	0	0	0	4
ENSCAFG00845021934	7	8	1	7
ENSCAFG00845021931	0	0	0	0
ENSCAFG00845021932	339	334	277	263
ENSCAFG00845021937	0	4	0	3
ENSCAFG00845021938	259	173	192	208
ENSCAFG00845021936	1432	1375	1410	1446
ENSCAFG00845008960	5257	5098	4857	5045
ENSCAFG00845008962	1	0	0	0
ENSCAFG00845006300	712	783	673	726
ENSCAFG00845008961	232	203	207	212
ENSCAFG00845021930	2726	2421	2415	2449
ENSCAFG00845006301	3849	3648	3884	4053
ENSCAFG00845008964	0	0	0	0
ENSCAFG00845006302	3	5	3	2
ENSCAFG00845008963	0	0	0	0
ENSCAFG00845006303	8	12	3	10
ENSCAFG00845008966	0	0	0	0
ENSCAFG00845006304	0	0	0	0
ENSCAFG00845008965	5	3	4	3
ENSCAFG00845006305	3	2	2	1
ENSCAFG00845008968	2	1	0	1
ENSCAFG00845006306	0	0	0	0
ENSCAFG00845008967	0	0	0	0
ENSCAFG00845006307	0	0	0	0
ENSCAFG00845006308	390	365	348	360
ENSCAFG00845008969	0	0	1	1
ENSCAFG00845006309	138	171	133	148
ENSCAFG00845021928	36	25	31	29
ENSCAFG00845021929	0	0	0	0
ENSCAFG00845021922	0	0	3	1
ENSCAFG00845021923	1638	1548	1439	1421
ENSCAFG00845021920	1	3	2	0
ENSCAFG00845021921	12	8	1	3
ENSCAFG00845021926	9	14	13	10
ENSCAFG00845021927	0	0	0	0
ENSCAFG00845021924	0	1	0	0
ENSCAFG00845021925	41	35	33	35

ENSCAFG00845008951	3	0	3	0
ENSCAFG00845008950	2109	1931	2344	2483
ENSCAFG00845008953	0	0	1	1
ENSCAFG00845008952	0	0	0	0
ENSCAFG00845008955	50	45	61	43
ENSCAFG00845008954	6	7	19	10
ENSCAFG00845008957	145	134	123	132
ENSCAFG00845008956	3	2	3	0
ENSCAFG00845008959	5	0	2	0
ENSCAFG00845008958	0	0	0	0
ENSCAFG00845021919	0	3	4	2
ENSCAFG00845021917	28	27	41	39
ENSCAFG00845021918	53	52	41	34
ENSCAFG00845021911	0	0	0	0
ENSCAFG00845021912	808	780	749	848
ENSCAFG00845021910	1	0	0	1
ENSCAFG00845021915	12	18	12	15
ENSCAFG00845021916	298	295	299	328
ENSCAFG00845021913	0	0	0	0
ENSCAFG00845021914	322	282	255	267
ENSCAFG00845008940	767	761	627	730
ENSCAFG00845008942	0	0	0	0
ENSCAFG00845008941	1	3	0	0
ENSCAFG00845008944	0	0	0	0
ENSCAFG00845008943	0	3	1	0
ENSCAFG00845008946	0	0	0	0
ENSCAFG00845008945	124	126	117	142
ENSCAFG00845008948	1	1	2	3
ENSCAFG00845008947	0	0	0	0
ENSCAFG00845008949	1133	1092	1105	1025
ENSCAFG00845021908	1	2	0	0
ENSCAFG00845021909	4661	4576	4469	4559
ENSCAFG00845021906	1577	1410	1632	1611
ENSCAFG00845021907	0	0	0	0
ENSCAFG00845021900	487	404	525	496
ENSCAFG00845021901	889	926	913	929
ENSCAFG00845021904	592	504	603	649
ENSCAFG00845021905	0	0	0	0
ENSCAFG00845021902	12	1	6	1
ENSCAFG00845021903	655	685	639	580
ENSCAFG00845008931	2717	2659	2551	2611

ENSCAFG00845008930	0	0	0	0
ENSCAFG00845008933	956	900	648	684
ENSCAFG00845008932	0	0	0	0
ENSCAFG00845008935	0	3	0	0
ENSCAFG00845008934	12	13	11	11
ENSCAFG00845008937	1	0	1	0
ENSCAFG00845008936	0	0	0	0
ENSCAFG00845008939	880	857	797	873
ENSCAFG00845008938	191	240	242	242
ENSCAFG00845018399	0	0	0	0
ENSCAFG00845018398	1295	1143	1284	1245
ENSCAFG00845018397	2596	2467	2502	2633
ENSCAFG00845018396	0	0	0	0
ENSCAFG00845018395	0	0	0	0
ENSCAFG00845018394	516	494	518	561
ENSCAFG00845018393	152	139	214	221
ENSCAFG00845018392	96	67	96	69
ENSCAFG00845018391	355	343	364	350
ENSCAFG00845018390	0	0	0	0
ENSCAFG00845018389	1908	1861	1922	1882
ENSCAFG00845018387	889	843	910	929
ENSCAFG00845018386	421	414	507	488
ENSCAFG00845018385	206	223	317	280
ENSCAFG00845018384	980	993	927	938
ENSCAFG00845018383	2534	2482	2281	2208
ENSCAFG00845018382	8	5	17	8
ENSCAFG00845018381	48	42	36	44
ENSCAFG00845018380	1743	1643	1894	1897
ENSCAFG00845018379	428	429	429	392
ENSCAFG00845018378	0	0	0	0
ENSCAFG00845018377	0	2	1	1
ENSCAFG00845006390	0	2	1	2
ENSCAFG00845018376	8	3	4	2
ENSCAFG00845006391	542	538	609	622
ENSCAFG00845018375	111	127	119	109
ENSCAFG00845006392	887	830	798	750
ENSCAFG00845018374	0	0	0	0
ENSCAFG00845006393	2754	2612	2700	2880
ENSCAFG00845018373	5	1	5	0
ENSCAFG00845006394	959	787	820	801
ENSCAFG00845018372	1	0	0	1

ENSCAFG00845006395	4	3	1	1
ENSCAFG00845018371	1	0	0	0
ENSCAFG00845006396	0	0	0	0
ENSCAFG00845018370	0	0	0	0
ENSCAFG00845006397	0	0	0	0
ENSCAFG00845006398	10	10	28	15
ENSCAFG00845006399	0	0	0	0
ENSCAFG00845018369	2	4	9	6
ENSCAFG00845018368	1	2	1	1
ENSCAFG00845018367	513	477	459	537
ENSCAFG00845018366	4394	4069	3831	3975
ENSCAFG00845018365	3	10	15	19
ENSCAFG00845006380	0	2	6	6
ENSCAFG00845018364	0	0	0	0
ENSCAFG00845006381	0	0	0	0
ENSCAFG00845018363	651	697	701	684
ENSCAFG00845006382	3296	3210	3264	3235
ENSCAFG00845018362	0	0	0	0
ENSCAFG00845006383	157	185	166	204
ENSCAFG00845018361	151	169	194	188
ENSCAFG00845006384	295	293	378	382
ENSCAFG00845018360	1406	1252	1406	1433
ENSCAFG00845006385	0	0	0	0
ENSCAFG00845006386	0	5	2	5
ENSCAFG00845006387	63	40	42	53
ENSCAFG00845006388	586	576	681	637
ENSCAFG00845006389	0	0	0	0
ENSCAFG00845018358	2	1	1	0
ENSCAFG00845018357	0	0	0	0
ENSCAFG00845018356	719	676	514	543
ENSCAFG00845018355	20	36	27	45
ENSCAFG00845018354	185	187	223	193
ENSCAFG00845018353	954	917	786	832
ENSCAFG00845006370	0	0	0	0
ENSCAFG00845018352	0	0	0	0
ENSCAFG00845006371	1890	1749	1871	2040
ENSCAFG00845018351	0	0	0	0
ENSCAFG00845006372	594	572	470	563
ENSCAFG00845018350	10	0	0	0
ENSCAFG00845006373	50	47	40	29
ENSCAFG00845006374	44	48	41	23

ENSCAFG00845006375	272	306	308	335
ENSCAFG00845006376	44	71	98	80
ENSCAFG00845006377	0	0	0	0
ENSCAFG00845006378	8	5	12	7
ENSCAFG00845006379	0	0	0	1
ENSCAFG00845018359	5	3	1	4
ENSCAFG00845018347	0	0	0	0
ENSCAFG00845021999	2	1	5	1
ENSCAFG00845018346	0	0	0	0
ENSCAFG00845018345	0	0	0	0
ENSCAFG00845021997	0	0	0	0
ENSCAFG00845021998	1	0	2	2
ENSCAFG00845018343	0	0	0	0
ENSCAFG00845018342	39	38	64	51
ENSCAFG00845018341	10	11	22	15
ENSCAFG00845006360	740	702	547	646
ENSCAFG00845018340	1	3	3	1
ENSCAFG00845006361	0	0	0	0
ENSCAFG00845021991	0	0	0	0
ENSCAFG00845006362	952	968	821	901
ENSCAFG00845021992	0	0	0	0
ENSCAFG00845006363	147	117	135	111
ENSCAFG00845006364	875	912	945	960
ENSCAFG00845021990	399	409	360	405
ENSCAFG00845006365	0	0	0	0
ENSCAFG00845021995	0	0	0	0
ENSCAFG00845006366	0	0	0	0
ENSCAFG00845021996	645	566	557	603
ENSCAFG00845006367	0	0	0	0
ENSCAFG00845021993	2582	2472	2491	2558
ENSCAFG00845006368	3	3	1	1
ENSCAFG00845021994	1947	1817	1966	1729
ENSCAFG00845006369	2	1	2	2
ENSCAFG00845018349	0	0	0	0
ENSCAFG00845018348	0	0	0	0
ENSCAFG00845018336	0	2	4	7
ENSCAFG00845021988	0	0	0	0
ENSCAFG00845018335	2	6	7	5
ENSCAFG00845021989	0	0	0	0
ENSCAFG00845018334	0	0	0	0
ENSCAFG00845021986	1175	993	1052	955

ENSCAFG00845018333	1072	1130	1096	1165
ENSCAFG00845021987	0	5	0	0
ENSCAFG00845018332	0	0	0	0
ENSCAFG00845018331	0	1	0	0
ENSCAFG00845018330	2	0	0	0
ENSCAFG00845006350	0	0	0	0
ENSCAFG00845021980	0	0	0	0
ENSCAFG00845006351	0	0	0	0
ENSCAFG00845021981	448	451	377	409
ENSCAFG00845006352	300	304	327	306
ENSCAFG00845006353	101	94	88	103
ENSCAFG00845006354	154	151	138	153
ENSCAFG00845021984	0	0	0	0
ENSCAFG00845006355	0	0	0	0
ENSCAFG00845021985	7229	6987	8621	8863
ENSCAFG00845006356	0	0	0	0
ENSCAFG00845021982	0	0	0	0
ENSCAFG00845006357	0	0	0	0
ENSCAFG00845021983	17	3	13	9
ENSCAFG00845006358	0	0	0	0
ENSCAFG00845006359	0	0	0	0
ENSCAFG00845018339	776	792	861	821
ENSCAFG00845018338	758	756	739	771
ENSCAFG00845018337	0	0	0	0
ENSCAFG00845018299	2150	2140	2071	1901
ENSCAFG00845018298	176	138	124	133
ENSCAFG00845018297	2249	2188	2010	2040
ENSCAFG00845018296	0	0	0	0
ENSCAFG00845018295	10	5	13	11
ENSCAFG00845018294	573	623	576	709
ENSCAFG00845018293	0	0	0	0
ENSCAFG00845018292	320	356	405	354
ENSCAFG00845018291	0	0	0	0
ENSCAFG00845018290	925	825	879	842
ENSCAFG00845008920	650	673	656	608
ENSCAFG00845008922	155	143	129	144
ENSCAFG00845008921	0	0	0	0
ENSCAFG00845008924	119	114	90	103
ENSCAFG00845008923	645	690	617	638
ENSCAFG00845008926	0	0	0	0
ENSCAFG00845008925	0	0	0	0

ENSCAFG00845008928	15	29	23	17
ENSCAFG00845008927	1726	1581	1636	1706
ENSCAFG00845008929	1	2	1	2
ENSCAFG00845008911	4	0	0	4
ENSCAFG00845008910	36	35	29	31
ENSCAFG00845008913	13460	12973	12447	13417
ENSCAFG00845008912	3	1	0	0
ENSCAFG00845008915	602	565	608	612
ENSCAFG00845008914	81	79	67	74
ENSCAFG00845008917	2463	2373	2323	2218
ENSCAFG00845008916	1113	1119	1072	1157
ENSCAFG00845008919	0	0	0	0
ENSCAFG00845008918	2027	1967	1954	1827
ENSCAFG00845008900	6	7	2	0
ENSCAFG00845008902	0	0	0	0
ENSCAFG00845008901	1	0	0	3
ENSCAFG00845008904	0	0	0	0
ENSCAFG00845008903	1	2	1	2
ENSCAFG00845008906	2762	2599	2732	2883
ENSCAFG00845008905	205	196	176	184
ENSCAFG00845008908	2	0	0	4
ENSCAFG00845008907	5	3	1	2
ENSCAFG00845008909	471	454	368	339
ENSCAFG00845018446	6	11	8	10
ENSCAFG00845018445	0	0	0	0
ENSCAFG00845018444	35	27	10	17
ENSCAFG00845018443	729	758	802	714
ENSCAFG00845018442	1	1	2	5
ENSCAFG00845018441	7	5	1	0
ENSCAFG00845018440	2	0	2	2
ENSCAFG00845006460	6	1	2	7
ENSCAFG00845006461	274	283	328	289
ENSCAFG00845006462	7292	6664	6949	7045
ENSCAFG00845006463	714	689	716	673
ENSCAFG00845006464	344	319	351	325
ENSCAFG00845006465	0	0	0	0
ENSCAFG00845006466	0	2	8	5
ENSCAFG00845006467	4	0	0	0
ENSCAFG00845006468	260	265	263	275
ENSCAFG00845006469	1595	1328	1422	1272
ENSCAFG00845018449	10	13	45	43

ENSCAFG00845018448	135	152	184	156
ENSCAFG00845018447	1462	1508	1341	1513
ENSCAFG00845018435	44	38	30	40
ENSCAFG00845018434	368	397	385	332
ENSCAFG00845018433	0	0	0	0
ENSCAFG00845018432	0	0	0	0
ENSCAFG00845018431	0	0	0	0
ENSCAFG00845018430	57	46	63	47
ENSCAFG00845006450	0	0	0	0
ENSCAFG00845006451	0	0	0	0
ENSCAFG00845006452	413	377	300	299
ENSCAFG00845006453	471	477	425	414
ENSCAFG00845006454	0	0	0	0
ENSCAFG00845006455	0	0	0	0
ENSCAFG00845006456	0	0	0	0
ENSCAFG00845006457	8	5	4	14
ENSCAFG00845006458	0	0	0	0
ENSCAFG00845006459	0	0	0	0
ENSCAFG00845018439	0	0	0	0
ENSCAFG00845018438	0	0	0	0
ENSCAFG00845018437	0	0	0	0
ENSCAFG00845018436	0	4	1	0
ENSCAFG00845018424	400	370	482	433
ENSCAFG00845018423	975	1023	1032	963
ENSCAFG00845018422	274	267	224	271
ENSCAFG00845018421	0	0	0	0
ENSCAFG00845018420	3	4	1	7
ENSCAFG00845006440	270	208	243	221
ENSCAFG00845006441	0	1	1	0
ENSCAFG00845006442	3	5	1	5
ENSCAFG00845006443	33	32	33	32
ENSCAFG00845006444	0	0	0	0
ENSCAFG00845006445	3	1	3	2
ENSCAFG00845006446	2	2	1	4
ENSCAFG00845006447	9	3	5	7
ENSCAFG00845006448	334	347	317	358
ENSCAFG00845006449	637	622	628	541
ENSCAFG00845018429	3	0	1	0
ENSCAFG00845018427	0	0	0	0
ENSCAFG00845018426	1045	1107	1094	1005
ENSCAFG00845018425	0	0	0	0

ENSCAFG00845018413	0	0	0	0
ENSCAFG00845018412	716	741	725	730
ENSCAFG00845018411	30	16	12	16
ENSCAFG00845018410	32	23	11	43
ENSCAFG00845006430	0	0	0	0
ENSCAFG00845006431	0	0	0	0
ENSCAFG00845006432	37	38	33	36
ENSCAFG00845006433	1018	1027	998	1064
ENSCAFG00845006434	15	9	7	11
ENSCAFG00845006435	717	766	778	784
ENSCAFG00845006436	925	968	1109	1159
ENSCAFG00845006437	115	122	146	129
ENSCAFG00845006438	0	0	0	0
ENSCAFG00845006439	578	562	588	645
ENSCAFG00845018419	1430	1287	1605	1535
ENSCAFG00845018418	1288	1235	1313	1257
ENSCAFG00845018417	7	3	14	6
ENSCAFG00845018416	0	0	0	0
ENSCAFG00845018415	1954	2053	1712	1785
ENSCAFG00845018414	186	183	188	211
ENSCAFG00845018402	1	0	2	0
ENSCAFG00845018401	5	4	3	3
ENSCAFG00845018400	2	2	1	3
ENSCAFG00845006420	39	42	40	49
ENSCAFG00845006421	0	0	0	0
ENSCAFG00845006422	0	0	0	0
ENSCAFG00845006423	100	79	69	90
ENSCAFG00845006424	122	127	112	133
ENSCAFG00845006425	0	0	0	0
ENSCAFG00845006426	1	0	0	0
ENSCAFG00845006427	5046	4876	5291	5309
ENSCAFG00845006428	77	72	129	97
ENSCAFG00845006429	117	149	132	131
ENSCAFG00845018409	1	2	0	0
ENSCAFG00845018408	3	2	4	0
ENSCAFG00845018407	0	0	0	0
ENSCAFG00845018405	0	0	0	0
ENSCAFG00845018404	0	1	0	1
ENSCAFG00845018403	0	0	0	0
ENSCAFG00845006410	0	0	0	0
ENSCAFG00845006411	0	2	1	0

ENSCAFG00845006412	433	430	393	411
ENSCAFG00845006413	0	0	0	0
ENSCAFG00845006414	0	0	0	0
ENSCAFG00845006415	2	1	0	1
ENSCAFG00845006416	5	4	7	3
ENSCAFG00845006417	135	87	126	134
ENSCAFG00845006418	965	1049	904	954
ENSCAFG00845006419	4449	4234	4013	4361
ENSCAFG00845006400	33	33	29	25
ENSCAFG00845006401	0	0	0	0
ENSCAFG00845006402	182	182	155	161
ENSCAFG00845006403	582	552	627	643
ENSCAFG00845006404	0	0	0	0
ENSCAFG00845006405	1025	936	855	875
ENSCAFG00845006406	949	861	915	907
ENSCAFG00845006407	675	675	713	703
ENSCAFG00845006408	0	0	0	0
ENSCAFG00845006409	1	1	0	0
ENSCAFG00845018499	12	13	10	17
ENSCAFG00845018498	58	59	54	53
ENSCAFG00845018497	5	1	1	0
ENSCAFG00845018496	2324	2371	2245	2282
ENSCAFG00845018495	0	0	0	0
ENSCAFG00845018494	6475	6219	5610	5724
ENSCAFG00845018492	0	0	0	0
ENSCAFG00845018491	0	0	0	0
ENSCAFG00845018490	0	0	0	0
ENSCAFG00845018489	0	0	2	2
ENSCAFG00845018488	1180	1042	995	1106
ENSCAFG00845018487	1734	1529	1644	1627
ENSCAFG00845018486	926	874	1004	982
ENSCAFG00845018485	81	94	78	102
ENSCAFG00845018484	0	0	0	0
ENSCAFG00845018483	133	93	126	127
ENSCAFG00845018482	0	0	0	0
ENSCAFG00845018481	0	3	4	2
ENSCAFG00845018480	0	0	0	0
ENSCAFG00845018479	26	18	8	20
ENSCAFG00845018478	16	12	21	18
ENSCAFG00845018477	2	1	2	3
ENSCAFG00845018476	2	2	0	5

ENSCAFG00845018475	193	186	195	204
ENSCAFG00845006490	0	0	0	0
ENSCAFG00845018474	295	300	308	319
ENSCAFG00845006491	47	54	41	59
ENSCAFG00845006492	3608	3681	3652	3689
ENSCAFG00845018472	360	450	373	335
ENSCAFG00845006493	0	0	0	0
ENSCAFG00845018471	573	581	984	914
ENSCAFG00845006494	429	406	419	382
ENSCAFG00845018470	0	0	0	0
ENSCAFG00845006495	0	0	0	3
ENSCAFG00845006496	5254	5227	5135	5339
ENSCAFG00845006497	0	0	0	0
ENSCAFG00845006498	554	573	630	622
ENSCAFG00845006499	4453	4330	4146	4205
ENSCAFG00845018468	0	0	0	0
ENSCAFG00845018467	3	0	0	3
ENSCAFG00845018466	2	0	0	0
ENSCAFG00845018465	482	588	469	541
ENSCAFG00845018464	0	0	0	0
ENSCAFG00845018463	307	288	288	306
ENSCAFG00845006480	146	157	133	111
ENSCAFG00845018462	0	0	0	0
ENSCAFG00845006481	0	0	0	0
ENSCAFG00845018461	52	43	36	40
ENSCAFG00845006482	0	0	0	0
ENSCAFG00845018460	702	734	730	714
ENSCAFG00845006483	254	293	279	288
ENSCAFG00845006484	0	0	0	0
ENSCAFG00845006485	1	2	2	2
ENSCAFG00845006486	450	404	445	402
ENSCAFG00845006487	2	1	8	3
ENSCAFG00845006488	5636	5476	5475	5661
ENSCAFG00845006489	2261	2120	2046	2019
ENSCAFG00845018469	41	39	31	36
ENSCAFG00845018457	0	0	0	0
ENSCAFG00845018455	81	101	113	125
ENSCAFG00845018454	12	18	12	14
ENSCAFG00845018453	743	720	521	643
ENSCAFG00845018452	2	4	3	4
ENSCAFG00845018451	0	0	0	0

ENSCAFG00845006470	54	48	67	54
ENSCAFG00845018450	0	0	0	0
ENSCAFG00845006471	13	13	12	5
ENSCAFG00845006472	3	0	7	10
ENSCAFG00845006473	0	0	0	0
ENSCAFG00845006474	0	0	0	0
ENSCAFG00845006475	0	0	0	0
ENSCAFG00845006476	0	0	0	0
ENSCAFG00845006477	136	97	147	115
ENSCAFG00845006478	2	1	1	2
ENSCAFG00845006479	690	590	712	737
ENSCAFG00845018459	0	0	0	0
ENSCAFG00845018458	0	0	0	0
ENSCAFG00845018567	298	334	264	296
ENSCAFG00845018566	0	0	0	0
ENSCAFG00845018565	0	0	0	2
ENSCAFG00845018564	3	3	0	6
ENSCAFG00845018563	0	0	0	0
ENSCAFG00845018562	2647	2506	2206	2088
ENSCAFG00845018561	0	0	0	0
ENSCAFG00845006580	222	238	211	227
ENSCAFG00845018560	0	0	0	0
ENSCAFG00845006581	9	5	4	7
ENSCAFG00845006582	0	0	0	0
ENSCAFG00845006583	6	0	3	0
ENSCAFG00845006584	2	0	4	6
ENSCAFG00845006585	43	45	41	41
ENSCAFG00845006586	2	0	1	0
ENSCAFG00845006587	0	3	0	0
ENSCAFG00845006588	0	0	0	0
ENSCAFG00845006589	101	135	131	127
ENSCAFG00845018569	89	142	74	98
ENSCAFG00845018568	0	0	0	0
ENSCAFG00845018556	262	186	216	211
ENSCAFG00845018555	147	137	164	129
ENSCAFG00845018554	17	21	15	27
ENSCAFG00845018553	1	0	0	0
ENSCAFG00845018552	4	1	2	4
ENSCAFG00845018551	0	0	0	0
ENSCAFG00845018550	1131	992	1112	1179
ENSCAFG00845006570	10	15	7	7

ENSCAFG00845006571	3	0	0	0
ENSCAFG00845006572	1047	908	1061	1132
ENSCAFG00845006573	0	0	0	0
ENSCAFG00845006574	0	0	0	0
ENSCAFG00845006575	7	2	2	0
ENSCAFG00845006576	1279	1309	1083	1110
ENSCAFG00845006577	0	0	1	0
ENSCAFG00845006578	373	405	364	393
ENSCAFG00845006579	2	0	1	0
ENSCAFG00845018559	356	385	413	429
ENSCAFG00845018558	10	0	8	10
ENSCAFG00845018557	0	0	0	0
ENSCAFG00845018545	756	768	804	755
ENSCAFG00845018543	307	346	278	266
ENSCAFG00845018542	0	0	0	0
ENSCAFG00845018541	0	0	0	0
ENSCAFG00845018540	0	0	0	0
ENSCAFG00845006560	653	624	632	653
ENSCAFG00845006561	0	0	0	0
ENSCAFG00845006562	0	0	0	0
ENSCAFG00845006563	524	475	503	507
ENSCAFG00845006564	35	37	43	27
ENSCAFG00845006565	0	0	0	0
ENSCAFG00845006566	9047	9012	10141	10253
ENSCAFG00845006567	769	751	753	772
ENSCAFG00845006568	86	67	72	60
ENSCAFG00845006569	128	88	104	109
ENSCAFG00845018549	0	0	0	0
ENSCAFG00845018548	0	0	0	0
ENSCAFG00845018547	0	0	0	0
ENSCAFG00845018546	90	88	70	88
ENSCAFG00845018534	0	0	0	0
ENSCAFG00845018533	0	0	0	0
ENSCAFG00845018532	30	30	32	32
ENSCAFG00845018531	833	823	879	903
ENSCAFG00845018530	1852	1954	1826	2097
ENSCAFG00845006550	1290	1103	1728	1552
ENSCAFG00845006551	0	1	2	0
ENSCAFG00845006552	65	67	35	45
ENSCAFG00845006553	0	0	0	0
ENSCAFG00845006554	549	473	433	502

ENSCAFG00845006555	764	744	816	783
ENSCAFG00845006556	0	0	0	0
ENSCAFG00845006557	0	0	0	0
ENSCAFG00845006558	0	0	0	0
ENSCAFG00845006559	0	0	0	0
ENSCAFG00845018539	1295	1267	1255	1238
ENSCAFG00845018538	0	0	0	0
ENSCAFG00845018536	22	35	12	20
ENSCAFG00845018535	53	30	51	46
ENSCAFG00845018523	0	0	0	0
ENSCAFG00845018522	0	0	0	0
ENSCAFG00845018521	167	152	170	134
ENSCAFG00845006540	0	0	0	1
ENSCAFG00845006541	1003	1016	1351	1297
ENSCAFG00845006542	0	1	1	0
ENSCAFG00845006543	1	1	0	2
ENSCAFG00845006544	379	426	383	405
ENSCAFG00845006545	0	0	0	0
ENSCAFG00845006546	0	0	0	0
ENSCAFG00845006547	7	6	8	6
ENSCAFG00845006548	151	139	128	126
ENSCAFG00845006549	1	2	2	0
ENSCAFG00845018529	1	0	0	0
ENSCAFG00845018528	12	5	2	2
ENSCAFG00845018527	2113	2123	1838	2013
ENSCAFG00845018526	308	219	284	322
ENSCAFG00845018525	0	0	0	0
ENSCAFG00845018524	0	0	0	0
ENSCAFG00845018512	0	0	0	0
ENSCAFG00845018511	13	10	17	10
ENSCAFG00845018510	552	508	524	564
ENSCAFG00845006530	860	789	973	974
ENSCAFG00845006531	2	2	3	4
ENSCAFG00845006532	1	0	0	0
ENSCAFG00845006533	147	148	112	161
ENSCAFG00845006534	0	0	0	0
ENSCAFG00845006535	6	10	17	14
ENSCAFG00845006536	799	760	675	723
ENSCAFG00845006537	199	171	115	93
ENSCAFG00845006538	0	0	0	0
ENSCAFG00845006539	1	0	0	1

ENSCAFG00845018519	177	143	157	128
ENSCAFG00845018518	0	0	0	3
ENSCAFG00845018517	9	4	11	3
ENSCAFG00845018516	0	0	0	0
ENSCAFG00845018515	0	0	0	0
ENSCAFG00845018514	0	0	0	0
ENSCAFG00845018513	0	0	0	0
ENSCAFG00845018501	10	7	1	7
ENSCAFG00845018500	0	0	0	0
ENSCAFG00845006520	24	27	20	32
ENSCAFG00845006521	190	169	178	200
ENSCAFG00845006522	67	60	50	40
ENSCAFG00845006523	1	0	0	0
ENSCAFG00845006524	0	0	0	0
ENSCAFG00845006525	10	12	10	10
ENSCAFG00845006526	10	11	10	8
ENSCAFG00845006527	0	0	0	0
ENSCAFG00845006528	0	0	0	0
ENSCAFG00845006529	3145	3072	2906	3037
ENSCAFG00845018509	798	710	872	904
ENSCAFG00845018508	0	0	0	0
ENSCAFG00845018507	0	0	0	0
ENSCAFG00845018506	137	112	138	151
ENSCAFG00845018505	0	0	0	0
ENSCAFG00845018504	0	0	0	0
ENSCAFG00845018503	0	0	0	0
ENSCAFG00845018502	0	0	0	0
ENSCAFG00845006510	0	0	0	0
ENSCAFG00845006511	679	643	573	577
ENSCAFG00845006512	0	2	1	1
ENSCAFG00845006513	0	0	0	0
ENSCAFG00845006514	0	0	0	0
ENSCAFG00845006515	0	0	0	0
ENSCAFG00845006516	0	0	0	0
ENSCAFG00845006517	1894	2034	1908	1859
ENSCAFG00845006518	2379	2367	2431	2424
ENSCAFG00845006519	2	0	0	0
ENSCAFG00845018599	5	1	1	4
ENSCAFG00845018598	3428	3250	3267	3245
ENSCAFG00845018597	9	3	5	6
ENSCAFG00845018596	1289	1153	1257	1208

ENSCAFG00845018595	0	0	0	0
ENSCAFG00845018594	1220	1070	1197	1297
ENSCAFG00845018593	483	501	495	448
ENSCAFG00845018592	1	1	2	0
ENSCAFG00845018591	0	0	0	0
ENSCAFG00845018590	1	0	0	0
ENSCAFG00845018589	2543	2508	2707	2570
ENSCAFG00845018588	0	0	0	0
ENSCAFG00845018587	1	1	1	2
ENSCAFG00845018586	0	0	0	0
ENSCAFG00845018585	0	0	0	0
ENSCAFG00845018583	0	0	0	0
ENSCAFG00845018582	1	1	0	0
ENSCAFG00845018581	2	0	1	0
ENSCAFG00845018580	0	0	0	0
ENSCAFG00845018578	0	0	0	0
ENSCAFG00845018577	1116	1109	1041	1074
ENSCAFG00845018576	0	2	0	0
ENSCAFG00845018575	0	0	0	0
ENSCAFG00845018574	0	0	0	0
ENSCAFG00845018573	561	572	602	512
ENSCAFG00845006590	478	453	435	423
ENSCAFG00845018572	1	3	1	4
ENSCAFG00845006591	0	0	0	0
ENSCAFG00845018571	2	0	0	0
ENSCAFG00845006592	2176	2200	2151	2251
ENSCAFG00845018570	2	0	0	0
ENSCAFG00845006593	0	0	0	0
ENSCAFG00845006594	0	0	0	0
ENSCAFG00845006595	0	0	1	0
ENSCAFG00845006596	745	686	697	683
ENSCAFG00845006597	0	0	0	0
ENSCAFG00845006598	5	4	8	1
ENSCAFG00845006599	72	64	25	45
ENSCAFG00845018579	1055	957	1009	1001
ENSCAFG00845006500	208	178	176	164
ENSCAFG00845006501	461	459	439	418
ENSCAFG00845006502	488	524	555	604
ENSCAFG00845006503	15	12	16	7
ENSCAFG00845006504	0	0	0	0
ENSCAFG00845006505	0	2	0	0

ENSCAFG00845006506	1	4	0	2
ENSCAFG00845006507	2	4	1	0
ENSCAFG00845006508	0	0	0	0
ENSCAFG00845006509	3280	3184	2767	2947
ENSCAFG00845008401	0	0	0	0
ENSCAFG00845008400	1	0	0	1
ENSCAFG00845008403	0	0	0	0
ENSCAFG00845008402	832	878	812	817
ENSCAFG00845008405	10	4	5	4
ENSCAFG00845008404	0	0	0	0
ENSCAFG00845008407	27	24	19	13
ENSCAFG00845008406	0	0	0	0
ENSCAFG00845008409	0	0	0	0
ENSCAFG00845008408	0	0	0	0
ENSCAFG00845021458	655	598	610	558
ENSCAFG00845021459	411	354	379	364
ENSCAFG00845008481	0	0	0	0
ENSCAFG00845008480	1	0	2	0
ENSCAFG00845008483	0	1	0	0
ENSCAFG00845008482	11	21	12	7
ENSCAFG00845008485	0	0	0	0
ENSCAFG00845021452	0	0	0	0
ENSCAFG00845008484	54	38	34	50
ENSCAFG00845021453	0	0	0	0
ENSCAFG00845008487	5	3	2	4
ENSCAFG00845021450	0	0	0	0
ENSCAFG00845008486	1	1	2	1
ENSCAFG00845021451	2214	2275	2175	2257
ENSCAFG00845008489	0	0	0	0
ENSCAFG00845021456	9359	9143	8440	8385
ENSCAFG00845008488	389	326	418	400
ENSCAFG00845021457	0	0	0	0
ENSCAFG00845021454	65	67	89	66
ENSCAFG00845021455	2	0	0	0
ENSCAFG00845021449	2	2	0	1
ENSCAFG00845021447	493	412	440	457
ENSCAFG00845021448	147	116	120	127
ENSCAFG00845008470	0	0	0	0
ENSCAFG00845008472	0	0	0	0
ENSCAFG00845008471	239	289	320	238
ENSCAFG00845008474	0	0	0	0

ENSCAFG00845021441	0	0	0	0
ENSCAFG00845008473	0	0	0	0
ENSCAFG00845021442	0	0	0	0
ENSCAFG00845008476	0	0	0	0
ENSCAFG00845008475	378	401	350	362
ENSCAFG00845021440	0	0	0	0
ENSCAFG00845008478	0	0	0	0
ENSCAFG00845008477	496	427	481	510
ENSCAFG00845021446	0	0	0	0
ENSCAFG00845021443	0	2	1	0
ENSCAFG00845008479	0	0	0	0
ENSCAFG00845021444	0	0	0	0
ENSCAFG00845021438	0	0	0	0
ENSCAFG00845021439	0	0	0	0
ENSCAFG00845021436	0	0	0	0
ENSCAFG00845021437	3	2	0	0
ENSCAFG00845008461	0	0	0	0
ENSCAFG00845008460	0	0	1	0
ENSCAFG00845008463	0	0	0	0
ENSCAFG00845021430	0	0	0	0
ENSCAFG00845008462	432	416	380	392
ENSCAFG00845021431	148	174	191	222
ENSCAFG00845008465	2062	1988	1931	2002
ENSCAFG00845008464	337	327	306	360
ENSCAFG00845008467	566	485	532	553
ENSCAFG00845021434	5	1	1	1
ENSCAFG00845008466	0	0	0	0
ENSCAFG00845021435	32	38	27	22
ENSCAFG00845008469	0	0	0	0
ENSCAFG00845021432	145	178	167	201
ENSCAFG00845008468	1554	1564	1546	1674
ENSCAFG00845021433	0	0	0	0
ENSCAFG00845021427	0	0	0	0
ENSCAFG00845021428	0	0	0	0
ENSCAFG00845021425	1080	1067	1092	1078
ENSCAFG00845021426	37	21	29	47
ENSCAFG00845008450	1	1	0	1
ENSCAFG00845021429	0	0	0	0
ENSCAFG00845008452	12	6	9	6
ENSCAFG00845008451	3803	3727	3671	3547
ENSCAFG00845021420	2	2	1	0

ENSCAFG00845008454	0	0	0	0
ENSCAFG00845008453	1	1	0	1
ENSCAFG00845008456	404	347	363	341
ENSCAFG00845021423	0	0	0	0
ENSCAFG00845008455	49	44	43	40
ENSCAFG00845021424	0	0	0	0
ENSCAFG00845008458	149	156	124	133
ENSCAFG00845021421	5051	5072	4980	4792
ENSCAFG00845008457	368	320	324	334
ENSCAFG00845021422	906	920	932	892
ENSCAFG00845008459	0	0	0	0
ENSCAFG00845021416	0	0	0	0
ENSCAFG00845021417	0	0	0	0
ENSCAFG00845021414	0	0	0	0
ENSCAFG00845021415	0	0	0	0
ENSCAFG00845021418	0	0	0	0
ENSCAFG00845021419	1388	1406	1524	1533
ENSCAFG00845008441	0	0	0	0
ENSCAFG00845008440	0	0	1	0
ENSCAFG00845008443	1000	885	910	954
ENSCAFG00845008442	0	0	0	0
ENSCAFG00845008445	2	7	3	4
ENSCAFG00845021412	0	0	0	0
ENSCAFG00845008444	443	464	419	388
ENSCAFG00845021413	53	55	39	63
ENSCAFG00845008447	1171	1119	1069	1181
ENSCAFG00845021410	804	723	798	774
ENSCAFG00845008446	1036	992	971	1072
ENSCAFG00845021411	0	0	0	0
ENSCAFG00845008449	16	7	10	18
ENSCAFG00845008448	0	0	0	0
ENSCAFG00845021405	0	0	0	0
ENSCAFG00845021406	0	0	0	0
ENSCAFG00845021403	183	173	159	164
ENSCAFG00845021404	0	0	0	0
ENSCAFG00845021409	179	198	188	239
ENSCAFG00845021407	0	0	0	0
ENSCAFG00845021408	300	251	277	245
ENSCAFG00845008430	0	0	0	0
ENSCAFG00845008432	2420	2293	2255	2238
ENSCAFG00845008431	55	38	55	55

ENSCAFG00845008434	0	0	0	0
ENSCAFG00845021401	0	0	0	0
ENSCAFG00845008433	0	0	0	0
ENSCAFG00845021402	120	124	125	111
ENSCAFG00845008436	0	0	0	0
ENSCAFG00845008435	0	0	0	0
ENSCAFG00845021400	0	0	0	0
ENSCAFG00845008438	0	0	0	0
ENSCAFG00845008437	686	689	670	677
ENSCAFG00845008439	219	193	183	152
ENSCAFG00845008421	0	0	0	0
ENSCAFG00845008420	53	33	39	40
ENSCAFG00845008423	2	7	10	8
ENSCAFG00845008422	0	0	0	0
ENSCAFG00845008425	0	0	0	0
ENSCAFG00845008424	131	100	79	81
ENSCAFG00845008427	0	0	0	0
ENSCAFG00845008426	0	0	0	0
ENSCAFG00845008429	0	0	0	0
ENSCAFG00845008428	0	0	0	0
ENSCAFG00845008410	0	0	0	0
ENSCAFG00845008412	0	0	0	0
ENSCAFG00845008411	0	0	0	0
ENSCAFG00845008414	17	13	27	23
ENSCAFG00845008413	1164	1147	1097	1100
ENSCAFG00845008416	0	0	0	0
ENSCAFG00845008415	62	80	59	74
ENSCAFG00845008418	47	37	52	47
ENSCAFG00845008417	0	0	0	0
ENSCAFG00845008419	0	0	0	0
ENSCAFG00845021397	0	0	0	0
ENSCAFG00845021398	6	4	5	6
ENSCAFG00845021395	0	0	0	0
ENSCAFG00845021396	0	0	0	0
ENSCAFG00845021399	0	0	0	0
ENSCAFG00845021390	323	331	323	401
ENSCAFG00845021393	0	1	1	1
ENSCAFG00845021394	0	0	1	0
ENSCAFG00845021391	6	0	0	2
ENSCAFG00845021392	0	0	0	0
ENSCAFG00845021386	652	646	614	721

ENSCAFG00845021387	246	248	235	231
ENSCAFG00845021384	1	0	2	0
ENSCAFG00845021385	50	26	43	32
ENSCAFG00845021388	2033	1837	1898	1833
ENSCAFG00845021382	0	0	0	0
ENSCAFG00845021383	0	0	2	0
ENSCAFG00845021380	0	0	0	0
ENSCAFG00845021381	194	184	171	170
ENSCAFG00845021375	41	40	28	24
ENSCAFG00845021376	0	0	0	0
ENSCAFG00845021373	711	641	614	614
ENSCAFG00845021374	0	2	1	0
ENSCAFG00845021379	241	242	199	239
ENSCAFG00845021377	73	74	78	69
ENSCAFG00845021378	1596	1456	1198	1197
ENSCAFG00845021371	0	0	0	0
ENSCAFG00845021372	2086	1899	1875	1936
ENSCAFG00845021370	0	0	0	0
ENSCAFG00845008391	452	449	464	427
ENSCAFG00845008390	0	0	0	0
ENSCAFG00845008393	0	0	0	0
ENSCAFG00845008392	0	0	0	0
ENSCAFG00845008395	2	4	1	4
ENSCAFG00845008394	1365	1305	1215	1234
ENSCAFG00845008397	74	106	85	87
ENSCAFG00845021364	5	7	1	0
ENSCAFG00845008396	2713	2586	2397	2378
ENSCAFG00845021365	0	0	0	0
ENSCAFG00845008399	0	0	0	0
ENSCAFG00845021362	0	0	0	0
ENSCAFG00845008398	0	0	0	0
ENSCAFG00845021363	0	0	0	0
ENSCAFG00845021368	0	0	0	0
ENSCAFG00845021369	0	0	3	0
ENSCAFG00845021366	72	80	62	82
ENSCAFG00845021367	1196	1239	1137	1168
ENSCAFG00845021360	0	0	0	0
ENSCAFG00845021361	0	0	0	0
ENSCAFG00845008380	379	288	314	275
ENSCAFG00845021359	864	748	824	881
ENSCAFG00845008382	0	0	0	0

ENSCAFG00845008381	404	387	380	420
ENSCAFG00845008384	52	57	91	84
ENSCAFG00845008383	210	224	254	217
ENSCAFG00845008386	68	74	88	79
ENSCAFG00845021353	0	0	0	0
ENSCAFG00845008385	2095	2050	2003	1899
ENSCAFG00845021354	31	12	13	13
ENSCAFG00845008388	0	1	0	0
ENSCAFG00845021351	1554	1479	1518	1426
ENSCAFG00845008387	287	271	320	306
ENSCAFG00845021352	470	352	336	378
ENSCAFG00845021357	0	0	0	0
ENSCAFG00845008389	1175	1194	1220	1163
ENSCAFG00845021358	617	584	568	572
ENSCAFG00845021355	757	763	710	715
ENSCAFG00845021356	1	1	0	2
ENSCAFG00845021350	191	221	337	414
ENSCAFG00845021348	12	5	14	10
ENSCAFG00845021349	1	1	3	6
ENSCAFG00845008371	879	801	801	820
ENSCAFG00845008370	114	114	148	184
ENSCAFG00845008373	23	26	39	22
ENSCAFG00845008372	5051	4742	4522	4528
ENSCAFG00845008375	0	0	0	0
ENSCAFG00845021342	4	6	8	2
ENSCAFG00845008374	896	921	919	858
ENSCAFG00845021343	0	0	0	0
ENSCAFG00845008377	6	5	4	2
ENSCAFG00845021340	738	744	718	718
ENSCAFG00845008376	0	0	0	0
ENSCAFG00845021341	47	47	49	58
ENSCAFG00845008379	24	20	7	14
ENSCAFG00845021346	1	0	0	0
ENSCAFG00845008378	185	209	173	185
ENSCAFG00845021347	0	0	1	0
ENSCAFG00845021344	0	0	2	0
ENSCAFG00845021345	580	558	597	547
ENSCAFG00845008520	0	0	0	0
ENSCAFG00845008522	0	0	0	0
ENSCAFG00845008521	0	0	0	0
ENSCAFG00845008524	1226	1307	1199	1181

ENSCAFG00845008523	13	11	20	19
ENSCAFG00845008526	251	227	240	227
ENSCAFG00845008525	1287	1144	1219	1277
ENSCAFG00845008528	1120	1057	1164	1135
ENSCAFG00845008527	0	0	0	0
ENSCAFG00845008529	0	0	0	0
ENSCAFG00845008511	62	69	60	76
ENSCAFG00845008510	0	0	0	0
ENSCAFG00845008513	2712	2554	2618	2554
ENSCAFG00845008512	908	884	858	914
ENSCAFG00845008515	3411	3444	3011	2988
ENSCAFG00845008514	0	0	0	0
ENSCAFG00845008517	403	425	386	357
ENSCAFG00845008516	0	0	0	0
ENSCAFG00845008519	328	348	300	285
ENSCAFG00845008518	1013	1041	1041	1016
ENSCAFG00845008500	0	0	0	0
ENSCAFG00845008502	0	0	0	0
ENSCAFG00845008501	1174	1127	974	946
ENSCAFG00845008504	371	319	140	163
ENSCAFG00845008503	0	0	0	0
ENSCAFG00845008506	0	0	0	0
ENSCAFG00845008505	1	1	1	2
ENSCAFG00845008508	78	85	47	48
ENSCAFG00845008507	0	0	0	0
ENSCAFG00845008509	5	3	9	9
ENSCAFG00845021579	0	0	0	0
ENSCAFG00845021573	6	7	16	15
ENSCAFG00845021574	418	384	389	394
ENSCAFG00845021572	241	187	260	208
ENSCAFG00845021577	11	12	2	5
ENSCAFG00845021578	4447	4374	4626	4679
ENSCAFG00845021575	1	0	0	0
ENSCAFG00845021576	0	0	1	1
ENSCAFG00845021570	47	29	45	42
ENSCAFG00845021568	0	0	0	0
ENSCAFG00845021569	5	1	0	0
ENSCAFG00845008591	761	807	882	771
ENSCAFG00845008590	2	9	3	2
ENSCAFG00845008593	421	439	436	442
ENSCAFG00845008592	1313	1279	1308	1211

ENSCAFG00845008595	0	0	0	0
ENSCAFG00845021562	0	0	0	0
ENSCAFG00845008594	0	0	0	0
ENSCAFG00845021563	1	2	4	6
ENSCAFG00845008597	906	971	847	836
ENSCAFG00845021560	1264	1212	1249	1220
ENSCAFG00845008596	283	272	302	246
ENSCAFG00845021561	16	14	13	22
ENSCAFG00845008599	41	41	37	53
ENSCAFG00845021566	173	213	218	180
ENSCAFG00845008598	0	0	0	0
ENSCAFG00845021567	0	0	0	0
ENSCAFG00845021564	0	0	0	0
ENSCAFG00845021565	2004	1850	2004	2060
ENSCAFG00845021559	7	3	7	8
ENSCAFG00845021557	0	0	0	0
ENSCAFG00845021558	29	26	22	26
ENSCAFG00845008580	205	169	158	181
ENSCAFG00845008582	0	0	0	0
ENSCAFG00845008581	0	0	0	0
ENSCAFG00845008584	474	495	466	496
ENSCAFG00845021551	11	9	6	12
ENSCAFG00845008583	0	0	0	0
ENSCAFG00845021552	43	50	54	43
ENSCAFG00845008586	0	1	0	4
ENSCAFG00845008585	0	0	0	0
ENSCAFG00845021550	821	790	760	755
ENSCAFG00845008588	0	0	0	0
ENSCAFG00845021555	2216	2152	2042	2052
ENSCAFG00845008587	140	117	98	99
ENSCAFG00845021556	0	0	0	0
ENSCAFG00845021553	207	232	243	202
ENSCAFG00845008589	2	4	8	2
ENSCAFG00845021554	0	0	0	0
ENSCAFG00845021548	16	23	20	21
ENSCAFG00845021549	1440	1373	1555	1380
ENSCAFG00845021546	0	0	0	0
ENSCAFG00845021547	23	39	31	28
ENSCAFG00845008571	0	0	0	1
ENSCAFG00845008570	87	80	82	108
ENSCAFG00845008573	0	1	1	2

ENSCAFG00845021540	123	140	117	101
ENSCAFG00845008572	22	35	41	37
ENSCAFG00845021541	4	2	2	2
ENSCAFG00845008575	443	433	433	456
ENSCAFG00845008574	9	8	18	6
ENSCAFG00845008577	0	0	0	0
ENSCAFG00845021544	230	204	175	197
ENSCAFG00845008576	12	5	13	8
ENSCAFG00845021545	0	0	0	0
ENSCAFG00845008579	56	67	48	50
ENSCAFG00845021542	12	4	17	14
ENSCAFG00845008578	750	716	859	888
ENSCAFG00845021543	1300	1251	1154	1088
ENSCAFG00845021537	950	926	958	938
ENSCAFG00845021538	1754	1645	1476	1602
ENSCAFG00845021535	3	2	2	0
ENSCAFG00845021536	0	0	0	0
ENSCAFG00845008560	63	53	51	44
ENSCAFG00845021539	0	0	0	0
ENSCAFG00845008562	29	15	17	17
ENSCAFG00845008561	2373	2127	2362	2431
ENSCAFG00845021530	1271	1266	1334	1280
ENSCAFG00845008564	0	0	0	0
ENSCAFG00845008563	40	26	43	15
ENSCAFG00845008566	0	0	0	0
ENSCAFG00845008565	0	0	0	0
ENSCAFG00845021534	90	81	86	61
ENSCAFG00845008568	0	1	0	0
ENSCAFG00845021531	16	23	26	8
ENSCAFG00845008567	602	606	548	525
ENSCAFG00845021532	2	0	3	3
ENSCAFG00845008569	12	11	26	12
ENSCAFG00845021526	4641	4566	4831	4785
ENSCAFG00845021527	32	41	21	20
ENSCAFG00845021524	1	0	1	0
ENSCAFG00845021525	5	7	5	7
ENSCAFG00845021528	0	1	0	0
ENSCAFG00845021529	1107	1071	1219	1187
ENSCAFG00845008551	98	85	71	69
ENSCAFG00845008550	0	0	0	0
ENSCAFG00845008553	515	453	359	332

ENSCAFG00845008552	277	274	260	305
ENSCAFG00845008555	0	0	0	0
ENSCAFG00845021522	211	197	196	207
ENSCAFG00845008554	0	0	0	0
ENSCAFG00845021523	0	4	0	0
ENSCAFG00845008557	0	0	0	0
ENSCAFG00845021520	2302	2152	1797	1980
ENSCAFG00845008556	0	0	0	0
ENSCAFG00845021521	0	0	0	0
ENSCAFG00845008559	0	0	0	0
ENSCAFG00845008558	0	0	0	0
ENSCAFG00845021515	270	229	190	199
ENSCAFG00845021516	0	0	0	0
ENSCAFG00845021513	0	1	1	0
ENSCAFG00845021514	23	8	8	12
ENSCAFG00845021519	687	585	756	776
ENSCAFG00845021517	300	308	284	253
ENSCAFG00845021518	48	46	49	50
ENSCAFG00845008540	0	0	0	0
ENSCAFG00845008542	305	322	289	324
ENSCAFG00845008541	1051	881	905	976
ENSCAFG00845008544	0	0	0	0
ENSCAFG00845021511	305	292	302	275
ENSCAFG00845008543	245	264	207	216
ENSCAFG00845021512	1081	973	1026	937
ENSCAFG00845008546	0	0	1	0
ENSCAFG00845008545	0	0	0	0
ENSCAFG00845021510	0	0	0	0
ENSCAFG00845008548	0	0	0	0
ENSCAFG00845008547	0	0	0	0
ENSCAFG00845008549	603	521	678	688
ENSCAFG00845021504	750	756	800	810
ENSCAFG00845021505	142	158	188	166
ENSCAFG00845021502	0	1	0	1
ENSCAFG00845021503	2051	2047	1939	2050
ENSCAFG00845021508	0	0	0	0
ENSCAFG00845021509	0	3	1	2
ENSCAFG00845021506	961	899	917	879
ENSCAFG00845021507	379	309	332	308
ENSCAFG00845008531	178	167	178	178
ENSCAFG00845008530	16	16	19	20

ENSCAFG00845008533	58	54	70	75
ENSCAFG00845021500	0	0	0	0
ENSCAFG00845008532	0	0	0	0
ENSCAFG00845021501	0	0	0	0
ENSCAFG00845008535	0	0	0	0
ENSCAFG00845008534	4	1	4	0
ENSCAFG00845008537	2111	2074	2286	2365
ENSCAFG00845008536	0	0	0	0
ENSCAFG00845008539	0	0	0	0
ENSCAFG00845008538	0	0	0	0
ENSCAFG00845021496	0	0	0	0
ENSCAFG00845021497	5942	5626	5525	5714
ENSCAFG00845021494	0	0	0	0
ENSCAFG00845021498	1	1	0	3
ENSCAFG00845021499	0	0	0	0
ENSCAFG00845021492	0	0	0	0
ENSCAFG00845021493	2	2	1	2
ENSCAFG00845021490	44	44	30	24
ENSCAFG00845021491	1384	1294	1497	1600
ENSCAFG00845021485	16	5	10	7
ENSCAFG00845021486	0	0	0	0
ENSCAFG00845021483	9	2	1	2
ENSCAFG00845021484	2329	2301	2813	2927
ENSCAFG00845021489	1	5	0	0
ENSCAFG00845021487	1	0	1	2
ENSCAFG00845021488	0	0	0	0
ENSCAFG00845021481	1525	1494	1427	1467
ENSCAFG00845021482	24	5	12	14
ENSCAFG00845021480	0	0	0	0
ENSCAFG00845021474	287	301	313	301
ENSCAFG00845021475	678	650	640	580
ENSCAFG00845021472	0	1	1	4
ENSCAFG00845021473	1300	1195	1305	1270
ENSCAFG00845021478	6	5	21	6
ENSCAFG00845021479	0	0	0	0
ENSCAFG00845021476	473	467	465	442
ENSCAFG00845021477	0	0	0	0
ENSCAFG00845021470	4	3	0	2
ENSCAFG00845021471	92	98	117	115
ENSCAFG00845008490	0	0	0	0
ENSCAFG00845021469	1195	1178	1076	971

ENSCAFG00845008492	0	0	0	0
ENSCAFG00845008491	0	0	0	0
ENSCAFG00845008494	990	1016	993	1060
ENSCAFG00845008493	870	876	760	875
ENSCAFG00845008496	4101	4090	4018	4082
ENSCAFG00845021463	0	0	0	0
ENSCAFG00845008495	1890	1832	1808	1847
ENSCAFG00845021464	0	1	3	0
ENSCAFG00845008498	1605	1554	1670	1891
ENSCAFG00845021461	27	39	34	34
ENSCAFG00845008497	0	5	2	7
ENSCAFG00845021462	1088	1047	1031	1037
ENSCAFG00845021467	938	887	824	834
ENSCAFG00845008499	11	25	22	17
ENSCAFG00845021468	0	0	0	0
ENSCAFG00845021465	205	183	209	207
ENSCAFG00845021466	28	28	39	30
ENSCAFG00845021460	553	582	794	694
ENSCAFG00845021614	0	0	0	0
ENSCAFG00845021615	6	4	5	7
ENSCAFG00845021612	4	4	4	3
ENSCAFG00845021613	0	0	0	0
ENSCAFG00845021618	0	0	0	0
ENSCAFG00845021619	429	400	417	439
ENSCAFG00845021616	0	0	0	0
ENSCAFG00845021617	2209	2198	2030	1967
ENSCAFG00845008641	581	568	636	607
ENSCAFG00845008640	2	0	5	3
ENSCAFG00845008643	626	610	577	680
ENSCAFG00845021610	803	794	753	848
ENSCAFG00845008642	184	188	248	246
ENSCAFG00845021611	0	0	0	0
ENSCAFG00845008645	681	590	623	643
ENSCAFG00845008644	546	533	578	578
ENSCAFG00845008647	0	0	0	0
ENSCAFG00845008646	0	0	0	0
ENSCAFG00845008649	0	0	0	2
ENSCAFG00845008648	0	0	0	0
ENSCAFG00845021609	0	0	0	0
ENSCAFG00845021603	636	571	582	555
ENSCAFG00845021604	0	0	0	0

ENSCAFG00845021601	0	0	0	0
ENSCAFG00845021602	12	7	12	11
ENSCAFG00845021607	54	44	48	60
ENSCAFG00845021608	1068	1058	968	1051
ENSCAFG00845021605	0	0	0	0
ENSCAFG00845021606	2	2	3	3
ENSCAFG00845008630	0	0	0	0
ENSCAFG00845008632	712	677	655	641
ENSCAFG00845008631	0	0	0	0
ENSCAFG00845021600	0	0	0	0
ENSCAFG00845008634	0	0	0	0
ENSCAFG00845008633	0	0	0	0
ENSCAFG00845008636	0	0	0	0
ENSCAFG00845008635	124	124	117	126
ENSCAFG00845008638	0	0	0	0
ENSCAFG00845008637	0	0	0	0
ENSCAFG00845008639	460	470	484	560
ENSCAFG00845008621	433	411	405	392
ENSCAFG00845008620	0	0	0	0
ENSCAFG00845008623	0	0	0	0
ENSCAFG00845008622	410	390	428	401
ENSCAFG00845008625	0	1	1	0
ENSCAFG00845008624	0	0	0	0
ENSCAFG00845008627	592	544	460	423
ENSCAFG00845008626	0	0	0	0
ENSCAFG00845008629	0	0	0	0
ENSCAFG00845008628	570	549	549	572
ENSCAFG00845008610	1	2	1	2
ENSCAFG00845008612	0	0	0	0
ENSCAFG00845008611	0	0	0	0
ENSCAFG00845008614	5	8	3	1
ENSCAFG00845008613	0	0	0	0
ENSCAFG00845008616	103	111	151	144
ENSCAFG00845008615	1	0	0	0
ENSCAFG00845008618	0	0	0	0
ENSCAFG00845008617	966	957	1039	1021
ENSCAFG00845008619	682	702	650	743
ENSCAFG00845008601	379	355	391	386
ENSCAFG00845008600	0	0	0	0
ENSCAFG00845008603	1218	1271	1543	1526
ENSCAFG00845008602	66	61	68	52

ENSCAFG00845008605	0	0	0	0
ENSCAFG00845008604	0	0	0	0
ENSCAFG00845008607	727	792	729	719
ENSCAFG00845008606	0	0	0	0
ENSCAFG00845008609	113	87	94	73
ENSCAFG00845008608	0	1	2	0
ENSCAFG00845031006	0	0	0	0
ENSCAFG00845031007	0	0	0	0
ENSCAFG00845031008	61	46	72	80
ENSCAFG00845031009	6	9	5	11
ENSCAFG00845031000	485	430	403	481
ENSCAFG00845031001	39	23	27	22
ENSCAFG00845031002	0	0	0	0
ENSCAFG00845031003	0	0	0	0
ENSCAFG00845031004	0	1	0	0
ENSCAFG00845031005	1282	1288	1085	1165
ENSCAFG00845031017	93	64	110	84
ENSCAFG00845031018	622	590	551	670
ENSCAFG00845031019	0	0	0	0
ENSCAFG00845031010	0	0	0	0
ENSCAFG00845031011	0	0	0	0
ENSCAFG00845031012	0	0	0	0
ENSCAFG00845031013	0	0	0	0
ENSCAFG00845031014	405	523	522	520
ENSCAFG00845031015	467	388	382	434
ENSCAFG00845031016	0	0	0	0
ENSCAFG00845018049	0	0	0	0
ENSCAFG00845018048	0	0	0	0
ENSCAFG00845018047	58	80	90	98
ENSCAFG00845006060	676	632	620	665
ENSCAFG00845018046	0	0	0	0
ENSCAFG00845006061	0	0	0	0
ENSCAFG00845018045	1676	1502	1736	1716
ENSCAFG00845006062	0	0	0	0
ENSCAFG00845018044	7	3	0	0
ENSCAFG00845006063	10	15	5	7
ENSCAFG00845018043	36	37	53	48
ENSCAFG00845006064	2459	2337	2519	2548
ENSCAFG00845018042	0	0	0	0
ENSCAFG00845021694	757	774	623	671
ENSCAFG00845006065	2101	1919	2163	2394

ENSCAFG00845018041	0	0	0	0
ENSCAFG00845021695	0	0	0	0
ENSCAFG00845006066	0	0	0	0
ENSCAFG00845018040	1632	1496	1586	1750
ENSCAFG00845021692	0	0	0	0
ENSCAFG00845006067	0	0	0	1
ENSCAFG00845021693	0	0	0	0
ENSCAFG00845006068	731	761	699	694
ENSCAFG00845021698	11	13	20	17
ENSCAFG00845006069	1602	1629	1579	1527
ENSCAFG00845021699	0	0	1	0
ENSCAFG00845021696	0	0	0	0
ENSCAFG00845021697	0	0	0	0
ENSCAFG00845021690	0	0	0	0
ENSCAFG00845021691	0	0	0	0
ENSCAFG00845018039	70	73	84	75
ENSCAFG00845018038	4	2	21	11
ENSCAFG00845018037	0	0	0	0
ENSCAFG00845021689	617	603	554	620
ENSCAFG00845018036	0	0	0	0
ENSCAFG00845018035	0	0	0	0
ENSCAFG00845006050	1375	1333	1478	1515
ENSCAFG00845018034	0	0	0	0
ENSCAFG00845006051	0	0	0	0
ENSCAFG00845018033	0	0	0	0
ENSCAFG00845006052	0	0	1	2
ENSCAFG00845018032	0	0	0	0
ENSCAFG00845006053	0	0	1	3
ENSCAFG00845018031	0	0	0	0
ENSCAFG00845021683	2	4	0	0
ENSCAFG00845006054	195	218	166	171
ENSCAFG00845018030	0	0	0	0
ENSCAFG00845021684	0	0	0	0
ENSCAFG00845006055	0	0	0	0
ENSCAFG00845021681	459	447	435	420
ENSCAFG00845006056	6	1	0	1
ENSCAFG00845021682	0	0	0	0
ENSCAFG00845006057	0	2	0	0
ENSCAFG00845021687	179	142	148	173
ENSCAFG00845006058	0	0	0	0
ENSCAFG00845021688	1261	1250	1247	1235

ENSCAFG00845006059	671	645	562	700
ENSCAFG00845021685	433	457	447	462
ENSCAFG00845021686	1147	1142	1155	1130
ENSCAFG00845021680	0	0	0	0
ENSCAFG00845018028	0	0	0	0
ENSCAFG00845018027	0	0	0	0
ENSCAFG00845018026	5	4	10	6
ENSCAFG00845021678	1220	1121	1284	1261
ENSCAFG00845018025	180	209	132	148
ENSCAFG00845021679	0	0	0	0
ENSCAFG00845018024	0	0	0	0
ENSCAFG00845018023	8	8	27	13
ENSCAFG00845006040	1	0	1	2
ENSCAFG00845018022	16	3	5	4
ENSCAFG00845006041	497	476	477	473
ENSCAFG00845018021	0	1	0	0
ENSCAFG00845006042	0	0	0	0
ENSCAFG00845018020	1553	1361	1456	1488
ENSCAFG00845021672	1	2	0	0
ENSCAFG00845006043	577	596	616	533
ENSCAFG00845021673	1	4	3	5
ENSCAFG00845006044	606	510	541	521
ENSCAFG00845021670	0	6	3	1
ENSCAFG00845006045	12	4	11	10
ENSCAFG00845021671	0	0	0	0
ENSCAFG00845006046	41	50	50	46
ENSCAFG00845021676	0	0	0	0
ENSCAFG00845006047	0	0	0	0
ENSCAFG00845021677	5	9	8	6
ENSCAFG00845006048	0	0	0	0
ENSCAFG00845021674	6	1	1	8
ENSCAFG00845006049	0	0	0	0
ENSCAFG00845021675	46	38	46	32
ENSCAFG00845018029	160	158	170	146
ENSCAFG00845018017	0	0	0	0
ENSCAFG00845021669	0	0	0	0
ENSCAFG00845018016	0	0	0	0
ENSCAFG00845018015	21	7	21	19
ENSCAFG00845021667	174	210	183	170
ENSCAFG00845018014	0	0	0	0
ENSCAFG00845021668	23	29	25	20

ENSCAFG00845008690	45	45	40	45
ENSCAFG00845018013	0	0	0	0
ENSCAFG00845018012	380	328	390	413
ENSCAFG00845008692	0	0	0	0
ENSCAFG00845018011	0	0	0	0
ENSCAFG00845006030	1099	1155	1076	1118
ENSCAFG00845008691	0	0	0	0
ENSCAFG00845018010	0	1	2	4
ENSCAFG00845006031	0	0	0	0
ENSCAFG00845008694	5	2	8	8
ENSCAFG00845021661	832	809	755	794
ENSCAFG00845006032	145	132	155	180
ENSCAFG00845008693	194	250	255	164
ENSCAFG00845021662	0	0	0	0
ENSCAFG00845006033	0	0	0	0
ENSCAFG00845008696	1	0	1	6
ENSCAFG00845006034	293	289	378	315
ENSCAFG00845008695	44	40	20	13
ENSCAFG00845021660	277	271	284	276
ENSCAFG00845006035	37	18	23	33
ENSCAFG00845008698	27	26	14	23
ENSCAFG00845021665	1311	1201	1305	1252
ENSCAFG00845006036	0	0	0	0
ENSCAFG00845008697	0	0	1	3
ENSCAFG00845021666	7	12	25	16
ENSCAFG00845006037	2302	2211	2316	2286
ENSCAFG00845021663	57	54	57	66
ENSCAFG00845006038	126	124	85	93
ENSCAFG00845008699	0	2	0	2
ENSCAFG00845021664	51	44	52	32
ENSCAFG00845006039	0	0	0	0
ENSCAFG00845018019	0	0	0	0
ENSCAFG00845018018	34	14	17	23
ENSCAFG00845018006	323	345	396	374
ENSCAFG00845021658	326	269	279	290
ENSCAFG00845018005	1	3	3	1
ENSCAFG00845021659	0	0	0	0
ENSCAFG00845018004	0	0	0	0
ENSCAFG00845021656	0	0	0	0
ENSCAFG00845018003	0	0	0	0
ENSCAFG00845021657	138	135	153	150

ENSCAFG00845018002	0	0	0	0
ENSCAFG00845018001	0	0	0	0
ENSCAFG00845008681	1	0	0	0
ENSCAFG00845018000	0	0	0	0
ENSCAFG00845008680	0	0	0	0
ENSCAFG00845006020	0	0	0	0
ENSCAFG00845008683	0	0	1	1
ENSCAFG00845021650	1	1	0	0
ENSCAFG00845006021	1081	1033	1140	1127
ENSCAFG00845008682	544	518	497	510
ENSCAFG00845021651	8	9	4	16
ENSCAFG00845006022	0	0	0	0
ENSCAFG00845008685	0	0	0	0
ENSCAFG00845006023	0	0	0	0
ENSCAFG00845008684	0	0	0	0
ENSCAFG00845006024	1	1	0	3
ENSCAFG00845008687	388	344	275	326
ENSCAFG00845021654	0	0	0	0
ENSCAFG00845006025	1167	1033	1143	1242
ENSCAFG00845008686	0	0	0	0
ENSCAFG00845021655	1	1	0	0
ENSCAFG00845006026	0	0	0	0
ENSCAFG00845008689	3062	2896	2701	2841
ENSCAFG00845021652	120	97	69	81
ENSCAFG00845006027	283	260	284	288
ENSCAFG00845008688	794	797	853	851
ENSCAFG00845021653	709	672	717	691
ENSCAFG00845006028	0	0	0	0
ENSCAFG00845006029	0	0	0	0
ENSCAFG00845018009	0	1	11	2
ENSCAFG00845018008	1502	1409	1369	1423
ENSCAFG00845018007	0	0	0	0
ENSCAFG00845021647	325	330	350	344
ENSCAFG00845021648	0	2	0	1
ENSCAFG00845021645	1839	1832	1792	1828
ENSCAFG00845021646	17	9	20	6
ENSCAFG00845008670	0	0	0	0
ENSCAFG00845021649	657	717	652	707
ENSCAFG00845008672	0	0	0	0
ENSCAFG00845006010	795	787	1923	1894
ENSCAFG00845008671	47	30	26	28

ENSCAFG00845021640	0	0	0	0
ENSCAFG00845006011	0	0	0	0
ENSCAFG00845008674	978	941	812	836
ENSCAFG00845006012	1009	918	1058	955
ENSCAFG00845008673	19	10	18	13
ENSCAFG00845006013	0	0	1	3
ENSCAFG00845008676	2	3	2	0
ENSCAFG00845021643	0	0	0	0
ENSCAFG00845006014	0	0	0	0
ENSCAFG00845008675	12	12	15	17
ENSCAFG00845021644	4	0	0	0
ENSCAFG00845006015	155	166	167	129
ENSCAFG00845008678	0	0	0	0
ENSCAFG00845021641	320	309	244	285
ENSCAFG00845006016	10	5	8	9
ENSCAFG00845008677	1449	1504	1639	1561
ENSCAFG00845021642	99	135	118	144
ENSCAFG00845006017	2	0	2	1
ENSCAFG00845006018	0	0	0	0
ENSCAFG00845008679	1	0	0	0
ENSCAFG00845006019	9	16	5	10
ENSCAFG00845021636	427	412	402	431
ENSCAFG00845021637	0	0	0	0
ENSCAFG00845021635	0	0	0	0
ENSCAFG00845021638	0	0	0	0
ENSCAFG00845021639	1	1	0	0
ENSCAFG00845008661	10	13	15	11
ENSCAFG00845008660	563	519	553	565
ENSCAFG00845006000	52	59	63	84
ENSCAFG00845008663	39	41	31	41
ENSCAFG00845006001	373	442	320	340
ENSCAFG00845008662	486	403	445	415
ENSCAFG00845006002	0	0	0	0
ENSCAFG00845008665	0	0	0	0
ENSCAFG00845021632	0	0	0	0
ENSCAFG00845006003	42	36	18	25
ENSCAFG00845008664	8	18	7	5
ENSCAFG00845021633	0	0	0	0
ENSCAFG00845006004	459	502	418	521
ENSCAFG00845008667	1042	1001	878	789
ENSCAFG00845021630	0	0	0	0

ENSCAFG00845006005	0	0	0	0
ENSCAFG00845008666	637	758	633	591
ENSCAFG00845021631	23	28	12	25
ENSCAFG00845006006	538	474	509	484
ENSCAFG00845008669	0	0	0	0
ENSCAFG00845006007	1642	1664	1490	1542
ENSCAFG00845008668	0	0	0	0
ENSCAFG00845006008	0	0	0	0
ENSCAFG00845006009	4703	4601	4626	4528
ENSCAFG00845021625	0	0	0	0
ENSCAFG00845021626	275	248	297	268
ENSCAFG00845021623	0	0	0	0
ENSCAFG00845021624	3	5	2	4
ENSCAFG00845021629	2	0	0	0
ENSCAFG00845021627	117	96	121	145
ENSCAFG00845021628	8	4	12	3
ENSCAFG00845008650	2049	2107	1969	1892
ENSCAFG00845008652	0	0	0	2
ENSCAFG00845008651	0	0	0	0
ENSCAFG00845008654	1	2	0	0
ENSCAFG00845021621	0	0	0	0
ENSCAFG00845008653	2026	2057	1931	1832
ENSCAFG00845021622	0	0	0	0
ENSCAFG00845008656	0	0	0	0
ENSCAFG00845008655	0	0	0	0
ENSCAFG00845021620	5	5	3	3
ENSCAFG00845008658	901	800	838	784
ENSCAFG00845008657	0	0	0	0
ENSCAFG00845008659	518	496	481	524
ENSCAFG00845021595	38	38	35	54
ENSCAFG00845021596	6046	6037	6121	6193
ENSCAFG00845021593	35	18	26	22
ENSCAFG00845021594	2	2	0	2
ENSCAFG00845021599	144	139	141	133
ENSCAFG00845021597	2065	2018	2148	2066
ENSCAFG00845021598	0	0	0	0
ENSCAFG00845021591	0	0	0	0
ENSCAFG00845021592	0	0	3	0
ENSCAFG00845021590	0	0	0	0
ENSCAFG00845021584	0	0	0	0
ENSCAFG00845021585	0	0	0	0

ENSCAFG00845021582	0	0	0	0
ENSCAFG00845021583	0	0	0	0
ENSCAFG00845021588	4	3	0	1
ENSCAFG00845021589	0	0	0	0
ENSCAFG00845021586	64	57	46	64
ENSCAFG00845021587	0	0	0	0
ENSCAFG00845021580	618	631	661	721
ENSCAFG00845021581	0	0	3	1
ENSCAFG00845021735	280	315	346	308
ENSCAFG00845021736	0	0	0	0
ENSCAFG00845021733	86	82	80	95
ENSCAFG00845021734	1445	1341	1369	1518
ENSCAFG00845021737	0	0	0	0
ENSCAFG00845021738	0	0	0	0
ENSCAFG00845008760	0	0	0	0
ENSCAFG00845008762	357	324	367	356
ENSCAFG00845006100	0	0	0	0
ENSCAFG00845008761	1214	1154	1256	1233
ENSCAFG00845006101	0	0	0	0
ENSCAFG00845008764	0	0	0	1
ENSCAFG00845021731	0	0	1	0
ENSCAFG00845006102	0	0	0	0
ENSCAFG00845008763	1049	1041	1066	1073
ENSCAFG00845021732	0	0	0	3
ENSCAFG00845006103	135	144	122	158
ENSCAFG00845008766	9	10	8	10
ENSCAFG00845006104	223	220	225	232
ENSCAFG00845008765	1309	1299	1441	1335
ENSCAFG00845021730	0	2	2	2
ENSCAFG00845006105	0	0	2	2
ENSCAFG00845008768	1528	1605	1598	1568
ENSCAFG00845006106	438	360	397	359
ENSCAFG00845008767	117	87	62	85
ENSCAFG00845006107	0	0	0	0
ENSCAFG00845006108	1	0	0	1
ENSCAFG00845008769	0	0	0	0
ENSCAFG00845006109	0	0	0	0
ENSCAFG00845021724	5	3	0	1
ENSCAFG00845021725	0	0	0	0
ENSCAFG00845021722	2	1	1	3
ENSCAFG00845021723	2	0	0	0

ENSCAFG00845021728	0	0	0	0
ENSCAFG00845021729	0	0	0	0
ENSCAFG00845021726	0	0	0	0
ENSCAFG00845021727	0	0	0	0
ENSCAFG00845008751	585	583	554	543
ENSCAFG00845008750	339	347	307	339
ENSCAFG00845008753	2	1	0	1
ENSCAFG00845021720	0	0	0	0
ENSCAFG00845008752	1	2	0	0
ENSCAFG00845021721	0	0	0	0
ENSCAFG00845008755	1	3	2	4
ENSCAFG00845008754	1441	1416	1444	1347
ENSCAFG00845008757	0	0	0	0
ENSCAFG00845008756	0	0	0	0
ENSCAFG00845008759	1944	1925	2017	1983
ENSCAFG00845008758	16	25	6	9
ENSCAFG00845021719	0	1	0	0
ENSCAFG00845021713	0	0	0	0
ENSCAFG00845021714	0	0	0	0
ENSCAFG00845021711	0	0	0	0
ENSCAFG00845021712	262	290	223	302
ENSCAFG00845021717	0	0	0	0
ENSCAFG00845021718	0	0	0	0
ENSCAFG00845021715	0	0	0	0
ENSCAFG00845021716	0	0	0	0
ENSCAFG00845008740	0	0	0	0
ENSCAFG00845008742	1899	1836	1783	1773
ENSCAFG00845008741	255	249	234	275
ENSCAFG00845021710	0	0	0	0
ENSCAFG00845008744	0	0	0	0
ENSCAFG00845008743	942	926	828	806
ENSCAFG00845008746	262	243	271	251
ENSCAFG00845008745	7	4	7	7
ENSCAFG00845008748	670	655	741	700
ENSCAFG00845008747	400	333	357	354
ENSCAFG00845008749	1227	1198	1185	1230
ENSCAFG00845021708	50	43	47	55
ENSCAFG00845021709	1593	1564	1580	1726
ENSCAFG00845021702	0	0	0	0
ENSCAFG00845021703	0	1	0	0
ENSCAFG00845021700	0	0	0	0

ENSCAFG00845021701	0	0	0	0
ENSCAFG00845021706	1	0	0	0
ENSCAFG00845021707	2	0	0	0
ENSCAFG00845021704	0	0	0	0
ENSCAFG00845021705	0	0	0	0
ENSCAFG00845008731	0	0	0	0
ENSCAFG00845008730	0	0	0	0
ENSCAFG00845008733	0	0	0	0
ENSCAFG00845008732	121	132	101	103
ENSCAFG00845008735	864	859	891	937
ENSCAFG00845008734	1	1	4	4
ENSCAFG00845008737	0	0	0	0
ENSCAFG00845008736	0	0	0	0
ENSCAFG00845008739	5	7	7	2
ENSCAFG00845008738	17	25	19	12
ENSCAFG00845008720	384	350	281	299
ENSCAFG00845008722	0	1	0	1
ENSCAFG00845008721	0	0	0	0
ENSCAFG00845008724	0	0	0	0
ENSCAFG00845031105	0	0	0	0
ENSCAFG00845008723	0	0	0	0
ENSCAFG00845031106	226	209	208	238
ENSCAFG00845008726	501	507	568	557
ENSCAFG00845031107	0	0	0	0
ENSCAFG00845008725	23	21	18	24
ENSCAFG00845031108	0	0	2	0
ENSCAFG00845008728	526	559	490	582
ENSCAFG00845031109	587	577	490	483
ENSCAFG00845008727	87	94	64	86
ENSCAFG00845008729	9	6	2	15
ENSCAFG00845031100	1	1	2	2
ENSCAFG00845031101	0	0	0	0
ENSCAFG00845031102	0	0	0	0
ENSCAFG00845031103	117	127	128	120
ENSCAFG00845031104	160	179	184	171
ENSCAFG00845008711	674	641	529	680
ENSCAFG00845008710	675	647	689	692
ENSCAFG00845008713	686	668	837	865
ENSCAFG00845031116	2692	2432	2682	2596
ENSCAFG00845008712	0	0	0	0
ENSCAFG00845031117	0	0	1	0

ENSCAFG00845008715	268	255	210	231
ENSCAFG00845031118	0	0	0	1
ENSCAFG00845008714	0	0	1	0
ENSCAFG00845031119	0	0	0	0
ENSCAFG00845008717	1623	1584	1499	1461
ENSCAFG00845008716	0	0	0	0
ENSCAFG00845008719	373	352	336	306
ENSCAFG00845008718	0	0	0	0
ENSCAFG00845031110	1	0	0	0
ENSCAFG00845031111	0	0	0	0
ENSCAFG00845031112	0	0	0	0
ENSCAFG00845031113	0	0	0	3
ENSCAFG00845031114	0	0	0	0
ENSCAFG00845031115	4	2	2	3
ENSCAFG00845008700	0	0	0	0
ENSCAFG00845008702	2	2	0	0
ENSCAFG00845008701	0	0	0	0
ENSCAFG00845008704	523	485	531	477
ENSCAFG00845008703	594	496	538	522
ENSCAFG00845008706	0	0	0	0
ENSCAFG00845008705	1	3	1	3
ENSCAFG00845008708	43	36	43	39
ENSCAFG00845008707	0	0	0	0
ENSCAFG00845008709	0	0	0	0
ENSCAFG00845031120	0	0	0	0
ENSCAFG00845031121	2614	2479	2654	2680
ENSCAFG00845031122	438	395	510	509
ENSCAFG00845018169	0	0	0	0
ENSCAFG00845006180	121	70	126	95
ENSCAFG00845018168	0	0	0	0
ENSCAFG00845006181	2345	2275	2248	2284
ENSCAFG00845018167	1	1	4	2
ENSCAFG00845006182	0	0	0	0
ENSCAFG00845018166	500	451	422	438
ENSCAFG00845006183	20	27	27	33
ENSCAFG00845018165	0	0	0	0
ENSCAFG00845006184	0	5	1	1
ENSCAFG00845018164	3	1	2	0
ENSCAFG00845006185	483	462	445	465
ENSCAFG00845018163	687	651	721	785
ENSCAFG00845006186	19	42	33	23

ENSCAFG00845018162	309	266	294	222
ENSCAFG00845006187	552	494	631	509
ENSCAFG00845018161	662	680	537	539
ENSCAFG00845006188	39	33	38	40
ENSCAFG00845018160	308	361	329	355
ENSCAFG00845006189	152	161	146	136
ENSCAFG00845018159	0	0	0	0
ENSCAFG00845018158	0	0	0	0
ENSCAFG00845018157	25	29	28	44
ENSCAFG00845006170	0	0	0	0
ENSCAFG00845018156	0	0	0	0
ENSCAFG00845006171	0	0	0	0
ENSCAFG00845018155	0	0	0	0
ENSCAFG00845006172	0	0	1	0
ENSCAFG00845018154	0	0	0	0
ENSCAFG00845006173	0	0	0	0
ENSCAFG00845018153	1026	1006	966	957
ENSCAFG00845006174	0	0	0	0
ENSCAFG00845018152	0	0	0	0
ENSCAFG00845006175	0	0	0	0
ENSCAFG00845018151	18	12	10	16
ENSCAFG00845006176	165	164	185	164
ENSCAFG00845018150	0	0	0	0
ENSCAFG00845006177	738	704	860	801
ENSCAFG00845006178	0	0	0	0
ENSCAFG00845006179	0	0	0	0
ENSCAFG00845018149	607	638	580	653
ENSCAFG00845018148	6	4	3	3
ENSCAFG00845018147	706	764	774	753
ENSCAFG00845021799	0	1	0	0
ENSCAFG00845018146	0	0	0	1
ENSCAFG00845018145	2	2	1	2
ENSCAFG00845006160	6	2	4	2
ENSCAFG00845018144	32	19	26	30
ENSCAFG00845006161	44	55	46	50
ENSCAFG00845018143	0	0	0	0
ENSCAFG00845006162	3560	3405	3564	3569
ENSCAFG00845018142	1008	935	1043	1089
ENSCAFG00845006163	0	0	0	0
ENSCAFG00845018141	45	38	32	48
ENSCAFG00845021793	243	286	257	249

ENSCAFG00845006164	0	0	0	0
ENSCAFG00845021794	1	6	11	2
ENSCAFG00845006165	39235	37608	32827	32070
ENSCAFG00845021791	0	0	0	0
ENSCAFG00845006166	148	127	77	100
ENSCAFG00845021792	16	11	8	20
ENSCAFG00845006167	0	0	0	0
ENSCAFG00845021797	0	1	0	0
ENSCAFG00845006168	0	1	0	0
ENSCAFG00845021798	2	1	0	1
ENSCAFG00845006169	209	147	167	177
ENSCAFG00845021795	0	0	0	0
ENSCAFG00845021796	0	0	0	0
ENSCAFG00845021790	0	0	0	0
ENSCAFG00845018138	0	0	0	0
ENSCAFG00845018137	71	59	59	42
ENSCAFG00845018136	0	0	0	0
ENSCAFG00845021788	0	0	0	0
ENSCAFG00845018135	0	0	0	0
ENSCAFG00845021789	1092	1154	1003	1075
ENSCAFG00845018134	0	0	0	0
ENSCAFG00845018133	0	0	0	0
ENSCAFG00845006150	0	0	0	0
ENSCAFG00845018132	0	0	0	0
ENSCAFG00845006151	2197	2138	2095	2237
ENSCAFG00845018131	206	205	202	205
ENSCAFG00845006152	1719	1707	1519	1479
ENSCAFG00845018130	0	0	0	0
ENSCAFG00845021782	3669	3662	3430	3418
ENSCAFG00845006153	57	34	39	31
ENSCAFG00845021783	0	0	0	0
ENSCAFG00845006154	1345	1299	1407	1390
ENSCAFG00845021780	536	509	481	496
ENSCAFG00845006155	4	3	4	0
ENSCAFG00845021781	0	0	0	0
ENSCAFG00845006156	1761	1623	1571	1559
ENSCAFG00845021786	6822	6604	6512	6937
ENSCAFG00845006157	361	347	318	340
ENSCAFG00845021787	2888	2826	2618	2693
ENSCAFG00845006158	97	85	118	101
ENSCAFG00845021784	17	10	11	19

ENSCAFG00845006159	0	0	0	0
ENSCAFG00845021785	0	0	0	0
ENSCAFG00845018139	129	104	103	114
ENSCAFG00845018127	9	9	13	15
ENSCAFG00845021779	557	562	518	512
ENSCAFG00845018126	125	90	106	85
ENSCAFG00845018125	9	2	4	2
ENSCAFG00845021777	726	603	569	646
ENSCAFG00845018124	1	1	1	1
ENSCAFG00845021778	0	0	0	0
ENSCAFG00845018123	964	980	1065	1075
ENSCAFG00845018122	0	0	0	0
ENSCAFG00845018121	0	0	0	0
ENSCAFG00845006140	3	2	0	0
ENSCAFG00845018120	1	4	6	7
ENSCAFG00845006141	0	1	0	0
ENSCAFG00845021771	12	19	42	15
ENSCAFG00845006142	0	0	0	0
ENSCAFG00845021772	0	0	0	0
ENSCAFG00845006143	0	0	0	0
ENSCAFG00845006144	0	0	0	0
ENSCAFG00845021770	0	0	0	0
ENSCAFG00845006145	105	103	94	126
ENSCAFG00845021775	0	0	0	0
ENSCAFG00845006146	789	779	743	781
ENSCAFG00845021776	2	0	0	0
ENSCAFG00845006147	0	0	0	0
ENSCAFG00845021773	0	0	0	0
ENSCAFG00845006148	259	201	223	194
ENSCAFG00845021774	0	0	0	0
ENSCAFG00845006149	0	0	0	0
ENSCAFG00845018129	2486	2325	2372	2281
ENSCAFG00845018128	271	237	175	225
ENSCAFG00845018116	170	198	170	223
ENSCAFG00845021768	0	0	0	0
ENSCAFG00845018115	1011	1023	877	934
ENSCAFG00845021769	0	0	0	0
ENSCAFG00845018114	0	0	0	0
ENSCAFG00845021766	2	10	4	3
ENSCAFG00845018113	6	10	6	3
ENSCAFG00845021767	0	0	0	0

ENSCAFG00845018112	0	1	0	0
ENSCAFG00845018111	0	0	0	0
ENSCAFG00845008791	0	0	0	0
ENSCAFG00845018110	0	0	0	0
ENSCAFG00845008790	0	0	0	0
ENSCAFG00845006130	1620	1541	1556	1598
ENSCAFG00845008793	0	0	0	0
ENSCAFG00845021760	2037	1858	1987	1863
ENSCAFG00845006131	0	0	0	0
ENSCAFG00845008792	475	439	492	556
ENSCAFG00845021761	9	18	17	10
ENSCAFG00845006132	445	429	465	430
ENSCAFG00845008795	573	546	586	597
ENSCAFG00845006133	0	0	0	0
ENSCAFG00845008794	440	435	446	459
ENSCAFG00845006134	0	0	0	0
ENSCAFG00845008797	0	0	0	0
ENSCAFG00845021764	931	840	917	913
ENSCAFG00845006135	25	25	28	36
ENSCAFG00845008796	0	0	0	0
ENSCAFG00845021765	692	599	699	666
ENSCAFG00845006136	4641	4461	4270	4153
ENSCAFG00845008799	0	0	0	0
ENSCAFG00845021762	861	858	754	718
ENSCAFG00845006137	1	0	0	0
ENSCAFG00845008798	1399	1423	1312	1352
ENSCAFG00845006138	8	9	4	5
ENSCAFG00845006139	3016	2987	2756	2542
ENSCAFG00845018119	0	0	0	0
ENSCAFG00845018118	0	0	0	0
ENSCAFG00845018117	585	626	672	720
ENSCAFG00845018105	0	0	0	0
ENSCAFG00845021757	1	0	0	0
ENSCAFG00845018104	0	0	0	0
ENSCAFG00845021758	0	0	0	0
ENSCAFG00845018103	1	5	6	3
ENSCAFG00845021755	400	413	375	381
ENSCAFG00845018102	353	383	345	451
ENSCAFG00845021756	267	242	251	240
ENSCAFG00845018101	1381	1397	1347	1281
ENSCAFG00845018100	0	0	0	0

ENSCAFG00845008780	0	0	0	0
ENSCAFG00845021759	449	469	498	540
ENSCAFG00845008782	2	0	0	0
ENSCAFG00845006120	0	0	0	0
ENSCAFG00845008781	0	0	0	0
ENSCAFG00845021750	0	0	0	0
ENSCAFG00845006121	4	7	1	1
ENSCAFG00845008784	64	55	38	39
ENSCAFG00845006122	1	1	0	0
ENSCAFG00845008783	574	586	575	537
ENSCAFG00845006123	861	879	881	799
ENSCAFG00845008786	194	208	213	223
ENSCAFG00845021753	0	0	0	0
ENSCAFG00845006124	0	0	0	0
ENSCAFG00845008785	2901	2846	3047	3236
ENSCAFG00845021754	2908	2948	2752	2892
ENSCAFG00845006125	27	23	32	33
ENSCAFG00845008788	0	0	0	0
ENSCAFG00845021751	0	0	0	0
ENSCAFG00845006126	0	1	1	1
ENSCAFG00845008787	2	0	1	9
ENSCAFG00845021752	37	25	17	39
ENSCAFG00845006127	552	531	553	605
ENSCAFG00845006128	0	0	0	0
ENSCAFG00845008789	0	0	0	0
ENSCAFG00845006129	349	403	352	369
ENSCAFG00845018109	0	0	0	2
ENSCAFG00845018108	789	749	707	702
ENSCAFG00845018107	0	0	0	0
ENSCAFG00845018106	1	2	3	1
ENSCAFG00845021746	723	688	654	757
ENSCAFG00845021747	1521	1390	1433	1515
ENSCAFG00845021744	1	6	1	0
ENSCAFG00845021745	782	751	814	699
ENSCAFG00845021748	0	0	0	0
ENSCAFG00845021749	1	2	3	1
ENSCAFG00845008771	9	8	8	5
ENSCAFG00845008770	0	0	0	0
ENSCAFG00845006110	327	276	268	266
ENSCAFG00845008773	356	307	367	386
ENSCAFG00845006111	0	1	0	0

ENSCAFG00845008772	0	0	1	0
ENSCAFG00845006112	476	415	432	468
ENSCAFG00845008775	1	2	3	1
ENSCAFG00845021742	630	615	566	608
ENSCAFG00845006113	11	13	13	10
ENSCAFG00845008774	2386	2319	2252	2312
ENSCAFG00845021743	314	328	237	268
ENSCAFG00845006114	390	384	333	376
ENSCAFG00845008777	0	0	0	0
ENSCAFG00845021740	0	0	0	0
ENSCAFG00845006115	4	7	4	13
ENSCAFG00845008776	0	0	0	0
ENSCAFG00845021741	23	10	27	17
ENSCAFG00845006116	0	0	0	0
ENSCAFG00845008779	155	158	156	201
ENSCAFG00845006117	392	377	216	249
ENSCAFG00845008778	455	454	406	465
ENSCAFG00845006118	46	65	35	51
ENSCAFG00845006119	0	0	0	0
ENSCAFG00845018099	0	0	0	0
ENSCAFG00845018098	454	445	411	423
ENSCAFG00845018097	0	0	0	0
ENSCAFG00845018096	115	95	98	66
ENSCAFG00845018095	304	297	271	267
ENSCAFG00845018094	205	188	190	206
ENSCAFG00845018093	0	0	0	0
ENSCAFG00845018092	0	0	0	0
ENSCAFG00845018091	508	530	535	518
ENSCAFG00845018090	485	483	519	475
ENSCAFG00845018089	3	2	0	1
ENSCAFG00845018088	4	7	5	11
ENSCAFG00845018087	661	611	602	607
ENSCAFG00845018086	0	0	0	0
ENSCAFG00845018085	12	9	12	17
ENSCAFG00845018084	7	12	6	3
ENSCAFG00845018083	37	50	61	64
ENSCAFG00845018082	2	2	1	1
ENSCAFG00845018081	1	0	4	0
ENSCAFG00845018080	0	0	0	0
ENSCAFG00845006090	42	46	58	48
ENSCAFG00845006091	0	1	0	1

ENSCAFG00845006092	0	0	0	1
ENSCAFG00845006093	0	0	0	0
ENSCAFG00845018079	22381	21947	21716	21245
ENSCAFG00845006094	244	237	234	253
ENSCAFG00845018078	1227	1274	1178	1279
ENSCAFG00845006095	650	625	575	663
ENSCAFG00845018077	0	0	0	0
ENSCAFG00845006096	467	463	406	387
ENSCAFG00845018076	0	0	0	0
ENSCAFG00845006097	124	129	111	110
ENSCAFG00845018075	1673	1631	1534	1693
ENSCAFG00845006098	2	2	10	2
ENSCAFG00845018074	1	0	0	0
ENSCAFG00845006099	0	0	0	0
ENSCAFG00845018073	2	0	0	0
ENSCAFG00845018072	3170	2938	3189	3131
ENSCAFG00845018071	0	0	0	0
ENSCAFG00845018070	0	0	0	0
ENSCAFG00845006080	0	0	0	0
ENSCAFG00845006081	0	0	0	0
ENSCAFG00845018069	0	0	0	0
ENSCAFG00845006082	0	0	0	0
ENSCAFG00845018068	3	3	10	14
ENSCAFG00845006083	6589	6832	7034	6684
ENSCAFG00845018067	0	0	0	0
ENSCAFG00845006084	757	726	676	816
ENSCAFG00845018066	2575	2448	2276	2457
ENSCAFG00845006085	4	0	0	1
ENSCAFG00845018065	12697	12197	11673	12424
ENSCAFG00845006086	696	653	659	619
ENSCAFG00845018064	0	0	0	0
ENSCAFG00845006087	0	0	0	0
ENSCAFG00845018063	0	0	0	0
ENSCAFG00845006088	0	0	0	0
ENSCAFG00845018062	47	63	66	60
ENSCAFG00845006089	0	0	0	0
ENSCAFG00845018061	0	0	0	0
ENSCAFG00845018060	20	23	23	13
ENSCAFG00845018059	0	0	0	0
ENSCAFG00845006070	1658	1537	1532	1596
ENSCAFG00845018058	3364	3462	3448	3367

ENSCAFG00845006071	1503	1460	1509	1623
ENSCAFG00845018057	27	18	14	18
ENSCAFG00845006072	1634	1551	1498	1370
ENSCAFG00845006073	4245	3807	4070	4222
ENSCAFG00845018055	22	15	6	6
ENSCAFG00845006074	2	8	2	3
ENSCAFG00845018054	0	0	2	3
ENSCAFG00845006075	6	6	3	6
ENSCAFG00845018053	0	0	0	0
ENSCAFG00845006076	1304	1277	658	655
ENSCAFG00845018052	8	5	6	7
ENSCAFG00845006077	2	2	0	1
ENSCAFG00845018051	2	0	1	0
ENSCAFG00845006078	0	2	0	0
ENSCAFG00845018050	0	0	0	0
ENSCAFG00845006079	193	160	166	178
ENSCAFG00845031028	0	0	0	0
ENSCAFG00845031029	0	0	0	0
ENSCAFG00845031020	489	474	332	381
ENSCAFG00845031021	0	0	0	0
ENSCAFG00845031022	1123	1010	870	904
ENSCAFG00845031023	0	0	0	0
ENSCAFG00845031024	0	0	0	0
ENSCAFG00845031025	1497	1554	1360	1370
ENSCAFG00845031026	0	0	0	0
ENSCAFG00845031027	3	1	1	0
ENSCAFG00845031030	453	489	450	453
ENSCAFG00845031039	0	5	0	0
ENSCAFG00845031031	1053	1020	1030	995
ENSCAFG00845031032	0	6	1	4
ENSCAFG00845031033	293	307	345	299
ENSCAFG00845031034	829	782	855	816
ENSCAFG00845031035	0	0	1	0
ENSCAFG00845031036	391	343	435	433
ENSCAFG00845031037	245	233	241	237
ENSCAFG00845031038	389	360	356	379
ENSCAFG00845031040	84	111	118	99
ENSCAFG00845031041	408	380	376	361
ENSCAFG00845031042	0	0	0	0
ENSCAFG00845031043	517	442	482	486
ENSCAFG00845031044	528	521	539	497

ENSCAFG00845031045	0	0	0	1
ENSCAFG00845031046	1	2	3	2
ENSCAFG00845031047	2	2	3	3
ENSCAFG00845031048	0	1	1	1
ENSCAFG00845031049	364	325	356	382
ENSCAFG00845031050	0	0	0	0
ENSCAFG00845031051	7	11	8	13
ENSCAFG00845031052	291	305	287	285
ENSCAFG00845031053	0	0	2	0
ENSCAFG00845031054	1114	1093	1188	1248
ENSCAFG00845031055	3	1	0	0
ENSCAFG00845031056	9	7	5	6
ENSCAFG00845031057	64	67	56	57
ENSCAFG00845031058	0	0	0	0
ENSCAFG00845031059	0	0	0	0
ENSCAFG00845031060	790	694	842	839
ENSCAFG00845031061	723	662	752	770
ENSCAFG00845031062	118	124	122	118
ENSCAFG00845031063	0	0	0	0
ENSCAFG00845031064	75	58	74	69
ENSCAFG00845031065	0	0	0	0
ENSCAFG00845031066	0	0	0	0
ENSCAFG00845031067	0	0	0	0
ENSCAFG00845031068	507	477	470	426
ENSCAFG00845031069	0	0	0	0
ENSCAFG00845031070	0	0	0	2
ENSCAFG00845031071	741	847	816	813
ENSCAFG00845031072	0	0	0	0
ENSCAFG00845031073	150	146	134	149
ENSCAFG00845031074	256	215	235	210
ENSCAFG00845031075	0	0	0	0
ENSCAFG00845031076	25	34	17	14
ENSCAFG00845031077	0	0	4	0
ENSCAFG00845031078	0	0	0	0
ENSCAFG00845031079	884	795	891	896
ENSCAFG00845031080	0	0	0	1
ENSCAFG00845031081	0	0	0	0
ENSCAFG00845031082	0	0	0	0
ENSCAFG00845031083	120	91	75	43
ENSCAFG00845031084	0	0	0	0
ENSCAFG00845031085	0	0	2	0

ENSCAFG00845031086	356	342	313	348
ENSCAFG00845031087	497	496	526	539
ENSCAFG00845031088	12	17	12	20
ENSCAFG00845031089	24	16	5	9
ENSCAFG00845031090	17	9	10	11
ENSCAFG00845031091	4	0	0	4
ENSCAFG00845031092	2	6	7	14
ENSCAFG00845031093	0	0	0	0
ENSCAFG00845031094	17	23	3	9
ENSCAFG00845031095	0	0	0	0
ENSCAFG00845031096	0	0	0	0
ENSCAFG00845031097	1455	1346	1365	1435
ENSCAFG00845031098	1314	1333	980	1095
ENSCAFG00845031099	3	4	3	1
ENSCAFG00845023639	1	0	0	0
ENSCAFG00845023638	884	861	932	852
ENSCAFG00845023637	0	0	0	0
ENSCAFG00845023636	595	488	515	495
ENSCAFG00845008001	0	0	0	0
ENSCAFG00845023631	720	683	743	707
ENSCAFG00845008000	1582	1463	1723	1783
ENSCAFG00845023630	16382	15947	16467	16308
ENSCAFG00845008003	0	0	0	0
ENSCAFG00845008002	9	3	7	8
ENSCAFG00845008005	0	0	0	0
ENSCAFG00845023635	468	517	475	463
ENSCAFG00845008004	920	898	1200	1311
ENSCAFG00845023634	16	12	12	11
ENSCAFG00845008007	0	0	0	0
ENSCAFG00845023633	0	0	0	0
ENSCAFG00845008006	230	235	220	227
ENSCAFG00845023632	0	0	0	1
ENSCAFG00845008009	1030	1083	1079	1032
ENSCAFG00845008008	21	17	18	28
ENSCAFG00845011657	0	0	0	0
ENSCAFG00845011658	133	124	160	163
ENSCAFG00845011659	4	0	1	1
ENSCAFG00845011653	277	315	374	390
ENSCAFG00845011654	0	0	1	0
ENSCAFG00845011655	1106	1165	1127	1135
ENSCAFG00845011656	661	659	771	744

ENSCAFG00845011650	0	0	0	0
ENSCAFG00845011651	0	0	0	0
ENSCAFG00845011652	1148	1067	1062	1057
ENSCAFG00845023628	0	0	0	0
ENSCAFG00845023627	21	26	15	14
ENSCAFG00845023626	1082	906	845	877
ENSCAFG00845023625	0	0	0	0
ENSCAFG00845023629	0	0	0	0
ENSCAFG00845023620	849	722	888	966
ENSCAFG00845023624	0	0	0	0
ENSCAFG00845023623	121	105	108	126
ENSCAFG00845023622	0	0	0	0
ENSCAFG00845023621	0	0	0	0
ENSCAFG00845011646	611	610	670	592
ENSCAFG00845011647	514	491	413	459
ENSCAFG00845011648	0	0	2	0
ENSCAFG00845011649	8	9	23	17
ENSCAFG00845011642	5181	4937	4882	5161
ENSCAFG00845011643	19	17	10	7
ENSCAFG00845011644	0	0	0	0
ENSCAFG00845011645	0	0	0	0
ENSCAFG00845011640	1	1	1	4
ENSCAFG00845011641	1518	1469	1435	1554
ENSCAFG00845023617	0	0	0	0
ENSCAFG00845023616	405	424	415	418
ENSCAFG00845023614	0	0	0	0
ENSCAFG00845023619	4	5	1	2
ENSCAFG00845023618	495	476	592	596
ENSCAFG00845023613	0	0	0	0
ENSCAFG00845023612	0	0	0	0
ENSCAFG00845023611	319	339	395	431
ENSCAFG00845023610	12958	12969	12640	12306
ENSCAFG00845011639	375	347	249	273
ENSCAFG00845011635	42	33	29	57
ENSCAFG00845011636	0	0	0	0
ENSCAFG00845011637	7	3	7	7
ENSCAFG00845011638	654	678	639	656
ENSCAFG00845011631	0	0	0	0
ENSCAFG00845011632	519	448	433	406
ENSCAFG00845011633	0	0	0	0
ENSCAFG00845011634	1171	1212	1155	1150

ENSCAFG00845011630	95	87	95	119
ENSCAFG00845023606	2621	2536	2374	2367
ENSCAFG00845023605	0	1	0	2
ENSCAFG00845023604	10	6	17	12
ENSCAFG00845023603	137	113	129	144
ENSCAFG00845023609	2	0	1	0
ENSCAFG00845023608	5	5	5	10
ENSCAFG00845023607	73	97	92	119
ENSCAFG00845023602	3	6	7	5
ENSCAFG00845023601	4984	4777	4844	4747
ENSCAFG00845023600	0	1	0	0
ENSCAFG00845011628	93	76	29	40
ENSCAFG00845011629	10	10	3	5
ENSCAFG00845011624	80	109	134	190
ENSCAFG00845011625	0	0	0	0
ENSCAFG00845011626	25	36	36	45
ENSCAFG00845011627	0	0	0	0
ENSCAFG00845011620	0	0	0	0
ENSCAFG00845011621	0	0	0	0
ENSCAFG00845011622	0	1	0	0
ENSCAFG00845011623	0	0	0	0
ENSCAFG00845011617	1	1	3	2
ENSCAFG00845011618	0	0	0	0
ENSCAFG00845011619	2712	2610	2604	2516
ENSCAFG00845011613	0	0	0	1
ENSCAFG00845011614	0	0	0	0
ENSCAFG00845011615	0	0	0	0
ENSCAFG00845011616	2	2	4	1
ENSCAFG00845011610	0	0	0	0
ENSCAFG00845011611	1200	1164	979	1052
ENSCAFG00845011612	0	0	0	0
ENSCAFG00845011606	0	0	0	0
ENSCAFG00845011607	122	108	90	93
ENSCAFG00845011608	0	1	1	1
ENSCAFG00845011609	0	5	0	0
ENSCAFG00845011602	1353	1338	1157	1257
ENSCAFG00845011603	0	0	2	0
ENSCAFG00845011604	0	0	0	0
ENSCAFG00845011605	0	0	0	0
ENSCAFG00845011600	11955	11380	11677	12579
ENSCAFG00845011601	303	277	289	284

ENSCAFG00845023598	14	16	5	6
ENSCAFG00845023597	305	301	350	338
ENSCAFG00845023596	0	0	0	0
ENSCAFG00845023599	192	203	151	209
ENSCAFG00845023590	390	312	388	488
ENSCAFG00845023594	0	0	0	0
ENSCAFG00845023593	846	717	766	745
ENSCAFG00845023592	0	0	0	0
ENSCAFG00845023591	0	0	0	0
ENSCAFG00845023587	166	152	151	159
ENSCAFG00845023586	184	168	203	154
ENSCAFG00845023585	3	0	4	5
ENSCAFG00845023584	0	0	0	0
ENSCAFG00845023589	425	403	476	492
ENSCAFG00845023588	135	115	140	161
ENSCAFG00845023583	0	0	0	0
ENSCAFG00845023582	2	1	3	5
ENSCAFG00845023581	30	22	15	27
ENSCAFG00845023580	2	5	6	5
ENSCAFG00845011590	152	132	162	160
ENSCAFG00845011591	1	1	2	5
ENSCAFG00845011592	0	0	0	0
ENSCAFG00845011593	0	0	0	0
ENSCAFG00845023576	798	893	736	773
ENSCAFG00845023575	8	9	4	4
ENSCAFG00845023574	29	28	8	11
ENSCAFG00845023573	196	182	162	168
ENSCAFG00845023579	1205	1176	1049	910
ENSCAFG00845023578	356	377	317	306
ENSCAFG00845023577	0	0	0	0
ENSCAFG00845023571	1	1	0	0
ENSCAFG00845023570	2651	2476	2592	2669
ENSCAFG00845011598	415	380	390	404
ENSCAFG00845011599	42	37	24	37
ENSCAFG00845011594	15	37	15	15
ENSCAFG00845011595	0	0	0	0
ENSCAFG00845011596	0	0	0	0
ENSCAFG00845011597	1658	1642	1613	1497
ENSCAFG00845011580	1	2	0	2
ENSCAFG00845011581	414	337	321	325
ENSCAFG00845011582	1400	1328	1191	1281

ENSCAFG00845023565	283	309	267	326
ENSCAFG00845023564	3280	3132	3274	3763
ENSCAFG00845023563	0	4	3	3
ENSCAFG00845023569	681	692	586	620
ENSCAFG00845023568	2	0	0	0
ENSCAFG00845023567	3	6	1	1
ENSCAFG00845023566	2183	2011	1841	1731
ENSCAFG00845023561	3	0	0	0
ENSCAFG00845023560	242	247	221	242
ENSCAFG00845011587	0	0	0	0
ENSCAFG00845011588	0	0	0	0
ENSCAFG00845011589	312	281	318	327
ENSCAFG00845011583	1110	962	864	857
ENSCAFG00845011584	61	58	84	75
ENSCAFG00845011585	104	108	91	87
ENSCAFG00845011586	718	740	698	753
ENSCAFG00845011570	0	0	1	2
ENSCAFG00845011571	1	2	0	0
ENSCAFG00845023559	55	32	33	52
ENSCAFG00845023554	0	4	2	1
ENSCAFG00845023553	0	0	0	0
ENSCAFG00845023552	0	0	0	0
ENSCAFG00845023551	0	2	5	5
ENSCAFG00845023558	0	0	0	0
ENSCAFG00845023557	1433	1313	1383	1333
ENSCAFG00845023556	14	22	9	22
ENSCAFG00845023550	208	176	162	157
ENSCAFG00845011576	0	0	1	0
ENSCAFG00845011577	1017	1035	1005	993
ENSCAFG00845011578	630	578	579	641
ENSCAFG00845011579	1902	1902	2037	2045
ENSCAFG00845011572	899	813	953	886
ENSCAFG00845011573	14	0	4	0
ENSCAFG00845011574	4463	4268	4135	4276
ENSCAFG00845011575	0	0	0	0
ENSCAFG00845023549	111	100	100	85
ENSCAFG00845011560	9	7	8	9
ENSCAFG00845023548	1	3	3	3
ENSCAFG00845023543	2	2	3	0
ENSCAFG00845023542	0	0	0	0
ENSCAFG00845023541	8	2	4	6

ENSCAFG00845023540	2198	2054	1905	2031
ENSCAFG00845023547	1920	1858	1787	1802
ENSCAFG00845023546	14686	13481	14906	15525
ENSCAFG00845023545	1467	1393	1436	1408
ENSCAFG00845023544	47	32	46	45
ENSCAFG00845011569	102	78	107	134
ENSCAFG00845011565	335	357	209	188
ENSCAFG00845011566	0	0	0	0
ENSCAFG00845011567	888	882	821	802
ENSCAFG00845011568	1049	987	886	1006
ENSCAFG00845011561	0	0	0	0
ENSCAFG00845011562	246	227	184	180
ENSCAFG00845011563	199	192	160	158
ENSCAFG00845011564	2	11	2	2
ENSCAFG00845023539	226	254	224	237
ENSCAFG00845023538	3	0	1	0
ENSCAFG00845023537	342	263	250	244
ENSCAFG00845023532	396	404	329	394
ENSCAFG00845023531	924	875	881	872
ENSCAFG00845023530	160	156	177	166
ENSCAFG00845023536	17	21	18	9
ENSCAFG00845023535	0	0	0	0
ENSCAFG00845023534	682	682	775	676
ENSCAFG00845023533	0	0	0	0
ENSCAFG00845011558	0	1	1	0
ENSCAFG00845011559	0	0	0	1
ENSCAFG00845011554	0	2	5	0
ENSCAFG00845011555	3	4	2	2
ENSCAFG00845011556	3041	2985	3102	3280
ENSCAFG00845011557	1121	1114	1200	1278
ENSCAFG00845011550	1	0	0	5
ENSCAFG00845011551	0	0	0	0
ENSCAFG00845011552	0	0	0	0
ENSCAFG00845011553	5924	5358	5774	5927
ENSCAFG00845023529	7	3	6	2
ENSCAFG00845023528	9	7	5	5
ENSCAFG00845023527	649	632	610	553
ENSCAFG00845023526	2	2	1	2
ENSCAFG00845023521	0	0	0	0
ENSCAFG00845023520	0	0	0	0
ENSCAFG00845023525	139	142	150	154

ENSCAFG00845023524	704	656	650	765
ENSCAFG00845023523	0	0	0	0
ENSCAFG00845011547	192	151	153	173
ENSCAFG00845011548	968	900	876	878
ENSCAFG00845011549	1	0	0	0
ENSCAFG00845011543	165	146	144	177
ENSCAFG00845011544	6	4	5	1
ENSCAFG00845011545	0	0	1	1
ENSCAFG00845011546	66	73	62	89
ENSCAFG00845011540	483	415	518	536
ENSCAFG00845011541	212	227	324	293
ENSCAFG00845011542	9	16	5	7
ENSCAFG00845023759	0	1	0	0
ENSCAFG00845023758	28	30	30	22
ENSCAFG00845023757	10746	10264	10813	11559
ENSCAFG00845008120	8	6	5	2
ENSCAFG00845008122	0	0	0	1
ENSCAFG00845023752	0	0	0	0
ENSCAFG00845008121	7	7	12	9
ENSCAFG00845023751	426	449	450	458
ENSCAFG00845008124	698	657	618	614
ENSCAFG00845023750	0	0	0	0
ENSCAFG00845008123	0	0	1	0
ENSCAFG00845008126	0	0	0	0
ENSCAFG00845023756	1	0	0	0
ENSCAFG00845008125	49	39	32	34
ENSCAFG00845023755	0	0	0	0
ENSCAFG00845008128	9	9	4	6
ENSCAFG00845023754	20	18	27	20
ENSCAFG00845008127	0	3	0	0
ENSCAFG00845023753	1	0	1	1
ENSCAFG00845008129	274	285	282	272
ENSCAFG00845011778	0	0	0	0
ENSCAFG00845011779	0	0	0	0
ENSCAFG00845011774	0	2	0	1
ENSCAFG00845011775	13	13	14	27
ENSCAFG00845011776	4	9	4	6
ENSCAFG00845011777	0	0	0	0
ENSCAFG00845011770	25	40	32	25
ENSCAFG00845011771	235	237	268	287
ENSCAFG00845011772	5	5	5	9

ENSCAFG00845011773	15	7	14	13
ENSCAFG00845023749	17	19	32	22
ENSCAFG00845023748	1339	1255	978	1054
ENSCAFG00845023747	218	201	216	253
ENSCAFG00845023746	13	14	6	19
ENSCAFG00845008111	13	17	13	12
ENSCAFG00845023741	693	700	694	595
ENSCAFG00845008110	0	0	0	2
ENSCAFG00845023740	0	0	0	0
ENSCAFG00845008113	0	0	0	0
ENSCAFG00845008112	1896	1908	1788	1754
ENSCAFG00845008115	0	0	0	0
ENSCAFG00845023745	0	0	0	0
ENSCAFG00845008114	0	0	0	0
ENSCAFG00845023744	493	434	461	401
ENSCAFG00845008117	47	46	33	70
ENSCAFG00845023743	0	0	0	0
ENSCAFG00845008116	4	0	3	3
ENSCAFG00845023742	0	0	0	0
ENSCAFG00845008119	76	55	75	76
ENSCAFG00845008118	0	0	0	1
ENSCAFG00845011767	183	183	179	212
ENSCAFG00845011768	0	0	0	0
ENSCAFG00845011769	0	1	0	0
ENSCAFG00845011763	0	0	0	0
ENSCAFG00845011764	20	30	19	16
ENSCAFG00845011765	0	0	0	0
ENSCAFG00845011766	0	0	0	0
ENSCAFG00845011760	0	0	0	0
ENSCAFG00845011761	120	95	115	118
ENSCAFG00845011762	823	781	759	697
ENSCAFG00845023738	0	0	0	0
ENSCAFG00845023737	740	679	633	685
ENSCAFG00845023736	247	236	270	275
ENSCAFG00845023735	0	0	0	0
ENSCAFG00845023739	259	226	243	232
ENSCAFG00845008100	9	2	6	11
ENSCAFG00845023730	6	1	0	3
ENSCAFG00845008102	0	0	0	0
ENSCAFG00845008101	282	280	250	303
ENSCAFG00845008104	0	0	0	2

ENSCAFG00845023734	0	0	0	0
ENSCAFG00845008103	0	0	1	0
ENSCAFG00845023733	516	534	504	412
ENSCAFG00845008106	0	0	0	0
ENSCAFG00845023732	0	0	0	0
ENSCAFG00845008105	5	5	17	10
ENSCAFG00845023731	574	606	480	406
ENSCAFG00845008108	0	0	1	0
ENSCAFG00845008107	0	0	0	0
ENSCAFG00845008109	0	0	0	0
ENSCAFG00845011756	2687	2673	2368	2371
ENSCAFG00845011757	78	109	60	54
ENSCAFG00845011758	0	0	0	0
ENSCAFG00845011759	0	0	0	0
ENSCAFG00845011752	1119	1039	1051	1043
ENSCAFG00845011753	32	60	48	50
ENSCAFG00845011754	1353	1273	1242	1306
ENSCAFG00845011755	2162	1985	2193	2303
ENSCAFG00845011750	0	0	0	0
ENSCAFG00845011751	1996	1779	1856	1809
ENSCAFG00845023727	0	1	5	0
ENSCAFG00845023726	1	3	3	5
ENSCAFG00845023725	656	542	624	594
ENSCAFG00845023724	0	0	0	0
ENSCAFG00845023729	1380	1238	1434	1385
ENSCAFG00845023728	0	0	0	0
ENSCAFG00845023723	0	1	0	0
ENSCAFG00845023722	235	168	222	213
ENSCAFG00845023721	0	0	0	0
ENSCAFG00845023720	0	0	0	0
ENSCAFG00845011749	0	0	0	0
ENSCAFG00845011745	2	1	3	1
ENSCAFG00845011746	446	390	399	405
ENSCAFG00845011747	594	656	664	700
ENSCAFG00845011748	642	638	590	564
ENSCAFG00845011741	34	37	34	21
ENSCAFG00845011742	1838	1827	1724	1884
ENSCAFG00845011743	0	0	0	0
ENSCAFG00845011744	0	0	0	0
ENSCAFG00845011740	2725	2625	2670	2643
ENSCAFG00845023716	0	1	0	0

ENSCAFG00845023715	258	197	234	227
ENSCAFG00845023714	2	4	1	7
ENSCAFG00845023713	299	320	315	413
ENSCAFG00845023719	776	778	797	817
ENSCAFG00845023718	0	0	0	0
ENSCAFG00845023717	0	0	0	0
ENSCAFG00845023712	0	0	0	0
ENSCAFG00845023711	0	0	0	0
ENSCAFG00845023710	45	37	34	42
ENSCAFG00845011738	385	394	421	346
ENSCAFG00845011739	212	194	231	206
ENSCAFG00845011734	670	701	628	712
ENSCAFG00845011735	2355	2195	1874	1835
ENSCAFG00845011736	41	47	90	69
ENSCAFG00845011737	4	0	1	0
ENSCAFG00845011730	254	272	227	232
ENSCAFG00845011731	173	178	158	165
ENSCAFG00845011732	21	13	22	15
ENSCAFG00845011733	774	794	745	672
ENSCAFG00845023705	0	0	0	0
ENSCAFG00845023704	0	0	0	0
ENSCAFG00845023703	0	0	0	0
ENSCAFG00845023702	510	411	450	442
ENSCAFG00845023709	291	325	306	252
ENSCAFG00845023708	0	0	0	0
ENSCAFG00845023707	56	60	50	51
ENSCAFG00845023706	379	364	345	362
ENSCAFG00845023701	0	0	0	0
ENSCAFG00845023700	0	0	0	0
ENSCAFG00845011727	259	266	235	233
ENSCAFG00845011728	1511	1438	1551	1526
ENSCAFG00845011729	111	83	94	99
ENSCAFG00845011723	15	14	22	9
ENSCAFG00845011724	261	229	288	234
ENSCAFG00845011725	8	9	12	5
ENSCAFG00845011726	26	30	35	17
ENSCAFG00845011720	0	0	0	0
ENSCAFG00845011721	261	217	262	274
ENSCAFG00845011722	3786	3748	3691	3624
ENSCAFG00845011709	657	551	702	695
ENSCAFG00845011716	6	10	11	2

ENSCAFG00845011717	386	371	310	291
ENSCAFG00845011718	0	0	0	0
ENSCAFG00845011719	5885	5942	4451	4469
ENSCAFG00845011712	492	458	464	481
ENSCAFG00845011713	337	314	337	360
ENSCAFG00845011714	1	1	0	1
ENSCAFG00845011715	0	0	0	0
ENSCAFG00845011710	0	0	0	1
ENSCAFG00845011711	0	0	0	0
ENSCAFG00845011705	0	0	0	2
ENSCAFG00845011706	503	448	338	326
ENSCAFG00845011707	822	780	901	883
ENSCAFG00845011708	168	159	154	195
ENSCAFG00845011701	0	0	0	0
ENSCAFG00845011702	0	0	0	0
ENSCAFG00845011703	57	50	78	89
ENSCAFG00845011704	0	0	0	0
ENSCAFG00845011700	243	266	270	257
ENSCAFG00845008081	0	0	0	0
ENSCAFG00845008080	159	130	152	148
ENSCAFG00845008083	72	95	83	90
ENSCAFG00845008082	0	0	0	0
ENSCAFG00845008085	29	28	38	33
ENSCAFG00845008084	0	0	0	0
ENSCAFG00845008087	0	0	2	0
ENSCAFG00845008086	0	0	0	0
ENSCAFG00845008089	813	780	676	709
ENSCAFG00845021056	0	0	0	0
ENSCAFG00845008088	1	1	2	0
ENSCAFG00845021057	39	34	32	18
ENSCAFG00845021054	0	0	0	0
ENSCAFG00845021055	432	392	376	382
ENSCAFG00845021058	0	0	0	0
ENSCAFG00845021059	0	0	0	0
ENSCAFG00845021052	506	476	446	411
ENSCAFG00845021053	0	0	0	0
ENSCAFG00845021050	40	44	53	47
ENSCAFG00845021051	0	0	1	2
ENSCAFG00845008090	2003	1656	1805	1813
ENSCAFG00845008070	0	0	0	0
ENSCAFG00845008072	583	583	642	665

ENSCAFG00845008071	0	0	0	0
ENSCAFG00845008074	0	2	0	0
ENSCAFG00845008073	408	373	343	355
ENSCAFG00845008076	678	649	621	664
ENSCAFG00845008075	0	0	0	0
ENSCAFG00845008078	72	64	56	69
ENSCAFG00845021045	0	0	0	0
ENSCAFG00845008077	38	39	63	43
ENSCAFG00845021046	4	11	2	9
ENSCAFG00845021043	105	103	124	120
ENSCAFG00845008079	0	0	0	0
ENSCAFG00845021044	0	0	0	0
ENSCAFG00845021049	0	0	0	0
ENSCAFG00845021047	0	0	0	0
ENSCAFG00845021048	0	0	0	0
ENSCAFG00845021041	5	1	2	7
ENSCAFG00845021042	0	0	0	0
ENSCAFG00845021040	0	0	0	0
ENSCAFG00845008061	0	0	0	0
ENSCAFG00845008060	0	0	0	0
ENSCAFG00845008063	0	0	0	0
ENSCAFG00845008062	1	0	1	1
ENSCAFG00845008065	0	0	0	0
ENSCAFG00845008064	2496	2347	2270	2449
ENSCAFG00845008067	0	1	2	5
ENSCAFG00845021034	1	0	0	0
ENSCAFG00845023697	1234	1111	1251	1334
ENSCAFG00845008066	322	285	241	343
ENSCAFG00845021035	3	3	1	0
ENSCAFG00845023696	0	0	0	0
ENSCAFG00845008069	0	0	0	0
ENSCAFG00845021032	0	0	0	0
ENSCAFG00845023695	0	0	0	0
ENSCAFG00845008068	1386	1351	1493	1592
ENSCAFG00845021033	200	198	198	184
ENSCAFG00845023694	1	0	0	0
ENSCAFG00845021038	0	3	1	1
ENSCAFG00845021039	0	0	0	0
ENSCAFG00845021036	1330	1366	1336	1289
ENSCAFG00845023699	910	898	769	808
ENSCAFG00845021037	34	35	49	41

ENSCAFG00845023698	0	0	0	0
ENSCAFG00845021030	0	0	0	0
ENSCAFG00845021031	545	440	418	418
ENSCAFG00845023692	6610	6373	6276	6421
ENSCAFG00845023691	0	0	0	0
ENSCAFG00845023690	0	0	0	0
ENSCAFG00845008050	1	2	3	1
ENSCAFG00845021029	1	0	0	1
ENSCAFG00845008052	3238	2965	3626	3663
ENSCAFG00845008051	1004	900	958	1015
ENSCAFG00845008054	1063	1001	1043	1105
ENSCAFG00845008053	159	157	141	150
ENSCAFG00845008056	1762	1729	1831	1948
ENSCAFG00845021023	9	5	2	12
ENSCAFG00845023686	14	10	15	10
ENSCAFG00845008055	0	2	0	0
ENSCAFG00845021024	0	0	0	0
ENSCAFG00845023685	23	15	23	26
ENSCAFG00845008058	8	2	2	0
ENSCAFG00845021021	0	0	0	0
ENSCAFG00845023684	340	367	352	324
ENSCAFG00845008057	0	0	0	0
ENSCAFG00845021022	0	0	0	0
ENSCAFG00845023683	34	36	24	22
ENSCAFG00845021027	7	12	55	51
ENSCAFG00845008059	1	0	2	3
ENSCAFG00845021028	0	0	0	0
ENSCAFG00845023689	0	0	2	2
ENSCAFG00845021025	340	340	364	435
ENSCAFG00845023688	0	0	0	0
ENSCAFG00845021026	349	295	332	307
ENSCAFG00845023687	0	0	0	0
ENSCAFG00845023682	619	638	626	629
ENSCAFG00845021020	937	865	925	934
ENSCAFG00845023681	7	4	3	4
ENSCAFG00845023680	0	0	0	0
ENSCAFG00845011690	1	3	3	3
ENSCAFG00845011691	0	0	0	0
ENSCAFG00845021018	0	0	0	0
ENSCAFG00845011692	0	0	0	0
ENSCAFG00845021019	0	0	0	0

ENSCAFG00845008041	97	70	104	98
ENSCAFG00845008040	413	414	443	498
ENSCAFG00845008043	945	1005	948	962
ENSCAFG00845008042	0	0	3	0
ENSCAFG00845008045	0	0	1	0
ENSCAFG00845021012	727	758	738	713
ENSCAFG00845008044	1385	1392	1227	1289
ENSCAFG00845021013	1913	1908	1905	1995
ENSCAFG00845023674	324	309	297	298
ENSCAFG00845008047	0	0	0	0
ENSCAFG00845021010	1240	1186	1151	1120
ENSCAFG00845023673	1084	1002	906	930
ENSCAFG00845008046	1004	891	884	844
ENSCAFG00845021011	4	0	2	9
ENSCAFG00845023672	23	16	25	15
ENSCAFG00845008049	0	0	0	0
ENSCAFG00845021016	92	85	91	77
ENSCAFG00845023679	13	5	8	8
ENSCAFG00845008048	0	0	0	0
ENSCAFG00845021017	0	0	0	0
ENSCAFG00845021014	0	0	0	0
ENSCAFG00845023677	364	366	414	436
ENSCAFG00845021015	55	49	200	168
ENSCAFG00845023676	0	0	0	0
ENSCAFG00845023671	5	3	11	2
ENSCAFG00845023670	18	15	21	24
ENSCAFG00845011697	0	0	0	0
ENSCAFG00845011698	0	0	0	0
ENSCAFG00845011699	0	3	1	4
ENSCAFG00845011693	55	47	61	42
ENSCAFG00845011694	0	0	1	0
ENSCAFG00845011695	641	705	652	652
ENSCAFG00845011696	9	3	0	4
ENSCAFG00845021009	0	1	1	0
ENSCAFG00845011680	522	588	505	561
ENSCAFG00845021007	42	31	18	24
ENSCAFG00845011681	0	0	0	0
ENSCAFG00845021008	4	12	5	8
ENSCAFG00845023669	0	1	1	0
ENSCAFG00845008030	15480	15205	15560	16482
ENSCAFG00845008032	692	727	665	698

ENSCAFG00845008031	0	1	0	0
ENSCAFG00845008034	3	4	5	2
ENSCAFG00845021001	6	4	1	5
ENSCAFG00845023664	6	7	8	6
ENSCAFG00845008033	336	333	318	375
ENSCAFG00845021002	658	603	621	647
ENSCAFG00845023663	5	8	8	7
ENSCAFG00845008036	23	27	37	25
ENSCAFG00845023662	125	126	125	109
ENSCAFG00845008035	1560	1483	1438	1475
ENSCAFG00845021000	0	0	0	0
ENSCAFG00845023661	0	0	2	0
ENSCAFG00845008038	0	0	0	0
ENSCAFG00845021005	0	0	0	0
ENSCAFG00845023668	3	2	4	9
ENSCAFG00845008037	1	0	0	0
ENSCAFG00845021006	0	0	0	0
ENSCAFG00845023667	0	0	0	0
ENSCAFG00845021003	0	0	0	0
ENSCAFG00845023666	0	0	0	0
ENSCAFG00845008039	285	194	225	270
ENSCAFG00845021004	0	0	0	0
ENSCAFG00845023665	0	0	0	0
ENSCAFG00845023660	3	5	5	9
ENSCAFG00845011686	0	0	0	0
ENSCAFG00845011687	185	143	204	214
ENSCAFG00845011688	191	179	150	160
ENSCAFG00845011689	143	131	116	95
ENSCAFG00845011682	103	111	92	85
ENSCAFG00845011683	11	16	13	11
ENSCAFG00845011684	30	36	10	19
ENSCAFG00845011685	8	12	9	4
ENSCAFG00845023659	0	0	0	2
ENSCAFG00845011670	1	0	2	0
ENSCAFG00845023658	6	2	4	0
ENSCAFG00845008021	50	59	48	50
ENSCAFG00845008020	4	4	2	4
ENSCAFG00845008023	294	301	247	238
ENSCAFG00845023653	3	2	1	4
ENSCAFG00845008022	288	297	265	229
ENSCAFG00845023652	1375	1343	1416	1433

ENSCAFG00845008025	1389	1303	1397	1370
ENSCAFG00845023651	11	15	11	18
ENSCAFG00845008024	0	0	0	0
ENSCAFG00845023650	0	1	3	0
ENSCAFG00845008027	0	0	0	0
ENSCAFG00845023657	48	39	43	55
ENSCAFG00845008026	1	0	0	0
ENSCAFG00845023656	175	177	160	192
ENSCAFG00845008029	0	0	0	0
ENSCAFG00845023655	361	363	366	362
ENSCAFG00845008028	0	1	4	0
ENSCAFG00845023654	685	657	656	657
ENSCAFG00845011679	64	61	80	61
ENSCAFG00845011675	0	0	0	0
ENSCAFG00845011676	0	0	0	0
ENSCAFG00845011677	998	992	1016	971
ENSCAFG00845011678	0	0	0	0
ENSCAFG00845011671	99	83	98	68
ENSCAFG00845011672	1	0	0	0
ENSCAFG00845011673	28	28	29	37
ENSCAFG00845011674	53	47	49	40
ENSCAFG00845023649	201	155	144	179
ENSCAFG00845023647	210	143	229	226
ENSCAFG00845008010	0	0	0	0
ENSCAFG00845008012	32	28	51	38
ENSCAFG00845023642	123	129	105	96
ENSCAFG00845008011	1	0	0	0
ENSCAFG00845023641	3	10	8	8
ENSCAFG00845008014	24	25	16	35
ENSCAFG00845023640	270	234	205	267
ENSCAFG00845008013	804	831	689	704
ENSCAFG00845008016	1	0	0	0
ENSCAFG00845023646	166	173	93	107
ENSCAFG00845008015	0	0	0	0
ENSCAFG00845023645	5178	5100	4400	4414
ENSCAFG00845008018	0	0	0	0
ENSCAFG00845023644	0	0	0	0
ENSCAFG00845008017	11	10	28	26
ENSCAFG00845023643	0	0	0	0
ENSCAFG00845008019	0	0	2	0
ENSCAFG00845011668	366	306	261	275

ENSCAFG00845011669	829	786	867	891
ENSCAFG00845011664	12	7	20	8
ENSCAFG00845011665	0	0	0	0
ENSCAFG00845011666	1206	1155	1044	1070
ENSCAFG00845011667	485	464	415	440
ENSCAFG00845011660	0	0	0	0
ENSCAFG00845011661	12	13	18	16
ENSCAFG00845011662	0	0	0	0
ENSCAFG00845011663	0	0	0	0
ENSCAFG00845021098	0	0	0	0
ENSCAFG00845021092	8	13	10	14
ENSCAFG00845021093	40	49	30	30
ENSCAFG00845021090	0	0	0	0
ENSCAFG00845021091	0	0	0	0
ENSCAFG00845021096	700	691	624	680
ENSCAFG00845021097	6562	6385	6810	7020
ENSCAFG00845021094	425	401	459	460
ENSCAFG00845021095	0	0	0	0
ENSCAFG00845021089	0	0	0	0
ENSCAFG00845021087	0	3	1	1
ENSCAFG00845021088	3	0	1	2
ENSCAFG00845021081	3064	2850	3129	3318
ENSCAFG00845021082	481	559	431	468
ENSCAFG00845021080	0	0	0	0
ENSCAFG00845021085	543	457	476	569
ENSCAFG00845021086	4	1	0	6
ENSCAFG00845021083	0	0	0	0
ENSCAFG00845021084	0	0	0	0
ENSCAFG00845021078	1327	1297	1329	1351
ENSCAFG00845021079	731	789	710	635
ENSCAFG00845021076	640	703	653	694
ENSCAFG00845021077	0	2	4	0
ENSCAFG00845021070	0	0	0	0
ENSCAFG00845021071	0	0	0	0
ENSCAFG00845021074	1020	866	900	826
ENSCAFG00845021075	0	0	0	0
ENSCAFG00845021072	0	0	5	0
ENSCAFG00845021073	732	622	666	665
ENSCAFG00845008092	393	454	359	445
ENSCAFG00845008091	5	0	3	2
ENSCAFG00845008094	0	0	0	0

ENSCAFG00845008093	148	109	134	133
ENSCAFG00845008096	0	0	0	0
ENSCAFG00845008095	2923	2694	3120	2893
ENSCAFG00845008098	1581	1545	1567	1569
ENSCAFG00845008097	0	0	0	0
ENSCAFG00845021067	0	0	0	0
ENSCAFG00845008099	0	0	0	0
ENSCAFG00845021068	1019	1057	980	1057
ENSCAFG00845021065	0	0	0	1
ENSCAFG00845021066	0	0	0	0
ENSCAFG00845021069	0	0	0	0
ENSCAFG00845021060	0	1	0	1
ENSCAFG00845021063	0	0	0	0
ENSCAFG00845021064	0	0	0	0
ENSCAFG00845021061	4	4	4	7
ENSCAFG00845021062	410	382	419	423
ENSCAFG00845011808	8	15	17	25
ENSCAFG00845011809	0	0	0	0
ENSCAFG00845011815	192	175	181	220
ENSCAFG00845011816	230	242	152	150
ENSCAFG00845011817	393	334	363	373
ENSCAFG00845011818	219	237	206	192
ENSCAFG00845011811	0	1	0	0
ENSCAFG00845011812	436	406	311	331
ENSCAFG00845011813	497	492	459	479
ENSCAFG00845011814	1	0	3	0
ENSCAFG00845011810	1170	1078	1083	1024
ENSCAFG00845011804	0	0	0	0
ENSCAFG00845011805	8	13	12	8
ENSCAFG00845011806	36	32	28	52
ENSCAFG00845011807	37	44	65	52
ENSCAFG00845011800	126	111	123	106
ENSCAFG00845011801	1	6	1	4
ENSCAFG00845011802	0	0	0	0
ENSCAFG00845011803	16	1	5	4
ENSCAFG00845021218	1001	930	890	858
ENSCAFG00845021219	617	607	571	541
ENSCAFG00845021216	0	0	0	1
ENSCAFG00845023879	527	597	535	523
ENSCAFG00845011890	15	5	8	7
ENSCAFG00845021217	335	311	284	286

ENSCAFG00845023878	319	293	253	275
ENSCAFG00845008241	469	491	312	283
ENSCAFG00845008240	0	0	0	0
ENSCAFG00845008243	994	953	912	901
ENSCAFG00845021210	0	1	0	1
ENSCAFG00845023873	2	0	0	0
ENSCAFG00845008242	0	0	3	0
ENSCAFG00845021211	1363	1308	1336	1389
ENSCAFG00845023872	4	6	14	12
ENSCAFG00845008245	256	282	245	254
ENSCAFG00845023871	0	3	0	2
ENSCAFG00845008244	139	136	176	176
ENSCAFG00845023870	0	0	0	0
ENSCAFG00845008247	0	0	0	0
ENSCAFG00845021214	0	1	0	0
ENSCAFG00845023877	1240	1354	1212	1239
ENSCAFG00845008246	0	0	0	0
ENSCAFG00845021215	1977	1983	1838	1873
ENSCAFG00845023876	18	11	11	4
ENSCAFG00845008249	1730	1676	1483	1595
ENSCAFG00845021212	24	7	13	17
ENSCAFG00845023875	0	1	0	0
ENSCAFG00845008248	3612	3269	3134	3106
ENSCAFG00845021213	7	9	2	8
ENSCAFG00845023874	0	0	1	0
ENSCAFG00845011899	0	0	0	0
ENSCAFG00845011895	0	0	0	0
ENSCAFG00845011896	3	5	5	12
ENSCAFG00845011897	0	0	0	0
ENSCAFG00845011898	0	0	0	0
ENSCAFG00845011891	14	22	11	8
ENSCAFG00845011892	296	350	311	301
ENSCAFG00845011893	1	8	6	1
ENSCAFG00845011894	557	513	607	664
ENSCAFG00845021207	0	0	0	0
ENSCAFG00845021208	0	0	0	0
ENSCAFG00845023869	0	1	0	0
ENSCAFG00845021205	46	53	78	68
ENSCAFG00845023868	582	535	622	646
ENSCAFG00845021206	0	1	2	0
ENSCAFG00845023867	673	627	687	649

ENSCAFG00845008230	0	0	0	0
ENSCAFG00845021209	1095	1101	991	963
ENSCAFG00845008232	5	2	5	5
ENSCAFG00845023862	149	173	112	128
ENSCAFG00845008231	513	521	530	494
ENSCAFG00845021200	0	0	0	0
ENSCAFG00845023861	0	0	0	0
ENSCAFG00845008234	1779	1800	1645	1628
ENSCAFG00845023860	56	58	46	48
ENSCAFG00845008233	1800	1600	1658	1724
ENSCAFG00845008236	0	0	0	0
ENSCAFG00845021203	2711	2726	2716	2877
ENSCAFG00845023866	688	680	701	611
ENSCAFG00845008235	0	0	0	0
ENSCAFG00845021204	573	541	542	488
ENSCAFG00845023865	0	0	0	0
ENSCAFG00845008238	1035	996	989	995
ENSCAFG00845021201	55	62	65	84
ENSCAFG00845023864	1	0	2	5
ENSCAFG00845008237	6345	5990	5399	5620
ENSCAFG00845021202	81	81	91	71
ENSCAFG00845023863	0	0	0	0
ENSCAFG00845008239	0	0	0	0
ENSCAFG00845011888	19	28	29	12
ENSCAFG00845011889	0	0	1	0
ENSCAFG00845011884	0	0	0	0
ENSCAFG00845011885	271	276	269	299
ENSCAFG00845011886	42	40	36	38
ENSCAFG00845011887	234	199	246	244
ENSCAFG00845011880	62	58	80	58
ENSCAFG00845011881	6	4	9	2
ENSCAFG00845011882	555	527	667	709
ENSCAFG00845011883	0	0	0	0
ENSCAFG00845023859	213	238	259	222
ENSCAFG00845023858	1034	985	879	973
ENSCAFG00845023857	0	0	0	0
ENSCAFG00845023856	0	0	0	0
ENSCAFG00845008221	680	654	647	733
ENSCAFG00845023851	1710	1595	1822	1758
ENSCAFG00845008220	0	0	0	0
ENSCAFG00845023850	0	0	0	0

ENSCAFG00845008223	0	0	0	0
ENSCAFG00845008222	0	0	0	0
ENSCAFG00845008225	0	0	0	0
ENSCAFG00845023855	0	0	0	0
ENSCAFG00845008224	0	0	0	2
ENSCAFG00845023854	2	0	0	0
ENSCAFG00845008227	4	8	12	5
ENSCAFG00845023853	3	1	8	2
ENSCAFG00845008226	1	0	0	1
ENSCAFG00845023852	2305	2285	2163	2185
ENSCAFG00845008229	961	946	831	902
ENSCAFG00845008228	0	0	0	0
ENSCAFG00845011877	0	0	0	0
ENSCAFG00845011878	113	109	103	133
ENSCAFG00845011879	0	0	0	0
ENSCAFG00845011873	0	0	0	0
ENSCAFG00845011874	0	0	0	0
ENSCAFG00845011875	0	0	0	0
ENSCAFG00845011876	2	1	5	4
ENSCAFG00845011870	1	0	0	1
ENSCAFG00845011871	61	65	53	101
ENSCAFG00845011872	0	0	0	0
ENSCAFG00845023848	328	286	292	326
ENSCAFG00845023847	37	30	42	48
ENSCAFG00845023846	4823	4629	4738	4764
ENSCAFG00845023845	0	0	0	0
ENSCAFG00845023849	0	0	0	0
ENSCAFG00845008210	0	0	0	0
ENSCAFG00845023840	0	0	1	0
ENSCAFG00845008212	723	682	575	568
ENSCAFG00845008211	5	2	1	4
ENSCAFG00845008214	0	0	0	0
ENSCAFG00845023844	416	430	405	401
ENSCAFG00845008213	210	199	220	227
ENSCAFG00845023843	1004	928	919	889
ENSCAFG00845008216	14	11	11	11
ENSCAFG00845023842	0	0	0	0
ENSCAFG00845008215	474	482	442	492
ENSCAFG00845023841	0	6	7	3
ENSCAFG00845008218	72	50	70	93
ENSCAFG00845008217	4	3	3	1

ENSCAFG00845008219	2	6	7	6
ENSCAFG00845011866	1	0	1	1
ENSCAFG00845011867	0	0	0	0
ENSCAFG00845011868	0	0	0	1
ENSCAFG00845011869	78	73	66	68
ENSCAFG00845011862	0	0	1	0
ENSCAFG00845011863	183	213	191	216
ENSCAFG00845011864	348	339	317	357
ENSCAFG00845011865	1142	1006	1284	1255
ENSCAFG00845011860	0	0	0	1
ENSCAFG00845011861	0	0	0	0
ENSCAFG00845023837	1	3	1	1
ENSCAFG00845023836	0	0	0	0
ENSCAFG00845023835	0	0	0	0
ENSCAFG00845023834	1230	1238	1125	1099
ENSCAFG00845023839	45	36	63	41
ENSCAFG00845023838	4597	4496	4652	4729
ENSCAFG00845008201	2	1	3	0
ENSCAFG00845008200	0	0	0	0
ENSCAFG00845008203	1475	1350	1419	1361
ENSCAFG00845023833	794	778	756	751
ENSCAFG00845008202	504	520	502	485
ENSCAFG00845023832	11	5	18	10
ENSCAFG00845008205	326	358	344	350
ENSCAFG00845023831	720	684	896	828
ENSCAFG00845008204	0	0	0	0
ENSCAFG00845023830	2817	2788	2578	2753
ENSCAFG00845008207	968	885	855	950
ENSCAFG00845011859	341	311	414	404
ENSCAFG00845008206	160	141	157	151
ENSCAFG00845008209	0	0	0	0
ENSCAFG00845008208	3	3	4	4
ENSCAFG00845011855	1414	1351	1304	1386
ENSCAFG00845011856	81	126	116	128
ENSCAFG00845011857	0	0	0	0
ENSCAFG00845011858	0	0	0	0
ENSCAFG00845011851	8743	8367	9092	9295
ENSCAFG00845011852	331	253	319	269
ENSCAFG00845011853	0	0	0	0
ENSCAFG00845011854	26	25	18	21
ENSCAFG00845011850	577	547	536	486

ENSCAFG00845023826	1164	1139	1213	1104
ENSCAFG00845023825	149	194	226	215
ENSCAFG00845023824	760	771	683	619
ENSCAFG00845023823	10	8	5	5
ENSCAFG00845023829	83	82	62	64
ENSCAFG00845023828	0	0	0	0
ENSCAFG00845023827	3	5	5	7
ENSCAFG00845023822	24	24	19	14
ENSCAFG00845023821	0	1	0	0
ENSCAFG00845023820	42	49	54	58
ENSCAFG00845011848	3	2	0	0
ENSCAFG00845011849	3948	3991	2520	2562
ENSCAFG00845011844	10530	10320	10185	10455
ENSCAFG00845011845	2	0	0	1
ENSCAFG00845011846	10	13	13	22
ENSCAFG00845011847	0	0	2	0
ENSCAFG00845011840	0	0	0	0
ENSCAFG00845011841	0	0	4	0
ENSCAFG00845011842	0	1	0	0
ENSCAFG00845011843	1921	1876	1902	1919
ENSCAFG00845023815	0	0	0	0
ENSCAFG00845023814	29	25	34	15
ENSCAFG00845023813	0	0	0	0
ENSCAFG00845023812	0	0	0	0
ENSCAFG00845023819	291	276	287	271
ENSCAFG00845023818	0	0	0	0
ENSCAFG00845023817	4	0	1	0
ENSCAFG00845023816	435	468	508	495
ENSCAFG00845023811	2	1	3	0
ENSCAFG00845023810	0	0	0	0
ENSCAFG00845011837	1720	1632	1852	1865
ENSCAFG00845011838	0	0	0	0
ENSCAFG00845011839	0	0	0	0
ENSCAFG00845011833	502	523	478	479
ENSCAFG00845011834	0	0	0	0
ENSCAFG00845011835	1451	1518	1382	1518
ENSCAFG00845011836	10	12	8	11
ENSCAFG00845011830	1	1	6	5
ENSCAFG00845011831	542	510	525	537
ENSCAFG00845011832	8246	7910	7481	7624
ENSCAFG00845023809	15	35	21	28

ENSCAFG00845023804	1	0	3	2
ENSCAFG00845023803	64	87	76	67
ENSCAFG00845023802	2	12	6	15
ENSCAFG00845023801	0	0	0	0
ENSCAFG00845023808	1053	1137	1092	1029
ENSCAFG00845023807	0	0	0	0
ENSCAFG00845023806	66	67	83	69
ENSCAFG00845023805	1722	1747	1632	1605
ENSCAFG00845011819	0	0	0	0
ENSCAFG00845023800	0	0	0	0
ENSCAFG00845011826	331	314	310	276
ENSCAFG00845011827	407	319	379	381
ENSCAFG00845011828	0	0	0	1
ENSCAFG00845011829	0	0	0	0
ENSCAFG00845011822	46	52	40	28
ENSCAFG00845011823	2669	2770	2454	2333
ENSCAFG00845011824	5966	5964	5642	6101
ENSCAFG00845011825	821	850	816	874
ENSCAFG00845011820	378	405	356	400
ENSCAFG00845011821	152	148	164	140
ENSCAFG00845021177	0	0	0	0
ENSCAFG00845021178	0	0	0	0
ENSCAFG00845021175	1	0	0	0
ENSCAFG00845021176	0	0	0	0
ENSCAFG00845021179	165	182	480	447
ENSCAFG00845021170	0	0	0	0
ENSCAFG00845021173	0	0	0	0
ENSCAFG00845021174	3953	3820	5294	5380
ENSCAFG00845021171	413	402	411	409
ENSCAFG00845021172	0	0	0	0
ENSCAFG00845008191	8	17	4	8
ENSCAFG00845008190	0	0	0	0
ENSCAFG00845008193	41	20	46	37
ENSCAFG00845008192	18	14	15	16
ENSCAFG00845008195	585	522	572	551
ENSCAFG00845008194	1516	1391	1289	1340
ENSCAFG00845008197	96	110	85	75
ENSCAFG00845008196	0	0	0	0
ENSCAFG00845008199	288	335	352	370
ENSCAFG00845021166	128	124	188	168
ENSCAFG00845008198	6	17	4	5

ENSCAFG00845021167	18	19	27	15
ENSCAFG00845021164	447	446	413	464
ENSCAFG00845021165	573	562	489	453
ENSCAFG00845021168	2300	2233	2150	2223
ENSCAFG00845021169	0	0	0	0
ENSCAFG00845021162	45440	44875	48277	50385
ENSCAFG00845021163	4	3	2	2
ENSCAFG00845021160	0	0	0	0
ENSCAFG00845021161	0	0	0	0
ENSCAFG00845008180	437	395	372	381
ENSCAFG00845008182	0	0	0	0
ENSCAFG00845008181	17	12	20	10
ENSCAFG00845008184	417	375	342	307
ENSCAFG00845008183	0	0	0	0
ENSCAFG00845008186	760	778	689	752
ENSCAFG00845008185	0	0	0	0
ENSCAFG00845008188	1856	1881	1869	1642
ENSCAFG00845021155	0	0	0	0
ENSCAFG00845008187	353	351	339	395
ENSCAFG00845021156	0	0	0	1
ENSCAFG00845021153	125	121	107	122
ENSCAFG00845008189	261	228	240	231
ENSCAFG00845021154	0	1	0	0
ENSCAFG00845021159	372	345	341	367
ENSCAFG00845021157	0	0	0	0
ENSCAFG00845021158	0	0	0	0
ENSCAFG00845021151	2	0	3	1
ENSCAFG00845021152	3	0	0	1
ENSCAFG00845021150	6	9	5	10
ENSCAFG00845008171	2252	2242	2415	2386
ENSCAFG00845008170	0	0	0	0
ENSCAFG00845008173	486	530	450	530
ENSCAFG00845008172	0	0	0	0
ENSCAFG00845008175	3	3	0	1
ENSCAFG00845008174	0	0	0	0
ENSCAFG00845008177	1758	1774	1974	1978
ENSCAFG00845021144	47	71	58	81
ENSCAFG00845008176	2	1	3	10
ENSCAFG00845021145	43829	42440	44878	44163
ENSCAFG00845008179	0	0	0	0
ENSCAFG00845021142	0	0	1	1

ENSCAFG00845008178	100	78	81	53
ENSCAFG00845021143	0	0	0	0
ENSCAFG00845021148	0	0	0	0
ENSCAFG00845021149	56	48	23	51
ENSCAFG00845021146	0	0	7	0
ENSCAFG00845021147	11	15	8	8
ENSCAFG00845021140	0	0	0	0
ENSCAFG00845021141	18	11	19	15
ENSCAFG00845008160	0	0	0	0
ENSCAFG00845021139	4137	3662	3904	4206
ENSCAFG00845008162	614	640	545	599
ENSCAFG00845008161	75	76	70	65
ENSCAFG00845008164	0	0	0	0
ENSCAFG00845008163	8	11	6	8
ENSCAFG00845008166	0	2	1	0
ENSCAFG00845021133	0	2	1	2
ENSCAFG00845023796	0	0	0	0
ENSCAFG00845008165	1	1	0	0
ENSCAFG00845021134	0	0	0	0
ENSCAFG00845023795	7	3	1	4
ENSCAFG00845008168	1073	1017	931	991
ENSCAFG00845021131	4	5	10	5
ENSCAFG00845023794	0	0	0	0
ENSCAFG00845008167	0	1	1	0
ENSCAFG00845021132	0	0	0	0
ENSCAFG00845023793	690	733	725	844
ENSCAFG00845021137	999	1042	1001	1054
ENSCAFG00845008169	20	16	30	25
ENSCAFG00845021138	0	0	0	0
ENSCAFG00845023799	929	934	901	954
ENSCAFG00845021135	1131	1072	1060	1073
ENSCAFG00845023798	0	0	0	0
ENSCAFG00845021136	1	0	0	0
ENSCAFG00845023797	1	0	2	0
ENSCAFG00845023792	0	0	0	0
ENSCAFG00845021130	30	29	32	37
ENSCAFG00845023791	2	1	1	0
ENSCAFG00845023790	3	2	9	3
ENSCAFG00845021128	0	0	0	0
ENSCAFG00845021129	3	3	0	0
ENSCAFG00845008151	545	491	480	398

ENSCAFG00845008150	7	3	5	5
ENSCAFG00845008153	0	0	0	0
ENSCAFG00845008152	0	0	0	0
ENSCAFG00845008155	0	0	0	0
ENSCAFG00845021122	0	0	0	0
ENSCAFG00845023785	4	7	5	4
ENSCAFG00845008154	757	775	833	783
ENSCAFG00845021123	0	0	0	0
ENSCAFG00845023784	0	0	0	0
ENSCAFG00845008157	386	365	371	357
ENSCAFG00845021120	0	0	0	0
ENSCAFG00845023783	0	0	2	1
ENSCAFG00845008156	1	0	0	0
ENSCAFG00845021121	0	0	0	1
ENSCAFG00845023782	0	0	0	0
ENSCAFG00845008159	0	0	0	0
ENSCAFG00845021126	599	698	608	596
ENSCAFG00845023789	0	0	2	0
ENSCAFG00845008158	4037	3925	3697	3694
ENSCAFG00845021127	0	0	0	0
ENSCAFG00845023788	6753	6416	6438	6579
ENSCAFG00845021124	681	662	618	637
ENSCAFG00845023787	85	71	48	30
ENSCAFG00845021125	0	0	0	0
ENSCAFG00845023786	0	0	4	0
ENSCAFG00845023781	0	0	0	0
ENSCAFG00845023780	0	0	0	0
ENSCAFG00845021119	0	0	0	0
ENSCAFG00845011790	303	314	292	302
ENSCAFG00845021117	319	330	330	412
ENSCAFG00845011791	0	0	0	0
ENSCAFG00845021118	494	463	474	487
ENSCAFG00845023779	1462	1513	1474	1409
ENSCAFG00845008140	0	0	0	0
ENSCAFG00845008142	81	84	71	75
ENSCAFG00845008141	0	0	0	0
ENSCAFG00845008144	1457	1302	1391	1399
ENSCAFG00845021111	151	153	155	117
ENSCAFG00845023774	0	0	0	0
ENSCAFG00845008143	790	837	665	680
ENSCAFG00845021112	337	303	280	263

ENSCAFG00845008146	7	8	8	4
ENSCAFG00845023772	12	11	5	11
ENSCAFG00845008145	5	12	3	5
ENSCAFG00845021110	0	0	0	0
ENSCAFG00845023771	0	0	0	0
ENSCAFG00845008148	790	836	782	779
ENSCAFG00845021115	3234	3045	3170	3097
ENSCAFG00845023778	3	4	1	1
ENSCAFG00845008147	0	0	0	0
ENSCAFG00845021116	0	0	0	0
ENSCAFG00845023777	783	730	657	628
ENSCAFG00845021113	0	0	0	0
ENSCAFG00845023776	1387	1309	1271	1241
ENSCAFG00845008149	309	285	239	240
ENSCAFG00845021114	1	0	0	0
ENSCAFG00845023775	0	0	0	0
ENSCAFG00845023770	847	725	620	647
ENSCAFG00845011796	0	0	0	0
ENSCAFG00845011797	2	3	9	8
ENSCAFG00845011798	761	612	609	569
ENSCAFG00845011799	0	0	0	0
ENSCAFG00845011792	0	0	0	0
ENSCAFG00845011793	17	11	21	23
ENSCAFG00845011794	0	0	0	4
ENSCAFG00845011795	185	154	197	183
ENSCAFG00845021108	140	151	170	162
ENSCAFG00845021109	2942	2859	2527	2400
ENSCAFG00845021106	524	466	458	519
ENSCAFG00845023769	1	0	1	5
ENSCAFG00845011780	188	189	117	108
ENSCAFG00845021107	3	2	1	3
ENSCAFG00845023768	448	393	335	409
ENSCAFG00845008131	618	568	628	628
ENSCAFG00845008130	0	0	0	0
ENSCAFG00845008133	200	183	184	144
ENSCAFG00845021100	20	19	23	20
ENSCAFG00845023763	0	0	0	0
ENSCAFG00845008132	17	9	8	5
ENSCAFG00845021101	0	0	0	0
ENSCAFG00845023762	543	530	585	586
ENSCAFG00845008135	0	0	0	0

ENSCAFG00845023761	460	437	516	438
ENSCAFG00845008134	7	7	5	2
ENSCAFG00845023760	0	0	0	0
ENSCAFG00845008137	0	0	0	0
ENSCAFG00845021104	471	426	427	429
ENSCAFG00845023767	564	575	625	669
ENSCAFG00845008136	353	384	319	258
ENSCAFG00845021105	5	2	3	2
ENSCAFG00845023766	294	273	310	259
ENSCAFG00845008139	3401	3293	3347	3329
ENSCAFG00845021102	0	0	0	0
ENSCAFG00845023765	0	0	0	0
ENSCAFG00845008138	193	188	105	118
ENSCAFG00845021103	17	8	11	12
ENSCAFG00845023764	0	0	0	0
ENSCAFG00845011789	2004	1851	1945	1902
ENSCAFG00845011785	132	121	120	123
ENSCAFG00845011786	0	0	0	0
ENSCAFG00845011787	34	31	32	30
ENSCAFG00845011788	2754	2650	2955	3007
ENSCAFG00845011781	5700	5547	5635	5678
ENSCAFG00845011782	0	0	0	0
ENSCAFG00845011783	155	157	126	143
ENSCAFG00845011784	1377	1493	1564	1462
ENSCAFG00845021199	21	42	32	27
ENSCAFG00845021197	0	0	0	0
ENSCAFG00845021198	0	2	2	1
ENSCAFG00845021191	1	0	0	0
ENSCAFG00845021192	403	364	371	415
ENSCAFG00845021190	0	0	0	0
ENSCAFG00845021195	34	34	25	33
ENSCAFG00845021196	0	0	0	0
ENSCAFG00845021193	87	67	91	99
ENSCAFG00845021194	1060	1008	855	966
ENSCAFG00845021188	883	842	831	810
ENSCAFG00845021189	0	0	0	0
ENSCAFG00845021186	283	259	288	246
ENSCAFG00845021187	688	730	796	872
ENSCAFG00845021180	135	150	133	117
ENSCAFG00845021181	2	1	0	0
ENSCAFG00845021184	0	0	0	0

ENSCAFG00845021185	0	0	0	0
ENSCAFG00845021182	5	7	2	12
ENSCAFG00845021183	0	1	0	1
ENSCAFG00845023914	0	0	0	0
ENSCAFG00845023913	0	0	0	0
ENSCAFG00845023912	0	0	2	0
ENSCAFG00845023911	123	109	124	148
ENSCAFG00845023918	0	0	0	0
ENSCAFG00845023917	0	0	0	0
ENSCAFG00845023916	12	14	17	10
ENSCAFG00845023915	0	0	0	0
ENSCAFG00845011929	47	39	65	60
ENSCAFG00845023910	0	0	0	0
ENSCAFG00845011936	0	0	0	0
ENSCAFG00845011937	0	0	0	0
ENSCAFG00845011938	466	485	485	434
ENSCAFG00845011939	0	0	0	0
ENSCAFG00845011932	0	0	0	0
ENSCAFG00845011933	0	0	0	0
ENSCAFG00845011934	6	3	5	3
ENSCAFG00845011935	10	2	6	4
ENSCAFG00845011930	1	0	0	0
ENSCAFG00845023909	0	2	0	0
ENSCAFG00845011931	343	374	353	299
ENSCAFG00845023908	0	0	0	0
ENSCAFG00845023903	0	0	0	0
ENSCAFG00845023902	337	335	362	332
ENSCAFG00845023901	0	0	0	0
ENSCAFG00845023900	2	3	1	3
ENSCAFG00845023907	0	0	0	0
ENSCAFG00845023906	0	0	0	0
ENSCAFG00845023904	0	0	1	2
ENSCAFG00845011918	0	1	0	0
ENSCAFG00845011919	802	732	815	832
ENSCAFG00845011925	758	819	840	824
ENSCAFG00845011926	0	0	0	0
ENSCAFG00845011927	1	0	3	3
ENSCAFG00845011928	279	243	228	205
ENSCAFG00845011921	0	1	0	0
ENSCAFG00845011922	44	40	43	35
ENSCAFG00845011923	0	0	0	0

ENSCAFG00845011924	126	109	157	141
ENSCAFG00845011920	1	2	3	1
ENSCAFG00845011907	125	118	68	101
ENSCAFG00845011908	58	47	51	34
ENSCAFG00845011909	428	467	389	421
ENSCAFG00845011914	2	4	0	0
ENSCAFG00845011915	0	0	0	0
ENSCAFG00845011916	1	1	0	1
ENSCAFG00845011917	0	3	2	0
ENSCAFG00845011910	2272	2256	1355	1461
ENSCAFG00845011911	0	0	0	0
ENSCAFG00845011912	1789	1721	1481	1573
ENSCAFG00845011913	45	33	35	43
ENSCAFG00845011903	0	0	0	0
ENSCAFG00845011904	96	81	52	60
ENSCAFG00845011905	0	1	0	1
ENSCAFG00845011906	0	1	4	3
ENSCAFG00845011900	0	0	0	0
ENSCAFG00845011901	3	1	1	2
ENSCAFG00845011902	0	0	0	0
ENSCAFG00845021339	1993	1869	1776	1927
ENSCAFG00845021337	0	0	0	0
ENSCAFG00845021338	0	0	0	0
ENSCAFG00845023999	0	0	0	0
ENSCAFG00845008360	0	0	0	0
ENSCAFG00845008362	3	0	0	0
ENSCAFG00845008361	0	0	0	0
ENSCAFG00845008364	0	0	0	0
ENSCAFG00845021331	0	0	0	0
ENSCAFG00845023994	3048	3029	2296	2358
ENSCAFG00845008363	0	0	0	0
ENSCAFG00845021332	35	28	28	41
ENSCAFG00845023993	0	0	0	0
ENSCAFG00845008366	49	51	35	31
ENSCAFG00845023992	1426	1436	1034	960
ENSCAFG00845008365	119	132	181	164
ENSCAFG00845021330	0	0	0	0
ENSCAFG00845023991	694	695	725	719
ENSCAFG00845008368	0	0	0	0
ENSCAFG00845021335	1274	1153	1303	1321
ENSCAFG00845023998	0	0	1	0

ENSCAFG00845008367	413	390	409	367
ENSCAFG00845021336	1127	1151	1080	1082
ENSCAFG00845023997	2	0	2	2
ENSCAFG00845021333	0	0	0	0
ENSCAFG00845023996	261	242	213	224
ENSCAFG00845008369	1546	1592	1492	1533
ENSCAFG00845021334	792	837	867	909
ENSCAFG00845023995	522	496	438	398
ENSCAFG00845023990	0	0	0	1
ENSCAFG00845021328	32	32	55	27
ENSCAFG00845021329	2781	2792	2847	2832
ENSCAFG00845021326	0	0	0	0
ENSCAFG00845023989	282	274	280	338
ENSCAFG00845021327	237	248	222	233
ENSCAFG00845023988	426	386	400	375
ENSCAFG00845008351	67	78	70	80
ENSCAFG00845008350	0	2	0	2
ENSCAFG00845008353	0	0	0	0
ENSCAFG00845021320	62	47	43	41
ENSCAFG00845023983	3	1	4	0
ENSCAFG00845008352	2	1	4	1
ENSCAFG00845021321	182	243	221	161
ENSCAFG00845023982	2	0	1	0
ENSCAFG00845008355	134	162	175	140
ENSCAFG00845023981	0	0	0	0
ENSCAFG00845008354	0	0	0	0
ENSCAFG00845023980	341	296	281	325
ENSCAFG00845008357	2326	2154	2206	2409
ENSCAFG00845021324	0	0	0	0
ENSCAFG00845023987	0	0	0	0
ENSCAFG00845008356	0	0	0	0
ENSCAFG00845021325	336	339	302	303
ENSCAFG00845023986	0	0	0	0
ENSCAFG00845008359	0	0	0	0
ENSCAFG00845021322	561	532	610	707
ENSCAFG00845023985	0	0	0	0
ENSCAFG00845008358	0	0	0	0
ENSCAFG00845021323	21	19	18	16
ENSCAFG00845023984	7	9	20	10
ENSCAFG00845021317	3	2	0	6
ENSCAFG00845021318	677	647	715	650

ENSCAFG00845023979	231	175	220	210
ENSCAFG00845021315	1	3	0	0
ENSCAFG00845023978	0	0	0	0
ENSCAFG00845021316	0	0	0	0
ENSCAFG00845023977	27	20	16	28
ENSCAFG00845008340	0	0	0	0
ENSCAFG00845021319	813	845	923	852
ENSCAFG00845008342	3100	2920	3136	3089
ENSCAFG00845023972	0	0	0	0
ENSCAFG00845008341	6	3	3	2
ENSCAFG00845021310	13	7	20	13
ENSCAFG00845023971	3	5	3	3
ENSCAFG00845008344	2556	2367	2393	2462
ENSCAFG00845008343	0	0	0	0
ENSCAFG00845008346	0	0	0	0
ENSCAFG00845021313	42	48	37	43
ENSCAFG00845023976	8	13	15	5
ENSCAFG00845008345	56	82	78	97
ENSCAFG00845021314	0	0	0	0
ENSCAFG00845023975	0	0	0	0
ENSCAFG00845008348	5	1	0	2
ENSCAFG00845021311	422	478	371	464
ENSCAFG00845023974	563	548	503	547
ENSCAFG00845008347	503	506	551	513
ENSCAFG00845021312	609	556	572	567
ENSCAFG00845023973	461	484	569	578
ENSCAFG00845008349	10	20	11	5
ENSCAFG00845011998	0	0	0	0
ENSCAFG00845011999	0	0	0	0
ENSCAFG00845011994	1453	1382	1426	1411
ENSCAFG00845011995	0	0	0	0
ENSCAFG00845011996	0	0	0	0
ENSCAFG00845011997	19	20	23	31
ENSCAFG00845011990	0	0	0	0
ENSCAFG00845011991	237	215	250	212
ENSCAFG00845011992	321	373	324	359
ENSCAFG00845011993	0	0	0	0
ENSCAFG00845021306	1397	1250	1329	1346
ENSCAFG00845023969	0	0	0	0
ENSCAFG00845021307	1843	1783	2013	2107
ENSCAFG00845023968	0	0	0	0

ENSCAFG00845021304	470	466	423	424
ENSCAFG00845023967	430	345	475	436
ENSCAFG00845021305	0	0	0	0
ENSCAFG00845023966	0	0	0	0
ENSCAFG00845021308	430	414	446	469
ENSCAFG00845021309	59	55	52	67
ENSCAFG00845008331	937	976	938	971
ENSCAFG00845023961	4222	4279	4289	4562
ENSCAFG00845008330	723	782	636	709
ENSCAFG00845023960	0	0	0	0
ENSCAFG00845008333	0	0	0	0
ENSCAFG00845008332	0	0	0	0
ENSCAFG00845008335	0	0	0	0
ENSCAFG00845021302	0	0	0	1
ENSCAFG00845023965	983	936	924	923
ENSCAFG00845008334	0	0	0	1
ENSCAFG00845021303	0	0	0	0
ENSCAFG00845023964	37	31	52	37
ENSCAFG00845008337	0	0	0	0
ENSCAFG00845021300	1785	1815	1391	1492
ENSCAFG00845023963	5	5	9	8
ENSCAFG00845008336	0	0	0	0
ENSCAFG00845021301	32	38	55	43
ENSCAFG00845023962	1	0	0	0
ENSCAFG00845008339	647	662	658	608
ENSCAFG00845008338	175	225	142	139
ENSCAFG00845011987	953	925	978	866
ENSCAFG00845011988	0	0	0	0
ENSCAFG00845011989	0	0	0	0
ENSCAFG00845011983	1	0	2	3
ENSCAFG00845011984	1396	1334	1228	1324
ENSCAFG00845011985	0	0	0	0
ENSCAFG00845011986	6179	5943	5434	5786
ENSCAFG00845011980	0	0	0	0
ENSCAFG00845011981	0	0	0	0
ENSCAFG00845011982	2016	1901	2083	2052
ENSCAFG00845023958	0	0	0	0
ENSCAFG00845023957	1578	1463	1827	1690
ENSCAFG00845023956	0	0	0	0
ENSCAFG00845023955	0	0	0	0
ENSCAFG00845023959	3	0	0	0

ENSCAFG00845008320	1111	1088	1124	1220
ENSCAFG00845023950	0	0	0	2
ENSCAFG00845008322	0	0	0	0
ENSCAFG00845008321	0	0	0	0
ENSCAFG00845008324	504	559	478	476
ENSCAFG00845023954	8	13	5	12
ENSCAFG00845008323	2642	2467	2371	2303
ENSCAFG00845023953	378	375	428	391
ENSCAFG00845008326	0	0	0	0
ENSCAFG00845008325	3687	3443	3257	3411
ENSCAFG00845023951	1	2	2	0
ENSCAFG00845008328	2679	2525	2405	2356
ENSCAFG00845008327	1098	993	989	1071
ENSCAFG00845008329	561	559	571	636
ENSCAFG00845011976	32	26	25	27
ENSCAFG00845011977	237	232	251	248
ENSCAFG00845011978	759	712	732	886
ENSCAFG00845011979	18	27	11	20
ENSCAFG00845011972	0	0	0	0
ENSCAFG00845011973	1221	1206	1161	1136
ENSCAFG00845011974	0	0	0	0
ENSCAFG00845011975	4000	4148	4212	4152
ENSCAFG00845011970	513	514	504	509
ENSCAFG00845011971	2	1	0	0
ENSCAFG00845023947	0	0	0	1
ENSCAFG00845023946	98	113	107	110
ENSCAFG00845023945	299	310	237	235
ENSCAFG00845023944	0	0	0	0
ENSCAFG00845023949	91	56	79	77
ENSCAFG00845023948	1	0	1	0
ENSCAFG00845008311	8	3	6	2
ENSCAFG00845008310	511	451	550	519
ENSCAFG00845008313	0	0	7	0
ENSCAFG00845023943	308	312	373	327
ENSCAFG00845008312	55	41	38	62
ENSCAFG00845023942	317	303	328	298
ENSCAFG00845008315	0	0	0	0
ENSCAFG00845023941	0	0	0	0
ENSCAFG00845008314	177	178	202	218
ENSCAFG00845023940	0	0	0	0
ENSCAFG00845008317	0	0	0	0

ENSCAFG00845011969	0	0	0	0
ENSCAFG00845008316	287	297	327	332
ENSCAFG00845008319	1	0	2	0
ENSCAFG00845008318	0	2	0	0
ENSCAFG00845011965	561	628	497	498
ENSCAFG00845011966	0	0	0	0
ENSCAFG00845011967	0	0	0	0
ENSCAFG00845011968	11	14	3	10
ENSCAFG00845011961	0	0	0	0
ENSCAFG00845011962	0	0	0	0
ENSCAFG00845011963	0	0	0	0
ENSCAFG00845011964	107	95	96	118
ENSCAFG00845011960	140	135	188	131
ENSCAFG00845023936	32	21	9	15
ENSCAFG00845023935	0	0	0	0
ENSCAFG00845023934	0	0	0	0
ENSCAFG00845023933	0	0	0	0
ENSCAFG00845023939	200	173	166	162
ENSCAFG00845023938	0	0	0	0
ENSCAFG00845023937	56	50	72	65
ENSCAFG00845008300	2	3	0	0
ENSCAFG00845008302	0	0	0	0
ENSCAFG00845023932	500	461	506	583
ENSCAFG00845008301	300	280	268	280
ENSCAFG00845023931	0	0	0	0
ENSCAFG00845008304	425	394	332	314
ENSCAFG00845023930	0	0	0	0
ENSCAFG00845008303	3	5	6	8
ENSCAFG00845008306	284	311	311	395
ENSCAFG00845011958	77	100	97	97
ENSCAFG00845008305	0	0	0	0
ENSCAFG00845011959	0	0	0	0
ENSCAFG00845008308	0	0	0	0
ENSCAFG00845008307	1861	1838	1813	1952
ENSCAFG00845011954	0	0	0	0
ENSCAFG00845008309	0	0	0	0
ENSCAFG00845011955	0	0	0	0
ENSCAFG00845011956	1267	1301	1231	1195
ENSCAFG00845011957	1	0	0	0
ENSCAFG00845011950	0	0	0	0
ENSCAFG00845011951	0	1	0	0

ENSCAFG00845011952	103	120	100	109
ENSCAFG00845011953	0	0	0	0
ENSCAFG00845023925	0	0	0	0
ENSCAFG00845023924	271	285	201	188
ENSCAFG00845023923	37	31	44	63
ENSCAFG00845023922	2047	1895	1848	1865
ENSCAFG00845023929	3	5	2	1
ENSCAFG00845023928	0	0	0	0
ENSCAFG00845023927	0	0	0	0
ENSCAFG00845023926	1541	1611	1467	1500
ENSCAFG00845023921	0	0	0	0
ENSCAFG00845023920	0	0	0	0
ENSCAFG00845011947	0	0	0	0
ENSCAFG00845011948	36	61	45	43
ENSCAFG00845011949	0	0	0	0
ENSCAFG00845011943	0	0	0	0
ENSCAFG00845011944	408	430	377	361
ENSCAFG00845011945	741	694	749	698
ENSCAFG00845011946	0	0	0	0
ENSCAFG00845011940	1	1	3	3
ENSCAFG00845011941	2	5	8	8
ENSCAFG00845011942	0	0	0	0
ENSCAFG00845023919	67	47	46	40
ENSCAFG00845021298	0	0	0	0
ENSCAFG00845021299	0	0	0	0
ENSCAFG00845021296	1	0	3	1
ENSCAFG00845021297	0	0	0	0
ENSCAFG00845021290	655	696	435	461
ENSCAFG00845021291	0	0	1	2
ENSCAFG00845021294	15	7	16	11
ENSCAFG00845021295	618	584	627	594
ENSCAFG00845021292	80	59	86	90
ENSCAFG00845021293	0	0	0	0
ENSCAFG00845021287	0	0	0	0
ENSCAFG00845021288	0	4	0	2
ENSCAFG00845021285	0	0	0	0
ENSCAFG00845021286	0	0	0	0
ENSCAFG00845021289	492	469	479	464
ENSCAFG00845021280	9	6	3	5
ENSCAFG00845021283	0	0	0	0
ENSCAFG00845021284	0	0	0	0

ENSCAFG00845021281	0	0	0	0
ENSCAFG00845021282	648	673	637	619
ENSCAFG00845021276	0	0	0	0
ENSCAFG00845021277	1877	1795	1829	2009
ENSCAFG00845021274	598	512	607	519
ENSCAFG00845021275	1339	1361	1420	1394
ENSCAFG00845021278	0	0	0	0
ENSCAFG00845021279	0	0	1	0
ENSCAFG00845021272	0	0	0	0
ENSCAFG00845021273	51	40	81	68
ENSCAFG00845021270	459	458	313	357
ENSCAFG00845021271	3491	3303	3485	3730
ENSCAFG00845008290	13	10	16	14
ENSCAFG00845008292	0	0	0	0
ENSCAFG00845008291	522	500	556	564
ENSCAFG00845008294	0	0	0	0
ENSCAFG00845008293	712	710	706	733
ENSCAFG00845008296	76	115	94	89
ENSCAFG00845008295	560	558	537	568
ENSCAFG00845008298	0	2	4	2
ENSCAFG00845021265	0	0	0	0
ENSCAFG00845008297	2	0	0	0
ENSCAFG00845021266	0	0	0	0
ENSCAFG00845021263	68	65	35	55
ENSCAFG00845008299	3	2	3	3
ENSCAFG00845021264	249	242	277	260
ENSCAFG00845021269	0	0	0	0
ENSCAFG00845021267	1207	1145	1122	1124
ENSCAFG00845021268	417	479	437	483
ENSCAFG00845021261	0	0	0	0
ENSCAFG00845021262	875	885	914	881
ENSCAFG00845021260	2	1	0	0
ENSCAFG00845008281	6	6	9	8
ENSCAFG00845008280	0	0	6	2
ENSCAFG00845008283	0	0	0	0
ENSCAFG00845008282	3	2	1	1
ENSCAFG00845008285	451	457	502	534
ENSCAFG00845008284	157	152	149	157
ENSCAFG00845008287	0	0	0	0
ENSCAFG00845021254	87	69	88	92
ENSCAFG00845008286	0	0	0	0

ENSCAFG00845021255	481	456	279	309
ENSCAFG00845008289	0	1	0	1
ENSCAFG00845021252	1296	1206	1117	1219
ENSCAFG00845008288	1068	1102	1016	1078
ENSCAFG00845021253	862	888	883	908
ENSCAFG00845021258	0	0	0	0
ENSCAFG00845021259	0	0	0	0
ENSCAFG00845021256	1	0	1	0
ENSCAFG00845021257	4	6	4	1
ENSCAFG00845021250	159	112	160	149
ENSCAFG00845021251	992	970	971	900
ENSCAFG00845008270	52	42	42	54
ENSCAFG00845021249	1	0	1	0
ENSCAFG00845008272	28	27	26	15
ENSCAFG00845008271	0	0	0	0
ENSCAFG00845008274	85	107	105	97
ENSCAFG00845008273	0	0	0	0
ENSCAFG00845008276	2033	1997	2035	2143
ENSCAFG00845021243	245	258	268	291
ENSCAFG00845008275	0	0	0	0
ENSCAFG00845021244	0	0	0	0
ENSCAFG00845008278	32	28	33	33
ENSCAFG00845021241	0	0	0	0
ENSCAFG00845008277	231	225	210	183
ENSCAFG00845021242	4	4	10	5
ENSCAFG00845021247	0	1	2	0
ENSCAFG00845008279	535	497	579	576
ENSCAFG00845021248	75	66	69	67
ENSCAFG00845021245	0	0	0	0
ENSCAFG00845021246	216	228	246	275
ENSCAFG00845021240	1324	1270	1126	1225
ENSCAFG00845021238	0	0	0	0
ENSCAFG00845021239	40	46	61	53
ENSCAFG00845008261	0	0	0	0
ENSCAFG00845008260	104	115	118	100
ENSCAFG00845008263	8618	8039	8562	8661
ENSCAFG00845008262	0	0	0	0
ENSCAFG00845008265	584	525	563	546
ENSCAFG00845021232	0	0	0	0
ENSCAFG00845023895	16	7	7	8
ENSCAFG00845008264	0	0	0	0

ENSCAFG00845021233	0	0	0	0
ENSCAFG00845023894	0	0	0	0
ENSCAFG00845008267	4032	3915	3655	3708
ENSCAFG00845021230	0	0	0	0
ENSCAFG00845023893	0	0	0	2
ENSCAFG00845008266	0	0	0	0
ENSCAFG00845021231	13	11	19	26
ENSCAFG00845023892	418	346	425	377
ENSCAFG00845008269	9481	8934	8828	9297
ENSCAFG00845021236	1	0	3	1
ENSCAFG00845023899	67	67	68	68
ENSCAFG00845008268	1053	987	1044	1139
ENSCAFG00845021237	29	18	14	41
ENSCAFG00845023898	22	24	22	22
ENSCAFG00845021234	77	55	56	51
ENSCAFG00845023897	766	766	805	769
ENSCAFG00845021235	612	575	537	636
ENSCAFG00845023896	0	0	0	0
ENSCAFG00845023891	0	1	0	0
ENSCAFG00845023890	84	74	97	87
ENSCAFG00845021229	3	3	4	4
ENSCAFG00845021227	0	0	0	0
ENSCAFG00845021228	792	711	721	752
ENSCAFG00845023889	0	0	0	1
ENSCAFG00845008250	0	0	0	0
ENSCAFG00845008252	1105	1155	985	973
ENSCAFG00845008251	0	0	0	0
ENSCAFG00845008254	805	809	859	924
ENSCAFG00845021221	254	259	237	255
ENSCAFG00845023884	267	227	257	226
ENSCAFG00845008253	0	0	0	0
ENSCAFG00845021222	0	0	0	0
ENSCAFG00845023883	0	0	0	0
ENSCAFG00845008256	22	14	10	21
ENSCAFG00845023882	29	21	23	18
ENSCAFG00845008255	251	235	213	207
ENSCAFG00845021220	4	13	13	20
ENSCAFG00845023881	0	0	0	0
ENSCAFG00845008258	540	564	573	593
ENSCAFG00845021225	20	14	19	29
ENSCAFG00845023888	1318	1232	1388	1388

ENSCAFG00845008257	0	1	1	0
ENSCAFG00845021226	0	0	1	0
ENSCAFG00845023887	0	0	0	0
ENSCAFG00845021223	25	15	20	6
ENSCAFG00845023886	2870	2732	2889	2860
ENSCAFG00845008259	0	0	0	0
ENSCAFG00845021224	0	0	0	0
ENSCAFG00845023885	0	0	0	0
ENSCAFG00845023880	0	0	0	0
ENSCAFG00845025818	0	2	0	0
ENSCAFG00845025819	0	0	0	0
ENSCAFG00845001849	0	0	0	0
ENSCAFG00845001847	0	1	2	0
ENSCAFG00845001848	1353	1301	959	871
ENSCAFG00845025815	186	205	196	192
ENSCAFG00845001845	120	137	119	105
ENSCAFG00845001846	0	0	0	0
ENSCAFG00845025817	0	0	4	2
ENSCAFG00845001843	0	0	0	0
ENSCAFG00845025810	282	231	243	246
ENSCAFG00845001844	0	0	0	0
ENSCAFG00845025811	0	0	0	0
ENSCAFG00845001841	901	815	903	819
ENSCAFG00845025812	450	355	400	349
ENSCAFG00845001842	0	0	0	0
ENSCAFG00845025813	1784	1815	1736	1807
ENSCAFG00845001840	136	146	135	155
ENSCAFG00845013839	0	0	0	0
ENSCAFG00845013836	6	8	10	5
ENSCAFG00845013835	1880	1937	1886	1822
ENSCAFG00845013838	183	183	144	155
ENSCAFG00845013837	32	32	22	18
ENSCAFG00845013832	312	347	506	602
ENSCAFG00845013831	166	180	160	202
ENSCAFG00845013834	8	1	8	13
ENSCAFG00845013833	108	114	122	107
ENSCAFG00845013830	308	287	273	296
ENSCAFG00845025807	0	0	0	0
ENSCAFG00845025808	0	0	0	0
ENSCAFG00845025809	0	0	0	0
ENSCAFG00845001858	251	193	228	216

ENSCAFG00845025803	0	0	0	0
ENSCAFG00845001859	230	260	301	272
ENSCAFG00845025804	0	0	0	0
ENSCAFG00845001856	4	4	1	8
ENSCAFG00845025805	40	41	40	29
ENSCAFG00845001857	1	0	2	0
ENSCAFG00845025806	0	1	0	0
ENSCAFG00845001854	1566	1614	1657	1741
ENSCAFG00845001855	0	0	0	0
ENSCAFG00845025800	0	0	0	0
ENSCAFG00845001852	0	0	0	0
ENSCAFG00845025801	3	0	0	2
ENSCAFG00845001853	1399	1366	1574	1811
ENSCAFG00845025802	0	0	0	0
ENSCAFG00845001850	929	928	843	851
ENSCAFG00845001851	0	0	0	0
ENSCAFG00845013829	38	48	37	32
ENSCAFG00845013828	6	6	21	15
ENSCAFG00845013825	17	21	26	19
ENSCAFG00845013824	922	1011	964	965
ENSCAFG00845013827	1972	1978	2000	2004
ENSCAFG00845013826	0	0	0	0
ENSCAFG00845013821	2712	2702	2512	2545
ENSCAFG00845013820	5	3	2	6
ENSCAFG00845013823	2886	2702	2674	2636
ENSCAFG00845013822	982	832	897	848
ENSCAFG00845001829	3	1	1	1
ENSCAFG00845001827	7983	7840	7633	7335
ENSCAFG00845001828	0	0	0	0
ENSCAFG00845001825	0	0	0	0
ENSCAFG00845001826	0	0	0	0
ENSCAFG00845001823	1111	983	1024	1095
ENSCAFG00845001824	0	0	0	0
ENSCAFG00845001821	0	0	0	1
ENSCAFG00845001822	481	421	447	452
ENSCAFG00845001820	1	2	0	3
ENSCAFG00845013818	367	377	346	319
ENSCAFG00845013817	192	169	112	162
ENSCAFG00845013819	0	0	1	1
ENSCAFG00845013814	1772	1766	1719	1644
ENSCAFG00845013813	15	18	12	15

ENSCAFG00845013816	35	17	29	37
ENSCAFG00845013815	2794	2654	2539	2651
ENSCAFG00845013810	1173	1093	1051	1130
ENSCAFG00845013812	166	171	173	152
ENSCAFG00845013811	0	0	0	0
ENSCAFG00845001838	0	0	0	0
ENSCAFG00845001839	0	0	0	0
ENSCAFG00845001836	0	0	0	0
ENSCAFG00845001837	0	0	0	0
ENSCAFG00845001834	0	2	0	0
ENSCAFG00845001835	1	4	4	7
ENSCAFG00845001832	0	0	0	0
ENSCAFG00845001833	0	0	0	0
ENSCAFG00845001830	2	2	1	0
ENSCAFG00845001831	458	397	411	484
ENSCAFG00845013807	52	45	37	29
ENSCAFG00845013806	123	83	91	114
ENSCAFG00845013809	1328	1351	1334	1313
ENSCAFG00845013808	442	428	406	364
ENSCAFG00845013803	477	448	403	505
ENSCAFG00845013802	1825	1620	1690	1700
ENSCAFG00845013805	0	0	0	0
ENSCAFG00845013804	90	70	102	112
ENSCAFG00845013801	0	1	0	0
ENSCAFG00845013800	1488	1453	1479	1542
ENSCAFG00845001807	0	0	0	0
ENSCAFG00845001808	0	0	0	0
ENSCAFG00845001805	1338	1342	1457	1406
ENSCAFG00845001806	979	928	878	941
ENSCAFG00845001803	354	318	399	358
ENSCAFG00845001804	0	0	0	0
ENSCAFG00845001801	3180	3007	3258	3158
ENSCAFG00845001802	0	0	0	0
ENSCAFG00845001800	0	0	0	0
ENSCAFG00845001818	0	0	0	0
ENSCAFG00845001819	0	0	0	0
ENSCAFG00845001816	1780	1758	1543	1606
ENSCAFG00845001817	0	0	0	0
ENSCAFG00845001814	0	0	0	0
ENSCAFG00845001815	3867	3979	3521	3421
ENSCAFG00845001812	691	661	626	560

ENSCAFG00845001813	200	162	127	165
ENSCAFG00845001810	0	0	0	1
ENSCAFG00845001811	0	0	0	0
ENSCAFG00845001809	0	0	0	0
ENSCAFG00845013791	0	0	0	0
ENSCAFG00845013790	0	0	0	0
ENSCAFG00845011130	0	0	0	0
ENSCAFG00845013793	0	0	0	0
ENSCAFG00845011131	847	705	729	705
ENSCAFG00845013792	18	6	11	12
ENSCAFG00845023119	0	0	0	0
ENSCAFG00845023114	5	8	4	2
ENSCAFG00845025777	0	0	0	0
ENSCAFG00845023113	0	0	0	0
ENSCAFG00845025778	3	2	5	4
ENSCAFG00845023112	0	5	2	1
ENSCAFG00845025779	0	0	0	0
ENSCAFG00845023111	0	0	0	0
ENSCAFG00845023118	1	3	0	3
ENSCAFG00845023117	862	888	878	840
ENSCAFG00845025774	3	1	6	3
ENSCAFG00845023116	356	312	290	322
ENSCAFG00845025775	8	5	8	12
ENSCAFG00845023115	22	22	18	16
ENSCAFG00845025776	1	0	0	1
ENSCAFG00845025770	18	18	23	20
ENSCAFG00845025771	0	0	0	0
ENSCAFG00845025772	26	26	37	35
ENSCAFG00845023110	0	0	0	0
ENSCAFG00845011136	360	346	323	325
ENSCAFG00845013799	0	0	0	0
ENSCAFG00845011137	3	2	0	2
ENSCAFG00845013798	5633	5605	5038	5456
ENSCAFG00845011138	1004	961	1094	1088
ENSCAFG00845011139	7	11	9	8
ENSCAFG00845011132	748	752	775	832
ENSCAFG00845013795	942	855	872	842
ENSCAFG00845011133	435	377	451	441
ENSCAFG00845013794	12	11	11	16
ENSCAFG00845011134	4	5	8	2
ENSCAFG00845013797	0	0	0	0

ENSCAFG00845011135	0	0	0	0
ENSCAFG00845013796	984	916	862	966
ENSCAFG00845013780	347	333	348	340
ENSCAFG00845013782	0	0	0	0
ENSCAFG00845023109	0	4	0	0
ENSCAFG00845011120	154	123	137	126
ENSCAFG00845013781	0	3	0	2
ENSCAFG00845023108	54	48	32	54
ENSCAFG00845023103	0	0	0	0
ENSCAFG00845025766	4	4	4	7
ENSCAFG00845023102	0	1	0	2
ENSCAFG00845025767	0	0	0	0
ENSCAFG00845023101	982	826	858	827
ENSCAFG00845025768	34	55	62	45
ENSCAFG00845023100	596	559	644	603
ENSCAFG00845025769	0	0	0	0
ENSCAFG00845023107	33	29	33	29
ENSCAFG00845025762	1182	1270	1037	1159
ENSCAFG00845023106	806	769	833	781
ENSCAFG00845025763	0	0	0	0
ENSCAFG00845023105	20	13	22	32
ENSCAFG00845025764	25	25	16	24
ENSCAFG00845023104	138	135	144	139
ENSCAFG00845025765	617	502	523	536
ENSCAFG00845025760	7	8	10	8
ENSCAFG00845025761	382	390	398	316
ENSCAFG00845011129	7	5	3	8
ENSCAFG00845011125	72	54	99	91
ENSCAFG00845013788	336	348	308	365
ENSCAFG00845011126	4	4	4	5
ENSCAFG00845013787	1470	1466	1401	1517
ENSCAFG00845011127	15	11	6	12
ENSCAFG00845011128	0	0	0	0
ENSCAFG00845013789	0	0	0	0
ENSCAFG00845011121	0	0	0	0
ENSCAFG00845013784	0	0	0	0
ENSCAFG00845011122	716	751	674	643
ENSCAFG00845013783	0	0	0	0
ENSCAFG00845011123	32	24	19	23
ENSCAFG00845013786	0	0	0	0
ENSCAFG00845011124	4	9	6	5

ENSCAFG00845013785	2447	2606	2399	2482
ENSCAFG00845013771	82	86	103	98
ENSCAFG00845013770	2191	2094	1976	2102
ENSCAFG00845025759	0	0	0	0
ENSCAFG00845001788	6	6	1	12
ENSCAFG00845025755	1	0	0	1
ENSCAFG00845001789	0	0	0	0
ENSCAFG00845025756	1	1	2	0
ENSCAFG00845001786	0	0	0	0
ENSCAFG00845025757	5	1	1	0
ENSCAFG00845001787	0	0	0	0
ENSCAFG00845025758	2406	2267	2237	2213
ENSCAFG00845001784	0	0	0	0
ENSCAFG00845025751	0	0	0	0
ENSCAFG00845001785	0	0	0	0
ENSCAFG00845025752	0	0	0	0
ENSCAFG00845001782	153	158	205	205
ENSCAFG00845025753	0	0	0	0
ENSCAFG00845001783	0	0	0	0
ENSCAFG00845025754	1057	941	867	861
ENSCAFG00845001780	0	0	0	0
ENSCAFG00845001781	2	1	0	0
ENSCAFG00845025750	9849	9252	10901	11302
ENSCAFG00845011118	4	6	9	7
ENSCAFG00845011119	0	0	0	0
ENSCAFG00845011114	0	0	0	0
ENSCAFG00845013777	17	14	15	9
ENSCAFG00845011115	0	0	1	0
ENSCAFG00845013776	262	289	307	357
ENSCAFG00845011116	82	58	60	88
ENSCAFG00845013779	246	218	233	252
ENSCAFG00845011117	1303	1253	1630	1579
ENSCAFG00845013778	2917	2774	2985	2858
ENSCAFG00845011110	0	0	0	0
ENSCAFG00845013773	80	51	69	62
ENSCAFG00845011111	793	724	715	759
ENSCAFG00845013772	1	1	2	0
ENSCAFG00845011112	0	0	0	0
ENSCAFG00845013775	0	0	0	0
ENSCAFG00845011113	285	293	246	262
ENSCAFG00845013774	6	6	9	5

ENSCAFG00845013760	0	0	0	0
ENSCAFG00845025748	10	21	24	17
ENSCAFG00845025749	0	3	2	0
ENSCAFG00845001799	0	0	0	0
ENSCAFG00845025744	0	0	0	0
ENSCAFG00845025745	762	704	873	843
ENSCAFG00845001797	0	0	0	0
ENSCAFG00845025746	65	72	71	68
ENSCAFG00845001798	3676	3531	3912	3922
ENSCAFG00845025747	0	1	0	0
ENSCAFG00845001795	0	0	0	0
ENSCAFG00845025740	25	21	40	17
ENSCAFG00845001796	0	0	0	0
ENSCAFG00845025741	0	0	0	0
ENSCAFG00845001793	3471	3213	2855	2844
ENSCAFG00845025742	0	0	0	0
ENSCAFG00845001794	0	0	0	0
ENSCAFG00845025743	1	1	0	0
ENSCAFG00845001791	5	2	3	3
ENSCAFG00845001792	0	0	0	0
ENSCAFG00845001790	594	640	657	650
ENSCAFG00845011107	142	153	155	153
ENSCAFG00845011108	0	0	0	0
ENSCAFG00845013769	3222	3134	3234	3276
ENSCAFG00845011109	26	20	19	9
ENSCAFG00845011103	0	0	0	0
ENSCAFG00845013766	824	763	975	957
ENSCAFG00845011104	0	0	0	0
ENSCAFG00845013765	0	0	0	0
ENSCAFG00845011105	194	258	175	173
ENSCAFG00845013768	331	311	309	284
ENSCAFG00845011106	0	0	0	0
ENSCAFG00845013767	219	219	210	183
ENSCAFG00845013762	5623	5321	4766	4750
ENSCAFG00845011100	0	0	0	0
ENSCAFG00845013761	454	455	456	418
ENSCAFG00845011101	0	0	0	0
ENSCAFG00845013764	525	557	492	559
ENSCAFG00845011102	435	445	460	490
ENSCAFG00845013763	726	654	677	684
ENSCAFG00845025737	482	390	396	409

ENSCAFG00845025738	445	419	547	496
ENSCAFG00845001768	129	153	130	125
ENSCAFG00845025739	4	3	3	1
ENSCAFG00845001769	17	15	20	11
ENSCAFG00845001766	3491	3235	4030	4082
ENSCAFG00845025733	247	209	228	254
ENSCAFG00845001767	0	0	0	0
ENSCAFG00845025734	0	0	0	0
ENSCAFG00845001764	0	0	2	2
ENSCAFG00845025735	0	0	0	0
ENSCAFG00845001765	2	2	0	2
ENSCAFG00845025736	0	0	0	0
ENSCAFG00845001762	0	0	0	0
ENSCAFG00845001763	0	0	0	0
ENSCAFG00845025730	820	753	775	895
ENSCAFG00845001760	0	0	0	0
ENSCAFG00845025731	1459	1280	1504	1545
ENSCAFG00845001761	0	0	0	0
ENSCAFG00845025732	0	0	0	0
ENSCAFG00845013759	2	0	2	0
ENSCAFG00845013758	519	474	475	533
ENSCAFG00845013755	0	0	0	0
ENSCAFG00845013754	9	8	10	12
ENSCAFG00845013757	20	22	11	19
ENSCAFG00845013756	0	0	0	0
ENSCAFG00845013751	650	625	608	608
ENSCAFG00845013750	1	0	0	2
ENSCAFG00845013753	0	0	0	0
ENSCAFG00845013752	1	1	0	0
ENSCAFG00845025726	0	0	0	0
ENSCAFG00845025727	209	225	191	211
ENSCAFG00845001779	0	0	1	0
ENSCAFG00845025728	0	0	0	0
ENSCAFG00845025729	1433	1261	1340	1260
ENSCAFG00845001777	2559	2494	2601	2604
ENSCAFG00845025722	1330	1203	1397	1443
ENSCAFG00845001778	768	648	625	618
ENSCAFG00845025723	974	1024	1027	979
ENSCAFG00845001775	2	0	0	5
ENSCAFG00845025724	1197	1252	1186	1187
ENSCAFG00845001776	0	0	0	0

ENSCAFG00845001773	426	437	457	478
ENSCAFG00845001774	0	0	0	0
ENSCAFG00845001771	625	594	622	629
ENSCAFG00845025720	2	9	5	1
ENSCAFG00845001772	2	2	2	0
ENSCAFG00845025721	1088	1137	1132	1156
ENSCAFG00845001770	0	0	0	0
ENSCAFG00845013748	1	1	1	0
ENSCAFG00845013747	1	2	0	0
ENSCAFG00845013749	2	3	2	0
ENSCAFG00845013744	2443	2387	2438	2591
ENSCAFG00845013743	994	896	994	995
ENSCAFG00845013746	0	0	0	0
ENSCAFG00845013745	1	0	3	0
ENSCAFG00845013740	0	0	0	0
ENSCAFG00845013742	194	177	187	242
ENSCAFG00845013741	0	0	0	0
ENSCAFG00845025719	0	0	0	0
ENSCAFG00845001748	186	173	223	193
ENSCAFG00845025715	4	3	6	4
ENSCAFG00845001749	0	0	0	0
ENSCAFG00845025716	0	0	0	0
ENSCAFG00845001746	1216	1285	1313	1390
ENSCAFG00845025717	0	0	0	1
ENSCAFG00845001747	2	0	0	4
ENSCAFG00845025718	0	0	0	0
ENSCAFG00845001744	0	0	0	0
ENSCAFG00845025711	671	538	469	522
ENSCAFG00845001745	0	0	0	0
ENSCAFG00845025712	0	0	0	0
ENSCAFG00845001742	992	856	907	953
ENSCAFG00845025713	1	1	2	0
ENSCAFG00845001743	0	0	0	0
ENSCAFG00845025714	0	0	0	0
ENSCAFG00845001740	0	0	0	0
ENSCAFG00845001741	0	0	0	0
ENSCAFG00845025710	74	65	87	50
ENSCAFG00845013737	2	0	0	0
ENSCAFG00845013736	7	13	2	10
ENSCAFG00845013739	336	313	276	280
ENSCAFG00845013738	1027	988	629	648

ENSCAFG00845013733	2289	2329	2353	2238
ENSCAFG00845013732	0	0	0	0
ENSCAFG00845013735	2	0	5	4
ENSCAFG00845013734	1	5	2	2
ENSCAFG00845013731	0	0	0	2
ENSCAFG00845013730	101	75	130	81
ENSCAFG00845025708	0	0	0	0
ENSCAFG00845025709	754	784	858	745
ENSCAFG00845001759	0	1	0	0
ENSCAFG00845025704	0	0	0	0
ENSCAFG00845025705	1265	1381	1340	1399
ENSCAFG00845001757	0	0	0	0
ENSCAFG00845025706	41	32	50	57
ENSCAFG00845001758	6173	6107	6336	6467
ENSCAFG00845025707	1	0	0	0
ENSCAFG00845001755	242	244	249	281
ENSCAFG00845025700	0	0	0	0
ENSCAFG00845001756	0	0	0	0
ENSCAFG00845025701	0	0	0	0
ENSCAFG00845001753	0	0	0	0
ENSCAFG00845025702	0	0	1	0
ENSCAFG00845001754	731	681	785	716
ENSCAFG00845025703	0	0	0	0
ENSCAFG00845001751	1540	1413	1517	1623
ENSCAFG00845001752	3870	3615	3876	3972
ENSCAFG00845001750	1089	1088	1005	973
ENSCAFG00845013729	3277	3267	3480	3458
ENSCAFG00845013726	86	52	54	53
ENSCAFG00845013725	877	741	1052	959
ENSCAFG00845013728	0	0	0	0
ENSCAFG00845013727	0	0	0	0
ENSCAFG00845013722	433	422	426	391
ENSCAFG00845013721	13	12	12	7
ENSCAFG00845013724	6	4	5	5
ENSCAFG00845013723	267	233	258	250
ENSCAFG00845013720	0	0	0	0
ENSCAFG00845023199	31	30	24	24
ENSCAFG00845023194	0	3	0	0
ENSCAFG00845023193	0	0	0	0
ENSCAFG00845023192	3969	3796	2970	2748
ENSCAFG00845023191	0	1	3	1

ENSCAFG00845023198	43	67	74	65
ENSCAFG00845023197	561	566	553	576
ENSCAFG00845023196	3	0	0	0
ENSCAFG00845023195	556	569	432	451
ENSCAFG00845023190	2699	2410	2579	2731
ENSCAFG00845023189	237	234	204	260
ENSCAFG00845023188	766	776	872	928
ENSCAFG00845023183	333	370	329	357
ENSCAFG00845023182	0	0	0	0
ENSCAFG00845023181	0	0	0	0
ENSCAFG00845023180	2189	2024	1880	1926
ENSCAFG00845023187	842	711	747	708
ENSCAFG00845023186	0	0	0	0
ENSCAFG00845023185	5	0	0	1
ENSCAFG00845023184	1104	1195	1082	1138
ENSCAFG00845011194	3	1	0	0
ENSCAFG00845011195	278	265	301	314
ENSCAFG00845011196	13	18	9	14
ENSCAFG00845011197	0	0	0	0
ENSCAFG00845011190	0	0	0	0
ENSCAFG00845011191	8	13	20	11
ENSCAFG00845011192	1653	1518	1549	1597
ENSCAFG00845011193	0	0	0	0
ENSCAFG00845023179	0	0	0	0
ENSCAFG00845023178	1	1	3	1
ENSCAFG00845023177	11	11	12	18
ENSCAFG00845023172	2	1	2	4
ENSCAFG00845023171	342	325	261	300
ENSCAFG00845023170	68	76	50	55
ENSCAFG00845023176	49	34	46	38
ENSCAFG00845023175	352	337	387	376
ENSCAFG00845023174	10	17	19	12
ENSCAFG00845023173	4515	4353	4174	4296
ENSCAFG00845011198	953	910	732	848
ENSCAFG00845011199	229	177	156	182
ENSCAFG00845011183	1186	1182	1111	1180
ENSCAFG00845011184	4	4	1	5
ENSCAFG00845011185	2821	2801	2513	2669
ENSCAFG00845011186	0	0	0	0
ENSCAFG00845011180	0	1	1	0
ENSCAFG00845011181	0	0	0	0

ENSCAFG00845011182	0	1	0	0
ENSCAFG00845023169	0	1	1	4
ENSCAFG00845023168	270	210	252	235
ENSCAFG00845023167	0	0	0	0
ENSCAFG00845023166	720	615	628	601
ENSCAFG00845023161	45	21	28	46
ENSCAFG00845023160	0	1	6	3
ENSCAFG00845023164	0	0	0	0
ENSCAFG00845023163	685	685	557	645
ENSCAFG00845023162	0	0	0	0
ENSCAFG00845011187	202	223	227	191
ENSCAFG00845011188	2648	2590	2542	2453
ENSCAFG00845011189	0	0	0	0
ENSCAFG00845011172	1691	1583	1621	1668
ENSCAFG00845011173	2	4	10	6
ENSCAFG00845011174	3831	3575	3737	3492
ENSCAFG00845011175	4053	3796	3845	3945
ENSCAFG00845011170	540	519	499	513
ENSCAFG00845011171	1295	1310	1241	1325
ENSCAFG00845023158	0	0	0	0
ENSCAFG00845023157	5	10	3	1
ENSCAFG00845023156	3931	3774	4153	4474
ENSCAFG00845023155	4	8	14	21
ENSCAFG00845023159	76	88	99	97
ENSCAFG00845023150	0	0	0	0
ENSCAFG00845023154	0	0	0	0
ENSCAFG00845023153	0	0	0	0
ENSCAFG00845023152	0	0	0	0
ENSCAFG00845023151	2	0	1	0
ENSCAFG00845011176	0	0	0	0
ENSCAFG00845011177	0	0	0	0
ENSCAFG00845011178	5	1	0	0
ENSCAFG00845011179	0	1	1	1
ENSCAFG00845011161	1893	1804	1822	1868
ENSCAFG00845011162	0	0	0	0
ENSCAFG00845011163	174	216	194	209
ENSCAFG00845011164	23	37	17	25
ENSCAFG00845011160	5	5	1	1
ENSCAFG00845023147	766	815	804	866
ENSCAFG00845023146	3	12	3	6
ENSCAFG00845023145	485	516	558	559

ENSCAFG00845023144	7	10	17	2
ENSCAFG00845023149	0	0	0	0
ENSCAFG00845023148	0	0	0	0
ENSCAFG00845023143	451	476	470	483
ENSCAFG00845023142	0	6	1	5
ENSCAFG00845023141	413	463	440	465
ENSCAFG00845023140	7228	6785	7466	7725
ENSCAFG00845011169	38	39	56	43
ENSCAFG00845011165	0	0	5	7
ENSCAFG00845011166	624	596	647	644
ENSCAFG00845011167	890	917	933	998
ENSCAFG00845011168	0	0	0	0
ENSCAFG00845011150	3092	2951	2717	2757
ENSCAFG00845011151	293	293	355	349
ENSCAFG00845011152	1	0	0	1
ENSCAFG00845011153	13	13	14	17
ENSCAFG00845025799	0	0	0	0
ENSCAFG00845023135	0	1	0	1
ENSCAFG00845023134	0	0	0	0
ENSCAFG00845023133	0	0	0	0
ENSCAFG00845025795	0	0	1	0
ENSCAFG00845023139	304	295	219	217
ENSCAFG00845025796	42	32	43	42
ENSCAFG00845023138	252	248	264	259
ENSCAFG00845025797	2150	1990	2138	2101
ENSCAFG00845023137	0	1	0	0
ENSCAFG00845025798	0	0	0	0
ENSCAFG00845025792	0	0	0	0
ENSCAFG00845025793	103	75	45	64
ENSCAFG00845025794	0	0	0	0
ENSCAFG00845023132	11	5	3	8
ENSCAFG00845023131	4	4	5	0
ENSCAFG00845023130	49	71	67	67
ENSCAFG00845025790	2584	2480	2546	2682
ENSCAFG00845011158	29	36	17	33
ENSCAFG00845011159	0	1	0	0
ENSCAFG00845011154	2	0	0	0
ENSCAFG00845011155	0	2	2	2
ENSCAFG00845011156	0	0	0	0
ENSCAFG00845011157	0	0	0	0
ENSCAFG00845011140	4	6	3	1

ENSCAFG00845011141	0	0	0	0
ENSCAFG00845011142	2365	2212	2137	2178
ENSCAFG00845023125	209	200	236	223
ENSCAFG00845023124	0	0	1	1
ENSCAFG00845025789	3	8	4	5
ENSCAFG00845023123	255	253	262	279
ENSCAFG00845023122	1787	1790	1799	1897
ENSCAFG00845023129	0	0	0	0
ENSCAFG00845025784	0	0	0	0
ENSCAFG00845023128	23	18	19	30
ENSCAFG00845025785	0	0	0	0
ENSCAFG00845023127	0	0	0	0
ENSCAFG00845025786	288	294	217	240
ENSCAFG00845023126	0	0	0	0
ENSCAFG00845025787	0	0	0	0
ENSCAFG00845025780	0	0	0	0
ENSCAFG00845025781	0	0	0	0
ENSCAFG00845025782	27	24	32	41
ENSCAFG00845025783	238	245	236	254
ENSCAFG00845023121	594	535	584	616
ENSCAFG00845023120	0	0	0	0
ENSCAFG00845011147	830	855	1017	957
ENSCAFG00845011148	0	0	0	0
ENSCAFG00845011149	2	13	5	4
ENSCAFG00845011143	313	249	335	287
ENSCAFG00845011144	1892	1710	1828	1952
ENSCAFG00845011145	6291	6033	4141	4130
ENSCAFG00845011146	0	0	0	0
ENSCAFG00845025939	0	0	0	0
ENSCAFG00845001968	2	0	0	0
ENSCAFG00845025935	0	0	0	0
ENSCAFG00845001969	0	0	0	0
ENSCAFG00845025936	3	0	2	4
ENSCAFG00845001966	1	0	2	0
ENSCAFG00845001967	904	940	944	951
ENSCAFG00845025938	936	875	931	952
ENSCAFG00845001964	0	0	1	0
ENSCAFG00845025931	0	0	0	0
ENSCAFG00845001965	134	134	91	121
ENSCAFG00845025932	754	777	683	777
ENSCAFG00845001962	274	232	201	210

ENSCAFG00845025933	8	6	5	9
ENSCAFG00845001963	0	0	0	0
ENSCAFG00845025934	109	79	87	111
ENSCAFG00845001960	3	0	0	0
ENSCAFG00845001961	487	443	357	445
ENSCAFG00845025930	0	0	0	0
ENSCAFG00845013957	203	187	220	219
ENSCAFG00845013956	200	196	253	203
ENSCAFG00845013959	177	157	163	199
ENSCAFG00845013958	31	16	34	19
ENSCAFG00845013953	1	0	0	0
ENSCAFG00845013952	2064	1892	2003	2126
ENSCAFG00845013955	209	207	250	232
ENSCAFG00845013954	0	1	1	1
ENSCAFG00845013951	0	0	0	0
ENSCAFG00845013950	0	0	0	0
ENSCAFG00845025928	0	0	0	0
ENSCAFG00845025929	2	0	3	1
ENSCAFG00845001979	888	804	855	828
ENSCAFG00845025924	1312	1419	1393	1316
ENSCAFG00845001977	653	699	724	716
ENSCAFG00845025926	0	0	0	0
ENSCAFG00845001978	0	0	0	0
ENSCAFG00845025927	0	0	0	0
ENSCAFG00845001975	2	3	3	1
ENSCAFG00845025920	0	0	0	0
ENSCAFG00845001976	885	829	822	822
ENSCAFG00845025921	929	823	880	800
ENSCAFG00845001973	2	1	3	0
ENSCAFG00845025922	3	4	1	0
ENSCAFG00845001974	0	0	0	0
ENSCAFG00845025923	4	0	2	0
ENSCAFG00845001971	0	0	0	0
ENSCAFG00845001972	0	0	0	0
ENSCAFG00845001970	2	5	2	0
ENSCAFG00845013949	0	1	0	0
ENSCAFG00845013946	2043	1936	2094	2078
ENSCAFG00845013945	6189	5791	5473	5570
ENSCAFG00845013948	0	0	0	0
ENSCAFG00845013947	68	63	57	67
ENSCAFG00845013942	4	10	4	3

ENSCAFG00845013941	0	0	0	0
ENSCAFG00845013944	0	0	0	0
ENSCAFG00845013943	0	0	2	0
ENSCAFG00845013940	0	0	0	0
ENSCAFG00845025917	1204	1249	1250	1301
ENSCAFG00845025918	5544	5542	4921	4992
ENSCAFG00845001948	232	240	248	233
ENSCAFG00845025919	323	368	296	282
ENSCAFG00845001949	1071	1011	1216	1277
ENSCAFG00845001946	0	0	0	2
ENSCAFG00845025913	8	9	21	8
ENSCAFG00845001947	0	0	0	0
ENSCAFG00845025914	2910	2677	2805	2859
ENSCAFG00845001944	0	0	0	0
ENSCAFG00845025915	249	263	255	243
ENSCAFG00845001945	0	0	0	0
ENSCAFG00845025916	44	37	48	63
ENSCAFG00845001942	0	0	0	0
ENSCAFG00845001943	0	0	1	0
ENSCAFG00845025910	0	0	2	0
ENSCAFG00845001940	116	121	104	127
ENSCAFG00845025911	1209	1094	1174	1383
ENSCAFG00845001941	368	306	314	355
ENSCAFG00845025912	0	0	0	0
ENSCAFG00845013939	503	504	497	491
ENSCAFG00845013938	0	0	0	0
ENSCAFG00845013935	0	0	0	0
ENSCAFG00845013934	0	0	0	0
ENSCAFG00845013937	99	105	127	96
ENSCAFG00845013936	228	174	192	199
ENSCAFG00845013931	992	879	879	1073
ENSCAFG00845013930	8	7	3	6
ENSCAFG00845013933	1469	1354	1940	1941
ENSCAFG00845013932	678	628	598	645
ENSCAFG00845025907	113	122	77	82
ENSCAFG00845001959	0	1	0	0
ENSCAFG00845025908	0	0	0	0
ENSCAFG00845025909	0	0	0	0
ENSCAFG00845001957	0	0	0	0
ENSCAFG00845025902	7	10	8	9
ENSCAFG00845001958	0	0	0	0

ENSCAFG00845025903	114	111	75	83
ENSCAFG00845001955	0	0	0	0
ENSCAFG00845025904	0	0	0	0
ENSCAFG00845001956	2	0	3	1
ENSCAFG00845025905	189	179	176	173
ENSCAFG00845001953	0	0	0	0
ENSCAFG00845001954	2	3	0	1
ENSCAFG00845001951	36	28	45	27
ENSCAFG00845001952	0	0	0	0
ENSCAFG00845025901	0	0	0	0
ENSCAFG00845001950	3	0	0	0
ENSCAFG00845013928	1	0	4	2
ENSCAFG00845013927	123	130	93	110
ENSCAFG00845013929	268	261	311	253
ENSCAFG00845013924	2082	2021	1774	1963
ENSCAFG00845013923	592	604	580	576
ENSCAFG00845013926	423	416	495	482
ENSCAFG00845013925	1	1	0	1
ENSCAFG00845013920	1077	1057	889	942
ENSCAFG00845013922	4448	4297	4237	4296
ENSCAFG00845013921	2	8	5	5
ENSCAFG00845001928	0	0	0	0
ENSCAFG00845001929	753	714	712	771
ENSCAFG00845001926	0	0	0	0
ENSCAFG00845001927	404	347	375	355
ENSCAFG00845001924	0	0	0	0
ENSCAFG00845001925	1767	1671	1868	1874
ENSCAFG00845001922	8	7	13	4
ENSCAFG00845001923	4	11	6	9
ENSCAFG00845001920	104	91	108	91
ENSCAFG00845001921	0	0	0	0
ENSCAFG00845013909	3	0	0	6
ENSCAFG00845013917	2	5	2	3
ENSCAFG00845013916	2	12	9	7
ENSCAFG00845013919	129	147	160	167
ENSCAFG00845013918	115	97	141	117
ENSCAFG00845013913	0	6	1	1
ENSCAFG00845013912	4	4	2	4
ENSCAFG00845013915	0	0	0	0
ENSCAFG00845013914	48	40	52	66
ENSCAFG00845013911	24	18	37	20

ENSCAFG00845013910	87	114	122	130
ENSCAFG00845001919	1849	1657	1696	1681
ENSCAFG00845001939	1	1	0	0
ENSCAFG00845001937	0	0	0	0
ENSCAFG00845001938	1324	1265	1217	1328
ENSCAFG00845001935	7	3	5	2
ENSCAFG00845001936	113	88	78	87
ENSCAFG00845001933	22	16	27	20
ENSCAFG00845001934	2673	2439	2435	2350
ENSCAFG00845001931	1	0	1	0
ENSCAFG00845001932	40	42	43	44
ENSCAFG00845001930	0	2	0	0
ENSCAFG00845013906	11	6	14	7
ENSCAFG00845013905	292	347	257	282
ENSCAFG00845013908	0	0	0	0
ENSCAFG00845013907	779	734	782	777
ENSCAFG00845013902	1304	1155	1245	1246
ENSCAFG00845013901	419	420	375	391
ENSCAFG00845013904	0	0	0	0
ENSCAFG00845013903	0	0	0	0
ENSCAFG00845013900	0	0	0	0
ENSCAFG00845001906	16	19	11	14
ENSCAFG00845001907	15	22	47	43
ENSCAFG00845001904	5839	5637	5968	6084
ENSCAFG00845001905	0	0	0	0
ENSCAFG00845001902	325	293	242	264
ENSCAFG00845001903	0	0	0	0
ENSCAFG00845001900	0	0	0	0
ENSCAFG00845001901	1	0	0	2
ENSCAFG00845001917	420	447	413	433
ENSCAFG00845001918	17	14	17	20
ENSCAFG00845001915	2840	2834	2470	2467
ENSCAFG00845001916	188	176	207	186
ENSCAFG00845001913	461	436	431	430
ENSCAFG00845001914	2295	2245	2012	2054
ENSCAFG00845001911	787	783	770	779
ENSCAFG00845001912	390	448	401	361
ENSCAFG00845001910	0	0	0	0
ENSCAFG00845001908	2083	1976	2397	2529
ENSCAFG00845001909	156	192	75	65
ENSCAFG00845011250	5	3	8	6

ENSCAFG00845011251	139	128	135	143
ENSCAFG00845011252	0	0	0	0
ENSCAFG00845023235	199	191	160	177
ENSCAFG00845025898	209	217	160	160
ENSCAFG00845023234	0	1	0	0
ENSCAFG00845025899	1060	991	1090	1055
ENSCAFG00845023233	0	0	0	0
ENSCAFG00845023232	4810	4646	4977	4928
ENSCAFG00845023239	31	35	31	33
ENSCAFG00845025894	0	4	1	4
ENSCAFG00845023238	0	0	0	0
ENSCAFG00845025895	0	0	0	0
ENSCAFG00845023237	1	3	0	0
ENSCAFG00845025896	1	0	0	3
ENSCAFG00845023236	0	0	0	0
ENSCAFG00845025897	0	0	0	0
ENSCAFG00845025890	1	0	0	0
ENSCAFG00845025891	3	0	4	1
ENSCAFG00845025892	10	4	5	7
ENSCAFG00845025893	2	0	1	0
ENSCAFG00845023231	142	146	160	100
ENSCAFG00845023230	0	0	0	0
ENSCAFG00845011257	0	0	2	0
ENSCAFG00845011258	20	18	11	15
ENSCAFG00845011259	0	0	0	0
ENSCAFG00845011253	0	0	0	0
ENSCAFG00845011254	0	0	0	0
ENSCAFG00845011255	0	0	0	1
ENSCAFG00845011256	0	0	0	0
ENSCAFG00845011240	0	0	0	0
ENSCAFG00845011241	1	0	0	0
ENSCAFG00845023229	0	0	1	2
ENSCAFG00845023224	0	0	0	0
ENSCAFG00845025887	1112	1100	1144	1185
ENSCAFG00845023223	3852	3652	3499	3417
ENSCAFG00845025888	20	14	12	18
ENSCAFG00845023222	21	11	9	12
ENSCAFG00845025889	0	0	0	0
ENSCAFG00845023221	23	34	34	44
ENSCAFG00845023228	835	817	852	798
ENSCAFG00845025883	335	337	373	438

ENSCAFG00845023227	0	1	0	0
ENSCAFG00845025884	326	324	369	365
ENSCAFG00845023226	6634	6608	7199	6856
ENSCAFG00845025885	8	15	11	10
ENSCAFG00845023225	12	9	23	14
ENSCAFG00845025886	53	43	42	44
ENSCAFG00845025880	8	3	5	8
ENSCAFG00845025881	0	0	0	0
ENSCAFG00845025882	2	0	0	0
ENSCAFG00845023220	41	32	43	22
ENSCAFG00845011246	0	0	0	0
ENSCAFG00845011247	0	0	0	0
ENSCAFG00845011248	0	0	0	0
ENSCAFG00845011249	0	0	0	0
ENSCAFG00845011242	701	617	712	683
ENSCAFG00845011243	2	0	0	0
ENSCAFG00845011244	0	0	0	0
ENSCAFG00845011245	1107	1144	1209	1320
ENSCAFG00845013890	0	0	0	0
ENSCAFG00845013892	70303	67653	77105	75545
ENSCAFG00845023219	698	630	619	702
ENSCAFG00845011230	0	0	4	0
ENSCAFG00845013891	0	0	0	0
ENSCAFG00845023218	450	390	456	531
ENSCAFG00845023213	1	9	0	4
ENSCAFG00845025876	20	5	12	6
ENSCAFG00845023212	0	0	0	0
ENSCAFG00845025877	0	0	0	0
ENSCAFG00845023211	0	0	0	1
ENSCAFG00845023210	0	0	0	0
ENSCAFG00845025879	0	0	0	0
ENSCAFG00845023217	46	59	52	50
ENSCAFG00845025872	222	226	216	189
ENSCAFG00845023216	1500	1578	1576	1500
ENSCAFG00845025873	1921	1874	1485	1395
ENSCAFG00845023215	0	1	2	0
ENSCAFG00845023214	1	0	0	0
ENSCAFG00845025875	15	13	5	9
ENSCAFG00845025870	11	10	1	4
ENSCAFG00845025871	129	113	102	110
ENSCAFG00845011239	7	2	7	5

ENSCAFG00845011235	444	392	461	509
ENSCAFG00845013898	0	0	0	0
ENSCAFG00845011236	547	476	510	515
ENSCAFG00845013897	0	0	2	2
ENSCAFG00845011237	3962	3870	3504	3456
ENSCAFG00845011238	452	412	463	476
ENSCAFG00845013899	2546	2426	2572	2501
ENSCAFG00845011231	0	0	0	0
ENSCAFG00845013894	99	102	85	85
ENSCAFG00845011232	0	0	0	1
ENSCAFG00845013893	1	1	0	0
ENSCAFG00845011233	0	0	0	0
ENSCAFG00845013896	755	753	824	699
ENSCAFG00845011234	260	270	233	253
ENSCAFG00845013895	5	13	12	15
ENSCAFG00845023209	0	0	0	0
ENSCAFG00845013881	0	4	1	0
ENSCAFG00845023208	384	339	370	339
ENSCAFG00845013880	1	0	0	0
ENSCAFG00845023207	0	0	0	0
ENSCAFG00845025869	0	0	0	0
ENSCAFG00845023202	0	0	0	0
ENSCAFG00845025865	94	118	111	126
ENSCAFG00845023201	4	7	10	9
ENSCAFG00845025866	0	0	0	0
ENSCAFG00845023200	0	0	0	0
ENSCAFG00845025867	3637	3301	2892	2979
ENSCAFG00845025868	0	2	0	2
ENSCAFG00845023206	942	896	974	947
ENSCAFG00845025861	2	8	1	0
ENSCAFG00845023205	1527	1502	1523	1493
ENSCAFG00845025862	426	358	366	391
ENSCAFG00845023204	0	1	2	0
ENSCAFG00845025863	492	460	405	455
ENSCAFG00845025864	0	0	0	0
ENSCAFG00845025860	0	0	0	0
ENSCAFG00845011228	3	0	3	1
ENSCAFG00845011229	0	0	0	0
ENSCAFG00845011224	0	0	0	0
ENSCAFG00845013887	0	0	0	0
ENSCAFG00845011225	3	1	9	2

ENSCAFG00845013886	4	9	15	25
ENSCAFG00845011226	135	115	122	125
ENSCAFG00845013889	225	183	172	190
ENSCAFG00845011227	834	789	949	886
ENSCAFG00845013888	0	0	0	0
ENSCAFG00845011220	8	3	4	1
ENSCAFG00845013883	120	108	107	87
ENSCAFG00845011221	30	28	30	34
ENSCAFG00845013882	570	518	599	545
ENSCAFG00845011222	64	59	66	54
ENSCAFG00845013885	0	0	0	0
ENSCAFG00845011223	0	0	0	0
ENSCAFG00845013884	0	1	0	0
ENSCAFG00845013870	427	349	363	359
ENSCAFG00845025858	463	431	379	503
ENSCAFG00845025859	309	293	271	272
ENSCAFG00845001889	44	26	41	29
ENSCAFG00845001887	3	5	5	4
ENSCAFG00845025854	2956	2941	2757	2635
ENSCAFG00845001888	3	0	1	0
ENSCAFG00845025855	0	0	0	0
ENSCAFG00845001885	502	471	467	469
ENSCAFG00845025856	22	15	14	12
ENSCAFG00845001886	27	38	32	29
ENSCAFG00845025857	601	519	581	620
ENSCAFG00845001883	1404	1292	1386	1447
ENSCAFG00845025850	20	24	15	19
ENSCAFG00845001884	0	0	0	0
ENSCAFG00845025851	0	0	0	0
ENSCAFG00845001881	23	20	7	16
ENSCAFG00845025852	2346	2423	2694	2737
ENSCAFG00845001882	1216	1115	1192	1279
ENSCAFG00845025853	42	51	60	45
ENSCAFG00845001880	0	0	0	0
ENSCAFG00845011217	1	0	0	2
ENSCAFG00845011218	0	0	0	0
ENSCAFG00845013879	160	127	118	155
ENSCAFG00845011219	91	115	117	86
ENSCAFG00845011213	13	15	16	22
ENSCAFG00845013876	0	0	0	0
ENSCAFG00845011214	0	1	0	0

ENSCAFG00845013875	670	650	674	656
ENSCAFG00845011215	2460	2330	2852	3087
ENSCAFG00845013878	0	0	0	0
ENSCAFG00845011216	1101	1083	1166	1181
ENSCAFG00845013877	0	0	0	0
ENSCAFG00845013872	0	0	0	0
ENSCAFG00845011210	222	172	212	179
ENSCAFG00845013871	0	0	0	0
ENSCAFG00845011211	25	17	16	15
ENSCAFG00845013874	460	408	465	457
ENSCAFG00845011212	0	0	0	0
ENSCAFG00845013873	267	239	287	262
ENSCAFG00845025847	1483	1399	1350	1408
ENSCAFG00845025848	1170	1033	1133	1213
ENSCAFG00845025849	931	903	824	936
ENSCAFG00845001898	4	1	5	2
ENSCAFG00845025843	222	212	201	215
ENSCAFG00845001899	0	0	0	0
ENSCAFG00845025844	27	35	32	43
ENSCAFG00845001896	8	14	3	14
ENSCAFG00845025845	74	80	133	172
ENSCAFG00845001897	3	6	6	6
ENSCAFG00845001894	0	0	0	0
ENSCAFG00845001895	86	69	86	88
ENSCAFG00845025840	0	0	0	0
ENSCAFG00845001892	0	0	0	0
ENSCAFG00845001893	0	0	0	0
ENSCAFG00845025842	0	0	0	0
ENSCAFG00845001890	0	0	0	0
ENSCAFG00845001891	269	284	249	248
ENSCAFG00845011206	0	0	0	1
ENSCAFG00845013869	691	730	623	628
ENSCAFG00845011207	7	5	4	8
ENSCAFG00845013868	0	0	1	0
ENSCAFG00845011208	0	0	0	0
ENSCAFG00845011209	0	0	0	0
ENSCAFG00845011202	0	0	0	0
ENSCAFG00845013865	1034	1036	914	988
ENSCAFG00845011203	0	0	0	0
ENSCAFG00845013864	3	0	3	0
ENSCAFG00845011204	1045	1116	1111	1053

ENSCAFG00845013867	0	0	0	0
ENSCAFG00845011205	917	852	867	894
ENSCAFG00845013866	31	37	41	36
ENSCAFG00845013861	21	23	20	20
ENSCAFG00845013860	69	74	89	97
ENSCAFG00845011200	0	0	0	0
ENSCAFG00845013863	0	0	0	1
ENSCAFG00845011201	0	0	0	0
ENSCAFG00845013862	245	260	221	205
ENSCAFG00845001869	0	0	0	0
ENSCAFG00845025836	76	67	71	78
ENSCAFG00845025837	3	2	11	8
ENSCAFG00845001867	4	7	2	3
ENSCAFG00845025838	0	0	0	0
ENSCAFG00845001868	6	4	7	2
ENSCAFG00845025839	0	0	0	1
ENSCAFG00845001865	0	0	0	0
ENSCAFG00845025832	0	0	0	0
ENSCAFG00845001866	0	0	0	0
ENSCAFG00845025833	1494	1506	1383	1400
ENSCAFG00845001863	0	0	0	0
ENSCAFG00845025834	0	4	0	2
ENSCAFG00845001864	62	51	55	68
ENSCAFG00845025835	0	0	0	0
ENSCAFG00845001861	3	5	2	2
ENSCAFG00845001862	10	10	6	6
ENSCAFG00845025830	0	0	0	0
ENSCAFG00845001860	6	3	4	1
ENSCAFG00845025831	148	154	140	186
ENSCAFG00845013858	0	0	0	0
ENSCAFG00845013857	0	0	0	0
ENSCAFG00845013859	0	0	1	0
ENSCAFG00845013854	0	0	0	0
ENSCAFG00845013853	80	81	58	88
ENSCAFG00845013856	2080	2098	2146	2345
ENSCAFG00845013855	0	3	0	0
ENSCAFG00845013850	0	0	2	4
ENSCAFG00845013852	101	74	123	116
ENSCAFG00845013851	2	5	5	3
ENSCAFG00845025829	5	2	0	3
ENSCAFG00845025825	487	457	635	561

ENSCAFG00845025826	0	0	0	0
ENSCAFG00845001878	375	429	453	513
ENSCAFG00845025827	0	0	0	0
ENSCAFG00845001879	16	13	11	8
ENSCAFG00845025828	30	29	31	31
ENSCAFG00845001876	293	299	246	224
ENSCAFG00845025821	185	136	227	220
ENSCAFG00845001877	4	3	0	0
ENSCAFG00845025822	626	652	668	657
ENSCAFG00845001874	249	245	279	255
ENSCAFG00845025823	0	0	0	0
ENSCAFG00845001875	0	1	2	0
ENSCAFG00845025824	152	131	139	142
ENSCAFG00845001872	137	148	177	201
ENSCAFG00845001873	1	4	1	2
ENSCAFG00845001870	2	3	1	3
ENSCAFG00845001871	920	788	896	864
ENSCAFG00845025820	0	0	1	0
ENSCAFG00845013847	1	2	0	1
ENSCAFG00845013846	1	4	1	0
ENSCAFG00845013849	0	0	0	0
ENSCAFG00845013848	1	5	2	1
ENSCAFG00845013843	3	11	18	20
ENSCAFG00845013842	1	4	4	1
ENSCAFG00845013845	3	0	0	1
ENSCAFG00845013844	1	1	1	4
ENSCAFG00845013841	52	42	63	65
ENSCAFG00845013840	3210	3199	3208	3319
ENSCAFG00845023299	0	0	0	0
ENSCAFG00845023298	0	5	2	0
ENSCAFG00845023293	443	396	572	598
ENSCAFG00845023292	4	6	12	6
ENSCAFG00845023291	0	0	0	0
ENSCAFG00845023290	506	419	425	437
ENSCAFG00845023297	1621	1548	1490	1552
ENSCAFG00845023296	2684	2693	2517	2686
ENSCAFG00845023295	1	0	0	0
ENSCAFG00845023294	4	18	13	24
ENSCAFG00845023289	0	0	1	0
ENSCAFG00845023288	17	24	13	20
ENSCAFG00845023287	191	188	209	214

ENSCAFG00845023282	167	195	164	138
ENSCAFG00845023281	0	0	0	0
ENSCAFG00845023280	2	4	2	6
ENSCAFG00845023286	0	2	0	0
ENSCAFG00845023285	0	0	0	0
ENSCAFG00845023284	2237	2251	2338	2417
ENSCAFG00845023283	1249	1252	1128	1195
ENSCAFG00845011293	0	0	0	0
ENSCAFG00845011294	2723	2811	2541	2569
ENSCAFG00845011295	1045	1033	938	982
ENSCAFG00845011296	298	275	300	266
ENSCAFG00845011290	0	0	0	0
ENSCAFG00845011291	0	0	0	0
ENSCAFG00845011292	0	0	0	0
ENSCAFG00845023279	0	0	0	0
ENSCAFG00845023278	2	0	1	0
ENSCAFG00845023277	0	0	0	0
ENSCAFG00845023276	0	0	0	0
ENSCAFG00845023271	864	816	1224	1306
ENSCAFG00845023270	2387	2310	1844	1801
ENSCAFG00845023275	589	559	652	626
ENSCAFG00845023274	309	285	284	294
ENSCAFG00845023273	17	18	18	43
ENSCAFG00845023272	0	0	0	0
ENSCAFG00845011297	555	546	503	559
ENSCAFG00845011298	0	0	0	0
ENSCAFG00845011299	30	34	21	23
ENSCAFG00845011282	1200	1149	1275	1351
ENSCAFG00845011283	2627	2596	3047	2958
ENSCAFG00845011284	0	0	0	0
ENSCAFG00845011285	0	0	0	0
ENSCAFG00845011280	0	0	0	0
ENSCAFG00845011281	0	0	0	0
ENSCAFG00845023268	896	864	863	759
ENSCAFG00845023267	5	16	13	5
ENSCAFG00845023266	0	0	0	0
ENSCAFG00845023265	1658	1719	1701	1671
ENSCAFG00845023269	7	2	4	7
ENSCAFG00845023260	7	4	7	12
ENSCAFG00845023264	216	184	204	204
ENSCAFG00845023263	0	0	0	0

ENSCAFG00845023262	1097	1115	1110	1142
ENSCAFG00845011286	195	224	214	209
ENSCAFG00845011287	5	11	24	19
ENSCAFG00845011288	570	527	634	595
ENSCAFG00845011289	19	22	22	26
ENSCAFG00845011271	0	0	0	0
ENSCAFG00845011272	0	0	0	0
ENSCAFG00845011273	0	0	0	0
ENSCAFG00845011274	17	28	25	20
ENSCAFG00845011270	136	151	145	141
ENSCAFG00845023257	0	0	0	0
ENSCAFG00845023256	1409	1229	1241	1382
ENSCAFG00845023255	0	0	0	0
ENSCAFG00845023254	0	0	0	0
ENSCAFG00845023259	1463	1442	1521	1546
ENSCAFG00845023258	605	595	659	620
ENSCAFG00845023253	471	457	502	531
ENSCAFG00845023252	264	270	232	219
ENSCAFG00845023251	0	0	0	0
ENSCAFG00845023250	194	204	192	209
ENSCAFG00845011279	3	0	5	0
ENSCAFG00845011275	0	0	0	0
ENSCAFG00845011276	0	2	1	2
ENSCAFG00845011277	3148	2959	2891	2997
ENSCAFG00845011278	104	69	90	80
ENSCAFG00845011260	1311	1311	1279	1286
ENSCAFG00845011261	375	342	384	333
ENSCAFG00845011262	357	309	282	295
ENSCAFG00845011263	0	0	0	0
ENSCAFG00845023245	0	0	0	0
ENSCAFG00845023244	1	0	0	0
ENSCAFG00845023243	2	8	3	5
ENSCAFG00845023249	125	133	121	120
ENSCAFG00845023248	2422	2384	2458	2501
ENSCAFG00845023247	0	0	0	0
ENSCAFG00845023242	140	122	130	119
ENSCAFG00845023241	618	608	609	608
ENSCAFG00845023240	504	488	505	488
ENSCAFG00845011268	0	0	0	0
ENSCAFG00845011269	408	370	267	285
ENSCAFG00845011264	0	1	1	3

ENSCAFG00845011265	1590	1478	904	985
ENSCAFG00845011266	340	234	343	323
ENSCAFG00845011267	179	192	251	227
ENSCAFG00845011419	5	7	5	8
ENSCAFG00845011415	0	2	1	0
ENSCAFG00845011416	0	0	0	0
ENSCAFG00845011417	7	5	2	4
ENSCAFG00845011418	0	0	0	0
ENSCAFG00845011411	1848	1764	2045	1971
ENSCAFG00845011412	493	491	489	466
ENSCAFG00845011413	0	0	0	0
ENSCAFG00845011414	208	232	224	229
ENSCAFG00845011410	244	251	234	209
ENSCAFG00845011408	41	41	64	38
ENSCAFG00845011409	3	0	4	4
ENSCAFG00845011404	8	8	8	6
ENSCAFG00845011405	208	223	160	154
ENSCAFG00845011406	9	12	8	18
ENSCAFG00845011407	0	0	0	0
ENSCAFG00845011400	49	68	86	74
ENSCAFG00845011401	29	15	17	15
ENSCAFG00845011402	0	0	0	0
ENSCAFG00845011403	0	0	0	0
ENSCAFG00845011370	79	79	56	79
ENSCAFG00845011371	190	158	137	148
ENSCAFG00845011372	0	0	0	0
ENSCAFG00845011373	1658	1558	1468	1539
ENSCAFG00845023356	863	818	789	823
ENSCAFG00845023355	0	2	3	0
ENSCAFG00845023354	5	4	3	1
ENSCAFG00845023353	970	940	952	1062
ENSCAFG00845023359	0	0	0	3
ENSCAFG00845023358	0	0	0	0
ENSCAFG00845023357	361	365	367	320
ENSCAFG00845023352	0	0	0	3
ENSCAFG00845023351	0	2	0	0
ENSCAFG00845023350	468	428	434	407
ENSCAFG00845011378	2542	2495	2527	2461
ENSCAFG00845011379	0	0	0	0
ENSCAFG00845011374	0	0	0	0
ENSCAFG00845011375	73	71	20	15

ENSCAFG00845011376	1	0	0	0
ENSCAFG00845011377	22	24	34	31
ENSCAFG00845011360	0	0	0	0
ENSCAFG00845011361	1	0	0	0
ENSCAFG00845011362	57	38	72	55
ENSCAFG00845023344	0	0	1	0
ENSCAFG00845023343	0	0	0	0
ENSCAFG00845023342	0	0	0	0
ENSCAFG00845023349	1	1	0	0
ENSCAFG00845023348	912	985	953	922
ENSCAFG00845023347	1670	1576	1444	1561
ENSCAFG00845023346	0	0	0	0
ENSCAFG00845023341	1018	1000	880	877
ENSCAFG00845023340	17	15	16	15
ENSCAFG00845011367	17	11	24	12
ENSCAFG00845011368	2060	2011	1970	1901
ENSCAFG00845011369	0	0	0	0
ENSCAFG00845011363	2276	2110	1913	2057
ENSCAFG00845011364	33	26	32	20
ENSCAFG00845011365	21906	21492	20271	19754
ENSCAFG00845011366	0	2	0	0
ENSCAFG00845011350	372	318	329	346
ENSCAFG00845011351	40	52	43	25
ENSCAFG00845023339	8	1	3	2
ENSCAFG00845023334	0	0	0	0
ENSCAFG00845025997	588	586	532	668
ENSCAFG00845023333	1790	1638	1676	1728
ENSCAFG00845025998	0	0	0	0
ENSCAFG00845023332	9	8	7	1
ENSCAFG00845025999	0	0	0	0
ENSCAFG00845023331	0	3	4	0
ENSCAFG00845023338	3	0	0	0
ENSCAFG00845025993	518	433	502	484
ENSCAFG00845023337	54	81	75	87
ENSCAFG00845025994	0	0	0	0
ENSCAFG00845023336	2	1	0	2
ENSCAFG00845025995	425	419	528	491
ENSCAFG00845023335	0	0	0	0
ENSCAFG00845025996	0	0	1	1
ENSCAFG00845025990	232	201	206	214
ENSCAFG00845025991	0	0	3	0

ENSCAFG00845025992	457	442	517	516
ENSCAFG00845023330	5	1	1	3
ENSCAFG00845011356	0	0	0	0
ENSCAFG00845011357	20	10	12	28
ENSCAFG00845011358	0	0	0	0
ENSCAFG00845011359	428	398	359	391
ENSCAFG00845011352	1	2	1	0
ENSCAFG00845011353	0	0	0	0
ENSCAFG00845011354	942	823	785	780
ENSCAFG00845011355	749	671	708	761
ENSCAFG00845023329	724	717	646	612
ENSCAFG00845011340	0	0	0	0
ENSCAFG00845023328	43	28	37	25
ENSCAFG00845023323	375	333	356	339
ENSCAFG00845025986	0	0	0	0
ENSCAFG00845023322	0	0	0	0
ENSCAFG00845025987	0	0	0	0
ENSCAFG00845025988	338	347	347	309
ENSCAFG00845023320	3	3	2	5
ENSCAFG00845025989	0	0	0	0
ENSCAFG00845023327	4	3	1	0
ENSCAFG00845025982	609	567	536	465
ENSCAFG00845023326	232	193	236	208
ENSCAFG00845025983	704	665	431	475
ENSCAFG00845023325	2	1	0	0
ENSCAFG00845025984	0	0	0	0
ENSCAFG00845023324	0	0	0	0
ENSCAFG00845025985	79	87	73	85
ENSCAFG00845025980	6075	5995	5616	5599
ENSCAFG00845011349	61	77	31	58
ENSCAFG00845011345	0	0	0	0
ENSCAFG00845011346	0	0	0	0
ENSCAFG00845011347	132	125	147	149
ENSCAFG00845011348	1512	1466	1537	1599
ENSCAFG00845011341	1209	1102	980	1096
ENSCAFG00845011342	408	383	459	432
ENSCAFG00845011343	0	0	0	1
ENSCAFG00845011344	33	36	43	31
ENSCAFG00845023319	360	349	311	286
ENSCAFG00845013991	47	53	45	54
ENSCAFG00845023318	0	0	0	0

ENSCAFG00845013990	0	0	0	0
ENSCAFG00845023317	0	0	0	1
ENSCAFG00845025979	353	291	325	252
ENSCAFG00845023312	2	2	0	1
ENSCAFG00845025975	0	0	0	0
ENSCAFG00845023311	0	3	0	2
ENSCAFG00845025976	163	143	140	134
ENSCAFG00845023310	21	21	22	23
ENSCAFG00845025977	1489	1533	1586	1588
ENSCAFG00845025978	0	1	0	0
ENSCAFG00845023316	0	0	0	0
ENSCAFG00845025971	0	0	0	0
ENSCAFG00845023315	703	662	586	593
ENSCAFG00845025972	3	1	10	6
ENSCAFG00845023314	1	4	2	1
ENSCAFG00845025973	0	0	0	0
ENSCAFG00845023313	57	64	68	59
ENSCAFG00845025974	1016	964	890	922
ENSCAFG00845025970	0	0	0	0
ENSCAFG00845011338	0	0	0	0
ENSCAFG00845011339	28	37	24	33
ENSCAFG00845011334	35	33	28	24
ENSCAFG00845013997	477	480	454	453
ENSCAFG00845011335	1814	1779	1569	1605
ENSCAFG00845013996	0	0	0	0
ENSCAFG00845011336	0	0	0	0
ENSCAFG00845013999	0	0	0	0
ENSCAFG00845011337	1309	1241	1279	1319
ENSCAFG00845013998	370	337	362	373
ENSCAFG00845011330	2754	2547	2860	2811
ENSCAFG00845013993	0	0	0	0
ENSCAFG00845011331	0	0	0	0
ENSCAFG00845013992	564	495	562	551
ENSCAFG00845011332	15	12	4	4
ENSCAFG00845013995	242	234	251	243
ENSCAFG00845011333	0	0	0	0
ENSCAFG00845013994	0	0	0	0
ENSCAFG00845023309	0	0	0	0
ENSCAFG00845013980	8	10	5	11
ENSCAFG00845023307	3557	3489	3524	3590
ENSCAFG00845023306	3650	3451	3333	3359

ENSCAFG00845025968	0	0	0	0
ENSCAFG00845025969	1	0	0	0
ENSCAFG00845023301	927	917	857	901
ENSCAFG00845025964	74	68	59	106
ENSCAFG00845023300	4	0	2	4
ENSCAFG00845025965	0	0	0	0
ENSCAFG00845025966	0	0	0	0
ENSCAFG00845025967	15	17	24	34
ENSCAFG00845023305	855	856	837	840
ENSCAFG00845025960	1830	1688	2200	2224
ENSCAFG00845023303	0	0	0	0
ENSCAFG00845025962	724	665	771	611
ENSCAFG00845023302	0	0	0	0
ENSCAFG00845025963	24	17	25	15
ENSCAFG00845011327	0	0	1	2
ENSCAFG00845011328	1384	1322	1326	1318
ENSCAFG00845013989	0	0	0	0
ENSCAFG00845011329	0	0	0	0
ENSCAFG00845011323	33	29	18	18
ENSCAFG00845013986	0	0	0	1
ENSCAFG00845011324	9	13	4	4
ENSCAFG00845013985	373	304	408	346
ENSCAFG00845011325	35	46	50	52
ENSCAFG00845013988	0	0	2	3
ENSCAFG00845011326	467	448	610	591
ENSCAFG00845013987	864	744	847	869
ENSCAFG00845013982	0	0	0	0
ENSCAFG00845011320	0	0	0	0
ENSCAFG00845013981	5	2	5	6
ENSCAFG00845011321	24	27	33	31
ENSCAFG00845013984	0	0	1	1
ENSCAFG00845011322	228	230	242	253
ENSCAFG00845013983	0	0	0	0
ENSCAFG00845025957	826	860	792	964
ENSCAFG00845025958	0	0	0	0
ENSCAFG00845001988	0	0	0	0
ENSCAFG00845025959	9888	9582	9799	10022
ENSCAFG00845001989	0	0	0	0
ENSCAFG00845001986	5	8	2	3
ENSCAFG00845025953	1269	1228	1096	1126
ENSCAFG00845001987	0	0	0	0

ENSCAFG00845025954	6	8	5	2
ENSCAFG00845001984	194	180	196	211
ENSCAFG00845025955	0	0	0	0
ENSCAFG00845001985	3040	2824	3110	3006
ENSCAFG00845025956	3169	3168	3555	3539
ENSCAFG00845001982	0	0	0	0
ENSCAFG00845001983	0	2	0	1
ENSCAFG00845025950	33	41	74	43
ENSCAFG00845001980	0	0	0	0
ENSCAFG00845025951	734	753	724	786
ENSCAFG00845001981	164	146	141	112
ENSCAFG00845025952	7163	7343	6855	6880
ENSCAFG00845011316	260	268	235	249
ENSCAFG00845013979	2	1	0	1
ENSCAFG00845011317	686	668	646	767
ENSCAFG00845013978	1	0	2	0
ENSCAFG00845011318	884	775	817	848
ENSCAFG00845011319	1258	1183	1227	1251
ENSCAFG00845011312	0	0	1	2
ENSCAFG00845013975	32	30	26	37
ENSCAFG00845011313	5	7	7	2
ENSCAFG00845013974	437	490	461	487
ENSCAFG00845011314	2	1	0	0
ENSCAFG00845013977	1	1	0	0
ENSCAFG00845011315	0	0	0	0
ENSCAFG00845013976	2	4	2	9
ENSCAFG00845013971	8	7	2	11
ENSCAFG00845013970	96	97	134	149
ENSCAFG00845011310	0	0	0	0
ENSCAFG00845013973	0	0	0	0
ENSCAFG00845011311	0	0	0	0
ENSCAFG00845013972	34	41	33	32
ENSCAFG00845025946	4878	4614	4505	4549
ENSCAFG00845025947	4	3	4	0
ENSCAFG00845001999	2	3	0	2
ENSCAFG00845025948	0	0	0	0
ENSCAFG00845025949	0	0	0	0
ENSCAFG00845001997	0	0	0	0
ENSCAFG00845025942	0	0	0	0
ENSCAFG00845001998	1	5	8	9
ENSCAFG00845025943	3	9	1	2

ENSCAFG00845001995	0	0	0	0
ENSCAFG00845025944	7	1	5	5
ENSCAFG00845001996	0	0	0	0
ENSCAFG00845025945	433	332	396	432
ENSCAFG00845001993	0	0	0	0
ENSCAFG00845001994	44	49	100	73
ENSCAFG00845001991	0	0	0	0
ENSCAFG00845025940	0	0	0	0
ENSCAFG00845001992	508	480	441	430
ENSCAFG00845025941	1658	1604	1459	1519
ENSCAFG00845011309	0	0	0	0
ENSCAFG00845001990	32	44	57	55
ENSCAFG00845011305	5	8	8	8
ENSCAFG00845013968	581	526	541	533
ENSCAFG00845011306	0	0	0	0
ENSCAFG00845013967	0	0	0	0
ENSCAFG00845011307	11	12	10	12
ENSCAFG00845011308	0	0	0	0
ENSCAFG00845013969	1272	1186	1048	1105
ENSCAFG00845011301	0	0	0	0
ENSCAFG00845013964	1	0	1	0
ENSCAFG00845011302	0	2	6	1
ENSCAFG00845013963	492	503	500	494
ENSCAFG00845011303	47	49	38	31
ENSCAFG00845013966	0	4	0	2
ENSCAFG00845011304	1117	1193	1156	1188
ENSCAFG00845013965	235	192	196	213
ENSCAFG00845013960	794	690	771	788
ENSCAFG00845013962	370	398	328	315
ENSCAFG00845011300	951	900	742	803
ENSCAFG00845013961	1	2	2	0
ENSCAFG00845023399	0	0	0	0
ENSCAFG00845023398	0	0	0	0
ENSCAFG00845023397	0	0	0	4
ENSCAFG00845023392	2	1	1	0
ENSCAFG00845023391	4	6	4	2
ENSCAFG00845023390	2668	2702	2824	2859
ENSCAFG00845023396	793	758	740	779
ENSCAFG00845023395	6132	5800	5620	5630
ENSCAFG00845023394	307	276	284	268
ENSCAFG00845023393	16923	16669	16477	17016

ENSCAFG00845023389	0	0	0	0
ENSCAFG00845023388	1697	1698	1689	1749
ENSCAFG00845023387	736	731	664	677
ENSCAFG00845023386	2	1	3	0
ENSCAFG00845023381	2	0	11	6
ENSCAFG00845023380	0	0	0	0
ENSCAFG00845023385	0	1	0	0
ENSCAFG00845023384	0	0	0	0
ENSCAFG00845023383	1390	1421	1339	1413
ENSCAFG00845023382	1454	1462	1547	1512
ENSCAFG00845011392	3007	2855	2848	2733
ENSCAFG00845011393	226	230	266	249
ENSCAFG00845011394	0	0	0	0
ENSCAFG00845011395	213	200	208	191
ENSCAFG00845011390	0	0	0	0
ENSCAFG00845011391	997	946	991	1085
ENSCAFG00845023378	1269	1219	1187	1117
ENSCAFG00845023377	335	316	202	230
ENSCAFG00845023376	4	4	0	0
ENSCAFG00845023375	0	0	0	0
ENSCAFG00845023379	55	44	54	53
ENSCAFG00845023370	0	3	0	1
ENSCAFG00845023374	23	26	18	15
ENSCAFG00845023373	4	4	3	3
ENSCAFG00845023372	186	179	160	144
ENSCAFG00845023371	767	786	807	821
ENSCAFG00845011396	501	471	447	464
ENSCAFG00845011397	200	173	173	181
ENSCAFG00845011398	2014	2050	1934	2074
ENSCAFG00845011399	0	0	0	0
ENSCAFG00845011381	0	0	2	0
ENSCAFG00845011382	305	297	313	273
ENSCAFG00845011383	437	463	462	455
ENSCAFG00845011384	171	171	166	168
ENSCAFG00845011380	541	529	479	510
ENSCAFG00845023366	0	0	0	0
ENSCAFG00845023365	120	101	119	120
ENSCAFG00845023364	0	0	0	0
ENSCAFG00845023369	554	568	549	544
ENSCAFG00845023368	45	33	29	38
ENSCAFG00845023363	19	22	21	25

ENSCAFG00845023362	0	0	2	3
ENSCAFG00845023361	5	0	0	0
ENSCAFG00845023360	0	0	0	0
ENSCAFG00845011389	20	12	5	8
ENSCAFG00845011385	243	199	194	156
ENSCAFG00845011386	0	0	0	0
ENSCAFG00845011387	89	103	112	128
ENSCAFG00845011388	0	0	0	0
ENSCAFG00845023518	15	16	24	16
ENSCAFG00845023517	1232	1166	1326	1221
ENSCAFG00845023516	656	623	521	439
ENSCAFG00845023515	0	0	0	0
ENSCAFG00845023519	7	3	5	1
ENSCAFG00845023510	5	2	0	1
ENSCAFG00845023514	481	431	468	449
ENSCAFG00845023513	2	2	0	2
ENSCAFG00845023512	2302	2332	2063	2081
ENSCAFG00845023511	0	0	0	0
ENSCAFG00845011536	0	0	0	0
ENSCAFG00845011537	5	6	9	1
ENSCAFG00845011538	5012	5039	4799	4993
ENSCAFG00845011539	1045	926	931	1021
ENSCAFG00845011532	0	0	0	0
ENSCAFG00845011533	10	5	4	2
ENSCAFG00845011534	499	505	456	484
ENSCAFG00845011535	316	337	339	381
ENSCAFG00845011530	261	255	311	303
ENSCAFG00845011531	9	1	5	0
ENSCAFG00845023507	0	0	0	0
ENSCAFG00845023506	0	0	0	0
ENSCAFG00845023505	0	0	0	0
ENSCAFG00845023504	1510	1460	1423	1436
ENSCAFG00845023509	11990	11767	11934	11863
ENSCAFG00845023508	0	0	0	0
ENSCAFG00845023503	2	4	5	2
ENSCAFG00845023502	311	281	339	343
ENSCAFG00845023501	1	0	2	5
ENSCAFG00845023500	0	0	0	0
ENSCAFG00845011529	104	108	138	106
ENSCAFG00845011525	0	0	0	0
ENSCAFG00845011526	401	424	444	387

ENSCAFG00845011527	4973	4869	4345	4419
ENSCAFG00845011528	1354	1284	1204	1233
ENSCAFG00845011521	274	240	263	281
ENSCAFG00845011522	216	272	297	313
ENSCAFG00845011523	1	0	7	1
ENSCAFG00845011524	0	4	2	1
ENSCAFG00845011520	0	0	0	0
ENSCAFG00845011518	16	11	9	10
ENSCAFG00845011519	11	12	14	8
ENSCAFG00845011514	0	0	0	0
ENSCAFG00845011515	451	399	435	421
ENSCAFG00845011516	18	25	21	27
ENSCAFG00845011517	0	0	0	0
ENSCAFG00845011510	0	1	0	0
ENSCAFG00845011511	0	0	0	0
ENSCAFG00845011512	0	0	0	0
ENSCAFG00845011513	578	494	504	554
ENSCAFG00845011507	1522	1488	1511	1560
ENSCAFG00845011508	0	0	0	0
ENSCAFG00845011509	504	517	464	472
ENSCAFG00845011503	0	0	0	0
ENSCAFG00845011504	0	0	0	0
ENSCAFG00845011505	509	451	534	494
ENSCAFG00845011506	11	2	0	1
ENSCAFG00845011500	2	0	6	0
ENSCAFG00845011501	0	0	0	0
ENSCAFG00845011502	6	5	12	7
ENSCAFG00845011491	0	0	0	0
ENSCAFG00845011492	2	6	11	9
ENSCAFG00845011493	74	58	70	62
ENSCAFG00845011494	1259	1123	1463	1555
ENSCAFG00845011490	539	595	497	554
ENSCAFG00845023476	0	0	0	0
ENSCAFG00845023475	3604	3405	3896	3996
ENSCAFG00845023474	0	0	0	0
ENSCAFG00845023479	3	7	2	4
ENSCAFG00845023478	1076	1022	610	672
ENSCAFG00845023473	95	65	89	110
ENSCAFG00845023472	9161	8784	8992	8988
ENSCAFG00845023471	1322	1259	1173	1229
ENSCAFG00845023470	9	9	9	12

ENSCAFG00845011499	0	0	0	0
ENSCAFG00845011495	0	0	0	0
ENSCAFG00845011496	1725	1595	1475	1552
ENSCAFG00845011497	0	0	0	0
ENSCAFG00845011498	1	0	0	1
ENSCAFG00845011480	2	9	13	3
ENSCAFG00845011481	2	0	0	1
ENSCAFG00845011482	2654	2605	2561	2701
ENSCAFG00845011483	51	54	57	59
ENSCAFG00845023466	0	0	0	0
ENSCAFG00845023465	225	237	262	230
ENSCAFG00845023464	0	0	0	0
ENSCAFG00845023463	1191	1149	1174	1181
ENSCAFG00845023469	2069	1982	2115	2139
ENSCAFG00845023468	111	130	108	106
ENSCAFG00845023467	80	80	61	81
ENSCAFG00845023462	1	1	4	0
ENSCAFG00845023461	0	0	0	0
ENSCAFG00845023460	119	124	176	148
ENSCAFG00845011488	0	0	0	0
ENSCAFG00845011489	0	2	0	1
ENSCAFG00845011484	1331	1425	1386	1368
ENSCAFG00845011485	0	0	0	0
ENSCAFG00845011486	300	273	298	298
ENSCAFG00845011487	1	2	0	0
ENSCAFG00845011470	6604	6340	6086	6321
ENSCAFG00845011471	0	0	0	0
ENSCAFG00845011472	0	0	0	0
ENSCAFG00845023455	2	2	5	4
ENSCAFG00845023454	3	7	2	8
ENSCAFG00845023453	0	0	0	0
ENSCAFG00845023452	0	0	0	0
ENSCAFG00845023459	1206	1209	1110	1112
ENSCAFG00845023457	0	0	0	0
ENSCAFG00845023456	0	0	0	0
ENSCAFG00845023451	317	328	372	365
ENSCAFG00845023450	0	0	0	0
ENSCAFG00845011477	1262	1268	1156	1103
ENSCAFG00845011478	0	0	0	0
ENSCAFG00845011479	484	435	404	415
ENSCAFG00845011473	0	0	0	0

ENSCAFG00845011474	1065	1042	996	1032
ENSCAFG00845011475	4	12	9	9
ENSCAFG00845011476	1	0	1	0
ENSCAFG00845011460	0	0	0	0
ENSCAFG00845011461	2616	2639	2508	2620
ENSCAFG00845023449	6	3	7	7
ENSCAFG00845023444	1348	1357	1229	1313
ENSCAFG00845023443	13	12	9	8
ENSCAFG00845023442	870	818	934	1015
ENSCAFG00845023441	2	2	0	0
ENSCAFG00845023448	137	120	120	119
ENSCAFG00845023447	6	8	20	14
ENSCAFG00845023446	0	0	0	0
ENSCAFG00845023445	8	8	7	2
ENSCAFG00845023440	76	86	83	64
ENSCAFG00845011466	0	0	0	0
ENSCAFG00845011467	1060	934	976	1054
ENSCAFG00845011468	0	0	0	0
ENSCAFG00845011469	20	27	16	13
ENSCAFG00845011462	15	16	10	21
ENSCAFG00845011463	82	105	78	102
ENSCAFG00845011464	134	134	142	131
ENSCAFG00845011465	954	928	986	945
ENSCAFG00845023439	1	0	0	0
ENSCAFG00845011450	1047	1116	935	975
ENSCAFG00845023438	2	4	2	5
ENSCAFG00845023433	0	0	0	0
ENSCAFG00845023432	208	217	117	124
ENSCAFG00845023431	0	0	2	1
ENSCAFG00845023430	2447	2207	2299	2200
ENSCAFG00845023437	455	430	463	388
ENSCAFG00845023436	0	0	0	0
ENSCAFG00845023435	17	6	13	6
ENSCAFG00845011459	91	65	116	83
ENSCAFG00845011455	3	0	0	0
ENSCAFG00845011456	4	6	4	9
ENSCAFG00845011457	0	0	0	0
ENSCAFG00845011458	0	0	0	2
ENSCAFG00845011451	0	0	0	2
ENSCAFG00845011452	4	6	6	10
ENSCAFG00845011453	0	0	0	0

ENSCAFG00845011454	61	66	61	67
ENSCAFG00845023429	1	4	1	4
ENSCAFG00845023428	394	371	403	401
ENSCAFG00845023427	0	0	0	0
ENSCAFG00845023422	240	247	239	264
ENSCAFG00845023421	47	46	37	38
ENSCAFG00845023420	294	279	288	302
ENSCAFG00845023426	0	2	7	3
ENSCAFG00845023425	0	0	0	1
ENSCAFG00845023424	0	0	0	0
ENSCAFG00845023423	895	862	811	752
ENSCAFG00845011448	11	8	13	10
ENSCAFG00845011449	0	0	0	0
ENSCAFG00845011444	324	351	330	335
ENSCAFG00845011445	671	752	684	673
ENSCAFG00845011446	0	1	4	4
ENSCAFG00845011447	153	133	139	148
ENSCAFG00845011440	0	0	0	0
ENSCAFG00845011441	355	408	320	378
ENSCAFG00845011442	0	5	0	1
ENSCAFG00845011443	1643	1604	1680	1565
ENSCAFG00845023419	0	0	0	0
ENSCAFG00845023418	263	222	263	249
ENSCAFG00845023416	2160	2329	2110	2128
ENSCAFG00845023411	309	267	436	461
ENSCAFG00845023410	0	0	0	0
ENSCAFG00845023415	0	0	0	0
ENSCAFG00845023414	2699	2570	2842	2993
ENSCAFG00845023413	942	934	859	822
ENSCAFG00845023412	4	3	7	6
ENSCAFG00845011437	0	0	0	0
ENSCAFG00845011438	393	368	453	413
ENSCAFG00845011439	0	0	0	0
ENSCAFG00845011433	5	5	1	4
ENSCAFG00845011434	18	4	20	11
ENSCAFG00845011435	396	375	476	484
ENSCAFG00845011436	0	0	0	0
ENSCAFG00845011430	2028	2036	1784	1724
ENSCAFG00845011431	2	0	0	2
ENSCAFG00845011432	0	0	0	0
ENSCAFG00845023408	536	476	405	453

ENSCAFG00845023407	0	0	0	0
ENSCAFG00845023406	5	3	0	4
ENSCAFG00845023405	182	169	176	166
ENSCAFG00845023409	0	0	0	0
ENSCAFG00845023404	4	0	2	2
ENSCAFG00845023403	0	0	0	0
ENSCAFG00845023402	881	862	829	944
ENSCAFG00845011426	0	0	0	1
ENSCAFG00845011427	0	0	0	0
ENSCAFG00845011428	0	0	0	0
ENSCAFG00845011429	0	0	0	0
ENSCAFG00845011422	297	285	319	324
ENSCAFG00845011423	3	1	1	7
ENSCAFG00845011424	0	0	0	0
ENSCAFG00845011425	0	0	0	0
ENSCAFG00845011420	738	745	531	620
ENSCAFG00845011421	0	0	0	0
ENSCAFG00845023499	526	486	585	505
ENSCAFG00845023498	43	59	54	54
ENSCAFG00845023497	828	858	692	811
ENSCAFG00845023496	0	0	0	0
ENSCAFG00845023491	0	0	0	0
ENSCAFG00845023490	1	8	5	4
ENSCAFG00845023495	196	212	227	246
ENSCAFG00845023493	680	647	537	581
ENSCAFG00845023492	981	960	847	828
ENSCAFG00845023488	0	0	0	0
ENSCAFG00845023487	2	2	3	0
ENSCAFG00845023486	2637	2581	2882	2985
ENSCAFG00845023485	3	3	1	3
ENSCAFG00845023489	947	884	984	1052
ENSCAFG00845023480	84	63	86	82
ENSCAFG00845023484	73	60	82	95
ENSCAFG00845023483	15	24	16	12
ENSCAFG00845023482	816	736	730	726
ENSCAFG00845023481	0	1	0	0
ENSCAFG00845025399	0	0	0	0
ENSCAFG00845025395	1554	1435	1402	1311
ENSCAFG00845025396	1	2	9	7
ENSCAFG00845025397	423	364	313	268
ENSCAFG00845025398	0	0	0	0

ENSCAFG00845025391	1119	1038	975	1037
ENSCAFG00845025392	959	895	910	868
ENSCAFG00845025393	0	0	0	0
ENSCAFG00845025394	0	1	0	0
ENSCAFG00845025390	1	1	0	0
ENSCAFG00845025388	1599	1501	1603	1558
ENSCAFG00845025389	0	0	0	0
ENSCAFG00845025384	0	0	7	1
ENSCAFG00845025386	822	807	761	915
ENSCAFG00845025387	0	0	0	0
ENSCAFG00845025380	0	0	0	0
ENSCAFG00845025381	0	0	0	0
ENSCAFG00845025382	0	0	0	0
ENSCAFG00845025383	17	10	9	12
ENSCAFG00845015970	0	0	0	0
ENSCAFG00845015971	0	0	0	0
ENSCAFG00845027959	887	858	968	934
ENSCAFG00845001328	8	6	9	9
ENSCAFG00845001329	2696	2579	2577	2577
ENSCAFG00845001326	0	0	0	0
ENSCAFG00845003989	0	0	0	0
ENSCAFG00845027956	0	0	0	0
ENSCAFG00845001327	1	5	3	4
ENSCAFG00845003988	0	0	0	0
ENSCAFG00845027955	0	0	0	0
ENSCAFG00845001324	0	0	0	1
ENSCAFG00845003987	0	0	0	0
ENSCAFG00845027958	1089	1025	960	968
ENSCAFG00845001325	85	87	82	75
ENSCAFG00845003986	3	0	0	1
ENSCAFG00845027957	0	0	0	0
ENSCAFG00845001322	696	611	494	539
ENSCAFG00845003985	0	0	0	0
ENSCAFG00845027952	668	594	597	671
ENSCAFG00845001323	0	0	0	0
ENSCAFG00845003984	4	1	2	2
ENSCAFG00845027951	0	0	0	0
ENSCAFG00845001320	165	158	212	187
ENSCAFG00845003983	0	0	0	0
ENSCAFG00845027954	8	10	4	10
ENSCAFG00845001321	28	40	36	38

ENSCAFG00845003982	0	0	0	0
ENSCAFG00845027953	523	552	474	529
ENSCAFG00845003981	0	0	0	0
ENSCAFG00845003980	0	0	0	0
ENSCAFG00845027950	0	0	0	0
ENSCAFG00845013319	32	36	34	40
ENSCAFG00845013318	0	0	0	0
ENSCAFG00845013315	0	0	0	0
ENSCAFG00845015978	0	0	0	0
ENSCAFG00845013314	2	2	2	2
ENSCAFG00845015979	774	761	604	690
ENSCAFG00845013317	312	346	363	427
ENSCAFG00845015976	0	0	0	0
ENSCAFG00845013316	0	0	0	0
ENSCAFG00845015977	1024	937	936	958
ENSCAFG00845013311	209	187	214	215
ENSCAFG00845015974	0	0	0	0
ENSCAFG00845013310	0	0	0	0
ENSCAFG00845015975	5	3	6	1
ENSCAFG00845013313	0	0	0	0
ENSCAFG00845015972	6	2	5	3
ENSCAFG00845013312	2	0	0	1
ENSCAFG00845015973	1651	1576	1786	1791
ENSCAFG00845015960	0	0	0	0
ENSCAFG00845027949	0	0	0	0
ENSCAFG00845027948	0	0	0	0
ENSCAFG00845001339	0	0	0	0
ENSCAFG00845001337	1240	1212	1214	1130
ENSCAFG00845027945	0	0	0	0
ENSCAFG00845001338	0	0	0	0
ENSCAFG00845003999	0	0	0	0
ENSCAFG00845027944	0	0	0	0
ENSCAFG00845001335	0	0	0	0
ENSCAFG00845003998	1183	1167	1130	1122
ENSCAFG00845027947	0	0	0	0
ENSCAFG00845001336	11	13	15	15
ENSCAFG00845003997	9	15	8	12
ENSCAFG00845027946	0	0	0	0
ENSCAFG00845001333	9	11	7	10
ENSCAFG00845003996	559	524	711	673
ENSCAFG00845027941	0	0	0	0

ENSCAFG00845001334	0	0	0	0
ENSCAFG00845003995	438	482	503	409
ENSCAFG00845027940	0	2	2	0
ENSCAFG00845001331	0	0	0	0
ENSCAFG00845003994	1099	1076	974	1096
ENSCAFG00845027943	0	0	0	0
ENSCAFG00845001332	100	89	117	145
ENSCAFG00845003993	34	27	30	25
ENSCAFG00845027942	102	107	78	46
ENSCAFG00845003992	59	43	33	51
ENSCAFG00845001330	51	58	52	64
ENSCAFG00845003991	3	12	6	9
ENSCAFG00845003990	1	1	1	3
ENSCAFG00845013308	0	0	0	0
ENSCAFG00845013307	1	3	2	2
ENSCAFG00845015969	151	196	221	214
ENSCAFG00845013309	0	0	0	0
ENSCAFG00845013304	8	13	9	16
ENSCAFG00845015967	0	0	0	0
ENSCAFG00845013303	0	0	0	0
ENSCAFG00845015968	0	0	0	0
ENSCAFG00845013306	0	0	0	0
ENSCAFG00845015965	69	48	63	77
ENSCAFG00845013305	0	0	0	1
ENSCAFG00845015966	443	473	416	409
ENSCAFG00845013300	15	14	9	14
ENSCAFG00845015963	1	0	5	3
ENSCAFG00845015964	0	0	0	0
ENSCAFG00845013302	0	0	0	0
ENSCAFG00845015961	0	0	0	0
ENSCAFG00845013301	2	0	3	0
ENSCAFG00845015962	5	0	2	0
ENSCAFG00845001308	2174	2227	2110	2176
ENSCAFG00845027938	218	241	212	201
ENSCAFG00845001309	48	31	46	33
ENSCAFG00845027937	0	0	0	0
ENSCAFG00845001306	14	19	22	34
ENSCAFG00845003969	0	1	3	2
ENSCAFG00845001307	0	0	0	0
ENSCAFG00845003968	0	0	0	0
ENSCAFG00845027939	84	51	77	96

ENSCAFG00845001304	743	729	788	868
ENSCAFG00845003967	1079	1045	1088	1114
ENSCAFG00845027934	0	0	0	0
ENSCAFG00845001305	4	1	3	6
ENSCAFG00845003966	4	1	4	0
ENSCAFG00845027933	0	0	0	0
ENSCAFG00845001302	0	4	7	2
ENSCAFG00845003965	0	2	2	1
ENSCAFG00845027936	279	290	340	340
ENSCAFG00845001303	32	33	34	33
ENSCAFG00845003964	21	15	18	19
ENSCAFG00845027935	0	0	0	0
ENSCAFG00845001300	1	0	0	2
ENSCAFG00845003963	2	0	4	5
ENSCAFG00845027930	19	14	20	19
ENSCAFG00845001301	0	1	0	3
ENSCAFG00845003962	779	712	763	810
ENSCAFG00845003961	4	4	8	2
ENSCAFG00845027932	41	52	38	40
ENSCAFG00845003960	0	0	0	0
ENSCAFG00845027931	1	0	0	0
ENSCAFG00845015958	0	0	0	3
ENSCAFG00845015959	1	0	0	0
ENSCAFG00845015956	0	0	0	0
ENSCAFG00845015957	0	0	0	0
ENSCAFG00845015954	46	62	60	45
ENSCAFG00845015955	602	693	608	735
ENSCAFG00845015952	0	0	0	0
ENSCAFG00845015953	0	0	0	0
ENSCAFG00845015950	4049	4048	3635	3883
ENSCAFG00845015951	161	143	129	129
ENSCAFG00845001319	0	0	0	0
ENSCAFG00845027927	556	533	585	592
ENSCAFG00845027926	523	540	553	507
ENSCAFG00845001317	701	691	659	606
ENSCAFG00845027929	406	436	419	508
ENSCAFG00845001318	195	183	217	173
ENSCAFG00845003979	0	0	0	0
ENSCAFG00845027928	2	0	0	0
ENSCAFG00845001315	0	0	0	0
ENSCAFG00845003978	0	0	0	0

ENSCAFG00845027923	712	725	634	601
ENSCAFG00845001316	0	0	0	0
ENSCAFG00845003977	6	4	3	2
ENSCAFG00845027922	25	35	27	31
ENSCAFG00845001313	0	0	0	0
ENSCAFG00845003976	2	2	2	2
ENSCAFG00845027925	5	9	7	5
ENSCAFG00845001314	253	199	202	183
ENSCAFG00845003975	3	3	3	3
ENSCAFG00845027924	3360	3157	3776	3614
ENSCAFG00845001311	242	270	259	279
ENSCAFG00845003974	131	125	145	153
ENSCAFG00845001312	1154	1048	1160	1226
ENSCAFG00845003973	4665	4464	4300	4139
ENSCAFG00845003972	0	2	0	2
ENSCAFG00845027921	0	0	0	0
ENSCAFG00845001310	121	110	162	142
ENSCAFG00845003971	376	330	285	334
ENSCAFG00845027920	0	2	0	0
ENSCAFG00845003970	0	0	0	0
ENSCAFG00845015949	545	520	560	618
ENSCAFG00845015947	2431	2344	2270	2309
ENSCAFG00845015948	0	0	0	0
ENSCAFG00845015945	830	824	847	877
ENSCAFG00845015946	172	158	127	159
ENSCAFG00845015943	0	0	0	0
ENSCAFG00845015944	0	0	0	0
ENSCAFG00845015941	0	0	0	0
ENSCAFG00845015942	24	22	24	8
ENSCAFG00845015940	0	0	0	0
ENSCAFG00845027919	23	33	36	29
ENSCAFG00845003949	0	0	0	0
ENSCAFG00845027916	443	421	382	455
ENSCAFG00845003948	0	0	0	0
ENSCAFG00845027915	542	527	606	613
ENSCAFG00845003947	162	171	176	164
ENSCAFG00845027918	0	0	0	0
ENSCAFG00845003946	3	8	0	10
ENSCAFG00845027917	1232	1226	1175	1180
ENSCAFG00845003945	0	0	0	0
ENSCAFG00845027912	0	0	0	0

ENSCAFG00845003944	3	2	3	0
ENSCAFG00845027911	769	716	713	667
ENSCAFG00845003943	19	22	9	7
ENSCAFG00845027914	0	0	0	0
ENSCAFG00845003942	0	0	0	0
ENSCAFG00845027913	47	54	41	43
ENSCAFG00845003941	1	0	3	0
ENSCAFG00845003940	289	264	294	310
ENSCAFG00845027910	1477	1410	1432	1271
ENSCAFG00845015938	0	0	0	0
ENSCAFG00845015939	2	1	1	3
ENSCAFG00845015936	0	2	0	5
ENSCAFG00845015937	4	10	15	22
ENSCAFG00845015934	437	463	392	307
ENSCAFG00845015935	0	0	0	0
ENSCAFG00845015932	0	2	1	5
ENSCAFG00845015933	688	646	853	865
ENSCAFG00845015930	359	317	286	310
ENSCAFG00845015931	0	0	0	0
ENSCAFG00845027909	4	3	3	0
ENSCAFG00845027908	0	0	0	0
ENSCAFG00845003959	60	74	141	150
ENSCAFG00845027904	412	365	391	388
ENSCAFG00845003958	0	0	0	0
ENSCAFG00845027907	1	0	0	0
ENSCAFG00845003957	49	57	84	97
ENSCAFG00845027906	46	65	66	55
ENSCAFG00845003956	0	0	0	0
ENSCAFG00845027901	57568	55810	55035	54716
ENSCAFG00845003955	5	9	1	5
ENSCAFG00845027900	0	0	0	0
ENSCAFG00845003954	35	27	25	37
ENSCAFG00845027903	0	0	0	0
ENSCAFG00845003953	44	42	35	39
ENSCAFG00845027902	0	0	0	0
ENSCAFG00845003952	0	0	0	0
ENSCAFG00845003951	0	0	0	0
ENSCAFG00845003950	397	332	698	704
ENSCAFG00845015929	2	0	1	3
ENSCAFG00845015927	0	0	0	0
ENSCAFG00845015928	0	0	0	0

ENSCAFG00845015925	0	0	0	0
ENSCAFG00845015926	0	1	0	0
ENSCAFG00845015923	101	112	87	84
ENSCAFG00845015924	0	0	0	0
ENSCAFG00845015921	0	0	0	0
ENSCAFG00845015922	111	98	122	114
ENSCAFG00845015920	0	0	0	0
ENSCAFG00845003929	0	0	0	0
ENSCAFG00845003928	154	180	186	168
ENSCAFG00845003927	0	0	0	0
ENSCAFG00845003926	1518	1326	1395	1471
ENSCAFG00845003925	11	15	22	19
ENSCAFG00845003924	0	0	0	0
ENSCAFG00845003923	495	491	503	492
ENSCAFG00845003922	34	17	17	43
ENSCAFG00845003921	3	3	5	3
ENSCAFG00845003920	493	440	487	418
ENSCAFG00845015918	0	0	0	0
ENSCAFG00845015919	0	0	0	0
ENSCAFG00845015916	1	0	0	0
ENSCAFG00845015917	0	0	0	0
ENSCAFG00845015914	2	1	1	0
ENSCAFG00845015915	12	7	19	24
ENSCAFG00845015912	0	0	0	0
ENSCAFG00845015913	1939	1872	2007	1885
ENSCAFG00845015910	0	0	0	0
ENSCAFG00845015911	6	3	3	8
ENSCAFG00845003939	0	0	0	0
ENSCAFG00845003938	1090	1029	908	969
ENSCAFG00845003937	0	0	0	0
ENSCAFG00845003936	0	0	0	0
ENSCAFG00845003935	0	0	0	0
ENSCAFG00845003934	0	0	0	0
ENSCAFG00845003933	312	322	335	315
ENSCAFG00845003932	0	0	0	0
ENSCAFG00845003931	635	654	621	656
ENSCAFG00845003930	85	72	57	71
ENSCAFG00845015909	0	0	0	0
ENSCAFG00845015907	0	0	0	0
ENSCAFG00845015908	0	0	2	0
ENSCAFG00845015905	0	0	0	0

ENSCAFG00845015906	0	0	0	0
ENSCAFG00845015903	4	16	4	13
ENSCAFG00845015904	278	284	331	364
ENSCAFG00845015901	0	0	0	0
ENSCAFG00845015902	45	33	52	46
ENSCAFG00845015900	0	0	0	0
ENSCAFG00845013395	70	62	54	42
ENSCAFG00845013394	0	0	0	0
ENSCAFG00845013397	626	656	659	631
ENSCAFG00845013396	312	314	325	319
ENSCAFG00845013391	41	62	99	80
ENSCAFG00845013390	366	370	328	342
ENSCAFG00845013393	137	146	148	138
ENSCAFG00845013392	0	0	0	0
ENSCAFG00845025377	0	0	0	0
ENSCAFG00845025378	0	0	0	0
ENSCAFG00845025379	0	0	2	0
ENSCAFG00845025373	1516	1490	1305	1327
ENSCAFG00845025375	1540	1539	1733	1719
ENSCAFG00845025376	0	0	0	0
ENSCAFG00845025370	1	1	0	0
ENSCAFG00845025371	4	14	8	12
ENSCAFG00845025372	58	34	40	56
ENSCAFG00845013399	0	0	0	0
ENSCAFG00845013398	382	356	409	497
ENSCAFG00845013384	190	206	125	121
ENSCAFG00845013383	790	733	741	758
ENSCAFG00845013386	0	0	0	0
ENSCAFG00845013385	96	75	98	74
ENSCAFG00845013380	0	0	0	0
ENSCAFG00845013382	0	0	0	0
ENSCAFG00845013381	0	1	0	0
ENSCAFG00845025366	2	2	1	2
ENSCAFG00845025367	11	12	11	22
ENSCAFG00845025368	72	59	64	62
ENSCAFG00845025369	0	0	0	0
ENSCAFG00845025362	148	115	125	135
ENSCAFG00845025363	602	631	576	556
ENSCAFG00845025364	1886	1783	1733	1650
ENSCAFG00845025365	0	0	0	0
ENSCAFG00845025360	414	421	500	462

ENSCAFG00845025361	0	0	0	0
ENSCAFG00845013388	0	0	0	1
ENSCAFG00845013387	629	519	510	482
ENSCAFG00845013389	4065	3787	3416	3394
ENSCAFG00845013373	1566	1652	1515	1504
ENSCAFG00845013372	0	0	0	0
ENSCAFG00845013375	0	0	0	0
ENSCAFG00845013374	1337	1199	1453	1490
ENSCAFG00845013371	248	275	323	306
ENSCAFG00845013370	0	0	0	0
ENSCAFG00845025359	0	0	0	0
ENSCAFG00845001388	1063	1127	1186	1204
ENSCAFG00845025355	802	747	645	629
ENSCAFG00845001389	0	0	0	0
ENSCAFG00845025356	0	0	0	0
ENSCAFG00845001386	101	129	104	137
ENSCAFG00845025357	0	1	1	6
ENSCAFG00845001387	0	0	0	0
ENSCAFG00845025358	0	0	0	0
ENSCAFG00845001384	226	205	229	260
ENSCAFG00845001385	306	268	259	245
ENSCAFG00845025352	2410	2369	2837	3074
ENSCAFG00845001382	27	20	10	28
ENSCAFG00845025353	2777	2606	2293	2384
ENSCAFG00845001383	123	130	117	125
ENSCAFG00845025354	157	131	109	112
ENSCAFG00845001380	0	0	0	1
ENSCAFG00845001381	86	59	69	52
ENSCAFG00845025350	546	574	517	539
ENSCAFG00845013377	0	0	0	0
ENSCAFG00845013376	407	393	378	344
ENSCAFG00845013379	374	441	401	427
ENSCAFG00845013378	0	0	0	0
ENSCAFG00845013362	0	0	0	0
ENSCAFG00845013361	31	40	45	45
ENSCAFG00845013364	1828	1691	1559	1433
ENSCAFG00845013363	103	118	99	100
ENSCAFG00845013360	0	0	0	0
ENSCAFG00845025348	2	0	3	0
ENSCAFG00845025349	0	0	0	0
ENSCAFG00845001399	860	842	814	899

ENSCAFG00845025344	4	2	6	3
ENSCAFG00845025345	0	0	0	1
ENSCAFG00845001397	920	871	838	837
ENSCAFG00845025346	366	360	398	453
ENSCAFG00845001398	0	0	0	0
ENSCAFG00845025347	1643	1551	1682	1698
ENSCAFG00845001395	1	1	2	1
ENSCAFG00845025340	0	0	0	0
ENSCAFG00845001396	7	5	6	5
ENSCAFG00845025341	7	31	28	20
ENSCAFG00845001393	1	1	0	0
ENSCAFG00845025342	792	807	888	904
ENSCAFG00845001394	0	0	1	0
ENSCAFG00845025343	0	0	0	0
ENSCAFG00845001391	3	3	4	6
ENSCAFG00845001392	1596	1582	1623	1528
ENSCAFG00845001390	0	0	0	0
ENSCAFG00845013369	555	567	478	559
ENSCAFG00845013366	65	55	62	73
ENSCAFG00845013365	0	0	0	0
ENSCAFG00845013368	38	36	63	46
ENSCAFG00845013367	0	0	0	0
ENSCAFG00845013351	221	190	206	218
ENSCAFG00845013350	1	1	3	0
ENSCAFG00845013353	2470	2251	2276	2381
ENSCAFG00845013352	0	0	0	0
ENSCAFG00845025337	301	312	275	309
ENSCAFG00845025338	397	340	294	352
ENSCAFG00845027999	1018	995	927	993
ENSCAFG00845001368	766	711	609	741
ENSCAFG00845025339	0	0	0	0
ENSCAFG00845001369	0	0	0	0
ENSCAFG00845001366	0	0	0	0
ENSCAFG00845025333	310	306	260	266
ENSCAFG00845027996	1	2	0	0
ENSCAFG00845001367	5	8	1	3
ENSCAFG00845025334	0	0	0	0
ENSCAFG00845027995	0	0	0	0
ENSCAFG00845001364	0	0	0	0
ENSCAFG00845025335	3788	3753	3483	3549
ENSCAFG00845027998	117	95	119	103

ENSCAFG00845001365	0	0	0	0
ENSCAFG00845025336	0	0	0	0
ENSCAFG00845027997	0	0	0	0
ENSCAFG00845001362	0	0	0	0
ENSCAFG00845027992	777	683	538	563
ENSCAFG00845001363	351	390	251	234
ENSCAFG00845027991	0	0	0	0
ENSCAFG00845001360	485	520	554	540
ENSCAFG00845025331	3	8	6	4
ENSCAFG00845027994	176	188	128	136
ENSCAFG00845001361	2	0	0	0
ENSCAFG00845025332	1	2	7	4
ENSCAFG00845027993	1386	1422	1324	1389
ENSCAFG00845027990	0	0	0	0
ENSCAFG00845013359	17	9	14	5
ENSCAFG00845013358	315	277	275	256
ENSCAFG00845013355	655	607	753	713
ENSCAFG00845013354	1	2	2	0
ENSCAFG00845013357	2	0	1	0
ENSCAFG00845013356	30	56	43	54
ENSCAFG00845013340	77	73	42	54
ENSCAFG00845013342	1	0	6	1
ENSCAFG00845013341	782	903	732	849
ENSCAFG00845025326	0	0	0	0
ENSCAFG00845027989	0	0	0	0
ENSCAFG00845025327	878	773	727	667
ENSCAFG00845027988	3	0	0	0
ENSCAFG00845001379	32	25	22	10
ENSCAFG00845025328	2721	2647	2565	2567
ENSCAFG00845025329	0	0	0	0
ENSCAFG00845001377	1	1	0	0
ENSCAFG00845025322	1910	1757	1884	2061
ENSCAFG00845027985	1509	1435	1323	1372
ENSCAFG00845001378	681	684	649	594
ENSCAFG00845027984	215	249	247	240
ENSCAFG00845001375	17	20	15	24
ENSCAFG00845025324	597	581	596	667
ENSCAFG00845027987	0	0	0	0
ENSCAFG00845001376	0	3	0	0
ENSCAFG00845025325	2	0	0	3
ENSCAFG00845027986	5638	5384	5340	5453

ENSCAFG00845001373	7344	6923	7100	7420
ENSCAFG00845027981	5	5	5	3
ENSCAFG00845001374	0	0	0	0
ENSCAFG00845027980	0	0	0	0
ENSCAFG00845001371	374	372	300	342
ENSCAFG00845025320	0	0	0	0
ENSCAFG00845027983	0	1	1	0
ENSCAFG00845001372	4821	4567	4232	4386
ENSCAFG00845025321	7057	6723	6440	6548
ENSCAFG00845027982	625	638	528	568
ENSCAFG00845001370	26	15	27	19
ENSCAFG00845013348	195	192	114	154
ENSCAFG00845013347	0	0	0	0
ENSCAFG00845013349	0	0	0	0
ENSCAFG00845013344	327	312	342	387
ENSCAFG00845013343	2034	2112	1947	1939
ENSCAFG00845013346	17	11	9	6
ENSCAFG00845013345	0	0	0	0
ENSCAFG00845015992	0	0	0	0
ENSCAFG00845015993	0	1	0	0
ENSCAFG00845013331	0	0	0	0
ENSCAFG00845015990	0	0	0	0
ENSCAFG00845013330	927	799	860	801
ENSCAFG00845015991	0	0	0	2
ENSCAFG00845025319	279	262	264	278
ENSCAFG00845001348	10	12	4	6
ENSCAFG00845025315	0	0	0	0
ENSCAFG00845027978	0	0	0	0
ENSCAFG00845001349	602	532	474	571
ENSCAFG00845025316	653	613	638	677
ENSCAFG00845027977	141	130	144	136
ENSCAFG00845001346	0	0	0	0
ENSCAFG00845025317	1189	1083	1117	1106
ENSCAFG00845001347	0	2	0	0
ENSCAFG00845025318	0	0	0	0
ENSCAFG00845027979	16	16	18	19
ENSCAFG00845001344	2	1	3	4
ENSCAFG00845025311	383	403	372	358
ENSCAFG00845027974	524	573	534	506
ENSCAFG00845001345	0	0	0	0
ENSCAFG00845025312	4	2	2	2

ENSCAFG00845027973	0	0	0	0
ENSCAFG00845001342	119	120	107	114
ENSCAFG00845025313	0	1	4	1
ENSCAFG00845027976	0	0	0	0
ENSCAFG00845001343	82	62	83	66
ENSCAFG00845025314	977	872	985	966
ENSCAFG00845027975	90	107	59	52
ENSCAFG00845001340	0	0	0	0
ENSCAFG00845027970	20	27	28	28
ENSCAFG00845001341	1018	964	973	930
ENSCAFG00845027972	0	0	0	0
ENSCAFG00845025310	0	0	0	0
ENSCAFG00845027971	0	0	0	0
ENSCAFG00845013337	0	0	0	0
ENSCAFG00845013336	36	30	29	40
ENSCAFG00845013339	542	462	598	588
ENSCAFG00845015998	0	0	0	0
ENSCAFG00845013338	0	0	0	0
ENSCAFG00845015999	1080	1002	941	1002
ENSCAFG00845013333	0	0	0	0
ENSCAFG00845015996	0	0	0	0
ENSCAFG00845013332	0	0	0	0
ENSCAFG00845015997	564	580	515	479
ENSCAFG00845013335	15	14	10	18
ENSCAFG00845015994	0	0	0	0
ENSCAFG00845013334	0	0	0	0
ENSCAFG00845015995	0	0	0	0
ENSCAFG00845015981	0	0	0	0
ENSCAFG00845015982	0	0	0	0
ENSCAFG00845013320	439	450	386	422
ENSCAFG00845015980	539	509	489	564
ENSCAFG00845025308	0	0	0	0
ENSCAFG00845025309	263	203	245	233
ENSCAFG00845001359	0	0	0	0
ENSCAFG00845025304	0	0	0	1
ENSCAFG00845027967	0	0	0	0
ENSCAFG00845025305	2	1	0	0
ENSCAFG00845027966	3731	3478	3450	3627
ENSCAFG00845001357	95	59	86	60
ENSCAFG00845025306	0	5	1	3
ENSCAFG00845027969	0	0	0	0

ENSCAFG00845001358	52	69	57	52
ENSCAFG00845025307	5	4	1	5
ENSCAFG00845027968	3	12	4	5
ENSCAFG00845001355	12	20	17	17
ENSCAFG00845025300	741	730	740	829
ENSCAFG00845027963	0	0	0	0
ENSCAFG00845001356	429	390	354	387
ENSCAFG00845027962	250	244	256	306
ENSCAFG00845001353	449	477	330	387
ENSCAFG00845025302	4	8	3	8
ENSCAFG00845027965	1532	1563	1387	1400
ENSCAFG00845001354	1532	1398	1482	1496
ENSCAFG00845025303	4402	4105	4177	4163
ENSCAFG00845027964	0	0	0	0
ENSCAFG00845001351	0	0	0	0
ENSCAFG00845001352	0	0	0	0
ENSCAFG00845027961	407	398	399	424
ENSCAFG00845001350	181	157	138	191
ENSCAFG00845027960	0	0	0	1
ENSCAFG00845013329	76	66	114	80
ENSCAFG00845013326	0	0	0	0
ENSCAFG00845015989	426	350	348	400
ENSCAFG00845013325	0	0	0	0
ENSCAFG00845013328	1157	1114	1147	985
ENSCAFG00845015987	3	0	1	0
ENSCAFG00845013327	22	22	14	24
ENSCAFG00845015988	197	191	229	211
ENSCAFG00845013322	4	3	7	5
ENSCAFG00845015985	0	0	0	0
ENSCAFG00845013321	0	0	0	0
ENSCAFG00845015986	105	110	98	85
ENSCAFG00845013324	1	0	0	0
ENSCAFG00845015983	1	6	1	3
ENSCAFG00845013323	3	3	0	2
ENSCAFG00845015984	0	0	0	0
ENSCAFG00845013430	0	0	0	0
ENSCAFG00845001449	1515	1433	1340	1380
ENSCAFG00845001447	0	0	0	0
ENSCAFG00845025414	0	0	0	0
ENSCAFG00845001448	492	526	472	540
ENSCAFG00845025415	84	89	56	63

ENSCAFG00845001445	735	751	764	851
ENSCAFG00845025416	130	147	174	142
ENSCAFG00845001446	0	0	0	1
ENSCAFG00845001443	3	0	1	3
ENSCAFG00845025410	0	2	1	3
ENSCAFG00845001444	599	585	667	633
ENSCAFG00845025411	55	61	55	63
ENSCAFG00845001441	0	0	0	0
ENSCAFG00845025412	0	0	0	0
ENSCAFG00845001442	0	0	0	0
ENSCAFG00845025413	0	0	0	0
ENSCAFG00845001440	0	0	0	0
ENSCAFG00845013439	62	67	79	59
ENSCAFG00845013436	0	0	0	0
ENSCAFG00845013435	2	2	0	2
ENSCAFG00845013438	372	343	320	324
ENSCAFG00845013437	0	0	0	0
ENSCAFG00845013432	309	307	268	281
ENSCAFG00845013431	181	221	175	188
ENSCAFG00845013434	28	19	14	14
ENSCAFG00845013433	4	3	4	7
ENSCAFG00845025407	149	169	141	170
ENSCAFG00845025408	494	496	433	352
ENSCAFG00845025409	0	0	0	0
ENSCAFG00845001458	0	0	0	0
ENSCAFG00845025403	0	0	0	0
ENSCAFG00845001459	0	0	0	0
ENSCAFG00845025404	0	0	0	0
ENSCAFG00845001456	0	0	0	0
ENSCAFG00845025405	0	0	0	0
ENSCAFG00845001457	2868	2835	2573	2743
ENSCAFG00845025406	554	502	555	540
ENSCAFG00845001454	1794	1577	1876	1666
ENSCAFG00845001455	0	0	0	0
ENSCAFG00845025400	0	0	0	0
ENSCAFG00845001452	954	970	827	842
ENSCAFG00845025401	0	0	0	1
ENSCAFG00845001453	15	24	25	24
ENSCAFG00845025402	554	494	621	668
ENSCAFG00845001450	991	971	969	932
ENSCAFG00845001451	33	30	27	33

ENSCAFG00845013429	790	759	777	750
ENSCAFG00845013428	216	200	196	254
ENSCAFG00845013425	0	0	0	0
ENSCAFG00845013424	0	0	0	0
ENSCAFG00845013427	21	30	27	20
ENSCAFG00845013426	0	0	0	0
ENSCAFG00845013421	0	0	0	0
ENSCAFG00845013420	1705	1760	1450	1485
ENSCAFG00845013423	1425	1470	1485	1497
ENSCAFG00845013422	404	332	344	361
ENSCAFG00845001429	0	0	0	1
ENSCAFG00845001427	1	0	11	5
ENSCAFG00845001428	78	112	77	82
ENSCAFG00845001425	372	398	346	320
ENSCAFG00845001426	41	38	47	64
ENSCAFG00845001423	0	0	0	0
ENSCAFG00845001424	1	0	4	1
ENSCAFG00845001421	118	99	114	114
ENSCAFG00845001422	1995	1934	1888	1972
ENSCAFG00845001420	0	0	0	0
ENSCAFG00845013418	0	0	0	0
ENSCAFG00845013417	3	0	0	0
ENSCAFG00845013419	0	0	0	0
ENSCAFG00845013414	1969	1818	1982	1966
ENSCAFG00845013413	0	0	0	0
ENSCAFG00845013416	605	567	595	640
ENSCAFG00845013415	2	2	2	2
ENSCAFG00845013410	0	0	0	0
ENSCAFG00845013412	162	126	128	126
ENSCAFG00845013411	1088	1055	1029	986
ENSCAFG00845001438	221	168	188	154
ENSCAFG00845001439	0	0	0	0
ENSCAFG00845001436	0	0	0	0
ENSCAFG00845001437	0	0	0	0
ENSCAFG00845001434	34	36	27	28
ENSCAFG00845001435	0	0	0	0
ENSCAFG00845001432	988	983	983	970
ENSCAFG00845001433	5767	5797	4947	5312
ENSCAFG00845001430	1	0	0	1
ENSCAFG00845001431	74	67	54	50
ENSCAFG00845013407	1297	1291	1063	1091

ENSCAFG00845013406	343	330	264	308
ENSCAFG00845013409	5	4	2	6
ENSCAFG00845013408	0	0	0	0
ENSCAFG00845013403	24	22	25	30
ENSCAFG00845013402	836	821	831	871
ENSCAFG00845013405	0	0	0	0
ENSCAFG00845013404	0	0	0	0
ENSCAFG00845013401	0	0	0	0
ENSCAFG00845013400	0	0	0	0
ENSCAFG00845001409	0	0	0	0
ENSCAFG00845001407	0	0	0	0
ENSCAFG00845001408	2147	2183	1951	1943
ENSCAFG00845001405	2	2	0	0
ENSCAFG00845001406	0	0	0	0
ENSCAFG00845001403	598	591	491	505
ENSCAFG00845001404	745	718	759	707
ENSCAFG00845001401	0	0	0	0
ENSCAFG00845001402	0	0	0	0
ENSCAFG00845001400	3	0	0	0
ENSCAFG00845001418	3926	3665	3794	3830
ENSCAFG00845001419	2	3	3	6
ENSCAFG00845001416	38	63	30	48
ENSCAFG00845001417	1919	1877	1193	1179
ENSCAFG00845001414	0	0	0	0
ENSCAFG00845001415	0	0	0	0
ENSCAFG00845001412	239	169	206	237
ENSCAFG00845001413	0	0	0	0
ENSCAFG00845001410	3	1	1	6
ENSCAFG00845001411	0	1	0	0
ENSCAFG00845025499	1028	1031	1020	1056
ENSCAFG00845025494	0	8	3	0
ENSCAFG00845025495	0	0	0	0
ENSCAFG00845025496	115	85	66	70
ENSCAFG00845025497	261	271	207	222
ENSCAFG00845025490	12	13	12	13
ENSCAFG00845025491	0	0	0	0
ENSCAFG00845025492	0	0	0	0
ENSCAFG00845025493	638	616	712	660
ENSCAFG00845025487	0	0	0	0
ENSCAFG00845025488	5	5	3	5
ENSCAFG00845025489	0	0	0	1

ENSCAFG00845025483	3	5	3	1
ENSCAFG00845025484	0	0	0	1
ENSCAFG00845025485	446	419	482	497
ENSCAFG00845025486	2	2	3	3
ENSCAFG00845025480	43	30	20	23
ENSCAFG00845025481	0	0	0	1
ENSCAFG00845025482	3	0	2	2
ENSCAFG00845013494	685	649	481	453
ENSCAFG00845013493	2743	2909	2700	2777
ENSCAFG00845013496	1836	1801	1739	1737
ENSCAFG00845013495	0	0	2	0
ENSCAFG00845013490	2818	2636	2570	2600
ENSCAFG00845013492	27	16	22	22
ENSCAFG00845013491	0	1	0	0
ENSCAFG00845025476	0	0	3	0
ENSCAFG00845025477	1	0	4	0
ENSCAFG00845025478	873	800	764	794
ENSCAFG00845025479	0	0	0	0
ENSCAFG00845025472	0	0	0	0
ENSCAFG00845025473	0	0	0	0
ENSCAFG00845025474	0	0	0	0
ENSCAFG00845025475	1436	1424	1289	1265
ENSCAFG00845025470	0	0	0	0
ENSCAFG00845025471	9	16	5	10
ENSCAFG00845013498	0	0	0	0
ENSCAFG00845013497	10	1	3	3
ENSCAFG00845013499	0	0	0	0
ENSCAFG00845013483	32	21	17	25
ENSCAFG00845013482	8	10	5	11
ENSCAFG00845013485	2439	2439	2472	2402
ENSCAFG00845013484	2	5	13	8
ENSCAFG00845013481	2216	2260	1860	1907
ENSCAFG00845013480	0	0	1	1
ENSCAFG00845025469	0	0	0	0
ENSCAFG00845025465	3	1	10	8
ENSCAFG00845025466	0	0	0	0
ENSCAFG00845025467	0	0	0	0
ENSCAFG00845025468	487	411	447	393
ENSCAFG00845025461	2024	2080	2387	2431
ENSCAFG00845025462	238	248	160	191
ENSCAFG00845025463	0	0	0	0

ENSCAFG00845025464	20	7	10	8
ENSCAFG00845025460	1	1	2	0
ENSCAFG00845013487	138	154	153	152
ENSCAFG00845013486	0	0	0	0
ENSCAFG00845013489	13	14	2	1
ENSCAFG00845013488	0	0	0	0
ENSCAFG00845013472	1	6	1	3
ENSCAFG00845013471	66	77	88	76
ENSCAFG00845013474	9	4	8	6
ENSCAFG00845013473	23056	21917	23631	23686
ENSCAFG00845013470	0	0	0	0
ENSCAFG00845025458	182	168	169	190
ENSCAFG00845025459	281	282	256	367
ENSCAFG00845001489	0	0	0	0
ENSCAFG00845001487	43	41	32	48
ENSCAFG00845025454	0	1	0	0
ENSCAFG00845001488	266	210	172	241
ENSCAFG00845025455	3	3	5	6
ENSCAFG00845001485	0	0	0	0
ENSCAFG00845025456	5586	5489	5413	5422
ENSCAFG00845001486	72	71	40	32
ENSCAFG00845025457	6831	6478	6707	7003
ENSCAFG00845001483	43	52	54	66
ENSCAFG00845025450	3	2	2	3
ENSCAFG00845001484	0	0	0	0
ENSCAFG00845025451	0	0	0	0
ENSCAFG00845001481	0	0	0	0
ENSCAFG00845025452	4	8	5	1
ENSCAFG00845001482	7	6	12	9
ENSCAFG00845025453	1	0	1	0
ENSCAFG00845001480	320	330	323	338
ENSCAFG00845013479	0	1	0	0
ENSCAFG00845013476	2544	2431	2560	2324
ENSCAFG00845013475	304	321	340	342
ENSCAFG00845013478	2	1	6	4
ENSCAFG00845013477	0	0	0	0
ENSCAFG00845013461	25	12	14	19
ENSCAFG00845013460	0	0	0	0
ENSCAFG00845013463	36	40	37	50
ENSCAFG00845013462	331	302	299	297
ENSCAFG00845025447	663	615	698	657

ENSCAFG00845025448	1152	1007	1025	1071
ENSCAFG00845001498	0	0	0	0
ENSCAFG00845001499	0	0	0	0
ENSCAFG00845025444	45	30	28	16
ENSCAFG00845001496	1	0	2	0
ENSCAFG00845025445	251	231	253	243
ENSCAFG00845001497	0	0	1	0
ENSCAFG00845025446	2	2	6	0
ENSCAFG00845001494	0	0	0	0
ENSCAFG00845001495	2	5	4	9
ENSCAFG00845025440	0	0	0	0
ENSCAFG00845001492	475	428	432	412
ENSCAFG00845025441	1780	1682	1611	1668
ENSCAFG00845001493	35	26	9	19
ENSCAFG00845025442	0	0	2	0
ENSCAFG00845001490	0	0	0	0
ENSCAFG00845001491	40	34	64	74
ENSCAFG00845013469	0	0	0	0
ENSCAFG00845013468	8	7	15	6
ENSCAFG00845013465	76	109	88	108
ENSCAFG00845013464	1	1	0	0
ENSCAFG00845013467	356	260	341	357
ENSCAFG00845013466	0	0	0	0
ENSCAFG00845013450	918	836	701	694
ENSCAFG00845013452	0	0	0	0
ENSCAFG00845013451	0	0	0	0
ENSCAFG00845001469	14	9	3	4
ENSCAFG00845025436	2	1	1	1
ENSCAFG00845025437	0	0	0	0
ENSCAFG00845001467	0	4	2	3
ENSCAFG00845025438	680	671	656	696
ENSCAFG00845001468	934	917	982	924
ENSCAFG00845025439	0	0	1	1
ENSCAFG00845001465	0	0	0	0
ENSCAFG00845025432	2360	2347	2486	2541
ENSCAFG00845001466	0	0	1	0
ENSCAFG00845025433	0	0	0	0
ENSCAFG00845001463	0	0	0	0
ENSCAFG00845025434	397	377	373	421
ENSCAFG00845001464	0	0	0	0
ENSCAFG00845025435	0	0	0	0

ENSCAFG00845001461	0	0	3	3
ENSCAFG00845001462	158	148	145	130
ENSCAFG00845025430	0	0	0	0
ENSCAFG00845001460	10	1	8	5
ENSCAFG00845025431	525	470	428	478
ENSCAFG00845013458	0	0	0	0
ENSCAFG00845013457	2776	2584	2262	2447
ENSCAFG00845013459	5620	5298	5730	5820
ENSCAFG00845013454	2132	2149	2012	2023
ENSCAFG00845013453	0	0	0	0
ENSCAFG00845013456	0	0	0	0
ENSCAFG00845013455	11	3	20	6
ENSCAFG00845013441	365	371	411	361
ENSCAFG00845013440	1647	1537	1443	1493
ENSCAFG00845025429	0	0	0	0
ENSCAFG00845025425	0	0	0	0
ENSCAFG00845025426	4	7	2	3
ENSCAFG00845001478	776	833	723	685
ENSCAFG00845025427	3	0	0	0
ENSCAFG00845001479	493	499	458	500
ENSCAFG00845025428	0	0	1	2
ENSCAFG00845001476	0	1	0	2
ENSCAFG00845025421	0	0	0	0
ENSCAFG00845001477	0	0	0	0
ENSCAFG00845025422	17	21	23	19
ENSCAFG00845001474	0	0	0	0
ENSCAFG00845025423	115	85	91	84
ENSCAFG00845001475	12	12	20	22
ENSCAFG00845025424	1	2	0	3
ENSCAFG00845001472	2	1	0	1
ENSCAFG00845001473	0	0	0	0
ENSCAFG00845001470	0	2	0	4
ENSCAFG00845001471	1295	1352	1222	1277
ENSCAFG00845025420	0	0	0	0
ENSCAFG00845013447	907	966	886	902
ENSCAFG00845013446	120	148	127	157
ENSCAFG00845013449	0	0	0	0
ENSCAFG00845013448	9	5	3	11
ENSCAFG00845013443	2209	2143	2135	2219
ENSCAFG00845013442	168	144	157	178
ENSCAFG00845013445	235	230	102	134

ENSCAFG00845013444	0	0	0	0
ENSCAFG00845001609	22	22	27	28
ENSCAFG00845001607	0	0	0	0
ENSCAFG00845001608	208	185	175	183
ENSCAFG00845001605	1810	1628	1782	1941
ENSCAFG00845001606	42	37	47	61
ENSCAFG00845001603	0	1	0	0
ENSCAFG00845001604	820	865	845	851
ENSCAFG00845001601	2286	2246	2302	2406
ENSCAFG00845001602	4	6	2	3
ENSCAFG00845001600	451	477	514	490
ENSCAFG00845001618	0	1	0	2
ENSCAFG00845001619	201	201	192	189
ENSCAFG00845001616	126	103	90	94
ENSCAFG00845001617	2558	2462	2233	2224
ENSCAFG00845001614	0	0	0	0
ENSCAFG00845001615	0	0	1	0
ENSCAFG00845001612	4	1	1	1
ENSCAFG00845001613	0	0	0	0
ENSCAFG00845001610	2	1	2	1
ENSCAFG00845001611	3002	2881	3227	3374
ENSCAFG00845013551	478	507	501	452
ENSCAFG00845013550	0	1	3	2
ENSCAFG00845025539	14	14	26	30
ENSCAFG00845001568	1814	1696	1931	1881
ENSCAFG00845025535	2873	2759	2761	2839
ENSCAFG00845001569	51	38	40	34
ENSCAFG00845025536	3	1	1	0
ENSCAFG00845001566	0	0	0	0
ENSCAFG00845025537	2	6	9	5
ENSCAFG00845001567	0	0	0	0
ENSCAFG00845025538	1	0	0	0
ENSCAFG00845001564	349	372	373	343
ENSCAFG00845025531	583	545	532	588
ENSCAFG00845001565	0	0	0	0
ENSCAFG00845025532	3005	2888	2813	2834
ENSCAFG00845001562	1110	1040	983	957
ENSCAFG00845025533	929	966	875	874
ENSCAFG00845001563	0	0	0	0
ENSCAFG00845025534	0	1	2	0
ENSCAFG00845001560	1	0	1	0

ENSCAFG00845001561	0	0	0	0
ENSCAFG00845025530	307	259	368	399
ENSCAFG00845013557	297	300	322	310
ENSCAFG00845013556	599	607	523	569
ENSCAFG00845013559	0	1	0	1
ENSCAFG00845013558	0	0	0	0
ENSCAFG00845013553	1325	1174	1318	1413
ENSCAFG00845013552	7140	7092	7236	7435
ENSCAFG00845013555	0	0	0	0
ENSCAFG00845013554	1010	963	897	836
ENSCAFG00845013540	1268	1130	986	1005
ENSCAFG00845025528	0	0	0	1
ENSCAFG00845025529	3666	3457	3510	3471
ENSCAFG00845001579	649	654	674	707
ENSCAFG00845025524	0	0	0	0
ENSCAFG00845025525	0	0	0	0
ENSCAFG00845001577	543	493	571	574
ENSCAFG00845025526	2272	2205	2426	2451
ENSCAFG00845001578	2	5	0	0
ENSCAFG00845025527	0	0	0	0
ENSCAFG00845001575	7773	7588	6975	7018
ENSCAFG00845025520	45	26	45	51
ENSCAFG00845001576	0	0	0	0
ENSCAFG00845001573	0	0	0	0
ENSCAFG00845025522	139	111	73	98
ENSCAFG00845001574	763	737	772	725
ENSCAFG00845025523	6	8	11	6
ENSCAFG00845001571	262	246	216	218
ENSCAFG00845001572	84	82	58	70
ENSCAFG00845001570	4052	4033	3815	3832
ENSCAFG00845013549	0	0	0	0
ENSCAFG00845013546	0	0	0	0
ENSCAFG00845013545	5	1	3	1
ENSCAFG00845013548	0	0	0	0
ENSCAFG00845013547	0	0	0	2
ENSCAFG00845013542	0	0	0	0
ENSCAFG00845013541	0	0	0	0
ENSCAFG00845013544	6	15	5	7
ENSCAFG00845013543	0	0	0	0
ENSCAFG00845025517	0	0	0	1
ENSCAFG00845025518	251	257	235	244

ENSCAFG00845001548	0	0	0	0
ENSCAFG00845025519	296	340	392	403
ENSCAFG00845001549	3	0	1	0
ENSCAFG00845001546	82	85	110	129
ENSCAFG00845025513	7	2	8	6
ENSCAFG00845001547	3392	3015	2970	3027
ENSCAFG00845025514	323	312	352	315
ENSCAFG00845001544	1	0	0	0
ENSCAFG00845025515	0	0	0	0
ENSCAFG00845001545	0	0	0	0
ENSCAFG00845025516	1215	1226	1106	1089
ENSCAFG00845001542	455	483	212	188
ENSCAFG00845001543	0	0	0	0
ENSCAFG00845025510	261	245	267	277
ENSCAFG00845001540	0	0	0	0
ENSCAFG00845025511	0	0	0	0
ENSCAFG00845001541	0	0	0	0
ENSCAFG00845025512	4915	4682	4659	4875
ENSCAFG00845013539	3	2	1	0
ENSCAFG00845013538	0	0	0	0
ENSCAFG00845013535	0	0	0	0
ENSCAFG00845013534	2580	2545	2361	2452
ENSCAFG00845013537	0	0	0	0
ENSCAFG00845013536	0	0	0	0
ENSCAFG00845013531	47	49	59	58
ENSCAFG00845013530	0	0	0	0
ENSCAFG00845013533	547	573	499	539
ENSCAFG00845013532	87	91	84	113
ENSCAFG00845025506	0	0	0	0
ENSCAFG00845001559	0	0	0	0
ENSCAFG00845025508	11	10	8	8
ENSCAFG00845025509	2	0	1	0
ENSCAFG00845001557	0	0	0	2
ENSCAFG00845025502	0	1	0	0
ENSCAFG00845001558	0	0	0	0
ENSCAFG00845025503	278	309	310	274
ENSCAFG00845001555	0	0	0	0
ENSCAFG00845025504	1288	1218	1234	1285
ENSCAFG00845001556	0	0	0	0
ENSCAFG00845025505	0	1	0	0
ENSCAFG00845001553	0	0	0	0

ENSCAFG00845001554	221	204	244	264
ENSCAFG00845001551	0	0	0	0
ENSCAFG00845025500	1812	1769	2225	2091
ENSCAFG00845001552	0	0	0	0
ENSCAFG00845001550	2	2	2	5
ENSCAFG00845013528	0	0	0	0
ENSCAFG00845013527	1565	1420	1449	1466
ENSCAFG00845013529	1453	1449	1373	1238
ENSCAFG00845013524	132	115	183	159
ENSCAFG00845013523	0	0	0	0
ENSCAFG00845013526	0	2	0	0
ENSCAFG00845013525	128	105	138	115
ENSCAFG00845013520	1	0	0	0
ENSCAFG00845013522	621	570	680	696
ENSCAFG00845013521	11	16	11	15
ENSCAFG00845001528	2079	1897	2048	2051
ENSCAFG00845001529	0	0	0	0
ENSCAFG00845001526	143	148	140	149
ENSCAFG00845001527	17	24	12	8
ENSCAFG00845001524	0	0	0	0
ENSCAFG00845001525	177	140	188	143
ENSCAFG00845001522	0	0	1	0
ENSCAFG00845001523	15	9	12	8
ENSCAFG00845001520	0	0	0	0
ENSCAFG00845001521	0	0	0	2
ENSCAFG00845013517	0	0	0	0
ENSCAFG00845013516	0	0	0	0
ENSCAFG00845013519	0	0	0	0
ENSCAFG00845013518	2398	2200	2288	2367
ENSCAFG00845013513	418	368	416	401
ENSCAFG00845013512	0	0	0	0
ENSCAFG00845013515	850	815	841	825
ENSCAFG00845013514	0	0	0	0
ENSCAFG00845013511	20	9	7	7
ENSCAFG00845013510	0	0	0	0
ENSCAFG00845001539	0	0	0	1
ENSCAFG00845001537	0	0	0	0
ENSCAFG00845001538	0	0	0	0
ENSCAFG00845001535	0	0	0	0
ENSCAFG00845001536	0	0	0	0
ENSCAFG00845001533	0	0	0	0

ENSCAFG00845001534	0	0	0	0
ENSCAFG00845001531	128	132	173	166
ENSCAFG00845001532	0	0	0	0
ENSCAFG00845001530	1	0	0	0
ENSCAFG00845013509	17	13	42	27
ENSCAFG00845013506	510	472	500	498
ENSCAFG00845013505	1146	1146	1157	1203
ENSCAFG00845013508	0	0	0	0
ENSCAFG00845013507	2	3	3	0
ENSCAFG00845013502	2	1	1	8
ENSCAFG00845013501	1	1	0	0
ENSCAFG00845013504	19	12	29	21
ENSCAFG00845013503	0	0	0	0
ENSCAFG00845013500	0	0	0	0
ENSCAFG00845001508	0	0	0	0
ENSCAFG00845001509	5	2	1	4
ENSCAFG00845001506	18	25	9	15
ENSCAFG00845001507	1	4	0	0
ENSCAFG00845001504	53	40	21	24
ENSCAFG00845001505	232	207	207	206
ENSCAFG00845001502	0	0	0	0
ENSCAFG00845001503	355	264	308	374
ENSCAFG00845001500	0	1	0	0
ENSCAFG00845001501	0	0	0	0
ENSCAFG00845001519	2	2	2	8
ENSCAFG00845001517	243	264	256	287
ENSCAFG00845001518	1844	1720	1905	1910
ENSCAFG00845001515	0	0	0	0
ENSCAFG00845001516	0	0	0	0
ENSCAFG00845001513	674	625	667	630
ENSCAFG00845001514	354	295	270	296
ENSCAFG00845001511	0	1	1	0
ENSCAFG00845001512	3	2	1	2
ENSCAFG00845001510	935	775	785	914
ENSCAFG00845025598	163	153	155	164
ENSCAFG00845025599	5	1	1	2
ENSCAFG00845025593	0	0	0	0
ENSCAFG00845025594	11	5	3	11
ENSCAFG00845025595	52	36	53	32
ENSCAFG00845025596	0	0	0	0
ENSCAFG00845025590	0	0	0	2

ENSCAFG00845025591	0	0	0	0
ENSCAFG00845025592	558	541	594	586
ENSCAFG00845025586	66	63	44	36
ENSCAFG00845025587	340	302	357	349
ENSCAFG00845025582	0	0	0	0
ENSCAFG00845025583	0	4	1	1
ENSCAFG00845025584	0	0	0	0
ENSCAFG00845025585	7	5	5	4
ENSCAFG00845025580	0	0	0	0
ENSCAFG00845025581	3984	3893	3335	3580
ENSCAFG00845013593	105	108	89	112
ENSCAFG00845013592	315	340	276	298
ENSCAFG00845013595	0	0	0	1
ENSCAFG00845013594	341	270	290	314
ENSCAFG00845013591	3191	3118	2963	2993
ENSCAFG00845013590	123	107	102	122
ENSCAFG00845025579	0	0	0	0
ENSCAFG00845025575	0	0	0	0
ENSCAFG00845025576	161	167	108	125
ENSCAFG00845025577	496	444	460	465
ENSCAFG00845025578	4	2	0	1
ENSCAFG00845025571	54	72	53	27
ENSCAFG00845025572	103	96	98	80
ENSCAFG00845025573	1	1	0	0
ENSCAFG00845025574	0	0	0	0
ENSCAFG00845025570	1149	1121	944	919
ENSCAFG00845013597	523	524	528	488
ENSCAFG00845013596	0	0	0	0
ENSCAFG00845013599	0	0	0	0
ENSCAFG00845013598	144	178	194	195
ENSCAFG00845013582	0	0	0	0
ENSCAFG00845013581	0	0	0	0
ENSCAFG00845013584	0	0	0	0
ENSCAFG00845013583	341	344	327	348
ENSCAFG00845013580	0	0	0	0
ENSCAFG00845025568	0	2	5	5
ENSCAFG00845025569	765	700	654	619
ENSCAFG00845025564	3	0	2	1
ENSCAFG00845025565	0	0	0	0
ENSCAFG00845025566	140	143	114	128
ENSCAFG00845025567	0	0	0	0

ENSCAFG00845025561	0	0	0	0
ENSCAFG00845025562	0	0	0	0
ENSCAFG00845025563	0	0	0	0
ENSCAFG00845013589	2086	1941	1804	1776
ENSCAFG00845013586	542	492	431	486
ENSCAFG00845013585	1176	1168	1273	1249
ENSCAFG00845013588	0	0	0	0
ENSCAFG00845013587	0	1	4	5
ENSCAFG00845013571	0	0	0	0
ENSCAFG00845013570	1494	1384	1570	1531
ENSCAFG00845013573	1008	903	1022	1064
ENSCAFG00845013572	6199	5705	6125	6375
ENSCAFG00845025557	333	318	291	336
ENSCAFG00845025558	2292	2275	2378	2373
ENSCAFG00845001588	0	0	0	0
ENSCAFG00845025559	0	0	0	0
ENSCAFG00845001589	0	0	0	0
ENSCAFG00845001586	0	0	0	0
ENSCAFG00845025553	151	151	121	118
ENSCAFG00845001587	1112	997	969	1066
ENSCAFG00845001584	271	211	236	265
ENSCAFG00845025555	1	1	0	1
ENSCAFG00845001585	2	0	1	0
ENSCAFG00845025556	1928	2003	1941	2049
ENSCAFG00845001582	2351	2178	2322	2293
ENSCAFG00845001583	1	0	0	0
ENSCAFG00845025550	1022	974	969	961
ENSCAFG00845001580	4	2	3	1
ENSCAFG00845025551	0	0	0	0
ENSCAFG00845001581	589	599	517	558
ENSCAFG00845013579	0	0	0	0
ENSCAFG00845013578	8	8	8	11
ENSCAFG00845013575	0	0	0	0
ENSCAFG00845013574	1	0	0	0
ENSCAFG00845013577	13	15	8	7
ENSCAFG00845013576	0	0	0	0
ENSCAFG00845013560	450	393	432	398
ENSCAFG00845013562	0	1	0	0
ENSCAFG00845013561	1128	1085	1122	1140
ENSCAFG00845025546	0	0	0	0
ENSCAFG00845025547	0	2	1	0

ENSCAFG00845001599	169	172	189	174
ENSCAFG00845025548	2	1	1	0
ENSCAFG00845025549	262	277	234	204
ENSCAFG00845001597	715	679	724	740
ENSCAFG00845025542	0	0	0	0
ENSCAFG00845001598	0	0	0	0
ENSCAFG00845025543	21	26	12	13
ENSCAFG00845001595	146	176	151	138
ENSCAFG00845025544	552	574	545	529
ENSCAFG00845001596	0	0	0	0
ENSCAFG00845025545	3	4	4	5
ENSCAFG00845001593	0	1	3	1
ENSCAFG00845001594	785	768	748	785
ENSCAFG00845001591	0	1	0	0
ENSCAFG00845025540	30	32	30	37
ENSCAFG00845001592	3	2	2	2
ENSCAFG00845025541	912	827	779	802
ENSCAFG00845001590	1	0	0	0
ENSCAFG00845013568	0	0	3	3
ENSCAFG00845013567	0	0	0	1
ENSCAFG00845013569	3287	3202	3158	3307
ENSCAFG00845013564	350	335	306	368
ENSCAFG00845013563	0	1	1	4
ENSCAFG00845013566	368	318	337	303
ENSCAFG00845013565	181	159	96	58
ENSCAFG00845023095	835	789	744	686
ENSCAFG00845023094	0	0	0	0
ENSCAFG00845023093	301	279	284	322
ENSCAFG00845023092	670	696	515	523
ENSCAFG00845023099	961	839	904	933
ENSCAFG00845023098	1449	1332	1491	1367
ENSCAFG00845023097	0	0	0	0
ENSCAFG00845023096	248	219	247	209
ENSCAFG00845023091	42	31	22	25
ENSCAFG00845023090	0	3	1	1
ENSCAFG00845023089	712	691	885	904
ENSCAFG00845023084	0	2	0	1
ENSCAFG00845023083	0	0	0	0
ENSCAFG00845023082	938	923	938	913
ENSCAFG00845023081	0	2	4	1
ENSCAFG00845023088	0	0	0	0

ENSCAFG00845023087	196	203	163	126
ENSCAFG00845023086	2	2	4	3
ENSCAFG00845023080	0	0	0	0
ENSCAFG00845001728	297	246	291	313
ENSCAFG00845001729	0	6	0	1
ENSCAFG00845001726	122	131	116	110
ENSCAFG00845001727	0	0	0	0
ENSCAFG00845001724	3	5	3	0
ENSCAFG00845001725	24	34	23	20
ENSCAFG00845001722	980	903	1124	928
ENSCAFG00845001723	1966	2022	1663	1737
ENSCAFG00845001720	1	1	16	4
ENSCAFG00845001721	214	210	244	245
ENSCAFG00845013719	0	0	0	0
ENSCAFG00845013718	0	0	0	0
ENSCAFG00845013715	4709	4517	4151	4076
ENSCAFG00845013714	0	0	0	0
ENSCAFG00845013717	508	514	512	516
ENSCAFG00845013716	18	26	7	11
ENSCAFG00845013711	28	35	21	39
ENSCAFG00845013710	1245	1249	1095	1160
ENSCAFG00845013713	551	583	535	543
ENSCAFG00845013712	4226	4113	3611	3710
ENSCAFG00845001739	2169	2092	2070	2129
ENSCAFG00845001737	0	1	1	0
ENSCAFG00845001738	2060	2028	2196	2273
ENSCAFG00845001735	12	12	0	8
ENSCAFG00845001736	0	0	0	0
ENSCAFG00845001733	3	2	0	1
ENSCAFG00845001734	0	0	0	0
ENSCAFG00845001731	1483	1403	1227	1353
ENSCAFG00845001732	0	2	0	0
ENSCAFG00845001730	1117	1070	1125	1059
ENSCAFG00845013708	0	0	0	0
ENSCAFG00845013707	635	591	570	597
ENSCAFG00845013709	0	0	0	0
ENSCAFG00845013704	0	0	0	0
ENSCAFG00845013703	2	2	2	3
ENSCAFG00845013706	0	1	0	1
ENSCAFG00845013705	96	95	73	100
ENSCAFG00845013700	2	2	0	2

ENSCAFG00845013702	2874	2631	2732	2749
ENSCAFG00845013701	670	740	647	677
ENSCAFG00845001708	0	0	0	0
ENSCAFG00845001709	0	0	0	0
ENSCAFG00845001706	149	162	124	165
ENSCAFG00845001707	0	0	0	0
ENSCAFG00845001704	1104	956	1048	908
ENSCAFG00845001705	12	12	14	9
ENSCAFG00845001702	6	8	10	9
ENSCAFG00845001703	144	100	84	86
ENSCAFG00845001700	0	0	0	0
ENSCAFG00845001701	0	2	0	1
ENSCAFG00845001719	122	133	101	134
ENSCAFG00845001717	14	17	29	20
ENSCAFG00845001718	0	0	0	0
ENSCAFG00845001715	0	0	0	0
ENSCAFG00845001716	894	941	812	826
ENSCAFG00845001713	0	0	0	0
ENSCAFG00845001714	0	0	0	0
ENSCAFG00845001711	0	0	0	0
ENSCAFG00845001712	636	625	689	691
ENSCAFG00845001710	5	8	8	16
ENSCAFG00845013670	0	0	0	0
ENSCAFG00845013672	93	75	80	90
ENSCAFG00845011010	0	0	0	0
ENSCAFG00845013671	37	41	12	25
ENSCAFG00845001689	0	0	0	0
ENSCAFG00845025656	0	0	0	0
ENSCAFG00845025657	60	29	32	40
ENSCAFG00845001687	118	103	114	91
ENSCAFG00845025658	0	0	0	1
ENSCAFG00845001688	171	135	260	278
ENSCAFG00845025659	5	0	0	3
ENSCAFG00845001685	0	0	0	0
ENSCAFG00845025652	0	1	0	0
ENSCAFG00845001686	8721	8550	8517	8696
ENSCAFG00845025653	4	5	6	10
ENSCAFG00845001683	0	0	0	0
ENSCAFG00845025654	0	0	0	0
ENSCAFG00845001684	79	63	72	87
ENSCAFG00845025655	1	1	1	0

ENSCAFG00845001681	0	0	0	0
ENSCAFG00845001682	0	0	0	0
ENSCAFG00845025650	707	652	729	794
ENSCAFG00845001680	0	0	0	1
ENSCAFG00845025651	0	0	0	0
ENSCAFG00845011019	2	0	4	0
ENSCAFG00845011015	0	0	0	0
ENSCAFG00845013678	4	5	6	16
ENSCAFG00845011016	40	42	39	43
ENSCAFG00845013677	632	573	554	645
ENSCAFG00845011017	0	1	4	4
ENSCAFG00845011018	508	465	523	544
ENSCAFG00845013679	346	394	226	263
ENSCAFG00845011011	1	0	0	0
ENSCAFG00845013674	0	0	0	0
ENSCAFG00845011012	1	4	4	2
ENSCAFG00845013673	266	263	258	233
ENSCAFG00845011013	0	1	0	0
ENSCAFG00845013676	1	0	0	1
ENSCAFG00845011014	184	215	208	211
ENSCAFG00845013675	3	5	11	9
ENSCAFG00845013661	0	0	0	1
ENSCAFG00845013660	125	76	113	107
ENSCAFG00845025649	0	0	0	0
ENSCAFG00845025645	470	414	416	452
ENSCAFG00845025646	8	2	4	4
ENSCAFG00845001698	0	0	0	0
ENSCAFG00845025647	6043	5975	6795	6688
ENSCAFG00845001699	4	1	9	14
ENSCAFG00845025648	1841	1781	1620	1557
ENSCAFG00845001696	430	410	435	401
ENSCAFG00845025641	8	2	0	3
ENSCAFG00845001697	305	246	269	261
ENSCAFG00845025642	17	26	3	6
ENSCAFG00845001694	0	0	0	0
ENSCAFG00845025643	1000	852	891	912
ENSCAFG00845001695	0	0	0	0
ENSCAFG00845001692	1433	1422	1475	1418
ENSCAFG00845001693	5264	5208	4912	5235
ENSCAFG00845001690	0	0	0	0
ENSCAFG00845001691	148	159	173	182

ENSCAFG00845025640	0	0	0	0
ENSCAFG00845011008	771	694	699	688
ENSCAFG00845011009	422	375	481	476
ENSCAFG00845011004	0	0	0	0
ENSCAFG00845013667	262	233	216	254
ENSCAFG00845011005	583	506	563	628
ENSCAFG00845013666	1665	1711	1735	1790
ENSCAFG00845011006	182	182	160	199
ENSCAFG00845013669	0	0	0	0
ENSCAFG00845011007	151	152	160	141
ENSCAFG00845013668	0	1	1	0
ENSCAFG00845011000	2845	2694	2431	2574
ENSCAFG00845013663	636	648	675	687
ENSCAFG00845011001	0	2	1	0
ENSCAFG00845013662	684	658	727	641
ENSCAFG00845011002	5	2	2	7
ENSCAFG00845013665	655	533	591	553
ENSCAFG00845011003	139	180	206	165
ENSCAFG00845013664	0	1	2	0
ENSCAFG00845013650	17	14	9	8
ENSCAFG00845025639	0	0	0	0
ENSCAFG00845001669	0	0	0	0
ENSCAFG00845001667	513	488	482	509
ENSCAFG00845025634	0	0	0	0
ENSCAFG00845001668	0	0	0	0
ENSCAFG00845025635	1	2	1	0
ENSCAFG00845001665	1797	1658	1715	1655
ENSCAFG00845025636	0	0	0	0
ENSCAFG00845001666	17	19	30	11
ENSCAFG00845025637	0	0	0	0
ENSCAFG00845001663	3811	3569	3218	3205
ENSCAFG00845025630	2	1	1	0
ENSCAFG00845001664	220	212	160	155
ENSCAFG00845025631	1084	1076	1087	1142
ENSCAFG00845001661	0	0	0	0
ENSCAFG00845025632	99	68	47	84
ENSCAFG00845001662	544	505	798	803
ENSCAFG00845025633	1	1	0	0
ENSCAFG00845001660	0	0	0	0
ENSCAFG00845013659	965	940	888	962
ENSCAFG00845013656	0	0	0	0

ENSCAFG00845013655	0	0	0	0
ENSCAFG00845013658	2702	2578	2652	2865
ENSCAFG00845013657	0	0	0	0
ENSCAFG00845013652	0	0	0	4
ENSCAFG00845013651	0	2	0	0
ENSCAFG00845013654	334	385	245	259
ENSCAFG00845013653	0	0	0	0
ENSCAFG00845025627	0	0	0	0
ENSCAFG00845025628	210	215	223	223
ENSCAFG00845025629	0	0	0	0
ENSCAFG00845001678	0	0	0	0
ENSCAFG00845025623	8	7	7	4
ENSCAFG00845001679	0	0	0	0
ENSCAFG00845025624	0	0	0	0
ENSCAFG00845001676	141	142	74	82
ENSCAFG00845001677	0	0	0	0
ENSCAFG00845025626	1110	1003	1032	1078
ENSCAFG00845001674	0	1	0	0
ENSCAFG00845001675	420	429	342	355
ENSCAFG00845025620	687	688	645	682
ENSCAFG00845001672	0	0	0	0
ENSCAFG00845025621	0	0	0	0
ENSCAFG00845001673	0	0	0	0
ENSCAFG00845025622	0	0	0	0
ENSCAFG00845001670	987	1080	916	1017
ENSCAFG00845001671	45	35	32	32
ENSCAFG00845013649	7	3	5	5
ENSCAFG00845013648	36	33	21	36
ENSCAFG00845013645	1574	1551	1580	1647
ENSCAFG00845013644	0	0	0	0
ENSCAFG00845013647	542	471	519	508
ENSCAFG00845013646	0	0	0	0
ENSCAFG00845013641	3233	3037	3202	3154
ENSCAFG00845013640	917	869	839	859
ENSCAFG00845013643	6349	5905	6693	6612
ENSCAFG00845013642	4	3	3	5
ENSCAFG00845001649	2	0	3	0
ENSCAFG00845025616	0	0	0	0
ENSCAFG00845025617	83	123	113	95
ENSCAFG00845001647	196	194	178	153
ENSCAFG00845025618	0	0	0	0

ENSCAFG00845001648	0	0	0	0
ENSCAFG00845025619	0	0	0	0
ENSCAFG00845001645	1	1	2	2
ENSCAFG00845025612	0	0	0	0
ENSCAFG00845001646	568	532	601	650
ENSCAFG00845001643	263	261	269	282
ENSCAFG00845025614	231	216	182	189
ENSCAFG00845001644	1509	1356	1377	1466
ENSCAFG00845025615	3169	3022	2971	3009
ENSCAFG00845001641	760	668	765	764
ENSCAFG00845001642	4	7	3	2
ENSCAFG00845025610	655	596	602	643
ENSCAFG00845001640	0	0	0	0
ENSCAFG00845025611	1566	1360	1485	1485
ENSCAFG00845013638	0	1	4	0
ENSCAFG00845013637	12	19	6	14
ENSCAFG00845013639	2481	2376	2315	2403
ENSCAFG00845013634	570	506	545	566
ENSCAFG00845013633	0	0	0	0
ENSCAFG00845013636	1298	1192	1132	1021
ENSCAFG00845013635	4	5	1	1
ENSCAFG00845013630	510	512	435	477
ENSCAFG00845013632	0	0	0	0
ENSCAFG00845013631	1393	1478	1309	1446
ENSCAFG00845025609	0	0	0	0
ENSCAFG00845025605	0	0	0	0
ENSCAFG00845025606	5	2	6	7
ENSCAFG00845001658	20	17	19	23
ENSCAFG00845025607	154	151	167	210
ENSCAFG00845001659	152	155	168	166
ENSCAFG00845025608	90	88	94	78
ENSCAFG00845001656	0	0	0	0
ENSCAFG00845025601	970	980	761	784
ENSCAFG00845001657	880	880	818	908
ENSCAFG00845025602	5	14	8	7
ENSCAFG00845001654	1	1	4	2
ENSCAFG00845025603	0	0	0	0
ENSCAFG00845001655	1	7	6	0
ENSCAFG00845025604	0	0	0	0
ENSCAFG00845001652	43	54	69	47
ENSCAFG00845001653	9619	9303	9892	9597

ENSCAFG00845001650	0	0	0	0
ENSCAFG00845001651	692	675	686	642
ENSCAFG00845025600	1	1	0	0
ENSCAFG00845013627	169	143	155	168
ENSCAFG00845013626	370	324	342	356
ENSCAFG00845013629	5	3	2	7
ENSCAFG00845013628	2153	2109	2096	1988
ENSCAFG00845013623	0	0	0	0
ENSCAFG00845013622	0	0	2	2
ENSCAFG00845013625	497	507	579	558
ENSCAFG00845013624	1420	1303	1334	1369
ENSCAFG00845013621	2	0	0	1
ENSCAFG00845013620	178	175	177	189
ENSCAFG00845001629	477	439	392	366
ENSCAFG00845001627	316	339	352	276
ENSCAFG00845001628	1236	1211	1056	1067
ENSCAFG00845001625	495	372	457	535
ENSCAFG00845001626	487	467	446	488
ENSCAFG00845001623	0	0	1	0
ENSCAFG00845001624	4572	4445	4521	4558
ENSCAFG00845001621	0	0	0	0
ENSCAFG00845001622	1743	1830	1819	1941
ENSCAFG00845001620	215	274	169	208
ENSCAFG00845013619	214	199	225	234
ENSCAFG00845013616	0	1	0	0
ENSCAFG00845013615	0	0	0	0
ENSCAFG00845013618	8	8	9	11
ENSCAFG00845013617	1256	1197	1209	1151
ENSCAFG00845013612	0	0	0	0
ENSCAFG00845013611	0	0	0	0
ENSCAFG00845013614	0	0	0	0
ENSCAFG00845013613	26	40	26	36
ENSCAFG00845013610	2	1	0	1
ENSCAFG00845001638	3	3	5	0
ENSCAFG00845001639	2368	2083	2392	2529
ENSCAFG00845001636	680	705	825	849
ENSCAFG00845001637	558	509	504	607
ENSCAFG00845001634	18	21	13	32
ENSCAFG00845001635	376	407	333	328
ENSCAFG00845001632	0	0	0	3
ENSCAFG00845001633	1636	1458	828	787

ENSCAFG00845001630	546	584	655	667
ENSCAFG00845001631	1	6	12	4
ENSCAFG00845013609	0	0	0	0
ENSCAFG00845013608	836	824	956	988
ENSCAFG00845013605	0	0	0	0
ENSCAFG00845013604	1282	1257	1143	1288
ENSCAFG00845013607	1468	1510	1374	1314
ENSCAFG00845013606	0	0	0	0
ENSCAFG00845013601	39	34	41	53
ENSCAFG00845013600	0	0	0	0
ENSCAFG00845013603	1337	1411	1390	1432
ENSCAFG00845013602	337	347	309	319
ENSCAFG00845011095	13	12	20	15
ENSCAFG00845011096	240	238	240	244
ENSCAFG00845011097	219	206	254	251
ENSCAFG00845011098	0	0	0	0
ENSCAFG00845011091	2	0	0	0
ENSCAFG00845011092	0	0	0	0
ENSCAFG00845011093	0	1	0	1
ENSCAFG00845011094	0	1	2	0
ENSCAFG00845023079	2457	2531	2544	2567
ENSCAFG00845011090	492	454	491	482
ENSCAFG00845023078	0	10	4	3
ENSCAFG00845023073	376	425	381	387
ENSCAFG00845023072	7	9	5	8
ENSCAFG00845023071	422	403	418	400
ENSCAFG00845023070	11	16	5	9
ENSCAFG00845023077	0	1	0	0
ENSCAFG00845023076	925	984	894	934
ENSCAFG00845023075	0	0	0	0
ENSCAFG00845023074	0	0	0	0
ENSCAFG00845011099	5024	4889	4622	4535
ENSCAFG00845011084	0	0	0	0
ENSCAFG00845011085	271	228	256	311
ENSCAFG00845011086	0	0	0	0
ENSCAFG00845011087	3931	3738	4163	4002
ENSCAFG00845011080	1094	1059	1210	1216
ENSCAFG00845011081	0	0	0	0
ENSCAFG00845011082	0	0	0	0
ENSCAFG00845011083	40	60	69	48
ENSCAFG00845023069	1	1	1	0

ENSCAFG00845023068	0	0	0	0
ENSCAFG00845023067	374	356	387	409
ENSCAFG00845023062	162	163	183	170
ENSCAFG00845023061	25	35	49	37
ENSCAFG00845023060	2	0	3	0
ENSCAFG00845023066	625	581	611	606
ENSCAFG00845023064	0	0	0	0
ENSCAFG00845023063	1	1	0	0
ENSCAFG00845011088	358	363	397	365
ENSCAFG00845011089	58	25	47	62
ENSCAFG00845011073	0	0	0	1
ENSCAFG00845011074	0	0	0	0
ENSCAFG00845011075	0	3	0	0
ENSCAFG00845011076	0	0	0	0
ENSCAFG00845011070	0	0	0	0
ENSCAFG00845011071	8535	8149	5264	5147
ENSCAFG00845011072	100	108	105	114
ENSCAFG00845023059	72	59	88	66
ENSCAFG00845023058	0	0	0	0
ENSCAFG00845023057	0	0	0	0
ENSCAFG00845023056	1135	1190	1256	1173
ENSCAFG00845023051	0	0	0	0
ENSCAFG00845023050	461	457	400	405
ENSCAFG00845023055	672	668	691	674
ENSCAFG00845023054	0	0	1	0
ENSCAFG00845023053	5323	5225	5412	5165
ENSCAFG00845023052	0	0	2	0
ENSCAFG00845011077	2	0	0	1
ENSCAFG00845011078	0	0	0	0
ENSCAFG00845011079	129	109	111	131
ENSCAFG00845011062	0	0	0	0
ENSCAFG00845011063	2	0	3	5
ENSCAFG00845011064	1	1	4	3
ENSCAFG00845011065	15	7	2	4
ENSCAFG00845011060	2	1	4	3
ENSCAFG00845011061	500	494	488	532
ENSCAFG00845023048	283	245	255	195
ENSCAFG00845023047	696	633	531	515
ENSCAFG00845023046	0	0	3	1
ENSCAFG00845023049	3	1	1	1
ENSCAFG00845023044	2	3	0	1

ENSCAFG00845023043	562	492	468	480
ENSCAFG00845023042	0	0	0	0
ENSCAFG00845023041	0	0	0	0
ENSCAFG00845011066	2575	2560	2590	2552
ENSCAFG00845011067	437	345	372	356
ENSCAFG00845011068	187	176	139	140
ENSCAFG00845011069	0	0	0	0
ENSCAFG00845011051	1231	1270	1287	1278
ENSCAFG00845011052	733	659	686	695
ENSCAFG00845011053	47	47	43	43
ENSCAFG00845011054	0	0	0	0
ENSCAFG00845011050	5	5	1	0
ENSCAFG00845023036	273	210	244	223
ENSCAFG00845023035	0	2	0	0
ENSCAFG00845023034	326	357	383	387
ENSCAFG00845025696	0	0	0	0
ENSCAFG00845025697	443	524	533	535
ENSCAFG00845023039	0	0	0	0
ENSCAFG00845025698	3053	2832	2881	2965
ENSCAFG00845023038	1654	1551	1651	1557
ENSCAFG00845025699	7	1	4	10
ENSCAFG00845025692	251	293	300	264
ENSCAFG00845025693	685	690	728	730
ENSCAFG00845025694	0	0	0	0
ENSCAFG00845025695	0	0	0	0
ENSCAFG00845023033	44	38	42	34
ENSCAFG00845023032	0	0	0	0
ENSCAFG00845023031	0	0	1	1
ENSCAFG00845025690	0	0	0	1
ENSCAFG00845023030	493	462	509	491
ENSCAFG00845025691	9	7	6	14
ENSCAFG00845011059	0	0	0	0
ENSCAFG00845011055	818	818	720	770
ENSCAFG00845011056	4	5	2	0
ENSCAFG00845011057	0	0	0	0
ENSCAFG00845011058	5121	5136	2644	3017
ENSCAFG00845011040	0	0	0	0
ENSCAFG00845011041	2	1	1	0
ENSCAFG00845011042	0	0	0	0
ENSCAFG00845011043	66	41	53	56
ENSCAFG00845023026	1	1	0	0

ENSCAFG00845025689	0	0	0	0
ENSCAFG00845023025	0	0	0	0
ENSCAFG00845023024	53	35	51	42
ENSCAFG00845023023	2754	2669	2635	2704
ENSCAFG00845025685	0	0	0	1
ENSCAFG00845023029	0	0	0	0
ENSCAFG00845025686	4	0	0	0
ENSCAFG00845023028	0	0	0	0
ENSCAFG00845023027	1450	1379	1356	1377
ENSCAFG00845025688	0	0	0	0
ENSCAFG00845025681	1063	1018	989	1001
ENSCAFG00845025682	0	0	0	0
ENSCAFG00845025683	124	109	167	136
ENSCAFG00845025684	40	18	28	26
ENSCAFG00845023022	0	0	0	0
ENSCAFG00845023021	17	20	24	19
ENSCAFG00845025680	489	500	499	543
ENSCAFG00845011048	19	15	18	23
ENSCAFG00845011049	2	2	3	2
ENSCAFG00845011044	6	0	7	1
ENSCAFG00845011045	0	0	1	0
ENSCAFG00845011046	362	382	328	331
ENSCAFG00845011047	93	99	78	102
ENSCAFG00845013692	25	17	26	30
ENSCAFG00845011030	4	1	5	0
ENSCAFG00845013691	133	93	75	114
ENSCAFG00845011031	3886	3584	3935	3959
ENSCAFG00845013694	39	36	53	34
ENSCAFG00845011032	3	7	3	1
ENSCAFG00845013693	1	0	0	0
ENSCAFG00845013690	631	688	714	763
ENSCAFG00845023015	0	0	0	2
ENSCAFG00845025678	0	0	0	0
ENSCAFG00845023014	192	185	203	145
ENSCAFG00845025679	2	0	0	0
ENSCAFG00845023013	2728	2482	2690	2809
ENSCAFG00845023012	32	19	26	23
ENSCAFG00845023019	455	430	463	462
ENSCAFG00845025674	0	0	0	0
ENSCAFG00845023018	1	0	0	0
ENSCAFG00845025675	66	62	56	43

ENSCAFG00845023017	0	0	0	0
ENSCAFG00845025676	103	96	85	70
ENSCAFG00845023016	2451	2332	2411	2367
ENSCAFG00845025677	0	0	0	0
ENSCAFG00845025670	10	11	9	7
ENSCAFG00845025671	451	388	375	387
ENSCAFG00845025672	10	16	9	16
ENSCAFG00845025673	0	0	0	0
ENSCAFG00845023011	0	0	0	0
ENSCAFG00845023010	2	0	3	2
ENSCAFG00845011037	1	0	4	0
ENSCAFG00845011038	954	919	1018	1107
ENSCAFG00845013699	0	0	0	0
ENSCAFG00845011039	591	619	584	600
ENSCAFG00845011033	3	4	1	10
ENSCAFG00845013696	372	323	400	484
ENSCAFG00845011034	21	14	31	28
ENSCAFG00845013695	0	0	0	0
ENSCAFG00845011035	0	0	0	0
ENSCAFG00845013698	0	0	1	0
ENSCAFG00845011036	1631	1565	1336	1420
ENSCAFG00845013697	923	812	901	866
ENSCAFG00845013681	100	131	95	100
ENSCAFG00845013680	0	0	0	0
ENSCAFG00845011020	1	0	0	0
ENSCAFG00845013683	0	0	0	2
ENSCAFG00845011021	0	5	2	0
ENSCAFG00845013682	1327	1273	1370	1390
ENSCAFG00845023009	359	273	298	271
ENSCAFG00845023004	0	0	0	0
ENSCAFG00845025667	0	0	0	0
ENSCAFG00845023003	784	783	644	642
ENSCAFG00845025668	2	4	2	2
ENSCAFG00845023002	0	0	0	0
ENSCAFG00845025669	35	21	25	19
ENSCAFG00845023001	0	0	0	0
ENSCAFG00845023008	1085	1086	1211	1149
ENSCAFG00845025663	14	9	9	11
ENSCAFG00845023007	3	0	4	0
ENSCAFG00845025664	2117	2149	1834	1884
ENSCAFG00845023006	2	0	0	0

ENSCAFG00845025665	484	491	470	456
ENSCAFG00845023005	2603	2557	2176	2225
ENSCAFG00845025666	3	1	1	4
ENSCAFG00845025660	1878	1830	1741	1738
ENSCAFG00845025661	0	0	0	0
ENSCAFG00845025662	0	0	0	0
ENSCAFG00845023000	1651	1522	1503	1568
ENSCAFG00845011026	1	0	0	0
ENSCAFG00845013689	3127	3104	2588	2508
ENSCAFG00845011027	2412	2345	2584	2720
ENSCAFG00845013688	0	0	2	1
ENSCAFG00845011028	377	407	385	404
ENSCAFG00845011029	474	457	451	503
ENSCAFG00845011022	578	558	584	567
ENSCAFG00845013685	5	7	12	1
ENSCAFG00845011023	4	1	3	1
ENSCAFG00845013684	823	833	712	737
ENSCAFG00845011024	66	82	64	62
ENSCAFG00845013687	4	0	1	9
ENSCAFG00845011025	30	46	28	40
ENSCAFG00845013686	0	0	0	0
ENSCAFG00845027599	156	138	176	153
ENSCAFG00845027596	0	2	2	4
ENSCAFG00845027595	14	7	16	7
ENSCAFG00845027597	912	881	866	813
ENSCAFG00845027592	1156	979	1036	1056
ENSCAFG00845027591	0	0	0	0
ENSCAFG00845027594	0	0	0	0
ENSCAFG00845027590	1	0	1	1
ENSCAFG00845027589	0	0	0	0
ENSCAFG00845027588	21	12	21	20
ENSCAFG00845027585	2	0	0	1
ENSCAFG00845027584	12	14	8	12
ENSCAFG00845027587	1	1	1	0
ENSCAFG00845027586	0	0	1	1
ENSCAFG00845027581	0	0	0	0
ENSCAFG00845027580	13	6	5	12
ENSCAFG00845027583	0	0	0	0
ENSCAFG00845027582	2068	2036	2178	2292
ENSCAFG00845015596	0	0	0	0
ENSCAFG00845015597	0	0	1	1

ENSCAFG00845015594	0	0	0	0
ENSCAFG00845015595	856	865	955	936
ENSCAFG00845015592	0	0	0	0
ENSCAFG00845015593	0	0	0	0
ENSCAFG00845015590	3680	3839	3688	3606
ENSCAFG00845015591	0	3	0	1
ENSCAFG00845027578	0	0	0	0
ENSCAFG00845027577	0	0	0	0
ENSCAFG00845027579	5	7	5	4
ENSCAFG00845027574	5	3	4	1
ENSCAFG00845027573	0	0	0	0
ENSCAFG00845027576	761	627	620	583
ENSCAFG00845027575	1	0	1	0
ENSCAFG00845027570	0	0	0	0
ENSCAFG00845027572	0	0	0	0
ENSCAFG00845027571	289	269	237	240
ENSCAFG00845015598	0	0	0	0
ENSCAFG00845015599	204	194	137	149
ENSCAFG00845015585	359	375	396	424
ENSCAFG00845015586	0	0	0	0
ENSCAFG00845015583	0	0	0	0
ENSCAFG00845015584	101	109	135	154
ENSCAFG00845015581	6	6	2	3
ENSCAFG00845015582	76	57	47	56
ENSCAFG00845015580	0	0	0	0
ENSCAFG00845027567	1370	1328	1201	1218
ENSCAFG00845027566	2	0	2	0
ENSCAFG00845027569	2196	2087	2136	2166
ENSCAFG00845027568	3	3	7	4
ENSCAFG00845027563	0	0	0	0
ENSCAFG00845027562	0	0	0	0
ENSCAFG00845027565	0	0	2	0
ENSCAFG00845027564	202	205	238	226
ENSCAFG00845027561	1	0	2	0
ENSCAFG00845027560	141	126	143	144
ENSCAFG00845015589	2	0	0	0
ENSCAFG00845015587	495	457	484	488
ENSCAFG00845015588	0	0	0	0
ENSCAFG00845003509	0	0	0	0
ENSCAFG00845003508	1	2	3	0
ENSCAFG00845003507	109	128	119	112

ENSCAFG00845003506	239	187	234	246
ENSCAFG00845003505	0	0	0	0
ENSCAFG00845003504	1	3	0	2
ENSCAFG00845003503	477	418	489	494
ENSCAFG00845003502	0	0	0	0
ENSCAFG00845003501	10438	10199	9443	9580
ENSCAFG00845003500	0	0	0	0
ENSCAFG00845003519	2085	2076	2160	2188
ENSCAFG00845003518	17	9	4	6
ENSCAFG00845003517	0	0	0	0
ENSCAFG00845003516	13272	12812	13554	13314
ENSCAFG00845003515	1101	1081	1071	1085
ENSCAFG00845003514	1562	1526	1440	1389
ENSCAFG00845003513	945	893	990	928
ENSCAFG00845003512	236	238	121	155
ENSCAFG00845003511	817	876	829	891
ENSCAFG00845003510	0	0	0	0
ENSCAFG00845015574	1397	1343	891	961
ENSCAFG00845015575	0	0	0	0
ENSCAFG00845015572	645	641	605	681
ENSCAFG00845015573	506	513	404	405
ENSCAFG00845015570	0	0	0	0
ENSCAFG00845015571	0	0	0	0
ENSCAFG00845027559	0	1	0	0
ENSCAFG00845003589	640	676	528	655
ENSCAFG00845027556	1	2	2	2
ENSCAFG00845003588	14	8	11	0
ENSCAFG00845027555	0	0	0	0
ENSCAFG00845003587	2	0	0	2
ENSCAFG00845027558	0	0	0	0
ENSCAFG00845003586	6738	6690	6839	6514
ENSCAFG00845027557	571	550	582	563
ENSCAFG00845003585	0	4	0	0
ENSCAFG00845027552	14	12	22	12
ENSCAFG00845003584	0	0	1	1
ENSCAFG00845027551	1	0	0	0
ENSCAFG00845003583	1616	1644	1494	1548
ENSCAFG00845027554	17	6	22	17
ENSCAFG00845003582	1170	1158	1088	1217
ENSCAFG00845027553	0	0	4	2
ENSCAFG00845003581	0	1	2	2

ENSCAFG00845003580	0	0	0	0
ENSCAFG00845027550	1	0	2	0
ENSCAFG00845015578	477	543	560	565
ENSCAFG00845015579	27	26	31	24
ENSCAFG00845015576	206	201	182	220
ENSCAFG00845015577	1176	1037	1175	1253
ENSCAFG00845015563	238	211	234	182
ENSCAFG00845015564	174	167	151	151
ENSCAFG00845015561	0	0	0	0
ENSCAFG00845015562	0	0	0	0
ENSCAFG00845015560	0	0	0	0
ENSCAFG00845027549	0	0	0	0
ENSCAFG00845027548	1139	1098	1154	1196
ENSCAFG00845027545	3	2	9	6
ENSCAFG00845003599	4	3	2	2
ENSCAFG00845027544	0	0	0	0
ENSCAFG00845003598	0	0	2	2
ENSCAFG00845003597	268	255	281	327
ENSCAFG00845027546	6	1	6	2
ENSCAFG00845003596	29	21	11	20
ENSCAFG00845027541	0	0	0	0
ENSCAFG00845003595	1429	1401	1188	1209
ENSCAFG00845027540	0	0	0	1
ENSCAFG00845003594	0	0	0	0
ENSCAFG00845027543	0	0	0	0
ENSCAFG00845003593	100	99	78	74
ENSCAFG00845027542	0	0	1	1
ENSCAFG00845003592	753	772	708	821
ENSCAFG00845003591	0	0	0	0
ENSCAFG00845003590	29	27	32	37
ENSCAFG00845015569	3	2	2	2
ENSCAFG00845015567	0	0	0	0
ENSCAFG00845015568	28	37	13	26
ENSCAFG00845015565	0	0	0	0
ENSCAFG00845015566	188	180	186	167
ENSCAFG00845015552	3	10	4	3
ENSCAFG00845015553	238	227	249	308
ENSCAFG00845015550	5963	5766	5185	5900
ENSCAFG00845015551	0	3	0	2
ENSCAFG00845027538	981	832	1004	1031
ENSCAFG00845027537	1	1	0	2

ENSCAFG00845003569	5	7	3	6
ENSCAFG00845003568	1212	1282	1082	1179
ENSCAFG00845027539	1315	1321	1031	1008
ENSCAFG00845003567	0	0	0	0
ENSCAFG00845027534	0	0	1	0
ENSCAFG00845003566	0	0	0	0
ENSCAFG00845027533	0	0	0	0
ENSCAFG00845003565	914	891	864	887
ENSCAFG00845027536	0	0	0	0
ENSCAFG00845003564	708	627	720	716
ENSCAFG00845027535	0	1	0	0
ENSCAFG00845003563	7	8	10	10
ENSCAFG00845027530	1013	1083	1135	1106
ENSCAFG00845003562	175	160	191	230
ENSCAFG00845003561	80	81	70	55
ENSCAFG00845027532	0	0	0	0
ENSCAFG00845003560	9	6	8	2
ENSCAFG00845027531	817	740	728	673
ENSCAFG00845015558	61	73	58	55
ENSCAFG00845015559	0	0	0	0
ENSCAFG00845015556	0	0	0	0
ENSCAFG00845015557	4906	4557	4816	5008
ENSCAFG00845015554	0	0	0	0
ENSCAFG00845015555	0	2	1	2
ENSCAFG00845015541	4	2	19	5
ENSCAFG00845015542	0	0	0	0
ENSCAFG00845015540	1581	1376	1520	1362
ENSCAFG00845027527	741	699	736	848
ENSCAFG00845027526	8742	8381	8294	8351
ENSCAFG00845003579	0	0	0	0
ENSCAFG00845027528	1	0	0	0
ENSCAFG00845003578	407	384	390	423
ENSCAFG00845027523	12	3	3	5
ENSCAFG00845003577	0	0	0	0
ENSCAFG00845027522	733	699	759	749
ENSCAFG00845003576	87	97	77	100
ENSCAFG00845027525	1504	1453	1455	1435
ENSCAFG00845003575	0	0	0	0
ENSCAFG00845027524	625	602	646	703
ENSCAFG00845003574	0	0	0	0
ENSCAFG00845003573	264	233	167	203

ENSCAFG00845003572	51	36	49	40
ENSCAFG00845027521	0	0	0	0
ENSCAFG00845003571	15	9	8	8
ENSCAFG00845027520	1	2	1	1
ENSCAFG00845003570	0	1	0	0
ENSCAFG00845015549	0	0	0	0
ENSCAFG00845015547	0	4	0	2
ENSCAFG00845015548	6	2	4	6
ENSCAFG00845015545	917	954	954	906
ENSCAFG00845015546	242	237	214	222
ENSCAFG00845015543	7475	7238	6292	6294
ENSCAFG00845015544	0	0	0	0
ENSCAFG00845015530	77	51	63	68
ENSCAFG00845015531	0	0	0	0
ENSCAFG00845027519	0	0	0	0
ENSCAFG00845003549	761	788	837	823
ENSCAFG00845027516	6	9	2	5
ENSCAFG00845003548	492	464	430	447
ENSCAFG00845027515	0	0	0	0
ENSCAFG00845003547	0	0	0	0
ENSCAFG00845027518	927	819	938	959
ENSCAFG00845003546	6	7	9	5
ENSCAFG00845027517	80	70	113	129
ENSCAFG00845003545	86	76	62	60
ENSCAFG00845027512	0	0	0	0
ENSCAFG00845003544	817	851	828	943
ENSCAFG00845027511	9	1	3	4
ENSCAFG00845003543	3	14	3	12
ENSCAFG00845027514	187	190	188	189
ENSCAFG00845003542	6089	6070	6377	6396
ENSCAFG00845027513	21	8	9	14
ENSCAFG00845003541	2982	2961	2791	2815
ENSCAFG00845003540	882	817	905	875
ENSCAFG00845027510	0	3	2	0
ENSCAFG00845015538	0	0	0	0
ENSCAFG00845015539	531	558	581	522
ENSCAFG00845015536	69	74	76	92
ENSCAFG00845015537	0	0	0	0
ENSCAFG00845015534	2367	2258	2518	2590
ENSCAFG00845015535	2	4	2	1
ENSCAFG00845015532	32	17	22	25

ENSCAFG00845015533	102	90	90	85
ENSCAFG00845015520	3704	3401	3538	3755
ENSCAFG00845027509	0	0	0	0
ENSCAFG00845027508	2727	2631	2566	2503
ENSCAFG00845027505	0	0	0	0
ENSCAFG00845003559	405	434	333	399
ENSCAFG00845027504	2298	2311	2149	2260
ENSCAFG00845003558	539	560	518	524
ENSCAFG00845027507	0	0	0	0
ENSCAFG00845003557	0	0	0	0
ENSCAFG00845027506	842	776	778	777
ENSCAFG00845003556	432	377	389	417
ENSCAFG00845027501	1	0	0	0
ENSCAFG00845003555	98	105	56	67
ENSCAFG00845027500	14	30	20	21
ENSCAFG00845003554	455	463	466	472
ENSCAFG00845027503	3	2	10	8
ENSCAFG00845003553	505	442	554	531
ENSCAFG00845003552	516	556	634	631
ENSCAFG00845003551	822	775	607	582
ENSCAFG00845003550	0	0	0	0
ENSCAFG00845015529	22	21	14	6
ENSCAFG00845015527	476	484	585	649
ENSCAFG00845015528	469	490	454	454
ENSCAFG00845015525	0	0	0	0
ENSCAFG00845015526	5	0	0	0
ENSCAFG00845015523	0	0	0	0
ENSCAFG00845015524	0	0	0	2
ENSCAFG00845015521	1550	1570	1450	1551
ENSCAFG00845015522	34	49	37	28
ENSCAFG00845003529	885	853	930	974
ENSCAFG00845003528	0	0	0	0
ENSCAFG00845003527	987	971	667	654
ENSCAFG00845003526	337	359	344	304
ENSCAFG00845003525	1466	1417	1741	1653
ENSCAFG00845003524	0	0	0	0
ENSCAFG00845003523	6355	6073	5982	6281
ENSCAFG00845003522	3	4	9	1
ENSCAFG00845003521	1	1	0	1
ENSCAFG00845003520	960	992	824	809
ENSCAFG00845015518	298	320	263	357

ENSCAFG00845015519	0	0	0	0
ENSCAFG00845015516	433	497	523	568
ENSCAFG00845015517	0	0	0	0
ENSCAFG00845015514	0	0	0	0
ENSCAFG00845015515	0	0	0	0
ENSCAFG00845015512	265	272	199	286
ENSCAFG00845015513	0	0	0	0
ENSCAFG00845015510	125	102	80	63
ENSCAFG00845015511	0	0	0	0
ENSCAFG00845003539	0	0	0	0
ENSCAFG00845003538	0	0	1	5
ENSCAFG00845003537	853	793	737	756
ENSCAFG00845003536	29	11	27	27
ENSCAFG00845003535	36	30	46	27
ENSCAFG00845003534	2840	2628	2809	2956
ENSCAFG00845003533	1773	1763	1782	1655
ENSCAFG00845003532	1	0	0	0
ENSCAFG00845003531	0	0	0	0
ENSCAFG00845003530	430	400	440	376
ENSCAFG00845015509	2	1	0	0
ENSCAFG00845015507	255	230	310	332
ENSCAFG00845015508	1206	1177	1205	1296
ENSCAFG00845015505	1319	1235	1189	1162
ENSCAFG00845015506	90	65	62	64
ENSCAFG00845015503	0	0	0	0
ENSCAFG00845015504	598	571	581	526
ENSCAFG00845015501	0	0	0	0
ENSCAFG00845015502	0	0	0	0
ENSCAFG00845015500	7	13	12	12
ENSCAFG00845025098	12	4	4	3
ENSCAFG00845025099	2	0	0	2
ENSCAFG00845025094	10	7	3	9
ENSCAFG00845025095	2	0	0	0
ENSCAFG00845025096	1515	1427	1485	1507
ENSCAFG00845025097	0	0	0	0
ENSCAFG00845025090	2	6	4	8
ENSCAFG00845025091	0	0	0	0
ENSCAFG00845025092	90	84	73	65
ENSCAFG00845025093	1458	1476	1241	1278
ENSCAFG00845025087	2	2	0	1
ENSCAFG00845025088	175	188	181	180

ENSCAFG00845025089	0	0	0	0
ENSCAFG00845025083	0	0	0	0
ENSCAFG00845025084	1507	1417	1480	1460
ENSCAFG00845025085	6	5	7	4
ENSCAFG00845025080	30	44	23	24
ENSCAFG00845025081	26	30	27	24
ENSCAFG00845025082	10	10	1	5
ENSCAFG00845013098	9	15	6	20
ENSCAFG00845013097	748	846	930	920
ENSCAFG00845013099	4342	4169	3170	3157
ENSCAFG00845013094	1776	1735	1809	1780
ENSCAFG00845013093	0	0	0	2
ENSCAFG00845013096	1119	1084	1077	1119
ENSCAFG00845013095	92	77	90	119
ENSCAFG00845013090	314	392	374	367
ENSCAFG00845013092	455	406	514	490
ENSCAFG00845013091	1	1	0	0
ENSCAFG00845025076	422	430	400	364
ENSCAFG00845025077	3831	3607	3709	3552
ENSCAFG00845025078	863	946	971	919
ENSCAFG00845025072	1405	1349	1357	1319
ENSCAFG00845025073	167	177	150	178
ENSCAFG00845025074	0	0	0	0
ENSCAFG00845025075	0	0	0	0
ENSCAFG00845025070	57	50	52	58
ENSCAFG00845025071	1	0	0	1
ENSCAFG00845013087	0	0	0	0
ENSCAFG00845013086	2	1	3	3
ENSCAFG00845013089	5935	5886	5799	6000
ENSCAFG00845013088	3	4	8	5
ENSCAFG00845013083	544	561	467	463
ENSCAFG00845013082	2812	2861	3298	3443
ENSCAFG00845013085	0	0	1	0
ENSCAFG00845013084	864	813	744	836
ENSCAFG00845013081	8	12	8	3
ENSCAFG00845013080	0	0	0	0
ENSCAFG00845025069	0	0	0	0
ENSCAFG00845025065	0	0	0	0
ENSCAFG00845025066	70	78	62	45
ENSCAFG00845025067	0	0	0	0
ENSCAFG00845025068	0	0	1	0

ENSCAFG00845025061	2	3	2	0
ENSCAFG00845025062	400	393	322	346
ENSCAFG00845025063	0	0	0	0
ENSCAFG00845025064	0	0	0	0
ENSCAFG00845025060	2	0	0	2
ENSCAFG00845013076	3787	3695	3530	3394
ENSCAFG00845013075	50	59	98	99
ENSCAFG00845013078	1536	1409	1285	1340
ENSCAFG00845013077	1	1	0	3
ENSCAFG00845013072	91	71	66	55
ENSCAFG00845013071	0	0	0	0
ENSCAFG00845013074	0	0	0	0
ENSCAFG00845013073	21	16	15	20
ENSCAFG00845013070	0	0	1	0
ENSCAFG00845025059	5	7	2	0
ENSCAFG00845001089	0	0	0	0
ENSCAFG00845001087	577	487	653	635
ENSCAFG00845025054	128	136	110	114
ENSCAFG00845001088	0	0	0	0
ENSCAFG00845025055	0	0	0	0
ENSCAFG00845001085	804	748	728	736
ENSCAFG00845025056	0	5	3	3
ENSCAFG00845001086	0	0	0	0
ENSCAFG00845025057	2863	2922	2732	2776
ENSCAFG00845001083	0	0	0	0
ENSCAFG00845025050	194	216	132	150
ENSCAFG00845001084	0	0	0	0
ENSCAFG00845025051	1	1	0	0
ENSCAFG00845001081	1486	1519	1407	1305
ENSCAFG00845025052	0	0	0	0
ENSCAFG00845001082	0	0	0	0
ENSCAFG00845025053	0	0	0	0
ENSCAFG00845001080	1080	1036	992	1053
ENSCAFG00845013079	0	0	1	0
ENSCAFG00845013065	157	176	216	195
ENSCAFG00845013064	1921	1966	1775	1781
ENSCAFG00845013067	0	0	0	0
ENSCAFG00845013066	87	81	76	85
ENSCAFG00845013061	0	0	0	0
ENSCAFG00845013060	1321	1264	1171	1134
ENSCAFG00845013063	0	0	0	0

ENSCAFG00845013062	389	398	311	408
ENSCAFG00845025047	18	12	8	14
ENSCAFG00845025048	1	0	2	2
ENSCAFG00845025049	0	0	0	0
ENSCAFG00845001098	6	4	4	0
ENSCAFG00845025043	0	0	0	0
ENSCAFG00845001099	0	0	0	0
ENSCAFG00845025044	1	1	4	4
ENSCAFG00845001096	1775	1625	1653	1841
ENSCAFG00845025045	848	730	847	838
ENSCAFG00845001097	0	0	0	0
ENSCAFG00845025046	161	146	132	118
ENSCAFG00845001094	16	6	17	14
ENSCAFG00845001095	0	2	0	4
ENSCAFG00845025040	1295	1369	1251	1229
ENSCAFG00845001092	0	0	0	0
ENSCAFG00845025041	0	0	0	0
ENSCAFG00845001093	168	198	192	175
ENSCAFG00845025042	41	33	54	24
ENSCAFG00845001090	0	0	0	0
ENSCAFG00845001091	0	0	0	0
ENSCAFG00845013069	0	0	0	0
ENSCAFG00845013068	0	0	0	0
ENSCAFG00845013054	0	0	0	0
ENSCAFG00845013053	2	0	0	0
ENSCAFG00845013056	182	156	170	199
ENSCAFG00845013055	1	3	0	3
ENSCAFG00845013050	0	0	0	0
ENSCAFG00845013052	774	733	611	666
ENSCAFG00845013051	0	0	0	0
ENSCAFG00845001069	0	0	0	0
ENSCAFG00845025036	0	0	0	0
ENSCAFG00845025037	7	0	6	3
ENSCAFG00845027698	0	0	0	0
ENSCAFG00845001067	0	0	0	0
ENSCAFG00845025038	193	194	174	182
ENSCAFG00845001068	86	89	89	98
ENSCAFG00845025039	0	0	0	0
ENSCAFG00845001065	976	858	867	876
ENSCAFG00845025032	5	0	0	0
ENSCAFG00845027695	8	1	3	5

ENSCAFG00845001066	3	2	1	1
ENSCAFG00845027694	2629	2538	2234	2258
ENSCAFG00845001063	960	920	983	1019
ENSCAFG00845025034	244	253	195	216
ENSCAFG00845027697	0	0	0	0
ENSCAFG00845001064	370	357	361	342
ENSCAFG00845025035	2	0	0	0
ENSCAFG00845027696	3	2	2	1
ENSCAFG00845001061	0	0	0	0
ENSCAFG00845027691	1	12	2	2
ENSCAFG00845001062	1467	1527	1178	1188
ENSCAFG00845027690	3	0	0	0
ENSCAFG00845025030	0	0	0	0
ENSCAFG00845027693	0	0	0	0
ENSCAFG00845001060	1	1	2	2
ENSCAFG00845025031	2523	2353	2261	2330
ENSCAFG00845027692	0	0	0	0
ENSCAFG00845013058	541	532	551	548
ENSCAFG00845013057	0	0	0	0
ENSCAFG00845013059	430	417	404	410
ENSCAFG00845013043	19	13	10	5
ENSCAFG00845013042	2481	2381	2267	2176
ENSCAFG00845013045	0	0	0	0
ENSCAFG00845013044	356	347	350	376
ENSCAFG00845013041	20	31	32	39
ENSCAFG00845013040	0	0	0	0
ENSCAFG00845025029	0	0	0	0
ENSCAFG00845025025	0	0	0	0
ENSCAFG00845027688	0	0	0	0
ENSCAFG00845025026	757	756	750	880
ENSCAFG00845027687	9	2	2	5
ENSCAFG00845001078	852	845	902	915
ENSCAFG00845025027	145	153	165	158
ENSCAFG00845001079	0	0	0	0
ENSCAFG00845025028	0	0	0	0
ENSCAFG00845027689	0	0	0	0
ENSCAFG00845001076	0	0	0	0
ENSCAFG00845025021	0	0	0	0
ENSCAFG00845027684	0	0	0	0
ENSCAFG00845001077	149	186	93	87
ENSCAFG00845025022	0	0	0	0

ENSCAFG00845027683	0	0	0	0
ENSCAFG00845001074	13	12	9	13
ENSCAFG00845025023	0	0	0	0
ENSCAFG00845027686	8	9	8	2
ENSCAFG00845001075	3	0	4	3
ENSCAFG00845025024	604	534	498	556
ENSCAFG00845027685	0	0	0	0
ENSCAFG00845001072	295	296	381	347
ENSCAFG00845027680	1167	1242	1377	1491
ENSCAFG00845001073	0	0	0	0
ENSCAFG00845001070	8	11	4	2
ENSCAFG00845027682	0	0	0	0
ENSCAFG00845001071	0	0	0	0
ENSCAFG00845027681	3	1	8	7
ENSCAFG00845013047	0	0	0	0
ENSCAFG00845013046	0	0	0	0
ENSCAFG00845013049	0	0	0	0
ENSCAFG00845013048	719	629	704	650
ENSCAFG00845003629	5	1	0	2
ENSCAFG00845003628	0	0	0	0
ENSCAFG00845003627	1	5	12	6
ENSCAFG00845003626	2713	2632	2187	2202
ENSCAFG00845003625	0	0	0	0
ENSCAFG00845003624	36	35	60	55
ENSCAFG00845003623	1410	1355	1327	1399
ENSCAFG00845003622	0	1	0	0
ENSCAFG00845003621	115	127	120	147
ENSCAFG00845003620	0	0	0	0
ENSCAFG00845015619	598	538	503	462
ENSCAFG00845015617	0	0	0	0
ENSCAFG00845015618	0	0	0	0
ENSCAFG00845015615	0	0	0	0
ENSCAFG00845015616	8	7	9	14
ENSCAFG00845015613	1	2	0	1
ENSCAFG00845015614	0	0	0	0
ENSCAFG00845015611	5	0	0	0
ENSCAFG00845015612	365	363	60	52
ENSCAFG00845015610	0	3	0	0
ENSCAFG00845003639	2	9	0	3
ENSCAFG00845003638	0	0	0	0
ENSCAFG00845003637	574	520	533	578

ENSCAFG00845003636	0	0	0	0
ENSCAFG00845003635	0	2	0	0
ENSCAFG00845003634	0	0	0	0
ENSCAFG00845003633	0	3	0	0
ENSCAFG00845003632	1099	1071	997	976
ENSCAFG00845003631	406	347	380	362
ENSCAFG00845003630	79	59	136	111
ENSCAFG00845015608	8	11	5	5
ENSCAFG00845015609	10146	9991	10668	10421
ENSCAFG00845015606	0	0	0	0
ENSCAFG00845015607	2873	2742	2589	2618
ENSCAFG00845015604	1217	1058	1168	1134
ENSCAFG00845015605	1683	1665	1845	1949
ENSCAFG00845015602	0	0	0	0
ENSCAFG00845015603	66	60	65	63
ENSCAFG00845015600	131	83	140	160
ENSCAFG00845015601	206	203	191	193
ENSCAFG00845003609	923	832	879	876
ENSCAFG00845003608	1128	1208	1104	1184
ENSCAFG00845003607	387	310	346	397
ENSCAFG00845003606	2136	1926	1864	1922
ENSCAFG00845003605	1048	984	1058	1115
ENSCAFG00845003604	0	0	0	0
ENSCAFG00845003603	1	0	0	1
ENSCAFG00845003602	642	628	541	579
ENSCAFG00845003601	1714	1570	1702	1569
ENSCAFG00845003600	368	342	396	347
ENSCAFG00845003619	0	0	0	1
ENSCAFG00845003618	3	3	0	3
ENSCAFG00845003617	0	0	0	0
ENSCAFG00845003616	3408	3144	3089	3213
ENSCAFG00845003615	299	323	308	347
ENSCAFG00845003614	12	7	5	11
ENSCAFG00845003613	23	32	21	33
ENSCAFG00845003612	0	1	0	4
ENSCAFG00845003611	393	440	421	395
ENSCAFG00845003610	0	0	0	0
ENSCAFG00845013032	2	1	3	5
ENSCAFG00845015695	0	0	0	0
ENSCAFG00845013031	377	388	368	330
ENSCAFG00845015696	0	0	1	0

ENSCAFG00845013034	0	3	4	3
ENSCAFG00845015693	7	9	9	12
ENSCAFG00845013033	0	0	0	0
ENSCAFG00845015694	0	0	0	0
ENSCAFG00845015691	0	0	0	0
ENSCAFG00845015692	0	0	0	0
ENSCAFG00845013030	2050	2013	2158	2079
ENSCAFG00845015690	0	0	0	0
ENSCAFG00845025018	881	861	883	897
ENSCAFG00845025019	426	398	388	407
ENSCAFG00845001049	407	396	406	458
ENSCAFG00845001047	6	2	8	5
ENSCAFG00845025014	541	481	512	474
ENSCAFG00845027677	16827	16335	15599	15927
ENSCAFG00845001048	0	0	0	0
ENSCAFG00845025015	0	0	0	0
ENSCAFG00845027676	8	11	3	9
ENSCAFG00845001045	239	274	254	264
ENSCAFG00845025016	0	0	0	0
ENSCAFG00845027679	1	1	2	1
ENSCAFG00845001046	1661	1547	1584	1670
ENSCAFG00845025017	7	6	2	10
ENSCAFG00845027678	877	841	829	882
ENSCAFG00845001043	0	0	0	0
ENSCAFG00845025010	0	0	0	0
ENSCAFG00845027673	0	0	0	0
ENSCAFG00845001044	10	9	11	15
ENSCAFG00845025011	0	0	0	0
ENSCAFG00845027672	0	0	0	0
ENSCAFG00845001041	0	0	0	0
ENSCAFG00845025012	804	800	735	704
ENSCAFG00845027675	0	0	0	0
ENSCAFG00845001042	0	0	0	0
ENSCAFG00845025013	6	1	16	1
ENSCAFG00845027674	654	570	600	624
ENSCAFG00845001040	7	11	9	11
ENSCAFG00845027671	0	0	0	0
ENSCAFG00845027670	307	267	320	328
ENSCAFG00845013039	0	4	6	6
ENSCAFG00845013036	0	0	0	0
ENSCAFG00845015699	0	0	0	0

ENSCAFG00845013035	205	177	104	144
ENSCAFG00845013038	51	53	44	38
ENSCAFG00845015697	352	262	370	396
ENSCAFG00845013037	32	25	26	33
ENSCAFG00845015698	0	0	1	0
ENSCAFG00845013021	881	874	884	914
ENSCAFG00845015684	43	54	34	25
ENSCAFG00845013020	3174	2889	2981	3221
ENSCAFG00845015685	545	564	619	681
ENSCAFG00845013023	0	0	0	0
ENSCAFG00845015682	2	7	2	9
ENSCAFG00845013022	165	152	181	167
ENSCAFG00845015683	1	3	1	10
ENSCAFG00845015680	150	181	140	151
ENSCAFG00845015681	0	0	0	0
ENSCAFG00845025007	2	2	6	8
ENSCAFG00845025008	255	185	249	231
ENSCAFG00845027669	7	3	2	4
ENSCAFG00845025009	8	5	4	8
ENSCAFG00845001058	0	0	0	0
ENSCAFG00845025003	0	0	0	0
ENSCAFG00845027666	4	4	2	0
ENSCAFG00845001059	273	273	302	277
ENSCAFG00845025004	0	0	5	3
ENSCAFG00845027665	160	190	143	123
ENSCAFG00845001056	0	0	0	0
ENSCAFG00845025005	1	0	0	0
ENSCAFG00845027668	1	0	1	0
ENSCAFG00845001057	1083	1126	986	1108
ENSCAFG00845025006	0	0	0	0
ENSCAFG00845027667	0	0	0	0
ENSCAFG00845001054	0	3	1	2
ENSCAFG00845027662	384	345	393	448
ENSCAFG00845001055	0	0	0	0
ENSCAFG00845025000	1881	1858	1836	1775
ENSCAFG00845027661	67	77	59	49
ENSCAFG00845001052	0	0	0	0
ENSCAFG00845025001	90	82	88	98
ENSCAFG00845027664	5964	5448	5617	5642
ENSCAFG00845001053	0	0	0	0
ENSCAFG00845025002	0	0	0	0

ENSCAFG00845027663	2204	2201	2232	2160
ENSCAFG00845001050	1446	1338	1197	1244
ENSCAFG00845001051	0	0	0	0
ENSCAFG00845027660	136	142	150	153
ENSCAFG00845013029	1777	1680	1484	1437
ENSCAFG00845013028	69	117	141	122
ENSCAFG00845013025	24	22	27	26
ENSCAFG00845015688	0	2	0	0
ENSCAFG00845013024	0	0	0	0
ENSCAFG00845015689	0	0	0	0
ENSCAFG00845013027	0	0	0	0
ENSCAFG00845015686	109	95	112	94
ENSCAFG00845013026	159	131	110	126
ENSCAFG00845015687	0	0	0	0
ENSCAFG00845013010	416	407	422	411
ENSCAFG00845015673	23	16	25	41
ENSCAFG00845015674	8420	7823	9112	9387
ENSCAFG00845013012	314	297	294	347
ENSCAFG00845015671	4	0	2	6
ENSCAFG00845013011	3189	3088	2907	2852
ENSCAFG00845015672	0	0	0	0
ENSCAFG00845015670	43	28	18	14
ENSCAFG00845001029	372	381	347	378
ENSCAFG00845027659	0	0	0	0
ENSCAFG00845027658	2450	2291	2373	2256
ENSCAFG00845001027	0	0	0	0
ENSCAFG00845001028	673	621	587	643
ENSCAFG00845003689	8	22	10	10
ENSCAFG00845001025	2	2	2	0
ENSCAFG00845003688	0	0	0	1
ENSCAFG00845027655	0	0	0	0
ENSCAFG00845001026	705	706	650	632
ENSCAFG00845003687	0	0	0	0
ENSCAFG00845027654	1380	1321	1547	1494
ENSCAFG00845001023	63	81	71	68
ENSCAFG00845003686	53	64	66	58
ENSCAFG00845001024	0	0	0	0
ENSCAFG00845003685	396	412	361	379
ENSCAFG00845027656	1	0	5	11
ENSCAFG00845001021	0	0	0	0
ENSCAFG00845003684	17	33	19	26

ENSCAFG00845027651	111	132	91	101
ENSCAFG00845001022	0	0	0	0
ENSCAFG00845003683	0	0	0	0
ENSCAFG00845027650	0	0	0	0
ENSCAFG00845003682	1	1	1	4
ENSCAFG00845027653	12	10	9	11
ENSCAFG00845001020	0	0	0	0
ENSCAFG00845003681	1195	1217	1091	1139
ENSCAFG00845027652	591	556	498	492
ENSCAFG00845003680	773	751	791	840
ENSCAFG00845013018	218	163	218	172
ENSCAFG00845013017	0	0	0	0
ENSCAFG00845015679	4	2	1	3
ENSCAFG00845013019	6038	5778	5402	5687
ENSCAFG00845013014	0	0	0	0
ENSCAFG00845015677	0	0	0	0
ENSCAFG00845013013	0	0	0	0
ENSCAFG00845015678	92	92	88	72
ENSCAFG00845013016	0	1	0	2
ENSCAFG00845015675	0	0	0	0
ENSCAFG00845013015	0	1	2	3
ENSCAFG00845015676	0	0	0	0
ENSCAFG00845015662	490	466	431	397
ENSCAFG00845015663	641	664	547	615
ENSCAFG00845013001	10	14	18	11
ENSCAFG00845015660	14163	13664	13836	14068
ENSCAFG00845013000	1048	1015	1032	1065
ENSCAFG00845015661	0	0	0	0
ENSCAFG00845027648	0	0	0	0
ENSCAFG00845027647	1550	1496	1353	1415
ENSCAFG00845001038	0	0	0	0
ENSCAFG00845001039	0	0	0	0
ENSCAFG00845027649	0	0	0	0
ENSCAFG00845001036	8	0	0	0
ENSCAFG00845003699	3	4	2	2
ENSCAFG00845027644	0	0	0	0
ENSCAFG00845001037	2011	1840	2257	2084
ENSCAFG00845003698	2	1	4	0
ENSCAFG00845027643	28	25	26	20
ENSCAFG00845001034	0	0	0	0
ENSCAFG00845003697	0	0	0	0

ENSCAFG00845027646	0	0	0	0
ENSCAFG00845001035	0	2	1	1
ENSCAFG00845003696	0	0	0	0
ENSCAFG00845027645	0	0	0	0
ENSCAFG00845001032	0	0	0	0
ENSCAFG00845003695	295	336	319	306
ENSCAFG00845027640	4920	4712	4233	4137
ENSCAFG00845001033	9	13	13	8
ENSCAFG00845003694	2	0	2	7
ENSCAFG00845001030	8	7	7	7
ENSCAFG00845003693	0	0	0	0
ENSCAFG00845027642	0	0	0	0
ENSCAFG00845001031	246	240	224	215
ENSCAFG00845003692	1334	1365	1131	1150
ENSCAFG00845027641	0	0	0	0
ENSCAFG00845003691	0	0	0	0
ENSCAFG00845003690	0	0	0	0
ENSCAFG00845013007	2225	2304	2258	2383
ENSCAFG00845013006	897	953	844	925
ENSCAFG00845013009	45	36	36	37
ENSCAFG00845015668	985	1021	954	948
ENSCAFG00845013008	156	162	189	149
ENSCAFG00845015669	6	7	6	8
ENSCAFG00845013003	27	34	35	34
ENSCAFG00845015666	3451	3450	2148	2097
ENSCAFG00845013002	0	1	0	2
ENSCAFG00845015667	119	143	122	131
ENSCAFG00845013005	4214	4260	3960	4135
ENSCAFG00845015664	0	0	0	0
ENSCAFG00845013004	0	0	0	0
ENSCAFG00845015665	26	47	37	47
ENSCAFG00845015651	0	0	0	0
ENSCAFG00845015652	0	0	0	0
ENSCAFG00845015650	262	247	249	216
ENSCAFG00845001009	4	4	4	5
ENSCAFG00845001007	5757	5883	5926	6021
ENSCAFG00845027637	0	0	0	0
ENSCAFG00845001008	0	0	0	0
ENSCAFG00845003669	615	576	657	723
ENSCAFG00845027636	1466	1377	1379	1478
ENSCAFG00845001005	0	0	0	0

ENSCAFG00845003668	0	0	0	0
ENSCAFG00845027639	799	855	814	793
ENSCAFG00845001006	2	0	0	1
ENSCAFG00845003667	0	0	0	0
ENSCAFG00845027638	354	348	326	320
ENSCAFG00845001003	1541	1494	1355	1254
ENSCAFG00845003666	0	0	0	0
ENSCAFG00845027633	0	2	1	0
ENSCAFG00845001004	1425	1442	1418	1390
ENSCAFG00845003665	0	0	0	0
ENSCAFG00845027632	150	150	153	156
ENSCAFG00845001001	0	0	0	0
ENSCAFG00845003664	892	789	786	750
ENSCAFG00845027635	0	0	0	0
ENSCAFG00845001002	3	5	3	3
ENSCAFG00845003663	0	0	0	0
ENSCAFG00845027634	0	0	0	0
ENSCAFG00845003662	1677	1568	1536	1463
ENSCAFG00845001000	1	0	0	0
ENSCAFG00845003661	2	0	0	0
ENSCAFG00845003660	0	0	0	0
ENSCAFG00845027631	40	37	35	35
ENSCAFG00845027630	0	0	0	0
ENSCAFG00845015659	6	11	10	13
ENSCAFG00845015657	97	110	94	75
ENSCAFG00845015658	31	20	21	21
ENSCAFG00845015655	0	0	0	0
ENSCAFG00845015656	0	0	0	0
ENSCAFG00845015653	1	0	0	0
ENSCAFG00845015654	2	0	1	0
ENSCAFG00845015640	17	14	26	15
ENSCAFG00845015641	0	0	0	0
ENSCAFG00845027629	2536	2313	1860	2045
ENSCAFG00845001018	0	0	0	0
ENSCAFG00845027626	2639	2612	2711	2747
ENSCAFG00845001019	9	2	14	5
ENSCAFG00845027625	1	0	0	0
ENSCAFG00845001016	0	0	0	0
ENSCAFG00845003679	3	4	1	1
ENSCAFG00845027628	6677	6341	6354	6413
ENSCAFG00845001017	40	29	33	23

ENSCAFG00845003678	1	0	2	2
ENSCAFG00845027627	43	40	72	75
ENSCAFG00845001014	0	0	0	1
ENSCAFG00845003677	0	0	0	0
ENSCAFG00845027622	0	0	0	0
ENSCAFG00845001015	0	0	0	0
ENSCAFG00845003676	0	0	0	1
ENSCAFG00845027621	5	9	9	13
ENSCAFG00845001012	0	0	0	0
ENSCAFG00845003675	35	42	44	48
ENSCAFG00845027624	0	0	0	0
ENSCAFG00845001013	1664	1626	1495	1579
ENSCAFG00845003674	0	0	0	0
ENSCAFG00845027623	0	0	0	0
ENSCAFG00845001010	56	40	44	33
ENSCAFG00845003673	5	0	2	1
ENSCAFG00845001011	0	0	1	2
ENSCAFG00845003672	11	17	4	5
ENSCAFG00845003671	0	0	0	0
ENSCAFG00845027620	2	0	5	1
ENSCAFG00845003670	0	4	1	0
ENSCAFG00845015648	153	209	115	129
ENSCAFG00845015649	11	9	12	11
ENSCAFG00845015646	0	0	0	0
ENSCAFG00845015647	160	140	110	130
ENSCAFG00845015644	0	2	1	4
ENSCAFG00845015645	381	382	322	372
ENSCAFG00845015642	0	0	0	0
ENSCAFG00845015643	1	0	0	3
ENSCAFG00845015630	421	394	341	332
ENSCAFG00845027619	429	418	362	330
ENSCAFG00845027618	1122	1160	1121	1121
ENSCAFG00845003649	0	0	0	0
ENSCAFG00845003648	0	0	0	0
ENSCAFG00845027615	0	0	0	0
ENSCAFG00845003647	1318	1247	1310	1227
ENSCAFG00845003646	912	917	1087	1067
ENSCAFG00845027617	41	34	31	53
ENSCAFG00845003645	0	0	0	0
ENSCAFG00845027616	0	0	0	0
ENSCAFG00845003644	353	316	338	301

ENSCAFG00845027611	8	9	8	2
ENSCAFG00845003643	110	100	105	110
ENSCAFG00845027610	1883	1942	1852	1992
ENSCAFG00845003642	0	0	0	0
ENSCAFG00845027613	2	7	2	0
ENSCAFG00845003641	80	94	65	111
ENSCAFG00845027612	0	0	0	0
ENSCAFG00845003640	164	159	146	167
ENSCAFG00845015639	2	3	2	4
ENSCAFG00845015637	52	39	48	42
ENSCAFG00845015638	1136	995	1007	1110
ENSCAFG00845015635	1	0	0	0
ENSCAFG00845015636	3	2	0	2
ENSCAFG00845015633	0	0	0	0
ENSCAFG00845015634	0	0	0	0
ENSCAFG00845015631	0	0	0	0
ENSCAFG00845015632	2	0	0	0
ENSCAFG00845027608	3	0	5	2
ENSCAFG00845027607	706	610	668	687
ENSCAFG00845027609	923	968	980	973
ENSCAFG00845003659	2	0	1	0
ENSCAFG00845027604	1997	1912	1932	2007
ENSCAFG00845003658	599	607	653	627
ENSCAFG00845027603	75	61	58	48
ENSCAFG00845003657	153	175	141	157
ENSCAFG00845027606	2042	1911	1895	1997
ENSCAFG00845003656	0	0	0	0
ENSCAFG00845027605	14	7	5	7
ENSCAFG00845003655	4	1	5	1
ENSCAFG00845027600	0	0	0	0
ENSCAFG00845003654	0	0	0	0
ENSCAFG00845003653	0	0	0	0
ENSCAFG00845027602	1	2	1	4
ENSCAFG00845003652	327	312	293	278
ENSCAFG00845027601	0	0	0	0
ENSCAFG00845003651	2566	2730	2596	2602
ENSCAFG00845003650	17	23	13	18
ENSCAFG00845015628	0	0	0	0
ENSCAFG00845015629	31	22	25	41
ENSCAFG00845015626	252	263	316	296
ENSCAFG00845015627	0	0	0	0

ENSCAFG00845015624	0	0	0	0
ENSCAFG00845015625	2	1	3	1
ENSCAFG00845015622	696	619	764	810
ENSCAFG00845015623	0	0	0	0
ENSCAFG00845015620	550	558	565	551
ENSCAFG00845015621	757	775	561	692
ENSCAFG00845025197	0	0	0	0
ENSCAFG00845025198	0	1	0	0
ENSCAFG00845025199	0	0	0	0
ENSCAFG00845025193	302	274	334	339
ENSCAFG00845025194	113	85	96	90
ENSCAFG00845025195	0	0	0	0
ENSCAFG00845025196	260	294	249	249
ENSCAFG00845025190	419	443	400	407
ENSCAFG00845025191	11	4	6	4
ENSCAFG00845025192	9	17	5	12
ENSCAFG00845025186	0	0	0	0
ENSCAFG00845025188	0	0	0	0
ENSCAFG00845025189	0	0	0	0
ENSCAFG00845025182	2212	2084	1846	2078
ENSCAFG00845025183	335	336	305	322
ENSCAFG00845025184	0	0	0	0
ENSCAFG00845025180	0	0	0	0
ENSCAFG00845025181	0	0	0	0
ENSCAFG00845013197	0	0	0	0
ENSCAFG00845013196	0	0	0	0
ENSCAFG00845013199	0	0	0	0
ENSCAFG00845013198	2	2	4	3
ENSCAFG00845013193	177	167	181	137
ENSCAFG00845013192	24	35	15	27
ENSCAFG00845013195	0	0	0	0
ENSCAFG00845013194	179	164	147	201
ENSCAFG00845013191	0	0	0	0
ENSCAFG00845013190	239	200	222	247
ENSCAFG00845025179	0	0	0	0
ENSCAFG00845025175	10	16	17	22
ENSCAFG00845025176	0	0	0	1
ENSCAFG00845025177	0	0	0	0
ENSCAFG00845025171	0	1	2	0
ENSCAFG00845025172	0	0	0	0
ENSCAFG00845025173	0	0	0	0

ENSCAFG00845025170	0	0	0	0
ENSCAFG00845013186	160	143	147	151
ENSCAFG00845013185	233	218	248	194
ENSCAFG00845013188	138	133	145	119
ENSCAFG00845013187	0	4	0	2
ENSCAFG00845013182	56	25	26	21
ENSCAFG00845013181	56	60	51	48
ENSCAFG00845013184	0	0	0	0
ENSCAFG00845013183	3788	3493	4194	4325
ENSCAFG00845013180	611	593	660	651
ENSCAFG00845025168	52	72	67	49
ENSCAFG00845025169	0	0	0	0
ENSCAFG00845025164	0	4	0	1
ENSCAFG00845025165	2	4	1	2
ENSCAFG00845025166	29	36	16	34
ENSCAFG00845025167	0	0	0	0
ENSCAFG00845025160	0	0	0	0
ENSCAFG00845025161	74	78	84	59
ENSCAFG00845025162	0	1	1	2
ENSCAFG00845025163	719	663	686	690
ENSCAFG00845013189	993	963	844	873
ENSCAFG00845013175	3	9	11	3
ENSCAFG00845013174	0	0	0	0
ENSCAFG00845013177	1171	1163	1166	1131
ENSCAFG00845013176	7	5	11	11
ENSCAFG00845013171	49	45	54	47
ENSCAFG00845013170	0	0	0	0
ENSCAFG00845013173	175	161	137	213
ENSCAFG00845013172	12491	12134	12332	12383
ENSCAFG00845025158	1	0	0	0
ENSCAFG00845001188	2	7	2	6
ENSCAFG00845025159	1	0	0	3
ENSCAFG00845001189	838	811	850	887
ENSCAFG00845001186	0	0	0	0
ENSCAFG00845025153	0	0	0	0
ENSCAFG00845001187	18	27	34	32
ENSCAFG00845025154	0	0	0	0
ENSCAFG00845001184	4	3	1	12
ENSCAFG00845025155	2	1	1	5
ENSCAFG00845001185	7	3	3	0
ENSCAFG00845025156	0	0	0	0

ENSCAFG00845001182	2	0	0	0
ENSCAFG00845001183	0	0	0	0
ENSCAFG00845025150	0	0	0	0
ENSCAFG00845001180	355	307	286	330
ENSCAFG00845025151	0	0	0	0
ENSCAFG00845001181	0	0	2	1
ENSCAFG00845025152	0	1	2	2
ENSCAFG00845013179	0	0	0	0
ENSCAFG00845013178	833	819	886	847
ENSCAFG00845013164	0	0	2	0
ENSCAFG00845013163	1580	1551	1440	1541
ENSCAFG00845013166	0	0	0	0
ENSCAFG00845013165	1648	1671	1432	1518
ENSCAFG00845013160	1	3	4	2
ENSCAFG00845013162	100	87	74	57
ENSCAFG00845013161	0	0	0	0
ENSCAFG00845025146	0	0	0	0
ENSCAFG00845025147	0	0	0	0
ENSCAFG00845001199	0	0	0	0
ENSCAFG00845025148	598	614	533	477
ENSCAFG00845025149	0	3	1	1
ENSCAFG00845001197	1	0	3	1
ENSCAFG00845025142	4	6	7	4
ENSCAFG00845001198	0	0	0	0
ENSCAFG00845025143	5	7	5	11
ENSCAFG00845001195	0	0	0	0
ENSCAFG00845025144	0	1	3	2
ENSCAFG00845001196	6157	5990	5694	5853
ENSCAFG00845025145	856	905	859	927
ENSCAFG00845001193	3	3	2	6
ENSCAFG00845001194	346	335	342	336
ENSCAFG00845001191	1187	1071	1133	1161
ENSCAFG00845001192	0	0	0	0
ENSCAFG00845025141	3	2	2	0
ENSCAFG00845001190	0	0	0	0
ENSCAFG00845013168	0	0	0	0
ENSCAFG00845013167	0	0	0	0
ENSCAFG00845013169	311	304	328	331
ENSCAFG00845027718	0	0	1	0
ENSCAFG00845027717	0	2	0	3
ENSCAFG00845003749	323	373	462	426

ENSCAFG00845003748	1592	1500	1707	1733
ENSCAFG00845027719	1790	1594	1807	1833
ENSCAFG00845003747	0	0	0	0
ENSCAFG00845027714	0	0	0	0
ENSCAFG00845003746	833	788	754	772
ENSCAFG00845027713	953	991	933	915
ENSCAFG00845003745	659	677	503	520
ENSCAFG00845027716	2	4	4	3
ENSCAFG00845003744	191	217	300	293
ENSCAFG00845003743	1693	1649	1603	1532
ENSCAFG00845027710	0	0	0	0
ENSCAFG00845003742	0	0	0	0
ENSCAFG00845003741	112	153	142	145
ENSCAFG00845027712	1	0	0	0
ENSCAFG00845003740	0	0	0	0
ENSCAFG00845027711	1	0	0	0
ENSCAFG00845015738	43	25	27	38
ENSCAFG00845015739	0	0	0	0
ENSCAFG00845015736	1	0	0	0
ENSCAFG00845015737	2	2	0	1
ENSCAFG00845015734	432	401	324	395
ENSCAFG00845015735	0	0	0	0
ENSCAFG00845015732	52	42	69	79
ENSCAFG00845015733	35	29	37	29
ENSCAFG00845015730	0	0	0	0
ENSCAFG00845015731	0	1	2	0
ENSCAFG00845027707	630	553	556	583
ENSCAFG00845027706	2947	2747	2550	2698
ENSCAFG00845027709	0	0	0	0
ENSCAFG00845003759	0	0	0	0
ENSCAFG00845027708	2	4	3	0
ENSCAFG00845003758	0	0	0	0
ENSCAFG00845027703	2	0	0	5
ENSCAFG00845003757	0	0	0	0
ENSCAFG00845027702	1842	1778	1764	1831
ENSCAFG00845003756	96	94	87	98
ENSCAFG00845027705	0	0	0	0
ENSCAFG00845003755	0	0	0	0
ENSCAFG00845027704	190	194	153	143
ENSCAFG00845003754	5306	5121	5340	5561
ENSCAFG00845003753	1658	1676	1840	1654

ENSCAFG00845003752	0	0	0	1
ENSCAFG00845027701	1396	1281	1187	1282
ENSCAFG00845003751	0	0	1	2
ENSCAFG00845027700	0	0	0	0
ENSCAFG00845003750	344	305	259	280
ENSCAFG00845015729	261	283	221	194
ENSCAFG00845015727	0	0	0	0
ENSCAFG00845015728	425	449	480	427
ENSCAFG00845015725	311	285	285	288
ENSCAFG00845015726	0	0	0	0
ENSCAFG00845015723	0	0	0	0
ENSCAFG00845015724	0	0	2	1
ENSCAFG00845015721	0	0	0	0
ENSCAFG00845015722	0	0	0	0
ENSCAFG00845015720	0	0	0	0
ENSCAFG00845003729	0	0	0	1
ENSCAFG00845003728	10	8	12	10
ENSCAFG00845003727	8	2	1	10
ENSCAFG00845003726	809	722	625	632
ENSCAFG00845003725	171	165	149	156
ENSCAFG00845003724	1	0	2	0
ENSCAFG00845003723	0	3	3	1
ENSCAFG00845003722	166	150	116	108
ENSCAFG00845003721	0	1	0	0
ENSCAFG00845003720	203	218	259	199
ENSCAFG00845015718	0	0	0	0
ENSCAFG00845015719	0	0	0	0
ENSCAFG00845015716	1344	1146	1189	1304
ENSCAFG00845015717	0	0	0	0
ENSCAFG00845015714	0	0	0	0
ENSCAFG00845015715	0	0	0	0
ENSCAFG00845015712	82	71	76	80
ENSCAFG00845015713	0	0	0	0
ENSCAFG00845015710	0	0	0	0
ENSCAFG00845015711	14	9	11	7
ENSCAFG00845003739	354	305	334	314
ENSCAFG00845003738	1	0	0	0
ENSCAFG00845003737	0	6	2	2
ENSCAFG00845003736	0	0	0	0
ENSCAFG00845003735	47	37	51	39
ENSCAFG00845003734	553	534	566	584

ENSCAFG00845003733	0	0	0	0
ENSCAFG00845003732	12	16	18	19
ENSCAFG00845003731	1765	1757	1680	1528
ENSCAFG00845003730	4	2	5	4
ENSCAFG00845015709	7	11	23	11
ENSCAFG00845015707	0	0	0	0
ENSCAFG00845015708	0	0	0	0
ENSCAFG00845015705	0	0	4	0
ENSCAFG00845015706	2	2	8	2
ENSCAFG00845015703	104	78	76	73
ENSCAFG00845015704	0	6	2	1
ENSCAFG00845015701	0	0	0	0
ENSCAFG00845015702	0	0	0	0
ENSCAFG00845015700	0	0	0	0
ENSCAFG00845003709	1389	1427	1425	1462
ENSCAFG00845003708	2	0	0	0
ENSCAFG00845003707	1	0	0	0
ENSCAFG00845003706	86	99	74	83
ENSCAFG00845003705	0	0	0	1
ENSCAFG00845003704	0	0	0	0
ENSCAFG00845003703	669	638	626	613
ENSCAFG00845003702	74	71	63	81
ENSCAFG00845003701	0	0	0	0
ENSCAFG00845003700	531	528	569	583
ENSCAFG00845003719	1341	1342	1383	1349
ENSCAFG00845003718	0	0	0	1
ENSCAFG00845003717	0	0	0	0
ENSCAFG00845003716	2969	2793	2876	2849
ENSCAFG00845003715	0	0	0	0
ENSCAFG00845003714	0	0	0	0
ENSCAFG00845003713	1360	1454	1301	1380
ENSCAFG00845003712	1	1	0	2
ENSCAFG00845003711	38	31	26	26
ENSCAFG00845003710	4	4	3	2
ENSCAFG00845013153	0	1	0	1
ENSCAFG00845013152	0	0	0	0
ENSCAFG00845013155	3441	3381	3543	3601
ENSCAFG00845013154	1	0	2	1
ENSCAFG00845013151	527	472	412	402
ENSCAFG00845013150	2	4	3	4
ENSCAFG00845025139	0	0	0	0

ENSCAFG00845001168	27	17	10	2
ENSCAFG00845025135	0	0	0	0
ENSCAFG00845027798	1166	1093	1017	1071
ENSCAFG00845001169	293	261	259	305
ENSCAFG00845025136	0	0	0	0
ENSCAFG00845027797	383	359	339	340
ENSCAFG00845001166	27	30	28	44
ENSCAFG00845025137	482	509	468	475
ENSCAFG00845001167	0	0	0	0
ENSCAFG00845025138	0	0	0	0
ENSCAFG00845027799	2	0	1	0
ENSCAFG00845001164	254	264	254	244
ENSCAFG00845025131	833	833	965	962
ENSCAFG00845027794	115	141	130	153
ENSCAFG00845001165	234	197	247	197
ENSCAFG00845025132	578	543	585	565
ENSCAFG00845027793	1	0	0	0
ENSCAFG00845001162	1	3	0	2
ENSCAFG00845025133	553	520	541	492
ENSCAFG00845027796	0	0	0	0
ENSCAFG00845001163	4	2	1	3
ENSCAFG00845025134	329	354	348	345
ENSCAFG00845001160	2	0	0	0
ENSCAFG00845027790	0	0	0	0
ENSCAFG00845001161	431	367	356	389
ENSCAFG00845027792	531	504	512	509
ENSCAFG00845025130	29	17	12	26
ENSCAFG00845027791	2	1	0	0
ENSCAFG00845013157	19	9	14	8
ENSCAFG00845013156	632	592	545	551
ENSCAFG00845013159	1	4	1	4
ENSCAFG00845013158	1	1	2	7
ENSCAFG00845013142	0	0	0	0
ENSCAFG00845013141	715	578	667	659
ENSCAFG00845013144	0	0	1	0
ENSCAFG00845013143	316	321	324	322
ENSCAFG00845013140	0	0	0	0
ENSCAFG00845025128	705	604	586	633
ENSCAFG00845025129	3261	2994	3341	3242
ENSCAFG00845001179	0	0	2	1
ENSCAFG00845025124	0	0	0	0

ENSCAFG00845027787	35	49	31	44
ENSCAFG00845027786	384	387	473	413
ENSCAFG00845001177	5	1	2	7
ENSCAFG00845025126	0	0	0	0
ENSCAFG00845027789	355	346	354	369
ENSCAFG00845001178	232	238	223	265
ENSCAFG00845025127	110	98	77	78
ENSCAFG00845027788	8	11	2	1
ENSCAFG00845001175	11	9	7	5
ENSCAFG00845025120	89	113	70	67
ENSCAFG00845027783	0	0	0	0
ENSCAFG00845001176	142	126	79	92
ENSCAFG00845025121	3	0	0	0
ENSCAFG00845027782	8	2	12	4
ENSCAFG00845001173	394	365	365	456
ENSCAFG00845025122	5903	5654	6533	6686
ENSCAFG00845027785	1261	1245	1283	1255
ENSCAFG00845001174	0	0	0	0
ENSCAFG00845025123	1832	1838	1807	1810
ENSCAFG00845027784	207	199	169	181
ENSCAFG00845001171	0	0	0	0
ENSCAFG00845001172	0	0	0	0
ENSCAFG00845027781	4	2	3	0
ENSCAFG00845001170	328	308	325	297
ENSCAFG00845027780	3	0	1	1
ENSCAFG00845013149	220	241	185	252
ENSCAFG00845013146	3	10	8	6
ENSCAFG00845013145	1	3	2	6
ENSCAFG00845013148	0	0	0	0
ENSCAFG00845013147	9	11	8	8
ENSCAFG00845013131	0	0	0	1
ENSCAFG00845015794	2097	2019	1969	2106
ENSCAFG00845013130	211	162	198	197
ENSCAFG00845015795	0	0	0	0
ENSCAFG00845013133	0	0	0	0
ENSCAFG00845015792	0	0	2	0
ENSCAFG00845013132	0	2	1	4
ENSCAFG00845015793	0	1	1	0
ENSCAFG00845015790	0	0	0	0
ENSCAFG00845015791	0	0	0	0
ENSCAFG00845025117	1015	941	895	846

ENSCAFG00845025118	7	4	6	12
ENSCAFG00845027779	809	762	695	711
ENSCAFG00845001148	0	0	0	0
ENSCAFG00845025119	3	0	0	0
ENSCAFG00845001149	234	248	231	242
ENSCAFG00845001146	2	4	5	1
ENSCAFG00845025113	5	0	2	2
ENSCAFG00845027776	175	157	163	178
ENSCAFG00845001147	2549	2541	2135	2115
ENSCAFG00845025114	1039	927	967	1028
ENSCAFG00845027775	1032	913	1189	966
ENSCAFG00845001144	0	0	0	0
ENSCAFG00845025115	0	0	0	0
ENSCAFG00845027778	0	0	0	0
ENSCAFG00845001145	0	2	0	0
ENSCAFG00845025116	199	191	197	200
ENSCAFG00845027777	446	495	491	504
ENSCAFG00845001142	0	1	2	0
ENSCAFG00845027772	3	0	0	3
ENSCAFG00845001143	1	0	0	0
ENSCAFG00845025110	235	280	296	278
ENSCAFG00845027771	883	842	837	785
ENSCAFG00845001140	266	272	179	164
ENSCAFG00845025111	324	310	301	360
ENSCAFG00845027774	3	2	1	1
ENSCAFG00845001141	78	89	67	67
ENSCAFG00845025112	1205	1204	1091	1166
ENSCAFG00845027773	68	77	61	64
ENSCAFG00845027770	533	643	635	690
ENSCAFG00845013139	2162	2098	2259	2329
ENSCAFG00845013138	0	0	0	0
ENSCAFG00845013135	2	0	8	5
ENSCAFG00845015798	0	0	0	0
ENSCAFG00845013134	752	745	820	859
ENSCAFG00845015799	41	50	40	56
ENSCAFG00845013137	0	0	0	0
ENSCAFG00845015796	0	0	0	0
ENSCAFG00845013136	8909	8456	7966	8092
ENSCAFG00845015797	0	0	0	0
ENSCAFG00845013120	0	0	0	0
ENSCAFG00845015783	20	21	11	10

ENSCAFG00845015784	6658	6215	6294	6149
ENSCAFG00845013122	25	29	26	16
ENSCAFG00845015781	538	485	592	549
ENSCAFG00845013121	0	0	0	1
ENSCAFG00845015782	0	0	0	0
ENSCAFG00845015780	2	3	1	2
ENSCAFG00845025106	0	0	0	0
ENSCAFG00845027769	0	0	0	0
ENSCAFG00845025107	0	6	1	1
ENSCAFG00845027768	0	0	0	0
ENSCAFG00845001159	112	93	77	93
ENSCAFG00845025108	836	695	640	811
ENSCAFG00845025109	0	0	0	0
ENSCAFG00845001157	1800	1728	1706	1625
ENSCAFG00845025102	50	60	38	35
ENSCAFG00845027765	37	60	47	59
ENSCAFG00845001158	220	216	158	177
ENSCAFG00845025103	269	227	258	294
ENSCAFG00845027764	1282	1127	1164	1190
ENSCAFG00845001155	58	33	32	39
ENSCAFG00845027767	0	0	0	0
ENSCAFG00845001156	0	0	5	1
ENSCAFG00845025105	0	1	0	0
ENSCAFG00845027766	28	24	32	18
ENSCAFG00845001153	1190	1130	1161	1155
ENSCAFG00845027761	0	2	0	1
ENSCAFG00845001154	582	535	530	567
ENSCAFG00845027760	226	205	214	214
ENSCAFG00845001151	456	474	483	448
ENSCAFG00845025100	0	0	1	0
ENSCAFG00845027763	723	740	816	807
ENSCAFG00845001152	0	0	0	0
ENSCAFG00845025101	0	0	0	0
ENSCAFG00845027762	7	11	2	4
ENSCAFG00845001150	2262	2335	2229	2221
ENSCAFG00845013128	1	4	7	8
ENSCAFG00845013127	0	0	0	0
ENSCAFG00845015789	0	0	0	0
ENSCAFG00845013129	39	12	28	24
ENSCAFG00845013124	757	776	795	781
ENSCAFG00845015787	110	109	110	109

ENSCAFG00845013123	219	214	197	205
ENSCAFG00845015788	1118	1087	998	976
ENSCAFG00845013126	9	5	5	7
ENSCAFG00845015785	0	0	0	1
ENSCAFG00845013125	0	0	0	0
ENSCAFG00845015786	284	286	259	227
ENSCAFG00845015772	672	717	611	623
ENSCAFG00845015773	0	1	0	2
ENSCAFG00845013111	1516	1440	1403	1437
ENSCAFG00845015770	0	0	0	0
ENSCAFG00845013110	121	113	114	105
ENSCAFG00845015771	2	5	4	12
ENSCAFG00845001128	0	0	0	0
ENSCAFG00845027758	210	220	222	205
ENSCAFG00845001129	380	362	424	444
ENSCAFG00845027757	0	0	0	0
ENSCAFG00845001126	2992	2789	2819	2813
ENSCAFG00845003789	7517	7224	7115	7396
ENSCAFG00845001127	37	33	12	31
ENSCAFG00845003788	754	755	744	838
ENSCAFG00845027759	0	0	0	0
ENSCAFG00845001124	1	6	9	2
ENSCAFG00845003787	247	232	182	178
ENSCAFG00845027754	2960	2777	2828	2976
ENSCAFG00845001125	6	4	2	2
ENSCAFG00845003786	2811	2755	3201	3211
ENSCAFG00845027753	1398	1418	1475	1459
ENSCAFG00845001122	0	0	0	0
ENSCAFG00845003785	14	11	11	17
ENSCAFG00845027756	0	0	0	0
ENSCAFG00845001123	0	0	0	0
ENSCAFG00845003784	0	0	0	0
ENSCAFG00845027755	0	0	0	0
ENSCAFG00845001120	1846	1741	2055	2220
ENSCAFG00845003783	13831	13187	14638	14938
ENSCAFG00845027750	15	10	12	11
ENSCAFG00845001121	0	0	0	2
ENSCAFG00845003782	2298	2138	1917	2022
ENSCAFG00845003781	0	0	0	0
ENSCAFG00845027752	1621	1653	1734	1837
ENSCAFG00845003780	27	24	15	13

ENSCAFG00845027751	3046	2997	3044	2993
ENSCAFG00845013117	4	6	1	4
ENSCAFG00845013116	857	807	885	823
ENSCAFG00845013119	66	57	43	62
ENSCAFG00845015778	0	0	0	0
ENSCAFG00845013118	1	0	2	2
ENSCAFG00845015779	1231	1156	1040	1094
ENSCAFG00845013113	7	7	5	12
ENSCAFG00845015776	822	751	791	709
ENSCAFG00845013112	0	0	0	0
ENSCAFG00845015777	5	2	0	4
ENSCAFG00845013115	493	439	512	514
ENSCAFG00845015774	0	0	2	0
ENSCAFG00845013114	1	2	0	0
ENSCAFG00845015775	252	266	223	273
ENSCAFG00845015761	4801	4482	4778	5110
ENSCAFG00845015762	0	0	0	0
ENSCAFG00845013100	734	691	712	757
ENSCAFG00845015760	125	112	114	127
ENSCAFG00845001139	182	196	140	154
ENSCAFG00845027747	0	0	0	0
ENSCAFG00845027746	0	0	0	0
ENSCAFG00845001137	7559	7059	7313	7521
ENSCAFG00845027749	0	0	0	0
ENSCAFG00845001138	0	0	0	0
ENSCAFG00845003799	217	234	171	150
ENSCAFG00845027748	2113	2114	2198	2042
ENSCAFG00845001135	2	2	2	4
ENSCAFG00845003798	1595	1635	1533	1565
ENSCAFG00845027743	1024	975	941	935
ENSCAFG00845001136	0	1	2	0
ENSCAFG00845003797	14	11	10	7
ENSCAFG00845027742	793	681	764	843
ENSCAFG00845001133	19	19	39	38
ENSCAFG00845003796	666	627	510	575
ENSCAFG00845027745	27	11	7	12
ENSCAFG00845001134	291	345	300	319
ENSCAFG00845003795	0	0	0	0
ENSCAFG00845027744	0	0	0	0
ENSCAFG00845001131	0	0	0	0
ENSCAFG00845003794	0	0	0	0

ENSCAFG00845001132	0	0	0	0
ENSCAFG00845003793	0	3	1	0
ENSCAFG00845003792	1560	1492	1524	1467
ENSCAFG00845027741	0	1	4	0
ENSCAFG00845001130	376	322	299	366
ENSCAFG00845003791	321	282	308	356
ENSCAFG00845027740	1	8	1	0
ENSCAFG00845003790	0	0	0	0
ENSCAFG00845013109	294	292	286	304
ENSCAFG00845013106	429	385	361	455
ENSCAFG00845015769	131	105	135	120
ENSCAFG00845013105	0	0	0	0
ENSCAFG00845013108	273	254	250	232
ENSCAFG00845015767	7	7	14	13
ENSCAFG00845013107	1446	1431	1509	1553
ENSCAFG00845015768	0	0	0	0
ENSCAFG00845013102	82	73	88	72
ENSCAFG00845015765	16	12	8	11
ENSCAFG00845013101	10	9	14	4
ENSCAFG00845015766	0	0	0	0
ENSCAFG00845013104	2370	2265	2096	1996
ENSCAFG00845015763	14	14	7	14
ENSCAFG00845013103	206	230	227	192
ENSCAFG00845015764	0	0	0	0
ENSCAFG00845015750	309	304	393	349
ENSCAFG00845015751	288	239	227	209
ENSCAFG00845027739	0	0	2	2
ENSCAFG00845001108	769	770	764	805
ENSCAFG00845001109	0	0	0	0
ENSCAFG00845001106	867	783	898	864
ENSCAFG00845003769	1	0	0	0
ENSCAFG00845027736	691	644	674	661
ENSCAFG00845001107	2704	2599	2589	2530
ENSCAFG00845003768	0	0	0	2
ENSCAFG00845027735	0	0	0	0
ENSCAFG00845001104	1023	986	992	1022
ENSCAFG00845003767	215	177	238	229
ENSCAFG00845027738	551	567	461	464
ENSCAFG00845001105	477	421	476	552
ENSCAFG00845003766	0	0	0	0
ENSCAFG00845027737	13	2	1	7

ENSCAFG00845001102	10	7	5	10
ENSCAFG00845003765	12	0	3	8
ENSCAFG00845001103	53	36	40	40
ENSCAFG00845003764	0	0	0	3
ENSCAFG00845027731	3	1	1	5
ENSCAFG00845001100	175	224	184	224
ENSCAFG00845003763	0	0	0	1
ENSCAFG00845027734	3	6	1	0
ENSCAFG00845001101	0	0	0	0
ENSCAFG00845003762	0	0	0	0
ENSCAFG00845027733	4	2	3	1
ENSCAFG00845003761	0	0	0	0
ENSCAFG00845003760	0	0	0	0
ENSCAFG00845027730	1	0	0	1
ENSCAFG00845015758	0	0	0	0
ENSCAFG00845015759	2854	2840	2621	2556
ENSCAFG00845015756	1822	1804	2002	2140
ENSCAFG00845015757	6	5	3	7
ENSCAFG00845015754	356	358	362	418
ENSCAFG00845015755	0	1	0	0
ENSCAFG00845015752	0	0	0	0
ENSCAFG00845015753	0	0	0	0
ENSCAFG00845015740	644	596	642	712
ENSCAFG00845027729	160	132	135	143
ENSCAFG00845027728	687	654	615	665
ENSCAFG00845001119	0	0	0	0
ENSCAFG00845001117	247	234	249	220
ENSCAFG00845027725	1	2	0	3
ENSCAFG00845001118	0	0	0	0
ENSCAFG00845003779	0	2	1	1
ENSCAFG00845001115	0	0	0	0
ENSCAFG00845003778	0	0	0	0
ENSCAFG00845027727	0	0	0	0
ENSCAFG00845001116	0	0	0	0
ENSCAFG00845003777	123	145	157	180
ENSCAFG00845027726	0	0	0	0
ENSCAFG00845001113	387	402	390	406
ENSCAFG00845003776	377	418	296	307
ENSCAFG00845027721	0	0	0	0
ENSCAFG00845001114	183	183	219	215
ENSCAFG00845003775	1530	1536	1375	1367

ENSCAFG00845027720	4	1	1	0
ENSCAFG00845001111	0	0	0	0
ENSCAFG00845003774	0	0	0	0
ENSCAFG00845027723	0	0	0	0
ENSCAFG00845001112	20	13	15	16
ENSCAFG00845003773	0	0	0	0
ENSCAFG00845027722	0	0	0	0
ENSCAFG00845003772	0	1	0	0
ENSCAFG00845001110	20	20	21	27
ENSCAFG00845003771	0	0	0	0
ENSCAFG00845003770	1076	1010	994	1020
ENSCAFG00845015749	0	0	0	0
ENSCAFG00845015747	0	0	0	0
ENSCAFG00845015748	0	0	0	0
ENSCAFG00845015745	463	414	464	446
ENSCAFG00845015746	16	10	6	12
ENSCAFG00845015743	276	262	139	122
ENSCAFG00845015744	2778	2742	2502	2364
ENSCAFG00845015741	1687	1554	1955	1774
ENSCAFG00845015742	0	0	0	0
ENSCAFG00845025296	1279	1235	1106	1191
ENSCAFG00845025297	0	0	0	0
ENSCAFG00845025298	0	0	0	0
ENSCAFG00845025299	0	0	0	0
ENSCAFG00845025292	0	0	0	0
ENSCAFG00845025293	0	0	0	0
ENSCAFG00845025295	227	196	173	219
ENSCAFG00845025290	0	0	0	0
ENSCAFG00845025291	1171	1089	1070	1170
ENSCAFG00845025289	959	961	871	804
ENSCAFG00845025285	2	3	1	1
ENSCAFG00845025286	5	7	2	9
ENSCAFG00845025287	343	443	371	383
ENSCAFG00845025288	0	0	0	0
ENSCAFG00845025281	48	38	44	44
ENSCAFG00845025282	0	0	0	0
ENSCAFG00845025284	0	0	0	0
ENSCAFG00845025280	368	368	360	405
ENSCAFG00845013296	3131	3108	2797	2842
ENSCAFG00845013295	1211	1208	706	703
ENSCAFG00845013298	8	7	17	6

ENSCAFG00845013297	0	0	0	0
ENSCAFG00845013292	0	0	0	0
ENSCAFG00845013291	37	40	34	43
ENSCAFG00845013294	0	0	0	0
ENSCAFG00845013293	0	1	0	0
ENSCAFG00845013290	295	301	267	304
ENSCAFG00845025278	0	0	0	0
ENSCAFG00845025279	1	0	0	0
ENSCAFG00845025274	0	0	0	0
ENSCAFG00845025275	750	768	816	780
ENSCAFG00845025277	353	372	401	373
ENSCAFG00845025270	0	0	0	0
ENSCAFG00845025271	0	0	0	0
ENSCAFG00845025273	11118	10235	10379	10686
ENSCAFG00845013299	2088	1903	2282	2194
ENSCAFG00845013285	68	69	90	86
ENSCAFG00845013284	0	0	0	0
ENSCAFG00845013287	4	1	11	5
ENSCAFG00845013286	0	0	0	0
ENSCAFG00845013281	782	740	817	760
ENSCAFG00845013280	12	11	19	8
ENSCAFG00845013283	0	0	0	0
ENSCAFG00845013282	1281	1244	1249	1224
ENSCAFG00845025267	24	27	14	26
ENSCAFG00845025268	438	453	377	369
ENSCAFG00845025269	0	0	0	0
ENSCAFG00845025263	0	0	0	0
ENSCAFG00845025264	709	730	753	765
ENSCAFG00845025265	0	0	0	0
ENSCAFG00845025266	0	0	0	0
ENSCAFG00845025260	0	1	0	0
ENSCAFG00845025261	0	0	0	0
ENSCAFG00845025262	1433	1324	1226	1229
ENSCAFG00845013289	17	15	15	11
ENSCAFG00845013288	0	0	0	0
ENSCAFG00845003909	1	1	2	1
ENSCAFG00845003908	329	278	232	307
ENSCAFG00845003907	5	3	4	1
ENSCAFG00845003906	66	99	57	69
ENSCAFG00845003905	753	610	728	761
ENSCAFG00845003904	0	0	0	0

ENSCAFG00845003903	38	33	32	43
ENSCAFG00845003902	414	379	425	433
ENSCAFG00845003901	2300	2160	1910	1911
ENSCAFG00845003900	3	6	4	1
ENSCAFG00845003919	74	51	63	61
ENSCAFG00845003918	1670	1739	1677	1695
ENSCAFG00845003917	1373	1292	1163	1165
ENSCAFG00845003916	131	130	84	96
ENSCAFG00845003915	0	0	0	0
ENSCAFG00845003914	1191	1170	1249	1286
ENSCAFG00845003913	1097	959	966	985
ENSCAFG00845003912	0	0	0	0
ENSCAFG00845003911	14	3	7	4
ENSCAFG00845003910	289	261	238	250
ENSCAFG00845015850	185	182	177	172
ENSCAFG00845001209	1	1	1	3
ENSCAFG00845027839	1	0	0	1
ENSCAFG00845027838	0	0	2	2
ENSCAFG00845001207	2465	2442	2371	2529
ENSCAFG00845001208	483	469	517	474
ENSCAFG00845003869	0	0	0	0
ENSCAFG00845001205	85	69	74	79
ENSCAFG00845003868	99	132	126	113
ENSCAFG00845027835	0	0	0	0
ENSCAFG00845001206	33	19	18	22
ENSCAFG00845003867	6	2	8	1
ENSCAFG00845027834	1294	1191	1335	1469
ENSCAFG00845001203	0	0	0	0
ENSCAFG00845003866	2	2	1	0
ENSCAFG00845027837	17	15	17	12
ENSCAFG00845001204	0	0	0	0
ENSCAFG00845003865	8	1	0	1
ENSCAFG00845001201	1012	914	940	922
ENSCAFG00845003864	2228	2198	2387	2358
ENSCAFG00845027831	105	108	86	102
ENSCAFG00845001202	446	494	505	487
ENSCAFG00845003863	1	0	0	0
ENSCAFG00845027830	230	268	195	194
ENSCAFG00845003862	325	333	301	325
ENSCAFG00845027833	1377	1326	1347	1358
ENSCAFG00845001200	13	6	6	4

ENSCAFG00845003861	895	850	745	736
ENSCAFG00845027832	0	0	0	0
ENSCAFG00845003860	0	0	0	0
ENSCAFG00845015859	70	79	74	60
ENSCAFG00845015857	0	0	0	0
ENSCAFG00845015858	0	0	0	0
ENSCAFG00845015855	43	48	37	47
ENSCAFG00845015856	314	235	294	272
ENSCAFG00845015853	1298	1132	1180	1246
ENSCAFG00845015854	0	0	0	0
ENSCAFG00845015851	24	30	28	22
ENSCAFG00845015852	3097	2905	3053	3132
ENSCAFG00845027828	0	0	0	0
ENSCAFG00845027827	257	242	279	301
ENSCAFG00845001218	17	18	25	34
ENSCAFG00845001219	513	520	458	416
ENSCAFG00845027829	28	33	25	26
ENSCAFG00845001216	25	10	13	5
ENSCAFG00845003879	727	572	728	692
ENSCAFG00845027824	0	0	0	1
ENSCAFG00845001217	0	0	0	0
ENSCAFG00845003878	114	109	133	132
ENSCAFG00845027823	0	0	0	0
ENSCAFG00845001214	206	195	184	184
ENSCAFG00845003877	0	0	0	0
ENSCAFG00845027826	1577	1454	1668	1626
ENSCAFG00845001215	78	83	81	94
ENSCAFG00845003876	0	0	0	0
ENSCAFG00845027825	0	0	0	0
ENSCAFG00845001212	24	14	18	23
ENSCAFG00845003875	200	226	136	138
ENSCAFG00845027820	0	0	0	0
ENSCAFG00845001213	0	3	5	2
ENSCAFG00845003874	0	1	0	0
ENSCAFG00845001210	1	1	0	0
ENSCAFG00845003873	1008	926	892	976
ENSCAFG00845027822	0	0	0	0
ENSCAFG00845001211	2472	2328	2455	2485
ENSCAFG00845003872	6	5	5	11
ENSCAFG00845027821	0	0	0	0
ENSCAFG00845003871	0	1	0	0

ENSCAFG00845003870	706	618	669	669
ENSCAFG00845015848	13	16	26	16
ENSCAFG00845015849	13	10	6	10
ENSCAFG00845015846	479	499	456	499
ENSCAFG00845015847	4129	3895	3671	3619
ENSCAFG00845015844	1	0	1	0
ENSCAFG00845015845	857	872	917	885
ENSCAFG00845015842	54	55	14	21
ENSCAFG00845015843	111	88	135	106
ENSCAFG00845015840	1	0	0	0
ENSCAFG00845015841	1162	1151	1311	1260
ENSCAFG00845027817	5	2	1	2
ENSCAFG00845003849	0	1	0	0
ENSCAFG00845027816	0	3	2	3
ENSCAFG00845003848	1532	1380	1534	1673
ENSCAFG00845027819	306	312	302	280
ENSCAFG00845003847	3	8	4	5
ENSCAFG00845027818	154	112	139	142
ENSCAFG00845003846	0	1	2	0
ENSCAFG00845003845	0	0	0	0
ENSCAFG00845027812	902	838	951	974
ENSCAFG00845003844	356	337	351	336
ENSCAFG00845027815	0	0	0	0
ENSCAFG00845003843	169	176	189	147
ENSCAFG00845027814	0	0	0	2
ENSCAFG00845003842	227	230	238	282
ENSCAFG00845003841	1	1	4	4
ENSCAFG00845003840	0	0	0	0
ENSCAFG00845027811	693	696	597	641
ENSCAFG00845027810	0	1	2	1
ENSCAFG00845015839	0	1	1	0
ENSCAFG00845015837	1203	1267	1377	1473
ENSCAFG00845015838	0	0	0	0
ENSCAFG00845015835	12	13	9	7
ENSCAFG00845015836	0	0	0	0
ENSCAFG00845015833	9	9	7	11
ENSCAFG00845015834	1032	1054	1194	1263
ENSCAFG00845015831	50	63	48	46
ENSCAFG00845015832	1746	1609	1708	1798
ENSCAFG00845015830	7	2	4	8
ENSCAFG00845027809	0	0	0	0

ENSCAFG00845027806	488	511	486	490
ENSCAFG00845027805	0	0	0	0
ENSCAFG00845003859	968	1023	1055	1093
ENSCAFG00845027808	634	693	547	593
ENSCAFG00845003858	3	4	9	4
ENSCAFG00845027807	15	12	12	20
ENSCAFG00845003857	172	209	205	203
ENSCAFG00845027802	2	0	0	0
ENSCAFG00845003856	463	412	524	537
ENSCAFG00845027801	131	158	112	117
ENSCAFG00845003855	0	0	0	0
ENSCAFG00845027804	0	0	0	0
ENSCAFG00845003854	957	953	848	882
ENSCAFG00845027803	0	0	0	0
ENSCAFG00845003853	2	1	0	0
ENSCAFG00845003852	227	213	284	265
ENSCAFG00845003851	516	537	537	608
ENSCAFG00845027800	1251	1180	1444	1381
ENSCAFG00845003850	748	723	679	788
ENSCAFG00845015828	2	3	0	0
ENSCAFG00845015829	869	845	816	819
ENSCAFG00845015826	0	0	0	0
ENSCAFG00845015827	42	48	25	41
ENSCAFG00845015824	5	0	0	1
ENSCAFG00845015825	0	0	0	0
ENSCAFG00845015822	390	435	416	408
ENSCAFG00845015823	1	1	0	1
ENSCAFG00845015820	0	0	0	0
ENSCAFG00845015821	476	409	511	489
ENSCAFG00845003829	0	0	3	0
ENSCAFG00845003828	102	82	126	103
ENSCAFG00845003827	0	0	0	0
ENSCAFG00845003826	0	0	0	0
ENSCAFG00845003825	0	0	0	0
ENSCAFG00845003824	134	140	151	105
ENSCAFG00845003823	281	273	266	276
ENSCAFG00845003822	102	100	77	97
ENSCAFG00845003821	34	43	46	39
ENSCAFG00845003820	1	2	0	1
ENSCAFG00845015819	0	0	0	0
ENSCAFG00845015817	1875	1713	1343	1390

ENSCAFG00845015818	0	0	0	0
ENSCAFG00845015815	789	669	690	720
ENSCAFG00845015816	0	0	0	0
ENSCAFG00845015813	79	55	58	56
ENSCAFG00845015814	724	695	681	719
ENSCAFG00845015811	3	2	1	1
ENSCAFG00845015812	0	0	0	0
ENSCAFG00845015810	9	6	10	17
ENSCAFG00845003839	3	4	1	7
ENSCAFG00845003838	1555	1462	1513	1594
ENSCAFG00845003837	0	0	0	0
ENSCAFG00845003836	0	0	3	0
ENSCAFG00845003835	0	0	0	0
ENSCAFG00845003834	0	0	0	0
ENSCAFG00845003833	0	0	0	0
ENSCAFG00845003832	0	0	1	0
ENSCAFG00845003831	1	0	0	1
ENSCAFG00845003830	0	0	0	0
ENSCAFG00845015808	0	0	0	0
ENSCAFG00845015809	0	0	1	1
ENSCAFG00845015806	0	0	0	0
ENSCAFG00845015807	1	0	0	1
ENSCAFG00845015804	10	12	11	10
ENSCAFG00845015805	468	390	458	417
ENSCAFG00845015802	0	0	0	0
ENSCAFG00845015803	0	0	0	1
ENSCAFG00845015800	1	1	0	0
ENSCAFG00845015801	416	414	440	401
ENSCAFG00845003809	533	515	523	482
ENSCAFG00845003808	950	952	949	903
ENSCAFG00845003807	3714	3396	3915	3945
ENSCAFG00845003806	1330	1320	1403	1408
ENSCAFG00845003805	0	0	3	3
ENSCAFG00845003804	0	0	0	0
ENSCAFG00845003803	0	0	0	0
ENSCAFG00845003802	0	4	2	4
ENSCAFG00845003801	21	29	24	20
ENSCAFG00845003800	1929	1919	1822	1674
ENSCAFG00845003819	0	0	0	0
ENSCAFG00845003818	1	2	3	0
ENSCAFG00845003817	0	0	0	1

ENSCAFG00845003816	0	0	0	0
ENSCAFG00845003815	89	89	130	118
ENSCAFG00845003814	211	195	190	157
ENSCAFG00845003813	0	0	0	0
ENSCAFG00845003812	11	15	8	16
ENSCAFG00845003811	0	0	0	0
ENSCAFG00845003810	3	1	3	2
ENSCAFG00845013274	0	0	0	0
ENSCAFG00845013273	0	0	0	0
ENSCAFG00845013276	2	1	0	0
ENSCAFG00845013275	260	217	275	245
ENSCAFG00845013270	2	4	1	2
ENSCAFG00845013272	0	0	0	0
ENSCAFG00845013271	756	724	684	689
ENSCAFG00845001289	0	1	0	0
ENSCAFG00845025256	12	7	5	9
ENSCAFG00845025257	8	8	9	8
ENSCAFG00845001287	200	171	199	194
ENSCAFG00845025258	0	2	2	2
ENSCAFG00845001288	0	0	1	0
ENSCAFG00845025259	0	0	0	0
ENSCAFG00845001285	707	634	670	728
ENSCAFG00845001286	691	565	703	691
ENSCAFG00845025253	0	0	0	0
ENSCAFG00845001283	0	0	0	0
ENSCAFG00845025254	12	22	26	12
ENSCAFG00845001284	4	6	6	8
ENSCAFG00845025255	560	578	557	550
ENSCAFG00845001281	153	152	124	134
ENSCAFG00845001282	104	90	69	77
ENSCAFG00845025250	0	0	0	0
ENSCAFG00845001280	0	0	0	0
ENSCAFG00845013278	0	0	0	0
ENSCAFG00845013277	38	41	45	45
ENSCAFG00845013279	1326	1189	1252	1221
ENSCAFG00845013263	0	0	0	0
ENSCAFG00845013262	0	0	0	0
ENSCAFG00845013265	1024	924	1038	1199
ENSCAFG00845013264	730	624	668	717
ENSCAFG00845013261	41	31	40	32
ENSCAFG00845013260	0	0	0	0

ENSCAFG00845025249	0	0	1	0
ENSCAFG00845025245	1228	1151	1170	1130
ENSCAFG00845025246	12	14	15	27
ENSCAFG00845001298	0	0	0	0
ENSCAFG00845025247	362	385	377	317
ENSCAFG00845001299	0	0	0	0
ENSCAFG00845001296	1294	1198	1260	1307
ENSCAFG00845025241	470	386	382	393
ENSCAFG00845001297	1369	1329	1362	1391
ENSCAFG00845025242	58	42	60	74
ENSCAFG00845001294	3	3	8	5
ENSCAFG00845025243	4	3	8	5
ENSCAFG00845001295	1	2	1	2
ENSCAFG00845025244	0	0	0	0
ENSCAFG00845001292	0	0	0	0
ENSCAFG00845001293	673	673	697	708
ENSCAFG00845001290	2542	2555	2226	2357
ENSCAFG00845001291	565	519	558	517
ENSCAFG00845025240	0	0	0	0
ENSCAFG00845013267	0	0	0	0
ENSCAFG00845013266	0	0	0	0
ENSCAFG00845013269	0	0	0	0
ENSCAFG00845013268	8989	8674	8068	8188
ENSCAFG00845013252	11	6	17	14
ENSCAFG00845013251	384	358	390	434
ENSCAFG00845013254	7	3	2	1
ENSCAFG00845013253	10	14	21	9
ENSCAFG00845013250	179	183	214	202
ENSCAFG00845025238	114	99	110	119
ENSCAFG00845025239	481	420	486	489
ENSCAFG00845001269	0	0	0	0
ENSCAFG00845001267	0	0	0	0
ENSCAFG00845025234	5	8	2	3
ENSCAFG00845027897	0	0	0	0
ENSCAFG00845001268	1514	1458	1308	1401
ENSCAFG00845025235	0	0	0	0
ENSCAFG00845027896	0	0	0	0
ENSCAFG00845001265	0	0	0	0
ENSCAFG00845025236	0	0	0	0
ENSCAFG00845027899	495	402	476	452
ENSCAFG00845001266	0	0	0	0

ENSCAFG00845025237	129	98	106	110
ENSCAFG00845027898	4229	4171	3864	4015
ENSCAFG00845001263	1165	1137	1059	948
ENSCAFG00845025230	0	0	0	0
ENSCAFG00845027893	6934	6783	6983	6927
ENSCAFG00845001264	198	144	204	218
ENSCAFG00845027892	0	5	2	3
ENSCAFG00845001261	73	80	64	51
ENSCAFG00845025232	0	0	0	0
ENSCAFG00845027895	4	5	4	9
ENSCAFG00845001262	3685	3519	3574	3401
ENSCAFG00845025233	0	1	1	0
ENSCAFG00845027894	0	0	0	0
ENSCAFG00845001260	0	0	0	0
ENSCAFG00845027891	5	3	7	4
ENSCAFG00845027890	35	16	24	21
ENSCAFG00845013259	0	0	0	0
ENSCAFG00845013256	774	749	733	697
ENSCAFG00845013255	1296	1201	1312	1334
ENSCAFG00845013258	0	0	0	0
ENSCAFG00845013257	25	43	37	47
ENSCAFG00845013241	0	0	0	0
ENSCAFG00845013240	0	0	0	0
ENSCAFG00845013243	29	13	6	9
ENSCAFG00845013242	21	16	17	18
ENSCAFG00845025227	14383	14180	14467	14823
ENSCAFG00845025228	0	0	0	0
ENSCAFG00845027889	0	0	0	0
ENSCAFG00845025229	1360	1231	1115	1135
ENSCAFG00845001278	0	0	0	0
ENSCAFG00845027886	2	0	0	0
ENSCAFG00845001279	0	0	0	0
ENSCAFG00845025224	6	4	4	9
ENSCAFG00845027885	3	3	4	3
ENSCAFG00845001276	452	458	434	422
ENSCAFG00845025225	7	2	1	2
ENSCAFG00845027888	0	0	0	0
ENSCAFG00845001277	0	0	0	1
ENSCAFG00845025226	145	160	144	145
ENSCAFG00845027887	1501	1361	1575	1573
ENSCAFG00845001274	2172	2261	2221	2252

ENSCAFG00845027882	346	346	316	364
ENSCAFG00845001275	0	2	0	0
ENSCAFG00845025220	445	476	378	360
ENSCAFG00845027881	1231	1123	1288	1179
ENSCAFG00845001272	9	13	4	12
ENSCAFG00845025221	1	0	0	0
ENSCAFG00845001273	81	129	93	110
ENSCAFG00845025222	2818	2660	2903	2786
ENSCAFG00845027883	0	0	2	0
ENSCAFG00845001270	47	68	61	101
ENSCAFG00845001271	992	883	997	982
ENSCAFG00845027880	733	735	716	749
ENSCAFG00845013249	18	16	31	13
ENSCAFG00845013248	0	0	0	0
ENSCAFG00845013245	111	144	98	84
ENSCAFG00845013244	4425	4451	3784	4060
ENSCAFG00845013247	0	0	0	0
ENSCAFG00845013246	20	23	16	13
ENSCAFG00845013230	0	0	0	0
ENSCAFG00845015893	0	0	0	0
ENSCAFG00845015894	14	10	6	12
ENSCAFG00845013232	0	0	0	0
ENSCAFG00845015891	257	244	232	312
ENSCAFG00845013231	0	0	0	0
ENSCAFG00845015892	0	0	0	0
ENSCAFG00845015890	120	103	111	90
ENSCAFG00845001249	305	254	352	313
ENSCAFG00845025216	229	161	205	232
ENSCAFG00845027879	0	0	0	0
ENSCAFG00845025217	9	13	10	8
ENSCAFG00845027878	28	21	10	15
ENSCAFG00845001247	0	0	0	0
ENSCAFG00845025218	0	0	0	0
ENSCAFG00845001248	1728	1660	1699	1689
ENSCAFG00845025219	0	0	0	0
ENSCAFG00845001245	598	540	566	576
ENSCAFG00845025212	2	2	4	4
ENSCAFG00845027875	1	3	0	1
ENSCAFG00845001246	0	0	0	2
ENSCAFG00845025213	0	1	4	2
ENSCAFG00845027874	7	4	9	5

ENSCAFG00845001243	649	612	593	549
ENSCAFG00845025214	3093	3012	2735	2762
ENSCAFG00845027877	14	11	11	26
ENSCAFG00845001244	5	1	0	2
ENSCAFG00845025215	0	0	0	0
ENSCAFG00845027876	0	0	0	0
ENSCAFG00845001241	1388	1375	1249	1266
ENSCAFG00845027871	0	0	0	0
ENSCAFG00845001242	1801	1711	1494	1588
ENSCAFG00845027870	641	628	643	760
ENSCAFG00845025210	6	5	9	5
ENSCAFG00845027873	29	21	5	15
ENSCAFG00845001240	12	23	13	7
ENSCAFG00845025211	429	423	376	365
ENSCAFG00845027872	0	0	0	0
ENSCAFG00845013238	0	0	0	1
ENSCAFG00845013237	0	0	0	0
ENSCAFG00845015899	2734	2640	2370	2494
ENSCAFG00845013239	0	0	0	0
ENSCAFG00845013234	0	0	0	0
ENSCAFG00845015897	346	335	314	316
ENSCAFG00845013233	660	681	572	563
ENSCAFG00845015898	1048	1089	1044	1033
ENSCAFG00845013236	0	0	0	0
ENSCAFG00845015895	0	0	0	0
ENSCAFG00845013235	39	50	44	36
ENSCAFG00845015896	0	0	0	1
ENSCAFG00845015882	0	0	0	0
ENSCAFG00845015883	0	0	0	0
ENSCAFG00845013221	334	304	310	277
ENSCAFG00845015880	0	0	0	0
ENSCAFG00845013220	383	416	289	349
ENSCAFG00845015881	1492	1520	1481	1579
ENSCAFG00845025209	4700	4624	4666	4542
ENSCAFG00845025205	609	707	624	570
ENSCAFG00845027868	490	519	508	543
ENSCAFG00845001258	1503	1588	1648	1597
ENSCAFG00845001259	7	5	7	6
ENSCAFG00845027869	7	3	6	8
ENSCAFG00845001256	0	0	0	0
ENSCAFG00845025201	0	0	0	0

ENSCAFG00845027864	1321	1375	1253	1176
ENSCAFG00845001257	1449	1359	1357	1443
ENSCAFG00845025202	11	12	16	12
ENSCAFG00845027863	2	0	0	0
ENSCAFG00845001254	1	2	9	0
ENSCAFG00845025203	0	0	0	0
ENSCAFG00845027866	0	0	0	0
ENSCAFG00845001255	1308	1214	1233	1370
ENSCAFG00845027865	5	1	2	3
ENSCAFG00845001252	1	1	2	0
ENSCAFG00845027860	1	0	0	0
ENSCAFG00845001253	0	0	4	0
ENSCAFG00845001250	11	4	11	11
ENSCAFG00845027862	15	12	15	15
ENSCAFG00845001251	462	463	487	473
ENSCAFG00845025200	0	0	0	0
ENSCAFG00845027861	0	0	0	0
ENSCAFG00845013227	0	0	0	0
ENSCAFG00845013226	955	959	1034	1010
ENSCAFG00845013229	687	704	626	671
ENSCAFG00845015888	28	24	19	21
ENSCAFG00845013228	64	57	78	57
ENSCAFG00845015889	2439	2376	2499	2383
ENSCAFG00845013223	0	0	0	0
ENSCAFG00845015886	2332	2248	2160	2196
ENSCAFG00845013222	685	609	732	789
ENSCAFG00845015887	698	652	611	609
ENSCAFG00845013225	446	385	389	413
ENSCAFG00845015884	0	0	0	0
ENSCAFG00845013224	596	532	547	474
ENSCAFG00845015885	941	901	970	971
ENSCAFG00845015871	41	52	25	24
ENSCAFG00845015872	0	0	0	0
ENSCAFG00845013210	44	50	23	47
ENSCAFG00845015870	0	0	0	0
ENSCAFG00845001229	0	0	0	0
ENSCAFG00845001227	140	138	157	145
ENSCAFG00845027857	0	0	0	0
ENSCAFG00845001228	41	34	22	43
ENSCAFG00845003889	0	0	0	3
ENSCAFG00845027856	0	0	0	0

ENSCAFG00845001225	0	0	0	0
ENSCAFG00845003888	1144	1090	959	965
ENSCAFG00845027859	110	133	93	123
ENSCAFG00845001226	333	313	382	419
ENSCAFG00845003887	3	7	3	11
ENSCAFG00845027858	1665	1608	1442	1417
ENSCAFG00845001223	0	0	0	0
ENSCAFG00845003886	11	8	6	3
ENSCAFG00845027853	1868	1816	2063	1937
ENSCAFG00845001224	3140	3081	2993	3265
ENSCAFG00845003885	515	478	454	521
ENSCAFG00845027852	0	0	0	0
ENSCAFG00845001221	0	0	0	0
ENSCAFG00845003884	1231	1171	1159	1184
ENSCAFG00845027855	3	1	3	7
ENSCAFG00845001222	0	0	0	0
ENSCAFG00845003883	86	79	65	47
ENSCAFG00845027854	0	0	1	0
ENSCAFG00845003882	0	0	0	0
ENSCAFG00845001220	485	466	481	494
ENSCAFG00845003881	0	0	0	0
ENSCAFG00845003880	1563	1479	1721	1683
ENSCAFG00845027851	0	0	0	0
ENSCAFG00845027850	0	0	0	0
ENSCAFG00845013219	0	2	2	0
ENSCAFG00845013216	1668	1661	1632	1710
ENSCAFG00845015879	780	754	729	682
ENSCAFG00845013215	294	320	284	313
ENSCAFG00845013218	465	494	481	455
ENSCAFG00845015877	4	3	2	1
ENSCAFG00845013217	0	0	0	0
ENSCAFG00845015878	910	876	839	774
ENSCAFG00845013212	17	10	12	15
ENSCAFG00845015875	0	0	0	0
ENSCAFG00845013211	2966	2906	2817	2908
ENSCAFG00845015876	474	525	422	443
ENSCAFG00845013214	1648	1619	1568	1712
ENSCAFG00845015873	253	197	220	255
ENSCAFG00845013213	1170	1150	1136	1229
ENSCAFG00845015874	1	11	5	4
ENSCAFG00845015860	0	0	1	0

ENSCAFG00845015861	569	495	519	526
ENSCAFG00845027849	453	473	490	458
ENSCAFG00845001238	217	198	230	211
ENSCAFG00845027846	2	1	0	2
ENSCAFG00845001239	84	73	66	56
ENSCAFG00845027845	0	0	0	0
ENSCAFG00845001236	0	0	0	0
ENSCAFG00845003899	0	0	0	0
ENSCAFG00845027848	0	1	0	0
ENSCAFG00845001237	0	0	0	0
ENSCAFG00845003898	1890	1709	1864	1929
ENSCAFG00845027847	3438	3213	3111	3224
ENSCAFG00845001234	0	0	0	0
ENSCAFG00845003897	12	21	16	8
ENSCAFG00845027842	5	8	4	12
ENSCAFG00845001235	79	98	61	78
ENSCAFG00845003896	15	8	5	13
ENSCAFG00845027841	1	0	0	1
ENSCAFG00845001232	1	1	1	0
ENSCAFG00845003895	20	25	41	28
ENSCAFG00845027844	186	184	192	125
ENSCAFG00845001233	0	0	0	0
ENSCAFG00845003894	1009	928	992	1034
ENSCAFG00845027843	22	29	29	13
ENSCAFG00845001230	329	433	371	376
ENSCAFG00845003893	14	12	21	15
ENSCAFG00845001231	678	651	557	586
ENSCAFG00845003892	0	4	1	6
ENSCAFG00845003891	293	362	339	291
ENSCAFG00845027840	22	29	23	22
ENSCAFG00845003890	0	0	0	0
ENSCAFG00845013209	124	114	106	78
ENSCAFG00845013208	0	0	0	0
ENSCAFG00845013205	548	543	558	573
ENSCAFG00845015868	24	20	24	18
ENSCAFG00845013204	0	0	0	0
ENSCAFG00845015869	0	0	0	0
ENSCAFG00845013207	569	583	652	674
ENSCAFG00845015866	0	0	0	0
ENSCAFG00845013206	3	1	1	0
ENSCAFG00845015867	0	0	0	0

ENSCAFG00845013201	0	0	0	0
ENSCAFG00845015864	0	0	0	0
ENSCAFG00845013200	0	0	0	0
ENSCAFG00845015865	15	17	7	7
ENSCAFG00845013203	1702	1783	1078	1135
ENSCAFG00845015862	0	0	0	0
ENSCAFG00845013202	173	132	146	153
ENSCAFG00845015863	0	0	0	0
ENSCAFG00845015178	581	643	610	593
ENSCAFG00845015179	0	0	0	0
ENSCAFG00845015176	0	0	0	0
ENSCAFG00845015177	60	51	67	51
ENSCAFG00845015174	0	0	0	0
ENSCAFG00845015175	581	548	496	574
ENSCAFG00845015172	0	0	0	0
ENSCAFG00845015173	2757	2570	2553	2563
ENSCAFG00845015170	849	830	846	852
ENSCAFG00845015171	2292	2303	2122	2281
ENSCAFG00845027159	2	2	7	3
ENSCAFG00845003189	0	0	0	0
ENSCAFG00845027156	1	5	0	4
ENSCAFG00845003188	119	126	210	198
ENSCAFG00845027155	0	0	0	0
ENSCAFG00845003187	1653	1496	1535	1622
ENSCAFG00845027158	0	0	0	0
ENSCAFG00845003186	129	147	108	165
ENSCAFG00845027157	0	0	0	0
ENSCAFG00845003185	26	27	20	19
ENSCAFG00845027152	0	0	0	0
ENSCAFG00845003184	0	0	0	0
ENSCAFG00845027151	1	0	0	1
ENSCAFG00845003183	0	0	0	0
ENSCAFG00845027154	918	895	773	886
ENSCAFG00845003182	2	0	1	0
ENSCAFG00845027153	0	0	0	0
ENSCAFG00845003181	0	0	0	0
ENSCAFG00845003180	0	0	0	0
ENSCAFG00845027150	250	242	254	244
ENSCAFG00845015167	0	0	0	0
ENSCAFG00845015168	329	313	319	299
ENSCAFG00845015165	0	0	0	0

ENSCAFG00845015166	0	0	0	0
ENSCAFG00845015163	0	0	0	0
ENSCAFG00845015164	0	0	0	0
ENSCAFG00845015161	2077	2066	1993	2142
ENSCAFG00845015162	0	0	0	0
ENSCAFG00845015160	1	0	0	0
ENSCAFG00845027149	87	74	76	44
ENSCAFG00845027145	8	1	1	6
ENSCAFG00845003199	0	0	0	0
ENSCAFG00845027144	2044	1963	2039	1965
ENSCAFG00845003198	0	0	0	0
ENSCAFG00845027147	32	40	16	16
ENSCAFG00845003197	0	1	0	0
ENSCAFG00845027146	0	0	0	0
ENSCAFG00845003196	0	0	0	0
ENSCAFG00845027141	0	1	0	2
ENSCAFG00845003195	423	453	396	435
ENSCAFG00845027140	0	0	0	0
ENSCAFG00845003194	3	2	2	0
ENSCAFG00845027143	391	379	386	366
ENSCAFG00845003193	0	0	0	0
ENSCAFG00845027142	0	0	0	0
ENSCAFG00845003192	0	0	0	0
ENSCAFG00845003191	844	801	841	863
ENSCAFG00845003190	435	367	392	386
ENSCAFG00845015169	632	605	597	693
ENSCAFG00845015156	1	8	6	4
ENSCAFG00845015157	0	0	0	0
ENSCAFG00845015154	165	132	173	169
ENSCAFG00845015155	21	4	12	14
ENSCAFG00845015152	0	0	0	0
ENSCAFG00845015153	1632	1388	1474	1413
ENSCAFG00845015150	92	136	134	140
ENSCAFG00845015151	0	0	0	0
ENSCAFG00845027138	1657	1464	1623	1626
ENSCAFG00845027137	781	707	715	702
ENSCAFG00845003169	0	0	0	0
ENSCAFG00845029799	1145	1134	1041	999
ENSCAFG00845003168	0	0	0	0
ENSCAFG00845027139	0	0	0	0
ENSCAFG00845003167	0	0	0	0

ENSCAFG00845027134	0	0	0	0
ENSCAFG00845029797	0	0	0	0
ENSCAFG00845003166	0	0	0	0
ENSCAFG00845027133	1	0	0	0
ENSCAFG00845029798	8	5	10	5
ENSCAFG00845003165	0	0	0	0
ENSCAFG00845027136	21	23	22	26
ENSCAFG00845029795	1568	1493	1300	1398
ENSCAFG00845003164	890	774	887	851
ENSCAFG00845029796	3	0	0	0
ENSCAFG00845003163	0	0	0	0
ENSCAFG00845027130	0	0	0	0
ENSCAFG00845029793	1352	1356	1324	1338
ENSCAFG00845003162	1428	1406	1423	1475
ENSCAFG00845029794	1	0	0	0
ENSCAFG00845003161	0	0	0	1
ENSCAFG00845027132	0	0	0	0
ENSCAFG00845029791	1276	1289	1304	1262
ENSCAFG00845003160	0	0	0	0
ENSCAFG00845027131	94	78	94	86
ENSCAFG00845029792	504	486	446	432
ENSCAFG00845029790	0	0	0	0
ENSCAFG00845015158	0	0	0	0
ENSCAFG00845015159	1	2	7	5
ENSCAFG00845015145	362	368	393	310
ENSCAFG00845015146	0	0	0	0
ENSCAFG00845015143	0	0	0	0
ENSCAFG00845015144	1	0	0	0
ENSCAFG00845015141	0	0	0	0
ENSCAFG00845015142	716	640	654	662
ENSCAFG00845015140	2546	2673	2478	2681
ENSCAFG00845027127	0	0	0	0
ENSCAFG00845027126	1504	1464	1646	1547
ENSCAFG00845027129	0	0	0	0
ENSCAFG00845029788	561	631	565	527
ENSCAFG00845003179	0	0	0	0
ENSCAFG00845027128	2	1	7	6
ENSCAFG00845029789	237	202	233	198
ENSCAFG00845003178	0	0	0	0
ENSCAFG00845027123	747	811	677	713
ENSCAFG00845029786	17	33	12	18

ENSCAFG00845003177	532	506	534	628
ENSCAFG00845027122	3	2	0	0
ENSCAFG00845029787	114	119	113	103
ENSCAFG00845003176	0	0	0	0
ENSCAFG00845027125	225	174	242	242
ENSCAFG00845029784	16	19	25	21
ENSCAFG00845003175	0	0	0	0
ENSCAFG00845029785	0	0	0	0
ENSCAFG00845003174	10	8	9	7
ENSCAFG00845029782	11	18	19	34
ENSCAFG00845003173	0	0	0	0
ENSCAFG00845029783	0	0	0	0
ENSCAFG00845003172	1921	1839	1785	1884
ENSCAFG00845027121	1572	1674	1702	1732
ENSCAFG00845029780	0	0	0	0
ENSCAFG00845003171	1804	1785	1787	1867
ENSCAFG00845027120	344	325	343	397
ENSCAFG00845029781	0	5	1	3
ENSCAFG00845003170	0	0	0	0
ENSCAFG00845015149	509	469	640	562
ENSCAFG00845015147	751	742	791	810
ENSCAFG00845015148	0	0	0	0
ENSCAFG00845015134	0	0	0	0
ENSCAFG00845017797	0	0	0	0
ENSCAFG00845015135	22	23	18	1
ENSCAFG00845017796	2	2	3	4
ENSCAFG00845015132	0	1	5	3
ENSCAFG00845017795	10	5	4	12
ENSCAFG00845015133	2	0	0	0
ENSCAFG00845017794	0	0	0	0
ENSCAFG00845015130	1856	1678	1689	1803
ENSCAFG00845017793	1	0	1	0
ENSCAFG00845015131	1176	1170	1172	1185
ENSCAFG00845017792	996	1027	869	911
ENSCAFG00845017791	7	7	2	4
ENSCAFG00845017790	1111	1143	1089	1172
ENSCAFG00845027119	5	6	6	1
ENSCAFG00845003149	154	135	108	133
ENSCAFG00845027116	2	4	0	4
ENSCAFG00845029779	2	0	2	0
ENSCAFG00845003148	0	0	0	0

ENSCAFG00845027115	986	966	882	976
ENSCAFG00845003147	690	640	639	725
ENSCAFG00845027118	0	1	0	2
ENSCAFG00845029777	0	0	0	0
ENSCAFG00845003146	0	0	0	0
ENSCAFG00845027117	0	0	0	0
ENSCAFG00845029778	0	0	0	0
ENSCAFG00845003145	7	4	3	5
ENSCAFG00845027112	0	0	0	0
ENSCAFG00845029775	0	0	0	0
ENSCAFG00845003144	207	201	211	168
ENSCAFG00845027111	0	0	0	0
ENSCAFG00845029776	0	1	0	0
ENSCAFG00845003143	372	386	466	463
ENSCAFG00845027114	0	0	0	0
ENSCAFG00845029773	1761	1571	1668	1723
ENSCAFG00845003142	0	0	0	0
ENSCAFG00845027113	643	648	761	741
ENSCAFG00845029774	3107	2859	3149	3192
ENSCAFG00845003141	5	4	5	0
ENSCAFG00845029771	0	0	0	0
ENSCAFG00845003140	219	227	211	217
ENSCAFG00845029772	0	7	8	1
ENSCAFG00845027110	0	0	0	0
ENSCAFG00845029770	2	0	0	0
ENSCAFG00845015138	5055	5060	4835	4978
ENSCAFG00845015139	205	222	217	234
ENSCAFG00845015136	3	1	0	1
ENSCAFG00845017799	0	0	0	0
ENSCAFG00845015137	0	0	0	0
ENSCAFG00845017798	8	8	1	5
ENSCAFG00845015123	38	24	19	23
ENSCAFG00845017786	66	46	39	62
ENSCAFG00845015124	346	341	235	255
ENSCAFG00845017785	7	4	6	3
ENSCAFG00845015121	0	0	0	0
ENSCAFG00845017784	0	0	0	0
ENSCAFG00845015122	353	322	339	288
ENSCAFG00845017783	4	1	0	0
ENSCAFG00845017782	73	127	106	113
ENSCAFG00845015120	479	434	502	502

ENSCAFG00845017781	0	0	0	0
ENSCAFG00845017780	14	9	7	8
ENSCAFG00845027109	0	0	0	0
ENSCAFG00845027108	1	8	4	3
ENSCAFG00845027105	0	0	0	0
ENSCAFG00845029768	568	554	579	599
ENSCAFG00845003159	0	0	0	0
ENSCAFG00845027104	0	0	0	0
ENSCAFG00845029769	19	9	15	16
ENSCAFG00845003158	0	1	2	2
ENSCAFG00845027107	0	0	0	0
ENSCAFG00845029766	0	0	0	0
ENSCAFG00845003157	943	952	901	1002
ENSCAFG00845027106	735	722	738	693
ENSCAFG00845029767	0	0	0	0
ENSCAFG00845003156	1	1	6	0
ENSCAFG00845027101	2	6	2	4
ENSCAFG00845029764	732	619	676	637
ENSCAFG00845003155	4	4	5	5
ENSCAFG00845027100	51	55	59	50
ENSCAFG00845029765	8	9	5	10
ENSCAFG00845003154	3	1	5	6
ENSCAFG00845027103	50	47	103	69
ENSCAFG00845029762	449	437	409	391
ENSCAFG00845003153	0	0	0	0
ENSCAFG00845027102	40	27	18	23
ENSCAFG00845029763	0	0	0	0
ENSCAFG00845003152	0	0	0	0
ENSCAFG00845029760	1	8	11	2
ENSCAFG00845003151	168	161	157	203
ENSCAFG00845029761	0	0	1	0
ENSCAFG00845003150	0	0	0	0
ENSCAFG00845015129	11	10	8	8
ENSCAFG00845015127	0	0	0	0
ENSCAFG00845015128	4	8	6	2
ENSCAFG00845017789	0	0	0	0
ENSCAFG00845015125	649	551	606	567
ENSCAFG00845017788	0	0	0	0
ENSCAFG00845015126	0	0	0	0
ENSCAFG00845017787	1079	1103	1062	1164
ENSCAFG00845015112	2	0	0	1

ENSCAFG00845017775	418	424	388	411
ENSCAFG00845015113	0	0	0	0
ENSCAFG00845017774	71	53	45	54
ENSCAFG00845015110	6	5	5	7
ENSCAFG00845017773	0	0	0	0
ENSCAFG00845015111	0	0	0	0
ENSCAFG00845017772	0	0	0	0
ENSCAFG00845017771	0	0	0	0
ENSCAFG00845017770	87	84	8	5
ENSCAFG00845005790	4	4	2	6
ENSCAFG00845003129	994	911	802	821
ENSCAFG00845005791	4	1	4	9
ENSCAFG00845029759	20	10	13	14
ENSCAFG00845003128	0	0	0	0
ENSCAFG00845005792	35	33	28	22
ENSCAFG00845003127	0	0	0	0
ENSCAFG00845005793	1780	1638	1640	1595
ENSCAFG00845029757	548	528	547	531
ENSCAFG00845003126	54	30	51	36
ENSCAFG00845005794	0	0	0	0
ENSCAFG00845029758	1365	1257	1417	1312
ENSCAFG00845003125	0	0	0	0
ENSCAFG00845005795	15	11	23	32
ENSCAFG00845029755	1	1	0	0
ENSCAFG00845003124	0	0	0	0
ENSCAFG00845005796	551	481	490	491
ENSCAFG00845029756	0	0	0	0
ENSCAFG00845003123	3	0	5	2
ENSCAFG00845005797	79	73	102	82
ENSCAFG00845029753	1	0	1	4
ENSCAFG00845003122	0	0	0	0
ENSCAFG00845005798	0	0	0	0
ENSCAFG00845029754	0	0	0	0
ENSCAFG00845003121	637	553	534	575
ENSCAFG00845005799	128	136	105	105
ENSCAFG00845029751	1	0	0	0
ENSCAFG00845003120	518	480	469	497
ENSCAFG00845029752	437	451	398	326
ENSCAFG00845029750	2255	2174	1831	2068
ENSCAFG00845015118	4	5	1	2
ENSCAFG00845015119	0	0	0	0

ENSCAFG00845015116	1	6	0	2
ENSCAFG00845017779	4	1	2	1
ENSCAFG00845015117	0	0	0	0
ENSCAFG00845017778	0	0	0	0
ENSCAFG00845015114	0	0	0	0
ENSCAFG00845017777	0	0	0	0
ENSCAFG00845015115	0	0	0	0
ENSCAFG00845017776	870	820	736	748
ENSCAFG00845015101	1	1	0	0
ENSCAFG00845017764	328	323	271	309
ENSCAFG00845015102	0	0	0	0
ENSCAFG00845017763	0	0	0	0
ENSCAFG00845017762	0	0	0	0
ENSCAFG00845015100	3123	3161	3132	2932
ENSCAFG00845017761	0	0	0	0
ENSCAFG00845017760	41	43	18	41
ENSCAFG00845005780	41	48	37	53
ENSCAFG00845029748	5	4	2	9
ENSCAFG00845003139	0	0	0	0
ENSCAFG00845005781	5	13	9	13
ENSCAFG00845029749	10	8	4	5
ENSCAFG00845003138	877	850	775	780
ENSCAFG00845005782	0	0	0	0
ENSCAFG00845029746	1167	1184	1110	1111
ENSCAFG00845003137	1347	1313	1211	1220
ENSCAFG00845005783	14	14	18	23
ENSCAFG00845029747	10	17	5	5
ENSCAFG00845003136	641	638	621	652
ENSCAFG00845005784	2	1	0	2
ENSCAFG00845029744	377	352	426	465
ENSCAFG00845003135	0	0	0	0
ENSCAFG00845005785	231	266	215	220
ENSCAFG00845029745	0	0	0	0
ENSCAFG00845003134	35	37	45	45
ENSCAFG00845005786	51	29	36	65
ENSCAFG00845029742	122	100	170	143
ENSCAFG00845003133	59	59	53	44
ENSCAFG00845005787	0	0	0	0
ENSCAFG00845029743	45	42	49	45
ENSCAFG00845003132	91	68	34	41
ENSCAFG00845005788	0	0	0	0

ENSCAFG00845029740	23	28	21	39
ENSCAFG00845003131	0	0	0	0
ENSCAFG00845005789	3	0	1	1
ENSCAFG00845029741	1	7	2	4
ENSCAFG00845003130	0	0	2	0
ENSCAFG00845015109	0	0	2	0
ENSCAFG00845015107	0	0	0	0
ENSCAFG00845015108	0	0	0	0
ENSCAFG00845017769	18	17	31	34
ENSCAFG00845015105	0	0	0	0
ENSCAFG00845017768	0	0	0	0
ENSCAFG00845015106	0	0	0	0
ENSCAFG00845017767	1023	1095	884	819
ENSCAFG00845015103	1210	1061	1071	989
ENSCAFG00845017766	399	391	426	396
ENSCAFG00845015104	519	505	478	539
ENSCAFG00845030728	311	290	194	252
ENSCAFG00845030729	1111	1131	1144	1242
ENSCAFG00845030731	167	215	166	153
ENSCAFG00845030732	0	0	5	0
ENSCAFG00845030733	6	8	7	12
ENSCAFG00845030734	322	314	271	290
ENSCAFG00845030735	9	4	7	3
ENSCAFG00845030736	147	190	162	156
ENSCAFG00845030737	0	0	0	0
ENSCAFG00845030738	548	573	550	560
ENSCAFG00845030730	787	762	684	828
ENSCAFG00845030739	0	0	1	0
ENSCAFG00845030742	0	0	0	1
ENSCAFG00845030743	1	0	0	2
ENSCAFG00845030744	2	5	7	3
ENSCAFG00845030745	1309	1216	1139	1210
ENSCAFG00845030746	0	0	0	0
ENSCAFG00845030747	0	0	0	0
ENSCAFG00845030748	102	78	84	105
ENSCAFG00845030749	20	34	35	26
ENSCAFG00845030740	402	394	317	397
ENSCAFG00845030741	1	0	5	2
ENSCAFG00845030753	2235	2158	1995	2014
ENSCAFG00845030754	310	290	331	328
ENSCAFG00845030755	46	30	33	34

ENSCAFG00845030756	181	155	142	136
ENSCAFG00845030757	0	0	0	0
ENSCAFG00845030758	26	10	11	4
ENSCAFG00845030759	143	137	194	179
ENSCAFG00845030750	2009	2018	1856	1854
ENSCAFG00845030751	273	249	259	274
ENSCAFG00845030752	0	0	0	0
ENSCAFG00845030764	0	0	0	0
ENSCAFG00845030765	1272	1170	1181	1143
ENSCAFG00845030766	90	68	60	76
ENSCAFG00845030767	162	165	186	186
ENSCAFG00845030768	1273	1214	1254	1204
ENSCAFG00845030769	2004	2047	2526	2636
ENSCAFG00845030760	15	2	8	10
ENSCAFG00845030761	399	328	361	387
ENSCAFG00845030762	4	0	4	0
ENSCAFG00845030763	67	88	106	101
ENSCAFG00845030775	0	1	0	0
ENSCAFG00845027199	0	0	3	3
ENSCAFG00845030776	468	484	500	499
ENSCAFG00845030777	649	631	605	600
ENSCAFG00845030778	1741	1666	1703	1702
ENSCAFG00845027196	0	0	0	0
ENSCAFG00845030779	0	2	1	1
ENSCAFG00845027195	737	755	588	585
ENSCAFG00845027198	0	2	1	3
ENSCAFG00845027197	0	0	0	0
ENSCAFG00845027192	1682	1534	1588	1561
ENSCAFG00845027194	0	0	0	0
ENSCAFG00845027193	0	0	0	0
ENSCAFG00845030770	67	100	82	95
ENSCAFG00845030771	99	117	66	103
ENSCAFG00845030772	1011	1054	1082	1079
ENSCAFG00845027190	0	0	0	0
ENSCAFG00845030773	0	1	0	0
ENSCAFG00845030774	1115	1119	939	1056
ENSCAFG00845027189	0	0	0	0
ENSCAFG00845030786	830	790	728	762
ENSCAFG00845027188	0	0	0	0
ENSCAFG00845030787	9	16	5	3
ENSCAFG00845030788	1514	1416	1380	1429

ENSCAFG00845030789	0	5	3	1
ENSCAFG00845027185	2273	2131	3007	2858
ENSCAFG00845027184	0	0	0	0
ENSCAFG00845027187	67	55	30	38
ENSCAFG00845027186	945	854	781	817
ENSCAFG00845027181	328	384	399	366
ENSCAFG00845027180	21	16	23	16
ENSCAFG00845027183	9	23	12	7
ENSCAFG00845030780	1	0	1	1
ENSCAFG00845027182	0	0	0	0
ENSCAFG00845030781	0	0	0	0
ENSCAFG00845030782	0	0	0	0
ENSCAFG00845030783	3	12	7	3
ENSCAFG00845030784	523	511	575	526
ENSCAFG00845030785	217	224	197	212
ENSCAFG00845015198	0	3	4	2
ENSCAFG00845015199	0	1	0	0
ENSCAFG00845015196	1090	1162	1011	1120
ENSCAFG00845015197	0	0	0	1
ENSCAFG00845015194	0	0	0	0
ENSCAFG00845015195	0	0	0	0
ENSCAFG00845015192	265	251	289	257
ENSCAFG00845015193	2	0	0	0
ENSCAFG00845015190	1	4	1	3
ENSCAFG00845015191	1907	1781	1905	1874
ENSCAFG00845027178	6	0	0	1
ENSCAFG00845030797	1653	1452	1733	1812
ENSCAFG00845027177	1	0	0	0
ENSCAFG00845030798	241	192	196	187
ENSCAFG00845030799	6483	6003	5303	5332
ENSCAFG00845027179	475	466	477	478
ENSCAFG00845027174	870	855	868	870
ENSCAFG00845027173	0	0	0	0
ENSCAFG00845027176	12	7	12	16
ENSCAFG00845027175	3324	3238	3339	3616
ENSCAFG00845027170	217	193	272	251
ENSCAFG00845030790	0	0	0	0
ENSCAFG00845027172	2	1	1	0
ENSCAFG00845030791	14	12	14	11
ENSCAFG00845027171	3841	3865	3678	3646
ENSCAFG00845030792	153	134	125	135

ENSCAFG00845030793	511	505	515	565
ENSCAFG00845030794	1325	1352	1371	1243
ENSCAFG00845030795	1786	1676	1753	1850
ENSCAFG00845030796	656	626	690	683
ENSCAFG00845015189	65	74	87	66
ENSCAFG00845015187	0	0	0	0
ENSCAFG00845015188	0	0	0	0
ENSCAFG00845015185	43	42	58	45
ENSCAFG00845015186	277	249	188	247
ENSCAFG00845015183	0	0	0	0
ENSCAFG00845015184	1	2	0	0
ENSCAFG00845015181	0	0	0	0
ENSCAFG00845015182	6	6	7	6
ENSCAFG00845015180	0	0	0	0
ENSCAFG00845027167	3	2	3	4
ENSCAFG00845027166	34	31	24	21
ENSCAFG00845027169	6	1	3	2
ENSCAFG00845027168	85	131	94	98
ENSCAFG00845027162	47	74	64	80
ENSCAFG00845027165	828	813	711	836
ENSCAFG00845027164	0	0	0	0
ENSCAFG00845027161	1089	1237	1479	1491
ENSCAFG00845027160	0	0	0	1
ENSCAFG00845030700	1067	1060	1149	1116
ENSCAFG00845030702	666	564	616	660
ENSCAFG00845030703	1	0	1	1
ENSCAFG00845030704	2090	2089	1911	1934
ENSCAFG00845030706	2	3	1	2
ENSCAFG00845030707	0	0	0	0
ENSCAFG00845030708	663	618	529	529
ENSCAFG00845030709	1177	1129	1072	1103
ENSCAFG00845030710	198	180	201	230
ENSCAFG00845030711	852	760	809	931
ENSCAFG00845030712	0	0	0	0
ENSCAFG00845030713	954	901	759	785
ENSCAFG00845030714	219	206	185	207
ENSCAFG00845030715	10	7	12	8
ENSCAFG00845030716	0	0	0	2
ENSCAFG00845030717	2	2	0	0
ENSCAFG00845030718	136	144	153	141
ENSCAFG00845030719	1349	1289	1303	1374

ENSCAFG00845030720	2618	2475	2412	2502
ENSCAFG00845030721	1489	1394	1390	1352
ENSCAFG00845030722	0	0	0	0
ENSCAFG00845030723	2140	1977	2085	2071
ENSCAFG00845030724	0	0	0	0
ENSCAFG00845030725	0	0	0	0
ENSCAFG00845030726	644	653	535	588
ENSCAFG00845030727	1	1	0	1
ENSCAFG00845017753	5	1	0	0
ENSCAFG00845017752	2	1	1	1
ENSCAFG00845017751	2479	2240	2155	2236
ENSCAFG00845017750	111	108	75	102
ENSCAFG00845003109	8	5	2	15
ENSCAFG00845029739	0	0	0	0
ENSCAFG00845003108	0	0	0	0
ENSCAFG00845003107	0	0	0	0
ENSCAFG00845029737	5	5	9	4
ENSCAFG00845003106	23	13	32	39
ENSCAFG00845005770	1	0	0	0
ENSCAFG00845029738	0	0	0	0
ENSCAFG00845003105	6726	6764	6044	6142
ENSCAFG00845005771	443	454	491	517
ENSCAFG00845029735	991	969	1037	1069
ENSCAFG00845003104	6	4	1	0
ENSCAFG00845005772	1	0	1	3
ENSCAFG00845029736	1689	1526	1569	1578
ENSCAFG00845003103	3	3	2	7
ENSCAFG00845005773	1328	1338	1217	1163
ENSCAFG00845029733	10	12	8	8
ENSCAFG00845003102	0	0	0	0
ENSCAFG00845005774	0	0	0	0
ENSCAFG00845029734	0	0	0	0
ENSCAFG00845003101	0	0	0	0
ENSCAFG00845005775	4	8	7	7
ENSCAFG00845029731	0	0	0	0
ENSCAFG00845003100	2694	2652	2510	2692
ENSCAFG00845005776	174	162	195	188
ENSCAFG00845029732	48	27	37	24
ENSCAFG00845005777	0	1	0	0
ENSCAFG00845005778	0	1	0	0
ENSCAFG00845029730	0	0	0	0

ENSCAFG00845005779	5	3	5	6
ENSCAFG00845017759	0	0	0	0
ENSCAFG00845017758	0	0	0	0
ENSCAFG00845017757	3189	3013	3501	3577
ENSCAFG00845017756	0	0	0	0
ENSCAFG00845017755	1273	1186	1258	1233
ENSCAFG00845017754	349	360	299	305
ENSCAFG00845017742	0	0	0	0
ENSCAFG00845017741	0	0	0	0
ENSCAFG00845017740	0	0	0	0
ENSCAFG00845029728	0	0	0	0
ENSCAFG00845003119	77	73	55	60
ENSCAFG00845029729	3	0	1	3
ENSCAFG00845003118	3	0	2	4
ENSCAFG00845029726	0	0	0	0
ENSCAFG00845003117	0	0	0	0
ENSCAFG00845029727	96	132	160	130
ENSCAFG00845003116	472	482	429	469
ENSCAFG00845005760	0	0	0	0
ENSCAFG00845029724	464	398	451	459
ENSCAFG00845003115	0	0	0	0
ENSCAFG00845005761	1193	1152	1170	1175
ENSCAFG00845029725	0	0	0	0
ENSCAFG00845003114	114	126	111	94
ENSCAFG00845005762	8	4	4	6
ENSCAFG00845029722	27	29	17	29
ENSCAFG00845003113	0	0	0	0
ENSCAFG00845005763	15	11	9	10
ENSCAFG00845029723	113	103	78	105
ENSCAFG00845003112	8	7	2	0
ENSCAFG00845005764	1	0	0	3
ENSCAFG00845029720	267	222	240	301
ENSCAFG00845003111	1	0	0	0
ENSCAFG00845005765	865	777	848	984
ENSCAFG00845029721	555	537	652	567
ENSCAFG00845003110	3	3	10	5
ENSCAFG00845005766	7	0	7	1
ENSCAFG00845005767	0	0	0	0
ENSCAFG00845005768	0	0	0	0
ENSCAFG00845005769	0	0	0	0
ENSCAFG00845017749	0	1	0	0

ENSCAFG00845017748	0	0	0	0
ENSCAFG00845017747	9	3	1	6
ENSCAFG00845017746	212	208	237	243
ENSCAFG00845017745	159	139	146	169
ENSCAFG00845017744	1609	1657	1500	1444
ENSCAFG00845017743	0	0	0	0
ENSCAFG00845017731	1	1	0	1
ENSCAFG00845017730	0	0	0	0
ENSCAFG00845029719	0	1	1	0
ENSCAFG00845029717	0	3	0	0
ENSCAFG00845029718	61	64	46	60
ENSCAFG00845029715	685	703	625	620
ENSCAFG00845029716	0	0	0	0
ENSCAFG00845029713	22	18	21	10
ENSCAFG00845005750	28	16	17	23
ENSCAFG00845029714	0	0	1	0
ENSCAFG00845005751	666	602	663	640
ENSCAFG00845029711	0	0	0	0
ENSCAFG00845005752	614	599	966	870
ENSCAFG00845029712	0	0	1	0
ENSCAFG00845005753	0	0	0	0
ENSCAFG00845005754	4	11	6	8
ENSCAFG00845029710	65	68	64	62
ENSCAFG00845005755	6	2	1	3
ENSCAFG00845005756	1649	1463	1383	1340
ENSCAFG00845005757	0	0	0	0
ENSCAFG00845005758	698	730	855	828
ENSCAFG00845005759	10590	10355	9665	9424
ENSCAFG00845017739	0	0	0	0
ENSCAFG00845017738	0	0	0	0
ENSCAFG00845017737	1083	1157	1046	1128
ENSCAFG00845017736	1060	983	980	1019
ENSCAFG00845017734	823	812	857	918
ENSCAFG00845017733	638	627	577	623
ENSCAFG00845017732	548	564	601	642
ENSCAFG00845017720	4	0	3	4
ENSCAFG00845029708	0	0	0	0
ENSCAFG00845029709	0	0	0	0
ENSCAFG00845029706	3	2	0	0
ENSCAFG00845029707	62	44	52	57
ENSCAFG00845029704	1	0	4	0

ENSCAFG00845029705	0	0	0	0
ENSCAFG00845029702	141	122	154	177
ENSCAFG00845029703	4	3	2	1
ENSCAFG00845005740	640	665	612	592
ENSCAFG00845029700	134	172	201	189
ENSCAFG00845005741	0	0	0	0
ENSCAFG00845029701	0	0	0	0
ENSCAFG00845005742	263	245	297	291
ENSCAFG00845005743	472	430	438	509
ENSCAFG00845005744	116	114	102	98
ENSCAFG00845005745	331	303	320	335
ENSCAFG00845005746	7624	7422	7140	7826
ENSCAFG00845005747	0	0	1	0
ENSCAFG00845005748	0	0	0	0
ENSCAFG00845005749	0	0	0	0
ENSCAFG00845017729	0	0	0	0
ENSCAFG00845017728	0	0	0	0
ENSCAFG00845017727	0	0	0	0
ENSCAFG00845017726	0	0	0	0
ENSCAFG00845017725	2	10	4	8
ENSCAFG00845017724	0	0	0	0
ENSCAFG00845017723	71	98	68	78
ENSCAFG00845017722	0	0	1	0
ENSCAFG00845017721	0	0	0	0
ENSCAFG00845005730	31	36	26	25
ENSCAFG00845005731	205	179	190	172
ENSCAFG00845005732	9	9	8	8
ENSCAFG00845005733	964	918	935	990
ENSCAFG00845005734	1	3	1	2
ENSCAFG00845005735	2	5	0	1
ENSCAFG00845005736	0	0	0	0
ENSCAFG00845005737	0	0	0	0
ENSCAFG00845017719	1	0	0	2
ENSCAFG00845005738	0	0	0	0
ENSCAFG00845017718	1007	921	908	948
ENSCAFG00845005739	149	162	139	131
ENSCAFG00845017717	0	3	4	6
ENSCAFG00845017716	0	0	0	1
ENSCAFG00845017715	295	272	203	270
ENSCAFG00845017714	1006	943	907	877
ENSCAFG00845017713	8	2	2	5

ENSCAFG00845017712	0	0	0	0
ENSCAFG00845017711	0	2	0	0
ENSCAFG00845017710	0	0	0	0
ENSCAFG00845005720	1005	972	969	879
ENSCAFG00845005721	9	3	9	4
ENSCAFG00845005722	3	7	12	13
ENSCAFG00845005723	218	228	319	281
ENSCAFG00845005724	1	0	1	3
ENSCAFG00845005725	0	0	0	4
ENSCAFG00845017709	1620	1634	1407	1344
ENSCAFG00845005726	0	0	0	0
ENSCAFG00845017708	817	783	895	867
ENSCAFG00845005727	1387	1426	1168	1201
ENSCAFG00845017707	0	0	0	0
ENSCAFG00845005728	10	14	5	5
ENSCAFG00845017706	0	0	0	0
ENSCAFG00845005729	9	13	11	4
ENSCAFG00845017705	0	0	0	0
ENSCAFG00845017704	0	0	0	0
ENSCAFG00845017703	7	9	4	12
ENSCAFG00845017702	3	0	8	2
ENSCAFG00845017701	0	1	0	0
ENSCAFG00845017700	0	0	0	0
ENSCAFG00845005710	84	80	87	98
ENSCAFG00845005711	0	0	0	0
ENSCAFG00845005712	13	5	8	8
ENSCAFG00845005713	1	1	3	5
ENSCAFG00845005714	0	0	0	0
ENSCAFG00845005715	31	13	17	15
ENSCAFG00845005716	0	0	0	0
ENSCAFG00845005717	0	0	0	0
ENSCAFG00845005718	0	0	0	0
ENSCAFG00845005719	0	0	0	0
ENSCAFG00845005700	2340	2240	1818	1894
ENSCAFG00845005701	107	108	116	120
ENSCAFG00845005702	1	1	0	0
ENSCAFG00845005703	2034	1911	1428	1436
ENSCAFG00845005704	0	0	0	0
ENSCAFG00845005705	3334	3235	3565	3565
ENSCAFG00845005706	155	135	141	135
ENSCAFG00845005707	0	0	0	0

ENSCAFG00845005708	0	0	0	0
ENSCAFG00845005709	1217	1149	1176	1160
ENSCAFG00845015299	1	4	2	8
ENSCAFG00845015297	0	0	0	0
ENSCAFG00845015298	1292	1148	1256	1292
ENSCAFG00845015295	0	0	0	0
ENSCAFG00845015296	0	2	0	4
ENSCAFG00845015293	0	0	1	0
ENSCAFG00845015294	702	632	701	817
ENSCAFG00845015291	1227	1148	1118	1147
ENSCAFG00845015292	0	0	0	0
ENSCAFG00845015290	246	227	240	310
ENSCAFG00845027277	0	0	0	0
ENSCAFG00845027276	0	0	0	0
ENSCAFG00845027279	0	0	0	0
ENSCAFG00845027278	0	0	1	0
ENSCAFG00845027273	490	458	544	568
ENSCAFG00845027272	0	0	0	0
ENSCAFG00845027275	0	1	1	0
ENSCAFG00845027274	78	73	76	68
ENSCAFG00845027271	9	13	14	16
ENSCAFG00845015288	600	506	561	502
ENSCAFG00845015289	0	0	0	0
ENSCAFG00845015286	119	117	135	150
ENSCAFG00845015287	4	1	2	1
ENSCAFG00845015284	0	2	1	1
ENSCAFG00845015285	0	0	0	0
ENSCAFG00845015282	842	764	692	686
ENSCAFG00845015283	0	0	0	0
ENSCAFG00845015280	7	1	10	6
ENSCAFG00845015281	169	155	202	154
ENSCAFG00845027269	34	35	50	39
ENSCAFG00845027266	0	0	0	0
ENSCAFG00845027265	0	0	0	0
ENSCAFG00845027268	1394	1379	1355	1498
ENSCAFG00845027267	0	0	0	1
ENSCAFG00845027262	3	0	2	1
ENSCAFG00845027261	104	125	132	130
ENSCAFG00845027264	1	1	1	1
ENSCAFG00845027263	64	54	65	59
ENSCAFG00845027260	6	7	14	3

ENSCAFG00845015277	0	0	0	0
ENSCAFG00845015278	3672	3416	2965	3064
ENSCAFG00845015275	0	0	0	0
ENSCAFG00845015276	1339	1278	1187	1272
ENSCAFG00845015273	3	3	1	5
ENSCAFG00845015274	0	2	0	0
ENSCAFG00845015271	3	4	3	4
ENSCAFG00845015272	177	162	142	166
ENSCAFG00845015270	0	0	0	0
ENSCAFG00845027259	5	7	4	0
ENSCAFG00845027258	2	2	4	1
ENSCAFG00845003289	3	0	0	1
ENSCAFG00845003288	391	373	351	344
ENSCAFG00845027255	1046	902	938	960
ENSCAFG00845003287	2	0	0	2
ENSCAFG00845027254	12	25	12	12
ENSCAFG00845003286	52	27	46	57
ENSCAFG00845027257	0	0	0	0
ENSCAFG00845003285	0	0	0	0
ENSCAFG00845027256	106	115	114	117
ENSCAFG00845003284	3178	3277	3201	3282
ENSCAFG00845027251	2	0	0	1
ENSCAFG00845003283	454	429	420	437
ENSCAFG00845027250	0	0	0	0
ENSCAFG00845003282	0	0	0	0
ENSCAFG00845027253	2	1	0	0
ENSCAFG00845003281	2354	2133	1755	1822
ENSCAFG00845003280	1	2	3	1
ENSCAFG00845015279	662	675	527	540
ENSCAFG00845015266	22	16	17	29
ENSCAFG00845015267	28980	27849	27582	28195
ENSCAFG00845015264	212	210	238	244
ENSCAFG00845015265	0	0	0	0
ENSCAFG00845015262	0	0	0	0
ENSCAFG00845015263	0	0	0	0
ENSCAFG00845015260	0	0	0	0
ENSCAFG00845015261	0	0	0	1
ENSCAFG00845027248	364	341	356	393
ENSCAFG00845027247	8	4	5	13
ENSCAFG00845027249	423	445	396	384
ENSCAFG00845003299	96	77	87	72

ENSCAFG00845027244	25	28	30	38
ENSCAFG00845003298	122	109	94	114
ENSCAFG00845027243	699	736	728	783
ENSCAFG00845003297	7673	7707	7589	7508
ENSCAFG00845027246	0	0	0	0
ENSCAFG00845003296	246	290	260	214
ENSCAFG00845027245	515	499	337	334
ENSCAFG00845003295	2552	2321	2278	2379
ENSCAFG00845027240	2	0	0	0
ENSCAFG00845003294	3024	2763	2765	2817
ENSCAFG00845003293	1409	1403	1595	1565
ENSCAFG00845027242	4	16	20	7
ENSCAFG00845003292	4	7	0	3
ENSCAFG00845003291	908	848	897	867
ENSCAFG00845003290	0	0	0	0
ENSCAFG00845015268	0	0	2	3
ENSCAFG00845015269	0	0	0	0
ENSCAFG00845015255	2	3	4	7
ENSCAFG00845015256	1332	1197	1101	1224
ENSCAFG00845015253	3	1	1	2
ENSCAFG00845015254	0	0	0	0
ENSCAFG00845015251	0	0	0	0
ENSCAFG00845015252	0	0	0	0
ENSCAFG00845015250	0	0	0	0
ENSCAFG00845027237	0	0	0	0
ENSCAFG00845003269	0	0	0	0
ENSCAFG00845027236	0	0	0	0
ENSCAFG00845003268	0	0	0	0
ENSCAFG00845027239	0	0	0	0
ENSCAFG00845029898	3	0	0	3
ENSCAFG00845003267	20	17	22	22
ENSCAFG00845027238	8	7	17	25
ENSCAFG00845029899	7	7	8	14
ENSCAFG00845003266	1760	1670	1775	1685
ENSCAFG00845027233	0	2	1	0
ENSCAFG00845029896	373	288	315	310
ENSCAFG00845003265	1197	1093	1198	1241
ENSCAFG00845027232	752	758	721	720
ENSCAFG00845029897	1373	1269	1237	1295
ENSCAFG00845003264	1271	1173	1138	1121
ENSCAFG00845027235	1246	1156	957	1043

ENSCAFG00845029894	38	18	20	27
ENSCAFG00845003263	0	0	0	0
ENSCAFG00845027234	35	20	27	21
ENSCAFG00845029895	0	0	0	0
ENSCAFG00845003262	0	0	0	0
ENSCAFG00845029892	4	2	4	5
ENSCAFG00845003261	28	16	17	15
ENSCAFG00845029893	189	186	157	153
ENSCAFG00845003260	8	8	12	5
ENSCAFG00845027231	0	0	2	2
ENSCAFG00845029890	0	0	0	0
ENSCAFG00845027230	0	0	0	0
ENSCAFG00845029891	0	0	0	0
ENSCAFG00845015259	0	0	0	0
ENSCAFG00845015257	7192	6824	6885	6872
ENSCAFG00845015258	601	562	595	595
ENSCAFG00845015244	327	305	313	300
ENSCAFG00845015245	385	409	370	370
ENSCAFG00845015242	0	0	0	0
ENSCAFG00845015243	2	2	1	0
ENSCAFG00845015240	0	0	0	0
ENSCAFG00845015241	3491	3310	3227	3167
ENSCAFG00845027229	0	0	0	1
ENSCAFG00845027226	161	158	185	196
ENSCAFG00845029889	0	0	0	0
ENSCAFG00845027225	0	0	0	0
ENSCAFG00845003279	909	826	875	869
ENSCAFG00845027228	261	228	196	218
ENSCAFG00845029887	5	3	3	4
ENSCAFG00845003278	23	39	19	26
ENSCAFG00845027227	73	86	96	90
ENSCAFG00845029888	9	19	17	16
ENSCAFG00845003277	11373	11136	10935	11131
ENSCAFG00845027222	328	348	349	305
ENSCAFG00845029885	0	0	0	0
ENSCAFG00845003276	5	5	6	5
ENSCAFG00845027221	273	283	314	330
ENSCAFG00845029886	16	10	16	15
ENSCAFG00845003275	276	282	291	273
ENSCAFG00845027224	315	304	322	248
ENSCAFG00845029883	0	2	0	3

ENSCAFG00845003274	1200	1059	1000	984
ENSCAFG00845027223	2	0	0	0
ENSCAFG00845029884	0	0	0	0
ENSCAFG00845003273	50	72	114	124
ENSCAFG00845029881	965	928	858	917
ENSCAFG00845003272	221	230	180	211
ENSCAFG00845029882	10	8	11	8
ENSCAFG00845003271	0	0	0	0
ENSCAFG00845027220	61	54	53	60
ENSCAFG00845003270	42	69	62	37
ENSCAFG00845029880	0	0	0	0
ENSCAFG00845015248	1238	1229	991	1194
ENSCAFG00845015249	115	157	94	92
ENSCAFG00845015246	907	848	786	879
ENSCAFG00845015247	592	580	606	600
ENSCAFG00845015233	0	0	0	0
ENSCAFG00845017896	0	0	0	1
ENSCAFG00845015234	0	0	1	1
ENSCAFG00845017895	0	0	0	0
ENSCAFG00845015231	0	1	0	0
ENSCAFG00845017894	0	0	0	0
ENSCAFG00845015232	71	54	33	61
ENSCAFG00845017893	969	1020	1080	1051
ENSCAFG00845017892	7	2	0	3
ENSCAFG00845015230	0	0	0	0
ENSCAFG00845017891	0	0	0	0
ENSCAFG00845017890	0	0	0	0
ENSCAFG00845027219	1	0	1	1
ENSCAFG00845027218	0	0	0	0
ENSCAFG00845003249	600	556	575	612
ENSCAFG00845003248	2998	2918	2797	3050
ENSCAFG00845027215	10	7	6	5
ENSCAFG00845029878	0	0	0	0
ENSCAFG00845003247	2	3	4	2
ENSCAFG00845029879	12	10	20	22
ENSCAFG00845003246	73	92	69	100
ENSCAFG00845027217	3056	2836	2619	2806
ENSCAFG00845029876	1	0	1	2
ENSCAFG00845003245	427	406	419	454
ENSCAFG00845027216	1879	1859	1927	2117
ENSCAFG00845029877	0	0	0	0

ENSCAFG00845003244	0	0	0	0
ENSCAFG00845027211	0	2	0	0
ENSCAFG00845029874	81	86	83	70
ENSCAFG00845003243	6284	5989	5887	5735
ENSCAFG00845027210	359	310	338	336
ENSCAFG00845029875	0	0	0	0
ENSCAFG00845003242	16	9	5	13
ENSCAFG00845027213	638	611	652	593
ENSCAFG00845029872	1898	1816	1740	1727
ENSCAFG00845003241	0	0	0	0
ENSCAFG00845029873	0	0	0	0
ENSCAFG00845003240	0	1	0	0
ENSCAFG00845029870	0	0	0	0
ENSCAFG00845029871	24	20	16	28
ENSCAFG00845015239	0	0	0	0
ENSCAFG00845015237	0	0	0	0
ENSCAFG00845015238	54	33	68	53
ENSCAFG00845017899	47	34	49	41
ENSCAFG00845015235	0	0	0	0
ENSCAFG00845017898	214	185	208	163
ENSCAFG00845015236	0	0	0	0
ENSCAFG00845017897	587	516	491	582
ENSCAFG00845015222	0	0	0	0
ENSCAFG00845017885	1	1	0	0
ENSCAFG00845015223	1	2	2	2
ENSCAFG00845017884	0	0	0	0
ENSCAFG00845015220	15005	14206	13671	14292
ENSCAFG00845017883	0	0	2	1
ENSCAFG00845015221	4735	4465	4511	4635
ENSCAFG00845017882	0	0	0	0
ENSCAFG00845017881	0	0	0	0
ENSCAFG00845017880	0	0	0	0
ENSCAFG00845027208	1023	921	868	979
ENSCAFG00845027207	0	0	0	0
ENSCAFG00845029869	0	1	0	0
ENSCAFG00845027209	0	0	0	0
ENSCAFG00845003259	0	0	0	0
ENSCAFG00845027204	0	0	0	1
ENSCAFG00845029867	220	218	235	235
ENSCAFG00845003258	0	0	0	1
ENSCAFG00845027203	1	0	0	0

ENSCAFG00845029868	0	0	0	0
ENSCAFG00845003257	791	821	755	829
ENSCAFG00845027206	0	0	0	1
ENSCAFG00845029865	0	0	0	0
ENSCAFG00845003256	390	371	406	344
ENSCAFG00845027205	5218	5174	5491	5634
ENSCAFG00845029866	500	483	516	550
ENSCAFG00845003255	1	0	1	0
ENSCAFG00845027200	60	60	96	83
ENSCAFG00845029863	1116	1059	950	863
ENSCAFG00845003254	2	3	4	0
ENSCAFG00845029864	0	0	0	0
ENSCAFG00845003253	2	1	1	1
ENSCAFG00845027202	1	0	2	0
ENSCAFG00845029861	1937	1836	1910	1885
ENSCAFG00845003252	895	829	829	787
ENSCAFG00845027201	0	0	0	0
ENSCAFG00845029862	0	0	0	0
ENSCAFG00845003251	1296	1083	1089	1122
ENSCAFG00845003250	2577	2421	2220	2401
ENSCAFG00845029860	234	209	334	335
ENSCAFG00845015228	170	175	168	170
ENSCAFG00845015229	884	911	955	1009
ENSCAFG00845015226	10111	9729	9509	9860
ENSCAFG00845017889	0	0	0	0
ENSCAFG00845015227	0	0	0	0
ENSCAFG00845017888	0	0	0	0
ENSCAFG00845015224	247	244	245	224
ENSCAFG00845017887	2	3	8	5
ENSCAFG00845015225	3	1	0	1
ENSCAFG00845017886	1	0	1	0
ENSCAFG00845030849	269	287	124	134
ENSCAFG00845030852	0	0	0	0
ENSCAFG00845030853	9	17	7	6
ENSCAFG00845030854	0	0	0	0
ENSCAFG00845030855	0	0	0	0
ENSCAFG00845030856	0	0	0	0
ENSCAFG00845030857	0	0	0	0
ENSCAFG00845030858	0	1	2	0
ENSCAFG00845030859	191	216	274	267
ENSCAFG00845030850	0	0	0	0

ENSCAFG00845030851	254	227	160	193
ENSCAFG00845030863	1410	1271	1342	1426
ENSCAFG00845030864	0	0	0	0
ENSCAFG00845030865	5256	5385	5682	5426
ENSCAFG00845030866	14	11	12	14
ENSCAFG00845030867	0	0	0	0
ENSCAFG00845030868	0	0	0	0
ENSCAFG00845030869	5	3	3	1
ENSCAFG00845030860	0	0	0	0
ENSCAFG00845030861	0	0	0	0
ENSCAFG00845030862	0	0	0	0
ENSCAFG00845030874	0	1	0	0
ENSCAFG00845030875	3993	3759	3546	3754
ENSCAFG00845030876	1	0	0	0
ENSCAFG00845030877	0	0	0	0
ENSCAFG00845030878	0	0	0	0
ENSCAFG00845030879	5	5	5	9
ENSCAFG00845030870	0	0	0	0
ENSCAFG00845030871	0	0	0	0
ENSCAFG00845030872	0	0	0	0
ENSCAFG00845030873	8	10	18	21
ENSCAFG00845030885	472	432	500	455
ENSCAFG00845030886	0	0	0	0
ENSCAFG00845030887	988	883	893	831
ENSCAFG00845030888	331	320	324	316
ENSCAFG00845030889	0	1	1	4
ENSCAFG00845030880	0	2	0	0
ENSCAFG00845030881	0	0	0	0
ENSCAFG00845030882	0	0	1	0
ENSCAFG00845030883	0	1	1	0
ENSCAFG00845030884	3	0	8	5
ENSCAFG00845030896	0	0	0	0
ENSCAFG00845030897	0	0	0	0
ENSCAFG00845030898	251	257	94	100
ENSCAFG00845030899	0	0	0	0
ENSCAFG00845030890	2	0	0	1
ENSCAFG00845030891	43	42	40	30
ENSCAFG00845030892	1233	1270	1075	1166
ENSCAFG00845030893	0	0	0	0
ENSCAFG00845030894	2	1	4	2
ENSCAFG00845030895	34	34	35	26

ENSCAFG00845027299	81	70	79	54
ENSCAFG00845027298	0	0	0	0
ENSCAFG00845027295	11	7	8	8
ENSCAFG00845027294	0	0	0	0
ENSCAFG00845027297	0	0	0	1
ENSCAFG00845027296	16	5	10	7
ENSCAFG00845027291	1134	1023	959	1012
ENSCAFG00845027290	246	197	230	254
ENSCAFG00845027293	0	0	0	0
ENSCAFG00845027292	1018	986	1020	993
ENSCAFG00845027288	0	0	0	0
ENSCAFG00845027287	92	73	92	95
ENSCAFG00845027289	900	849	895	974
ENSCAFG00845027283	0	0	0	0
ENSCAFG00845027286	645	677	632	732
ENSCAFG00845027285	0	0	0	0
ENSCAFG00845027280	1	0	0	0
ENSCAFG00845027282	3121	2941	2852	3018
ENSCAFG00845027281	1364	1342	1463	1465
ENSCAFG00845005810	31	40	48	34
ENSCAFG00845005811	0	0	0	0
ENSCAFG00845005812	573	516	518	510
ENSCAFG00845005813	0	0	0	0
ENSCAFG00845005814	0	0	0	0
ENSCAFG00845005815	0	0	0	1
ENSCAFG00845005816	52	39	28	33
ENSCAFG00845005817	1434	1342	1465	1496
ENSCAFG00845005818	0	0	0	0
ENSCAFG00845005819	1	0	0	0
ENSCAFG00845005800	0	0	0	0
ENSCAFG00845005801	0	0	0	0
ENSCAFG00845005802	728	608	555	547
ENSCAFG00845005803	20	16	19	15
ENSCAFG00845005804	3	3	2	2
ENSCAFG00845005805	1202	1084	962	1047
ENSCAFG00845005806	375	371	377	368
ENSCAFG00845005807	0	0	0	0
ENSCAFG00845005808	0	0	0	0
ENSCAFG00845005809	0	0	0	0
ENSCAFG00845030800	0	0	0	0
ENSCAFG00845030801	6	4	6	3

ENSCAFG00845030802	0	0	0	0
ENSCAFG00845030803	0	0	0	0
ENSCAFG00845030804	1	4	1	1
ENSCAFG00845030805	829	706	764	773
ENSCAFG00845030806	517	454	507	540
ENSCAFG00845030807	2	1	0	0
ENSCAFG00845030808	805	863	984	998
ENSCAFG00845030809	2893	3009	2684	2909
ENSCAFG00845030810	74	67	107	95
ENSCAFG00845030811	0	0	0	0
ENSCAFG00845030812	1195	1122	1366	1392
ENSCAFG00845030813	0	0	0	0
ENSCAFG00845030814	2	2	8	5
ENSCAFG00845030815	669	787	660	657
ENSCAFG00845030816	0	0	0	0
ENSCAFG00845030817	0	0	0	0
ENSCAFG00845030818	0	0	0	0
ENSCAFG00845030819	0	0	0	0
ENSCAFG00845030820	1620	1510	1229	1309
ENSCAFG00845030821	2	9	2	3
ENSCAFG00845030822	1567	1378	1442	1378
ENSCAFG00845030823	13	20	12	11
ENSCAFG00845030824	338	310	361	330
ENSCAFG00845030825	993	1023	922	887
ENSCAFG00845030826	0	0	0	0
ENSCAFG00845030827	475	478	422	464
ENSCAFG00845030828	0	0	0	0
ENSCAFG00845030829	0	0	0	0
ENSCAFG00845030830	8	1	10	6
ENSCAFG00845030831	17	21	24	18
ENSCAFG00845030832	0	0	0	0
ENSCAFG00845030833	0	0	0	1
ENSCAFG00845030834	125	128	169	169
ENSCAFG00845030835	0	0	0	2
ENSCAFG00845030836	0	0	0	0
ENSCAFG00845030837	0	0	0	0
ENSCAFG00845030838	0	0	0	0
ENSCAFG00845030839	0	0	0	0
ENSCAFG00845030841	0	0	0	0
ENSCAFG00845030842	0	0	0	0
ENSCAFG00845030843	0	0	0	0

ENSCAFG00845030844	0	0	0	0
ENSCAFG00845030845	1	0	0	0
ENSCAFG00845030846	0	0	0	0
ENSCAFG00845030847	0	0	0	0
ENSCAFG00845030848	0	0	0	0
ENSCAFG00845030840	0	0	0	0
ENSCAFG00845015211	0	0	0	0
ENSCAFG00845017874	0	0	0	1
ENSCAFG00845015212	0	0	0	0
ENSCAFG00845017873	0	0	0	0
ENSCAFG00845017872	15	9	17	15
ENSCAFG00845015210	11	13	15	21
ENSCAFG00845017871	0	0	0	0
ENSCAFG00845017870	0	0	0	0
ENSCAFG00845003229	1135	1068	1119	1157
ENSCAFG00845003228	0	0	0	1
ENSCAFG00845005890	898	865	742	879
ENSCAFG00845029858	2367	2162	2374	2546
ENSCAFG00845003227	5	1	2	3
ENSCAFG00845005891	1940	1949	1934	2097
ENSCAFG00845029859	0	0	0	0
ENSCAFG00845003226	179	191	187	161
ENSCAFG00845005892	276	270	293	310
ENSCAFG00845029856	1235	1160	1206	1238
ENSCAFG00845003225	3	3	6	0
ENSCAFG00845005893	0	0	0	0
ENSCAFG00845029857	1304	1135	1262	1233
ENSCAFG00845003224	606	622	767	857
ENSCAFG00845005894	0	0	0	0
ENSCAFG00845029854	450	372	459	458
ENSCAFG00845003223	16	17	28	49
ENSCAFG00845005895	36	19	44	28
ENSCAFG00845029855	985	883	771	753
ENSCAFG00845003222	0	0	0	0
ENSCAFG00845005896	154	157	159	163
ENSCAFG00845029852	1692	1669	1562	1680
ENSCAFG00845003221	435	447	287	376
ENSCAFG00845005897	1214	1256	1250	1224
ENSCAFG00845029853	188	151	166	182
ENSCAFG00845003220	1592	1476	1570	1623
ENSCAFG00845005898	0	0	0	0

ENSCAFG00845029850	0	0	0	0
ENSCAFG00845005899	0	0	0	0
ENSCAFG00845029851	20	21	17	15
ENSCAFG00845015219	1096	1126	1022	1109
ENSCAFG00845015217	1941	1903	1917	2051
ENSCAFG00845015218	527	502	507	548
ENSCAFG00845017879	692	641	589	552
ENSCAFG00845015215	0	0	0	0
ENSCAFG00845017878	1847	1661	1772	1774
ENSCAFG00845015216	0	0	0	0
ENSCAFG00845017877	92	69	60	84
ENSCAFG00845015213	0	0	1	0
ENSCAFG00845017876	371	331	284	298
ENSCAFG00845015214	5	8	5	8
ENSCAFG00845017875	122	136	121	145
ENSCAFG00845015200	2197	2230	2545	2355
ENSCAFG00845017863	46	49	32	46
ENSCAFG00845015201	1940	1915	1948	1900
ENSCAFG00845017862	160	183	152	187
ENSCAFG00845017861	419	416	390	362
ENSCAFG00845017860	0	1	0	0
ENSCAFG00845029849	763	667	1046	938
ENSCAFG00845003239	0	0	0	0
ENSCAFG00845029847	172	124	150	150
ENSCAFG00845003238	0	0	0	0
ENSCAFG00845005880	0	0	0	0
ENSCAFG00845029848	240	212	236	253
ENSCAFG00845003237	0	0	0	0
ENSCAFG00845005881	2403	2432	2467	2638
ENSCAFG00845029845	224	260	177	183
ENSCAFG00845003236	0	1	1	0
ENSCAFG00845005882	0	0	0	0
ENSCAFG00845029846	1473	1372	1375	1460
ENSCAFG00845003235	0	0	0	0
ENSCAFG00845005883	4	1	1	0
ENSCAFG00845029843	0	0	0	0
ENSCAFG00845003234	212	203	221	253
ENSCAFG00845005884	0	0	0	0
ENSCAFG00845029844	0	0	0	0
ENSCAFG00845003233	50	46	62	55
ENSCAFG00845005885	416	413	423	377

ENSCAFG00845029841	2	2	0	1
ENSCAFG00845003232	13	15	32	30
ENSCAFG00845005886	278	291	265	303
ENSCAFG00845029842	3978	3847	3156	3175
ENSCAFG00845003231	0	0	0	0
ENSCAFG00845005887	1580	1444	1562	1466
ENSCAFG00845003230	1491	1433	1466	1572
ENSCAFG00845005888	7	6	8	3
ENSCAFG00845029840	4409	4312	5402	5454
ENSCAFG00845005889	514	513	471	489
ENSCAFG00845015208	0	0	0	0
ENSCAFG00845015209	1487	1405	1345	1449
ENSCAFG00845015206	66	64	54	82
ENSCAFG00845017869	0	0	0	0
ENSCAFG00845015207	0	0	0	1
ENSCAFG00845017868	0	0	0	1
ENSCAFG00845015204	0	0	0	0
ENSCAFG00845017867	648	633	654	681
ENSCAFG00845015205	0	0	0	0
ENSCAFG00845017866	859	886	838	898
ENSCAFG00845015202	0	0	0	0
ENSCAFG00845017865	18	17	13	22
ENSCAFG00845015203	137	130	149	172
ENSCAFG00845017864	0	2	0	2
ENSCAFG00845017852	0	0	0	0
ENSCAFG00845017851	7146	7075	6641	6935
ENSCAFG00845017850	4	0	0	0
ENSCAFG00845003209	1420	1380	1462	1329
ENSCAFG00845003208	0	0	0	0
ENSCAFG00845029838	36	22	44	21
ENSCAFG00845003207	3	0	2	1
ENSCAFG00845029839	65	57	76	53
ENSCAFG00845003206	1121	1068	1131	1125
ENSCAFG00845029836	1046	1034	961	1049
ENSCAFG00845003205	0	0	0	0
ENSCAFG00845029837	330	306	290	252
ENSCAFG00845003204	2088	2018	1952	2142
ENSCAFG00845005870	0	0	0	0
ENSCAFG00845029834	552	558	519	568
ENSCAFG00845003203	200	179	157	159
ENSCAFG00845005871	0	0	0	0

ENSCAFG00845029835	0	1	0	0
ENSCAFG00845003202	3	2	4	1
ENSCAFG00845005872	4	7	1	3
ENSCAFG00845029832	0	0	0	0
ENSCAFG00845003201	31	36	35	50
ENSCAFG00845005873	0	0	0	0
ENSCAFG00845029833	549	536	466	555
ENSCAFG00845003200	0	0	0	0
ENSCAFG00845005874	545	538	591	586
ENSCAFG00845029830	74	53	57	85
ENSCAFG00845005875	0	0	0	0
ENSCAFG00845029831	643	524	588	657
ENSCAFG00845005876	0	0	0	0
ENSCAFG00845005877	839	807	879	859
ENSCAFG00845005878	0	0	4	1
ENSCAFG00845005879	1623	1617	1456	1513
ENSCAFG00845017859	0	0	0	0
ENSCAFG00845017858	2817	2850	2683	2685
ENSCAFG00845017857	3	4	5	0
ENSCAFG00845017856	319	308	272	262
ENSCAFG00845017855	0	0	0	0
ENSCAFG00845017854	8	9	6	12
ENSCAFG00845017853	0	0	0	0
ENSCAFG00845017841	61	50	47	59
ENSCAFG00845017840	384	394	319	307
ENSCAFG00845029829	0	0	0	0
ENSCAFG00845003219	1	0	1	2
ENSCAFG00845029827	95	105	109	111
ENSCAFG00845003218	1419	1429	1224	1199
ENSCAFG00845029828	1844	1573	1679	1873
ENSCAFG00845003217	0	0	0	0
ENSCAFG00845029825	0	1	1	0
ENSCAFG00845003216	7	0	2	2
ENSCAFG00845029826	18	7	5	5
ENSCAFG00845003215	138	128	140	179
ENSCAFG00845029823	0	0	0	0
ENSCAFG00845003214	0	1	0	1
ENSCAFG00845005860	1494	1436	1568	1613
ENSCAFG00845029824	0	0	0	0
ENSCAFG00845003213	3	2	1	1
ENSCAFG00845005861	3	0	0	0

ENSCAFG00845029821	392	431	362	433
ENSCAFG00845003212	2464	2474	2117	2128
ENSCAFG00845005862	1	3	0	2
ENSCAFG00845029822	0	0	0	0
ENSCAFG00845003211	6	5	6	9
ENSCAFG00845005863	0	0	0	0
ENSCAFG00845003210	0	0	0	0
ENSCAFG00845005864	2	4	12	5
ENSCAFG00845029820	45	34	41	32
ENSCAFG00845005865	250	223	238	261
ENSCAFG00845005866	0	0	0	0
ENSCAFG00845005867	246	235	246	230
ENSCAFG00845005868	0	0	0	0
ENSCAFG00845005869	0	0	0	0
ENSCAFG00845017849	0	0	0	0
ENSCAFG00845017848	105	91	79	109
ENSCAFG00845017847	0	0	0	0
ENSCAFG00845017846	0	0	0	1
ENSCAFG00845017845	0	0	0	0
ENSCAFG00845017844	0	0	0	0
ENSCAFG00845017843	1	6	2	7
ENSCAFG00845017842	6	6	5	5
ENSCAFG00845017830	0	1	0	0
ENSCAFG00845029818	0	0	3	0
ENSCAFG00845029819	0	0	0	3
ENSCAFG00845029816	1	0	4	1
ENSCAFG00845029817	0	0	0	0
ENSCAFG00845029814	1837	1606	1675	1665
ENSCAFG00845029815	1	0	0	0
ENSCAFG00845029812	491	520	581	534
ENSCAFG00845029813	8	0	4	2
ENSCAFG00845005850	1	0	0	0
ENSCAFG00845029810	2	0	0	1
ENSCAFG00845005851	0	0	0	1
ENSCAFG00845029811	2	2	1	2
ENSCAFG00845005852	0	0	0	0
ENSCAFG00845005853	7	1	2	2
ENSCAFG00845005854	1	0	2	1
ENSCAFG00845005855	2048	1951	1836	1915
ENSCAFG00845005856	694	705	610	654
ENSCAFG00845005857	0	0	0	0

ENSCAFG00845005858	0	0	0	0
ENSCAFG00845005859	0	0	0	0
ENSCAFG00845017839	927	962	869	990
ENSCAFG00845017838	1	1	1	2
ENSCAFG00845017837	263	231	477	470
ENSCAFG00845017836	470	442	476	407
ENSCAFG00845017835	0	0	0	0
ENSCAFG00845017834	0	0	0	0
ENSCAFG00845017833	3858	3752	3471	3458
ENSCAFG00845017832	311	310	290	304
ENSCAFG00845017831	839	812	911	880
ENSCAFG00845029809	152	192	119	106
ENSCAFG00845029807	0	0	0	0
ENSCAFG00845029808	0	0	0	0
ENSCAFG00845029805	0	0	0	0
ENSCAFG00845029806	5163	5045	5050	5380
ENSCAFG00845029803	1867	1851	1882	1963
ENSCAFG00845029804	0	0	0	0
ENSCAFG00845029801	8056	7666	6953	6909
ENSCAFG00845029802	0	0	0	0
ENSCAFG00845005840	91	78	59	74
ENSCAFG00845029800	17	11	14	8
ENSCAFG00845005841	0	0	0	0
ENSCAFG00845005842	786	790	799	825
ENSCAFG00845005843	0	0	0	0
ENSCAFG00845005844	2238	2034	2117	2204
ENSCAFG00845005845	0	0	0	0
ENSCAFG00845005846	0	0	0	0
ENSCAFG00845005847	844	798	940	953
ENSCAFG00845017829	1145	1040	1192	1255
ENSCAFG00845005848	2	0	0	0
ENSCAFG00845017828	0	0	0	0
ENSCAFG00845005849	62	52	90	68
ENSCAFG00845017826	0	0	0	0
ENSCAFG00845017825	1245	1263	1284	1296
ENSCAFG00845017824	76	85	60	83
ENSCAFG00845017823	23	35	29	21
ENSCAFG00845017822	183	170	158	153
ENSCAFG00845017821	2	0	0	0
ENSCAFG00845017820	0	0	0	0
ENSCAFG00845005830	1566	1227	1355	1535

ENSCAFG00845005831	170	129	142	148
ENSCAFG00845005832	3	3	6	4
ENSCAFG00845005833	0	0	0	0
ENSCAFG00845005834	72	69	43	61
ENSCAFG00845005835	0	0	0	0
ENSCAFG00845017819	0	0	0	0
ENSCAFG00845005836	1028	994	1010	1099
ENSCAFG00845017818	0	0	0	0
ENSCAFG00845005837	89	71	70	107
ENSCAFG00845017817	69	78	71	61
ENSCAFG00845005838	212	200	244	242
ENSCAFG00845017816	0	0	0	0
ENSCAFG00845005839	0	2	0	0
ENSCAFG00845017815	1	0	0	0
ENSCAFG00845017814	0	0	0	0
ENSCAFG00845017813	0	0	0	0
ENSCAFG00845017812	71	71	66	72
ENSCAFG00845017811	8813	8600	9143	9165
ENSCAFG00845017810	34	10	6	11
ENSCAFG00845005820	0	0	0	0
ENSCAFG00845005821	206	210	188	182
ENSCAFG00845005822	0	0	0	0
ENSCAFG00845005823	203	177	193	174
ENSCAFG00845017809	361	312	340	333
ENSCAFG00845005824	0	0	0	0
ENSCAFG00845017808	0	0	0	0
ENSCAFG00845005825	1	0	1	0
ENSCAFG00845017807	1062	889	1092	1110
ENSCAFG00845005826	0	0	0	0
ENSCAFG00845017806	0	0	0	0
ENSCAFG00845005827	0	0	0	0
ENSCAFG00845017805	11025	10770	13864	13870
ENSCAFG00845005828	0	0	0	3
ENSCAFG00845017804	0	0	0	0
ENSCAFG00845005829	689	714	680	651
ENSCAFG00845017803	341	356	294	325
ENSCAFG00845017802	1	0	1	0
ENSCAFG00845017801	0	0	0	0
ENSCAFG00845017800	0	0	0	0
ENSCAFG00845027398	172	155	147	165
ENSCAFG00845027397	0	0	0	0

ENSCAFG00845027399	0	0	0	0
ENSCAFG00845027394	4906	4539	4357	4385
ENSCAFG00845027393	0	0	0	0
ENSCAFG00845027396	211	181	244	203
ENSCAFG00845027395	2691	2523	2769	2807
ENSCAFG00845027390	0	2	0	0
ENSCAFG00845027392	0	0	0	0
ENSCAFG00845027391	0	0	1	0
ENSCAFG00845027387	1456	1440	1322	1355
ENSCAFG00845027386	2	1	3	0
ENSCAFG00845027389	83	75	87	76
ENSCAFG00845027388	0	0	0	0
ENSCAFG00845027383	0	0	0	0
ENSCAFG00845027384	0	1	2	0
ENSCAFG00845027381	47	36	42	36
ENSCAFG00845027380	0	0	0	0
ENSCAFG00845015398	0	0	1	1
ENSCAFG00845015399	0	0	0	0
ENSCAFG00845015396	0	2	0	4
ENSCAFG00845015397	244	238	64	61
ENSCAFG00845015394	265	263	264	267
ENSCAFG00845015395	1028	997	835	817
ENSCAFG00845015392	0	0	0	0
ENSCAFG00845015393	402	388	381	431
ENSCAFG00845015390	489	476	494	480
ENSCAFG00845015391	3	0	0	0
ENSCAFG00845027379	1	4	2	2
ENSCAFG00845027376	27	17	35	34
ENSCAFG00845027375	4094	4013	3911	3836
ENSCAFG00845027378	0	0	0	0
ENSCAFG00845027377	10	5	4	2
ENSCAFG00845027372	0	0	0	0
ENSCAFG00845027371	0	0	0	0
ENSCAFG00845027374	179	199	160	185
ENSCAFG00845027373	1	1	0	1
ENSCAFG00845027370	0	0	0	0
ENSCAFG00845015387	118	124	130	179
ENSCAFG00845015388	325	316	331	317
ENSCAFG00845015385	20	18	20	14
ENSCAFG00845015386	0	0	0	0
ENSCAFG00845015383	4	0	3	8

ENSCAFG00845015384	0	0	0	0
ENSCAFG00845015381	38	36	35	46
ENSCAFG00845015382	1	0	2	0
ENSCAFG00845015380	0	0	0	0
ENSCAFG00845027369	150	165	203	187
ENSCAFG00845027368	12	9	2	9
ENSCAFG00845027365	1002	971	970	931
ENSCAFG00845027364	0	0	0	0
ENSCAFG00845027366	0	0	0	0
ENSCAFG00845027361	82	81	52	88
ENSCAFG00845027360	13	15	12	15
ENSCAFG00845027363	6	5	4	6
ENSCAFG00845027362	0	0	0	0
ENSCAFG00845015389	0	0	0	0
ENSCAFG00845015376	0	1	0	0
ENSCAFG00845015377	10	10	5	4
ENSCAFG00845015374	0	0	0	0
ENSCAFG00845015375	0	0	0	0
ENSCAFG00845015372	0	0	0	0
ENSCAFG00845015373	0	0	0	0
ENSCAFG00845015370	0	0	0	0
ENSCAFG00845015371	833	821	763	730
ENSCAFG00845027358	0	0	0	0
ENSCAFG00845027357	0	0	0	0
ENSCAFG00845003389	0	0	0	0
ENSCAFG00845003388	5	11	8	5
ENSCAFG00845027359	5	2	1	1
ENSCAFG00845003387	5	2	2	2
ENSCAFG00845027354	0	0	0	0
ENSCAFG00845003386	0	0	0	0
ENSCAFG00845027353	4	8	9	3
ENSCAFG00845003385	0	0	0	0
ENSCAFG00845027356	0	0	0	0
ENSCAFG00845003384	874	844	883	947
ENSCAFG00845027355	0	0	0	0
ENSCAFG00845003383	58	84	85	80
ENSCAFG00845027350	0	1	0	0
ENSCAFG00845003382	471	495	506	525
ENSCAFG00845003381	13	18	7	11
ENSCAFG00845027352	1094	1065	1065	1020
ENSCAFG00845003380	0	0	0	0

ENSCAFG00845027351	4	3	1	3
ENSCAFG00845015378	0	0	0	0
ENSCAFG00845015379	39	49	34	38
ENSCAFG00845015365	0	4	2	1
ENSCAFG00845015366	0	0	0	0
ENSCAFG00845015363	0	0	1	0
ENSCAFG00845015364	0	0	0	0
ENSCAFG00845015361	7	11	8	8
ENSCAFG00845015362	674	651	615	556
ENSCAFG00845015360	8	8	5	6
ENSCAFG00845027347	250	218	269	296
ENSCAFG00845027346	1	1	2	1
ENSCAFG00845027349	0	0	0	0
ENSCAFG00845003399	1	0	0	4
ENSCAFG00845027348	0	0	0	0
ENSCAFG00845003398	0	0	0	0
ENSCAFG00845027343	997	978	916	849
ENSCAFG00845003397	135	127	108	122
ENSCAFG00845027342	88	91	93	93
ENSCAFG00845003396	0	0	0	0
ENSCAFG00845027345	1893	1905	1941	1910
ENSCAFG00845003395	23	10	5	17
ENSCAFG00845027344	0	0	0	0
ENSCAFG00845003394	0	0	0	0
ENSCAFG00845003393	1124	1109	1026	1080
ENSCAFG00845003392	0	0	0	0
ENSCAFG00845027341	9	3	0	0
ENSCAFG00845003391	290	262	326	372
ENSCAFG00845027340	0	0	0	0
ENSCAFG00845003390	0	0	0	0
ENSCAFG00845015369	0	0	0	0
ENSCAFG00845015367	1	1	1	0
ENSCAFG00845015368	16	2	11	5
ENSCAFG00845015354	51	40	31	37
ENSCAFG00845015355	18	7	20	22
ENSCAFG00845015352	0	0	0	0
ENSCAFG00845015353	7	5	7	2
ENSCAFG00845015350	79	60	62	80
ENSCAFG00845015351	3	0	9	5
ENSCAFG00845027339	0	0	1	2
ENSCAFG00845003369	0	2	4	4

ENSCAFG00845027336	0	0	0	0
ENSCAFG00845029999	0	0	0	0
ENSCAFG00845003368	492	462	466	460
ENSCAFG00845027335	12	3	8	10
ENSCAFG00845003367	20	12	14	25
ENSCAFG00845029997	0	0	0	0
ENSCAFG00845003366	0	0	0	0
ENSCAFG00845027337	0	0	0	0
ENSCAFG00845029998	0	1	0	2
ENSCAFG00845003365	602	550	582	605
ENSCAFG00845027332	136	143	99	107
ENSCAFG00845029995	0	0	0	0
ENSCAFG00845003364	0	1	0	0
ENSCAFG00845027331	73	54	46	53
ENSCAFG00845029996	0	0	0	0
ENSCAFG00845003363	0	0	0	0
ENSCAFG00845027334	379	359	368	375
ENSCAFG00845029993	39	33	56	60
ENSCAFG00845003362	3794	3888	3302	3443
ENSCAFG00845027333	0	0	0	0
ENSCAFG00845029994	774	835	998	948
ENSCAFG00845003361	7	14	5	2
ENSCAFG00845029991	0	0	0	0
ENSCAFG00845003360	309	294	286	317
ENSCAFG00845029992	1378	1345	1264	1303
ENSCAFG00845027330	1	7	5	5
ENSCAFG00845029990	0	0	0	0
ENSCAFG00845015358	0	0	0	0
ENSCAFG00845015359	0	0	0	0
ENSCAFG00845015356	0	0	0	0
ENSCAFG00845015357	0	0	0	0
ENSCAFG00845015343	0	0	0	0
ENSCAFG00845015344	650	587	636	643
ENSCAFG00845015341	10	14	13	9
ENSCAFG00845015342	0	0	1	0
ENSCAFG00845015340	0	0	0	0
ENSCAFG00845027329	1	1	0	0
ENSCAFG00845027328	2427	2329	2482	2527
ENSCAFG00845027325	0	0	0	2
ENSCAFG00845029988	3	1	3	1
ENSCAFG00845003379	0	0	0	0

ENSCAFG00845027324	468	504	452	432
ENSCAFG00845029989	99	90	67	95
ENSCAFG00845003378	1116	906	895	1017
ENSCAFG00845029986	0	0	0	0
ENSCAFG00845003377	0	0	0	0
ENSCAFG00845027326	594	601	584	606
ENSCAFG00845029987	73	78	73	82
ENSCAFG00845003376	0	1	0	0
ENSCAFG00845029984	0	0	0	0
ENSCAFG00845003375	0	0	0	0
ENSCAFG00845027320	1400	1348	1351	1356
ENSCAFG00845029985	0	1	0	0
ENSCAFG00845003374	0	0	0	0
ENSCAFG00845027323	0	0	0	0
ENSCAFG00845029982	9	7	1	8
ENSCAFG00845003373	422	420	421	420
ENSCAFG00845027322	5869	5635	7017	7187
ENSCAFG00845029983	0	0	0	0
ENSCAFG00845003372	24	35	21	30
ENSCAFG00845029980	2	4	0	4
ENSCAFG00845003371	0	0	0	0
ENSCAFG00845029981	0	0	0	0
ENSCAFG00845003370	298	261	377	332
ENSCAFG00845015349	0	0	0	0
ENSCAFG00845015347	0	0	0	0
ENSCAFG00845015348	186	208	243	313
ENSCAFG00845015345	0	0	2	0
ENSCAFG00845015346	0	0	0	0
ENSCAFG00845030973	1410	1382	1263	1388
ENSCAFG00845030974	0	0	0	0
ENSCAFG00845030975	3198	3170	3168	3140
ENSCAFG00845030976	16	13	31	21
ENSCAFG00845030977	5	6	5	6
ENSCAFG00845030978	0	0	0	0
ENSCAFG00845030979	0	0	0	1
ENSCAFG00845030970	26	21	31	21
ENSCAFG00845030971	0	0	0	0
ENSCAFG00845030972	0	0	0	0
ENSCAFG00845030984	0	0	0	0
ENSCAFG00845030985	780	723	750	833
ENSCAFG00845030986	0	0	0	0

ENSCAFG00845030987	0	0	0	0
ENSCAFG00845030988	134	98	127	143
ENSCAFG00845030989	384	354	490	513
ENSCAFG00845030980	41	61	21	33
ENSCAFG00845030981	0	1	2	2
ENSCAFG00845030982	0	0	0	0
ENSCAFG00845030983	0	0	0	0
ENSCAFG00845030995	0	0	1	1
ENSCAFG00845030996	0	0	0	0
ENSCAFG00845030997	961	870	854	833
ENSCAFG00845030998	836	851	851	877
ENSCAFG00845030999	18	13	9	11
ENSCAFG00845030990	0	0	0	0
ENSCAFG00845030991	0	0	0	0
ENSCAFG00845030992	1385	1285	1206	1241
ENSCAFG00845030993	0	0	0	0
ENSCAFG00845030994	273	271	288	307
ENSCAFG00845005930	0	0	0	0
ENSCAFG00845005931	2421	2474	2346	2327
ENSCAFG00845005932	5193	4940	5560	5718
ENSCAFG00845005933	344	364	315	398
ENSCAFG00845017919	0	0	0	0
ENSCAFG00845005934	182	134	199	123
ENSCAFG00845017918	352	328	355	427
ENSCAFG00845005935	0	0	0	0
ENSCAFG00845017917	0	0	0	0
ENSCAFG00845005936	0	0	0	0
ENSCAFG00845017916	0	1	0	0
ENSCAFG00845005937	132	112	135	168
ENSCAFG00845017915	1	3	2	3
ENSCAFG00845005938	0	0	0	0
ENSCAFG00845017914	4	6	13	1
ENSCAFG00845005939	2742	2704	2587	2679
ENSCAFG00845017913	0	0	0	0
ENSCAFG00845017912	0	0	0	0
ENSCAFG00845017911	0	0	0	0
ENSCAFG00845017910	0	0	0	0
ENSCAFG00845005920	1930	1721	1911	1857
ENSCAFG00845005921	395	339	320	317
ENSCAFG00845017909	0	0	0	0
ENSCAFG00845005922	362	359	364	366

ENSCAFG00845017908	684	718	446	439
ENSCAFG00845030900	0	0	0	0
ENSCAFG00845005923	2052	1954	1923	1942
ENSCAFG00845017907	0	0	1	0
ENSCAFG00845030901	0	0	1	0
ENSCAFG00845005924	0	0	0	0
ENSCAFG00845017906	0	0	0	0
ENSCAFG00845030902	403	383	349	388
ENSCAFG00845005925	0	0	0	0
ENSCAFG00845017905	6306	6098	5636	5696
ENSCAFG00845030903	253	281	249	268
ENSCAFG00845005926	96	109	116	104
ENSCAFG00845017904	32	19	24	17
ENSCAFG00845005927	158	143	180	224
ENSCAFG00845017903	211	235	236	210
ENSCAFG00845005928	3	6	13	6
ENSCAFG00845017902	0	0	0	0
ENSCAFG00845005929	494	470	419	366
ENSCAFG00845017901	0	0	0	0
ENSCAFG00845017900	1319	1277	1086	1139
ENSCAFG00845030904	0	0	0	0
ENSCAFG00845030905	0	2	0	0
ENSCAFG00845030906	1228	1107	1044	1061
ENSCAFG00845030907	74	55	50	67
ENSCAFG00845030908	0	0	0	0
ENSCAFG00845030909	4	0	0	3
ENSCAFG00845005910	3096	2821	2882	2895
ENSCAFG00845030910	6	10	10	6
ENSCAFG00845005911	489	496	560	489
ENSCAFG00845030911	0	0	0	0
ENSCAFG00845005912	1	0	2	0
ENSCAFG00845030912	0	0	0	0
ENSCAFG00845005913	0	0	0	0
ENSCAFG00845030913	448	411	348	387
ENSCAFG00845005914	1605	1576	1677	1590
ENSCAFG00845030914	219	228	276	249
ENSCAFG00845005915	0	0	0	0
ENSCAFG00845005916	28	15	16	11
ENSCAFG00845005917	2585	2466	2055	1998
ENSCAFG00845005918	21	9	4	6
ENSCAFG00845005919	324	378	210	240

ENSCAFG00845030915	0	0	0	0
ENSCAFG00845030916	133	147	118	113
ENSCAFG00845030917	3	3	0	0
ENSCAFG00845030918	0	0	0	2
ENSCAFG00845030919	45	30	34	22
ENSCAFG00845030920	0	0	0	0
ENSCAFG00845030921	0	0	0	0
ENSCAFG00845005900	216	211	188	221
ENSCAFG00845030922	0	1	0	0
ENSCAFG00845005901	0	0	0	0
ENSCAFG00845030923	295	294	303	309
ENSCAFG00845005902	882	850	835	825
ENSCAFG00845030924	0	0	0	1
ENSCAFG00845005903	111	83	86	91
ENSCAFG00845030925	604	587	536	540
ENSCAFG00845005904	0	0	0	0
ENSCAFG00845005905	1191	1174	1331	1251
ENSCAFG00845005906	1628	1498	1456	1492
ENSCAFG00845005907	972	910	817	858
ENSCAFG00845005908	0	0	0	0
ENSCAFG00845005909	374	354	301	336
ENSCAFG00845030926	0	0	0	0
ENSCAFG00845030927	0	0	0	0
ENSCAFG00845030928	0	0	1	0
ENSCAFG00845030929	0	0	0	0
ENSCAFG00845030930	232	199	251	213
ENSCAFG00845030931	0	0	0	0
ENSCAFG00845030932	3	0	2	2
ENSCAFG00845030933	0	0	0	0
ENSCAFG00845030934	0	0	0	0
ENSCAFG00845030935	0	0	0	0
ENSCAFG00845030936	109	108	92	99
ENSCAFG00845030937	1	0	0	0
ENSCAFG00845030938	0	0	0	0
ENSCAFG00845030939	0	1	0	2
ENSCAFG00845030940	0	0	0	0
ENSCAFG00845030941	3	3	1	2
ENSCAFG00845030942	0	0	0	0
ENSCAFG00845030943	0	0	0	0
ENSCAFG00845030944	22729	22191	23097	23151
ENSCAFG00845030945	0	1	3	0

ENSCAFG00845030946	79	91	95	89
ENSCAFG00845030947	0	0	0	0
ENSCAFG00845030948	639	674	699	691
ENSCAFG00845030949	0	0	0	0
ENSCAFG00845030951	634	674	685	688
ENSCAFG00845030952	0	0	0	0
ENSCAFG00845030953	0	0	1	0
ENSCAFG00845030954	0	0	0	0
ENSCAFG00845030955	0	0	0	0
ENSCAFG00845030956	732	774	662	727
ENSCAFG00845030957	773	729	660	764
ENSCAFG00845030958	0	0	0	0
ENSCAFG00845030950	0	0	0	0
ENSCAFG00845030959	0	0	0	0
ENSCAFG00845030962	46	33	48	43
ENSCAFG00845030963	46	46	53	61
ENSCAFG00845030964	2	4	3	4
ENSCAFG00845030965	0	0	0	0
ENSCAFG00845030966	0	0	0	0
ENSCAFG00845030967	61	73	31	49
ENSCAFG00845030968	228	269	243	264
ENSCAFG00845030969	7	7	18	12
ENSCAFG00845030960	0	0	0	0
ENSCAFG00845030961	0	0	0	1
ENSCAFG00845015332	0	0	0	0
ENSCAFG00845015333	2	6	2	3
ENSCAFG00845017994	1	4	0	0
ENSCAFG00845015330	2	0	5	1
ENSCAFG00845017993	28	23	23	20
ENSCAFG00845015331	0	0	0	0
ENSCAFG00845017992	0	0	0	0
ENSCAFG00845017991	602	596	440	442
ENSCAFG00845017990	0	0	0	0
ENSCAFG00845027318	0	0	0	0
ENSCAFG00845027317	0	0	0	0
ENSCAFG00845003349	61	69	73	59
ENSCAFG00845029979	0	0	0	0
ENSCAFG00845003348	406	428	436	416
ENSCAFG00845027319	52	58	52	48
ENSCAFG00845003347	2	3	2	3
ENSCAFG00845027314	0	1	0	0

ENSCAFG00845029977	532	509	467	475
ENSCAFG00845003346	182	171	184	182
ENSCAFG00845027313	0	0	0	0
ENSCAFG00845029978	2935	2961	2976	2887
ENSCAFG00845003345	25	18	23	21
ENSCAFG00845027316	104	94	88	110
ENSCAFG00845029975	0	1	0	0
ENSCAFG00845003344	0	0	0	0
ENSCAFG00845027315	11	10	10	5
ENSCAFG00845029976	0	0	0	0
ENSCAFG00845003343	1292	1228	1438	1454
ENSCAFG00845027310	2	0	3	5
ENSCAFG00845029973	51	32	31	35
ENSCAFG00845003342	4	3	4	2
ENSCAFG00845029974	2	1	1	1
ENSCAFG00845003341	0	0	0	0
ENSCAFG00845027312	61	48	38	47
ENSCAFG00845029971	1	5	0	0
ENSCAFG00845003340	273	283	349	298
ENSCAFG00845027311	0	0	0	0
ENSCAFG00845029972	0	0	0	0
ENSCAFG00845029970	0	0	0	0
ENSCAFG00845015338	0	0	0	0
ENSCAFG00845015339	0	0	0	0
ENSCAFG00845015336	0	0	0	0
ENSCAFG00845017999	0	0	0	0
ENSCAFG00845015337	0	0	0	0
ENSCAFG00845017998	0	0	4	1
ENSCAFG00845015334	0	0	0	0
ENSCAFG00845017997	0	0	1	3
ENSCAFG00845015335	1829	1860	1859	1799
ENSCAFG00845017996	0	0	0	0
ENSCAFG00845015321	454	366	440	373
ENSCAFG00845017984	0	0	0	0
ENSCAFG00845015322	136	123	136	105
ENSCAFG00845017983	0	0	0	0
ENSCAFG00845017982	722	745	708	722
ENSCAFG00845015320	1671	1655	1860	2087
ENSCAFG00845017981	934	873	1025	1047
ENSCAFG00845017980	0	0	0	0
ENSCAFG00845027307	0	0	0	0

ENSCAFG00845027306	564	515	551	563
ENSCAFG00845027309	17	12	7	15
ENSCAFG00845029968	0	0	0	0
ENSCAFG00845003359	0	0	0	0
ENSCAFG00845027308	0	0	0	0
ENSCAFG00845029969	0	0	0	0
ENSCAFG00845003358	0	0	0	0
ENSCAFG00845027303	253	271	443	433
ENSCAFG00845029966	1	4	0	1
ENSCAFG00845003357	111	96	159	143
ENSCAFG00845027302	32	26	31	26
ENSCAFG00845029967	44	57	36	40
ENSCAFG00845003356	0	0	0	0
ENSCAFG00845027305	627	523	606	699
ENSCAFG00845029964	0	0	0	0
ENSCAFG00845003355	571	529	670	673
ENSCAFG00845027304	0	0	0	0
ENSCAFG00845029965	0	0	0	2
ENSCAFG00845003354	0	0	0	0
ENSCAFG00845029962	0	0	0	0
ENSCAFG00845003353	2183	2202	2022	2054
ENSCAFG00845029963	0	0	1	1
ENSCAFG00845003352	0	0	0	0
ENSCAFG00845027301	14	13	11	3
ENSCAFG00845029960	0	0	0	0
ENSCAFG00845003351	0	0	0	0
ENSCAFG00845029961	0	0	0	0
ENSCAFG00845003350	723	684	542	570
ENSCAFG00845015329	0	0	0	0
ENSCAFG00845015327	0	0	0	0
ENSCAFG00845015328	104	69	94	80
ENSCAFG00845017989	3	3	5	5
ENSCAFG00845015325	0	0	0	0
ENSCAFG00845017988	1152	1086	1296	1209
ENSCAFG00845015326	260	308	490	538
ENSCAFG00845017987	1001	961	846	854
ENSCAFG00845015323	35	57	68	58
ENSCAFG00845017986	0	0	0	0
ENSCAFG00845015324	0	1	0	0
ENSCAFG00845017985	3	1	0	2
ENSCAFG00845015310	98	79	123	111

ENSCAFG00845017973	0	0	0	0
ENSCAFG00845015311	38	36	29	29
ENSCAFG00845017972	8	13	1	1
ENSCAFG00845017971	1	1	2	0
ENSCAFG00845017970	0	0	0	1
ENSCAFG00845003329	42	25	24	31
ENSCAFG00845029959	297	315	317	322
ENSCAFG00845003328	306	230	249	230
ENSCAFG00845003327	0	0	0	0
ENSCAFG00845029957	0	0	0	0
ENSCAFG00845003326	529	495	548	520
ENSCAFG00845005990	0	0	0	0
ENSCAFG00845029958	0	0	1	0
ENSCAFG00845003325	10	8	7	10
ENSCAFG00845005991	301	304	119	124
ENSCAFG00845029955	598	631	609	629
ENSCAFG00845003324	0	0	0	0
ENSCAFG00845005992	260	318	208	156
ENSCAFG00845029956	0	0	0	0
ENSCAFG00845003323	881	804	670	818
ENSCAFG00845005993	647	674	670	657
ENSCAFG00845029953	41	27	41	23
ENSCAFG00845003322	2085	2069	1889	1971
ENSCAFG00845005994	177	196	70	54
ENSCAFG00845029954	2514	2430	2419	2486
ENSCAFG00845003321	5	5	4	4
ENSCAFG00845005995	14	10	18	16
ENSCAFG00845029951	0	0	0	0
ENSCAFG00845003320	0	0	0	1
ENSCAFG00845005996	3	7	4	4
ENSCAFG00845029952	1040	940	896	901
ENSCAFG00845005997	7470	7026	6679	6599
ENSCAFG00845005998	0	0	0	0
ENSCAFG00845029950	0	0	0	0
ENSCAFG00845005999	0	0	0	0
ENSCAFG00845015318	3	0	0	0
ENSCAFG00845015319	0	0	0	0
ENSCAFG00845015316	0	0	0	0
ENSCAFG00845017979	0	0	0	0
ENSCAFG00845015317	0	0	0	0
ENSCAFG00845017978	8	6	7	8

ENSCAFG00845015314	0	0	0	0
ENSCAFG00845017977	0	0	0	0
ENSCAFG00845015315	3463	3217	3202	3227
ENSCAFG00845017976	0	2	0	2
ENSCAFG00845015312	0	0	0	0
ENSCAFG00845017975	3562	3559	3486	3423
ENSCAFG00845015313	0	0	0	0
ENSCAFG00845017974	1520	1520	1333	1455
ENSCAFG00845017962	0	0	0	0
ENSCAFG00845015300	40	48	37	51
ENSCAFG00845017961	1481	1387	1205	1291
ENSCAFG00845017960	0	0	0	0
ENSCAFG00845029948	0	0	0	0
ENSCAFG00845003339	0	0	0	0
ENSCAFG00845029949	0	0	0	0
ENSCAFG00845003338	21	14	7	11
ENSCAFG00845029946	31	20	27	9
ENSCAFG00845003337	0	0	0	0
ENSCAFG00845029947	93	126	112	146
ENSCAFG00845003336	525	541	476	470
ENSCAFG00845005980	0	0	2	0
ENSCAFG00845029944	0	0	0	0
ENSCAFG00845003335	1910	1803	1704	1872
ENSCAFG00845005981	7	3	3	3
ENSCAFG00845029945	56	44	49	49
ENSCAFG00845003334	105	111	89	92
ENSCAFG00845005982	40	39	38	51
ENSCAFG00845029942	13	4	6	6
ENSCAFG00845003333	960	944	1017	1055
ENSCAFG00845005983	431	410	452	435
ENSCAFG00845029943	0	2	0	0
ENSCAFG00845003332	378	389	364	348
ENSCAFG00845005984	2	1	4	2
ENSCAFG00845029940	531	437	400	437
ENSCAFG00845003331	693	655	632	725
ENSCAFG00845005985	10	3	9	8
ENSCAFG00845029941	0	0	0	0
ENSCAFG00845003330	6	4	0	2
ENSCAFG00845005986	0	0	0	0
ENSCAFG00845005987	0	0	0	0
ENSCAFG00845005988	0	0	0	0

ENSCAFG00845005989	40	31	44	31
ENSCAFG00845015309	351	333	318	319
ENSCAFG00845015307	2	5	2	1
ENSCAFG00845015308	0	0	0	0
ENSCAFG00845017969	59	41	90	113
ENSCAFG00845015305	0	0	0	0
ENSCAFG00845017968	1320	1328	1371	1482
ENSCAFG00845015306	0	0	0	0
ENSCAFG00845017967	4	4	3	2
ENSCAFG00845015303	0	0	0	0
ENSCAFG00845017966	5672	5382	5014	5076
ENSCAFG00845015304	61	59	42	39
ENSCAFG00845017965	72	82	68	90
ENSCAFG00845015301	564	625	603	649
ENSCAFG00845017964	0	0	1	0
ENSCAFG00845015302	1	1	0	0
ENSCAFG00845017963	0	0	0	0
ENSCAFG00845017951	0	0	0	0
ENSCAFG00845017950	0	0	0	0
ENSCAFG00845003309	177	125	170	183
ENSCAFG00845029939	83	74	82	78
ENSCAFG00845003308	2269	2046	2246	2406
ENSCAFG00845003307	62	71	74	69
ENSCAFG00845029937	12	2	4	8
ENSCAFG00845003306	0	0	0	1
ENSCAFG00845029938	923	832	858	908
ENSCAFG00845003305	166	153	155	197
ENSCAFG00845029935	96	89	80	93
ENSCAFG00845003304	366	381	298	299
ENSCAFG00845029936	1311	1292	1150	1160
ENSCAFG00845003303	17	8	3	6
ENSCAFG00845029933	0	0	0	0
ENSCAFG00845003302	0	2	0	0
ENSCAFG00845005970	445	381	398	482
ENSCAFG00845029934	4	14	9	11
ENSCAFG00845003301	1331	1334	1172	1343
ENSCAFG00845005971	30	44	35	44
ENSCAFG00845029931	7	8	11	10
ENSCAFG00845003300	5	6	6	6
ENSCAFG00845005972	805	761	638	650
ENSCAFG00845029932	1	2	2	3

ENSCAFG00845005973	239	224	266	235
ENSCAFG00845005974	4	0	2	0
ENSCAFG00845029930	0	1	5	0
ENSCAFG00845005975	2617	2414	1906	1990
ENSCAFG00845005976	23	29	22	22
ENSCAFG00845005977	299	256	303	317
ENSCAFG00845005978	0	0	0	0
ENSCAFG00845005979	261	227	243	232
ENSCAFG00845017959	0	0	0	0
ENSCAFG00845017958	143	118	105	114
ENSCAFG00845017956	10	8	12	19
ENSCAFG00845017955	0	0	0	0
ENSCAFG00845017954	0	0	0	0
ENSCAFG00845017953	0	0	0	0
ENSCAFG00845017952	0	0	0	0
ENSCAFG00845017940	79	82	52	62
ENSCAFG00845029928	924	812	871	860
ENSCAFG00845003319	762	767	763	699
ENSCAFG00845029929	7	9	10	13
ENSCAFG00845003318	0	0	0	0
ENSCAFG00845029926	60	41	36	55
ENSCAFG00845003317	2	0	3	0
ENSCAFG00845029927	0	0	0	0
ENSCAFG00845003316	0	0	0	0
ENSCAFG00845029924	1961	1936	1764	1804
ENSCAFG00845003315	657	551	608	697
ENSCAFG00845029925	0	0	0	0
ENSCAFG00845003314	1198	1085	1141	1109
ENSCAFG00845029922	1314	1198	1322	1299
ENSCAFG00845003313	0	0	0	0
ENSCAFG00845029923	0	0	0	0
ENSCAFG00845003312	18	14	5	17
ENSCAFG00845005960	19	9	7	17
ENSCAFG00845029920	423	437	421	467
ENSCAFG00845003311	714	694	662	699
ENSCAFG00845005961	222	202	157	147
ENSCAFG00845029921	0	0	0	0
ENSCAFG00845003310	544	539	496	511
ENSCAFG00845005962	271	291	283	304
ENSCAFG00845005963	1	0	0	0
ENSCAFG00845005964	400	401	361	404

ENSCAFG00845005965	0	1	0	1
ENSCAFG00845005966	872	819	724	747
ENSCAFG00845005967	198	222	237	276
ENSCAFG00845005968	0	0	0	0
ENSCAFG00845005969	1952	1791	1877	1809
ENSCAFG00845017949	196	192	195	216
ENSCAFG00845017948	0	0	0	0
ENSCAFG00845017947	39	40	45	53
ENSCAFG00845017946	1	0	0	0
ENSCAFG00845017945	670	575	660	607
ENSCAFG00845017944	0	0	0	0
ENSCAFG00845017943	307	322	300	328
ENSCAFG00845017942	0	2	0	1
ENSCAFG00845017941	0	0	0	0
ENSCAFG00845029919	1	2	4	10
ENSCAFG00845029917	0	0	0	0
ENSCAFG00845029918	971	941	1022	971
ENSCAFG00845029915	3750	3621	3717	3837
ENSCAFG00845029916	790	715	653	646
ENSCAFG00845029913	4	14	3	6
ENSCAFG00845029914	2817	2673	2645	2625
ENSCAFG00845029911	0	0	0	0
ENSCAFG00845029912	9	4	2	7
ENSCAFG00845005950	5440	5098	5258	5204
ENSCAFG00845029910	154	151	139	125
ENSCAFG00845005951	24	12	6	13
ENSCAFG00845005952	0	0	0	0
ENSCAFG00845005953	7	11	7	4
ENSCAFG00845005954	3415	3365	3327	3181
ENSCAFG00845005955	0	0	0	0
ENSCAFG00845005956	192	170	185	210
ENSCAFG00845005957	900	842	767	839
ENSCAFG00845017939	668	663	642	617
ENSCAFG00845005958	0	0	0	0
ENSCAFG00845017938	0	0	0	0
ENSCAFG00845005959	290	299	277	233
ENSCAFG00845017936	0	0	0	0
ENSCAFG00845017935	0	0	0	0
ENSCAFG00845017934	0	0	0	0
ENSCAFG00845017933	11	10	5	12
ENSCAFG00845017932	0	0	0	0

ENSCAFG00845017931	52	40	32	30
ENSCAFG00845017930	22	14	15	11
ENSCAFG00845029908	880	885	871	937
ENSCAFG00845029909	34	70	32	34
ENSCAFG00845029906	847	779	689	716
ENSCAFG00845029907	3059	2946	2894	2921
ENSCAFG00845029904	0	0	0	1
ENSCAFG00845029905	1527	1438	1474	1536
ENSCAFG00845029902	2	0	0	0
ENSCAFG00845029903	1319	1163	1394	1464
ENSCAFG00845029900	0	0	0	0
ENSCAFG00845029901	0	0	0	0
ENSCAFG00845005940	0	0	0	0
ENSCAFG00845005941	1	4	0	1
ENSCAFG00845005942	2052	2016	2095	2239
ENSCAFG00845005943	0	0	0	0
ENSCAFG00845005944	481	436	472	503
ENSCAFG00845005945	0	0	0	0
ENSCAFG00845017929	277	259	264	288
ENSCAFG00845005946	864	882	905	883
ENSCAFG00845017928	69	57	65	69
ENSCAFG00845005947	0	0	0	0
ENSCAFG00845017927	0	0	0	0
ENSCAFG00845005948	605	592	809	739
ENSCAFG00845017926	0	0	0	0
ENSCAFG00845005949	823	769	784	870
ENSCAFG00845017925	0	0	0	0
ENSCAFG00845017924	2	0	0	0
ENSCAFG00845017923	0	0	0	0
ENSCAFG00845017922	0	0	0	0
ENSCAFG00845017921	109	85	80	115
ENSCAFG00845017920	16	9	22	32
ENSCAFG00845027497	355	360	404	367
ENSCAFG00845027496	0	0	0	0
ENSCAFG00845027499	1924	1985	1601	1733
ENSCAFG00845027498	5	3	8	9
ENSCAFG00845027493	19	15	16	8
ENSCAFG00845027492	21237	20694	16173	16302
ENSCAFG00845027495	0	1	0	0
ENSCAFG00845027494	500	436	457	458
ENSCAFG00845027491	0	0	0	0

ENSCAFG00845027490	3787	3455	3504	3649
ENSCAFG00845027489	1584	1550	1388	1508
ENSCAFG00845027486	380	358	358	377
ENSCAFG00845027485	6	2	5	6
ENSCAFG00845027488	0	0	0	0
ENSCAFG00845027487	5080	4910	5350	5578
ENSCAFG00845027482	0	0	0	0
ENSCAFG00845027481	2224	2237	2031	2064
ENSCAFG00845027484	0	0	0	0
ENSCAFG00845027483	0	0	1	0
ENSCAFG00845027480	0	0	0	0
ENSCAFG00845015497	787	738	689	755
ENSCAFG00845015498	4	4	3	2
ENSCAFG00845015495	829	775	784	838
ENSCAFG00845015496	0	0	0	0
ENSCAFG00845015493	422	412	401	503
ENSCAFG00845015494	0	0	0	0
ENSCAFG00845015491	0	0	0	0
ENSCAFG00845015492	10	16	10	8
ENSCAFG00845015490	0	0	0	0
ENSCAFG00845027478	21	11	13	21
ENSCAFG00845027475	1114	1037	985	972
ENSCAFG00845027477	7	3	2	3
ENSCAFG00845027476	0	0	0	0
ENSCAFG00845027471	0	3	2	4
ENSCAFG00845027470	10	11	12	9
ENSCAFG00845027473	0	0	1	1
ENSCAFG00845027472	239	224	218	202
ENSCAFG00845015499	1	0	0	0
ENSCAFG00845015486	0	0	0	0
ENSCAFG00845015487	0	0	0	0
ENSCAFG00845015484	0	0	0	0
ENSCAFG00845015485	0	0	0	0
ENSCAFG00845015482	0	0	0	0
ENSCAFG00845015483	700	674	710	759
ENSCAFG00845015480	1	0	0	4
ENSCAFG00845015481	0	0	0	0
ENSCAFG00845027468	0	0	0	0
ENSCAFG00845027467	0	0	0	0
ENSCAFG00845027469	0	0	0	0
ENSCAFG00845027464	6	2	3	3

ENSCAFG00845027463	0	2	0	0
ENSCAFG00845027465	1	0	0	0
ENSCAFG00845027460	2393	2193	2050	2116
ENSCAFG00845027462	0	0	0	0
ENSCAFG00845027461	2273	2077	2414	2510
ENSCAFG00845015488	112	122	120	134
ENSCAFG00845015489	341	373	376	405
ENSCAFG00845015475	0	0	0	0
ENSCAFG00845015476	942	1037	984	1030
ENSCAFG00845015473	0	0	0	0
ENSCAFG00845015474	675	639	672	673
ENSCAFG00845015471	0	0	0	0
ENSCAFG00845015472	1	1	0	1
ENSCAFG00845015470	0	1	0	0
ENSCAFG00845027457	0	0	0	0
ENSCAFG00845003489	1	2	3	1
ENSCAFG00845027456	0	1	0	0
ENSCAFG00845003488	0	0	0	0
ENSCAFG00845003487	216	164	185	211
ENSCAFG00845027458	101	94	102	120
ENSCAFG00845003486	38	40	42	57
ENSCAFG00845027453	23	18	11	12
ENSCAFG00845003485	0	4	3	0
ENSCAFG00845027452	140	123	97	103
ENSCAFG00845003484	0	0	0	0
ENSCAFG00845027455	2454	2254	2495	2478
ENSCAFG00845003483	7	3	11	6
ENSCAFG00845027454	0	3	6	4
ENSCAFG00845003482	0	0	0	2
ENSCAFG00845003481	0	0	0	1
ENSCAFG00845003480	4603	4436	4319	4486
ENSCAFG00845027451	0	0	0	1
ENSCAFG00845027450	0	0	0	0
ENSCAFG00845015479	960	806	919	864
ENSCAFG00845015477	24	22	13	6
ENSCAFG00845015478	320	314	259	270
ENSCAFG00845015464	0	0	0	0
ENSCAFG00845015465	0	0	0	0
ENSCAFG00845015462	41	34	29	59
ENSCAFG00845015463	0	0	0	0
ENSCAFG00845015460	759	789	748	767

ENSCAFG00845015461	0	0	0	0
ENSCAFG00845027449	3301	3209	3153	3217
ENSCAFG00845027446	44	38	45	44
ENSCAFG00845027445	0	0	0	0
ENSCAFG00845003499	6	0	2	3
ENSCAFG00845027448	0	0	0	0
ENSCAFG00845003498	1529	1457	1546	1535
ENSCAFG00845027447	77	54	67	68
ENSCAFG00845003497	481	540	558	509
ENSCAFG00845027442	360	341	296	297
ENSCAFG00845003496	0	0	0	0
ENSCAFG00845027441	1093	1000	1156	1058
ENSCAFG00845003495	1730	1773	1249	1170
ENSCAFG00845027444	0	0	0	0
ENSCAFG00845003494	3711	3691	3325	3561
ENSCAFG00845027443	113	101	246	240
ENSCAFG00845003493	0	0	0	0
ENSCAFG00845003492	520	474	483	470
ENSCAFG00845003491	1433	1286	1135	1188
ENSCAFG00845027440	11	8	16	7
ENSCAFG00845003490	0	0	0	0
ENSCAFG00845015468	0	0	0	0
ENSCAFG00845015469	0	0	0	0
ENSCAFG00845015466	2	7	1	4
ENSCAFG00845015467	0	0	0	0
SPARC	1	0	2604	2417
ENSCAFG00845015453	8	13	9	8
ENSCAFG00845015454	0	0	0	0
ENSCAFG00845015451	0	0	0	0
ENSCAFG00845015452	603	646	679	628
ENSCAFG00845015450	1309	1271	1455	1520
ENSCAFG00845027439	0	0	0	1
ENSCAFG00845027438	1369	1403	1387	1421
ENSCAFG00845003469	334	309	339	335
ENSCAFG00845003468	886	832	765	785
ENSCAFG00845027435	1354	1464	1383	1367
ENSCAFG00845003467	0	0	0	0
ENSCAFG00845027434	0	0	0	0
ENSCAFG00845003466	7	7	4	7
ENSCAFG00845027437	31	19	13	11
ENSCAFG00845003465	996	1001	1133	1115

ENSCAFG00845027436	591	647	629	609
ENSCAFG00845003464	0	0	0	0
ENSCAFG00845027431	0	0	0	0
ENSCAFG00845003463	2175	2127	2263	2162
ENSCAFG00845027430	0	0	0	0
ENSCAFG00845003462	287	288	320	342
ENSCAFG00845027433	455	434	532	541
ENSCAFG00845003461	0	0	0	0
ENSCAFG00845027432	114	102	124	122
ENSCAFG00845003460	68	51	68	70
ENSCAFG00845015459	2803	2537	3019	2918
ENSCAFG00845015457	358	344	297	317
ENSCAFG00845015458	5	2	3	1
ENSCAFG00845015455	0	0	0	0
ENSCAFG00845015456	3742	3718	3671	3849
ENSCAFG00845015442	0	0	0	0
ENSCAFG00845015443	2	1	4	2
ENSCAFG00845015440	0	0	0	0
ENSCAFG00845015441	0	0	2	1
ENSCAFG00845027428	2827	2530	2764	2643
ENSCAFG00845027427	0	0	0	0
ENSCAFG00845027429	0	0	0	0
ENSCAFG00845003479	0	0	0	0
ENSCAFG00845027424	482	460	405	411
ENSCAFG00845003478	172	157	112	130
ENSCAFG00845027423	0	0	0	0
ENSCAFG00845003477	779	799	735	798
ENSCAFG00845027426	0	0	0	0
ENSCAFG00845003476	5	8	10	0
ENSCAFG00845027425	19	20	21	7
ENSCAFG00845003475	46	43	42	40
ENSCAFG00845027420	409	394	412	452
ENSCAFG00845003474	830	783	656	618
ENSCAFG00845003473	0	0	2	0
ENSCAFG00845027422	391	410	438	473
ENSCAFG00845003472	1040	992	959	1037
ENSCAFG00845027421	0	0	0	0
ENSCAFG00845003471	861	715	715	823
ENSCAFG00845003470	2	0	2	0
ENSCAFG00845015448	0	0	1	0
ENSCAFG00845015449	0	0	0	0

ENSCAFG00845015446	0	0	0	0
ENSCAFG00845015447	0	0	0	0
ENSCAFG00845015444	0	0	0	0
ENSCAFG00845015445	1544	1499	1537	1506
ENSCAFG00845015431	2	3	5	6
ENSCAFG00845015432	416	394	393	438
ENSCAFG00845015430	1	3	1	2
ENSCAFG00845027417	1	0	3	0
ENSCAFG00845003449	80	64	45	69
ENSCAFG00845027416	1182	1108	1196	1178
ENSCAFG00845003448	0	3	1	3
ENSCAFG00845027419	3248	3233	3149	3061
ENSCAFG00845003447	10	15	15	10
ENSCAFG00845027418	2	0	6	10
ENSCAFG00845003446	1483	1458	1864	1929
ENSCAFG00845027413	1	0	0	0
ENSCAFG00845003445	857	812	751	728
ENSCAFG00845027412	0	0	0	0
ENSCAFG00845003444	236	253	256	307
ENSCAFG00845027415	2075	2001	2256	2368
ENSCAFG00845003443	284	250	267	312
ENSCAFG00845027414	159	139	154	130
ENSCAFG00845003442	386	362	297	358
ENSCAFG00845003441	856	783	789	790
ENSCAFG00845003440	0	0	0	0
ENSCAFG00845027411	852	875	603	569
ENSCAFG00845027410	1882	1806	1732	1842
ENSCAFG00845015439	0	0	0	0
ENSCAFG00845015437	62	46	57	84
ENSCAFG00845015438	0	0	0	0
ENSCAFG00845015435	0	0	0	0
ENSCAFG00845015436	349	365	351	332
ENSCAFG00845015433	0	0	0	0
ENSCAFG00845015434	0	0	0	0
ENSCAFG00845015420	1610	1586	1733	1840
ENSCAFG00845015421	0	0	0	0
ENSCAFG00845027409	0	0	0	0
ENSCAFG00845027405	0	0	0	0
ENSCAFG00845003459	1546	1450	1421	1366
ENSCAFG00845027408	0	0	0	0
ENSCAFG00845003458	0	0	1	0

ENSCAFG00845027407	338	382	342	334
ENSCAFG00845003457	143	133	138	144
ENSCAFG00845027402	39	61	55	70
ENSCAFG00845003456	2502	2341	2488	2734
ENSCAFG00845027401	0	0	0	0
ENSCAFG00845003455	0	0	0	0
ENSCAFG00845027404	0	0	0	0
ENSCAFG00845003454	861	772	920	863
ENSCAFG00845027403	0	0	0	0
ENSCAFG00845003453	0	0	0	0
ENSCAFG00845003452	510	542	594	507
ENSCAFG00845003451	0	0	0	0
ENSCAFG00845027400	20	13	32	19
ENSCAFG00845003450	0	0	0	0
ENSCAFG00845015428	0	0	0	0
ENSCAFG00845015429	0	0	0	0
ENSCAFG00845015426	0	2	0	0
ENSCAFG00845015427	0	0	0	0
ENSCAFG00845015424	5	1	5	2
ENSCAFG00845015425	0	0	0	0
ENSCAFG00845015422	0	0	0	0
ENSCAFG00845015423	378	337	308	327
ENSCAFG00845015410	0	0	0	1
ENSCAFG00845003429	0	0	0	0
ENSCAFG00845003428	0	0	0	0
ENSCAFG00845003427	0	0	0	0
ENSCAFG00845003426	3249	3107	3274	3403
ENSCAFG00845003425	279	280	266	297
ENSCAFG00845003424	3221	3125	2807	2906
ENSCAFG00845003423	1086	1122	989	932
ENSCAFG00845003422	15	20	23	19
ENSCAFG00845003421	2	0	6	3
ENSCAFG00845003420	13	4	14	6
ENSCAFG00845015419	326	302	267	275
ENSCAFG00845015417	0	0	0	0
ENSCAFG00845015418	0	0	0	0
ENSCAFG00845015415	353	352	330	324
ENSCAFG00845015416	1	1	0	2
ENSCAFG00845015413	0	0	0	0
ENSCAFG00845015414	1103	1038	998	1052
ENSCAFG00845015411	0	0	0	0

ENSCAFG00845015412	6	10	4	2
ENSCAFG00845003439	2	3	4	0
ENSCAFG00845003438	204	246	259	223
ENSCAFG00845003437	745	682	702	723
ENSCAFG00845003436	243	217	227	191
ENSCAFG00845003435	0	0	0	0
ENSCAFG00845003434	0	0	0	0
ENSCAFG00845003433	0	0	2	0
ENSCAFG00845003432	398	376	344	354
ENSCAFG00845003431	441	465	429	392
ENSCAFG00845003430	2	0	2	0
ENSCAFG00845015408	0	0	0	0
ENSCAFG00845015409	54	38	41	49
ENSCAFG00845015406	0	0	5	0
ENSCAFG00845015407	819	877	776	731
ENSCAFG00845015404	0	0	0	0
ENSCAFG00845015405	0	0	0	0
ENSCAFG00845015402	1046	1081	854	995
ENSCAFG00845015403	21	12	19	22
ENSCAFG00845015400	0	0	0	0
ENSCAFG00845015401	2494	2435	2690	2786
ENSCAFG00845003409	459	437	442	431
ENSCAFG00845003408	128	95	62	67
ENSCAFG00845003407	1958	1796	1514	1540
ENSCAFG00845003406	9113	8840	8939	8975
ENSCAFG00845003405	0	0	0	0
ENSCAFG00845003404	900	909	891	878
ENSCAFG00845003403	2	0	2	1
ENSCAFG00845003402	28	31	36	71
ENSCAFG00845003401	2594	2480	2363	2395
ENSCAFG00845003400	0	0	0	0
ENSCAFG00845003419	0	0	0	0
ENSCAFG00845003418	3	1	2	3
ENSCAFG00845003417	0	0	4	0
ENSCAFG00845003416	8	13	6	3
ENSCAFG00845003415	3	0	1	0
ENSCAFG00845003414	337	297	325	311
ENSCAFG00845003413	0	0	0	1
ENSCAFG00845003412	117	96	95	105
ENSCAFG00845003411	376	353	316	322
ENSCAFG00845003410	3	7	0	1
